# *PLOS ONE* 2017 Reviewer and Editorial Board Thank You

**DOI:** 10.1371/journal.pone.0194158

**Published:** 2018-03-15

PLOS and the *PLOS ONE* team would like to express our appreciation for our academic editors, guest editors, and reviewers who contributed to the peer-review process this past year. We are indebted to volunteers who generously give their time and expertise to thoroughly review research and advance Open Access. In 2017, *PLOS ONE* received the help of over 4,400 Editorial Board members and 500 guest editors to curate nearly 45,000 submissions. Along with the participation of 63,000 reviewers, we were able to publish more than 20,000 articles with meaningful and impactful results.

The names of our 2017 editors that handled submitted manuscripts appear in the Supporting Information as S1 Editor List and as S1 Guest Editor List. Our reviewers appear in the Supporting Information as S1–S5 Reviewer List. With genuine gratitude, we thank you all for your dedicated support of *PLOS ONE* and our efforts to promote Open Science, thereby contributing to the scientific community as a whole. Thank you all.

## Supporting information

S1 Editor List Alphabetical list of 2017 *PLOS ONE* Editorial Board Members. (PDF)

S1 Guest Editor List Alphabetical list of 2017 *PLOS ONE* Guest Editors. (PDF)

S1 Reviewer List Alphabetical list of 2017 *PLOS ONE* peer reviewers (A-D).
(PDF)

S2 Reviewer List Alphabetical list of 2017 *PLOS ONE* peer reviewers (E-J).
(PDF)

S3 Reviewer List Alphabetical list of 2017 *PLOS ONE* peer reviewers (K-N).
(PDF)

S4 Reviewer List Alphabetical list of 2017 *PLOS ONE* peer reviewers (O-S).
(PDF)

S5 Reviewer List Alphabetical list of 2017 *PLOS ONE* peer reviewers (T-Z).
(PDF)
