# Supplementary material for: PLOS ONE 2017 Reviewer and Editorial Board Thank You
Source: PLoS One. 2018 Mar 15;13(3):e0194158. doi: 10.1371/journal.pone.0194158 (PMC5854357; doi:10.1371/journal.pone.0194158)
Supplement: S1 Editor List — (PDF) [file pone.0194158.s001.pdf]

PLOS ONE would like to thank all those who served on the journal's Editorial Board in 2017:

Katriina Aalto-Setala
Alejandro Aballay
Siddique A. Abbasi
Ashraf B. Abdel-Naim
Amar Abderrahmani
Zaid Abdo
Amir Abdollahi
Hideharu Abe
Keiko Abe
Takeru Abe
Pasquale Abete
Alash'le G. Abimiku
Zsolt Ablonczy
Abdelilah Aboussekhra
Thomas Abraham
William R. Abrams
Yael Abreu-Villaça
Osama Ali Abulseoud
Serena Aceto
Varenyam Achal
Krishnendu Acharya
Alessandro Achilli
Ted S. Acott
Rodney D. Adam
Andrew Adamatzky
Jean Adams
Michelle M. Adams
Elsa Addessi
Christina L. Addison
Zach N. Adelman
Salvatore Adinolfi
Paul A. Adlard
Christof Markus Aegerter
Kamyar Afarinkia
Gijs B. Afink
Farhat Afrin
Sudha Agarwal
Ritesh Agarwal
Pradeep K. Agarwal
Gabriel Agbor
Ashutosh Nath Aggarwal
Irina U. Agoulnik
Christian Agrillo
Francisco Aguayo
Marta Agudo-Barriuso
Marcia B. Aguila
Ruben Claudio Aguilar
Abelardo I. Aguilera
Helmut Ahammer
Golo Ahlenstiel
Muzamil Ahmad
Shama Ahmad
Rasheed Ahmad
Aftab Ahmad
Aamir Ahmad
Shawn Ahmed
Niyaz Ahmed
Rashida Ahmed
S. Ashraf Ahmed
Aamir Ahmed
Byeong-Cheol Ahn
Ingo Ahrens
Sunil K. Ahuja
Jyrki Ahveninen
Xun Ai
Jinglu Ai
Tomohiko Ai
Elena Aikawa
Mohammed Akaaboune
Tadayuki Akagi
Fadi G. Akar
Yoshiki Akatsuka
Mohammad R. Akbari
Suminori Akiba
Tomi F. Akinyemiju
Taishin Akiyama

Orhan Aktas
Elkan G. Akyürek
Jumana Yousuf Al-Aama
Claude Alain
Nehad M. Alajez
Mohamad Alameddine
Arsham Alamian
William Alazawi
Emidio Albertini
Urs Albrecht
Benedicte Riber Albrectsen
Rafael Aldabe
Alexander V. Alekseyenko
Branko Aleksic
André Aleman
Marià Alemany
Riccardo Alessandro
Sheila Alexander
Lena Alexopoulou
Kristin J. Al-Ghoul
Mohammad Ali
Jauhar Ali
Raghib Ali
Mani Alikhani
Adel M. Alimi
Alberto Aliseda
Anna Alisi
Robin Allaby
Irving Coy Allen
Benjamin Lee Allen
Philip Allen
Rachel Louise Allen
Bernadette Allinquant
Alejandro Almarza
Adelaide Almeida
Graca D. Almeida-Porada
Marta M. Alonso
Gianfranco D. Alpini
Thierry Alquier
Eduardo G. Altmann
Christian Friedrich Altmann
Deborah A. Altomare
Inés Álvarez
Diego Alvarez de la Rosa
Gualtiero Alvisi
Stephen E. Alway
Sara Amancio
Salomon Amar
Luís A. Nunes Amaral
Frederic Amblard
Zandrea Ambrose
Roberto Ambrosini
Carlos E. Ambrósio
Thierry Amédée
Roberto Amendola
A. R. M. Ruhul Amin
Nicola Amodio
Lingling An
Madhur Anand
Shrikant Anant
Kurt I. Anderson
Andrew Anderson
Michael G. Anderson
Rozalyn M. Anderson
David R. Andes
Claudia D. Andl
Usha P. Andley
Giuseppe Andò
Shaida A. Andrabi
Paula B. Andrade
Miguel A. Andrade-Navarro
Isabelle Andre
Frédéric André
Graciela Andrei
Zane B. Andrews
Ioannis P. Androulakis
Hagen Andruszkow
Juan A. Añel
Peter C. Angeletti
Adriano Angelucci
Philip Anglewicz
Arga Chandrashekar Anil
Muna Anjum
Lucio Annunziato
Aftab A. Ansari
Andrea Antal

Ruby John Anto
Alessandro Antonietti
Christophe Antoniewski
Antony Nicodemus Antoniou
Maxim Antopolsky
Ichio Aoki
Cristian Apetrei
Marco Apollonio
Vasu D. Appanna
Silke Appel
Hossam M. M. Arafa
Ken Arai
Gururaj Arakeri
Wagner L. Araujo
Nuno Araujo
Filippos A. Aravanopoulos
Natarajan Aravindan
Jack Arbiser
Luca Paolo Ardigò
Thomas Arendt
Ramon Arens
Ana Paula Arez
Oscar Arias-Carrion
Kevin K. Ariën
Hiroyoshi Ariga
Mehrdad Arjomandi
Dan E. Arking
Robert Alan Arkowitz
Raphaël Arlettaz
Ines Armando
Cristina Armas
Anna R. Armitage
Derek Henry Arnold
Dawn L. Arnold
Danilo Arnone
Ricardo Aroca
Raffi V. Aroian
Pankaj Kumar Arora
Rajesh Arora
Valder R. Arruda
Ruben Artero
Wolfgang Arthofer
Thiruma V. Arumugam
Atsushi Asakura
Masato Asanuma
Hitoshi Ashida
Ali A. Ashkar
Hassan Ashktorab
Hossam M. Ashour
Nick Ashton
Monika R. Asnani
Jane Elizabeth Aspell
Pontus Aspenstrom
Patricia Aspichueta
Shervin Assassi
Eric Asselin
Piia Susanna Astikainen
Dhammika Nanda Atapattu
Stephen L. Atkin
Bernard Attali
Houssam Attoui
Paul J. Atzberger
Shannon Wing-Ngor Au
Harald Auge
Tim D. Aumann
Ferdinando Auricchio
Valeria Avdoshina
Alessio Avenanti
Matias A. Avila
Jesus M. Avilés
Massimo Avoli
Hani A. Awad
Belay T. Ayele
Nukhet Aykin-Burns
Eduard Ayuso
Samy A. Azer
Luciano Cesar Pontes Azevedo
Ramy K. Aziz
Jan P. A. Baak
Horacio Bach
Michael P. Bachmann
Markus M. Bachschmid
Peter H. Backx
Tudor C. Badea
Joel S. Bader
Michael Bader

Jonathan H. Badger
Christopher Bae
Kwang-Hyun Baek
Maria R. Baer
Andrew Baggaley
Carolyn J. Baglole
Serena M. Bagnasco
Yong-Sun Bahn
Guihua Bai
Xue-Feng Bai
Yidong Bai
Paul N. Baird
Niranjan Baisakh
Vladimir B. Bajic
Ruth E. Baker
Michelle L. Baker
Jennifer L. Baker
Chandra Shekhar Bakshi
Sambit Bakshi
Jacint Balaguer
Kithiganahalli N. Balaji
Ramesh Balasubramaniam
Robert F. Baldwin
Raffaella Balestrini
Mitchell F. Balish
Esteban Ballestar
Daniel J. Ballhorn
Ramesh Balusu
Giorgos Bamias
Karin Bammann
Syuhei Ban
Obul Reddy Bandapalli
Christianne Bandeira de Melo
Thomas Bandholm
Yasuko Bando
Naren L. Banik
Sam C. Banks
Geetha P. Bansal
Pedro V. Baptista
Hamid Reza Baradaran
Danny Barash
Olivier Barbier
Tiago M. Barbosa
Andrea Barbuti
Joseph J. Barchi
Kim A. Bard
Barbara Bardoni
William Barendse
Noël C. Barengo
Judit Bar-Ilan
Carolina Barillas-Mury
Jacob Barkley
Ruanne V. Barnabas
Steven Barnes
Jean-Claude Baron
Marco Giorgio Baroni
Muhammad Barozai
Alain Barrat
Pasqual Barretti
Paul A. Bartell
Alessandro Bartolomucci
Jason Jeremy Sinclair Barton
Richard H. Barton
Ludek Bartos
Birke Bartosch
Enrico Baruffini
Baruch Barzel
Mikael Bask
Tobias Isaac Baskin
Diane C. Bassham
Brock Bastian
Joyoti Basu
Warren Batchelor
Tone Frost Bathen
Surinder K. Batra
Da-Tian Bau
Chris T. Bauch
Olivier Baud
Michael Baudis
Michel Baudry
Joseph Alan Bauer
Wolfgang Rudolf Bauer
Mathias Baumert
Heiner Baur
Ivan Baxter
Antony Bayer

Jagadeesh Bayry
Maxim Bazhenov
Alessandra N. Bazzano
Nicole Beard
Carrie E. Bearden
Elaine L. Bearer
Brian Lee Beatty
Eduard J. Beck
Karsten Becker
Travis Beddoe
Jeffrey M. Beekman
Jeff A. Beeler
Martin Beer
Simon Beggs
Christopher Beh
Maik Behrens
Thomas Behrens
Omid Beiki
Kimon Bekelis
Andrea Belgrano
Abbes Belkhiri
Scarlett L. Bellamy
Saverio Bellusci
Christophe Beloin
Robert Belshaw
Antonio Paolo Beltrami
Suliann Ben Hamed
Charaf Benarafa
Sompop Bencharit
Mohammed Bendahmane
Claudia F. Benjamim
Lbachir Benmohamed
Joshua B. Benoit
Panayiotis V. Benos
Sliman J. Bensmaia
Guy A. M. Berbers
Rebecca Berdeaux
Iris Berent
Ivan A. Berg
Lars Berglund
Sven Bergström
Giovanna Bermano
Olivia Bermingham-McDonogh
Daniel J. Bernard
Giacomo Bernardi
Jorge Bernardino de la Serna
Boris C. Bernhardt
Harold S. Bernstein
Jean-Guy Berrin
Louis-Felix Bersier
Gina Bertocci
Antonio Bertoletti
Francesco Bertolini
Cristiano Bertolucci
Luc Bertrand
Stéphanie Bertrand
Robert C. Berwick
Josette Bettany-Saltikov
Annamaria Bevivino
Surajit Bhattacharjya
Sanjoy Bhattacharya
Samir Bhattacharya
Sukesh R. Bhaumik
Anirban Bhunia
Sujit Kumar Bhutia
Zulfiqar A. Bhutta
Giuseppe Biagini
Luigi Bianchi
Carlo Nike Bianchi
Cesario Bianchi
Nuno Bicho
Peter F. Biehl
Patrick Jon Biggs
Gary S. Bilotta
Verner Peter Bingman
Alexander Binshtok
Nanette H. Bishopric
Sylvie Bisser
Indranil Biswas
Kaushik Biswas
Animesh Biswas
Ashis Biswas
Peyman Björklund
Niklas K. Björkström
Petter Bjornstad
François Blachier

Peter C. Black
Jason Blackard
Ira J. Blader
Jeffrey L. Blanchard
Paul Blanchon
Robert Daniel Blank
Benjamin M. Blau
Miguel A. Blazquez
Wolfgang Blenau
Michelle L. Block
Bertrand Blondeau
Gareth Bloomfield
Robert Blum
David Blum
Miroslav Blumenberg
Pierre Bobé
Tomasz Bochenek
Claudi L. H. Bockting
Jerzy Bodurka
Simon Body
Philippe Boeuf
Harm Bogaard
Titus J. Boggon
Kath Bogie
Matthew Bogyo
Gil Bohrer
Alexandre Boissonnas
Bazartseren Boldgiv
Chiara Bolego
Johan J. Bolhuis
Johan Bollen
Johannes Boltze
Subbarao Bondada
Vladimir E. Bondarenko
Luca Bondioli
Ben Bond-Lamberty
Raffaella Bonecchi
Josh Bongard
Halvard Bönig
Ferruccio Bonino
Gianluca Bontempi
David Neil Bonter
Adrianus Cm Boon
David L. Boone
David R. Booth
Thomas Boraud
Iman Borazjani
David R. Borchelt
Renee M. Borges
Claudio Borghi
Katherine A. Borkovich
Cesar V. Borlongan
Lutz Bornmann
Consuelo Borras
Francesca Borrelli
Steffen Borrmann
Ray Borrow
Santanu Bose
Luciano Bosso
C. Andrew Boswell
Michel Botbol
Donald P. Bottaro
Yvonne Böttcher
Abderrezak Bouchama
Marie-Josée Boucher
Pierre Boudinot
Dmitri Boudko
Thierry Boulinier
Michael E. Boulton
Gerrit J. Bouma
Jérémie Bourdon
Kostas Bourtzis
Vassiliki A. Boussiotis
Daniel Bouvard
Mary Bowen
Prosper N. Boyaka
Mark S. Boyce
Monika Bradl
Robert Bradley
Sean Brady
Christian Braendle
Érika Martins Braga
Paula Braitstein
Scott Brakenridge
Pablo Brañas-Garza
Igor Branchi

Thomas Brand
David Douglass Brand
Johanna M. Brandner
Darrell Brann
Elvira Brattico
Shawn B. Bratton
Lidia Adriana Braunstein
Kelly A. Brayton
Björn Brembs
Caroline H. Brennan
Jerome W. Breslin
Casper J. Breuker
Diego Breviario
Matthias Briel
Vera Bril
Hassan Brim
Catherine A. Brissette
Robert Britton
Guy N. Brock
Gudrun A. Brockmann
Jeffrey L. Brodsky
James P. Brody
Basil Brooke
Shira Lynn Broschat
Jürgen Brosius
Susan Jane Broughton
Keith William Brown
Kirk Warren Brown
Kevin Scott Brown
Jon Brown
Stephanie Brown
Glenn F. Browning
Stephanie T. Broyles
Amanda Bruce
Sonia Brucki
Holger Brüggemann
Helge Bruns
Lorenzo Brusetti
Klaus Brusgaard
Vladimir Brusic
Mary Bryk
Fabio Bucchieri
Shilpa J. Buch
Maciej Buchowski
Jeffrey Buckel
Gavin Buckingham
Ashley Maurice Buckle
Hikmet Budak
Laszlo Buday
Nediljko Budisa
Irina Budunova
Valquiria Bueno
Raffaele Bugiardini
Bang V. Bui
Catalin Buiu
Joseph K. Bump
Jacob Guy Bundy
Kevin D. Bunting
Emanuele Buratti
Irina Burd
Emmanuel A. Burdmann
Harold A. Burgess
Robert D. Burk
Thomas H. J. Burne
Jorge S. Burns
Kevin Burrage
Christina A. Bursill
Rainer Bussmann
Benedetta Bussolati
Pierre Busson
Paco Bustamante
Patrick Butaye
Peter Butko
Michael B. Butterworth
Raffaella Buzzetti
Siddappa Byrareddy
Kimberly R. Byrnes
Baltica Cabieses
Qiliang Cai
Tao Cai
Huaibin Cai
Xiaodong Cai
Yang Cai
Christian Cajochen
Laura Calabresi
Francesc Calafell

Jose A. L. Calbet
Adriana Calderaro
Christine A. Caldwell
Joseph A. Califano
George Calin
Raffaele A. Calogero
John Calvert
Diego Calvisi
Niels Olsen Saraiva Câmara
Elissa Z. Cameron
D. William Cameron
Douglas A. Campbell
Moray Campbell
Kevin Camphausen
Giovanni Camussi
Hector Candela
Cong Cao
Yongchang Cao
Bing-Yang Cao
Heping Cao
Cristian Capelli
Steve Caplan
Andrea Caporali
Francesco Cappello
Massimo Caputi
David Caramelli
Christopher Carcaillet
Pere-Joan Cardona
Marly Augusto Cardoso
Paolo Carloni
Clotilde K. S. Carlow
Morgan E. Carlson
Yohay Carmel
Ryan M. Carnahan
Tom J. Carney
Luis Carretero
David Carrier
Dee A. Carter
David Raul Francisco Carter
Calogero Caruso
Luzia Helena Carvalho
Leonardo Jose de Moura Carvalho
Dulce Elena Casarini
Eric Cascales
Giovanni Casella
Philippe Castagnone-Sereno
Jose G. Castaño
Riccardo Castiglia
Filippo Castiglione
Javier S. Castresana
Alberico Catapano
Janet Catov
Luigi Cattaneo
Byron Caughey
Giacomo Cavalli
Andrea Cavalli
Luigi Maria Cavallo
Elena Cavarretta
Joan A. Caylà
Marek Cebecauer
Just Cebrian
Francesca Ceccherini-Silberstein
Shan Cen
Valentin Ceña
Cristina Cereda
Nicolas Cermakian
Jose A. Chabalgoity
Maurice J. Chacron
Brian P. Chadwick
Karl X. Chai
Zhenhua Chai
Lisa Chakrabarti
Debasis Chakrabarty
Shukti Chakravarti
Nicolas Chaline
Etienne Challet
Jeffrey Chalmers
Martin Chalumeau
Mathias Chamaillard
Alanna M. Chamberlain
Roger Chammas
Chi-Chao Chan
Kwok Hung Chan
Kelvin Yuen Kwong Chan

David Wai Chan
Christina Chan
Renee W. Y. Chan
Michael K. Chan
Zhulong Chan
Chi Bun Chan
Dhyan Chandra
Yung-Fu Chang
Jeffrey S. Chang
Chin-Kuo Chang
Yu-Jia Chang
Ing-Feng Chang
Tailoi Chan-Ling
Linda Chao
Maura (Gee) Geraldine Chapman
Georges Chapouthier
Alain Charbit
Thierry Chardot
Benjamin D. Charlton
Charlotte Charpentier
Stéphane Charpier
Isabelle Charrier
Lucienne Chatenoud
Delphi Chatterjee
Dipankar Chatterji
Vishnu Chaturvedi
Christos Chatziantoniou
Gyaneshwer Chaubey
Gautam Chaudhuri
Ashok Chauhan
Franck Chauvat
Triantafyllos Chavakis
Esteban Chaves-Olarte
Peh Yean Cheah
William Checkley
Alain Chédotal
Farid F. Chehab
Leonardo Chelazzi
Srikumar Chellappan
Isabelle Chemin
Zhiwei Chen
Cathy W. S. Chen
Yan Chen
Robert Chen
Xiaoli Chen
Peter Chen
Xinbin Chen
Chin-Tu Chen
Chi-Ling Chen
Lin Chen
Jing Chen
Aimin Chen
Jeremy J. W. Chen
Han Y. H. Chen
Maohua Chen
Tzong-Yueh Chen
Jonathan Hon-Kwan Chen
Chien-Sheng Chen
Suzie Chen
Kewei Chen
Xiongwen Chen
Yanguang Chen
Guo-Qiang Chen
Chih-Jung Chen
Zhong-Hua Chen
Chunxian Chen
Yuan-Jia Chen
Han-Chiao Isaac Chen
Chaolun Allen Chen
Jin-Ran Chen
Qun Chen
Mike Chen
Zhongxue Chen
Yang-Ching Chen
Shilin Chen
Li-Mei Chen
Zhukuan Cheng
Xiaodong Cheng
Xianwu Cheng
Zhihui Cheng
Juei-Tang Cheng
Venugopalan Cheriyath
Stacey Cherny
Jonathan Chevrier
Jay Chhablani

Dante R. Chialvo
Keng-Hwee Chiam
Tzen-Yuh Chiang
André Chiaradia
Lorenzo Chiariotti
Ornit Chiba-Falek
Roberto Chiesa
Jeremiah Chikovore
Joseph Chilcot
Wei-Chun Chin
John A. Chiorini
Giuseppe Chirico
Nakul Chitnis
Charles Y. Chiu
Chung-Jung Chiu
Kin-Sang Cho
Doo-Sup Choi
Jonghoon Choi
Kim-Kwang Raymond Choo
Imti Choonara
Arvind Chopra
Sanjay Haresh Chotirmall
Anuradha Chowdhary
Gerardo Chowell
Julie A. Chowen
Hanna Christiansen
Brian Christie
Magdalena Chrzanowska-Wodnicka
Pei-Yi Chu
Hong Wei Chu
Jen-Hsiang Chuang
Eric Y. Chuang
Michael Hoonbae Chung
Chun Kee Chung
Yeonseok Chung
Sookja Chung
Andrew Churg
Chih-Pin Chuu
Massimo Ciccozzi
Andrea Cignarella
Andrea Cimarelli
Daniela Cimini
Roberta Cimmaruta
Alessio Cimmino
Pietro Cipresso
Patrick C. Cirino
Vitaly Citovsky
Leon Claessens
Bruno Clair
Jesse L. Clark
Timothy Darren Clark
Simon J. Clark
David J. Clark
Stephen L. Clarke
Paul Robert Cleary
James D. Clelland
Axel Cloeckaert
Paul A. Cobine
Amanda M. Cockshutt
Luca Cocolin
Claudia Torres Codeço
Loren D. Coen
Tom Coenye
Lark L. Coffey
Frederick M. Cohan
Irun R. Cohen
Noam A. Cohen
Malka Cohen-Armon
Marco Colasanti
Donn J. Colby
Alexander M. Cole
William B. Coleman
Ross Coleman
Craig Eliot Coleman
Jonathan A. Coles
Donald James Colgan
Giorgio Colombo
Gualtiero I. Colombo
Fabio Cominelli
Lisa Conboy
J. Alberto Conejero
Marco Congedo
John Conly
Philippe Connes
Che John Connon

James R. Connor
Marcia Edilaine Lopes Consolaro
Sofia Consuegra
Mark R. Cookson
Austin John Cooney
Brenton G. Cooper
Domenico Coppola
Vincenzo Coppola
Richard Cordaux
Nils Cordes
Estelle Cormet-Boyaka
Stephania A. Cormier
Stephen J. Cornell
Nicolas Corradi
Ignacio Correa-Velez
Aldo Corriero
Ilaria Corsi
Manuel João Costa
Max Costa
Luis Costa
Sergio Costa Oliveira
Claudio M. Costa-Neto
Marcello Costantini
Alix Therese Coste
Eithne Costello
Michael Costigan
Daniela Cota
Sue Cotterill
Roger A. Coulombe
Serena Counsell
Franck Courchamp
Michael A. Cousin
Francisco M. Couto
Mihai Covasa
Lauren Ashley Cowart
Benjamin J. Cowling
Dermot Cox
James Coyne
Mario Cozzolino
John A. Craft
Alister G. Craig
Corentin Cras-Méneur
Paolo Cravedi
Dana C. Crawford
James J. Cray Jr.
François Criscuolo
Stefania Crispi
Fatima Crispi
Jose C. Crispin
Marco Cristani
Anna Kristina Croft
Simon J. Cropper
Damian Christopher Crowther
Mathew S. Crowther
Wim E. Crusio
Rogelio Cruz-Martinez
Peter Csermely
Laszlo Csernoch
Attila Csikász-Nagy
Suresh Cuddapah
Zongbin Cui
Ranji Cui
Zoran Culig
Daniel Cullen
Richard Culleton
Thiago Mattar Cunha
Edecio Cunha-Neto
Giuseppe Curcio
Sean P. Curran
Kevin P. M. Currie
Bruce S. Cushing
Salvatore Cuzzocrea
Gennady Cymbalyuk
Lucette A. Cysique
Paula A. da Costa Martins
Marcel Daadi
Krystyna Dąbrowska
Fulvio d'Acquisto
Francesca D'Addio
Soheil S. Dadras
Daniele Daffonchio
Etienne Dague
Shaojun Dai
Felipe Dal Pizzol
Yamini Dalal

Andrew R. Dalby
Doralyn S. Dalisay
Emanuele G. Dalla Torre
Hans G. Dam
Margot Damaser
Fábio M. Damatta
Erik H. J. Danen
Christopher M. Danforth
Yong-Hui Dang
Ganesh Dangal
Bryan C. Daniels
Robert Dante
Ming Dao
Li Daqing
Andrea Dardis
Jean-Luc E. P. H. Darlix
Gokul M. Das
Surajit Das
Anindita Das
Gobardhan Das
Suryasarathi Dasgupta
Chandravanu Dash
Prasun K. Datta
Sibnarayan Datta
Alessandro Datti
Jean Daunizeau
Sabato D'Auria
Alessandro D'Ausilio
Andrew Davies
C. Todd Davis
Keith R. Davis
Sarah C. Davis
Roberta Davoli
Thomas L. Dawson
Abhijit De
W. F. de Boer
Alexandre G. de Brevern
Robertus A. M. de Bruin
Fernando de Castro
Maite de Castro
Ivan de Curtis
Jan de Fockert
Vittorio de Franciscis
Anne S. De Groot
Wouter de Herder
Vinicio A. de Jesus Perez
Juan C. de la Torre
Floris P. de Lange
Herminia de Lencastre
Marc H. E. de Lussanet
Paulo De Marco Júnior
Ramon Andrade De Mello
Gonzalo G. de Polavieja
Valli De Re
Salvatore De Rosa
Bert De Smedt
Ive De Smet
Giuseppe Vittorio De Socio
Russell J. de Souza
Deborah Dean
Wendy Dean
Margaret M. DeAngelis
Peter K. Dearden
Sumitra Deb
Waldemar Debinski
Chitrita DebRoy
Stéphane Declerck
Yuriy Dedkov
Gagan Deep
Vadim E. Degtyar
Faramarz Dehghani
Matthias Dehmer
Sharon Dekel
Olaf M. Dekkers
Juan Carlos del Alamo
Filippo Del Bene
M. Consuelo del Cañizo
Maurizio Del Poeta
Mária A. Deli
Odir Antônio Dellagostin
Persio Dello Sbarba
Dominique Delmas
Giovanni Delogu
Neal A. DeLuca
Vincenzo DeLuca
Sylvain Delzon

Yong Deng
Hao Deng
Dajun Deng
Zhaohong Deng
Z. Daniel Deng
Vida A. Dennis
Alok Deoraj
Tobias Derfuss
Kebede Deribe
Bart Dermaut
José Guilherme Behrensdorf Derraik
Gemma Elizabeth Derrick
Sébastien Descamps
Abhishek Deshpande
Nicolas Desneux
Maurizio D'Esposito
Jean-Luc Desseyn
Mickaël Desvaux
Robert W. Dettman
Joseph Devaney
Timothy P. Devarenne
Brecht Devleesschauwer
Joanne M. Devlin
Andrew T. DeWan
Saikat Dewanjee
Charlene S. Dezzutti
Sangeeta Dhaubhadel
Marc Dhenain
Navneet K. Dhillon
Yuanpu Peter Di
Ferdinando Di Cunto
Riccardo Di Giminiani
Simone Di Giovanni
Arianna Di Napoli
Javier Marcelo Di Noia
Giuseppe di Pellegrino
Francesco Di Russo
Antonino Di Stefano
Jiajie Diao
João Miguel Dias
Emmanuel Dias-Neto
Bruno Lourenco Diaz
María Carmen Díaz Roldán
Guillermo Diaz-Pulido
Frederic Dick
Joseph Clifton Dickens
Alex Dickson
Francesco Dieli
David Joseph Diemert
Binh An Diep
Kottarappat N. Dileepan
Patrizio Dimitri
Dimiter S. Dimitrov
George Dimopoulos
Qiang Ding
Tzvetanka D. Dinkova
Rod K. Dishman
Joseph DiStefano III
Thomas Dittmar
Kimon Divaris
Vishal Diwan
Oleg Y. Dmitriev
Renwick Dobson
Aristides Docoslis
Christopher B. Doering
Junsang Doh
Hideyuki Doi
Riccardo Dolcetti
Juan M. Dominguez
Bruce R. Donald
Julie G. Donaldson
Qunfeng Dong
Yufeng Dong
Maureen J. Donlin
Christopher M. Doran
Pablo Dorta-González
Mirella Dottori
Dejing Dou
Daniel Doucet
Michael E. Douglas
Constantine Dovrolis
Jennifer Beam Dowd
David W. Dowdy
Joël R. Drevet
Steven J. Drews

Petros Drineas
Paul C. Driscoll
Stuart E. Dryer
Chenyu Du
Shao Jun Du
Lanying Du
Wenjie Duan
Marie-Pierre Dubé
Ludwig Dubois
James A. Duce
John Duda
Anette Duensing
N. S. Duesbery
Jozef Dulak
J. Stephen Dumler
Anna Dunaevsky
Gary L. Dunbar
Steven R. Duncan
Melinda Duncan
Sébastien Duperron
Daniel E. Duplisea
Denis Dupuy
William Durante
Caroline Durif
Daniel Durstewitz
Jean-Claude Dussaule
Amit Dutt
Adrian G. Dyer
Simon Dymond
Petras Dzeja
Valsamma Eapen
Conrad P. Earnest
Ivano Eberini
Matthias Eberl
Kristie L. Ebi
Klaus Ebmeier
Esmaeil Ebrahimie
Miriam Echevarría
Richard L. Eckert
Tobias Eckle
David T. Eddington
Mariola J. Edelmann
Andreas B. Eder
Owain Rhys Edwards
Philip Alexander Efron
Christophe Egles
Oliver Eickelberg
Henrik Einwächter
Leonard Eisenberg
Peter Eklöv
Charbel El Bcheraoui
Sam Eldabe
James Bradley Elder
Wael El-Deredy
Mounya Elhilali
M. Carolina Elias
Kathrin Eller
Nathan A. Ellis
Osman El-Maarri
Stefan Elmer
Mohammed E. Elsalanty
Hany A. El-Shemy
Costanza Emanueli
Frank Emmert-Streib
Nicole Endlich
Jacob Engelmann
Adam J. Engler
Christoph Englert
Omolola Eniola-Adefeso
Danilo Ercolini
Gabor Erdoes
Isil Ergin
Kimmo Eriksson
James M. Ervasti
Alejandro Escobar-Gutiérrez
Hector Escriva
Mariapaz Espinosa
Alejandro A. Espinoza Orías
L. Michel Espinoza-Fonseca
Susanna Esposito
M. Faadiel Essop
Francisco J. Esteban
William J. Etges
Nima Etminan
Eliseo A. Eugenin
Alistair Robert Evans

Conor L. Evans
Nir Eynon
Hiroshi Ezura
Antonio Facchiano
Cecile Fairhead
Syed Faisal
Catherine Faivre-Sarrailh
Patrizia Falabella
Marco Falasca
Barbara Fam
Xiaobing Fan
Guo-Chang Fan
Yong Fan
Victoria Y. Fan
Shenying Fang
David D. Fang
Deyu Fang
Eric Brian Faragher
Alfonso Fasano
Michael Ernst-Heinrich Fassbender
S.Hossein Fatemi
Dimitris Fatouros
Giampiero Favato
Guido Favia
Stefano Federici
Maurizio Federico
Carol Feghali-Bostwick
Michael G. Fehlings
Heinz Fehrenbach
Frank Alexander Feltus
Ying-Mei Feng
Youjun Feng
Wenke Feng
Brock Fenton
Robert A. Fenton
Miguel A. Fernandez
José A. Fernández Robledo
Narcis Fernandez-Fuentes
Pedro Fernandez-Funez
Delmiro Fernandez-Reyes
Martin Fernandez-Zapico
Rashida A. Ferrand
Nicola Ferri
Raffaele Ferri
Sebastian C. A. Ferse
Marco Festa-Bianchet
Matthew Fidelibus
Edda Fiebiger
Ruth Filik
Scott G. Filler
Stéphanie Filleur
Helen Fillmore
Michael L. Fine
David I. Finkelstein
Anthony Fiorillo
Paolo Fiorina
Gabriele Fischer
Uwe Fischer
Matthew C. Fisher
Gordon Fisher
Gilberto Fisone
Patricia Fitzgerald-Bocarsly
David Flaspohler
Johannes Fleckenstein
Sheila M. Fleming
Andres R. Floto
Stephen S. Fong
Peter P. Fong
Leng Huat Foo
Christiane Forestier
Gianluigi Forloni
Albert J. Fornace Jr
Naomi Forrester
Thomas Forsthuber
Patrice E. Fort
Anny Fortin
Dimitrios Fotiadis
Nicholas Simon Foulkes
Hayley J. Fowler
Matthew P. Fox
Michael A. Fox
Leonardo Fraceto
Diego Fraidenraich
Heather Francis
Rodrigo Franco

Renato Franco
Nikolaos Frangogiannis
Ingmar H. A. Franken
Alexander W. E. Franz
Andrea Franzetti
Martin Gerbert Frasch
Abigail Fraser
Pina Fratamico
Franca Fraternali
Giacomo Frati
A. Lenin Fred
David N. Fredricks
Jonathan H. Freedman
James Freeman
Alexander N. Freiberg
Michael Freitag
Kathleen Freson
Oliver Frey
Brigitte M. Frey
Esteban Andres Fridman
Iddo Friedberg
Tim Friede
Matt Friedman
Doron Friedman
Peter A. Friedman
Alex Friedrich
Friedrich Frischknecht
Laura Frishman
Jörg Hermann Fritz
Holger Fröhlich
Fabrizio Frontalini
Mark A. Frye
Binying Fu
Jian Fu
Kai Fu
Sebastien Fuchs
Rita Fuchs
Mariana M. P. B. Fuentes
Sebastian D. Fugmann
Jong-Ling Fuh
Barbara Fuhrman
Hodaka Fujii
Tohru Fukai
Yoshihiro Fukumoto
Stephany Fulda
Dorian Q. Fuller
Jennifer C. Fung
Roberto Furlan
Clemens Fürnsinn
Ivan J. Fuss
Sandra B. Gabelli
Attila Gacser
Sudhindra R. Gadagkar
Alain-Pierre Gadeau
Amit Gaggar
Joel Joseph Gagnier
Paul J. Galardy
Massimiliano Galdiero
Philippe A. Gallay
Alvaro Galli
Lazaros K. Gallos
Imed Eddine Gallouzi
Andrew C. Gallup
Ferenc Gallyas Jr.
Karen L. Gamble
Yang Gan
Siew Hua Gan
T. R. Ganapathi
A. Ganesan
Giuseppe Gangarossa
Nupur Gangopadhyay
Roman R. Ganta
Apar Kishor Ganti
Soren Gantt
Shou-Jiang Gao
Qian Gao
Jian-Xin Gao
Lei Gao
Feng Gao
Xin Gao
Yulin Gao
Chang-Qing Gao
Emmanuel Gaquerel
Silvio Garattini
Jose Manuel Garcia Aznar
Pablo Garcia de Frutos

Carlos Garcia de Leaniz
Alberto García-Basteiro
Aurora García-Gallego
José-María García-García
J. Gerardo García-Lerma
Mikel Garcia-Marcos
Rafael Garcia-Mata
Jordi Garcia-Ojalvo
Steven Allen Gard
David S. Gardner
Pradeep K. Garg
Floriana Gargiulo
Krishna Garikipati
Pere Garriga
Andrei L. Gartel
Antonella Gasbarri
Stephane Gasman
Mauro Gasparini
Maria Gasset
Michelle Louise Gatton
Nick Gay
Stefan Gebhardt
Fabrizio Gelain
Mathias Gelderblom
Juri G. Gelovani
Daniele Generali
Damian Christopher Genetos
David Gent
Valérie Geoffroy
Irene Georgakoudi
Nikolaos Georgantzis
Joseph George
Anthony M. George
Robert J. Geraghty
André Paul Gerber
Sharon Gerecht
Roman G. Gerlach
Stephane Germain
Matthew Germino
Hernâni Gerós
Edward Gershburg
Murad Ghanim
Saeid Ghavami
Pietro Ghezzi
Jagadananda Ghosh
Samiran Ghosh
Debashis Ghosh
Guillermo H. Giambartolomei
Maria Grazia Giansanti
Emiliano Giardina
Yann Gibert
Spencer B. Gibson
Mark Gijzen
Sam Gilbert
Giorgio F. Gilestro
Andrew C. Gill
Matthew S. Gill
Daniel Gillet
David P. Gillikin
Thomas H. Gillingwater
Stephen E. Gilman
Edward Giniger
Stephen D. Ginsberg
Francesco Giorgino
Olivier Gires
Alessandro Giuffrè
Michele Giugliano
Georgios V. Gkoutos
Wolfgang Glanzel
Stefan Glasauer
Norbert Gleicher
John I. Glendinning
John W. Glod
Joseph Charles Glorioso
Christian Gluud
Lise Lotte Gluud
M. Maria Glymour
Ajay Goel
Stefano Goffredo
Jeffrey A. Gold
Anna Carla Goldberg
Jeremy D. Goldhaber-Fiebert
Gustavo Henrique Goldman
Ellen R. Goldman
Bob Goldstein
Edward Goldstein

Delia Goletti
Déla Golshayan
Aldrin V. Gomes
Hector Gomez
Gabriela B. Gomez
Sergio Gómez
Diego F. Gomez-Casati
Jesus Gomez-Gardenes
Lourdes Gómez-Gómez
Dusan Gomory
Raquel Goncalves
Zhiyuan Gong
Qiyong Gong
Pedro Gonzalez
Germán E. González
Pedro Gonzalez-Alegre
Jose Luis Gonzalez-Andujar
Concepción Gonzalez-Bello
Antonio Gonzalez-Bulnes
José M. González-Méijome
John Goodrich
Subash C. B. Gopinath
Olga Y. Gorlova
Elena Gorokhova
Sven Bernhard Gould
Marie Jose Goumans
Sebastien Gourbiere
Alessandro Gozzi
Alena Grabowski
Luis Graca
Jordi Gracia-Sancho
Ilana Graetz
Susan Marie Graham
Paul Graham
Jessica Adrienne Grahn
Sander Granneman
Giovanni Grasso
Brian Gratwicke
Ronald H. Gray
Magdalena Grce
Jason Grebely
Andy J. Green
Stefan J. Green
Colin Green
John Green
Elisa Greggio
Luisa Gregori
Aric Gregson
Pierre Gressens
Michael E. Grigg
Ramon Grima
Anatoly V. Grishin
Sergei Grivennikov
Laurent Groc
Vince Grolmusz
Wulfila Gronenberg
Stan Gronthos
Rita Grosch
Michela Grosso
Tudor Groza
Tilman Grune
Salvatore Gruttadauria
Quanquan Gu
Oreste Gualillo
Camillo Gualtieri
Le Luo Guan
Adam J. Guastella
Jacopo Guccione
Teja Guda
Raul Narciso Carvalho Guedes
Agustín Guerrero-Hernandez
Fernando Guerrero-Romero
Nuri Gueven
Jiang Gui
Gaël Guilhem
Gilles J. Guillemin
Hervé Guillou
Maria Gulinello
Donald Gullberg
Sinan Guloksuz
Ming Guo
Xuejiang Guo
Nancy Lan Guo
Haitao Guo
Wenge Guo
Yiru Guo

Vineet Gupta
Dinesh Gupta
Sudhiranjan Gupta
Ravindra K. Gupta
Sudeep Gupta
Vijai Gupta
Robert Guralnick
Roi Gurka
Attila Gursoy
Eva Gutheil
José María Gutiérrez
Roee Gutman
Julie Gutman
Sung Ho Ha
Nikolas K. Haass
Georg Häcker
Jeffrey M. Haddad
Nouchine Hadjikhani
Leontios Hadjileontiadis
Christoph E. Hagemeyer
Christian Andrew Hagen
Sinuhe Hahn
Cecil D. Hahn
Neena B. Haider
Mehrdad Hajibabaei
Ramin M. Hakami
Mohamed Ali Hakimi
Siân E. Halcrow
Rebecca A. Hall
Kay Hamacher
Björn Hamberger
Michael Hamblin
Frederick G. Hamel
Shalaka Hampras
Michelle Hampson
Arum Han
Jae Yong Han
Gang Han
Xiaonan Han
Guoqi Han
Kap-Hoon Han
Yuepeng Han
Yiping Han
Weiqing Han
Zhaozhong Han
Ronald Hancock
Lynn E. Hancock
John Travers Hancock
David J. Handelsman
Marc Hanewinkel
Bo Hang
Immo A. Hansen
Steen Henning Hansen
Peter J. Hansen
Jürgen Harder
Tilmann Harder
Karen Hardy
Daniel Barry Hardy
Joshua M. Hare
Jaroslaw Harezlak
Edward William Harhaj
Leila Harhaus
Pirkko L. Härkönen
Ilan Harpaz-Rotem
Diane Medved Harper
David Harrich
Steven Harris
Keith M. Harris
Fiona Harris
Jeffrey K. Harrison
Kevin Harrod
Anne C. Hart
John P. Hart
Dominik Hartl
Lisa Hartling
M. Elizabeth Hartnett
James K. Hartsfield
Kim J. Hasenkrug
Kenji Hashimoto
Seyed Ehtesham Hasnain
Imtaiyaz Hassan
Martine Hausberger
Frank Havemann
Richard G. Haverkamp
Shannon M. Hawkins
Dror Hawlena

Satoru Hayasaka
Naoyuki Hayashi
Johannes Haybaeck
Finbarr Hayes
Shawn Hayley
Graeme Hays
Elliott Lee Hazen
Alain Haziot
Meian He
Weijing He
Bin He
Na He
Yong He
Guangyuan He
Huiguang He
Zhili He
Xiaoming He
Bin He
Chunyan He
Ya-Wen He
Zhengbing He
Yuxian He
Joshua L. Heazlewood
Christopher Heeschen
Nagendra R. Hegde
Berthold Heinze
Clemens Heiser
Andreas Hejnol
James Fielding Hejtmancik
Samuli Helle
Manuela Helmer-Citterich
Fred J. Helmstetter
Charlotte K. Hemelrijk
Jan M. Hemmi
Susanne Hempel
Michael Hendricks
Jeroen Hendrikse
Petr Heneberg
Michael Hensel
Yann Herault
Christian Herder
Karl Herholz
Carlos Hermenegildo
Marcelo Hermes-Lima
Adrian V. Hernandez
Pilar Hernandez
Alejandro Raul Hernandez
Montoya
Edgar Hernandez-Andrade
Tina Hernandez-Boussard
Enrique Hernandez-Lemus
Eder Guillermo Herrera
Alfredo Herrera-Estrella
Jose Ignacio Herrero
Michael H. Herzog
Wolfgang R. Hess
Michal Hetman
Judi Hewitt
Dominique Heymann
Jan Geert Hiddink
Dennis M. Higgs
Kristiina Hildén
Philip C. Hill
Edna Hillmann
Peter James Hills
Robert K. Hills
Karen Hind
Dariush Hinderberger
Kensuke Hirasawa
Emilio Hirsch
Peter F. Hitchcock
Anita B. Hjelmeland
Paulo Lee Ho
Mitchell Ho
Yuan-Soon Ho
Wenzhe Ho
Mojgan Hodaie
Stephen Hodgins
Heather Hoffmann
Andreas-Claudius Hoffmann
Thomas G. Hofmann
Andreas Hofmann
Philip J. Hogarth
Jörg D. Hoheisel
Peter Hohenstein
Petter Holme

David Holowka
Christian Holscher
Judith Homberg
Xiao-Yue Hong
Yiguo Hong
Jeum Kyu Hong
Dong Hoon Shin
Jane Hoppin
Mohammad O. Hoque
Elvira Hörandl
Arie Horowitz
Malcolm James Horsburgh
Tanya Horsley
Marc S. Horwitz
Yoko Hoshi
Yujin Hoshida
Yoshihiko Hoshino
Khaled Hossain
Ling Hou
Chenping Hou
Andreas Houben
Nicolas Houlie
Daniel Houser
Jon C. D. Houtman
Thomas R. Howdieshell
Piers Douglas Lionel Howe
Peter Howell
Daniela Flavia Hozbor
Marta Letizia Hribal
Janet Hsiao
Chuhsing Kate Hsiao
Ying-Hen Hsieh
Hsi-Lung Hsieh
Yi-Hsien Hsieh
Chih-Hao Hsieh
Patrick C. H. Hsieh
Valerie W. Hu
Xiaosong Hu
Wenhui Hu
Cheng Hu
Dewen Hu
Jianjun Hu
Yi Hu
Chun-Hsi Huang
Jee-Fu Huang
Yu Huang
Hao Huang
Yhu-Chering Huang
Jinhai Huang
Wendong Huang
Yi-Hsiang Huang
Qingyang Huang
Xuhui Huang
Yuan Huang
Victor C. Huber
Dorothee Huchon
Barry I. Hudson
Michael Shing-Yan Huen
Rodrigo Huerta-Quintanilla
François Hug
Elizabeth Hughes
Dafeng Hui
J. Joe Hull
Sandrine Humbert
Jean-François Humbert
Thomas Hund
Teh-Ia Huo
Robert Hurst
Salik Hussain
Nguyen Tien Huy
Snehalata Huzurbazar
Jiang-Shiou Hwang
Sheng-Ping Lucinda Hwang
David R. Hyde
David Hyrenbach
Marco Iacoboni
Adrianna Ianora
A. Mark Ibekwe
Andrea Icks
Ellen Idler
Marco Idzko
Koichi M. Iijima
Kazutaka Ikeda
Tetsuro Ikegami
Ryozo Imai
Axel Imhof

Carmen Infante-Duarte
Hanne Ingmer
Pär K. Ingvarsson
Satoshi Inoue
Nigel Irwin
Richard E. Isaacson
Petros Isaakidis
Yoshitaka Isaka
Mark Isalan
Carlos M. Isales
Akira Ishihama
Yoshiro Ishimaru
Fakir M Amirul Islam
Saeed Islam
Hiroyuki Itabe
Etsuro Ito
Miren Iturriza-Gómara
Yury P. Ivanenko
Zoran Ivanovic
Juraj Ivanyi
Magnus Ivarsson
Kazuya Iwamoto
Jose Mg Izarzugaza
Angelo A. Izzo
Esmaiel Jabbari
Monica M. Jablonski
Catherine L. Jackson
Jon M. Jacobs
David S. Jacobs
Ilse D. Jacobsen
Steven Jacobson
Sanjay B. Jadhao
Ganesh Chandra Jagetia
Mohamed Zain Zulfiqhar Jahufer
Mukesh Jain
Pankaj Jaiswal
Leighton R. James
Yih-Kuen Jan
Eric Jan
Nihar Ranjan Jana
Lutz Jäncke
Karin Jandeleit-Dahm
Andreas R. Janecke
Sung Key Jang
Damir Janigro
Axel Janke
Veerle Janssens
Heather B. Jaspan
Arul Jayaraman
John Lynn Jefferies
Mika Jekabsons
Wolfgang E. B. Jelkmann
Eric Jellen
Albert Jeltsch
Clinton N. Jenkins
Barbara Jennings
Anja T. R. Jensen
Jong-Seong Jeon
Jae-Wook Jeong
Ruth Jepson
Samithamby Jeyaseelan
Vivekanand Jha
Vishal Jhanji
Ravi Jhaveri
Zhanjun Jia
Shibo Jiang
Quan Jiang
Yong Jiang
Zhigang Jiang
Bing-Hua Jiang
Bin Jiang
Junfeng Jiao
Susana Jiménez-Murcia
Dong-Yan Jin
Xia Jin
Jian Jing
Dong-Gyu Jo
Sudisha Jogaiah
Ulrich Joger
Ludger Johannes
Erik C. Johnson
Christopher James Johnson
Welkin E. Johnson
Colin Johnson
Blake Johnson

Jaap A. Joles
Julia A. Jones
Henrik Jönsson
I. King Jordan
Shijo Joseph
David Jourd'heuil
Rex Eugene Jung
Juan Luis Jurat-Fuentes
Jeffrey Jutai
Lars Kaderali
D. M. Kado
Philipp J. Kahle
Chikara Kaito
Panagiotis Kalaitzis
Ruslan Kalendar
Vipin Chandra Kalia
Tanya V. Kalin
Vladimir V. Kalinichenko
Ganjam V. Kalpana
Jason M. Kamilar
Juliane Kaminski
Harm H. Kampinga
Akio Kanai
Tatsuo Kanda
Osamu Kaneko
Jean Kanellopoulos
Colette Kanellopoulos-Langevin
Rui Kang
Hojeong Kang
Sang-Moo Kang
Natarajan Kannan
Chryso Kanthou
Jyotshna Kanungo
Makoto Kanzaki
Katy C. Kao
Maria Kaparakis-Liaskos
Amit Kapoor
Zoi Kapoula
Sophia N. Karagiannis
Petros C. Karakousis
Nikos K. Karamanos
Dimitrios Karamichos
Vardan Karamyan
Goran Karapetrov
Efstathios Karathanasis
Leszek Karczmarski
Jörn Karhausen
Petr Karlovsky
Sadashiva S. Karnik
Manjula Karpurapu
Susanne Kaser
Fatah Kashanchi
Khalil Kashkush
Jan Kassubek
Rajesh Gopalrao Katare
Tadafumi Kato
Takuma Kato
Masaru Katoh
Jordy Kaufman
Gunnar F. Kaufmann
Rupert Kaul
Alexandra Kavushansky
Manfred Kayser
Kylene Kehn-Hall
Miklos S. Kellermayer
Christina A. Kellogg
John J. Kelly
Gregory M. Kelly
Jake Kerby
Irina Kerkis
Ozlem Keskin
Hans A. Kestler
Brian Key
Alexandra Key
Mohammad Ebrahim Khamseh
Wasif N. Khan
M. Firoze Khan
Hafiz T. A. Khan
Gulfaraz Khan
Muhammad Khurram Khan
Asad U. Khan
Raya Khanin
Hemant Khanna
Rohit C. Khanna
Mohamed T. Khayyal
Jong Seong Khim

Reza Khodarahmi
Yury E. Khudyakov
Zoha Kibar
Stefan Kiebel
Stefan Kiechl
Steffen Kiel
Alexandre Hiroaki Kihara
Chang H. Kim
Hoguen Kim
Yoon Ki Kim
Samuel Kim
Hongkyun Kim
Jeong-Ho Kim
Do Young Kim
Jayoung Kim
Jung-Woong Kim
Sung Wan Kim
Jonghan Kim
Beom Seok Kim
Jung-Eun Kim
Seung Up Kim
Kyoung Heon Kim
Tjeerd Kimman
Akinori Kimura
Anthony E. Kincaid
Sonja Kinner
Rudolf Kirchmair
Martyn Kirk
Marian Kjellevold
Robyn S. Klein
Jens Kleinjung
Christoph Kleinschnitz
Dimitris Kletsas
Athol Victor Klieve
A. Peter Klimley
Jan Kluytmans
Michael Klymkowsky
Michael Knapp
Olaf Kniemeyer
Matty Knight
Laura J. Knoll
Jason Glenn Knott
Dennis C. Ko
Bostjan Kobe
Firas H. Kobeissy
Karl-Wilhelm Koch
Theresa M. Koehler
Deanna M. Koepp
Tsuyoshi Koide
Kin-Hang Kok
Yoshihiro Kokubo
Frank T. Kolligs
Sergios-Orestis Kolokotronis
Motohiro Komaki
Natalia L. Komarova
Yulia Komarova
Masaaki Komatsu
Melanie Königshoff
Marina Konopleva
Dimitris L. Kontoyiannis
John Matthew Koomen
Murray Korc
Michael S. D. Kormann
Sergey Korolev
Maya Koronyo-Hamaoui
Konstantinos Kostikas
Erika Kothe
Sonja Kotz
Yu Ru Kou
Katerina Kourentzi
Sotirios Koutsopoulos
Michael Koval
Susan Kovats
Roberto Andre Kraenkel
Ralf Krahe
Florian Krammer
Laurent Kremer
Eric J. Kremer
Laurent Kreplak
Mariska E. Kret
Jens Kreth
Andreas Krieg
Viswanathan V. Krishnan
Evelyn Kroesbergen
Andrew J. Kroll
Florian Kronenberg

Rebecca A. Krukowski
Wilfried A. Kues
Thomas A. Kufer
Jens H. Kuhn
Oscar P. Kuipers
Helena Kuivaniemi
Thomas Kukar
Rakesh Kukreja
Pawan L. Kulwal
Sanjai Kumar
Ashok Kumar
Anil Kumar
Lalit Kumar
Saravana Kumar
Shashi Kumar
Nirbhay Kumar
Ashok Kumar
Navnith K. P. Kumaran
Chandan Kumar-Sinha
Tsutomu Kume
Muthusamy Kunnimalaiyaan
Matjaž Kuntner
Gotthard Kunze
Ho-Chang Kuo
Chih-Horng Kuo
Kornelius Kupczik
Gary Kupfer
Eiko Eurya Kuramae
Petri Kursula
Jürgen Kurths
Miyako Kusano
Masataka Kuwana
Michael Kyba
Natasha Kyprianou
Massimo Labra
Juan-Pablo Labrador
Nathalie Labrecque
Jean-Marc Lacape
Nicola Lacetera
Ezio Laconi
Shannon L. Ladeau
Robert M. Lafrenie
Victor Alberto Laguna-Torres
Tim Lahm
Martina Lahmann
Garet P. Lahvis
Jui-Yang Lai
Hsin-Chih Lai
Erh-Min Lai
Liangxue Lai
Katariina Laine
Majlinda Lako
Jerson Laks
Madepalli K. Lakshmana
Hon-Ming Lam
Wendy Wing Tak Lam
Wilbur Lam
Christophe Lamaze
Eric Gordon Lamb
Cornelis B. Lambalk
Sergio A. Lambertucci
Renaud Lambiotte
Maya Dimova Lambreva
Claus Lamm
Mikko Juhani Lammi
Severine Lamon
Ruth Landau
Alan Landay
Nicoletta Landsberger
Julia Lane
Scott M. Langevin
Berthold Langguth
Thomas Langmann
Gordon Langsley
Lucia R. Languino
Humberto Lanz-Mendoza
Markus Lappe
Alexander Larcombe
Vincent Larivière
Peter E. Larsen
Charles R. Larson
Bruce A. Larson
Corinne Ida Lasmezas
Joerg Latus
Philipp Latzin
Andy T. Y. Lau

Eric Hy Lau
Wan Yee Joseph Lau
Jörn Lausen
Derek Laver
Pascal M. Lavoie
Matthew Law
Michael Barton Laws
Victoria Lawson
Claudio R. Lazzari
Chiara Lazzeri
Weidong Le
Bernard Le Foll
Herve Le Stunff
Walter S. Leal
Camille Lebarbenchon
Mikhail A. Lebedev
Nikolai Lebedev
Irina V. Lebedeva
Binnaz Leblebicioglu
Sean Bong Lee
Ji-Hyun Lee
Ju-Seog Lee
Jung Weon Lee
Jung Eun Lee
Kyung S. Lee
Bok-Luel Lee
Samuel A. Lee
Hyun-Sung Lee
Yin-Won Lee
Leo T. O. Lee
Yungling Leo Lee
Jung Ryeol Lee
Seon-Woo Lee
Seungbok Lee
Pei-Lin Lee
Myon-Hee Lee
Albert Lee
Sang H. Lee
I-Ching Lee
Seok-Geun Lee
Joohyung Lee
Chon-Lin Lee
Alexander Leemans
Jason R. Lees
Markos Leggas
Giuseppe Legname
R. Michael Lehman
Sune Lehmann
Hans-Joachim Lehmler
Benfang Lei
Xu Lei
Nic D. Leipzig
Luciana C. C. Leite
Maria Leite-de-Moraes
José A. Lemos
Megan D. Lenardon
Wayne I. Lencer
Fenfei Leng
Christophe Lenglet
Laurel L. Lenz
Tiziana Leone
Zoya Leonenko
Christine Leong
Livia Leoni
Christopher A. Lepczyk
Christophe Leroyer
Andres G. Lescano
Gregory Lesinski
Edward J. Lesnefsky
Adam Lesner
Maciej S. Lesniak
Olivier Lespinet
Yuk Fai Leung
Sima Lev
Yaakov Koby Levy
Raphael Levy
Alfred S. Lewin
Patrick Lewis
Joel Lexchin
Xiao-Jiang Li
Tiansen Li
Ziyin Li
Yuqing Li
Lixiang Li
Xia Li
Kui Li

Lei Li
Shengxu Li
Songhai Li
Zhenyu Li
Maoteng Li
Qizhai Li
Xia Li
Chengdao Li
Bo Li
Wan-Ju Li
Yan Li
Wei Li
Yi Li
Yun Li
Dongmei Li
Mei Li
Suxia Li
Xiangzhen Li
Jin-Tian Li
Cheng–Sen Li
Xiangtao Li
Jian Jian Li
Xiang Li
Zongjin Li
Yi Li
Xiu-Qing Li
Giovanni Li Volti
Feng Liang
Peipeng Liang
Mingzhi Liao
Daniel H. Libraty
Mathias Lichterfeld
Karen Lidzba
Stefan Liebner
Michael Lierz
Marc Liesa
Jonathan Lifshitz
David A. Lightfoot
Kah-Leong Lim
Yong Pyo Lim
Viviane D. Lima
Federica Limana
Baochuan Lin
Jinxing Lin
Ching-Po Lin
Senjie Lin
Wenyu Lin
Han-Chieh Lin
Zhicheng Carl Lin
Chung-Ying Lin
Fa-Hsuan Lin
Ying-Ju Lin
Hai-Yan Lin
Meng C. Lin
Wen-Xiong Lin
Rafael Linden
Erjun Ling
Feng Ling
Ralf A. Linker
Igor Linkov
Vincenzo Lionetti
Stamatis-Nick Liossis
Michael J. Lipinski
Frederique Lisacek
Paloma B. Liton
Yong Liu
Zhong-Jian Liu
Chunming Liu
Chunyu Liu
Ji-Hong Liu
Pan-Ping Liu
Xuefeng Liu
Jian Liu
Bin Liu
Guei-Sheung Liu
Guangwei Liu
Jinny L. Liu
Hanjun Liu
Jian-Guo Liu
Nan Liu
Chaojie Liu
Zheng Liu
Chen-Hua Liu
Xiaohua Liu
Alexander V. Ljubimov
Anthony W. I. Lo

Kwok-Wai Lo
Chung-Ming Lo
Jean-Marc A. Lobaccaro
Alessio Lodola
David M. Loeb
Marie Lof
Xian Jun Loh
Steven Arthur Loiselle
Bruno Lomonte
David Long
Travis Longcore
Russell R. Lonser
Juan J. Loor
Guillermo López Lluch
Miguel Lopez-Ferber
Cecilio López-Galíndez
Marcelo Gustavo Lorenzo
Christopher J. Lortie
Stefan Lötters
Edward J. Louis
Matthieu Louis
Stelios Loukides
Denis Loustau
Jason B. Love
Connie Lovejoy
Christian Lovis
Sergi Lozano
Lin Lu
Rui Lu
Tao Lu
Sheng-Nan Lu
Hua Lu
Qing Lu
Wang-Jin Lu
Yoel Lubell
Paul Lucas
Alejandro Lucía
Fabio Lucidi
John Luk
Lewis Lukens
Peter Lundberg
Zhao-Qing Luo
Feng Luo
Hong Luo
Wenbo Luo
Jia Luo
Xi Luo
Zhong-Cheng Luo
Nan Luo
Yi Luo
Raul M. Luque
Arthur J. Lustig
Heike Lutermann
Esther Lutgens
Dawn Sywassink Luthe
Aernout Luttun
Adrian J. F. Luty
Andrea Luvisi
Zhihan Lv
Lisa Carlson Lyons
Grant Lythe
William W. Lytton
Wujun Ma
Jun Ma
Daqing Ma
Wen-Lung Ma
Jiyan Ma
Xin-Liang Ma
Zheng-Liang Ma
Xiaolei Ma
Yan Ma
Stefan Maas
Andrea Macaluso
Ben D. MacArthur
Roberto Macchiarelli
Guy A. MacGowan
Ricardo Bomfim Machado
Miguel Machuqueiro
Brian R. MacKenzie
Michele Madigan
Shiro Maeda
Giovanni Maga
Marco Magalhaes
Vanesa Magar
Laura A. Magee
Sanjay B. Maggirwar

Ramamurthy Mahalingam
Brion Maher
Andrew R. Mahon
Andre Maia Chagas
Domenico Maiorano
Kristen C. Maitland
Carl G. Maki
Makoto Makishima
Luc Malaval
Jesus E. Maldonado
Luis Angel Maldonado
Manjarrez
Rayaz Ahmed Malik
Krishna M. G. Mallela
Bibekanand Mallick
Moises Mallo
Manuel S. Malmierca
Jesús Malo
Fabrizio Mammano
Kwan Man
Ichiro Manabe
Emmanuel Manalo
Shekhar C. Mande
Mercedes Susan Mandell
Olivia Manfrini
Riccardo Manganelli
Balaji Manicassamy
Richard Mankin
Arto Mannermaa
Ben J. Mans
Ed Manser
Ashham Mansur
Nicholas J. Mantis
Roberto Mantovani
Lamberto Manzoli
Jian-Hua Mao
Jingdong Mao
Tapio Mappes
Antonella Marangoni
Miguel Maravall
Alessandro Marcello
Massimo Marchiori
Franck Marchis
Alessandra Marengoni
Antoni Margalida
Leonid Margolis
Daniel S. Margulies
Bernard Mari
Christophe Mariat
Daniele Marinazzo
Claudio Romero Farias Marinho
Leonardo Mariño-Ramírez
Frederic Marion-Poll
Wanda Markotter
Andrew C. Marr
Vicki Marsh
James A. R. Marshall
Christopher D. Marshall
Lorna Marson
Fabio Martelli
Stephen J. Martin
Sally Martin
Federico Martinelli
Francesco Martines
Jose Angel Martinez Climent
Francisco Martinez-Abarca
Susana Martinez-Conde
Silvia Martínez-Llorens
Ligia O. Martins
Andrea Martinuzzi
Michael Massiah
Ramin Massoumi
Naoki Masuda
Juan Mata
Vikram Mathews
Timothy C. Matisziw
Minami Matsui
Jonathan I. Matsui
Takuya Matsumoto
Hiroaki Matsunami
Yutaka J. Matsuoka
Michiya Matsusaki
Sachiko Matsuzaki
Atsushi Matsuzawa
Joseph J. Mattapallil
Fabrizio Mattei

Pallab K. Maulik
Natasha M. Maurits
Robin Charles May
Clarissa Menezes Maya-Monteiro
Olga Mayans
Claudine Mayer
Sylvie Mazoyer
Rachid Mazroui
Marianna Mazza
Silvia Mazzuca
Grainne Mary McAlonan
Brian D. McCabe
Joseph H. McCarty
James M. McCaw
Kevin McCluskey
David L. McCormick
Jean L. McCrory
Scott W. McCue
James Edgar McCutcheon
Scott McDonald
Andrew McDowell
Sheena McGowan
Alistair P. McGregor
Melvin G. McInnis
David D. McKemy
Peter John McKenna
Christopher W. McKindsey
Margaret E. McLaughlin-Drubin
Ian McLoughlin
Paul McNeil
James P. Meador
Andrea Mechelli
Warren H. Meck
Stephan Meckel
Miguel Angel Medina
Rajesh Mehrotra
Kapil Mehta
Anand S. Mehta
Lin Mei
Ulrich Melcher
Stefano Meletti
Jaymie Meliker
Duane Mellor
Rossana C. N. Melo
Jose Melo-Cristino
Marco Meloni
Carlo Meloro
Maeli Melotto
Frank Melzner
John E. Mendelson
Liset Menendez de la Prida
Luis Menéndez-Arias
Gustavo Batista Menezes
Zhefeng Meng
Adina Maya Merenlender
Peter Mergaert
William H. Merigan Jr.
Bruno Merk
Roeland M. H. Merks
Tesfaye B. Mersha
Frédéric Mertens
Marc W. Merx
Alex Mesoudi
Ilhem Messaoudi
Claudia Mettke-Hofmann
Konradin Metze
Dennis W. Metzger
Eliane F. Meurs
Axel Meyer
Craig Meyers
David Meyre
Dengshun Miao
Pawel Michalak
Kristin Michel
Claudia Miele
Martine Migaud
Antimo Migliaccio
Cathy Mihalopoulos
Matthew C. Mihlbachler
Takeshi Miki
Manlio Milanese
Peter M. Milgrom
Frederick W. Miller
Todd Miller
Allison Miller

Mark Webber Miller
Francis Miller Jr.
Ken Mills
Xiang Jia Min
Kyung-Jin Min
Masabumi Minami
Tohru Minamino
Budiman Minasny
Jens Minnerup
Eric M. Mintz
Barbara Mintzes
Monica Miozzo
Vincenzo Miragliotta
Harald Mischak
Dan Mishmar
Yogendra Kumar Mishra
Ramesh Kumar Mishra
Rajeev Misra
Fanis Missirlis
Nerges Mistry
Caroline Mitchell
Nandita Mitra
Neena Mitter
Eliane N. Miyaji
Sayuri Miyamoto
Toshiyuki Miyata
Norikatsu Miyoshi
Emiko Mizoguchi
Itzhak Mizrahi
Mohammad R. K. Mofrad
Masaki Mogi
Rajiv R. Mohan
Jacqueline Mohan
Rajesh Mohanraj
Subhra Mohapatra
Pierre Moine
Imelda K. Moise
Christine Moissl-Eichinger
Ethan Moitra
Igor Mokrousov
Juan Carlos Molinero
Bertrand Mollereau
Tapan Kumar Mondal
Mario U. Mondelli
Alexander A. Mongin
Daniel Monleon
Ali Montazeri
Courtney G. Montgomery
Darren J. Moore
Anne C. Moore
Spencer Moore
Ana Mora
Josué de Moraes
Paula V. Morais
Maria Moran
Luciano Andrade Moreira
Nei Moreira
Francisco Moreira
Silvia N. Moreno
Edgardo Moreno
Yamir Moreno
Eduardo Moreno
Gabriel Moreno-Hagelsieb
Gregorio Moreno-Rueda
Rafael Moreno-Sanchez
José A. Morgado-Díaz
Keisuke Mori
Lisa A. Morici
Takaya Moriguchi
Flaviano Morone
Andrea Morrione
Alessio Mortelliti
Kevin Mortimer
J. Bruce Morton
Rory Edward Morty
Antonio Moschetta
George Mosialos
Csaba Moskát
R. Lee Mosley
Joël Mossong
M. D. A. Motaleb
Michael A. Motes
Jean-Pierre Mothet
Andrea Motta
Sophie Mouillet-Richard
Vincent Mouly

Catherine Mounier
Ivan Cruz Moura
André Mouraux
Laurent Mourot
Parvin Mousavi
Tim A. Mousseau
Jordi Moya-Larano
Rosa Maria Affonso Moysés
Riccardo Mozzachiodi
Jorge Marin Mpodozis
Tarek Msadek
Michael Muders
Mohana Krishna Reddy Mudiam
Scott N. Mueller
Ken-Ichi Mukaisho
Amitava Mukherjee
Partha Mukhopadhyay
Marc Muller
Rolf Müller
Bertram Müller-Myhsok
Gabriele Multhoff
Srinivas Mummidi
Cesar V. Munayco
Ulrike Gertrud Munderloh
Rachata Muneepeerakul
Gnanasekar Munirathinam
Craig Murdoch
William J. Murphy
Ashlesh K. Murthy
Antonio Musaro
Gokhan M. Mutlu
Marco Muzi-Falconi
Garry Stewart Anthony Myers
Ivan R. Nabi
Angel Nadal
Sathyamangla Venkata Naga Prasad
Ram Nagaraj
Takeshi Nagasaka
Michael Nagler
Julian R. Naglik
Brahim Nait-Oumesmar
Behzad Najafian
Joseph Najbauer
Rafael J. Najmanovich
Hiroyasu Nakano
Sang-Chul Nam
Jin Nam
Rajasekaran Namakkal Soorappan
Mahesh Narayan
Raja Narayanan
Christine Nardini
Marko Nardini
Fabio S. Nascimento
Marcelle Nascimento
Serge Nataf
Kalimuthusamy Natarajaseenivasan
Urs M. Nater
Dhruba Naug
Alfons Navarro
Carlos A. Navas
Hiroyuki Nawa
Tim S. Nawrot
Daniel E. Naya
Ara Nazarian
Lishomwa C. Ndhlovu
Brett Neilan
Robert Nerenberg
Pratibha V. Nerurkar
Luis Eduardo Soares Netto
Stephan C. F. Neuhauss
Michael Nevels
Richard David Newcomb
Peter A. Newman
Robert L. Newton
Olivier Neyrolles
Carl Ng
Lisa F. P. Ng
Quan Sing Ng
Doan Tm Ngo
Tuan Van Nguyen
Henry T. Nguyen
M. Hong Nguyen

Xijun Ni
Raymond Niaura
Joseph T. Nickels
Mark Patrick Nicol
Daotai Nie
Feiping Nie
Thomas Niederkrotenthaler
Randall P. Niedz
James C. Nieh
Morten Nielsen
Kirsten Nielsen
Heiner Niemann
William C. Nierman
Jérôme Nigou
Dragana Nikitovic
Nikolas Nikolaidis
Georgios K. Nikolopoulos
Daisuke Nishi
Noriyuki Nishida
Hisao Nishijo
Takashi Nishikawa
Wataru Nishimura
Hiroshi Nishiura
Aleksandra Nita-Lazar
Douglas F. Nixon
Antal Nógrádi
Ruben Nogueiras
Anna Nolan
Abdisalan Mohamed Noor
Giuseppe Danilo Norata
Antoine Nordez
Saima Noreen
Mohd Noor Norhayati
David G. Norman
Christopher M. Norris
William H. J. Norton
Francois Nosten
Giuseppe Novelli
Giuseppina Novo
Ariel Novoplansky
Minou Nowrousian
Ulrich Nübel
Rachel A. Nugent
Syam Nukavarapu
Olga Cristina Pastor Nunes
Dmitry I. Nurminsky
Michael Nurmohamed
Howard Nusbaum
Chukwumere Nwogu
George-John Nychas
Joshua J. Obar
Assad Anshuman Oberai
Monika Oberer
Tatiana M. Oberyszyn
Alexander G. Obukhov
Bonnie O'Connor
Agricola Odoi
Olorunseun Ogunwobi
Andreas Ohlmann
Masuo Ohno
Yoshikazu Ohya
Naoki Oiso
David M. Ojcius
Toshiyuki Ojima
Jason F. Okulicz
Baldo Oliva
Pedro L. Oliveira
Jake Olivier
Jeff Ollerton
Kenneth M. Olsen
Michael F. Olson
Donald R. Olson
I. Anna S. Olsson
Michal A. Olszewski
Bolajoko O. Olusanya
Abdelwahab Omri
Tolu Oni
N. Charlotte Onland-Moret
Eng Eong Ooi
Yutaka Oono
Don Operario
Covadonga Orejas
Matej Orešič
Joseph P. R. O. Orgel
Ludovic Orlando
Mohammed S. Orloff

Sandra Orsulic
Pavel I. Ortinski
Justin R. Ortiz
Robert Oshima
Daniel Osorio
Henrik Oster
Henrik Österblom
Oksana Ostroverkhova
Mario A. Ostrowski
Marisa Otegui
Geir Ottersen
Michael Otto
Cees Oudejans
Michel M. Ouellette
Tim D. Oury
Christos A. Ouzounis
Ofer Ovadia
Cristina Óvilo
Gozde Ozakinci
Egon Anderson Ozer
Antonio G. Pacheco
Emanuele Paci
Eugenio Paci
Davide Pacini
Svetlana Pack
Jaya Padmanabhan
Parasuraman Padmanabhan
Johnny Padulo
Joseph S. Pagano
Cristiano Pagnini
Madhukar Pai
Rebecca Painter
Coro Paisan-Ruiz
Samuel Rezende Paiva
Utpal Pal
Soumitro Pal
Elisabetta Palagi
Senthilnathan Palaniyandi
Nades Palaniyar
Francesc Palau
Antonio Palazón-Bru
Alexander F. Palazzo
Swati Palit Deb
Pierlorenzo Pallante
Komaraiah Palle
Subba Reddy Palli
Johan Pallud
Nicholette D. Palmer
Arnar Palsson
An Pan
Jingxuan Pan
Xiaoping Pan
Chongle Pan
Chen-Wei Pan
Koustubh Panda
Gunjan Pandey
Siyaram Pandey
Girdhar K. Pandey
Udai Pandey
Maharaj K. Pandit
Alexander V. Panfilov
Xiaoming Pang
Aditya Bhushan Pant
Kostas Pantopoulos
Roberto Papa
Salvatore Papa
Gianpaolo Papaccio
Christos Papadelis
Nikos T. Papadopoulos
Elena Papaleo
Thalia Papayannopoulou
Tamás Papp
Francesco Pappalardo
Hanu R. Pappu
Rodolfo Paranhos
Dimitrios Paraskevis
P. Pardha-Saradhi
Daniel Paredes-Sabja
Swarup Kumar Parida
Satya Parida
Nehal A. Parikh
Tanya Parish
Sohee Park
Man-Seong Park
Deric M. Park
Jong-In Park

Emily J. Parker
John Parkinson
Aristeidis Parmakelis
Marie-Laure Parmentier
Alessandro Parolari
Janet F. Partridge
Suhel Parvez
Louis R. Pasquale
Alberto G. Passi
Claudio Passino
Geraldo A. Passos
Annalisa Pastore
Kevin Paterson
Santosh K. Patnaik
Jayadeep Patra
Heather M. Patterson
Tommy Pattij
Hemant K. Paudel
Annika Paukner
Richard Paul
Friedemann Paul
Gianni Pavan
Martin Pavelka
Marina A. Pavlova
Jodi Pawluski
William A. Paxton
Jorge Paz-Ferreiro
Valerio Pazienza
Cameron Peace
Eve-Isabelle Pecheur
Robert N. Pechnick
Myron Peck
Shyamal D. Peddada
Wendy A. Peer
Beatriz Pelacho
Sergio Pellis
Leandro Peña
Ramona Natacha Pena I Subirà
Gurudutt Pendyala
Zuogang Peng
Marzio Alfio Pennisi
Thomas Penzel
José César Perales
Manuel Perea
Luísa Maria Sousa Mesquita Pereira
Lygia V. Pereira
Inês A. Cardoso Pereira
Marco Peresani
María Angeles Pérez
Antonio Perez-Martinez
Eugene A. Permyakov
Carlo Federico Perno
Dragan Perovic
Mark Allen Pershouse
Matthew Anthony Perugini
Lorenzo Peruzzi
Maurizio Pesce
Hans-Ulrich Peter
Michael Peters
Karin E. Peterson
Jonathan M. Peterson
Kathy Petoumenos
Michael D. Petraglia
Pier Giorgio Petronini
Sarah L. Pett
Salvatore Petta
Sebastien Pfeffer
Dzung Pham
Thanh G. Phan
William D. Phillips
Wayne A. Phillips
Robert S. Phillips
Andrew Philp
Maria Francesca Piacentini
Didier Picard
Mauro Picardo
Ciriaco A. Piccirillo
Raymond J. Pickles
Marcio Pie
Massimo Pietropaolo
Jakob Pietschnig
Giuseppe Pignataro
Jay Pillai
Ron Pinhasi
João Pinto

Gianfranco Pintus
Paolo Piras
Matteo Pirro
Alexander N. Pisarchik
Dario Pisignano
Juan Carlos Pizarro
Carmine Pizzi
Salvatore V. Pizzo
Josep V. Planas
Emmanuel Planel
Paul J. Planet
Robert Planque
Michelina Plateroti
Alan Graham Pockley
Boris Podobnik
Stefanie Pöggeler
Stefan Pöhlmann
Lakshminarayana Polavarapu
Irina Polejaeva
Guido Poli
Michael Polymenis
Jean-François Pombert
Daniela Ponce
Giovanni Ponti
Mikhail M. Pooggin
Venuprasad K. Poojary
Art F. Y. Poon
Michel R. Popoff
Alexey Porollo
Enzo Porrello
James Porter
Manuel Portero-Otin
Manuel Portolés
Maarten Postma
Marie-Claude Potier
Benjamin Poulter
Nader Pouratian
Pedro Póvoa
Sriharsa Pradhan
Gustavo Pradilla
Sean Michael Prager
Manoj Prasad
Antje Prasse
Stephen C. Pratt
Tobias Preis
Thomas Preiss
Louis S. Premkumar
Garrett Prestage
Matt A. Price
Brendan D. Price
Nicholas Seow Chiang Price
Claude Prigent
Josef Priller
Shankar Prinja
Kathleen R. Pritchett-Corning
Ludmila Prokunina-Olsson
Vasilis J. Promponas
Paul Pronyk
Roberto Pronzato
Paul Proost
Stephen R. Proulx
Michael J. Proulx
Nicholas J. Provart
Paolo Provero
Federica Provini
Patrick Prunet
Reeta Prusty Rao
Jude Marek Przyborski
Maurice Ptito
Read Pukkila-Worley
Louise Purton
Krzysztof Pyrc
Qian Qian
Zhaohui Qin
Xinghui Qiu
Jianming Qiu
Gao-Feng Qiu
Chao Qiu
Caroline Quach
Federico Quaini
Zhe-Xue Quan
Dale A. Quattrochi
Antonietta Quigg
Petra Quillfeldt
Janet Quinn
Terence J. Quinn

Luis Eduardo M. Quintas
Alexander Rabchevsky
Thierry Rabilloud
Mitchell Rabinowitz
Craig A. Radford
Filippo Radicchi
Gajendra P. S. Raghava
Ram K. Raghavan
Yvan Rahbé
Mahfuzar Rahman
Abidur Rahman
M. Sohel Rahman
Pasquale Raia
Nigel E. Raine
Sari Helena Räisänen
Johnson Rajasingh
Istvan Rajcan
Raghavan Raju
Zoltán Rakonczay Jr.
Stuart Raleigh
Stuart Alexander Ralph
Sreeram V. Ramagopalan
Harsh Raman
Rajesh Ramanathan
Jose Javier Ramasco
Ramani Ramchandran
Pranela Rameshwar
Joe W. Ramos
Lennart Randau
Paul A. Randazzo
Shoba Ranganathan
Christopher V. Rao
Hengyi Rao
A. L. N. Rao
A. Gururaj Rao
Cedric Raoul
Fabio Rapallo
Arash Rashed
Randall Lee Rasmusson
Nadine Ravel
Ann Rawkins
John F. Rawls
Ratna B. Ray
Ranjit Ray
Imran Razzak
Francisco X. Real
Hugo Rebelo
Gianpaolo Reboldi
David Reby
Sakamuri V. Reddy
Hemachandra Reddy
Jay Reddy
Gadi V. P. Reddy
Aaron W. Reed
Phil Reed
Judy R. Rees
Thomas A. Reh
Nicholas G. Reich
Sean Reid
Manuel Joaquín Reigosa
Gwendolen Reilly
Markus Reindl
David J. Reiner
Kurt O. Reinhart
Rui Manuel Reis
Johannes Reisert
Gernot Reishofer
Giuseppe Remuzzi
Lei Ren
Alvaro Rendon
Jyothi Rengarajan
Giuseppe Rengo
Laurent Rénia
Michel Renou
Gourapura J. Renukaradhya
Andre M. N. Renzaho
Stephen C. Resch
Sylvie Rétaux
Constantino Carlos Reyes-Aldasoro
Human Rezaei
Rita Rezzani
Jong Rho
Francesco Ria
Domenico Ribatti
Zaccaria Ricci

Fabio Luigi Massimo Ricciardolo
Benjamin Edward Rich
Yolande Richard
Kristy L. Richards
Bryce Richardson
Thomas L. Richie
Mark Simonds Riddle
Dean E. Riechers
Christian U. Riedel
Frederic Rieux-Laucat
Howard Riezman
Paul D. Riggs
Bruce B. Riley
Riikka Rinnan
Arun Rishi
Praveen Rishi
Marco Rito-Palomares
Susan R. Rittling
Daniel Rittschof
Christian Rixen
Seungil Ro
Stanley J. Robboy
David D. Roberts
Tony Robillard
D. Ashley Robinson
Marc Robinson-Rechavi
Craig N. Robson
Alfred L. Roca
Danilo Roccatano
Duccio Rocchini
Luis M. Rocha
Sonia Rocha
Flavio Rocha
Daniel Rockey
Luigi F. Rodella
Marcio L. Rodrigues
Fernando Rodrigues-Lima
B. Rodríguez
Antoni Rodriguez-Fornells
Carlos M. Rodriguez-Ortigosa
Cesar Rodriguez-Saona
Francisco Rodriguez-Valera
Ryan K. Roeder
Bernard Aj Roelen
Klaus Roemer
Lesley Joy Rogers
Igor B. Rogozin
Tae-Young Roh
Myung-Il Roh
Holger Rohde
Sabine Rohrmann
Sergio R. Roiloa
Antonis Rokas
Louise A. Rollins-Smith
Peter G. Roma
Gregg Roman
Stephanie S. Romanach
Maria Fiammetta Romano
Andrej A. Romanovsky
Floyd Romesberg
Andrea Romigi
Suzan H. M. Rooijakkers
Ilse Rooman
Roy Martin Roop II
Yan Ropert-Coudert
Pierre Roques
Rafael Rosell
James T. Rosenbaum
Karen Rosenberg
Paul B. Rosenberg
Peter Rosenberger
Joshua L. Rosenbloom
Cheryl S. Rosenfeld
Pauline Ross
Sergio Rossi
Rossella Rota
Marcello Rota
Martin E. Rottenberg
Martin Rottman
Hatem Rouached
Tracey Rouault
Jean-Pierre Rouault
Philippe Rouet
Anna Roujeinikova
Alexandre Roulin
Jagat Kumar Roy

Daniel E. Rozen
Celine Rozenblat
Elena A. Rozhkova
Zhi Ruan
Jianhua Ruan
Dustin Rubenstein
Daniel L. Rubin
Tiziana Rubino
Boris Rubinsky
Uwe Rudolph
Olav Rueppell
Florence Ruggiero
Jodie L. Rummer
Mark A. Runco
Klemens Ruprecht
Bayden D. Russell
Charles J. Russell
Michael A. Russello
Danilo Russo
Emilio Russo
Suzannah Rutherford
Gerard Roel Rutteman
Benjamin I. Ruttenberg
Ilya Ruvinsky
Andrey E. Ryabinin
Sadie Jane Ryan
Valentin V. Rybenkov
Kelli K. Ryckman
Bernhard Ryffel
Bart Rypma
Wang-Shick Ryu
Choong-Min Ryu
Hoon Ryu
Andrey Rzhetsky
Jamil S. Saad
Hatem E. Sabaawy
Massimo Sacchetti
Geetanjali Sachdeva
Emma Sacks
Sakthivel Sadayappan
Scheherazade Sadegh-Nasseri
Junichi Sadoshima
Bhaskar Saha
Upendra M. Sainju
Leonor Saiz
Manabu Sakakibara
Kentaro Q. Sakamoto
Naoya Sakamoto
Jon T. Sakata
Dennis Salahub
Mohammad Saleem
Fadi N. Salloum
Freddie Salsbury Jr.
Mauro Salvi
Siba K. Samal
Rajeev Samant
Dino Samartzis
Suryaprakash Sambhara
Dorit Samocha-Bonet
Jaime Sampaio
Anthony Peter Sampson
Michel Samson
James E. Samuel
David C. Samuels
Javier Sanchez
Zila M. Sanchez
Víctor Sánchez-Margalet
Jose M. Sanchez-Ruiz
Øyvind Sandbakk
Johan K. Sandberg
Devinder Sandhu
Jeff M. Sands
Paul Sandstrom
Qing-Xiang Amy Sang
Miguel A. F. Sanjuán
Osman Alimamy Sankoh
Anderson de Souza Sant'Ana
Fabio Santanelli di Pompeo d'Illasi
Mario L. Santiago
Miguel Santin
Daniele Santini
Hélder A. Santos
Janine Santos
Subhabrata Sanyal
Yolanda Sanz

Anna Sapino
Ligia M. Saraiva
Pierangelo Sardo
Gabriele Saretzki
Devanand Sarkar
Sabrina Sarrocco
Güher Saruhan-Direskeneli
Marinko Sarunic
Daimei Sasayama
Hidenori Sassa
Juan Sastre
Krish Sathian
Makoto Sato
Minoru Satoh
Abhay R. Satoskar
Sevtap Savas
Hideyuki Sawada
Amr H. Sawalha
Robert Gary Sawers
Nancy M. Sawtell
Enrico Scalas
Monica Scali
Giuseppe Scapigliati
Maria Rosaria Scarfi
Aldo Scarpa
Cristoforo Scavone
Eliana Scemes
Matthew B. Schabath
Joseph Schacherer
Martin Schädler
Andreas Schäfer
Gerwin Schalk
Henk D. F. H. Schallig
Luis M. Schang
Dena L. Schanzer
Peter Schausberger
Andre Scherag
Roberta W. Scherer
Jay D. Schieber
Bernd Schierwater
Raphael Schiffmann
Oliver Schildgen
Niels O. Schiller
Michael Schindler
Felix Schlachetzki
Stefan Schlatt
Tamar Schlick
Jens Schlossmann
Christian Schmahl
Harald H. H. W. Schmidt
Jennifer V. Schmidt
Edward E. Schmidt
Benedikt R. Schmidt
Ulrike Schmidt
Axel K. Schmitt
Francois G. Schmitt
Fernando Schmitt
Jürgen Schmitz
Mirco Schmolke
Monika Schmoll
Lynn M. Schnapp
Bradley S. Schneider
Marlon R. Schneider
Joel M. Schnur
Andrew Scholey
Henrike Scholz
Christian Schönbach
C. Mary Schooling
Veronika Schöpf
Francisco J. Schopfer
Gideon Schreiber
Katrin Schröder
Michael Schubert
Raymond Schuch
Peter Schuck
Markus Schuelke
Christian Schulz
David J. Schulz
Guy J. P. Schumann
Wolf-Hagen Schunck
Simone Schütz-Bosbach
Adam J. Schwarz
Oliver Schweiger
Luitgard Schwendenmann
Friedhelm Schwenker
Christian Schwentner

Enzo Pasquale Scilingo
Jamie Kathleen Scott
Maxwell John Scott
Hazel R. C. Screen
Thomas J. Scriba
Tiffany Seagroves
Rebecca Sear
Michael Sears
Leonardo A. Sechi
Timothy W. Secomb
Soraya Seedat
Antonio Carlos Seguro
Shamala Devi Sekaran
Mohamed N. Seleem
Gernot Sellge
Vimal Selvaraj
Linda A. Selvey
Szabolcs Semsey
Utpal Sen
Irene Sendiña-Nadal
Chaminda Jayampath Seneviratne
Shantanu Sengupta
John M. Senko
Masaharu Seno
Balasubramanian Senthilkumaran
Jeong-Sun Seo
Giuseppe Sergi
Guido Serini
Rosa Serra
Emmanuel Serrano Ferron
J. Seshu
Karol Sestak
Jaswinder K. Sethi
Gautam Sethi
Peter Setlow
Sergey Shabala
William M. Shafer
Lion Shahab
Mohammad Shahid
Jeffrey Shaman
Simon Francis Shamoun
(Amir) Homayoun Shams
Bin Shan
Hong Shang
Esaki M. Shankar
Sharmila Shankar
Bhavani Shankar
Renfu Shao
Mark S. Shapiro
Igor V. Sharakhov
Jyotika Sharma
K. Krishna Sharma
Manu Sharma
Pushpa Sharma
Dror Sharon
V. Prasad Shastri
Hagit Shatkay
Peter Shaw
Matthew Shawkey
Gregory Shearer
Eric A. Shelden
Yiqun G. Shellman
Rulong Shen
Han-Ming Shen
Wei Shen
Michael Sherman
Jonathan H. Sherman
Salah A. Sheweita
Qinghua Shi
Lei Shi
Wei Shi
Xing-Ming Shi
Honglian Shi
Haitao Shi
Yuyan Shi
Xuan-Zheng Shi
Paul Gerard Shiels
Clive Shiff
Yen-Yu Ian Shih
Chiaho Shih
Hiroshi Shiku
Eiji Shimizu
Tatsuo Shimosawa
Eui-Cheol Shin

Jae-Ho Shin
Toshi Shioda
Shin-Han Shiu
Ralph V. Shohet
Noam Shomron
Neal Shore
Yogesh S. Shouche
Naglaa H. Shoukry
Anil Shrestha
Viji Shridhar
Mark G. Shrime
Alexander Chong Shu-Chien
Deepak Shukla
Arun Shukla
Pratyoosh Shukla
Rosely Sichieri
Luisa Siculella
Allan Siegel
Britta Siegmund
Mariano Sigman
Israel Silman
Lucas C. R. Silva
Alessandro Silvani
Olivier Silvie
Robert B. Sim
J. Pedro Simas
Umberto Simeoni
Sidney Arthur Simon
Michel Simon
George Simos
Roberta Sinatra
Aran Singanayagam
Andrew C. Singer
Florian Singer
Ravindra N. Singh
Shree Ram Singh
Brij Singh
Jaswinder Singh
Lalit Pukhrambam Singh
Amit Singh
Pankaj K. Singh
Udai P. Singh
Kamaleshwar P. Singh
Keshav K. Singh
Tiratha Raj Singh
Ajay Pratap Singh
Mohar Singh
Dinender K. Singla
Photini Sinnis
Anna-Leena Sirén
Angela Sirigu
Sodiomon Bienvenu. Sirima
Cesare R. Sirtori
Giovanni Sitia
Efthimios M. C. Skoulakis
Mikael Skurnik
David A. Slattery
Kristel Sleegers
Katie Elizabeth Slocombe
Andrzej T. Slominski
Rob Slotow
Nicolas Sluis-Cremer
Guy Smagghe
Neil R. Smalheiser
Richard Jay Smeyne
Hauke Smidt
Tara C. Smith
Kenny Smith
Brenda Smith
Alastair Smith
Thierry Smith
Lachlan J. Smith
Zachary A. Smith
Matthew E. Smith
Victoria C. Smith
Wm. Leo Smith
Mary C. Smith Fawzi
Michael Smotherman
Georges Snounou
Paula Soares
Claudio M. Soares
Robert W. Sobol
Kenneth Söderhäll
Irene Söderhäll
José L. Soengas
Alexander N. Sokolov

Igor Sokolov
Bernd Sokolowski
Maria Sola
Christophe Sola
Aldo Solari
Alessandra Solari
Thierry Soldati
Ricard V. Solé
Samuel G. Solomon
Ilia Solov'yov
Ken Solt
Christopher M. Somers
Martin Sommer
Claudia Sommer
Monica Soncini
Qing Song
Yiqing Song
Ping Song
Xiao Song
Chunhua Song
Linsheng Song
Young-Hwa Song
Silvia C. Sookoian
Tuck Wah Soong
Carles Soriano-Mas
Giovanni Sotgiu
Erik Sotka
John Souglakos
Mohammed Soutto
Vanessa Souza-Mello
Ali A. Sovari
Graça Soveral
J. David Spafford
Pieter Spanoghe
Paul Spearman
Roberto F. Speck
Matthaios Speletas
Juliet V. Spencer
Markus Sperandio
Niko Speybroeck
Tobias Spielmann
Maria Spies
Mark Spigelman
Charalampos Spilianakis
Donatella Spinelli
Gaia Spinetti
Cassandra Nichole Spracklen
Rosanna Squitti
Srinand Sreevatsan
Narayanaswamy Srinivasan
Manoj Srinivasan
Srinivasa M. Srinivasula
Robert B. Srygley
Beate St Pourcain
Francesco Staffieri
Simona Stäger
Harald Staiger
Lucas J. Stal
Emmanuel Andreas Stamatakis
Martin Stangel
Jo-Ann L. Stanton
Roscoe Stanyon
Daniel T. Starczynowski
Peter Starkel
Randi Starrfelt
J. Christopher States
Catherine A. Staton
Roland Steck
Robert E. Steele
Gary Stein
Dieter Steinhilber
Jena J. Steinle
Dov Joseph Stekel
Salomon M. Stemmer
Andreas Stengel
Ian D. Stephen
Stanislaw Stepkowski
Nigel K. Stepto
Annette Sterr
Brian Stevenson
James P. Stewart
Robert Stewart
Ewout W. Steyerberg
Heinrich Sticht
Knut Stieger
Ymkje Stienstra

Jonathan Stiles
Matthias Stöck
Cheryl A. Stoddart
Georg Stoecklin
Tobias Stoeger
Keith Stokes
Gustavo Stolovitzky
Michael J. Stout
Jose Antonio Stoute
Cordula M. Stover
Adam Stow
Yves St-Pierre
Aaron F. Straight
Olaf Strauß
Pavel Strnad
Martina Stromvik
Paul C. Struik
Collin M. Stultz
Roger Sturmey
Joy Sturtevant
Grace E. Stutzmann
Xiao Su
Shuo Su
Yunchao Su
Vivek Subbiah
Suresh Kumar Subbiah
Selvakumar Subbian
Senthil Kumar Subramanian
Rajagopal Subramanyam
Agathe Subtil
Prasanta K. Subudhi
Jan S. Suchodolski
Omar Sued
Garret Suen
Izumi Sugihara
Cassidy Rose Sugimoto
Reiko Sugiura
Hussein Suleman
Beth A. Sullivan
David J. Sullivan Jr.
Masahiko Sumitani
Meng-Xiang Sun
Genlou Sun
Kang Sun
Hao Sun
Shao-Chen Sun
Beicheng Sun
Lu-Zhe Sun
Qing-Yuan Sun
Qinghua Sun
Jianzhong Sun
Shi-Yong Sun
H. Sunny Sun
Jun Sun
Jie Sun
Durai Sundar
Raman Meenakshi Sundaram
Shian-Ying Sung
Philip Supply
Mark A. Sussman
Robert Sutherland
Susmit Suvas
Hiromu Suzuki
Akio Suzumura
Inge Marie Svane
Per Svenningsson
Anand Swaroop
Sharon Swartz
David L. Swerdlow
William M. Switzer
Ehsan U. Syed
Gergely Szakacs
Pal Bela Szecsi
Josué Sznitman
Edgardo Szyld
Arantxa Tabernero
Yvette Tache
Gilda Tachedjian
Yoshiyuki Tachibana
Michael Taffe
Elda Tagliabue
Shahrad Taheri
Heidar-Ali Tajmir-Riahi
Nobuyuki Takahashi
Nori Takei
Kazuhiro Takemoto

Patricia Talamas-Rohana
Marco Tamietto
Min-Han Tan
Ming Tan
Ivan Tancevski
Veena Taneja
Haixu Tang
Jianming Tang
Chih-Hsin Tang
Patrick Tang
Shao-Jun Tang
Yaoliang Tang
Daolin Tang
Dalin Tang
Ming Tang
Tieqiao Tang
Michael A. Tangrea
Robert L. Tanguay
Hiromu Tanimoto
Yoshiaki Taniyama
Herbert B. Tanowitz
Malú G. Tansey
Qian Tao
Yi Tao
Giovanni Targher
Kjetil Tasken
Satyanarayana Tatineni
Pedro Tauler
Nektarios Tavernarakis
William Rowland Taylor
Cormac T. Taylor
Andrew W. Taylor
Simon Taylor
Bradley Taylor
Bamidele O. Tayo
Marinus F. W. Te Pas
Ryan M. Teague
Kok Keng Tee
Muy-Teck Teh
Cristina Teixeira
Francisco J. Tejedor
Fabien Tell
Piero Andrea Temussi
Hugo ten Cate
Olle Terenius
Jefferson Terry
Dawit Tesfaye
Luca Testa
Kevin K. A. Tetteh
Farook Thameem
Douglas H. Thamm
Nandor Gabor Than
Thomas H. Thatcher
Mukund Thattai
Kednapa Thavorn
Steven M. Theg
Franziska Theilig
Volker Thiel
Benjamin Thierry
Diana M. Thomas
Jean-Léon Thomas
Bobby Thomas
Florian P. Thomas
Torsten Thomas
Richard L. Thompson
Cristiane Thompson
Fabiano L. Thompson
Benjamin Thompson
Jo Thompson Coon
Claire Thorne
Erik V. Thuesen
Praveen Thumbikat
Bin Tian
Jie Tian
Xiuchun Tian
Yin Tintut
Stefano Tiziani
David M. Tobin
Michal Toborek
Gregory P. Tochtrop
Peter Alan Todd
Matthew H. Todd
Mathias Toft
Amanda Ewart Toland
Elena Tolkacheva
Stephen Mark Tompkins

Hung Ton-That
Laszlo Tora
Karen M. Tordjman
Maria Lina Tornesello
Christopher Torrens
Eduard Torrents
Gianluca Tosini
Jorg Tost
Jonathan David Touboul
Martin J. Tovée
Kazunori Toyoda
Philip C. Trackman
Rochelle E. Tractenberg
Vladimir Trajkovic
Anna Tramontano
Ulrich S. Tran
Lam-Son Phan Tran
Gregory Tranah
Yara M. Traub-Csekö
Alexander J. Travis
Jason R. Tregellas
John S. Tregoning
François Tremblay
Jose G. Trevino
Viviana Trezza
Susheela Tridandapani
Claudio Tripodo
Ralph A. Tripp
Prabodh Kumar Trivedi
Giancarlo Troncone
Caroline L. Trotter
Ramon Trullas
Randy D. Trumbower
Robert Y. Tsai
Alexander C. Tsai
Der-Chong Tsai
Yiu Fai Tsang
Daniel J. Tschumperlin
Herman Tse
Scheffer Tseng
George Tserpes
Athanassios C. Tsikliras
Effie C. Tsilibary
Konstantinos K. Tsilidis
Lev Tsimring
Stella E. Tsirka
Sophia Tsoka
Maria Tsokos
Takafumi Tsuboi
Kenji J. Tsuchiya
Hiroyuki Tsuchiya
Moriya Tsuji
Yoshiaki Tsuji
Hirokazu Tsukaya
Jianhua Tu
Juan Tu
Tamir Tuller
Stephen J. Turner
Juha Tuukkanen
Anil K. Tyagi
Radouil Tzekov
Monica Uddin
Shahadat Uddin
Venkatachalam Udhayakumar
Matsuo Uemura
Victor M. Ugaz
Satish Ukkusuri
Ilya Ulasov
Henning Ulrich
Govindhaswamy Umapathy
Shahid Umar
James G. Umen
Derya Unutmaz
Turgay Unver
Cosimo Urgesi
Masuko Ushio-Fukai
Jamunarani Vadivelu
Krishna Prasad Vadrevu
Chandan Vaidya
Pedro Antonio Valdes-Sosa
Giovanna Valenti
Alexander Valentine
John F. Valentine
Gaetano Valenza
Nicole Valenzuela
Marti Vall

Therese van Amelsvoort
Robert J. van Beers
Willem J. H. van Berkel
Peter van Bogaert
Els J. M. van Damme
Peter van den Besselaar
Ruud van den Bos
Eva Van den Bussche
Peter G. van der Velden
Johan van der Vlag
Patrick van der Wel
Lucas van der Woude
Michiel van Elk
Martijn van Griensven
Leo A. van Grunsven
Thorbald van Hall
Gilles van Luijtelaar
Peter M. A. van Ooijen
Jim van Os
Leonard Simon van Overbeek
Mark J. van Raaij
Hedderik van Rijn
Frank J. van Rijnsoever
Maurice A. M. van Steensel
Hendrik W. van Veen
Andre van Wijnen
Jacobus P. van Wouwe
Nico W. Van Yperen
Menno C. van Zelm
Richard van Zyl-Smit
Jean-Marc Vanacker
Luca Vanella
Steven M. Varga
Deepak Vashishth
Eleni Vasilaki
Nikos Vasilakis
Cristina Vassalle
Hubert Vaudry
Lloyd Vaughan
Demetrios G. Vavvas
Rafael Vazquez-Duhalt
Christian Veauthier
Gayatri Vedantam
Rakesh N. Veedu
Gert Jan C. Veenstra
Digna Velez Edwards
Giovanni G. Vendramin
Thiyagarajan Vengatesen
Kumar Venkitanarayanan
Salvador Ventura
Julio Vera
Antonio Verdejo-García
Tom Verguts
Bruno Verhasselt
Esther Marianna Verheyen
Chandra Verma
Suresh Kumar Verma
Geerat J. Vermeij
Eric Vermetten
Sten H. Vermund
David Vernon
Akos Vertes
Mette Vestergård
David S. Vicario
Ales Vicha
Mark H. Vickers
Neeraj Vij
Jose M. G. Vilar
Mauro Villarini
Josep A. Villena
Jose Vina
Andrés Viña
Maria Cristina Vinci
Manlio Vinciguerra
Joao P. B. Viola
Amarjit Singh Virdi
Gianni Virgili
Laurent Viriot
Marie-Joelle Virolle
Virginia J. Vitzthum
Antonia Vlahou
Michael R. Volkert
Erik von Elm
Arndt von Haeseler
Ulrich von Hecker
Matthias G. von Herrath

Lorenz von Seidlein
Frank Voncken
John Vontas
Marc Vooijs
Christian R. Voolstra
Kay C. Vopel
Martin Voracek
Ina Maja Vorberg
Michiel Voskuil
Daniel E. Voth
Kent E. Vrana
Nalini Kumar Vudattu
Vladyslav Vyazovskiy
Claire Wade
Gebhard Wagener
Wolfgang Wagner
Bridget Wagner
Daniel-Christoph Wagner
Tom Waigh
Mark A. Wainberg
Ari Waisman
Hironori Waki
Piotr Walczak
Jonas Waldenström
Lourens J. Waldorp
John Wallace
Graham R. Wallace
Joseph M. Wallace
Ross Frederick Waller
Consuelo Walss-Bass
Martin Walter
Xiaoang Wan
Kai Wang
Yun Wang
Ping Wang
Yanchang Wang
Tony T. Wang
Shixia Wang
Yi Wang
Yu Wang
Zonghua Wang
De-Hua Wang
Xiaoying Wang
Qiang Wang
Xiao-Wei Wang
Qiang Wang
Kai Wang
Yuanquan Wang
Meijing Wang
Zhengqi Wang
Yingxiao Wang
Tian Wang
Hanping Wang
Yeng-Tseng Wang
James H. C. Wang
Li Wang
Tai Wang
Dong Wang
Xiujun Wang
Junwen Wang
Kunbo Wang
Rui-Wu Wang
Wei Wang
Jieru Wang
Guoying Wang
Zhengfeng Wang
Yue Wang
Fei Wang
Hongyan Wang
Yan-Ling Wang
Pei-Ning Wang
Long Wang
Ying-Jan Wang
Xiao-Dong Wang
Hua Wang
Chuan-Chao Wang
Licheng Wang
Xiaohui Wang
Shiping Wang
Qiming Jane Wang
Xiaofeng Wang
Hong Wang
Hong Wanjin
Meni Wanunu
Lawrence M. Ward
Gulam Waris

Digby F. Warner
Kishore K. Wary
Katsumi Watanabe
Koichi Watashi
Grant Waterer
Dan Weary
Edward Webb
Andrew Webber
Martin Sebastian Weber
Christopher R. Weber
Heiner Wedemeyer
Andreas Wedrich
Christian Wegener
Barbara Wegiel
Xander H. T. Wehrens
Qingyi Wei
Huafeng Wei
Zhi Wei
Leyi Wei
Chongyi Wei
Norbert Weidner
Joel H. Weiner
Aviv M. Weinstein
Tiffany L. Weir
David Weiss
Robert S. Weiss
Stefan F. T. Weiss
Alessandro Weisz
Zilong Wen
Z. Tom Wen
Rong Wen
Shi Wu Wen
Fenghua Wen
Xuchu Weng
Thomas Wennekers
James West
Sandy D. Westerheide
Matthew Whim
Ryan Whitby
Helen White-Cooper
Gerhard Wiche
Jelte M. Wicherts
Claude Wicker-Thomas
Hans-Joachim Wieden
Ryan E. Wiegand
Heinz Wiendl
Frank Wieringa
Harriet Wikman
Andrew C. Wilber
Andrea S. Wiley
Joshua F. Wiley
Katalin Andrea Wilkinson
Jennifer L. Wilkinson-Berka
Bart O. Williams
John Leicester Williams
Cecilia Williams
Mark Alexander Williams
Richard C. Willson
James Wilsdon
Richard A. Wilson
Brenda A. Wilson
Rick K. Wilson
Carol J. Wilusz
Sabine Windmann
Robert D. Winfield
Christoph Winkler
Robert A. Winn
Alan Winston
Stephan N. Witt
Paul Eckhard Witten
Alexander Wlodawer
Christiane E. Wobus
Dominik Wodarz
Collynn Woeller
Andrew Wolfe
Stefan Wölfl
Gayle E. Woloschak
Nai Sum Wong
Chun-Ming Wong
G. William Wong
Vincent Wong
Sek-Man Wong
Kwong-Kwok Wong
William Oki Wong
Patrick C. Y. Woo
Charles Jonathan Woodrow

Kerry Woolfall
R. Mark Wooten
K. Brad Wray
Christine Wrenzycki
James M. Wright
Neil T. Wright
Rongling Wu
Han-Chung Wu
Xifeng Wu
Ping-Hsun Wu
Yuntao Wu
Shu-Biao Wu
Keqiang Wu
Qing Wu
Gen Sheng Wu
Min-Hsien Wu
Wen-Chih Hank Wu
Guangyu Wu
Qiang Wu
Zhi-Ying Wu
Min Wu
Ye Wu
Yongle Wu
Gerhard Wunderlich
Anton Wutz
Doug Wylie
Glenn R. Wylie
Houhui Xia
Yinglin Xia
Feng Xia
Qingzhong Xiao
Jingfa Xiao
Gaoxi Xiao
Zhongcong Xie
Xing Xie
Shang-Ping Xie
Jingwu Xie
Yi Xing
Zheng Xing
Momiao Xiong
Eric Xu
Xiaolei Xu
Ying Xu
Shuang-Yong Xu
Wenqing Xu
Benjamin Xu
Zongli Xu
Bing Xu
Shang-Zhong Xu
Wei Xu
Changjie Xu
Yan Xu
Jun Xu
Jianhua Xu
Jun Xu
Hui-Xiong Xu
Peng Xu
Jun Xu
Ying Xu
Mingqing Xu
Junjie Xu
Jianzhen Xu
Yu Xue
Bin Xue
Rattan Singh Yadav
Nagendra Yadava
Soroku Yagihashi
W. C. Yam
Masaya Yamamoto
Yoshihiro Yamanishi
Yoshio Yamaoka
Atsuko Yamashita
Taro Yamashita
Hidenori Yamasue
Shin Yamazaki
Yong-Bin Yan
Riqiang Yan
Chunhong Yan
Burton B. Yang
Ching-Hong Yang
Yang Yang
Wan-Xi Yang
Daichang Yang
Xiao-Jun Yang
Jinn-Moon Yang
Zeng-Ming Yang

G. Yang
Xiao-Feng Yang
Chuen-Mao Yang
Fan Yang
Xiaoyan Yang
Li Yang
Guangxiao Yang
You Yang
Haibing Yang
Pingfang Yang
Isaac Yang
Zhi Min Yang
Shihui Yang
Jianjun Yang
Jian Yang
Krassimir Yankulov
Kentaro Yano
Cedric Yansouni
Dezhong Yao
Yong-Gang Yao
Pew-Thian Yap
Vinod K. Yaragudri
Andrew J. Yates
Sheng Ye
Xiaoqin Ye
Eldad Yechiam
Hui-Ling Yen
Suresh Yenugu
Laxmi Yeruva
Andrew Yeudall
Huso Yi
Ali Önder Yildirim
Arda Yildirim
Özlem Yilmaz
Sha Yin
Hang Hubert Yin
Tongming Yin
Junji Yodoi
Byung-Jun Yoon
Kyoung-Jin Yoon
Akihiko Yoshimura
Marcel Yotebieng
Roger C. Young
Kyle A. Young
Bridget Young
Reza Yousefi
Mohammed Yousfi
Xue-Jie Yu
Xiao-Fang Yu
Fu-Shin Yu
Jr-Kai Sky Yu
Jun Yu
Choongho Yu
Yang Yu
Jianhua Yu
Kefei Yu
Liqing Yu
Hong-Guo Yu
Lu-Gang Yu
Ming-Lung Yu
Sidney Yu
Y. Adam Yuan
Fan Yuan
Jiajin Yuan
Wenping Yuan
Tifei Yuan
Weihua Yue
Junming Yue
Bi-Song Yue
Chiou-Hwa Yuh
Joshua Yukich
Sung-Hwan Yun
Wing-Ho Yung
Vyacheslav Yurchenko
Andrey M. Yurkov
Katherine Yutzey
Mark D. Zabel
Olga A. Zabotina
Amir A. Zadpoor
Alessandro Zagatto
Elias T. Zambidis
Dario S. Zamboni
Lorenzo Zane
Runguo Zang
Gianluigi Zanusso
Carlos Zaragoza

Dmitri Zaykin
Cecilia Zazueta
Hajo Zeeb
Mirjam M. Zegers
Tanja Zeller
Ana Claudia Zenclussen
Fan-Gang Zeng
Weiming Zeng
Xiangxiang Zeng
Li Zeng
Alma Zernecke
Kornelius Zeth
Dimitrios Zeugolis
Xiangming Zha
Wang Zhan
Chiyu Zhang
Yang Zhang
Luwen Zhang
Y. H. Percival Zhang
Aimin Zhang
Baohong Zhang
Huiping Zhang
Guihong Zhang
Jinfa Zhang
Zhang Zhang
Jianwei Zhang
Ren Zhang
Jin-Song Zhang
Zhao Zhang
Nanyin Zhang
Jian Zhang
Yingchun Zhang
Guoping Zhang
Qijing Zhang
Zhiwu Zhang
Hong Zhang
Daoqiang Zhang
Jianmin Zhang
Le Zhang
Zi-Ke Zhang
Zhengguang Zhang
Luo Zhang
Zhe Zhang
Meijia Zhang
Tianzhen Zhang
Hong-Liang Zhang
Xiang Yang Zhang
Jing A. Zhang
Wenyi Zhang
Ge Zhang
Chi Zhang
Zhiqian Zhang
Youjun Zhang
Qinghui Zhang
Dingguo Zhang
Jianhua Zhang
Ming Zhang
Lubo Zhang
Yanbin Zhang
Yanqiao Zhang
Peng Zhang
Qinhong Zhang
Heye Zhang
Qin Zhang
Jinsong Zhang
Xiaoliang Zhang
Lanjing Zhang
Yingfeng Zhang
Zhuo Zhang
Harry Zhang
Zhongming Zhao
Shuhong Zhao
Richard Y. Zhao
You-Yang Zhao
Jianjun Zhao
Shanshan Zhao
Shuqing Zhao
Min Zhao
Feng Zhao
Chunfeng Zhao
Zhi-Ming Zheng
Jialin Charles Zheng
Song Guo Zheng
Yingfeng Zheng
Jie Zheng
Deyou Zheng

Yiwen Zheng
Yun Zheng
Degui Zhi
Boris Zhivotovsky
Guangming Zhong
Zhongjun Zhou
Renping Zhou
Changsong Zhou
Yunli Zhou
Fengfeng Zhou
Dongsheng Zhou
Huaijun Zhou
Xu-Jie Zhou
Zhi Zhou
Xiangtian Zhou
Juan Zhou
Hua Zhou
Weijun Zhou
Meixue Zhou
Shengtao Zhou
Kun Yan Zhu
Wei-Guo Zhu
Xuewei Zhu
Donghui Zhu
Liping Zhu
Jiahua Zhu
Zhiming Zhu
Asim Zia
Kirk J. Ziegler
Elke Zimmermann
Andreas Zirlik
Gernot Zissel
Michal Zmijewski
Carmine Zoccali
Michal Zochowski
Erwin G. Zoetendal
Quan Zou
Xi-Nian Zuo
Zhiyi Zuo

Advisory Board Members:

Hilda Bastian
Victoria Braithwaite
Patricia Brown
Ellen Clayton
Sarah Edwards
Adriane Fugh-Berman
Gilly Griffin
Ian Kerridge
Georgia Mason
Joy Mench
David Moher
David Morton
Jing-Bao Nie
Klaus Oberauer
Mark Prescott
Wendy Rogers
Christopher Scott
Jerome Amir Singh
Sisira Siribaddana
Jerrold Tannenbaum
Francois Venter
