# Supplementary material for: PLOS ONE 2017 Reviewer and Editorial Board Thank You
Source: PLoS One. 2018 Mar 15;13(3):e0194158. doi: 10.1371/journal.pone.0194158 (PMC5854357; doi:10.1371/journal.pone.0194158)
Supplement: S1 Guest Editor List — (PDF) [file pone.0194158.s002.pdf]

*PLOS ONE* would like to thank all those who served as Guest Academic Editors for the journal in 2017:

Christina Abdel Shaheed
Naeemah Abrahams
Mahmoud Abu-Shakra
Victor Adekanmbi
Koki Agarwal
Luca Agnifili
Mohamed Azmi Ahmad Hassali
Ahmad N. Al-Dissi
Jan-Willem Alffenaar
Veerasathpurush Allareddy
Marco Altini
Alison Anderson
Veronica Andreo
Michele Andreucci
Eric R. Anson
Armand Antommaria
Deborah Apthorp
Elisabete Aramendi
Phil Asherson
Shervin Assari
Deanna J. Attai
Lukoye Atwoli
Ahmed Awadein
Kieran Ayling
Junhong Bai
A. Bailey
Lorenzo Ball
Sarah Bangs
Anita P. Barbee-Cunningham
Yechezkel Barenholz
Galinos Barmparas
Geoffrey Barnes
Gregory S. Barsh
Marco Bassani
Maya Basu
Girish Bathla
Phil Batterham
Martin Bech
Mulugeta Belay
Benjamin Bellows
Dan Benjamini
Margaret Bentley
Janneke Berecki-Gisolf
Nancy Berman
Eta S. Berner
Graziella Berta
Godfrey Biemba
Sarah Birken
Jennifer Blum
Herbert H. Blumberg
Daniel Boduszek
Ivo G. Boneca
John M. Boone
Jeffrey Allen Borgia
A. F. Bouwman
Laurent Boyer
Anne E. M. Brabers
Nicola Bragazzi
Jonathan Braun
Christopher Brennan-Jones
Nicola Bridges
Eleonora Brivio
Clarissa Brocklehurst
Joelle M. Brown
Daniel J. Brown
Charles R. Brown
Sharon Mary Brownie
David Buchs
Shyam Sundar Budhathoki
Andreas Buechner
Mart Buekers
Marco Buenrostro-Nava
Kyle J. Burghardt
Martin Burtscher
Melanie D. Bussey
Meghan Byrne

Zhiqiang Cai
Fabio Calefato
Emily Callander
Paloma Gómez Campelo
Ozgu Can
Claudia Cappa
Gregory Carling
Danielle C. Cath
Emilio Cervantes
Douglas L. Chalker
Cliffton Chan
Larry Chang
Cyril Charles
Anthony Charles
Rima Chatterjee
Oliver Chen
Francesca Chiesi
Hyunmi Choi
Rubens Chojniak
Enrica Ciucci
Alejandra Clark
Jasmin Cloutier
Guy Cloutier
Otavio Rizzi Coelho-Filho
Pieter Coenen
Lindi-Marie Coetzee
Oded Cohen
Karen Cohen
Elizabeth Colantuoni
Rose Collard
Marissa Collins
Natalie Colson
Ignacio Conget
Ziva D. Cooper
Andrea Coppadoro
Rita Cordovil
Angelo Brandelli Costa
Kaitlin Costello
Hugh Craig
Felix Creutzig
Wenguo Cui
Peter Czermak
Marek Czosnyka
Annegret Dahlmann-Noor
Linh Dang
Margot Darragh
Anup Das
Kaberi Dasgupta
Ian C. Davidson
Judith Ann Dean
Peter F. Delaney
Paddy Dempsey
Nicolas Depauw
Jagadish M. Deshpande
Radhika Devraj
Jenna Dixon
Therese Djarv
Michelle Dohm
Deborah Donnell
Neal Doran
Dusana Dorjee
Alan W. Dow
Robert Dreibelbis
Rebecca Duerr
Ewan Dunbar
Nicholas D. Duran
Christine E. East
Karen N. Eggleston
Dana Joseph Ehret
Peter Eickholz
Robert Eikelboom
Melissa A. Elafros
Islam Y. Elgendy
Hamdy Elsayed-Awad
Bryant England
Ipek Ensari
Nülüfer Erbil
Catherine J. Evans
John Evans
Jahan Fahimi
Saeideh Fallah Fini
Almo Farina
Damien Roger Farine
Catherine Feart
Olavo Fernandes
Fernando Fernandez-LLimos
Cristine Homsi Jorge Ferreira
Albert Figueras

Guido Filler
Walter Finsinger
Horacio Firmino
Laura Folgori
Patrice Forget
Rita Formisano
Israel Franco
Jessica Myers Franklin
Mirella Fraquelli
Jörg Frommer
Ingo Frommholz
Todsaporn Fuangrod
Sara Fuentes Perez
Sadaaki Fukui
Steve Huntz Fung
Isaac Chun-Hai Fung
Belinda J. Gabbe
Maurizio Galderisi
Sarah Gammage
Christos Ganos
Jonathan Garcia
Linda J. Garcia
Becky L. Genberg
Bishwajit Ghose
Sara Gianella
Clare Gilbert
Emma Giles
José Antonio Gil-Montoya
Antonio Z. Gimeno-Garcia
Michael R. Gionfriddo
Christophe Giraud-Carrier
Mary Glover-Amengor
Timo Gnambs
Brian Godman
Sarah Gollust
Nihan Gonulol
Russell Goodall
Michael L. Goodman
Chaitra Gopalappa
Dipti Govil
Stephen Michael Graham
Yannick Griep
Kristi Griffiths
Andrea Gruneir
Andrzej Grzybowski
Sylvia Fátima Santos Guerra
Ricardo Queiroz Gurgel
Helena Hachul
David Hackney
Andrew Hales
Juho Hamari
Jeff Hamm
Lin Han
Erin Haramoto
Jane Harries
Paul Richard Harrigan
Joanna Hart
Erik K. Hartmann
Baktiar Hasan
Dana Hashim
Robert K. Heaton
M. Heijnen
Oskari Heikinheimo
Otto Helve
Yves Henchoz
Devon Hensel
Robert Hepach
Luiz U. Hepp
Mehdi Heydari
Noora Hirvonen
Renee Hoch
Robert S. Hogg
Kristine Hopkins
Susan Horton
Fabian Huettig
Maciej Huk
David Hutton
Onur Ismi
Salomon Israel
Khawar Jabran
Robert J. Jackson
Jeremy Michael Jacobs
Sudarshan Jadcherla
Grazyna Jasienska
Gursimer Jeet
Godwin Terver Jombo
Charles Jonassaint
Rafal Jończyk

Steven L. Jones
Holger Joswig
Slavisa Jovanovic
Susanne Kaae
Rujvi Kamat
Tomonori Kanda
Se Woong Kang
Lillian Kao
Katherine J. Karriker-Jaffe
Vilvapathy Senguttuvan Karthikeyan
Diego Kaski
Andrea Kassner
Shane A. Kavanagh
Benjamin Christopher Kearns
Ashish K. Khanna
Khaled Khatab
Dohyeong Kim
Maria Kleinstaeuber
Rüya-Daniela Kocalevent
Philipp D. Koellinger
Patrick Kolsteren
Helen Kopnina
Konrad P. Kording
Mirjam Körner
Samet Kose
Gerald Kost
Alexander Kretschmer
Candyce Kroenke
Nithin Kumar
Yong-Hong Kuo
Ratilal Lalloo
Paul A. Lapchak
Kenzie Latham-Mintus
Simona Lattanzi
Kim Lavoie
Lambros Lazuras
Nathan K. LeBrasseur
Sun Y. Lee
Joy Li-Yueh Lee
Wei-Ning Lee
R. K. Lee
Arleen A. Leibowitz
Andrew Lepp
Peter R. Lewis
Xiaojuan Li
Zhan Li
Sai Liang
Sian Yik Lim
Christopher D. Lippitt
Marco Livesu
Steven LoBello
David S. Logerstedt
Marisol López-López
Benjamin Lopman
Cembalo Luigi
Geping Luo
John MacDonald
Adriano Magli
Surakameth Mahasirimongkol
Oluwole Daniel Makinde
Mohammad Maktoomi
Irene Cristina Mammarella
Sundhiya Mandalia
Anatole Manzi
Machteld Marcelis
Hanna Marno
Denis Martin
Tamara Martin Gimenez
Ana Marusic
Kei Masani
Natalie Matosin
Hiroshi Matsuda
Claudia Trindade Mattos
Brandon R. McFadden
Corey McGee
Bano Mehdi
Rodolfo Mendoza-Denton
Annette M. Mercer
Susana Merino
Markus Metsälä
Andreas Mierau
Atsushi Miyamoto
Mollie A. Monnig
Jagidesa Moodley
Ginny Moore
Orna Mor
Aythami Morales

Dimitrios K. Moutopoulos
Cheryl A. Moyer
Ute Mueller
Jannes Muenchow
Taulant Muka
Juliane Müller
Samantha Munroe
Gregg R. Murray
Damian Murray
Beate Muschalla
Geofrey Musinguzi
Masanori Nagamine
Jason M. Nagata
Aritro Nath
Gabriel Natividad
Subas Neupane
Aaron Jon Newman
Yann Nguyen
Makandwe Nyirenda
Alberto Ocaña
Atte Oksanen
Malgorzata Olejnik
Tom Olino
Mehmet Yekta Oncel
Temitope Olabisi Onuminya
Andrea D. Orsey
Lukas Oudejans
Pavel V. Ovseiko
Amir H. Pakpour
Georgia Panayiotou
Filomena Papa
Leon-Etienne Parent
Kourosh Parham
Mijung Park
Sonak Pastakia
Shane Patman
Gabriela Paz-Bailey
Vishnu S. Pendyala
Kalyana Chakravarthy
Pentapati
Beata Peplonska
Eduardo Roque Perna
Borut Peterlin
Sean D. Peterson
Benjamin A. Philip
L. Alison Phillips
Barbora Piknova
Manuela Pinzari
Dimitris N. Politis
Margareth Portela
James M. Pringle
Ari Probandari
Janey Prodoehl
Amy Prunuske
Andrew D. Pucker
Iratxe Puebla
Shuby Puthussery
Xinying Qiu
Jenna Quinto
Peter Rabinowitz
Jeffrey J. Rade
Gurch Randhawa
Sarbin Ranjitkar
Julie Redfern
Matthias Reeh
Jurgen Rehm
Natalia Reich-Stiebert
Roberto E. Reis
Delphine Renard
Soo Rhee
Debra Rickwood
Ian R. H. Rockett
Rachel F. Rodgers
Chris Rogers
Leàn Rolfes
Ola Rolfson
Jan-Erik Romar
Daniel Romer
Danya Rosen
David L. Rosen
Meagen Rosenthal
Peter F. W. M. Rosier
Michael W. Ross
D. G. Rossiter
Rebecca C. Rossom
John Rovers
Mollie A. Ruben
Marc D. Ryser

Simona Sacco
Ahmed S. Safwat
Salah A. M. Said
Olivier Saint-Lary
Alfonso Saiz-Lopez
Abraham Salinas-Miranda
Tim Salomons
Zainab Samaan
Priyabrata Santra
Rafael Sarkis-Onofre
Mark Savill
Marion Saville
Andreas Schadschneider
Paula Schaiquevich
Celena Scheede-Bergdahl
Laura Scherer
John Schieffelin
Thomas Schlösser
Rebecca Schnall
Peter J. Schulz
Gundula Schulze-Tanzil
Hannes Schwandt
Holly Seale
Dorothy D. Sears
Ary Serpa Neto
Daniel Serrani Azcurra
Juan Manuel Serrano-Rodríguez
Tayyab Ikram Shah
J. A. Shaw
Kerry Sherman
Catherine Sherrington
Grigori Sidorov
Debjani Sihi
Gaurav Sikri
Claudionor R. Silva
Patrícia P. Silveira
Anna Simonin
David A. Sinclair
Dheer Singh
Herman Sintim
Fuschia Sirois
Shankar Siva
Justin Smith
Roland A. Snijder
Majid Soleimani-Damaneh
Mark W. Sonderup
Allison P. Squires
Rajagopalan Srinivasan
Nicola Stead
Jennifer Steele
Michael B. Steinborn
Arnaud Steyaert
Heidi Stöckl
Manuela Straneo
Eva Elisabeth Stüeken
John A. Sturgeon
Bala Subramaniam
Hao Sun
Amitabh Bipin Suthar
Josefin Sveen
Ka-Wai Tam
Ernest Tambo
Shigeho Tanaka
Li Tang
Jakub Taradaj
Donald Tashkin
Peter M. ten Klooster
Bo Thamdrup
Mieke Beth Thomeer
Caroline Thompson
Peter Thorne
Dirk Tischler
Nick Todd
Andrew Alexander Tokmakoff
Massimiliano Toscano
Nicolas Tsapis
Laurie Twells
Costin Daniel Untaroiu
Rosa Maria Urbanos Garrido
Sergio A. Useche
Mojtaba Vaismoradi
Emily Vala-Haynes
Elvira Valera
Pablo Vallejo-Medina
Margriet van Baar
Erica van den Akker
Just Alexander van der Linde

Joseph Van Sickels
Chris Van Weel
André A. J. Van Zundert
John A. Vargo
Viktor Vegh
Emma Veitch
Pablo R. Vellando
Robert A. Veselis
Mario Vetrano
Nora Silvana Vigliecca
Simona Viglio
Daniel Vigo
Neil J. Vincent
Alexander Vlantis
Dina Vojinovic
Umberto Volpe
Steve Vucic
Antonia L. Wadley
Bradley H. Wagenaar
Jo Waller
Yuanyuan Wang
Xin Shelley Wang
Sarah Warkentin
Jane Warland
Sandra Webber
S. Samuel Weigt
Heide Weishaar
Benedict Weobong
Alexander J. Werth
Stephanie Wilkie
Jenny M. Wilkinson
Paige L. Williams
Fernando A. Wilson
Thomas Wingfield
Melissa H. Withers
James Wolffsohn
Davene Wright
Chen-Chi Wu
Guanghua Xiao
Hongwei Xu
Xiaohui Xu
Lynn Yee
Sanichiro Yoshida
Xiping Yu
Francesco Zaccardi
Soroush Zaghi
Mussaret Bano Zaidi
Daniel Capella Zanotta
Feng Zhang
Roseanna N. Zia
J. Jaime Zúñiga-Vega
