# Supplementary material for: PLOS ONE 2017 Reviewer and Editorial Board Thank You
Source: PLoS One. 2018 Mar 15;13(3):e0194158. doi: 10.1371/journal.pone.0194158 (PMC5854357; doi:10.1371/journal.pone.0194158)
Supplement: S1 Reviewer List — Alphabetical list of 2017 PLOS ONE peer reviewers (A-D). (PDF) [file pone.0194158.s003.pdf]

PLOS ONE would like to thank all those who reviewed on behalf of the journal in 2017:

Jerome A.
Farhan Aadil
Eivind Aadland
Jens Aagaard-Hansen
Anna-Mari Aalto
Juha Aalto
Lauri Aaltonen
Mikko Aaltonen
Maria Aamelfot
Aase Aamland
Zach Aanderud
Carolien Aantjes
Hilde Aardema
Andre Aarnink
Roy Aaron
Shawn Aaron
Ulrika Aasa
Jan Aasly
Tor Aasmundstad
Mervi Aavikko
Hashir Aazh
Aminuddin Ab Ghani
Nurul Hidayah Ab Rahman
Mohd Nadhir Ab Wahab
Andrew Abaasa
Elena Abad
Catalina Abad Rabat
Eduardo Abade
Arkan Abadi
Tabare Abadie
Diana Abad-Tortosa
Tatjana Abaffy
Amanuel Abajobir
Georgios Abakoumkin
Evgeny Abakumov
Jenny Abanto
Samuel Abariga
Aniekan Abasiattai
Davide Abate
John Abatzoglou
Theodore Abatzopoulos
Serebe Abay
Solomon Abay
Mohammed Abba
Yusuf Abba
Alexander Abbas
Faisal Abbas
James Abbas
Khushnood Abbas
Naeem Abbas
Syed Abbas
Amir Abbasi
Jessica Abbate
Giovanni Abbate-Daga
Wissem Abbes
Kirsten Abbot-Smith
Carla Abbott
Catherine Abbott
Christopher Abbott
David H. Abbott
Felicity Abbott
Jeffrey Abbott
Jessica Abbott
Sabra Abbott
Giovanni Abbruzzese
Sophie Abby
Elsayed Abd Allah
Ekram Abd El Wahab
Ahmed Abd El Wahed
Ekram Abd El-Wahab
Harris Shah Abd Hamid
Ana Abdala
Mahmoud Abdalah
Mohammad Abd-Alhaseeb
Fábio Abdalla
Mahmoud Abdalla
Abdelmooty Abd-Alla
Hanaa Reyad Abdallah

Ayesha Abdeen
Rania Abdel Hay
Ahmed Abdel Moneim
Bahaa-Eldin Abdel Rahim
Almoataz Youssef Abdelaziz
Heba Abdel-Aziz
Mohamed Abdelbary
Rania Abdel-Basset Sanad
Mohamed Abdel-Daim
Hesham Abdeldayem
Rewaida Abdel-Gaber
Mohamed Abdelhady
Hany Abdel-Hafiz
Sonia Abdelhak
Ali Abdelhameed
Sherif Abdelhamid
Maha Abdelhaq
Salim Abdelilah-Seyfried
Ossama Abdelkhalik
Mohamed Abdellah
Noura Abdellah
Ahmed Abdel-Latif
Mohamed Abdelmegeed
A. M. Abdel-Mohsen
Mohamad Abdel-Mohsen
Patrícia Verardi Abdelnur
Anas AbdelRahman
Mohamed AbdelRahman
Mostafa Abdelrahman
Sherif Abdel-Rahman
Ahmed Abdel-Razik
Nourtan Abdeltawab
Ayana Abdeta
Hamidreza Abdi
Kianoush Abdi
Edimansyah Abdin
Mohammad Abdolahad
Asghar Abdoli
Hamid Abdollahi
Rostam Abdollahi-Arpanahi
Parviz Abdolmaleki
Kartini Abdul Jabar
Muhammad Khalis Abdul Karim
Nazia Abdul Majid
Marlinda Abdul Malek
Ali Abdul Munaim
Nusaibah Abdul Rahim
Fatimah Abdul Razak
Asharaf Abdul Salam
Siti Fairus Abdul Sani
Ali Abdul Sater
Mohamed Faizal Abdul-Careem
Shafi'i Muhammad Abdulhamid
Mehmet Abdulhayoglu
Regina Abdulkader
Ahmad Faizal Abdull Razis
Ameer Abdulla
Adina Abdullah
Afnizanfaizal Abdullah
Asnawi Abdullah
Kamarul Amin Abdullah
Laila Abdullah
Maha Abdullah
Noorhidawati Abdullah
Aous Abdulmajeed
Hunar Abdulrahman
Mahera Abdulrahman
Anmar Abdul-Rahman
Gaddafi Abdul-Salaam
Ali Abdul-Sater
Mohammed Abdulwahhab Abdulwahid
Jaafar Abduo
I. Y. Abdurakhmonov
Ibrokhim Abdurakhmonov
Ishmail Abdus-Saboor
Fumiyoshi Abe
Kohtaro Abe
Koji Abe
Sarah Abe
Shinji Abe
Takashi Abe
Christopher Abeare
Fekadu Abebe
Kaleab Abebe
Tilahun Abebe
Ana Abecasis
Leticia Abecia
Tjakko Abee

Filip Van Abeele
Mathias Abegg
Christian Abel
Thomas Abel
Kristopher Abell
Pedro Abellán
Bart Aben
Ludovico Abenavoli
Michael Abend
Daniel Aberdam
Christoffer Åberg
Avraham Abergel
Judith Aberle
Vinay Abhyankar
Charbel Abi Khalil
Akebe Luther King Abia
Md. Ruhul Abid
Mohammad Abid
Shad Abid
Gbadegesin Abidoye
Marianne Abi-fadel
Livny Abigail
Niveen AbiGhannam
Giuseppina Abignano
Niels Abildgaard
Adewuya Abiodun Olugbenga
Olajide Abiola
Ajibola Abioye
Ronit Abir
Ramin Abiri
Carolyn Abitbol
Jean Abitbol
Marc Abitbol
Samuel Abiven
Alfonso Abizaid
Adib Abla
Drew Abney
Da Costa Aboagye
Vicente Aboites
Nafati Aboserwal
Hussein Abou Abbass
Hadi Abou el Hassan
Abdelrahman Abou Rawash
Ahmed Abouelnour
Jad AbouKhalil
Hassan Aboul-Enein
Fatma Aboulnasr
Anna Abou-Raya
Ahmed Abouseif
Sameh Abouzid
Abdeljalil Abouzouggar
Yosef G. Abraha
Allistair Abraham
Analìa Abraham
Eleni Abraham
Georgi Abraham
Ivo Abraham
Naeemah Abrahams
Ana Abraido-Lanza
Victoria Abraira
Francesca Abramo
Steven Abramowitch
Matthew Abramowitz
Lise Abrams
Ronald W. Abrams
Jeremy Abramson
Natalia Abramson
Steven Abramson
Jacqueline Abranches
Ana Abrantes
Florence Abravanel
Tina Abrefa-Gyan
Isabel A. Abreu
Patrícia Antonia Estima Abreu
F. Alberto Abreu-Grobois
Ana Lucia Abreu-Silva
Neha Abrol
Ravinder Abrol
Saeid Abroun
Ouza Abrous-Belbachir
Rana Abu Farha
Ibrahim M. Abu Reidah
Muna Abu Sin
Khalid Abualsaud
Muhammad Abubakar
Mustapha Abubakar
Usman Abubakar
Aierken Abudu

Sara Abuelmaali
Angel Abuelo
Salem Abukres
Lisa Abuogi
Sachiyo Aburatani
Rania Abutarboush
Timothy Abuya
Ahmed Abuzaid
Vanda Acácio
Ali Acar
Cengiz Acar
Taylan Acar
Alessio Accardi
Giulia Accardi
Luisa Accardi
Rosita Accardi
Ettore Accolla
Manfred Accrombessi
Pablo Acebal
Alberto Acedo
Luis Acedo
Arianna Aceti
Paola Aceto
Flor Acevedo
Miguel Acevedo
Pelayo Acevedo
Satya Achanta
Arnab Acharya
Arun Acharya
Kshitish Acharya
Om Prakash Acharya
Pawan Acharya
Vishal Acharya
David Achat
Maria Achatz
Guillaume Achaz
Emmanuel Acheampong
Franklin Acheampong
Amit Achhra
Sharon Achilles
Hammadi Achour
Sami Achour
Laura Acion
Lia Elena Aciu
Jason Acker
Rochelle Ackerley
Rakefet Ackerman
William Ackerman IV
Mathias Ackermann
Paul Ackermann
Gareth Ackland
Steven Ackleson
Sarah Ackley
Karen Ackroff
Gerardo Acosta
Juan Acosta
Sandra Acosta
Dionisio Acosta Mena
Hector Gabriel Acosta Mesa
Eva Acosta Rodriguez
D. Acosta-Avalos
Luis Actis
Guglielmo Actis Dato
Jeffrey Actor
Claudia Acuna-Castillo
Darío Acuña-Castroviejo
Oreste Acuto
Stefan Aczel
Hisashi Adachi
Masao Adachi
Megumi Adachi
Minaco Adachi
Taiki Adachi
Takeshi Adachi
Satomi Adachi-Akahane
Timothy Adair
Alejandro Adam
Barry Adam
Greer Adam
Ishag Adam
Mary Adam
Pascal Adam
Rosalyn Adam
Vojtech Adam
Manolis Adamakis
Agata Adamczyk
Amy Adamczyk
A. Adameova

Christoph Adami
Alessio Adamiano
David Adamkin
Diane Adamo
Christina Adams
Colin Adams
Crystal Adams
David Adams
Dean Adams
Douglas Adams
Jonathan Adams
Kenneth Adams
Lisa Adams
Matthew Adams
Rick Adams
Robert Adams
Thomas Adams
Tim Adams
Vanessa Adams
Zeno Adams
Lucile Adams-Campbell
Chris Adamson
Ana Adan
Metadel Adane
Vyacheslav Adarichev
Ian Adcock
Balasubrahmanyam Addepalli
Merideth Addicott
Maria Filippa Addis
Piero Addis
Anthony Addison
Oluwatomisin Adebanjo
Adekunle Adebowale
Adebola Adedimeji
Eric Adee
Dennis Adeegbe
Peter Adegbola
Coen Adema
Abiodun Adeoye
Monsuru Adepeju
Yusuf Adeshina
Adetola Adesida
Olatunji Adetokunboh
Babatunde Adewale
Adewole Adewumi
Emmanuel Adewuyi
Rasheed Adeyemi
Adebolajo Adeyemo
Oyedele Adeyi
John Adgate
Anupam Adhikari
Atin Adhikari
Badri Adhikari
Bipin Adhikari
J. Adhikari
Lasith Adhikari
Rajan Adhikari
Rajendra Adhikari
Sondipon Adhikari
Arun Adhikary
Till Adhikary
Sankar Adhya
Tapan Adhya
Sam Adie
Satish Adiga
Juma Adinan
Bryon Adinoff
Luigi Elio Adinolfi
Noam Adir
David Adjei
Tomabu Adjobimey
Becky Adkins
Deanna Adkins
Elizabeth Adkins-Regan
Mehrdad Adl
Ben Adlam
Andy Adler
Jeremy Adler
John Adler
Peter Adler
Sophie Adler
Steffen Adler
Norman Adlung
Zoe Admas
Ashfaq Adnan
Sylvain Adnet
Philip Kofi Adom
Philip Adongo

Ralph Adophs
Teresa Adragao
Eric Adriaenssens
Troncoso-Ponce Adrian
Rodríguez Adriana
Madinah Adrus
Nathalie Aelterman
Vincent Aengevaeren
Philipp Aerni
Joachim Aerts
Johan Aerts
Daniel Aeschlimann
Antreas Afantitis
Taiwo Afe
Solange Afeche
Nicholas Affrunti
A. Rahim Afidah
Bosede Afolabi
Adeleye Afolayan
Eve Afonso
Kelvin Afrashtehfar
Nikolaos Afratis
Evans Afriyie-Gyawu
Baris Afsar
Golshan Afshari
Babak Afsharipour
Rebecca Aft
Kiran Aftab
Taimoor Afzal
Patricia Agaba
Nera Agabiti
Victor Agadjanian
R. P. Agaev
Dritan Agalliu
Suneth Agampodi
Mehmet Agaoglu
Giuseppe Agapito
Tundi Agardy
Ameeta Agarwal
Anuj Agarwal
Anurag Agarwal
Divyansh Agarwal
Gunjan Agarwal
Koki Agarwal
Kshitij Agarwal
Mayank M. Agarwal
Pinky Agarwal
Rajiv Agarwal
Renu Agarwal
Shubham Agarwal
Sunita Agarwal
Suresh Agarwal
Martin-Paul Agbaga
Stephen Agboola
Sune Agersnap
Amita Aggarwal
Arun Aggarwal
Karan Aggarwal
Artee Aggrawal
Samuel Aggrey
Ali Aghdassi
Sonia Aghighi
Rokhsareh Aghili
Kingsley Agho
Avelin Aghokeng
Hermann Agis
Paul Agius
Robert Agler
Elena Agliari
Nancy Agmon-Levin
Ingi Agnarsson
Luca Agnelli
Tove Agner
Giulio Agnetti
Philippa Agnew
Luca Agnifili
Kanishk Agnihotri
Paolo Agnolucci
Nneamaka B. Agochukwu
Antonella Agodi
Simon Agolory
Vatche Agopian
Rafiou Agoro
Valter Agosti
Aubert Agostini
Marco Agostini
Silvia Agostini
Alexander Agoulnik

Laurence Agouridas
Alok Agrawal
Anurag Agrawal
Arpana Agrawal
Devendra Agrawal
Ruchi Agrawal
Suraksha Agrawal
Ruben Agrelo
Paul Agris
Maria Teresa Aguado
Francisco Aguayo
Jaime Aguayo
Antonio Agudo
Maria Agüera
Zaida Agüera
Anna Caroline Aguiar
Francisca Aguiar
Eliot Aguilar
Oscar Aguilar
Patricia Aguilar
Marta Aguilar Perez
Hector Aguilar-Carreno
Hugo Aguilar-Díaz
Nancy Edith Aguilar-Olivos
Raul Aguilar-Roblero
Carlos Aguilar-Trigueros
Felipe Aguilera
Stacy Aguilera
Sergio Aguilera Albesa
E. Aguilera-Tejero
Nacho Aguilo
Armando Aguirre
Jesus Aguirre
Jose Aguirre
Windsor Aguirre
A. I. Aguirre-Salado
Tolulope Agunbiade
Agnes Agunos
Trevor Agus
Tetsuro Agusa
Juan Pablo Agusil
J. Agusti
Rina Agustina
Montserrat Agut
Thais Agut
Lemese Ah Tow Edries
Abdul Ahad
Aftab Ahamed
Ghasem Ahangari
Seyedhossein Aharinejad
Efrat Aharonov-Majar
Dragana Ahel
Ivan Ahel
Justice Aheto
Justice Moses K. Aheto
Laurent Ahiablame
Per Ahlberg
Thomas Ahlering
Niklas Ahlgren
Ulf Ahlgren
Mark Ahlman
Arti Ahluwalia
Aasim Ahmad
Abu Ahmad
Ali Ahmad
Anis Ahmad
Aryati Ahmad
Basir Ahmad
Iftikhar Ahmad
Iman Ahmad
Iqbal Ahmad
Jamal Ahmad
M. Ahmad
Mahmood Ahmad
Muhammad Ahmad
Musheer Ahmad
Nafees Ahmad
Nazni Ahmad
Nihal Ahmad
Niyaz Ahmad
Rafi Ahmad
Sabbir Ahmad
Saheem Ahmad
Shaad Ahmad
Shandar Ahmad
Syed Ahmad
Mohamed Ahmad Hassali
Siti Khuzaimah Ahmad Sharoni

Gabby Ahmadia
Ali Ahmadian
Leila Ahmadian
Arash Ahmadivand
F. Ahmadizar
Mostafa Ahmadzadeh
Anwar Ahmed
Charles Ahmed
Chulbul Ahmed
Hamdia M. Ahmed
Hussein Abdullah Ahmed Ahmed
Isam A. Ahmed
Jamil Ahmed
Laving Ahmed
Marwa Fawzy El Metwaly Ahmed
Monjur Ahmed
Nisar Ahmed
S. Ansar Ahmed
S. Sohail Ahmed
Saad Ahmed
Sayem Ahmed
Syed Masud Ahmed
Syud Ahmed
Yashi Ahmed
Zaghloul Ahmed
Gorkan Ahmetoglu
Narges Ahmidi
Hee-Ran Ahn
Hyeong Sik Ahn
Jin-Hyun Ahn
Ki Ok Ahn
Richard Ahn
Seong Joon Ahn
Sun Joo (Grace) Ahn
Sungwoo Ahn
Tae-Hyuk Ahn
Woo-Young Ahn
Yong-Yeol Ahn
Youngmee Ahn
Collins Ahorlu
Kai Ahrendt
Robert Ahrens
Elizabeth Åhsberg
Mehmet Ahsen
Banu Ahtam
Nitin Ahuja
Punkaj Ahuja
Shama Ahuja
Guanhua Ai
Junmei Ai
Qinghui Ai
Tao Ai
Xun Ai
Setsuya Aiba
Stefano Aicardi
Mohammed Aider
Frederik Aidt
Allison Aiello
Ernesto Aiello
Marco Aiello
Beatrice Aighewi
Bertrand Aigle
Michael Aigner
Ken-Ichi Aihara
Elena Aikawa
Christopher Aiken
Glen Aiken
Judd Aiken
Robert Aiken
Laurie Ailles
Vishukumar Aimanianda
Flavio Aimbire
Alberto Aimo
Jason Aimone
David Ainley
Jose Ainsa
Elizabeth Ainsbury
Ben Ainsworth
Elizabeth Ainsworth
Hannah Ainsworth
K. E. Airaksinen
Katherine Aird
Marta Aires-de-Sousa
Valentina Airi
Cristina Airoldi
H. A. Aisa
Daniela Aisenberg
Lamia Ait-Ali

Srinivasulu Aitipamula
Elisabeth Aitken
Jonathan Aitken
Karen Aitken
Marrtín-Pintado Aitor
Slimane Ait-Si-Ali
Yoshifusa Aizawa
Oluwaremilekun Ajakaye
Hoori Ajami
Mokrish Ajat
Itopa Ajayi
Oluseyi O. Ajayi
Marina Ajduković
Marco Ajelli
Matthew Ajemian
Budi Aji
Olusola Ajilore
Masahiko Ajiro
Maria Antonietta Ajmone-Cat
Kolapo Ajuwon
Metin Ak
Hiroyuki Akagawa
Tadamichi Akagi
Tomohiko Akahoshi
Masami Akai
Oluwatoyin Akande
Masaharu Akao
Yukio Akasaki
Alpaslan Akay
Chihiro Akazawa
Hiroshi Akazawa
James Akazili
Malahat Akbarfahimi
Mustafa Akbulut
H. Resit Akcakaya
Aliye Akcali
Guray Akdogan
Örjan Åkerborg
Pete Akers
R. Akers
Walter Akers
Mark Akeson
Agneta Åkesson
S. M. Javid Mahmoudzadeh Akherat
Andrei Akhmetzhanov
Abdullah Akhtar
Muhammad Akhtar
Riaz Akhtar
Yasmin Akhtar
Baharak Akhtardanesh
Sayeba Akhter
Yusuf Akhter
Adnan Akhunzada
Toshihiko Aki
Pierre Akilimali
Cem Akin
Heather Akin
Modupe Akinola
Jocelyn Akins
Robert Akins
Akanni Akinyemi
Sadanori Akita
Hideo Akiyama
Masashi Akiyama
Yoshimitsu Akiyama
Takashi Akiyoshi
Tatsuya Akizuki
Taner Akkin
Ramesh Akkina
Junya Ako
Blessing Akombi
Patrick Akpaka
Zohaib Akram
Michel Akselrod
Alexander Aksenov
Murat Aksoy
Erdem Aktas
Ian Akyildiz
Cihangir Akyol
Levent Akyürek
Amr Al Abed
Tarek Al Baghal
Mohammed Al Balwi
Tareq Al Saadi
Fatima Al Sayah
Thamer Al Slamah
Olufunke Alaba
Mirit Aladjem

Kaat Alaerts
Sumithra Alagarsamy
Yagiz Alagoz
Alexandre Alahi
Ghiath Alahmad
Abraham Al-Ahmad
Zahra Alahmed
Fahad S. Al-Ajlan
Amos Alakonya
Shereen Al-Ali
A. Alam
A. H. M. Alam
Anwar Alam
Asim Alam
Hasan Alam
Khurshid Alam
M. Alam
M. Jahangir Alam
M. M. Mahbub Alam
Mohammad Alam
Nazmul Alam
Syed Imteyaz Alam
Uazman Alam
Suhail Al-Amad
Luis Alameda
Samah Alamery
Hasanat Alamgir
Mohammed Alamgir
Andrea Alamia
Mahdi Alamili
Jarmo Alander
Sarah Alansari
Raid Alany
Merve Alanyali
Larbi Alaoui
Mohana Alapati
Francisco Alarcon
Juan Marcos Alarcon
Pablo Alarcon
Vernadeth Alarcon
Francisco Javier Alarcón López
Francisco Alarcon-Chaidez
Enrique Alarcón-Gutiérrez
Pascale Alard
Maha Al-Asmakh
Kyriaki Alatzoglou
Juan Jose Alava
Afsaneh Alavi
Nazanin Alavi
Beatrice Alba
Daoud Al-Badriyeh
Rafael Albaladejo
Jasim Albandar
Chris Albanese
Maria Albanese
Diego Albani
Domenico Albano
Gian Luigi Albano
Pedro Albarran
Daniel Albaugh
Matthew Albaugh
Ali H. Al-Bayatti
Marina Albentosa
Birgit Alber
Ane Alberdi
Antton Alberdi
Lilia Alberghina
Lavinia Alberi
Lorenzo Alberio
Christoph Albers
Paul Albers
James Albert
Joerg Albert
Markus Albert
Stefan Albert
Steven Albert
Juan Alberti
Allepuz Alberto
Filipe Alberto
Cristine Alberton
Bart Alberts
Lindsey Albertson
Timothy E. Albertson
Roger Albin
Manuel Leonard Albis
Jacqueline Alblas
Alessandro Alboresi
Daniel Albrecht

Harald H. Albrecht
Kathryn Albrecht
Moritz Albrecht
Philipp Albrecht
Sandra Albrecht
Cheryl Albright
E. Albuquerque
Maicon Albuquerque
Kevin Alby
Maria Alcaide
Sarah Alcala
Roy Alcalay
Antonio Alcami
Luiz Alcantara
Jordi Alcaraz
Luis David Alcaraz
Stefano Alcaro
Gabriel Alcoba
Joe Alcock
Lisa Alcock
Martin Alcordo
John Alcorn
José Alda
Anthony Aldave
A. P. Aldenkamp
Thomas Alderliesten
Harold Alderman
Robyn Alders
Hl Alderson
Rita Aldini
Linda Aldoory
Alison Aldrich
Stephen Aldrich
Kristina Aldridge
Silvio Aldrovandi
Hakan Aldskogius
Victor Alegana
Ana Luisa Aleixo
Carmen Alejo-Plata
Asma'A Al-Ekrish
Krasimira Aleksandrova
A. V. Alekseenko
Ivan Alekseichuk
Alexander Alekseyenko
Ivana Aleksić
Julie Aleman
Tomas Aleman
Heliodoro Alemán Mateo
Sergio Alemano
Silvia Alemany
Mussie Alemayehu
Taddese Alemu
Talia Alenabi
Gitana Alencikiene
Kefyalew Addis Alene
Olatunji Alese
Andrey Aleshkin
Marianna Alesi
Laura Alessandretti
Cristiano Alessandri
Guido Alessandri
Marco Alessandrini
Edward Alessi
Jessica Alessi
Alberto Aleta
John Aletta
Linda Alex
Nickolaos Alexacis
Vasileia-Ismini Alexaki
Alana Alexander
Charlotte Alexander
Craig Alexander
Derek Alexander
Gerianne Alexander
Janet Alexander
Jeffrey Alexander
Karen Alexander
Nathan Alexander
Neal Alexander
R. Todd Alexander
Rebecca Alexander
Suceena Alexander
Thomas Alexander
Timothy Alexander
Soren Alexandersen
Rojas-Molina Alexandra
Frederic Alexandre
Marie-Cecile Alexandre-Gouabau

Thomas Alexandridis
Antigoni Alexandrou
Evan Alexandrou
Boian Alexandrov
Dmitry Alexeev
Mikhail Alexeyev
Oleg Alexeyev
Christoph Alexiou
Athina Alexopoulou
Emil Alexov
M. Alfa
Massimo Alfano
Carlos Alfaro
Manuel Alfaro
Raquel Alfaro-Sánchez
Denis Alferez
Santiago Alferez
Jan-Willem Alffenaar
Sofa Alfian
Carlo Alfieri
Carolina Alfieri
Fabio Alfieri
Lorenzo Alfieri
Roberta Alfieri
Zarko Alfirevic
José Alfonso
João Alfredo de Moraes
Vincent Algalarrondo
Mabel Algeciras
Holly Algood
Obada Alhabashneh
Sahal Al-Hajoj
Basil Alhassan
Robert Alhassan
Mahmoud Al-Hindi
Hamed Alhoori
Irfan Ali
Jannath Ali
Joseph Ali
Liaqat Ali
Md. Asraf Ali
Mohamed Ali
Mohammad Ali
Mohammed Eunus Ali
Muhammad Ali
Nabeela Ali
Nahid Ali
Naushad Ali
Rashid Ali
Samina Ali
Shafat Ali
Shawkat Ali
Ajmal Alì
Ricardo Alia
Saeid Aliabadi
Hadi Aliakbarpour
Cristiano Alicino
Jesus Alierta
Gjumrakch Aliev
Marco Alifano
Konstantinos Aliferis
Nabil-Fareed Alikhan
Mona Alikhani
Warren Alilain
Jorge Alio
Babak Alipanahi
Mohammadhossein Alipour
Reid Alisch
Eva Alisic
Noorjahan Banu Alitheen
Anne Alix
Jalal A. Aliyev
Gambo Aliyu
Mojtaba Alizadeh
Morad Alizadeh
Patrick Alizai
Hareth Al-Janabi
Salem Aljareh
Taiseer Aljazzar
Mohammed Aljearah
Loai Aljerf
A. Aljizeeri
Karim Alkadhi
Ron Alkalay
Sezai Alkan
Uri Alkan
Wynand Alkema
Huda Al-Khalaf

Moh Alkhamis
Mohammad Alkhamis
Khaled Alkharsah
Alyaa Rajih Mahmood Al-Khateeb
Mohamad Alkhouli
Fowzan Alkuraya
Ziyad Allahem
Mohammad Allahtavakoli
Mehdi Allahyari
Jean-Pierre Allain
Theresa Allain
Benjamin Allaire
Robert Allaker
Suleyman Allakhverdiev
Iqbal Allam
Alison Allan
Andrea Allan
Bridie Allan
David Allan
Gerard Allan
Keith Allan
Richard Allan
Benoit Allard
Marc Allard
Veerajalandhar Allareddy
Veerasathpurush Allareddy
Nancy Allbritton
Karel Allegaert
Sarah Allegra
Andrew Allen
Annette Allen
Bryan Allen
Charles Allen
Geraldine Allen
Isabel Allen
Jane Allen
Keith Allen
Kyle Allen
Lee-Ann Allen
Margaret Allen
Matthew Allen
Matti Allen
Michael Allen
Pablo Allen
Richard Allen
Rosalind Allen
Sam Allen
Susan Allen
Tom Allen
Vivian Allen
Standish K. Allen Jr.
Matthew Allender
Diane Allen-Gipson
Emma Allen-Vercoe
Stefano Allesina
Randall Alley
Abdel Alli
Niccolò Allievi
Ney Alliey-Rodriguez
Juri Allik
John Allingham
Ed Allison
Kirk Allison
McFall Allison
Paul Allison
W. Ted Allison
Tamara Alliston
Richard Allmendinger
Heather Allore
Yves Allory
Mary Allred
Richard Allsopp
Anna Allue Guardia
John Allum
Thomas Ally
Alberto Alma
Juan Almagro
Pere Almagro
Amy Alman
Andrés Almansa
Abdullah Almansour
Badar Almarri
Alex Almasan
Ana Almeida
André Almeida
Andrea Almeida
António Almeida
Carina Almeida

Cláudia Choma Bettega Almeida
Danilo Almeida
Gustavo Almeida
Igor Almeida
Izabela Almeida
Marcia Almeida
Maria Almeida
Maria Ines Almeida
Matheus Almeida
Patricia Elaine Almeida
Raquel Almeida
Rodrigo Almeida
Vívian Almeida
Tânia Almeida Alves
Jean Almeida Espinoza
Mauricio Almeida-Gomes
Adriana Almeida-Rodriguez
Hesham Al-Mekhlafi
Saleh Almenawer
Muhammad Qutayba Almerie
Jose Almirall
Abdulrahman Al-Moghairi
Essam Almoraissi
Zack Almquist
Ahmad Al-Mrabeh
Kristian Almstrup
Hisham Al-Mubaid
Hamad Almuhannadi
A. Almuhit
Fuad Alnajjar
Jason Aloisio
Luigi Aloj
Abhay Alok
Basem Al-Omari
Ilan Alon
Debasmita Alone
Covadonga Alonso
Hany Alonso
Juan Alonso
Leandro Alonso
Sergio Alonso
Patricia Alonso-Fernández
Paloma Alonso-Magdalena
Noam Alperin
Allan Alpern
David Alpers
J. Alpert
Melchor Alpízar Salazar
Ali Alqarni
Ahmad Alqudah
Saed Alrabaee
Ghassan Alregib
Dhafer M. Alsaad
A. Al-Samman
Mohammad Alshayeb
Fatima Alshbool
Samah Alshehri
J. Alspaugh
Peter Alstone
Khaled Al-Surimi
Karen Alt
Abdullah Al-Taiar
Bassam Altamimi
Julio Altamirano
Leopoldo Altamirano
Luca Altamore
Mohamad Al-Tannir
Gheona Altarescu
William Altemeier
Ammar Altemimi
Jens Altenbernd
Markus Altenburger
David Alter
Ronnie Alterman
Kursat Altinbas
Ismail Sengor Altingovde
Marco Altini
Ismail Altintop
Doug Altman
Maria Altman
Andre Altmann
Friedrich Altmann
Laura Altobelli
Richard Altschuler
Abdullah Altunışık
Res Altwegg
Kishore Alugupalli
Maria Alva

Christine Alvarado
Marco Alvarenga
Bernardo Alvarez
Carlos Alvarez
Cecilia Alvarez
Cristian Alvarez
Eduardo Alvarez
Elena Alvarez
Eva Alvarez
Fernando Alvarez
Iñaki Alvarez
Julio Alvarez
José Álvarez-Castillo
Pedro Alvarez-Diaz
Diego Alvarez-Estevez
Cristina Alvarez-Martinez
Gerardo Alvarez-Uria
Andrew Alverson
Ada Alves
Benner Alves
Charline Alves
Gilberto Alves
Ricardo Alves
Sandra Alves
Tiago Alves
Inar Alves Castro
Karuza Alves Pereira
Fracisca Alves-Cardoso
Estevao Alves-Silva
Heidi Al-Wassia
Hany Aly
Markus Aly
Hussein Aly Ibrahim
Bandar Alyami
Ali Alzahrani
Maria Ida Amabile
Raghavendra Amachawadi
Seigo Amachi
Jeffrey Amack
João Amador
Kikuko Amagase
Mary Amalaradjou
M. Javad Aman
Diego Amancio
Mojtaba Amani
Diana Amantea
Claudio Amanti
Amanullah Amanullah
Ahmad Amanzada
Rama Rao Amara
Fernanda Amaral
Teresa Amaral
Helena Amaral Espírito-Santo
Renata Cecília Amaro
Sergio Amaro
S. Amasheh
Flore Amat
Gimano Amatngalim
Katherine Amato
Jason Amatoury
James Amatruda
Roshani Amatya
Fumimasa Amaya
Iván Amaya
Peter Amaya
Eric Amazeen
Polemnia Amazeen
Bharath Ambale-Venkatesh
Omotuyole Ambali
Rashmi Ambasta
Atul Ambekar
Gregory Amberg
Satyajit Ambike
Richard Ambinder
Christopher Ambrey
Lilliam Ambroggio
Peter Ambros
Divakar Ambrose
Concetta Ambrosino
Luigi Ambrosio
Renato Ambrosio
Geza Ambrus
Rikard Ambrus
Ole Herman Ambur
Suman Ambwani
Evans Ameade
Guillermo Ameer
Oluwatoyin Ameh

Elaine Amella
Rose Mary Amenga-Etego
Jennifer Amengual
Emilia Amengual-Cladera
Amal Amer
Bashar Amer
Hatem Amer
Pietro Ameri
Nancy Ames
Burim Ametaj
Carmen Amezcua-Prieto
Mohamed Amgad
K. Amico
Ali Amid
Angela Amigoni
Marcel Amills
Amnay Amimeur
Ruhul Amin
Samirkumar Amin
Fariba Amini
Mamuda Aminu
Saeed Aminzadeh
Lisa Amir
Alireza Amirbaigloo
Steve Amireault
Bahman Amiri
Esmaeil Amiri
Masoud Amiri
Mina Amiri
Z. Amiri Tehranizadeh
Mahmood Amiry-Moghaddam
Gabi Amitai
Eisuke Amiya
Muhammad Shoaib Amjad
Evžen Amler
Navneet Ammal Kaidery
Fabian Amman
El-Desouky Ammar
Christian Ammer
Brooke Ammerman
Joshua Ammerman
Sylwia Ammoun
Joshua Amo-Adjei
Linda Amoah
Winfried Amoaku
Antonio Amodeo
Olukemi Amodu
Behzad Amoozgar
Francisco Amorim
Irina Amorim
Vittoria Amorosi
Maria Amoroso
Lucia Amoruso
John Amory
Samson Amos
Julien Amour
P. Amouroux
Agbessi Amouzou
Apostolos Ampatzoglou
Emmanuel Ampofo
Luca Ampollini
Karin Amrein
Moez Amri
Manoj Amrutkar
Eli Amson
Orna Amster-Choder
Sylvie Amu
Timothy Amukele
Borko Amulic
Paul Amuna
Tore Amundsen
Melissa Amundson
Alexey Amunts
Bo An
Chansik An
Chunju An
Diaoguo An
Gary An
Hailong An
Li An
Nan An
Shi-Qi An
Yom An
Yu An
Allison Anacker
Ramon Anadon
Pinelopi Anagnostopoulou
David Anaki

Khairul Anam
Praveen Anand
S. Anand
Saket Anand
Ramu Anandakrishnan
Madhu Anand-Srivastava
Frank Anania
Cande Ananth
Ramya Ananthakrishnan
Vaishnavi Ananthanarayanan
Jaithri Ananthapavan
G. Anantharamaiah
Bharath Ananthasubramaniam
Adam Anas
Dafni Anastasiadi
Vikas Anathy
Alessandro Anav
Antonio R. Anaya
Juan-Manuel Anaya
Anand Anbarasu
Kumarasamy Anbarasu
Pieter Anborgh
Leonardo Ancillotto
Mebeaselassie Andargie
Elisar Andari
Nicole Andeen
Gregor Andelfinger
Matheu Ander
Robert Anderberg
Christine Anderl
Tessa Andermann
Hans-Joachim Anders
Juanita Anders
Sven Anders
Asger Andersen
Bogi Andersen
Catherine Andersen
Erik Andersen
Flemming Littrup Andersen
Jan Terje Andersen
Jens Peter Andersen
Kathryn Andersen
Kjeld Andersen
Mark T. Andersen
Nicholas Andersen
Niels Andersen
Niels Gerner Andersen
Petersejer Andersen
Alex Anderson
Annette Anderson
Ariana Anderson
Barbara Anderson
Benjamin Anderson
Brendan Anderson
C. S. Anderson
Carl Anderson
Charles Anderson
Craig Anderson
Cynthia Anderson
Deborah Anderson
Dennis Anderson
Douglas Anderson
Dwight Anderson
Emily Anderson
Erik Anderson
Ethan Anderson
Garret Anderson
Jacqueline Anderson
Janeane Anderson
Jason Anderson
Jeffrey Anderson
Jill Anderson
Joel Anderson
John Anderson
John T. Anderson
Karen Anderson
Kenneth Anderson
Kristin Anderson
Larry Anderson
Lucy Anderson
Martha Anderson
Melissa Anderson
Michael Anderson
Nathaniel Anderson
Nigel Anderson
O. Anderson
Paul Anderson
Rhys Anderson

Samira Anderson
Sarah Anderson
Sharolyn Anderson
Thomas Anderson
Todd Anderson
Vicki Anderson
Ariana E. Andersona
Björn Andersson
Jan Andersson
Karl Andersson
Karl-Erik Andersson
Maria Andersson
Martin Andersson
Mats Andersson
Rune Andersson
Anand Kumar Andiappan
Attila Andics
Nadya Andini
Anuska Andjelkovic
Thomas Andl
Fumiaki Ando
Istvan Ando
Kanae Ando
Sakai Ando
Shuntaro Ando
Francis-Xavier Andoh-Adjei
Noemi Andor
Danielle Andrade
Denis Andrade
Eduardo Andrade
Flavia Andrade
Galdino Andrade
Hélida Andrade
Juan Andrade
Lúcia Andrade
Luciano Andrade
Luis Andrade
Magaiver Andrade
Marcelo Andrade
Rosa Andrade
J. D. Andrade Filho
Fernando Andrade-Narvaez
Johanna Andrae
Ibolya Andras
Prabha Andraweera
Galuppo Andrea Giannotti
Elisabeth Andreadou
Ioanna Andreadou
Vincent Andrearczyk
Harry Andreassen
Maria Grazia Andreassi
Erik Andreasson
Kristofer Andréasson
Eran Andrechek
Dmitry Andreev
Elena Andreeva
Graciela Andrei
Marco Andrello
Veronica Andreo
Laura Andreoli
Solange Andreoni
Mauro Andreotti
Francesco Andreozzi
Aline Andres
Douglas Andres
Mariano Andrés
Bjarne Andresen
Celia Andreu-Sánchez
Angeline Andrew
E. Andrew
Rose Andrew
Andrew Andrews
Clinton Andrews
Elizabeth Andrews
Elizabeth. Andrews
Joel Andrews
Sam Andrews
Sarah Andrews
Simon Andrews
Timothy Andrews
H. J. Andreyev
Alex Andrianopoulos
Petra Andries
Iris Andriessen
Ovidiu Andronesi
Jan Andrysek
Anna Andrzejewska
Ritu Aneja

Susan Anenberg
Andrew Anesetti-Rothermel
Michael Anestis
Gianfranco Anfora
Laura Anfossi
Simone Anfossi
Michael Angarone
Baskaran Angathevar
Narayanasamy Angayarkanni
Andrea Angeletti
Gloria Angeletti
Silvia Angeletti
Paolo Angeli
Timothy Angeli
Frederic Angelier
Italo F. Angelillo
Christine Angelini
Claudia Angelini
David Angelini
Elsa Angelini
Samer Angelone
Tommaso Angelone
Maria Angelova
Andrej Angelovski
Arild Angelsen
Irene Angeluccetti
Francesco Angelucci
Renato Anghinah
Kenneth Angielczyk
Jeromy Anglim
Fabrice Ango
Marco Angriman
Javier Angulo
Sergio Angulo
Brian Angus
Luis Anibarro
Eric M. Anicich
Cuneyd Anil
Mohammad Hossein Anisi
Abdul Aziz Aniza
Muna F. Anjum
Mustafa Ankarali
Cristopher James Anker
Daniel Ankrah
Anna Carbone Anna Carbone
Anandhan Annadurai
Augustina Annan
Christine Anne Ganzer
Christopher Annear
Vito Annese
James Annesi
Brian Annex
Bruno Annibale
Paolo Annibale
Peter Annighöfer
Andrea Annoni
Maria Grazia Annunziata
Rachel Annunziato
Maria Añón
Makoto Anraku
Benjamin Ansa
Mireille Ansaldi
Arya Ansari
Bilal Ansari
Hamid Reza Ansari
Helal Ansari
Mairaj Ansari
Moien Ansari
Suhail Ansari
Sharon Ansboro
Laura Anselmi
Kristi Anseth
Muhammad Anshari
Eric Anson
Yonathan Anson
Chrisph Ansorge
Matthew Anstey
Nicola Anstice
Davor Antanasijevic
Michael Anteau
Nadav Antebi-Gruszka
Francisco Antequera
Juliana Antero-Jacquemin
Donald Anthony
James Anthony
Karen Anthony
Nicholas Anthony
Richard Anthony

Tracy Anthony
Tamil Selvan Anthonymuthu
Fabrice Antigny
Joseph Antin
Dmitry Antipov
Ya Antipov
Marlies Antlanger
Dujmovic Anto
Antonio Antoccia
Pierre-Olivier Antoine
Thessicar Antoine
Sophie Antoine-Jonville
Armand Antommaria
Andrea Anton
Sylvia Anton
Alessandro Antonelli
Antonella Antonelli
Cristiano Antonelli
Marta Antonelli
Raffaele Antonelli Incalzi
Costin Antonescu
Chrystalina Antoniades
Marco Antoniades
Anastasia Antoniadou
William Antonialli
Sylvain Antoniazza
Angelo Antonini
Gloria Antonini
Andréa Antonio
Filippini Antonio
Stavros Antoniou
Sabina Antoniu
Katarina Antonsdotter Hylthén
Emilia Antonucci
Toni Antonucci
Binu Antony
Justin Antony
Veena Antony
Jędrzej Antosiewicz
Athanasios C. Antoulas
Natalia Antropova
Carlos Antunes
Edson Antunes
Elsa Antunes
Luis Antunes
Paula Antunes
Markus Antwerpen
Ainur Anuar
Kingsley Anukam
Dilly Anumba
Krishnamurthy Anupama
Jana Anvari
Iqbal Anwar
Masood Anwar
Muhammad Anwar
Salvatore Maria Anzalone
Michele Anzidei
Hong Ao
Hushan Ao
Mingxin Ao
Ping Ao
Shinya Aoi
Takashi Aoi
Fred Aoki
Junken Aoki
Koh Aoki
Maria Aoki
Naohiro Aoki
Takaaki Aoki
Takatoshi Aoki
Tatsuo Aoki
Miguel Aon
Fauze Aouada
Mabel Aoun
Takashi Aoyama
Takuma Aoyama
Tomoki Aoyama
Eunice Aparecida Bianchi Galati
Abelardo Aparicio
David Aparicio
Hugo Aparicio
Oscar Aparicio
Samuel Aparicio
Virginia Aparicio
Esin Apaydin Varol
Nareg Apelian
Bettye Apenteng
Yiorgos Apidianakis

Benjawan Apinonkul
Andrew Aplin
Gerard Apodaca
Daniel Apolinario
Camilo Aponte-Santamaria
Patrick Apopa
João Apóstolo
Nadezda Apostolova
Art Appel
Silke Appel-Cresswell
Klaus Appenroth
Bernard Appiah
Kofi Appiah
Brian Appleby
Roger Applegate
Wendy Applequist
Luca Appolloni
Tamar Aprahamian
Alessio Aprile
William Apro
Werner Apt
Manasi Apte
Minoti Apte
Suneel Apte
Udayan Apte
Alan Apter
Gisele Apter
Deborah Apthorp
Stefano Aquaro
James Aquavella
Christopher Aquina
Gabriella Aquino
Jailane Aquino
Ignacio Ara
Marzieh Araban
Yaseen Arabi
Michal Arabski
Demet Arac
Ramos Araceli
Mallikarjuna Aradhya
Chinmay Aradhye
S. M. Arafat
Waleed Arafat
Henio Henrique Aragão Rêgo
Pablo Aragón
Minori Arahori
Ken Arai
Toshiro Arai
Muhammad Arain
Yasuyuki Arakane
Kazuharu Arakawa
Satoko Arakawa
Hitoshi Araki
Kimi Araki
Shin-Ichi Araki
Elisa Araldi
Elisabete Aramendi
Spyridon Arampatzis
Francisco Arancibia
Walter Arancio
Agustin Aranda
Mimi Arandjelovic
Amelia Aránega
Zolt Arany
Maria Jose Aranzana
Hideki Araoka
Toshikazu Araoka
Joaquin Araos
Romulo Araoz
Isil Aras
Toru Arase
Ryan Arathimos
Carla Araujo
Daniele Araujo
Mariana Araujo
Natalia Araujo
Ricardo Araujo
Ronaldo Araujo
António Araújo
Gabriel Araújo
Livia Araujo de Carvalho
Lara Arauna
Gayatri Aravind
Shigeki Arawaka
Azizollah Arbabisarjou
Kestutis Arbaciauskas
Brian Arbogast
Victoria Arbour

Benjamin Arbuckle
Fritzie Arce
Kevin Arceneaux
Ezequiel Arceo-May
Peter Arcese
Annie Archambault
Denis Archambault
Éric Archambault
Vincent Archambault
Amena Archer
Cristina Archer
Crystal Archer
John Archibald
Lisa Archibald
Nancie Archin
Govindaraju Archunan
Nico Arcilla
Carla Renata Arciola
Vincenzo Arcoraci
Babak Ardekani
Jeffrey Ardell
Amanda Ardente
Moshe Arditi
Julie Ardley
Daniela M. Arduíno
Ana Paula Arêas
Yupaporn Areepong
Gabriel Arellano
Lucrecia Arellano
Olatunde Aremu
Fabio Arena
Claudia Arenaccio
Francisco Arenas
Angela M. Arenas-Gamboa
Cesar Arenas-Mena
Alejandro Arenas-Pinto
Douglas Arenberg
Isabel Arend
Jan Arends
Lisa Arendt
Michael Arendt
Glenn Arendts
A. Katrin Arens
Philipp Arens
Christine Arenson
Matthew Arentz
Juan Arevalo
José Ramón Arévalo
Cynthia Arfken
Pedram Argani
Peter Argenta
Ahmadreza Argha
Angel Argiles
Maria Argos
Françoise Argoul
Maria Argudín
Amaël Arguel
Anthoula Argyri
Stelios Arhondakis
Alarico Ariani
Margarita Arianoutsou
Natalia Arias
Sarah Arias
Miguel Arias Estrada
Alejandro Arias Vasquez
Yassine Aribi
A. Aricescu
Tomoki Arichi
Maurizio Arico
Barak Ariel
Frederic Ariey
Khalid Arif
Mohammad Arif
Siti Arifin
Wan Nor Arifin
Damla Arifoğlu
Muthuvel Arigovindan
Ingrid Arijs
Siwaret Arikit
Rubab Arim
E. Arima
Shuji Arima
Takahiro Arima
Taro Arima
Kohki Arimitsu
Shin-Ichi Arimura
Joaquín Ariño
Stefania Arioli

Robert Aris
Resul Arisoy
Ines Aristegui
Nobuko Arisue
Mikio Arita
Anoma Ariyawardana
Miguel Ángel Ariza Gracia
Jalal Arjomandi
Elizabeth Arkema
Alexander Arkhipkin
Kenton Arkill
Mary Arkoosh
Jean-Benoît Arlet
Frederick Armah
Andrea Armani
M. Armaou
Jonathan Armbruster
Jean-Baptiste Armengaud
Jose Angel Armengol Butrón de Mújica
Paul Armistead
Ian Armit
Kenneth Armitage
Mike Armour
Ian Armstead
William Armstead
Daniel Armstrong
Glen Armstrong
J. Armstrong
Margaret Armstrong
Melissa Armstrong
Nicola Armstrong
Nicole Armstrong
Sandra Armstrong
Jean-Francois Arnal
Fernanda Arnaldez
Rima Arnaout
George Arnaoutakis
Nicolas Arnaud
Karen Arndt
Patrick Arndt
Alain Arneodo
Luis Arnes
Isabelle Arnet
Antje Arnold
Craig Arnold
Denis Arnold
Dirk Arnold
Donald Arnold
Michelle Arnold
Sarah Arnold
Steven Arnold
W. Arnold
Maria Arnone
David Arnosti
Elizabeth Arnott
Martijn Arns
Luiz Aroeira
Neelima Aron
Neil Aronin
Bruce Aronow
Wilbert Aronow
Jan Magnus Aronsen
Dominik Aronsky
Brian Aronson
Doron Aronson
Jeffrey Aronson
Naomi Aronson
Anju Arora
Gaurav Arora
Manish Arora
Naresh Arora
Rishi Arora
Sandeep Arora
Vineet Arora
Paolo Arosio
Bruno Arpino
Amaia Arranz-Otaegui
Gloria Arratia
Marco Arrese
Agnes Arrey
Protus Arrey Tarkang
Gustavo Arriaga
Gabriela Arrifano
F. Arrighetti
Sebastian Arriola Apelo
Holly Arrow
Cheryl Arrowsmith
Juan Arroyo

Jorge Arroz
E. F. Arruda
Marco Antonio Arruda
Paulo Arruda
Ethem Arsava
Jbid Arsenyan
Mehreen Arshad
Ayca Arslan-Ergul
Javier Arsuaga
Mehmet Artac
Olatz Arteaga
Catalina Arteaga de Castro
Kyle Artelle
Panagiotis Artemiadis
Enrique Artero
Carlos Arteta
James Arthos
Agnes Arthur
Janelle Arthur
Mary Arthur
Rohan Arthur
Terrance Arthur
Oriol Artime
Guilherme Artioli
Carol Artlett
Isabella Artner
Ferruh Artunc
M. Arufe
Velmurugesan Arulampalam
Sophie Arulkumaran
Baskar Arumugam
Vaithilingaraja Arumugaswami
Banu Arun
Nanditha Arun
N. Arunachalam
Sudha Arunachalam
Lauren Arundell
Anshu Arundhati
Shalini Arunogiri
Carles Arus
Patrick Arveux
Mina Arvin
Achyut Aryal
Krishna Aryal
Muna Aryal
Uma Aryal
Juvid Aryaman
Ni Aryastami
Okailey Aryeetey
Amirhossein Arzani
Jonathan Arzt
Sylvia Asa
Nana Asabere
Abu Bakar Ali Asad
Shinichi Asada
Md. Asaduzzaman
Jamshid Asadzadeh
Kinji Asahina
Yasuhiro Asahina
David Asai
Manabu Asai
Yusuke Asai
Tetsuya Asakawa
Takanori Asakura
Tomiko Asakura
Kwame Asamoa
Katsura Asano
Kenichi Asano
Kenichiro Asano
Manabu Asano
John Asara
Kei Asayama
Penny Asbell
Jakob Asberg
Michaela Aschan
Frank Asche
Jörg Aschenbach
Andy Aschenbrenner
David Ascher
Jill Ascher
Dana Ascherman
Daniela Aschieri
Gianluca Ascolani
Gualberto Asencio-Cortés
Likawunt Asfaw
Sassan Asgari
Waseem Asghar
Hafez Asgharzadeh

Dipankar Ash
Stephen Ash
Nathaniel Ashby
Richard Ashby
Mark Ashcraft
Mark Ashe
Muhammad Ashfaq
Paul Ashigbie
Neal Ashkanasy
Yosef Ashkenazy
Elizabeth Ashley
Michael Ashley
Patricia Ashley
Richa Ashma
Amy Ashman
Jonathan Ashmore
Rehab Ashour
Muhammad Ashraf
Kaveh Ashrafi
Toni Ashton
Jonathan Ashwell
Chris Ashwin
Paul Ashwood
William Ashworth
Laureano Asico
Salah Asida
Michael Asiedu
Robert A. Asiedu
Mehar Asif
Angeliki Asimaki
Kjetil Ask
Deborah Askew
Robert Askins
Johan Askling
Maria Askmyr
Rebecca A. Aslakson
Adnan Aslam
Muhammad Aslam
Sher Aslam
Dilek Aslan
Ioannis Aslanides
Oleg Aslanidi
Reto Asmis
James Asmus
Laura Asnaghi
Ahmad Asoodeh
Nils Asp
Javier Aspa
Julie Aspden
Danijela Ašperger
Esther Aspinall
Mark Asplen
Becca Asquith
Fariba Assadi-Porter
Michal Assaf
Shervin Assari
Sara Assecondi
Yibeltal Assefa
Rosanna Asselta
Mahfoud Assem
Gianni Assenza
Ayman Assi
Tarek Assi
Elisa Assirelli
Ananda Assis
Benoît Assogba
Sabrina Assoumou
Pascal Astagneau
Janie L. Astephen Wilson
Rajiv Asthana
Sarah Astill
Susana Astiz
Laura Astolfi
Elizabeth Aston
Todd Astorino
Elaine Astrand
Renato Astray
Anne Nordrehaug Astrøm
Giovanni Astuti
Jagadamba Aswathappa
Attila Aszodi
Kamran Atabai
Narges Atabaki
Ricardo Ataide
Livia Ataíde
Koray Atalag
Caglar Ataman
Sergei Atamas

Biljana Atanasova
Kalina Atanasova
Boyko Atanassov
Pascal Nji Atanga
Abdon Atangana
U. H. Atasever
Polly Atatoa Carr
Victoria Ateca
Irene Athanassakis
Christos Athanassiou
Simone Athayde
Giridhar Athrey
Sheela Athreya
Vidya Athreya
Yacine Atif
Ximena Atilano-Carsi
Canan Atilgan
Gul Atilla
William Atiomo
Nadia Atiya
Chloe Atkins
Ryan Atkins
Mark Atkinson
Michael Atkinson
Prescott Atkinson
Stephanie Atkinson
Stephen Atkinson
Ella Atlas
Reynir Atlason
Venkata Atluri
Barbaros Atmaca
Sachin Atre
Pranita Atri
José Joaquín Atria
Phimon Atsawasuwan
Masanori Atsukawa
Tatsuya Atsumi
Simon Kwaku Attah
Deanna J Attai
Geoffrey Attardo
Rajpaul Attariwala
Husain Attarwala
Navid Attary
Giovanna Attene
Matias Attene Ramos
Ahmed Attia
Alan Attie
Durga Attili
Domenico Attinà
Liliana Attisano
Nii Attoh-Okine
Kar Attramadal
Kuldeep Attri
Ravindra Attur
Fidelis Atuo
Gurdeep Atwal
Craig Atwood
Fabiola Atzeni
Shiu Lun Ryan Au Yeung
Lauren Aubert
Mylène Aubertin-Leheudre
C. Aubron
Berengere Aubry-Rozier
Georg Auburger
Matteo Audano
Heinrich Audebert
Carolyn Audet
Gerald Audette
Jonathon Audia
Sylvain Audia
Francois Audibert
Carina Audisio
Paolo Audisio
Asta Audzijonyte
Carol Auer
Manfred Auer
Nancy A. Auer
Ulrike Auer
Joshua Auerbach
Michael Auerbach
Randy Auerbach
Sanford Auerbach
Victoria Auerbuch
Christoph Aufricht
Christopher Auger
Nathalie Auger
Sandrine Auger
David August

Furaha August
Paul August
Peter Auguste
J. C. Augustin
Jean Augustinack
Karen Augustine-Rauch
Antonios Augustinos
Livia Augusto
Rosângela Augusto
Per Augustsson
Robert Augustyniak
Ryszard Auksztulewicz
J. B. Auliac
Keith Aune
Eindra Aung
Myo Nyein Aung
Thet Aung
May Aung-htut
Timothy Aungst
Christine Aurich
Elisabetta Aurino
Igor Aurrekoetxea
Gabriele Ausiello
Alessio Ausili
Brandon Ausk
Marcel Ausloos
Laurent Aussel
Sabine Aust
Marie Austdal
Emily Austen
Erin Austen
Joseph Austen
Bradley Austin
Elizabeth Austin
Gregory Austin
R. Austin
Ryan Austin
Chantal Autexier
Matthieu Authier
Philippe Autier
Timo Autio
Matthew Auton
Brigitte Autran
Stephane Auvin
Petri Auvinen
Paul Auwaerter
Byron Au-Yeung
Georg Auzinger
Javier Avalos
Raga Avanasi Avanas
Laura Avanzino
Michele Avanzo
R. Avasare
Fikri Avci
Meltem Avci-Adali
T. V. Avdeenko
Selmir Avdic
Christine Avena
Annemariè Avenant-Oldewage
Sofia Avent
Bruno Averbeck
Susan Averett
Alexander Averianov
Sergey Averkin
Julien Averous
Amanda Avery
Ann Avery
Yossef Avgay
Sharon Avidor
Hillel Aviezer
Andrea Avila
Daiana Avila
Guillermo Avila
Jacob O. Avila
Carla Ávila
Federico Avila-Moreno
Manuel Aviles
Edvard Avilés
Amir Aviram
Eli Avisar
Abraham Aviv
Meital Avivi-Reich
Dror Avni
Yoram Avnimelech
Alberto Avolio
Karen Avraham
Evangelia Avramidou
Dimitrios Avramopoulos

Arnaud Avril
Natalia F. Avrova
Joseph Avruch
Dimitrios Avtzis
Abdelmoneim Awad
Ali Ismail Awad
Ola Awad
Ahmed Awadein
Shakil Awan
Gordon Awandare
Sita Awasthi
Rajeshwar Awatramani
Yitagesu Aweke
Elizabeth Awick
Tunmise Awojobi
Quentin Awori
David Axelrod
Rita Axelrod
Paul Axelsen
Jonas Axelsson
Pierre-Paul Axisa
Michael Axtell
Frolan A. Aya
Nagi Ayad
Masahiko Ayaki
Ana Patricia Ayala
Marcela Ayala
Yuna Ayala
Mohammed Ayalew
Nir Ayalon
Adeolu Babatunde Ayanwale
Parviz Ayazi
Alper Aydemir
Anna Lisa Aydin
Betul Aydin
Koray Aydin
Nizamettin Aydin
Suleyman Aydin
Tuba Aydin
Dogu Baran Aydogan
Asnakew Ayele
Olufemi Ayeni
Duncan Ayers
Jacob Ayers
Joe Ayers
John Ayers
Katherine Ayers
Mustafa Batuhan Ayhan
Burcu Aykac Fas
June Ayling
Kieran Ayling
Juan Ayllon
Segolene Ayme
Laure Aymé
Koya Ayonrinde
Nadia Ayoub
Florence Ayral
Jaya Aysola
Serpil Aytac
Darshini Ayton
Jennifer Ayton
Scott Ayton
A. Aytulun
Maria Ayub
Maria Ayuela
Miriam Ayuso
Muniappan Ayyanar
B. Vijayalakshmi Ayyar
Velpandi Ayyavoo
Abed Azab
Abul Kalam Azad
Kishwar Azad
Sandro Azaele
Henri Azais
Muhammad Azam
Hamed Azami
Beatriz Azanza
Taj Azarian
Andrea Azcarate-Peril
M. Andrea Azcarate-Peril
Veronica Azcutia Criado
Abdussalam Azem
Catarina Azeredo
Ana Raquel Azevedo
Manuela Azevedo
Nuno Azevedo
Paulo Azevedo
Ruben Azevedo

Ricardo Azevedo Pereira
Ezinwanne Azfredrick
Husain Aziz
Hussein Aziz
Moammir Aziz
Syed Aziz
Tipu Aziz
Zahra Aziz
Kamalrul Azlan Azizan
Fawaz Azizieh
Rasoul Azizipanah-Abarghooee
Andrew Azman
Azlinda Azman
Shazli Azmi
Shila Azodi
Joseph Azok
Maria Azrad
Armando Azua-Bustos
Andres Azuero
Miyuki Azuma
Mizuki Azuma
Toshifumi Azuma
Yoshiaki Azuma
Sausan Azzam
Carlo Azzarri
Mohammad Azzeh
Lorenzo Azzi
Ricardo Azziz
Binukumar B.
Abhay B. Ramachandra
Patrice Baa-Puyoulet
Keith Baar
Rodrigo Baarderas
Jantiene Baartman
Paul Baas
Hideo Baba
Kenkichi Baba
Reizo Baba
Fatima Baba-Ari
Vladimir Babaev
Stella Babalola
Sankhiros Babapoor
Nicolas Babault
Anna Babayan
Akira Babazono
Paul Babb
Elizabeth Babcock
Molly Babel
Marc Babi
Peter Babinec
Gabriele Babini
Juliet Babirye
John Babish
Sudhir Babji
Tatiana Babkina
George Bablekos
Jeff Babon
Ben Babourina-Brooks
Vytenis Babrauskas
B. Kalyana Babu
Ganesh Babu
Giridhara R. Babu
Mohan Babu
Subramanian Babu
Sergio Babudieri
Ganesh Babulal
Ana Baburamani
Andy Babwah
Mateusz Baca
Robert Bacallao
Marcos Antonio Bacarin
Mark Baccei
Dario Bacchini
Christian Bacci
Jonas Bacelis
Fernando Bach
Lennart Bach
Richard Bach
Ketema Bacha
Phil Bachand
Dominique Bachelet
Herve Bachelez
Benedicte Bachelot
Cédric Bacher
Daniela Bacherini
Kurt Bachmaier
Sara Bachman
Lutz Bachmann

Michael Bachmann
Markus Bachschmid
Mahesh Bachu
Roberto Bacilieri
Susanne Back
H. Backman
Samuel Backman
Richard Backs
Charles Bacon
Christopher Bacon
Oliver Bacon
Karl Bacos
Istvan Baczko
Xóchitl Bada
Ekaterina Badaeva
Ines Badano
Amel Badawi
Christopher Badcock
Roland Baddeley
Melissa Badding
Nahed Baddour
Natalie Baddour
Alexandra Badea
Robert Badeau
Raul Badell
Andrea Baden
Tom Baden
Klaus Badenhoop
David Bader
Kenneth Bader
Mazen Bader
Oliver Bader
Catherine Badgley
Grayson Badgley
Giovanni Badiali
Behnam Badie
Parisa Badiee
Anand Badigannavar
Juan J. Badimon
Pierre Badin
Raghuram Badmi
Ebrahim Badran
Mohamed Badr-El-Dine
Motasim Badri
Farid Abd Elreheem Badria
Stephen Badylak
Gi-Yeul Bae
Hyunsu Bae
Jeehyeon Bae
Jin-Woo Bae
Joonwon Bae
Kwang-Hak Bae
Sang Kyun Bae
Sang-Cheol Bae
Sejong Bae
Young Min Bae
Yun Soo Bae
Gyeong-Hun Baeg
Joonsang Baek
Jung Hwan Baek
S. H. Baek
Seungik Baek
Yousoon Baek
Guy Baele
Rebecca Baer
Melissa Baese-Berk
Jared Baeten
Delphine Baetz
Sonia Baeva
Selene Báez
J. Antonio Baeza
Marcelo Baeza
Raymundo Baez-Mendoza
Amit Bafana
Nirmalya Bag
Sumit Bag
Ujwal Bagal
Tarique Bagalkot
Mihir Bagchi
Milan Bagchi
Binnur Bagci
Ulas Bagci
Deniz Bagdas
Umesh Bageshwar
Giosue Baggio
Hugo Baggio
Aaron Baggish
James Baggs

Mohammad Bagheri
Saghar Bagheri
Zahra Bagheri
Habib Baghirov
Cedo Bagi
Demetrius Bagley
Allesadro Bagnato
Virginie Bagneux
Arianna Bagnis
Paola Bagnoli
Kathleen L. Bagot
Aditya Bagrodia
Ranjit Bahadur
Nixon Bahamon
El Mustapha Bahassi
Hilla Bahat
Walaa Baher
Ahmed Bahieldin
Suhad Bahijri
Justin Bahl
Christie Bahlai
Jörg Bahm
Amir Sajjad Bahman
Thomas Bahmer
Angela Bahns
Sola Bahous
Nathan Bahr
Elmostafa Bahraoui
Mohamed Ali Bahri
Pankaj Bahuguna
Aiping Bai
Bin Bai
Bing Bai
Bo Bai
Fan Bai
Ge Bai
Haihua Bai
Hua Bai
Jane Bai
Jianfa Bai
Jiawei Bai
Jie Bai
Jinhe Bai
Julio Bai
Junhong Bai
Péter Bai
Shasha Bai
Shu-Nong Bai
Songling Bai
Xiaoyan Bai
Xuejun Bai
Yaohui Bai
Yuling Bai
Yuqiang Bai
Zhi Yi Bai
Simone Baiardi
Ilaria Baiardini
Maria Baias
Shakuntala Baichoo
Frank Baiden
Uwe Baier
Ahmed Baig
Mumtaz Baig
Nath Baiju
Jongyoun Baik
Diego Bailetti
Frank Bailey
Heather Bailey
Helen Bailey
James Bailey
Julia Bailey
Kira Bailey
Michael Bailey
Regan Bailey
Richard Bailey
Robert Bailey
Shara Bailey
Stephanie Bailey
Stephen Bailey
Steven Bailey
Susan Bailey
Howard Bailit
Renée Baillargeon
Andrew Baillie
Ian Baillie
Louisa Baillie
Aurelien Baillon
Marc Bailly-Bechet

Jeremy Bailoo
Calum Bain
Daniel Bain
Daryl Bainbridge
Kathleen E. Bainbridge
Wilma Bainbridge
Michael Baine
Anthony Baines
Debbie Baines
John Baines
Francesco Baino
Leonardo Baiocchi
Woei-Nan Bair
Kalyana Bairapareddy
Andrew Baird
Donald Baird
Ian Baird
Kevin Baird
Liam Baird
C. Noel Bairey Merz
Laurent Bairy
Manish Bais
Jamie Baisden
Daniele Baisero
Víctor Baizabal-Aguirre
José Fidel Baizabal-Carvallo
Jasmohan Bajaj
Daria Bajerlein
Juliana Bajic
Vladan Bajic
Ana Bajo
Baubak Bajoghli
Pietro Bajona
Akhilesh Bajpai
Jyoti Bajpai
Prachi Bajpai
Ashish Bajracharya
Ali Bajwa
Maarten Bak
Petros Bakakos
Jeffrey A. Bakal
Muhammad Zubair Abu Bakar
Nur Salwani Bakar
Tamam Bakchoul
C. Baker
Chris Baker
Christopher Baker
David Baker
Edward Baker
Henry Baker
Jess Baker
Joseph Baker
Joshua Baker
Marc A. Baker
Matthew Baker
Max Baker
Nevin Baker
Olga Baker
Robert Baker
Sal Baker
William Baker
Hooman Bakhshi
Inger Bakken
Christian Bakker
David Bakker
Jamie Bakkum-Gamez
Giorgos Bakoyannis
Mahmoud Bakr
Tatjana Bakran-Petricioli
George L Bakris
Shelly Bakst
Chandrasekhar Bal
P. Bal
Elif Bal Beşikçi
Carey Balaban
Praveen Balabaskaran Nina
Anoop Balachandran
Kartik Balachandran
Premalatha Balachandran
Vinod Balachandran
Olga Balafa
Priyanka Balaganapathy
P. Babu Balagopal
Kuberan Balagurunathan
Mohamed Balaha
M. Balaji
Hanumantha Rao Balaji Raghavendran
Kalpana Balakrishnan

Karuppiah Balakrishnan
Pachamuthu Balakrishnan
V. Balamurugan
Fran Balamuth
Ivan Balan
Venkatesh Balan
Alfonso Balandra
R. Balandrán-Quintana
Pooja Balani
Katherine N. Balantekin
Vicent Balanza
Vicente Balanzà
Katherine D. Balasingham
Márta Balaskó
Michal Balass
Ronald Balassanian
Dorairajan Balasubramanian
Poonkuzhali Balasubramanian
Sivakumar Balasubramanian
Sundaravadivel Balasubramanian
Sanjeeva Balasuriya
Giovanni Balato
Praful Balavant Balavant
David Balayssac
Carolina Balazs
Margit Balazs
Mahmoud Balbaa
Sebastian Balbach
Andre Balbi
María Elvira Balcells
R. Torra Balcells
Taye Balcha
Hatice Balci Yuce
Gerd Balcke
Carsten Balczun
Daniela Baldantoni
Loretta Baldassar
R. P. Baldasso
J. Baldauf
Arturo Balderas
Nicholas Balderston
Alfonso Baldi
Elisabetta Baldi
Pierre Baldi
Alberto Baldini
Nicola Baldini
Matheus Baldissera
Guilherme Baldo
Marcelo Baldo
Dennis Baldocchi
Richard Baldock
Laura Baldomà
Megan Baldridge
Lodovico Balducci
Andrew Baldwin
Austin Baldwin
Cynthia Baldwin
James Baldwin
Jennifer Baldwin
Keith Baldwin
Susan Baldwin
Dumitru Baleanu
Bachir Balech
Olivier Balédent
Pablo Balenzuela
Panagiotis Balermpas
Constantino Balestra
Giorgio Balestra
Damien Balestrino
Christophe Baley
Tanjore Balganesh
Kiran Bali
Stefano Balietti
Reshma Baliga
Vikram Baliga
Peter Balint-Kurti
Mitchell Balish
Carmela Balistreri
Ethan Balk
Lisanne Balk
Wayne Balkan
Beverley Balkau
Jennifer Balkus
Frances Balkwill
Andrew D. Ball
Barry Ball
Simon Ball
Steven Ball

Terry Ball
Tonio Ball
Anand Ballal
Petek Ballar
Clive Ballard
Guy Ballard
Kirrie Ballard
Cherry Ballard-Croft
Francesca Ballarini
Andrea Ballatore
Marlies Ballegeer
Nikolaus Ballenberger
Joseph Ballenger
Laura Ballerini
Sergi Ballespí
Coralio Ballester
Diana Ballesteros
Manuel Ballesteros
Stefano Ballestri
Britta Ballhausen
Jean-Luc Balligand
Robert Balling
Megan Ballinger
Peter E. Ballmer
Daynia Ballot
Jon Ballou
Michael Ballou
Lauren Balmert
John Balmes
Zsolt Balogh
Esra Baloglu
Maria Balota
Sadegh Balotf
Cabral Balreira
Michela Balsamo
Elise Balse
Sigal Balshine
Steve Balsis
Henrik Balslev
Marie Balslev-Harder
Joshua Balsters
Brian Balta
Ivan Baltadzhiev
Valeria Baltar
Baltazar Baltazar
Isabelle Baltenweck
Vincent Balter
Jacques Balthazart
Simone Baltrusch
Jay Baltz
Pascal Baltzer
Peter Baluk
Dodge Baluya
Ondrej Balvin
Bijorn Balzamino
Laura Balzer
Colleen Bamford
Bert Bammens
Stephen Ban
Yusuke Ban
Maciej Banach
Niaz Banaei
Mahzarin Banaji
Rafael Bañares
Neil Banas
Pavel Banas
Kailash Banasure
Agata Banaszek
A. Banazadeh
Germana Bancone
Vimla Band
Jorge Banda
Nirmal Banda
Fatemeh Bandarian
Altaf H. Banday
Manuel Bande
Borwin Bandelow
Sunita Bandewar
Swati Bandi
Silvia Bando
Gretchen Bandoli
Sascha Bandulik
Arunava Bandyopadhaya
Amal Bandyopadhyay
Dhrubajyoti Bandyopadhyay
Dipankar Bandyopadhyay
K. K. Bandyopadhyay
Jonas Banefelt

A. Banerjee
Aparajita Banerjee
Arup Banerjee
Debabrata Banerjee
Debarshi Banerjee
Dibyajyoti Banerjee
Imon Banerjee
Jheelam Banerjee
Kaushik Banerjee
Manidipa Banerjee
Pratik Banerjee
Priyam Banerjee
Rebecca Banerjee
Rintu Banerjee
Sagarika Banerjee
Sandip Banerjee
Sanjay Banerjee
Sanjeev Banerjee
Santanu Banerjee
Sharmistha Banerjee
Soma Banerjee
Sreeparna Banerjee
Sube Banerjee
Sudip Banerjee
Sulagna Banerjee
Sushanta Banerjee
Versha Banerji
Zsófia Bánfalvi
Akash Bang
Christofer Bang
Heejung Bang
Lasse Bang
S. Banga
Jörg-Martin Bangen
Adrian Bangerter
James Bangs
Max Bangs
Gabor Banhegyi
Paolo Bani
Aria Baniahmad
Alice Baniel
Pabitra Banik
Pauline Baniqued
Thomas Banitz
Bettina Bankamp
Roxanne Banker
Richard Bankert
Vassya Bankova
David Banks
H. Thomas Banks
M. Banks
Martin Banks
Scott Banks
Jinan Banna
Wilbert Bannenberg
Rajdeep Bannerjee
Norbert Bannert
Antje Banning
Kristy Bannister
Roger Bannister
Georgios Banos
Nuria Banos
Dipika Bansal
Meena Bansal
Monika Bansal
Mukesh Bansal
Mukul Bansal
Ruchi Bansal
Christopher Banse
James Banta
Heike Bantel
Helen Banwell
Ashley Banyard
Bin Bao
Changjun Bao
Jiusheng Bao
Shuming Bao
Sonia Bao
Sujin Bao
Wei Bao
Wenzheng Bao
Xiaodong Bao
Xiaoming Bao
Xiaoyong Bao
Yan-Ping Bao
Yantao Bao
Yinguang Bao
Yongming Bao

Yuping Bao
Yuqian Bao
Zhenmin Bao
Zhirong Bao
Anton Baotic
Marine Baptissart
Abrahão Baptista
Murilo Baptista
Tiago Baptista
Peter Barabas
Silvia Barabino
Aleksandra Barac
Ana Barac
Andrea Baragetti
Ivano Baragetti
Meir Barak
Amr Barakat
Ruben Barakat
Gehanath Baral
Pravas Baral
Larissa Baraldi
Agnieszka Baran
Timir Baran Jha
Yoav Bar-Anan
Pilar Sainz De Baranda
Petr Baranov
V. Baranov
Ancha Baranova
Vikas Baranwal
Sergio Baranzini
Edwine Barasa
Nikolay Barashkov
Marco Barassi
Ashoktaru Barat
Carlos Barata
Keith Baratz
Alberto Barausse
Emilio Barba
Paola Barba
Rafael Barba-Alvarez
Ignazio Barbagallo
Isabel Barbancho
Cecile Barbaret
Christian Barbato
Mario Barbato
Mary Barbe
Jayme Barbedo
Lindley Barbee
Anjuli Barber
Sarah Barber
Stuart Barber
Lisa Barbera
Joan Barberà
Giuseppe Barberio
Matthew Barberio
Andrea Barberis
Shannon Barber-Meyer
Anthony Barbet
Egidio Barbi
Mariette Barbier
Chiara Barbieri
Fabio A. Barbieri
Fabio Barbieri
Katherine Barbieri
Lucia Barbieri
Nicolle Barbieri
Ricardo Barbieri
Silvia Stella Barbieri
Dominique Barbolosi
Doris Barboni
Angela Barbosa
Dulce Barbosa
Jeam Barbosa
Karla Barbosa
Leandro Barbosa
Diana Barbosa Cunha
Sandra Barbosa-Silva
Edel Barbosa-Stancioli
Natalia Barboza
Sukhadeo Barbuddhe
Adrian Barbul
José Alexandre Barbuto
Maria Barca
Jacqueline Barcelar
Christovam Barcellos
Franklin Barcellos
Silvia Barcellos
Pedro Barcellos-de-Souza

Maria Luisa Barcena de Arellano
Ilaria Barchetta
Aaron Barchowsky
Marcello Barcinski
Robert Barclay
Frederic Bard
Michal Bardecki
John Barden
Janine Barden-O'Fallon
Maria Barderas
Nathalie Bardet
Valerie Bardet
Dwaipayan Bardhan
Soraya Bardien
Bart Bardoel
Dana Bardolph
Babara Maria Bardoni
Edouard Bardou-Jacquet
Linnea Bärebring
David Barefield
Nathalie Bareille
Behrang Barekatain
Clemens Barends
Eric Barends
Yechezkel Barenholz
J. P. Barfield
Kenneth Barfod
Lea Barfod
Sarah Barga
Michael Barger
Thereza Bargut
Debmalya Barh
Bradford Barham
Lawrence Barham
Ausaf Bari
Elia Bari
Sailen Barik
Saroj Barik
Lucio Barile
Fabio Barili
Luisa Barin
Emma Barinas-Mitchell
Zeren Baris
Serap Barış
Andrea Barison
María Julia Barisón
Bettina Baritsch-Fritz
Joseph Bariyanga
Christina Barja-Fidalgo
Sharma Barjesh Chander
Alice Barkan
Ariel Barkan
Sapha Barkati
Bridget Barker
Christopher Barker
James Barker
Jocelyn Barker
Jon Barker
Peter Barker
Melissa Barker-Haliski
Timothy Barkham
Ahmed Barkia
Amanda Barkley
Amanda Barkley-Levenson
Vassilis Barkoukis
Panos Barlas
Mario Barletta
Jonathan Barlow
Karen Barlow
Scott Barman
Ruanne Barnabas
Celine Barnadas
Cynthia Barnard
Emma Barnard
Marie Barnard
Shanis Barnard
Edyta Barnaś
Amber E. Barnato
Allison Barner
Andrew Barnes
Anna Barnes
David Barnes
Geoffrey Barnes
Hilary Barnes
Irene Barnes
Jarrod Barnes
Kimberly Barnes
Matthew Barnes

Sean Barnes
Tamsin Barnes
Adrian Barnett
Anthony Barnett
Cleveland Barnett
George Barnett
James Barnett
Lionel Barnett
Ross Barnett
Susan Barnett
Michael Barnett-Cowan
Anthony Barnhart
Scott Barnhart
Nicolas Barnich
Matthew Barnidge
Jonathan Barnoud
Sven Barnow
Lester Barnsley
Victor Barocas
Viachaslau Barodka
Andrew Baron
David Baron
Emily Baron
Frederic Baron
Jason Baron
Andrea Baroncelli
Damiano Baroncini
Simon Baron-Cohen
Mark Barone
Gianluca Baroni Svegliati
Katie Barott
Liron Bar-Peled
Christina Barr
Ian Barr
Martin Barr
Maureen Barr
Paul Barr
Stewart Barr
José Barrabés
Juan Barrada
Manoel Barral-Netto
Rodolphe Barrangou
Francisco Barrantes
Jacques Barrat
Jonathan Barratt
Paul Barratt
Verónica Barraza
D. Barre
Frederick Barreau
Ivan Barreda
Esther Barreiro
Josefina Barrera-Cortes
Emiliano Barreto
Fellype Barreto
Goncalo Barreto
Andre Barreto-Vianna
Angela Barrett
Barbara Barrett
Craig Barrett
Maeve Barrett
Neville Barrett
Rod Barrett
Ted Barrett
Jeffrey Barrick
Antoni Barrientos
Rafael Barrientos
Guillermina Barril Cuadrado
Roberto Barrio
Paula Barrionuevo
Arantza Barrios
Clara Barrios
Maite Barrios
Rocío Barrios
Noelia Barrios Garcia
Alfred Barritt
Lisa Barroilhet
Andrew Barron
Anthony Barron
Susan Barron
Christina Barron-Ortiz
Ceres Barros
Diogo Barros
Francisco Barros
Lillian Barros
Maria Paloma Barros
Marlene Barros
Judit Barroso
Leticia Barroso

Renato Barroso
Ubirajara Barroso
Paul Barrow
George Barrowclough
Romina Barrozo
Vanessa Barrs
Amanda Barry
Cornelius Barry
David Barry
Johanna Barry
Peter Barry
Robert Barry
Sarah Barry
Sean Barry
Jessica Barson
Yakov Bart
Jiri Bartek
Frank Bartel
Sabine Bartel
Tim Bartels
Shannon Bartelt-Hunt
Alison Barth
Beatrix Barth
Holger Barth
James Barth
Luisa Barthauer
Dorothy Barthelemy
Marc Barthelemy
Lyric Bartholomay
Marlise Ladvocat Bartholomei-Santos
John B Bartholomew
Sharon Bartholomew-Began
Melissa Bartick
Suzanne Bartle-Haring
Ed Bartlett
Harrison Bartlett
Sofia R. Bartlett
Laura Bartley
Colleen Bartman
Thomas Bartnikas
Erzsebet Bartolak-Suki
Claudia Bartoli
Manuela Bartoli
Barbara Bartolini
Valerio Bartolino
Ramon Bartolo
Jordi Bartolomé
Al Bartolucci
Andrew Barton
Hazel Barton
John P Barton
Dariusz Bartosik
Rafal Bartoszewski
Eva Bartova
Mandy Bartsch
Ronny Bartsch
Hammurabi Bartuma
Paramita Baruah
Paolo Barucca
Celaleddin Barutçular
Elaine Baruwa
Melanie Barwick
Martin Barwood
Claudia Barz
Eran Barzilay
Joshua Barzilay
Luisa Barzon
Biraj Basak
Randall Basaraba
B. Basavanagoud
Corey Basch
Erin Baschal
Christiane Baschien
Leonardo Basco
Mirko Basen
Susan Baserga
Ethan Bash
Margaret Bash
Mohammad Bashaar
Davood Bashash
Meredith Bashaw
Tom Bashford
Mohammed Bashir
Azadeh Bashiri
Pavel Bashtrykov
David Bashwiner
Cecilia Basiglio
Carlo Basile

Franco Basile
John Basile
Pierpaolo Basile
G. Basini
Sulman Basit
Vaseeharan Baskaralingam
Harihara Baskaran
Katja Bäsler
Bhoja Basnet
Rahul Basole
Bryce Basques
Marco Bassani
Dave Bassett
Emma Bassett
Abhinav Bassi
Daniela Bassi
Katia Bassichetto
Seraina Bassin
Christine Bassis
Dirk Bassler
Bruno Basso
Daniela Basso
Daniele Basso
Stefano Basso
Anna Bassols
Alexander Bassuk
Agnieszka Basta-Kaim
Lisa Bastarache
Francois Bastardie
Deepak Bastia
Jojanneke Bastiaansen
Anna Bastian
Boris Bastian
Stefano Bastianini
Armanda Bastos
Francisco Bastos
João Bastos
Reginaldo Bastos
Alakananda Basu
Bhakti Basu
Hirak Basu
Raj Basu
Sandip Basu
Udita Basu
Robindra Basu Roy
Ken Batai
Ibrahim Batal
Eric Batchelor
Hannah Batchelor
Brian Bateman
Ted Bateman
Michael Bates
Sara Bates
Deborah Bateson
Girish Bathla
Erik Bathoorn
Anwar Batieha
Mona Batish
Antonio Batista
Miguel Batista
Wagner Batista
Sofia Batista Leite
Jacqueline Batley
Daniel Batlle
Javier Batlle
Hitesh Batra
Payal Batra
John Batsis
Sebastiano Battaglia
Vincenza Battaglia
Robert Battat
Sylvain Battault
Barbara Battelle
Sonia Batten
Phil Batterham
Giovanna Battipaglia
John Battista
Joeseph Battistelli
Andrea Battisti
Antonio Battisti
Corrado Battisti
James Battisti
Luca Battistini
Federico Battiston
Cynthia Battle
Michele Battle
Neha Batura
Florent Baty

Anthony Baucum
Olivier Baud
Amandine Baude
Sophie Baudic
Kaitlin Baudier
Veronique Baudin-Creuza
Christophe Baudouin
Antoine Baudrimont
Amy Bauer
Anna Bauer
Jan Bauer
José Roberto de Oliveira Bauer
Katherine Bauer
Katrin Bauer
Natalie Bauer
Raymond Bauer
Thomas Bauer
Wolfgang Bauer
S. K. Bauerjee
Lee Baugh
Sebastian Bauhoff
Heiko Bauke
Shawn Bauldry
Graham Baum
Jamie Baum
Marianna Baum
Martina Baum
Michel Baum
Sarah Baum
Grzegorz Bauman
Barbara Baumann
Hannes Baumann
Hendrik Baumann
Nicola Baumann
Tobias Baumann
Oliver Bäumchen
Philipp Baumeister
Luis Baumela
Ben Baumer
Florian Baumgart
Julia Kathrin Baumgart
Christina Baumgartner
Andreas Baumler
Iliana Baums
Heike Baumuller
Jeffery Baur
Cristina Baus
Nikolaus Bausch-Becker
I. Baussano
Christophe Bauters
Christian Bautista
Henry Bautista-Amorocho
Matthias Bauwens
Laura Bavagnoli
Sina Bavari
Giorgio Bavestrello
Edith Bavin
Ryan Bavis
Pradyuamna Baviskar
Rajesh Bawa
Raman Baweja
Amy Baxter
Bryan Baxter
David Baxter
Nancy Baxter
Nielson Baxter
Nicolas Bayan
Mohammad Bayat
Kaleab Baye
Andreas Bayer
Angela Bayer
Barbara Bayer
Monika Bayer
Otmar Bayer
Till Bayer
Wibke Bayer
Antoni Bayes-Genis
R. Bayford
Anne Bay-Jensen
Adil Baykasoglu
Sandrine Bayle
M. M. Bayliak
Alastair Baylis
Christopher Bayliss
Richard Bayliss
Recep Bayraktar
Elisa Bayraktarov
Hasan Bayram

Paul Bays
Melissa Baysari
Sultan Baytas
Haydee Bazan
Jose Bazan
Fuller Bazer
Sergei Bazhan
Dmitry Bazhanov
Nadia Bazihizina
Christopher Bazinet
Lauren Bazinet
Joel Bazira
Babak Bazrgari
Ivonne Bazwinsky-Wutschke
Claudia Bazzini
Chiara Bazzocchi
Joanna Beachy
Gordon Beakes
Jacob Beal
Ty Beal
Meghan Beale
Christopher Beam
Nancy Beam
Gillian Beamer
Thomas Bean
Rachel Louise Beanland
Vanessa Beanland
Adam Bear
Daniel Beard
Jessica Beard
John Beardall
David Bearden
Scott Beardsley
Gonzalo Bearman
Mj Beasley
Marco Beato
Alan Beaton
Andrea Beaton
Abi Beatson
Bradley Beattie
Michael Beattie
Alexis Beatty
Julia Beatty
Tsevi Beatus
Terri Beaty
David Beauchamp
Gary Beauchamp
Guy Beauchamp
Jonathan Beauchamp
Mark Beauchamp
Patrick Beauchesne
Charlotte Beaudart
Anne Beaudreau
Laurent Beauguitte
Michael Beaulieu
Bruno Beaumelle
Coreen Beaumier
Julia Beaumont
Marc Beaumont
Martin Beaumont
Robin Beaumont
Marie Beauséjour
Audrey Beaussart
Martyn Beaven
Thomas Beaver
Cecile Bebear
Penelope Bebeli
Lisa Bebell
Ilia Beberashvili
Inout Bebu
Francisco Beca
Laia Bécares
Hernán Beccacece
Francesca Beccati
Andrea Becchetti
Luca Becchetti
Marco Beccuti
Alejandro Beceiro
Lorena Becerra-Valdivia
Bodil Bech
Martin Bech
Etelvino Bechara
Meagan Bechel
Peter Moritz Becher
Arnaud Béchet
Lars Bechmann
Michael Becich
Alan Beck

Allen Beck
Ben Beck
Kathryn Beck
Melinda Beck
Moriah Beck
Sara Beck
Wladimir Beck
Alistair Becker
Annette Becker
Anton Becker
Ben Becker
Christian Becker
Daniel Becker
Guilherme Becker
Ingebor Becker
Jörg Becker
Laren Becker
María Becker
Matt Becker
Peter Becker
Silke Becker
Stefan Becker
Susan Becker
Susanne Becker
Torben Becker
Walter Becker
Philipp Beckerle
Oliver M. Beckers
Ronald Beckett
Carla Beckham
Sarah Beckham
Tess Becking
Gloria L. Beckles
Danielle Beckman
Robert Beckman
Manfred Beckmann
Michael Beckmann
Signe Beck-Nielsen
Emilie Bedard
Michel Bedard
Simon Beddows
Gregory Bedecarrats
Tibor Bedekovics
Aurélie Bedel
Lynn Bedford
Edward Bednarczyk
Marek Bednarczyk
Robert Bednarczyk
Petr Bednarik
Nina Bednarsek
Barbara Bedogni
Miguel Bedoya Perez
Jeffrey Bedwell
Kristin Beebe
Stephen Beebe
John Beeching
Hans Beeckman
Aaron Beedle
Martine Beeftink
Peter Beek
Jeff Beeler
Karen Beemon
Ella Been
Jan-Willem M. Beenakker
David Beer
Meinrad Beer
Stephen Beer
Peter Beernink
Jonathan Beever
Christopher Beevers
Mohammad Asim Beg
Grace Begany
Gerrit Begemann
John Beggs
Cesare Beghi
Ettore Beghi
Paola Begini
David Begley
Tadhg Begley
Chiara Begliomini
Rodolfo Begue
Anton Béguin
David Begun
Joachim Behar
Francine Behar Cohen
Jaideep Behari
Sanjay Behari
Shahriar Behboudi

Iman Beheshti
Ali Behfarnia
Robert Behle
David Behm
Jan Behmann
Daniel Behme
Cecilia Behn
Sascha Behnk
Ali Behnood
Oliver Behr
Rudiger Behr
Spencer Behr
Hamid Behravan
Dominik Behrendt
Donald Behringer
Roozbeh Behroozmand
Frank Beier
John Beier
Claudia Beiersmann
Sima Beigoli
Hamid Beigy
Niek Beijerink
Robert Beiko
Traude Beilharz
Gregory Beilman
Peter beim Graben
Hannes Beims
Ina Beintner
Ricardo Beiras
Marc Beirer
Tim Beißbarth
Greg Beitel
Jeremy R. Beitler
Elie Beit-Yannai
Donald Beitz
Eric Beitz
Asim Bej
Alexandre Bejanin
Bram Bekaert
Teshome Megersa Bekele
Isabelle Bekeredjian-Ding
Sander Bekeschus
Jolita Bekhof
Hesna Bektas
Coraline Bel
Carmen Belacchi
Vikas Belamkar
Ingrid Belan
Emmanuelle Belanger
Myriam Belanger
Salwa Belaqziz
Ariel Belasen
Pablo Belaunzarán Zamudio
Mulugeta Belay
Tesfaye Belay
Maria Belcastro
Justin Belcher
Ricardo Beldade
Detlev Belder
Laurent Bélec
Delia Belelli
Maria Belén Herrero
Leo Beletsky
Manfred Beleut
Inna Belfer
Antonino Belfiore
Michael Belfort
Akram Belghith
Safya Belghith
Liliana Belgioia
Danielle Belgrave
Eleni Beli
Petra Beli
Dominique Belin
Frida Belinky
Marc Bélisle
Boris Belitsky
Murat Belivermiş
Jose Belizario
Adrian Bell
Andrew Bell
Brian Bell
Damon Bell
Elizabeth Bell
Jason Bell
Jerold Bell
Joshua Bell
Kevin Bell

Nathaniel Bell
Nina Bell
Richard Bell
Scott Bell
Sue Bell
Terrence Bell
Trevor Bell
Felipe Belladonna
Bryan Bellaire
Nacer Bellaloui
Francesco Bellanti
Juan Bellas
Antonio Bellasi
Andrea Bellavia
Pierre Simon Bellaye
Riccardo Bellazzi
Ashwin Belle
Andrea Bellelli
Stefano Bellentani
Clare Bellhouse
Chiara Bellia
Julie Belliere
Melena Bellin
Michelle Bellingham
Peter Bellingham
Jennifer Bellis
France Bellisle
Alexandre Bello
Giambattista Bello
Gonzalo Bello
Nicholas Bello
Gianni Bellocchi
Vivian Bellofatto
Nicola Bellomo
Elena Belloni
Stefano Bellosta
Patrice Bellot
Abdelouahab Bellou
Benjamin Bellows
Luis Belo
Vinícius Belo
Alexandre Belot
Travis Belote
Apostolos Beloukas
Vsevolod Belousov
Denise Belsham
E. F. Belshe
Daniel Belsky
Daniel Belstrøm
Suzanne Belton
Jerzy Beltowski
Anna Beltrame
John Beltrame
Carlo Alberto Beltrami
Fernando Beltrami
Hiram Beltran-Sanchez
Barbara Beltz
Rhonda Belue
Clara Belzer
Véronique Belzil
Luc Belzunces
Amin Bemanian
Michael Bemben
Bill Bement
Saoussen Ben Abdallah
Anis Ben Ammar
Abdelkarim Ben Ayed
Moufida Ben Nasr
Bruria Ben Zeev
María Julia Bena
Laura Benaglia
Mohamed Benahmed
Said Benaissa
Omar Benali
Gil Benard
Philippe Benaroch
Noam Ben-Asher
Barbara Benassi
B. B. Benatti
Claudia Benavente
Andres Benavides-Serralde
Berenice Benayoun
Stefano Benazzi
Andrea Benazzo
Jochanan Benbassat
Mark Benbow
Nanette Benbow
Massimiliano Bencardino

Eric Benchimol
Matthew Bendall
Pavan Bendapudi
Eliel Ben-David
Uri Ben-David
Dirk Bender
G. Bender
Max Ernst Bender
Morgan Bender
Manon Benders
Felipe Bendezu
Lusiane Bendhack
Juliane Bendig
David Benditt
Casper Bendixsen
Caterina Bendotti
Iddo Ben-Dov
György Benedek
Andrea Benedetti
Celso Benedetti
Yanina Benedetti
Lisandro Benedetti-Cecchi
Stefano Benedini
Luciano Beneduce
Giovanni Benelli
Fabian Benencia
Itzhak Benenson
Ayse Bener
Roberta Benetti
Stefania Benetti
Rafael Benevenuto
Miriam Benezra
Valentina Benfenati
Nerys Benfield
Russell Benford
Annah Bengesai
Angela Bengtson
Ewert Bengtsson
David Benhaim
Avi Ben-Haroush
Shomron Ben-Horin
Ariela Benigni
Matthew Benigni
Jean-Pierre Benitah
Ana Benítez-López
Javier Benítez-Porres
Jon Benito
Pedro Benito
Julián Benito-León
Ivor Benjamin
Laura Benjamin
Longo-Mbenza Benjamin
Robert Benjamin
Benjamin Benjamin Ridenhour
Soumaya Benjelloun
Peter Benn
Jada Benn Torres
Christopher Benner
Ina Benner
Devasier Bennet
Abigail Bennett
Alan Bennett
Alex Bennett
Alison Bennett
C. Bennett
Christopher Bennett
David Bennett
Georgina Bennett
Jabbar Bennett
Jeff Bennett
John E. Bennett
Jonathan Bennett
Lauren Bennett
Rebecca Bennett
Robert Bennett
James Bennett Jr.
Rachel Bennetts
Jörn Bennewitz
Marion Bennie
Felix Benninger
Abby Benninghoff
Edwin Bennink
Sirish Bennuri
Sasisekhar Bennuru
Harvinder Bennypaul
David Benoist
Gérard Benoit
Gillet Benoit

Joshua Benoit
Marie-Odile Benoit-Biancamano
Ethan Benore
Anna Benrick
Moustafa Bensafi
Mohammed Bensellam
Rotem Ben-Shachar
Anabela Bensimon-Brito
Sandra Bensmihen
Alex Benson
Dudley Benson
Eric Benson
Frew Benson
John Benson
M. Benson
Paul Benson
Thomas Benson
Valerie Benson
Jonathan Benstead
Judith Benstein
Smadar Ben-Tabou de-Leon
Adam Bentham
James Bentham
Alison Bentley
David Bentley
Margaret Bentley
David Benton
Lorna Benton
Matthew Benton
Michael Benton
Mariana Bentosela
Yaakov Bentov
David Ben-Tovim
David Bentrem
Leonie Bentsink
Francesca Benuzzi
Amine Benyamina
J. Philipp Benz
Recep Benzer
Karen Benzies
Anat Ben-Zvi
Haim Ben-Zvi
Anna Concetta Berardi
Emanuele Berardi
Francesco Berardinelli
Marika Berchicci
Aikaterini Berdiaki
Yevgeny Berdichevsky
Tristan Bereau
Yoella Bereby-Meyer
Ludek Berec
Janneke Berecki-Gisolf
Bradley Bereitschaft
Michael Bereman
Michael Berenbrink
Evelien Berends
Bettina Berendt
Michael Berens
Simone Beretta
Alexander Berezin
Anders Berg
Cynthia A. Berg
Daniela Berg
Lisa Berg
Mark Berg
Stefan Berg
Alessandro Bergamasco
Marco Bergamin
Elena Bergamini
Alberto Bergareche Yarza
Eivind Berge
Laurent Berge
Lillian Berge
Sara Bergek
Corinna Bergelt
Daniel Bergemalm
Arthur Bergen
Werner Bergen
Adam Berger
Bettina Berger
Elizabeth Berger
Helmut Berger
Hester Berger
Joel Berger
Miles Berger
Monique Berger
Sebastian Berger
Sergio Bergese

Ron Bergevoet
Ruth Berggren
J. Bergh
Ina Bergheim
Vincenzo Berghella
Andrea Berghold
Mathilde Berghout
Lars Berglund
Aviv Bergman
Brandon Bergman
Elizabeth Bergman
Thore Bergman
Christina Bergmann
Georg Bergmann
Jan Bergmann
Philipp Bergmann
Ralf Bergmann
Til Ole Bergmann
Tjard Bergmann
Ulrich Bergmann
Felipe Bergo
Audrey Bergouignan
Jean Bergounioux
Joel Bergqvist
Brock Bergseth
Niels Bergsland
Ted Bergstrom
Ulf Bergstrom
M. Berin
Ioana Berindan-Neagoe
Stine Bering
Andre Beringhs
Carolina Berini
Edmond Berisha
Visar Berisha
Alexander Beristain
Bradford Berk
Sara Berkelhamer
Ben Berkhout
James Berkley
Bruce Berkowitz
Natacha Berkowitz
Samuel Berkowitz
Seth Berkowitz
Tomas Berl
Keith Berland
Juan Berlanga
Anna Berlin
Cecilia Berlin
Jordan Berlin
Léo Berline
Lawrence Berliner
Fiammetta Berlinguer
Ariel Berlinski
Daniel Berlowitz
David Berlowitz
Gordon Berman
Nicolas Berman
Reena Berman
Jesus F. Bermejo-Martin
Emma Bermingham
Olga Bermudez
José-María Bermúdez de Castro
Carl Bern
Antonio Bernabe-Ortiz
Carmelo Bernabeu
César Bernal
Daniel Bernard
David Bernard
Edwin J. Bernard
Enrico Bernard
Jessica Bernard
Karen Bernard
Kathryn Bernard
Kristin Bernard
Philippe Bernard
Quentin Bernard
Stephen Bernard
Virginie Bernard
Luciano Bernardi
Stella Bernardi
Gabriele Bernardini
Nunzia Bernardini
José Ignacio Bernardino
Nathalie Bernard-Marissal
Antonio Bernardo
Umberto Bernardo
Alessio Bernasconi

Eta Berner
Logan Berner
Stefan Bernet
Peter Berney
Frank Bernhard
Christopher Bernhardt
Stephan Bernhart
Eric Bernicker
Nicholas Bernier
Steve Bernier
Stine Bernitz
David Bernlohr
Daniel Bernoulli
Gregory Berns
Aaron Bernstein
Joshua Bernstein
Kyle Bernstein
Paul Bernstein
Steven Bernstein
Wolfram Bernstorff
Erik Berntorp
Mb Berntsen
Sveinung Berntsen
Edurne Berra
Stefano Berretti
Eduardo Berriatua
Kent Berridge
Leigh Berrie
Sonia Berrih-Aknin
Federica Berrilli
Carmen Berrocal
Brad Berron
Catherine Berry
Don Berry
Elaine Berry
Hugues Berry
Marla Berry
Meredith Berry
Peter Berry
William R. Berry
Wim Bert
Graziella Berta
Assunta Bertaccini
Anthony Bertagnolli
Marco Bertamini
Federica Bertaso
Cinzia Bertea
Sven Bertel
Randi Bertelsen
Kimberly Bertens
Greg Bertenshaw
Laureline Berthelot
Nicolas Berthelot
Francois Berthiaume
Celine Berthier
Franz Berthiller
Viviana Berthoud
Francois Berthoux
Federico Berti
Stefan Berti
Fabrice Bertile
Stefan Bertilsson
Benjamin Bertin
Gwladys Bertin
Robert Bertin
Caterina Bertini
Farryl Bertmann
Mark Bertness
Eric Bertoft
Laura Bertola
Luca Bertolaccini
Silvano Bertoldo
A. Bertoletti
Giulia Bertolin
Giovanni Bertolini
Giulia Bertolini
Marta Bertolini
Martinna Bertolini
Maurizio Bertollo
Giuseppe Bertoni
Martino Bertoni
Amelia Bertozzi-Villa
John Bertram
Lars Bertram
Raymond Bertram
Alexander Bertrams
Daniel Bertrand
Sophie Bertrand

William Bertrand
Frédéric Bertucci
David Berube
Nicolas Bérubé
Jaime Berumen
Daniel Berwick
Marianne Berwick
Tiffany Berzins
Frank Besag
Julie Besco
Bruno Besen
Shadi Beshai
Samuel Beshers
Thor Besier
Cagri Besirli
John Besley
Antoine Besnard
Yulia Bespalaya
Filipa Bessa
Lucinda Bessa
Florence Besse
Cecilia F. Bessega
Debra Bessen
Marc Besson
Pierre Besson
Pascal Bessong
Aaron Best
Al Best
Conor Best
John Best
Katharine Best
Lawrence Best
Maisy Best
Michael Best
Robert Best
Victoria Best
Sébastien Besteiro
Dirk Bester
Maxim Bester
Anne-Kathrin Bestgen
Timothy Bestor
Carsten Beta
Swati Betharia
Christina Bethell
Kathleen Bethin
Paul Bethke
Andrew Betik
Michiel Betjes
Esther Betran
Ana Pilar Betrán
Simret Betsha
Kirsten Bett
Stefano Bettati
Conceição Bettencourt
Lucien Bettendorff
Jan Bettgenhaeuser
Joseph Betthauser
Federico Betti
Pete Bettinger
Alessandra Bettiol
Judy Bettridge
Dean Betts
James Betts
K. Betts
Christian Betz
Marian Betz
Timo Betz
Jean-Baptiste Beuscart
Rolf Georg Beutel
Sascha Beutel
Annie Beuve
Carmen Beuzón
David Bevan
Jennifer Bevan
Rebecca Bevans
Laure Beven
David Beversdorf
Catherine Bevier
Antonio Bevilacqua
Carole Bewley
Rudi Beyaert
May Beydoun
Martin Beye
Tafese Beyene
Andreas Beyer
Andreas Beyerlein
Elisabeth Beyersmann
Tahsin Beyzadeoglu

Ondrej Bezdicek
Jacob Bezemer
Gustavo Bezerra
Marcelo Bezerra
Rosângela Maria Neves Bezerra
Dmitri Bezinover
Katerina Bezrukova
Michael Bezuhly
Tunira Bhadauria
Jehangir Bhadha
Anirban Bhaduri
Sumita Bhaduri-McIntosh
Punyasloke Bhadury
Akshaya Bhagavathula
Ashok Bhagwat
Mehul Bhakta
Tek Bhalla
Deevia Bhana
Dinesh Bhandari
Rashna Bhandari
Sanjay Bhandari
Tulsi Bhandari
Vineet Bhandari
Ashwin Bhandiwad
Syon Bhanot
V. Bhanuprakash
Lalita Bharadwaj
Mausumi Bharadwaj
Shrikant Bharadwaj
Pandurangan Bharathi
Anshu Bhardwaj
Neeru Bhardwaj
Rukmini Bhardwaj
Aditi Bhargava
Ajay Bharti
Deepa Bhartiya
Kiran Bhaskar
Sangeeta Bhaskar
Nilesh Bhaskarrao Bahadure
Subha Bhassu
Amritha Bhat
Manzoor Bhat
R. Bhat
Uttam Bhat
Vikrant Bhateja
Pooja Bhati
Anuj Bhatia
Arti Bhatia
Haresh Bhatia
Madhav Bhatia
Sudeep Bhatia
Satish Bhatla
Aarushi Bhatnagar
Amit Bhatnagar
Julu Bhatnagar
Rakesh Bhatnagar
Vipul Bhatnagar
Pavan Bhatraju
Ami Bhatt
Jay Bhatt
Tanvi Bhatt
Gopal Bhatta
Raghavendra Bhatta
Ananda Bhattacharjee
Ashish Bhattacharjee
Madhuchhanda Bhattacharjee
Niloy Bhattacharjee
Sandipan Bhattacharjee
Sonali Bhattacharjee
Debswapna Bhattacharya
Joydeep Bhattacharya
Parna Bhattacharya
Satya Bhattacharya
Sohinee Bhattacharya
Sudeepto Bhattacharya
Sunanda Bhattacharya
Syamal Bhattacharya
Dhananjay Bhattacharyya
Indraneel Bhattacharyya
Krishnendu Bhattacharyya
Mrinal Bhattacharyya
Samit Bhattacharyya
Santanu Bhattacharyya
Suvendra Bhattacharyya
Nirjal Bhattarai
Nishan Bhattarai
Jasvinder Bhatti
Junaid Bhatti

M. M. Bhatti
Sajjad Haider Bhatti
Yosef Bhatti
Ashay Bhatwadekar
Siddhartha Bhaumik
Penta Bhavanadhar
Rozina Bhimani
Ravinay Bhindi
Pervinder Bhogal
Upendra Bhojani
Pranav Bhounsule
Sanjukta Bhowmick
Sankha Bhowmick
Dipankar Bhowmik
Tanmay Bhowmilk
Alauddin Bhuiyan
Muhammed Bhuiyan
Arun Bhunia
Siddhi Bhusal
Nidhi Bhutani
Mahmood Bhutta
Chuan-Xing Bi
Chun Bi
Gongbing Bi
Hongtao Bi
Rui Bi
Rutian Bi
Tinai Bi
Xin Bi
Yan Bi
Yanhong Bi
Ye Bi
Yuhai Bi
Sibhatu Biadgiligne
Giacomo Biagi
Taher Biala
Slawomir Bialek
Michał Białek
Michal Bialy
Chao Bian
Desong Bian
Hui-Jie Bian
Jiang Bian
Jifeng Bian
Liming Bian
Weixin Bian
Yiwen Bian
Zhaoxiang Bian
Carlo Bianca
Enrica Bianchi
Francesca Antonella Bianchi
Francesca Bianchi
Giacomo Bianchi
Matt Bianchi
Silvia Bianchi
Valerio Bianchi
Bianca Bianco
Pasquale Bianco
Piero Bianco
Rohan Bianco
Michela Biancolella
Luigi Biancone
Giovanni Bianucci
Alexandre Biasi
Emiliano Biasini
Silvia Biasotti
Nadia Biassou
Kyle Bibby
Thomas Bibby
Peter Biberthaler
A. Ozan Bicen
David Bichell
Kai Bickenbach
Mark Bicket
Paula Bickford
Penny Bickle
Myriam Bickle Graz
Stephen Bickler
Mehdi Bida
Jean-Pierre Bidanel
Mallikarjun Bidarimath
Keshore Bidasee
Owen R. Bidder
Chuck Biddle
Stuart Biddle
Leticia Bidegaray-Batista
Edmund Biden
Dennis Bideshi

Felicitas Bidlack
Martin Bidlingmaier
Jean Bidlot
Pernille Bidstrup
Joseph Bidwell
Tongde Bie
Yiming Bie
Karen Bieback
Lori Biederman
Jossi Biedermann
Wioletta Biel
Lawrence Bielak
Bernhard Bielesz
Katie Biello
Maximilian Bielohuby
Leonard Bielory
Godfrey Biemba
Christian G. Bien
François Bienvenu
D. Bienzle
David Bierbach
G. Bierbaum
Gabriele Bierbaum
Loic Biere
S. Beth Bierer
Wouter Bierman
Daniel Biermann
Lauren Biermann
Joanna Biernacka
Bernhard Biersack
Karolina Bierza
Jan Bieschke
Barbara Biesecker
Jessica Biesiekierski
Franck Biet
Andreas Bietenbeck
Angelo Bifone
Peggy Biga
Elisabetta Bigagli
Shoaleh Bigdeli
Ann Bigelow
Daniel Bigelow
James Bigelow
Kimberly Bigelow
Patrick Biggar
Manus Biggs
Trent Biggs
Erin Bigler
G. Biglino
Giovanni Biglino
Lavi Bigman
Elena Bignami
Darell Bigner
Sarah Bigot
Rosemarie Bigsby
Emmanuel Bigwan
Helene Bihan
Shailesh Bihari
Nooshin Bijari
Janetta Bijl
Allert Bijleveld
Rob Bijlsma
Boris Bikbov
Aida Bikic
Rama Kamesh Bikkavilli
Benjamin T. Bikman
Ali Bikmoradi
Marom Bikson
Usama Bilal
Antonio Bilancio
Jade Bilardi
Katia Bilardo
Vedran Bilas
Kenneth Bilchick
Anton J. Bilchik
Gust Bilcke
Trine Bilde
Phillip Bilderback
Natasha Bilenko
Berkin Bilgic
Kerstin Bilgmann
Stephanie Bilinovich
Alyssa Bilinski
Moses T. Bility
Filip Biljecki
Andras Bilkei-Gorzo
Baki Billah
Elisabeth Billard

Matthew Billard
Veronique Billat
Marie Billaud
Jose Biller
Henny Billett
John Billimek
Sandra Billinger
Curtis Billings
Jenny Billings
Jutta Billino
Helen Billman-Jacobe
Grzegorz Bilo
Elizabeth Bilodeau
Guillaume Bilodeau
Federico Bilotta
Johan Bilsen
Anna Bilska-Kos
Benjamin Bimber
Luis Bimbo
Diane Bimczok
Christian Bime
Li Bin
Wang Binbin
Paola Binda
Markus Bindemann
Christoph Binder
Henry Binder
Marco Binder
Martin Binder
Prem Bindrban
Laurence Bindschedler
Saskia Bindschedler
Giorgio Binelli
Mario Binelli
Isabelle Binet
C. Raymond Bingham
Roland Bingisser
Rodrigo Bini
Aristea Binia
Łukasz Binkowski
Martin Binks
Richard Binney
Sandra Binning
Tony Binns
Mickael Binois
Gianni Biolo
Massimo Bionaz
Antonio Biondi
Emanuele Biondi
Stefania Biondi
Giuseppe Biondi-Zoccai
Alessio Emanuele Biondo
V. Biousse
Burak Bircan
Colin Birch
Joanna Birch
Karen Birch
Rachael Birch
Martin Birchall
James Birchler
Adam Bird
Broxton Bird
Christopher Bird
Marie-Louise Bird
Michael Bird
Nathan Bird
Raymond Birge
Ruthie Birger
Zewdie Birhanu
Nana-Kwadwo Biritwum
Matthew Birk
Max Birk
Marianne Birkeland
Sarah Birken
Peter Birkholz
Christoph Birkl
Lauren Birks
Serge Birman
Trevor Birmingham
Henrik Birn
Angela Birnbaum
Yochai Birnbaum
Ruth Birner-Gruenberger
Ewan Birney
David Birnkrant
Eva Biro
Christine Biron
David Biron

L. Birsa
Catherine M. Birt
P. S. Birthal
Andrew Birtles
Mulatu Biru
Ingvars Birznieks
Filippo Biscarini
Claudio Biscaro
Heather Bischel
Sonja Bisch-Knaden
Jason Bischof
Martin Bischof
Alexander Bischoff
Sebastian Bischoff
Maria Assunta Biscotti
Alexandre Bisdorff
Ryan S. Bisel
David Bishai
Christopher Bishop
Corey Bishop
Erin Bishop
Jacob Bishop
Kristina Bishop
Martin Bishop
Kimberly Bishop-Lily
Patrizia Bisiacchi
Till Biskup
James Bisley
Marco Bisoffi
Tiziana Bisogno
Stephanie Bispo
Bernie Bissett
Linda Bisson
John Bissonette
Annie Bissonnette
Damber Bista
Jitendra Biswal
Manas Biswal
Shyam Biswal
Abhishek Biswas
Aviroop Biswas
Manosh Biswas
Saswati Biswas
Sumalika Biswas
Craita Bita
Davide Bitetto
Tesera Bitew
Gulfidan Bitirgen
Elena Bitocchi
Giovanni Bittante
H. C. Bittenbender
Joerg Bittenbring
Patrick Bitterman
Leonora Bittleston
Eric Bittman
Stefan Bittner
Natalie Bitto
Mozhgan Bizhang
Jean-Pierre Bizimana
Ivana Bjedov
Vesna Bjegovic-Mikanovic
Andreas Bjerrum
Jan Bjordal
Mats Bjork
Ingemar Björkhem
Fredrik Björklund
Heidi Björklund
Geir Bjørklund
Maria Björkqvist
Johannes Björkstrand
Lars Bjørndal
Jacob Bjorner
Thea Bjørnland
Mikael Björnstedt
Trine Bjøro
Crysten Blaby-Haas
Yoann Blache
Jan Blacher
Pierre Blacher
Alex Black
Christopher Black
Dennis Black
Kirsten Black
Lauren Black
Marsha Black
Matt Black
Patrick Blackall
Daniel Blackburn

Jessica Blackburn
Carl Blackman
Leila Blackman
Heath Blackmon
Richard Blackmon
Murray Blackmore
Paul Blackmore
Craig Blackstone
Neil Blackstone
Emily Blackwell
Simon Blackwell
Susanna Blackwell
Christopher Blackwood
Sandra Blaess
Milisa Blagojevic-Bucknall
Roman Blaheta
Bonnie Blaimer
Cindy Blair
David Blair
G. Blair
Jaime Blair
Laura Blair
Caroline Blais
Lucie Blais
John Blake
Randolph Blake
Jaishri Blakeley
William Blakely
Rebekah L. Blakemore
Joshua Blakeslee
John Blakey
Susan Blalock
William Blalock
Sean Blamires
Etienne Blanc
Caroline Blanchard
Frederic Blanchard
Stéphane Blanche
Claudio Inostroza Blancheteau
Marc-André Blanchette
Robert Blanchette
Loïc Blanchon
Sylvain Blanchon
Anibal Blanco
Carlos Blanco
Fernando Blanco
José Ramón Blanco
Luis Blanco
Mariana Blanco
Leocadio Blanco-Bercial
Francisco Blanco-Vaca
Ruth Bland
James Blande
Stéphanie Blandin
Yannick Blandin
Giovanni Blandino
Manuel Blandino
Alison Blane
Jeff Blaney
Douglas Blank
Stephanie Blank
Ulf Blanke
Jill Blankenship
Kevin Blankenship
Terry Blankenship-Paris
Joel Blankson
Hart Blanton
Ronald Blasberg
Julia Blasch
Fernando Blasco
Hélène Blasco
Hilario Blasco-Fontecilla
Mark Blaser
Martin Blaser
Annalisa Blasetti
Annabel Blasi
Damian Blasi
Elisabetta Blasi
Emanuele Blasi
Janusz Blasiak
Gregory Blass
Janusz W. Blaszczyk
Sophie Blat
Keith Blatner
Christine Blattner
Frank Blattner
Sergio Blay
Douglas Blayney

Jane Blazeby
Olesya Blazhenkova
Ma Blazquez
Jesús Blázquez
Marie Bleakley
Leszek Błędzki
Margit Bleecker
Hannah Blencowe
Molly Bletz
Sophie Bleves
James Blevins
John Blevins
Jon Blevins
Nikolas Blevins
Audrey Blewer
Ronald Bleys
Janneke Blijlevens
Cecilie Hveding Blimark
Imrgard Blindow
Mikhail Blinikov
James Bliska
Joseph Bliss
Eliza Bliss-Moreau
Dana Bliuc
David Blizard
Evan Bloch
Mark Bloch
Eric Block
Geoffrey Block
Jörn Block
Marc Block
Martin Block
Robert Block
Stefanie Block
Ariel Blocker
Nichlolas Blockley
Heike Blockus
Elke Bloem
Oswald Bloemen
Jos Bloemers
Marcus Bloice
Andrea Bloise
Enrico Bloise
Daan Blok
David Blok
Lucie Blok
Harry Blokhuis
Arjan Blokland
Brittany Blomberg
Jonas Blomberg
Marie Blomberg
Rune Blomhoff
Jane Blood-Siegfried
Devin Bloom
Hanna Bloomfield
Susan Bloomfield
John Blosnich
Ralf Blossey
Hervé Blottiere
Dieter Blottner
Boris Blotto
Cedric Blouin
Stéphane Blouin
Yann Blouin
Paul Blount
Robert Blount
Michael Blower
David Bluemke
Burton Bluhm
Ian Blum
Jennifer Blum
Bruce Blumberg
Francine Blumberg
Herbert H. Blumberg
Margaret Blume-Kohout
Helena Blumen
Yair Blumenfeld
Justin Blumenstiel
Joshua Blumenstock
David Blumenthal
Paul Blumenthal
Robert Blumenthal
Alice Blumenthal-Dramé
Janell Blunt
Jan Blustein
Andreas Blutke
Gerold Blutman
Astrid Blystad

Dag-Ragnar Blystad
Richard Blythe
Michaela Blyton
Peter Boag
Adriel Boals
Peter Boan
Daniel Boateng
Godfred Boateng
Adeline Boatin
Timothy Boaz
Flaviu Bob
Anna K. Bobak
Marina Bobkova
Lyuba Bobova
Elzbieta Bobrowicz-Campos
Paulo Estefano Bobrowiec
Ladislav Bocak
Anna Boccaccio
Francesco Boccalatte
Enrico Bocci
Stefania Boccia
Thomas Bochaton
Natalia Bochkina
Aaron Bochner
Mandy Bochnia
Matthias Bochtler
Beth Bock
C. Thomas Bock
Naomi Bock
Marija Bockarjova
Detlef Bockenhauer
Maximilian Bockhorn
Alfred Böcking
Christopher Bockisch
Richard Bockman
Dirk Bockmuehl
Philippe Bocquier
Christian Bocti
Enrica Boda
Surendra Bodakhe
Dhananjay Bodas
Manish Bodas
Raúl Bodas
Jacques Boddaert
Xavier Boddaert
Crystal Boddie
Lynne Boddy
Michael Bode
Joseph Boden
Lisa Boden
Robert Boden
Jason Bodily
Johanna Bodin
Antonio Bodini
Ivan Bodis-Wollner
Charles Bodmer
Michele Bodmer
Rolf Bodmer
Lisa Bodnar
Richard Bodnar
Péter Bodnár
Donald Bodner
Mathilde Body-Malapel
Pauline Boeckrxstaens
Carsten Boehler
Alexandria Boehm
Amelia Boehme
Stefen Boehme
Kasey Boehmer
Jan Boehnke
Susan Boehnke
Hennie Boeije
Michael Boele van Hensbroek
Wilbert Boelens
T. Sonia Boender
Wouter Boendermaker
Helena Boene
Marianne Boeni-Schnetzler
Diana Boer
Joel Boerckel
Benjamin Boerebach
Patrick Boerlin
David Boerma
Marjan Boerma
Ties Boerma
Ferdinando Boero
Melanie Boerries
Philipp Boersch-Supan

Doeke Boersma
Bobby Boessenecker
Sanne Boesveldt
Ryan Boettger
David Boettiger
Paolo Boffano
Amy Bogaard
Mikhail Bogachev
Sergé Bogaerts
Alemtsehay Bogale
Arthur Bogan
Michael Bogan
Giorgio Bogani
Clifton Bogardus
Jim Bogart
Lara Bogart
Evgeny Bogdanov
Sophie H. Bogers
Angela Boggero
Ian Boggero
Henrik Bøggild
Nansi Boghossian
Nathaniel Bogie
Carsten Bogler
Luisa Bogliolo
Isaac Bogoch
Vanya Bogoeva
Julius Bogomolovas
Bjarte Bogstad
Igor Bogunovic
Milena Bogunovic
Petr Bogusch
Jaroslav Boháč
Johannes Bohacek
Richard Bohannon
Richard W. Bohannon
Jonathan Bohbot
Jason Bohland
Leah Bohle
Michael Böhm
Ruwen Böhm
Björn Bohman
Dirk Bohmann
Frank D. Böhmer
Andrea Bohn
Barbara Bohn
Erwin Bohn
Delwayne Bohnenstiehl
Marc Bohner
Kyle Bohnert
Cara Bohon
Iryna Bohovych
Abhishek Bohra
Ben Bohrer
M. Boiago
Charlotte Boichat
Adrien Boillot
Mirian Boim
I. F. S. F. Boin
Adrienne Boire
Matthieu Boisgontier
Marie-Christophe Boissier
Mariana Boité
Florence Boitrelle
Mark Boitt
Gregory Boivin
Xavier Boivin
Ester Boix
Jasmina Bojadzieva
Jasmine Bojadzieva
Maciej Bojanowski
Piotr Bojarski
Iva Bojic
Sanja Bojic
Suzana Bojic
Eddie Bokkers
Niklas Boknas
Alex Bokov
Michał Bola
Enrique Bolado-Martinez
Patrick Bolan
Carlos Bolanos
Wesley Bolch
Kata Bölcskei
Stefan Bolder
Paolo Boldrini
Rosa Bolea
Annemarie Boleij

Annette Boles
Ann Bolger
Fergus Bolger
Niall Bolger
Azam Bolhassani
Hanna Bolibok-Brągoszewska
Steve Bolin
Subhashini Bolisetty
Sema Bolkent
Kaatje Bollaerts
Mark J. Bolland
Kenneth Bollen
Bernardo Bollen Pinto
Daniel Bolliger
Bethany Bolling
Sveva Bollini
Paul Bollyky
Saverio Bolognani
Silverio Bolognani
Renata Bolognesi
Nadia Bolognini
Monique Bolotin-Fukuhara
Catherine Bolten
Doron Boltin
Mark Bolton
Matthias Bolz
Alejandro Bolzán
Hee-Seung Bom
Christoffer Boman
Aureliano Bombarely
Sébastien Bommart
Richard Bomphrey
Morgane Bomsel
Elisa Bona
Juan Bonachela
Fabrizia Bonacina
Carla Denise Bonan
Isabelle Bonan
Carl Bonander
Enrica Bonanni
Joseph Bonanno
Ian Marc Bonapace
Silvia Bonardi
Lawrence Bonassar
Gabriel Bonaterra
Mauricio Bonatto Machado de Castilhos
Rachael Bonawitz
Antonello Bonci
Gregor Bond
Keosha Bond
Malcolm Bond
Mark Bond
Michelle R. Bond
Morgan Bond
Charles Bond Jr.
Subbarao Bondada
Ana-Nicoleta Bondar
Jesper Bonde
Maria Bondesson
Daniel Bone
Ivo Boneca
Claudia Bonecker
Francesco Bonella
Andrea Bonetto
Billie Bonevski
Cristina Bonferoni
Silvia Bonfiglio
David J. Bonfil
Ricardo Bonfil
Brenda Bongaerts
Erik Bongcam-Rudloff
Anelí Bongers
Marlies Bongers
Massimo Bongiovanni
Corinna Bonhage
Andrew Bonham
Katia Boniface
Wes Bonifay
Leonardo Bonilha
Vera Bonilha
Carolina Bonilla
Keith Bonin
Mariangela Bonizzoni
Silvia Bonizzoni
Cory Bonn
Matthew Bonnan
Thomas Bonnard
Valerie Bonnardel

Xavier Bonnefont
Tyler Bonnell
Guusje Bonnema
Daniel Bonner
James Bonner
Timethia Bonner
Florian Bönner
Susan Bonner-Weir
Cédrick Bonnet
Muriel Bonnet
Pascal Bonnet
Udo Bonnet
Vincent Bonnet
Xavier Bonnet
Stephanie Bonney
Renzo Bonofiglio
Francesco Bonomi
Mario Bonomini
Adriana Bonomo
Robert Bonomo
Silvia Bonomo
Carmelo Bonsignore
Maria Bonsignore
Marc Bonten
Alexandre M. J. J. Bonvin
Ga Hun Boo
David Boocock
Basmattee Boodram
Linda Booij
Martijn Booij
Michael Book
Wayne Book
Cara L. Booker
Jordan Booker
Dorret Boomsma
Mei Ying Boon
Mieke Boon
Teck Boon Low
Jan Boone
John Boone
Peter Boone
Jelle Boonekamp
Chanchai Boonla
Jarungwit Boonnorat
Kansri Boonpragob
Wimonrut Boonsatean
Panadda Boonserm
Wijnand Boonstra
Cécile Boot
Ken Boote
Anthony Booth
James A. Booth
R. Booth
Tom Booth
Vernon Booth
Warren Booth
Sriram Boothalingam
Lynda Boothroyd
George Booz
Jean Chrisostome Bopassa
Stephen Boppart
Jacob Bor
Utpal Bora
Anupom Borah
Jonathan Borak
Debasish Boral
Christian Borch
Jost Borcherding
Glen Borchert
Sven Borchmann
Elizabeth Borda
Theda Borde
Kira Borden
Christophe Bordi
Juliano Bordignon
Michael Bordonaro
María Bordons
Julian Borejdo
Nicole Borel
Erika Borella
Bindi Borg
Bridget Borg
Ana Luiza Borges
Bárbara Borges
Boniek Borges
Celina Borges
Cibele Borges
Karin Borges

Paulo Borges
Vítor Borges
Márcio Borges-Martíns
Ana Cristina Borges-Oliveira
Jan Borggrefe
José Borghans
Alessandro Borghesi
Fabrizio Borghesi
Alberto Borghetti
Giulia Borghetti
Alessandro Borghi
Andrea Borghini
Herman Borghys
Marta Borgi
Jeffrey Borgia
Paola Borgiani
Alessandra Borgognone
Sara Borgomaneri
Stefan Borgwardt
Hossein Borhan
Giuseppe Boriani
Michael Borich
Andrea Borini
Angel Borja
Raúl Monroy Borja
Angelika Borkowetz
Jakub Borkowski
Kat Bormann
Myriam Bormans
Arnob Bormudoi
Yan Borné
Stephen Bornemann
Helmar Bornemann-Cimenti
Rosa Börner
Stephanie Bornes
Gudrun Bornette
Daniela Börnigen
Riana Bornman
Uwe Bornscheuer
Javier Borondo
Anton Borovjagin
Ilya Borovok
James Borowiec
Marek Borowiec
Avinoam Borowsky
Marco Borra
J. M. Borràs
Maria Borrego
Antoni Borrell
James Borrelli
Maria-Teresa Borrello
Stuart Borrett
Paola Borroni
Ray Borrow
Richard Borrows
Pascal Borry
Melinda Bors
Denny Borsboom
Karen Borschmann
Simone Borsci
Lubor Borsig
Aleksandra Bortel
Alip Borthakur
Grażyna Bortnowska
Marco Bortolato
Marta Bortoletto
Raul Bortolin
Eugenio Bortolini
Guilherme A. Bortolotto
Ieda Maria Bortolotto
Giacomo Bortolussi
Eric Bortz
Sergii Boryshpolets
Stanislav Borysov
Peter Borza
Mauro Borzio
Dienke Bos
Kristen Bos
Mettine Bos
Peter Bos
Lisardo Boscá
Astrid Bosch
Guido Bosch
Irene Bosch
Linda Bosch
Pablo Bosch
Thijs Bosch
Bert Bosche

Cecile Boscher
Clarissa Boschiero
Lynn Boschloo
Francesca Boscia
Andrea Bosco
Annalisa Bosco
Francesca Bosco
Giovanni Bosco
Anindya Bose
Indranil Bose
Sutapa Bose
Tanay Bose
Tanima Bose
Michal Bosela
Emmanuel Boselli
Steven Bosinger
Catharine Bosio
Michael Boska
Maarten Bosland
Jean Louis Bosmans
Judith Bosmans
Emanuel Boss
Wendy Boss
Matthias Bossard
Katharine Bossart
Robert Bossarte
Janine Bosse
Patrick Bosshart
Torsten Bossing
Gabriel T. Bosslet
Xavier Bossuyt
Jenny Bosten
Bruce Bostrom
Katrina Bosward
Steven Bosworth
Issac Botche
Héctor Botella
Francisco Botella Robles
Anna Both
Graham Bothamley
Jeffrey R. Botkin Botkin
Nikolai Botkin
Elena Boto
Marjorie Bott
Cristian Botta
Federico Botta
Luca Botta
Roberto Botta
Francesca Bottacini
Daniele Bottai
Chotima Böttcher
Eva Böttcher-Friebertshäuser
Daniela Bottero
Daniele Botticelli
Cristina Bottino
Tomaso Bottio
Walter Bottje
Christian Bottomley
Lindsay Bottoms
Kai Bötzel
Magda Bou Dagher-Kharrat
Rami Bou Khalil
George Bou-Assaf
Nabila Bouatia-Naji
Hasna Boubakri
Laura Boucai
Jorge Boucas
Sheena Bouch
Josee Bouchard
Philippe Bouchard
Nicole Boucher
Audrey Bouchet
Didier Bouchon
Guillaume Bouchoux
Drion Boucias
Emmanuel Boucrot
Arnaud Boudenot
Frédéric Boudon
Jeanette Boudreau
Pierre Boudry
Kris Boudt
Michel Boufadel
Roland Bouffanais
Carole Bougault
Alexandre Bougdour
Gaelle Bougeard
Sabri Boughorbel
Dumith Bou-Habib

Eric Bouhassira
Sotiria Boukouvala
Ana Boulanger
Jason Boulanger
Mathieu Boulanger
Steeve Boulant
Olivier Boulard
Yves Boulard
Erin Bouldin
Elizabeth Boulding
Carole Bouleuc
Richard Bouley
Areti Boulieri
Thomas Boulin
Daniel Boullosa
Christa Boulos
G. Boulouis
Etienne Boulter
Sarah Boulton
Michel Boulvain
Yap Boum II
Mark-Bram Bouman
Kyria Boundy-Mills
Abdenour Bounihi
Vasiliki Bountziouka
Jerome Bouquet
Lo Bour
Kyle Bourassa
Mark Bourassa
Aïda Bouratbine
Nikolaos Bourbakis
Mathieu Bourbonnais
Amelia Bourceret
Romain Bourcier
Paul Bourdeau
Nicolas Bourdillon
Emmanuel Bourdon
Stephane Bourg
Florence Bourgeois
Mickael Bourgoin
Thierry Bourgoin
Marcelo Bourguignon
Hervé Bourhy
Virginie Bourlier
Chloe Bourmaud
Pierrick Bourrat
Audrey Bourret
Vincent Bourret
Patrick Bours
Frédéric Bousefsaf
Teun Bousema
Chad Bousman
Salah Boussen
Alex Boussioutas
Jean-Marie Bouteiller
Martyn Boutelle
Sébastien Boutin
Stan Boutin
Angela Boutte
Remi Boutteau
Emmanuelle Bouveret
Charles Bouveyron
Nicole Bouvier
Andre Bouville
Luc Bouwens
Don Bouwhuis
A. Bouwman
Ian Bouyoucos
Thierry Bouyssou
Marco Bove
Judith Bovée
Bernice Bovenkerk
Jordi Bover
Jeanne Bovet
Alan Bovik
Edward Bowden
Jocelyn Bowden
Michaela Bowden
Laura Bowe
Steven Bowe
Brian Bowen
David Bowen
Jennifer Bowen
Kathryn Bowen
Lisabeth Bowen
Nathan Bowen
Richard Bowen
Timothy Bowen

Terra Bowen-Reid
Dan Bower
Peter Bower
Bruce Bowerman
Melissa Bowerman
Alex Bowers
Andre Bowers
Russel Bowler
Ryan Bowles
April Bowling
Christopher Bowlus
Doug Bowman
Jeff Bowman
Marjorie Bowman
Nicholas Bowman
Timothy Bowman
Sarandeep Boyanapalli
Sébastien Boyas
Brendan Boyce
Anders Boyd
Brian Boyd
Lara Boyd
Norman Boyd
Ryan Boyd
Steven Kyle Boyd
Doug Boyer
Katharyn Boyer
Laurent Boyer
Michael Boyer-Guittaut
Eleanor Boyle
Elizabeth Boyle
James Boyle
Jon Boyle
Kevin Boyle
Malcolm Boyle
Noel Boyle
Primrose Boynton
Murat Boysan
Liang Boyuan
Aline Bozec
Ibrahim Bozgeyik
Ioannis Boziaris
Banu Bozkurt
Latife Bozkurt
Kayvan Bozorgmehr
Joel Bozue
Chiara Bozzacchi
Federico Bozzetti
Maria Bozzetti
Poul-Erik Braad
Georg Brabant
Mary Brabeck
Jan Brabek
Anne Brabers
William Bracamonte-Baran
Ana Paula Bracarense
Massimo Bracci
Annalisa Bracco
Sam Bracebridge
Anthony Brach
Shay Bracha
Markus Brachner
Elena Brachtel
Cameron Bracken
Heathe Bracken-Grissom
Brad Brad
Mohamed Bradai
Allison Bradbury
Louis Bradbury
Aaron Braddy
Laura Braden
Andrew Bradford
Barry Bradford
Bret H. Bradley
Charles Bradley
Chris Bradley
Colin Bradley
Darcy Bradley
Elizabeth Bradley
Jeremy Bradley
John Bradley
Jonathan Bradley
Peter Bradley
Robert Bradley
Shelton Bradrick
Amy Bradshaw
John Bradshaw
Pamela Bradshaw

Rosie Bradshaw
Matthew Brady
Nancy Brady
Patrick Brady
Johannes Braegelmann
Senne Braem
Albert Braeuning
Gilberto Braga
José Braga
L. H. Braga
Roberto Alves Braga
Nicola Bragazzi
Darrin Brager
Luca Braglia
Alessandra Bragonzi
Dan Braha
Michel Brahic
Alain Braillon
Chananel Braiman
Joseph Brain
Susan Brain
Antony Braithwaite
R. Braithwaite
C. Brakenridge
Sigrid Brakken
Emilia Bramanti
Michela Brambatti
Christian Brambilla
Anke Bramesfeld
Peter Bramley
Katrina Bramstedt
Antoine Branca
Ferdinando Branca
Rosa Tamara Branca
Gianluca Brancaccio
Andrea Brancale
Lorenzo Brancaleon
Vincenzo Brancaleone
Philip Branch
Sara Branch
V. Branchi
Cristina Branco
Luis Branco
Denys Brand
Henk Brand
Matthias Brand
Serge Brand
John Branda
Maria Brandão
Terry Brandebourg
Daniel Brandeis
Myrto Brandeker
Adriano Brandelli
Susanne Brander
Jay Brandes
Mirko Brandes
Andreas Brandholt
Christopher Brandl
Scott Brandl
Simon Brandl
Amanda Brandon
Karen Brandon
Beate Brand-Saberi
Ivan Brandslund
Jesper Brandstorp-Boesen
Annely Brandt
Eric Brandt
Jaden Brandt
Kirsten Brandt
Laura Brandt
Mark Brandt
Martina Brandt
Mary Brandt
Viktor Brandtneris
Laura Brannelly
Jonny Branney
Tiffany Brannon
Meret Branscheidt
Robert Bransfield
Ryan Branski
Richard Branson
Richard Bränström
Steven Brant
Milam Brantley
Anne Lise Brantsaeter
Norica Branza Nichita
Pardeepinder Brar
Satinder Brar

Jan Brascamp
Trevor Brasel
Tom Brashers-Krug
James Brasic
Ana Brasileiro
Kathleen Brasky
Paul Brassard
Jim Brasseur
Kevin Brasseur
Holly Bratcher
Palle Bratholm
Kaitlin Bratlie
Gennady Bratslavsky
Susan Bratton
Lisa Bratzke
Lars Bräuer
Marie Brault
Andrew Braun
Camrin Braun
Edward Braun
Jerome Braun
Jonathan Braun
Jurgen Braun
Kim Braun
Sascha Braun
Urs Braun
Bjorn Braunschweig
Bjoern Braunstein
Chad Brautigam
Paula Braveman
Nathalie Bravenboer
Eric Braverman
Alejandra Bravo
Cristián Bravo
Gina Bravo
Boris E. Bravo-Ureta
Andy Bray
J. Bray
Timothy Bray
Jordan Brayanov
João Bráz
Diane Braza
Noli Brazil
Paulo Braz-Silva
Danny Brazzale
Hana Brborovic
Mariana Brea
Maxime Breban
Nicholas Brecha
Siegmar Breckle
Annette Breckwoldt
Eduardo Breda
Leandro Breda
Cato Brede
Raphael Breder
Dara Bree
Benjamin Breeden
David Breen
Elizabeth Breen
Paul Breen
Elizabeth Breeze
Paul Breeze
Penny Breeze
Jessica Bregant
Andreas Breidbach
Ari Breiner
Korbinian Breinl
Haim Breitbart
Luana Cassandra Breitenbach Barroso Coelho
Brent Breithaupt
Fritz Breithaupt
Katja Breitkopf-Heinlein
Joerg Breitkreutz
Rainer Breitling
Maya Breitman
Fredrik Brekke
Rolf Brekken
Juergen Brem
Joel Breman
Erhard Bremer
Ross Bremner
Paul Brenchley
Andrea Brenes-Soto
Christoph Brenker
Jay Brenman
J. Brenna
Alana Brennan

Benjamin Brennan
Eoin Brennan
Irina Brennan
Jonathan Brennan
Lisa Brennan
Marnie Brennan
Patrick Brennan
Paul M. Brennan
Sean R Brennan
Tim Brennan
Erin Brennand
Christopher Brennan-Jones
Catherine Brenner
Chad Brenner
Eli Brenner
M. Harvey Brenner
Matthew Brenner
Megan Brenner
Robert Brenner
Stephan Brenner
Thorsten Brenner
David Brent
Bruno Brentan
Roberto Bresin
Alexander Breskin
Paul Breslin
Rosalind Breslow
Jacqueline Bresnahan
Philip Bresnahan
Christophe Bressac
Brigitte Bressac-de Paillerets
Fabiana Bressan
Eadric Bressel
Denise Bressler
Jan Bressler
Paul Bressloff
Patrick Brest
Marian Brestic
Brian Brestovac
Stephane Bretagne
Laura Bretherton
Ghislain Breton
David Brett-Major
Christian Brettschneider
Frank Bretz
Walter Bretz
Knut Bretzke
Kenneth Breuer
Damien Brevers
Gerhard Breves
Iver Brevik
Bruce Brew
Nadine Brew
Alison Brewer
George J. Brewer
Michael Brewer
Tom Brewer
Timothy Brewerton
Chris Brewin
Alexandra Brewis
Amy Brewster
David Brewster
Leonid Breydo
Kristijan Breznik
Jean-Michel Brezun
Emilio Bria
Ali Brian
Lisa Briand
Romain Briandet
Sophie Briand-Feve
Federica Briani
Shabeg Briar
Jean-Louis Briaud
Ana Bribian
G. Brichetto
Cameron Brick
Stephen Brickley
Ian Bricknell
Christopher Bridge
Diane Bridge
Emma Bridger
Dave Bridges
James Bridges
Nicola Bridges
Phillip Bridges
Darren Bridgewater
Laura Bridgewater
Peter Bridgewater

Byram Bridle
Helen L. Bridle
David Brieber
Jay Brieler
James Brien
Andre Briend
Stephen Brienesse
David Briers
Yves Briers
Michael Briga
Ingo Brigandt
Maximiliano Brigante
Angela Briganti
Stefania Briganti
Matthew Brigger
Brandon Briggs
Denise Briggs
Farren Briggs
John Brigham
Carlo Briguori
David Briles
Julie Brill
Stefano Brillanti
Federico Brilli
Andrew Brim
Kim Brimhall
Fadi Brimo
Pedro Brinca
Ingar Brinck
Constance Brinckerhoff
Ryan C. Brindle
Arvid Bring
Françoise Bringel
Sebastian Brings
Anna T. Brini
Christiaan Beyers Brink
Luann Brink
Rob Brink
Yolandi Brink
Kees Brinkman
Candice Brinkmeyer-Langford
Ralph Brinks
Martin Brinkworth
Shaun Brinsmade
Thomas Brioche
Christian Brion
Oscar Briones
K. H. Briones Claudett
Frederic Brioude
Francois Brischoux
Gabriel Brisola
Benoit Brisson
Heather Briston
Michael Bristow
Andre Brito
Jose Brito
Luiz Fernando Brito
Nélida Brito
Jonathan Britt
Evan Brittain
Domenico Britti
Rodrigo Britto
Erika Britz
Eric Britzke
Philip Britz-McKibbin
Davide Brivio
Eleonora Brivio
Francesca Brivio
Verónica Briz
Ambra Brizi
Maria Brizzi
Maria Felice Brizzi
Zoran Brkanac
Suzana Rimac Brnčić
Andrew Broadbent
Roland Broadbent
M. Jana Broadhurst
William Broadhurst
David C. Broadstock
Gloria Broadwater
James Broadway
Patricia Brocardo
Luca Brocca
Stefania Brocca
Egidio Brocca-Cofano
Francesco Broccolo
Franck Brocherie
Christopher Brochu

Matthias Brock
Kelvin Brockbank
Jeremy Brockelsby
Thomas Brocker
Susan Brockerhoff
Dimo Brockhoff
Karin Brocki
Heather Brockway
Peter Brodersen
Graham Brodie
Jon Brodie
Scott Brodie
Seth Brodie
Bertha Brodin
Tom Brodnicki
Alexander Brodsky
Sergey Brodsky
Cláudia Brodskyn
Amy Brodtmann
Benjamin D. Brody
Felix Broecker
Corey Broeckling
Rikke Broegaard
Mans Broekgaarden
Birit Broekman
Mark M. T. J. Broekman
Meike Broemer
Stefan Broer
Massimo Broggini
Simone Brogi
Saverio Brogna
Jakob Bro-Jorgensen
Cole Brokamp
Fernanda Brollo
John Bromfield
Kristin Broms
Alain Bron
Carel Bron
Mario Brondani
David Broniatowski
P. Bronstad
Aurora Brønstad
Jeff Bronstein
Cara Brook
J. Brooke
Martin Brooke
Paul Brookes
Zoe Brookes
Amanda Brooks
David Brooks
James Brooks
Kelly Brooks
Kevin Brooks
Logan Brooks
Lyndon Brooks
Phillip Brooks
Rechele Brooks
Samantha Brooks
Simon Brooks
Geoff Brookshire
Wouter Broos
Ann-Christin Brorsson
Mikael Brosche
Jan Brosens
Jeannette Brosig-Koch
Peter Brossart
Florence Brossier
Rebecca Brotman
Jose M. Brotons
Carlos Brotons-Cuixart
Marco Brotto
Eugenia Broude
Mark Brough
Paula Brough
Luc Brouillet
Gueorgui Broukhanski
Benedicte Brounais
Philippe Brouqui
Louise Brousseau
Isabelle Broutin
Andrew Brouwer
Cory Brouwer
Ingeborg Brouwer
Miranda Brouwer
Henriëtte Brouwer-Middelesch
Melissa Brouwers
Alan Brown
Alexander Brown

Amanda Brown
Andre Brown
Andrew Brown
Anthony Brown
Azby Brown
Caleb Brown
Candice M. Brown
Charles Brown
Colin Brown
Craig Brown
Daniel Brown
Daniel J. Brown
David Brown
Deborah Brown
Denver Brown
Douglas Brown
Edwina Brown
Eric Brown
Felicity Brown
Frankie Brown
Gillian Brown
Gordon Brown
Grant Brown
Gregory Brown
Hamish Brown
Hannah Brown
Heather Brown
J. Brown
J. Quincy Brown
Jacques Brown
James Brown
Joelle Brown
Joshua Brown
Juanita Brown
Karen T. Brown
Kelly Brown
Kevin Brown
Kevin E. Brown
Lawrence Brown
Lawrence W. Brown
Lillian Brown
Lou Ann Brown
Mark Brown
Michael Brown
Molly Brown
Nancy Brown
Paul Brown
Paula Brown
Solange Brown
Stephen H. M. Brown
Steve Brown
Steven Brown
Stuart Brown
Susan Brown
Todd Brown
Tyler Brown
Wendy Brown
Holly Brown-Borg
Sara Brownell
Carole Browner
Ursa Brown-Glaberman
Sharon Brownie
Dawn M. Browning
Kirsteen Browning
Steven Browning
Jeremy Brownlie
Sarah Brown-Schmidt
Jeffrey Brownson
Buddy Brownstein
Jens Ivar Brox
Hal Broxmeyer
Petr Broz
Frank Brozovich
Anna Brozyna
Claudio Brozzoli
Julius Brtko
John Brubacher
Linda Brubaker
Beau Bruce
Eleanor Bruce
Julie Bruce
Thiago Bruce
Sandra Brucet
Iris Bruchhaus
P. L. Bruckner
Tim-Allen Bruckner
Sabrina Brückner
Katrina Brudzynski

Tanja Bruechert
Robert Brueggeman
Angela Brueggemann
Jason Bruemmer
Gemma Bruera
Harald Bruessow
S. Brugaletta
Jean-François Brugère
Daniel Brugman
Radan Bruha
Alejandro Bruhn
Sjoerd Bruijn
Patricia Bruijning-Verhagen
Peter Bruins
Sylvia Bruisten
Stanley Brul
Audrey Brumback
Paul Brumer
Jason Brumitt
Chanson Brumme
Susanne Brummelte
Boris Brumshtein
Fernando Brun
Paul Brunault
Giuseppe Brundu
Jurgen Brune
Anne Bruneau
Angele Bruneillere
Diede Brunen
Frédéric Brunet
Antonio Brunetti
Giacomina Brunetti
Natale Brunetti
Nicola Brunetti-Pierri
Sonja Brungs
Amalia Bruni
Paola Bruni
John Bruning
Sascha Brunke
Melanie Brunn
Cornelia Brunner
Iris Brunner
Juerg Brunnschweiler
Barbara Bruno
Christie Bruno
John Bruno
Paul Bruno
Rafaela Bruno
Robert Bruno
Stefania Bruno
José Bruno-Bárcena
Andre Brunoni
Gianluca Brunori
Celine Brunot-Gohin
Mabel Brunotto
Tony Bruns
Jason Brunson
Berit Brurok
Silvio Brusaferro
Matteo Bruschettini
Fabrizio Bruschi
Maurizio Bruschi
Stefano Bruscoli
Oystein Bruserud
Nele Brusselaers
Nickolay Brustovetsky
Tom Brutsaert
Jesper Bruun
Olivier Bruyère
Petr Bruza
Maria Bruzelius
Angela Bruzzaniti
Ariana Bruzzone
Octavio Bruzzone
Arielle Bryan
Laura Bryan
Nathan Bryan
Andrew Bryant
Camron Bryant
Gregory Bryant
Josephine Bryant
Megan Bryanton
Ashley Bryce
Josef Bryja
Michal Brylinski
Eric Brymer
Randy Bryner
Mark Brynildsen

Marc Brysbaert
Patrick Brzoska
Emilia Brzosko
Zbigniew Brzozka
Marlena Brzozowa
Anna Brzozowska
Paweł Brzuzan
Heng-Fu Bu
Kai Bu
Zhan Bu
Zhongming Bu
Alexis Buatois
Jamile Bubadué
Isabella Buber-Ennser
Concetta Bubici
Giovanni Bubici
Maurice Bucagu
Franz Bucar
Annalisa Bucchi
David Bucci
Maria Pia Bucci
Chiara Bucciarelli-Ducci
Bob Bucelli
Eric Buch
Alastair Buchan
Blake Buchan
John Buchan
Briggs Buchanan
David Buchanan
Elizabeth Buchanan
Gordon Buchanan
John Buchanan
Daniel Bucher
Andre Buchheister
Andrea Buchholz
Bruno Buchholz
Malte Buchholz
Sascha Buchholz
Reiner Buchhorn
Anatoly Buchin
Robert Buchkowski
David Buchner
Florian Buchner
D. J. Buchsbaum
Ana Bucic
Gabriela Bucini
Gregory Buck
Hudson Buck
Maren Buck
Marcos Silveira Buckeridge
Carol Bucking
David Buckley
Hallie Buckley
J. Buckley
John Buckley
Katherine Buckley
Laura Buckley
Mark Buckley
Michael Buckley
Nicholas Buckley
Rachel Buckley
Ralf Buckley
Frederick Buckner
Octavian Bucur
Mesut Budak
Ann Budd
Tom Budd
Katharina Budde
Matthew Budde
Thomas Budde
Shyam Budhathoki
Suman Budhwani
Mauricio Budini
Michał Budka
Bruce Budowle
Steven Budsberg
Alexandre Budu
Nurcan Buduneli
Katarzyna Budzynska
Ronny Buechel
Christa Buechler
Andreas Buechner
Christian Bueger
Mart Buekers
Joseph Buell
Jose Buenaposada
Jefferson Antonio Buendia
Michaela Buenemann

Alfonso Bueno
Raphael Bueno
Ruben Bueno
Aurora Bueno-Cavanillas
Diana Bueno-Gutierrez
Bruno Bueno-Silva
Marco Buenrostro-Nava
Pascal Buenzli
Claudia Buerger
Florian Buether
Pierre Buffet
Annalisa Buffo
Gesine Bug
Aurelia Bugaiska
Stephanie Bugden
James Bugeza
Timothy Bugg
Heiko Bugger
Tim Bugni
Leandro Bugoni
Jennifer Bugos
Beáta Bugyi
Jan-Hendrik Buhk
Ethan Buhr
Michael Buhrmester
Catalin Buhusi
Christine Bui
Samantha Bui
Thanh Bui
Tuan Bui
Francesco Buia
Arjan Buis
Yves Buisson
Albert Buitenhuis
Maria Jose Buitrago
Christo Buizert
Alessandra Buja
Emmanuel Bujold
Salome Bukachi
Henryk Bukowski
Radek Bukowski
William Bukowski
Alexander Bukreyev
Dieter Bulach
Michał Bulc
Philippe Bulet
Omar Bulgari
Cynthia Bulik
Sheana Bull
Susan Bull
Tim Bull
Camilo Bulla
Jan Bulla
Stephan Bullard
Esther Bullitt
Susan Bullman
Victoria Bullock
John Bullough
Andrew Bulmer
Vincent Bulone
Rebecca Bulotsky Shearer
Daniel Bulte
Janet Bultitude
Laurent Bultot
Zafer Bulut
Dirk Bumann
Jan Bumb
Chris Bumby
Jesse Bump
Donna Bunch
Kanitta Bundhamcharoen
Mirco Bundschuh
David Bundy
Leonid Bunegin
Chris Buneo
Mary Bunge
Silvia Bunge
Stephen Bunker
Lina Bunketorp-Kall
Christian Bunn
Paul Bunn
Eline Bunnik
Kanokwan Bunsawat
Claudia Buntrock
Samantha Bunzli
Marine Buon
Francesco Buonocore
Dean Buonomano

Michell Buonopane
Danilo Buonsenso
Xabier Buque
Yoram Burak
Raquel Buranelli
Charles Burant
Jose Buratini
Laura Buratti
George Burba
Christian Burban
Peter Burbelo
Javier. Burchard
Brendan Burchell
David Burchfield
Roxana Burciu
Irene Burckhardt
Frank Burczynski
Martin Burd
Nicholas Burd
Brittany Burda
Hynek Burda
Kvetoslava Burda
Franciszek Burdan
Christy Burden
Joanna Burdette
Nicole Burdick Sanchez
Justin Burdine
Tricia Burdo
Thomas Burdon
Jasmina Burdzovic Andreas
Ronan Bureau
Vladimír Bureš
Andre Buret
Theresa Burg
Daniel Burgas
F. Burgaya
Jeffrey Burgdorf
Colleen Burge
Dylan Burge
Mark Burge
Lot Burgemeister
Christina Burger
Huibert Burger
Joanna Burger
Pamela Burger
Erik Burgerhout
Graham Burgess
John Burgess
Matthew Burgess
Shawn Burgess
Treena Burgess
Kyle J. Burghardt
Arthur Burghes
Gaetan Burgio
Giovanni Burgio
Judee Burgoon
John Ashley Burgoyne
Nik Burhan
Maggie Burhans
Katrin Burkart
Candace Burke
Darren Burke
Gaelen Burke
Ian Burke
Matthew Burke
Michael Burke
Sean Burke
Shauna Burke
Tom Burke
Kent Burkey
Brant Burkhardt
Gillian Burkhardt
Pawel Burkhardt
Jason Burkhead
Kristin Burkholder
Michael Burkitt
Howard Burkom
Andre Burkovski
A. Wesley Burks
Charles Burks
Stephen Burks
Rishi Burlakoti
Alfredo Burlando
Barbara Burleigh
Philippe Burlina
William Burlingham
Alita Burmeister
David Burmeister

Jacob M. Burmeister
David Burnes
Phil Burnet
Barrington Burnett
Rosemary Burnett
Brendan Burns
Christine Burns
Jane Burns
Katherine Burns
Mark Burns
Michael Burns
Mike Burns
Pippa Burns
Richard Burns
Sean Burns
Stephen Burns
Timothy Burns
Zachary Burns
Shauna Burnsilver
Alberto Búrquez
Brandon K. Burr
Hannah Burrack
Pablo Burraco
Peter Burrage
Bryna Burrell
Melissa Burroughs Peña
Carole Burrow
Heather Burrow
Anne Burrows
Catherine Burrows
Mike Burrows
Vincent Burrus
Moa Bursell
David Burstein
Michael Bursztyn
John Burt
Chris Burtin
A. Mike Burton
Andrew Burton
Charles Burton
Edward Burton
Elissa Burton
G. Burton
J. W. Burton
Patrick Burton
Ron Burton
Rosie Burton
Dallas Burtraw
Martin Burtscher
Benjamin Burwitz
Oleksandr Burylko
Sakib Burza
Beata Burzynska
Artur Burzyński
Naftali Busakhala
Gianni Busato
Philip Busbee
Jasmin Busch
Jonathan Busch
Rebecca Busch
Robert Busch
Theresa Busch
H. Buscher
Konrad Buscher
Claas Buschmann
Michael Buschmann
Lowell Busenitz
Claudio Busettini
Florence Buseyne
Adam Bush
Alex Bush
Ashley Bush
Daniel Bush
David Bush
Karen Bush
William Bush
Peter Busher
Wade Bushman
Florent Busi
Daniel Maria Busiello
Julia Busik
Doreen Busingye
Susan Buskin
Steven Buskirk
Andrea Busnelli
Antonio Buso
Nuria Busquets
Karen Bussard

Bert Busscher
Bjoern Busse
Carol Bussey
Melanie Bussey
Giovanni Bussi
Hermann Bussmann
Gianni Bussolati
Ovidio Bussolati
Alejandro Bustamante
M. A. Bustamante
Ramiro Bustamante
Víctor Bustamante
Ivan Bustamante Filho
Ricardo Bustos Guajardo
Ana Busturia
Sachit Butail
Jon Butchar
Jonathan Butchar
Matthew Butchbach
Neville Butcher
Marie José Butel
Anzhelika Butenko
Jacopo Buti
Peter Bütikofer
Vince L. Butitta
Alexandra Butler
Alison Butler
Andrew C. Butler
Aodhan Butler
Brian Butler
Caitlyn Butler
David Butler
Erin Butler
Geraldine Butler
James Butler
John Butler
Katelyn Butler
Marguerite Butler
Mark Butler
Mary Butler
Matthew Butler
Stephen Butler
Thomas Butler
Tom Butler
Monica Butnariu
José Butori Lopes de Faria
Marina Butovskaya
Oleg Butovsky
Phyllis Butow
Melinda Butsch Kovacic
Van Butsic
Elke Butt
Louise Butt
Bettina Buttaro
Barbara Buttenfield
Klaus Buttenschoen
Stephen Butterfill
Frank Buttgereit
Valentina Buttiglione
Oliver Büttner
Mark Button
Christine Buttorff
Nilza Buttow
Mirko Buttrini
Carter Butts
Daniel Butts
Anja Buttstedt
Alexander Butwick
Helmut Butzkueven
Anne Buve
Joel Buxbaum
David Buxton
Iain Buxton
Rachel Buxton
Richard Buxton
Jan-Pieter Buylaert
Heloise Buys
Wouter Buytaert
Erkan Buyuk
Ender Büyükgüzel
Marilia Buzalaf
Leonora Buzanska
Marcos Buzanskas
Lubos Buzna
Hatice Dogan Buzoglu
Pietro Buzzini
Mutsa Bwakura-Dangarembizi
Enkhmaa Byambaa

Winston Byblow
Sergio Bydlowski
Ali Bydon
Derek Byers
Emily Byers
James Byers
Kaylee Byers
Krista Byers-Heinlein
Ib Bygbjerg
Magnus Bygren
Siarhei Bykau
Maria Bykhovskaia
Yelena Bykhovskaya
Julie Byles
Carma Bylund
Katarzyna Byrka
A. Byrne
Audi Byrne
Dallan Byrne
Helen Byrne
Karl Byrne
Kerry Byrne
Maria Byrne
Richard (Dick) Byrne
Richard Byrne
Robert Byrne
Elizabeth Byrnes
Kenneth Byron
Jinyoung Byun
Danilo Bzdok
Pablo C. Baldí
Joan Caballeria
Armando Caballero
Francisco Félix Caballero
Julio Caballero
Maria Dolores Caballero
Susana Caballero
Juan Caballero-Perez
Miguel Cabanellas-Reboredo
Allison Caban-Holt
Claudio Cabello
Claudio Cabello-Verrugio
Ricardo Cabeza
Maria Cabezas
Manuela Cabiati
Simona Cabib
Andrea Cabibbe
Giuseppe Cabibbo
Candido Cabo
Catalina Cabot
Florence Cabot
Ryan Cabot
Antonio Cabral
Luís Cabral
Pedro Cabrales
Carolina Cabral-Santos
Cecilia Cabrera
Antonio Cabrera de León
Delia Cabrera DeBuc
Jan Cabri
Elsa Cabrita
Miriam Cabrita
Rita Cacace
Ercan Cacan
Lucio Caccamo
Nadia Caccamo
Silvoa Caccia
Francesco Cacciatore
Joanne Cacciatore
Michael Cacciatore
Santa Olga Cacciola
Adalgisa Caccone
Alfredo Caceres
Billy Caceres
Alejandro Cáceres
Dante Cáceres
Nicolas Cachanosky
Thibaut Cachon
Jerome Cachot
Dorina Cadar
Diego Cadavid
Luis Cadavid
Brian Cade
Dave Cade
Anthony Cadena
Hinsby Cadillo-Quiroz
Lisa Cadmus-Bertram
Eduardo Cadore

Jef Caers
Line Caes
António Caetano
Gina Caetano
Cecilia Café
Stefano Caffarri
Anwen Caffell
Barbara Caffery
L. Caffino
Conor Caffrey
Jane Caffrey
Sonia Cafieri
Jonathan Cagan
Giuseppina Caggiano
Rachele Cagliani
Alexandro Cagliari
Petra Cagnardi
Simone Cagno
Christine Cagnon
Lucero Cahuana-Hurtado
Changmeng Cai
Chao Cai
Chen-Leng Cai
George Cai
Guoping Cai
Hua Cai
Jianchao Cai
Jiansheng Cai
Kaiquan Cai
Li Cai
Li Zhe Cai
Michael Cai
Minggang Cai
Qing Po Cai
Qingqiong Cai
Shanshan Cai
Shengguan Cai
Shi-Min Cai
Shuang Cai
Shuqun Cai
Tianxi Cai
Tommaso Cai
Wei Cai
Weibo Cai
Wenhua Cai
Xia Cai
Xiao-Hong Cai
Xiaoqing Cai
Xiong Cai
Xiuling Cai
Xun Cai
Yan Cai
Yikun Cai
Yongli Cai
Yu-Dong Cai
Zhejun Cai
Zhen Cai
Zhenguang Cai
Gianluca Caiazzo
R. Caiazzo
Carlos Caicedo Bastidas
Alexander Caicedodorado
Celia Caillet-Saguy
Julie Caillon
Melissa Caimano
Kelvin Cain
Kristal Cain
Saverio Caini
Kylie Cairns
Matthew Cairns
Gaetano Cairo
Mitchell Cairo
Nicole Caixeiro
Luciano Caixeta
Ozgur Cakir
Tunahan Cakir
Canan Cakirlar
Bulent Cakmak
Dimitrios Cakouros
Antonio Cala
Steven Cala
Joseph Calabrese
Vittorio Calabrese
Bianca Calabria
Ricardo Calado
Rodrigo Calado
Gloria Calaf
Antonia Calafat

Timothy Calamaras
Jimmy Calanchini
Blair Calanci
Sebastiano Calandra
Emanuela Calandri
Alessia Calcagni
Maria Calcagnotto
Nigel Calcutt
Benoit Caldairou
Adele Caldarella
Eloiza Caldart
Vinicius Caldart
Daniel Caldeira
Maria Elena Caldeon-Segura
Alistair Calder
Philip Calder
M. R. Calderon
Tina Calderon
Luis Calderon-Aguilera
Lilian Calderon-Garciduenas
Marissa Caldow
Paul Caldron
Josep Calduch-Giner
Aaron Caldwell
Ann Caldwell
Barrett S. Caldwell
Claude Caldwell
Kevin Caldwell
Kim Caldwell
Maeve Caldwell
Federica Calevro
Claudia Cali
Corrado Cali
Ricardo Caliari
Steven Caliari
Rudy Calif
Maria Caligo
Daniele Calistri
David Calkins
Marcus Calkins
Monica Calkins
Douglas Call
Jarrod Call
Bernarda Calla
Brid Callaghan
Michael Callaghan
Timothy Callaghan
Rebecca Callahan
Priscilla Callahan-Lyon
Marie-Pierre Callait-Cardinal
John Callan
Mitchell Callan
Denton Callander
Emily Callander
Maurizio Callari
Clifton Callaway
Todd Callaway
Rachael Callcutt
Giuditta Callea
Jesus Maria Calleja
Jose Luis Calleja
Fernando Callejas-Albiñana
Patrick Callery
Peter Callery
Fabrizio Calliada
Danilo Calliari
Robin Callister
Josep Callizo
Virginie Callot
William A. Calo
Antonio Calò
Rachel Calogero
Natalia Calonghi
Martina Calore
Ryan Calsbeek
Mario Calus
Thomas S. Calvard
Vincenza Calvaruso
Michael Calver
Amanda Calvert
Sarah Calvert
Juan Calvete
Hector Calvete-Sogo
Rossella Calvi
Cinzia Calvio
B. Calvo
Manuel Calvo
Maria Angeles Calvo

Rocio Calvo
Andrea Calvo Echenique
Monica Calvo-Polanco
Laura Calza
Sara Calzavarini
Luigino Calzetta
Laurence Calzone
Hakan Cam
Carlos Camacho
José-Fernando Camacho-Vallejo
Simonetta Camandola
William Camann
Amadou Camara
Carlos Camara-Lemarroy
Alessandra Camarca
Adriana Camargo
Antonio Camargo
Carlos Camargo
Ilana Camargo
Luiz Sérgio Camargo
Amelia Camarinha-Silva
Alessandra Cambi
Valentina Cambiano
Sebastien Cambier
Marie Anne Cambon-Bonavita
Marie-Anne Cambon-Bonavita
Erik Cambria
Daymi Camejo
Benoit Camenen
Emanuela Camera
Anne-Linda Camerini
Irene Camerlink
Dawn Cameron
Donnie Cameron
Heather Cameron
Kenneth Cameron
Lisa Cameron
Michael Cameron
Nicole Cameron
Robert Cameron
Scott Cameron
Sharon Cameron
Carlo Camilloni
Jorge Caminos
Antoni Camins
Irina Caminschi
Antonio Cammarata
Florence Cammas
Analuiza Camozzato
Elizabeth A. Camp
Ernest Camp
Michele Campagna
Luisa Campagnolo
Michael Campana
Steven Campana
Claudia Campanella
Luigi Campanella
Valentina Campanelli
Alexandra Campbell
B. Campbell
Benjamin Campbell
Benjamin R Campbell
Bruce Campbell
Colin Campbell
Duncan Campbell
Edward Campbell
Eleanor Campbell
Ellsworth Campbell
Emily Campbell
Hamish Campbell
Iain Campbell
Ian Campbell
Janis Campbell
Jonathan Campbell
Lee Ann Campbell
Lisa Campbell
Matthew Campbell
Patricia Campbell
Rebecca Campbell
Richard A. Campbell
Roderick Campbell
Sharon Campbell
Steven Campbell
Tavis Campbell
W. Campbell
Martha Campbell-Thompson
Sandra Campeanu
Paloma Gómez Campelo

Vito Campese
Oscar Campetella
Clément Campillo
Giovanna Cristina Campione
Nicolas Campione
Fabio Campioni
Giuseppina Campisi
Guillermo Campitelli
D. Campobello
Orlando Campolo
Alessandra Campos
Eduardo Campos
Eleonora Campos
Elisa Campos
Jennifer Campos
Jose Luis Campos
Maria Campos
Mateus Campos
Nicole Campos
Samuel Campos
Sara Campos
Ana Campos Marin
Ahimsa Campos-Arceiz
Eric Campos-Canton
Paulo Campregher
Carlos Camps
Manel Camps
Tomas Camps
Jorge Campusano
Susana Campuzano
Victoria Campuzano
Frederic Camu
Lionel Camus
Ozgu Can
David Canaday
Nelson Augusto Canal
Donaldo Canales
Elisabetta Canali
Bruno Canard
Alberto Canarini
João Canário
Luis Cañas
Rafael Cañas
Bernard Canaud
Guillaume Canaud
Maureen Canavan
Federico Canavese
Sule Canberk
Corrado Cancedda
J. Cancela
Javier José Cancela
José Cancela
Saranya Canchi
M. Canda
Selami Candan
Robin Candau
Jessica Cande
Leonardo Candela
Pietra Candela
Eduardo Candelario-Jalil
Jean-Jacques Candelier
Luca Candeloro
Esin Candemir
Sophie Candfield
E. Candi
Matteo Candidi
D. Candotti
Fabio Candotti
Bridget Candy
Miguel Cañedo-Argüelles
Laura Canesi
Stefano Canessa
Martha Canfield
Nuno Canha
Helena Canhao
Patrice Cani
Nuria Canibe
Manuela Caniça
Romolo Caniglia
Maria Canillas
Martin Canis
Michel Canis
Mayilee Canizares
Christine Canman
Marissa Cann
Giuseppe Cannalire
Antonio Cannatelli
Alessandro Cannavo

Suzanne Cannegieter
Belinda Cannell
Joseph Canner
Linda Cannizzaro
Renato Cannizzaro
Barbara Cannon
Brad Cannon
Chuck Cannon
Judy Cannon
Ainara Cano
Alberto Cano
Francisco Cano
Stefan Cano
Virginie Canoine
William Canon
Barbara Canonico
François Canonne-Hergaux
Mary Cano-Sarabia
A. Canovas
David Canovas
Manuel Cánovas
Ana Canovas Martinez
Fuat Emre Canpolat
Vincenzo Cantaluppi
Lourdes Cantarero-Arévalo
Francesco Cantatore
Robert Canter
Jaime Cantera
Jose L. Cantero
Chiara Cantiani
Laura Cantini
Vito Cantisani
Carlos Canto
Fabio Canto
Efrain Canto-Lugo
Gador Canton
Mauricio Cantor
Sharon Cantor
Arnulfo Gonzalez Cantu
Irina Canu
Federico Canzian
Vincenzo Canzonieri
Bin Cao
Binyun Cao
Dianjun Cao
Dong Cao
Fangbin Cao
Fuyuan Cao
Gaoyi Cao
Guangwen Cao
Guanqun Cao
Hong Cao
Hongsheng Cao
Hongyuan Cao
Jian Cao
Jiang Cao
Jie Cao
Jingli Cao
Jun Cao
Junzhe Cao
Kai Cao
Kan Cao
Lianjie Cao
Lixiang Cao
Miao Cao
Nan Cao
Peijian Cao
Peng Cao
Pinlu Cao
Qi Cao
Ruifeng Cao
Sha Cao
Sheng Cao
Shuanghe Cao
Weibiao Cao
Xianghui Cao
Xiaolong Cao
Xiaoyan Cao
Xu Cao
Yan Cao
Yi Cao
Yopng-Ping Cao
Yu Cao
Yu-Qing Cao
Zhe Cao
Zhipeng Cao
Maurizio Caon
Christiane Caouette

Pasquale Capaccio
Jesse Capacelatro
Jamie Capal
Jacek Capala
Elizabeth Capaldi
Gabriele Capannini
Serge Caparos
Elise Capella
Gabriel Capellá
Giovanni Capelli
Irene Capelli
V. L. Capelozzi
Patrice Capers
Quinn Capers IV
Ana Caperta
Amanda Capes-Davis
Yassemi Capetanaki
Amedeo Capetti
Jacob Capin
John Capinera
Dario Capizzi
Louis Caplan
Steve Caplan
Jackie Caplan-Auerbach
Jodi Caple
Ben Caplin
Alessandro Capo
John Capobianco
Paolo Capodaglio
Marco Capogrosso
Francesca Capon
Vittorio Capozzi
Alejandra Capozzo
Celine Cappe
Mario Cappellette Jr.
Vera Cappelletti
Enrico Cappellini
Germana Cappellini
Angelo Cappello
Francesco Cappello
Judith Capper
Shannon Capps
Justin Cappuzzo
C. Monica Capra
Laura Capranica
Piera Capranzano
Valerio Capraro
Stella Capriglione
Francesco Caprio
Massimiliano Caprio
Stuart Capstick
Silvia Capuani
Annalisa Capuano
Mattia Capulli
Margareth Capurro
Laurence Capus
Palmer Cara
Rey Carabeo
Caroline Caradu
Eric Caragata
Michele Caraglia
Laura Caram
Maria-Jose Caramujo
Marcelo D. Carattino
Anthony Caravaggi
Francesco Caravelli
Nicolas Carayol
José J. Carbajal-Hernández
Julio Carballido
Fabricio Carballo
Martin Carballo-Pacheco
Claus-Christian Carbon
Francis Carbonaro
Emilio Carbone
Michele Carbone
Simona Carbone
Angel A. Carbonell-Barrachina
Pablo Carbonell-Bejerano
Franck Carbonero
Esperanza Carcache de Blanco
Andrea Carcelen
Nick Carcioppolo
Roderick Card
Rosanna Cardani
Ring Cardé
Michelle Cardel
Jesús Cardells-Peris
Marcus Carden

Ibsen C. Cardenas
María Del Carmen Cárdenas-Aguayo
Leopoldo Eduardo Cárdenas-Barrón
M. Alejandro Cardenete
Valentina Cardi
Alessio Cardillo
Giuseppe Cardillo
Rhonda Cardin
Eric Cardinale
Massimiliano Cardinale
Vincenzo Cardinale
Pablo Cardinal-Fernández
Beatrice Cardinali
Giorgia Cardinali
Lucilla Cardinali
Genis Cardona
Nathalia Cardona
Paula Cardona
Nora Cardona-Castro
Armando Cardoso
Cristina Cardoso
Danon Cardoso
Felipe Cardoso
Hugo Cardoso
Luis Cardoso
Rosilene Fressatti Cardoso
Kitty Cardwell
Alessandra Carè
Lucio Careddu
Carine Carels
Jan Carette
Marie-France Carette
Giuseppina Caretti
Melissa Carew
Alison Carey
James Carey
Kathleen Carey
Robert Carey
Stephanie Carey
Angelo Carfi
Alessia Cariani
Marica Cariello
Jessica Carilli
Petronila Carillo
Paloma Cariñanos
Fabrizio Carinci
Gerardo Carino
Marios Cariolou
Oriol Caritg
Dan Cariveau
Maria Carlan Silva
Carsten Carlberg
Corinne Carle
Alfonso Carleo
Nadia Carlesso
R. Nicholas Carleton
Marco Carletti
Aurelien Carlier
Angela Carlin
Bradley Carlin
Danielle J. Carlin
Celia Carlini
Elisa Carlino
Jay Carlisle
Chiara Carlomagno
Paolo Carloni
Silvia Carlos
Carmelo Carlo-Stella
Bruce Carlson
Christina Carlson
Erin Carlson
Grant Carlson
J. Andrew Carlson
Jenny Carlson
Jordan Carlson
Lawrence Carlson
Nicholas Carlson
Nichole Carlson
Susan Carlson
Jens Carlsson
Eric Carlström
Mattias Carlström
James Carlucci
Roberto Carlucci
Erberto Carluccio
Cameron Carlyle
James Carlyle
Robert Carlyon

Christopher Carman
David Carmena
Shai Carmi
Joseph Carmichael
Maria Carmo-Fonseca
Carmelo Carmona-rivera
Gilles Carnac
Angus Carnegie
Cláudia Carneiro
Fernando Carneiro
Regina Carneiro
Amancio Carnero
Andrés Carnero
Dawn Carnes
Daniela Carnevale
Elaine Carnevale
Giorgio Carnevale
Roberto Carnevale
Luca Carnevali
Ryan Carney
Simon Carney
Sebastien Carnicella
Nicole Carnt
Tim Caro
Jildaz Caroff
Jerusalin Carol
José Carolino
François Caron
Jeffrey Caron
Alejandro Caro-Quintero
Erik Caroselli
Monica Carosi
Ylenia Carotenuto
Yanink Caro-Vega
Berit Carow
G. Carpagnano
Catherine Carpenter
David Carpenter
Ellen Carpenter
Guy Carpenter
James Carpenter
Kevin Carpenter
Mark Carpenter
Simon Carpenter
Timothy Carpenter
Giovanna Carpi
Ilaria Carpinella
Guido Carpino
Pedro Carpintero
Frances Carr
Georgina Carr
Jillian Carr
John Carr
Richard Carr III
Clotilde Carra
Natacha Carragher
Camillo Carrara
Raffaele Carraro
Luis Carrascal
Cristina Carrasco
Javier Carrasco
Maria Carrasco
Marina Carrasco Llatas
Pilar Carrasco-Garrido
Kermit Carraway
Justin Carre
Madeleine Carreau
Alex Carrega
Rodrigo Carregaro
Margaret Carrel
Douglas Carrell
Leah Carreon
Teresia Carreon
Helaine Carrer
Ana Carrera
Guillermo Carrera
Olaia Carrera
Elena Carreras
Miguel Ángel Carretero
Danielle Carrick
João Carriço
Fabian Carriconde
Erica Carrier
Frederic Carriere
Yves Carriere
Patrizia Carrieri
Vincenzo Carrieri
Maria Vincenza Carriero

A. Carrillo
Maria Carrillo
Rogelio Carrillo González
Enrique Carrillo-de-Santa-Pau
Maria Alicia Carrillo-Sepulveda
Christine Carrington
Mark Carrington
Francisco Carrión
Àngela Carrió-Seguí
Eva Carro
Gareth Carrol
Chad Carroll
E. Carroll
John Carroll
Robert Carroll
Steven Carroll
Suzanne Carroll
Philip Carrott
Danilo Carrozzino
Vern Carruthers
Alan Carson
Brian Carson
James Carson
Joann Carson
Kristin Carson
Richard Carson
Valerie Carson
Oliver Carsten
Bendix Carstensen
Jacob Carstensen
Angelino Carta
Monica Cartelle Gestal
Marc Cartellieri
A. Brent Carter
A. W. Carter
Carol Carter
Clay Carter
E. Carter
Eric Carter
Evan Carter
Gerald Carter
Greg Carter
Hannah Carter
Morgan Carter
Stephen Carter
Tonia Carter
Virginia Carter Leno
Joan Cartes
Richard Carthew
Alexandra Carthey
Olivier Cartiaux
Sian Cartland
Kathleen Cartmell
Rufus Cartwright
Christopher Carty
Michael Carty
Marie-Line Caruana
Oliviero Carugo
Em Caruso
Nicolás Caruso
Emmanuel Caruyer
Danny Carvajal
Rd Carvajal
Luis Carvajal-Carmona
Raquel De Vasconcellos Carvalhaes de Oliveira
A. P. Carvalho
Agostinho Carvalho
Ana Luisa Carvalho
Brendan Carvalho
Daniel Carvalho
Ines Carvalho
Jorge Carvalho
Luis Carvalho
Luisa Carvalho
Marilia Carvalho
Natalie Carvalho
Simone Carvalho
Tecia Carvalho
Thiago Carvalho
Verena Carvalho
Jaqueline Carvalho de Oliveira
Michael Carvan III
Wayne Carver
Anne-Ruxandra Carvunis
Eduardo Casado
José Luis Casado
Patrizia Casagrandeproietti

Renato Casagrandi
Paolo Casali
Ferran Casals
Beata Casanas
Didier Casane
Emilio Casanova
Mikel Casares
Renato Corrêa Viana Casarin
Dulce Elena Casarini
Gloria Casaroli
Alejandro Casas
Irene Casas
Jérome Casas
Mariana Casas
Juan Armando Casas Mollano
Sergio Casas Tintó
Sergio Casas-Flores
Giovanni Casazza
João Cascalheira
Adam Case
Elizabeth Case
Kelsey Case
Laura Case
Chiara Caselli
Elisabetta Caselli
Richard Caselli
Stefano Caserta
Aleix Cases
Luca Casettari
Gregory Casey
Joan Casey
Sara Casey
Theresa Casey
Robin Cash
Patricia Casino
Grace I. L. Caskie
Giulia Casorati
Corey Casper
Keith Casper
Robert Casper
Luis Caspeta
Rachel Caspi
Gladys Cassab
Helen Cassaday
Alexandra Cassado
Geovanni Cassali
Fabricio Cassan
Barbara Cassani
Anne-Marie Cassard
Jp Cassella
Daniele Cassese
Pamela Cassiday
Brittany Cassidy
Rachel Cassidy
Sophie Cassidy
Jean-Jacques Cassiman
Ruas Cassio
Stuart Casson
Vincent Cassone
Antonella Castagna
Fausto Castagnetti
Silvana Castaldi
Clotilde Castaldo
Carlos Castaneda
Homero Castaneda
Luis Castañeda
Santos Castañeda
Diego Castanera
C. Castano
Emanuele Castano
Gustavo Castano
Justo P. Castaño
Darren Casteel
Jean-Christophe Castel
Mathieu Castela
Francisco Castelán
Beatrix Castelar
Rene Castelein
Jonas Castelijns
Madhura Castelino
Gemma Castella
Joan Castellano
F. Xavier Castellanos
Ignacio Castellanos
Anna Maria Castellazzi
Luigi Castelli
Alessandra Castellini
Miris Castello

Nicholas Castello
Manuel Castellote
Barbara Castelnuovo
Giulio Castelpietra
Serenella Castelvecchio
John Casterline
Perrine Castets
Dominique Castex
Analia Castiglioni
Paolo Castiglioni
Patrice Castignolles
Andrea Castillo
Daniel Castillo
Eduardo Castillo
Jorge Castillo
Pablo Castillo
Richard Castillo
María Castillo-García
Ricardo Castillo-Neyra
Alexandra Castillo-Ruiz
Steven Castle
Sophie Castle-Clarke
Erica Casto
Annie Castonguay
Gabriella Castoria
Cristina Castracani
Mauricio Castrejón
Maija Castren
Antonio Castrillo
Alexandre Castro
Cecilia Castro
Helena Castro
I. T. Castro
Isabel Castro
Joseane Castro
Juan Castro
L. Filipe Castro
Marcelo Castro
Marcia Castro
Michelle Castro
Pedro M. Castro
Rosario Castro
Talita Castro
Thiago Castro
Vanina Castroagudin
Ivan Castro-Arellano
William Castro-Borges
Maria Castro-Codesal
Erico Castro-Costa
Pilar Castro-Diez
Hugo Castro-Faria-Neto
Enrique Castro-Sánchez
Theodore Castro-Santos
Ana Maria Castrucci
Anna Casu
Manuela Casula
Clayton Caswell
Hal Caswell
Neus Català
Ana Catalan
Pilar Catalán
Patrick Catalano
Angel Cataldi
Sergio Catanozi
Ilaria Catapano
Antonino Catara
Jamie Cate
Matjaž Čater
Joan Cates
Danielle Cath
Auriemma Catherine
Nisha Catherine
Emilie Catherinot
Henry Cathey
Andrew Catley
Jesse Catlin
Sergiu-Bogdan Catrina
Ines Catry
Dario Cattaneo
Marco Cattaneo
Zaira Cattaneo
Nicole Cattano
Xavier Catteau
Luigi Cattivelli
Diego Cattoni
Lisa Caturegli
Christopher Caudill
Christelle Cauffiez

George Caughey
James Cauraugh
Ryan Causby
Claudia Regina Cavaglieri
Luca Cavagnaro
Larissa Maria Cavalcante
Ricardo Cavalcante
João Henrique Cavalcanti
Luciano Cavalcanti
Elisabetta Ada Cavalcanti-Adam
Francisca Cavaleiro
João Cavaleiro Rufo
Matteo Cavaliere
Marina Cavalieri
Vincenzo Cavalieri
L. H. Cavallari
Sebastiano Cavallaro
Laura Cavallero
Giulia Cavalli
Luciane Cavalli
Roberta Cavalli
Giorgio Cavallini
Andrea Cavanna
Vagner Cavarzere
Claudio Cavasotto
Andrea Cavazzoni
Claudio Cavazzuti
Kyle Cave
Nick Cave
Douglas Cavener
Michel Cavigelli
Sabri Cavkaytar
James F. Cawley
Jane Cawson
Remy Cazabet
Danielle Cazabon
Marine Cazenave
Silvia Cazorla
Valentina Cazzato
Dario Cazzola
Gabriel Cea
Michele Cea
Alejandro Ceballos
Hernan Ceballos
Juan Cebral
Ana Isabel Cebria
Gerardo Cebrian
Manuel Cebrian
Daniela Ceccarelli
F. Ceccarelli
Giancarlo Ceccarelli
Giulia Ceccherelli
Serena Cecchetti
Antonella Cecchettini
Giovanni Cecchetto
Rossana Cecchi
Erika Cecchin
Alessandra Cecchini
Hennessey Cecilia
Fabrizio Ceciliani
Nerry Cecilio
Rolando Ceddia
Arthur Cederbaum
Gustavo Cediel
Alessia Cedola
Susana Cedres
Angelo Cefalu
Wendy Cegielski
Lasse Cehofski
Isil Cekic-Nagas
Franco Celada
Antonio Celani
M. Emre Celebi
Alessia Celeghin
Soledad Celej
Mehmet Çelen
Mehmet Celenk
David Celentano
Mara Rúbia Celes
Benedetto Celesia
Pablo Celhay
Sebahattin Celik
Serdar Celik
Gonca Çelik
Yakup Celikbilek
Sergej Celikovsky
Bonnet Céline
Eleonora Cella

Jean Celli
Gilles Cellier
Connie Celum
Jaka Cemazar
Nicolas Cenac
Angela Cenci
Simone Cenci
Fernando Cendes
Maysa S. Cendoroglo
Can Cenik
Mari Carmen Cenit
Edina Cenko
Nunzio Cennamo
Rob Center
Javier Cepeda
Eugene Ceppa
Pasquale Cepparulo
Andrea Cerasuolo
Maria Ceravolo
Jorge Cerda
Artemi Cerdà
Joan Cerdà
Pablo Cerdán
Marney Cereda
Lage Cerenius
Paulo Ceresini
Idris Cerkez
Eren Çerman
Valeria Cernaro
Tomislav Cernava
Martin Cernvall
Jiry Cerny
Elizabeth Cerny-Chipman
Cheryl Cero
Andrea Ceron
Mauro Ceroni
Carlo Cerrano
Matteo Cerri
Paulo Cerri
Francesco Cerritelli
Laura Cersosimo
Gabriela Certad
Stefania Ceruti
Carlos Cervantes
Isabel Cervantes
Tiziana Cervelli
Adelheid Cerwenka
Piotr Ceryngier
Maria Florencia Cesani
Carina Cesar
Lima César F.
Thomas Cesario
Andrea Ceschi
Petr Česla
Elizabeth Cespedes Feliciano
Daniele Cespi
Raymond Cespuglio
Daniela Cesselli
Amedeo Cesta
Resul Cesur
Merih Cetinkaya
Enrico Cetrano
Catherine Cetre-Sossah
Melissa Ceuterick
Sergio Cevallos-Ferrizb
Lucia Cevidanes
Sabina Cevoli
Pieter-Jan Ceyssens
Sarah Ceyssens
Sudha Cg
Ratnasekhar Ch
Eugene Ch'Ng
Christine Cha
Dong Cha
Jeong-Heon Cha
Jung-Yul Cha
Seunghee Cha
Helmi Chaabène
Karima Chaabna
Chishala Chabala
Nzapfurundi Chabikuli
Aude Chabirand
Christopher Chabot
Lauren Chaby
Silvana Chacha
Elisabeth Chachaty
Bryce Chackerian
Carlos Chacon

Dustin Chacon
Marucia Chacur
Steven Chadban
Jayashree Chadchankar
Rakesh K. Chadda
Ahmad Chaddad
Alejandro Chade
Mandeep Chadha
V. Chadha
Elizabeth Chadwick
Kyle Chadwick
Jung-Il Chae
Sophia Chae
Suhn-Kee Chae
Benjamin Chaffee
Tyler Chafin
Jamie Chaft
Ana Cláudia Chagas de Paula
Frédéric Chagué
Harinder Chahal
Abdullah Chahin
Lana Chahine
Javaan Chahl
Jorge Chahla
Rakesh Chahota
Gaoshang Chai
Jianfang Chai
Jing Chai
Lijun Chai
Qiang Chai
Rifai Chai
Shauna-Lee Chai
Xinyu Chai
Yifeng Chai
Young Gyu Chai
Yu-Na Chai
Yunrong Chai
Lidya Chaidir
Leila Chaieb
Nils Chaillet
Anaïs Chailleux
Antoine Chaillon
Benny Chain
Kittipong Chaisiri
Vijender Chaitankar
Amitabh Chak
Adam Chaker
Aron Chakera
M. Chakhtoura
Murali Chakinala
Paul Antony Chakkiath
Alok Chakrabarti
Baidyanath Chakrabarti
Lisa Chakrabarti
Manoj Chakrabarti
Mrinmay Chakrabarti
Oishee Chakrabarti
Pinak Chakrabarti
Rahul Chakrabarti
Ratna Chakrabarti
Rina Chakrabarti
Sasanka Chakrabarti
Subhabrata Chakrabarti
Subrata Chakrabarti
Shantanu Chakrabartty
Pradip Chakraborti
Tapati Chakraborti
Arpita Chakraborty
Chandan Chakraborty
Nilanjan Chakraborty
Nilay Chakraborty
Nirali Chakraborty
Pranesh Chakraborty
Raja Chakraborty
Somsubhra Chakraborty
Sounak Chakraborty
Subrata Chakraborty
Supriya Chakraborty
Sutirtha Chakraborty
Trinad Chakraborty
Sudha Chakrapani
Venkatesan Chakrapani
Srinandan Chakravarthy
Ritu Chakravarti
Runu Chakravarty
Sumana Chakravarty
David Chakravorty
Somnath Chakravorty

Debanjan Chakroborty
Alcmène Chalazonitis
Vladislava Chalei
Roberto Chalela Rengifo
Lorraine Chalifour
Douglas Chalker
Roger Chalkley
Pratap Challa
Rajesh Challa
John Challis
Rosemary Challis
James Chalmers
Natalia Chalmers
Helena Chaloupková
Laura Chama
Jamshidkhan Chamani
Andrew Chamberlain
Catherine Chamberlain
Jon Chamberlain
Samuel Chamberlain
Maxime Chamberland
Jordan Chamberlin
Alison Chambers
Benedict Chambers
Charlotte Chambers
Christopher Chambers
Jeremy Chambers
Mark Chambers
Mary Chambers
Setsuko Chambers
Tamy Chambers
Gabriel Chamie
Dick Chamla
Larry Chamley
Carine Chamon
Daniel Chamorro
Benoit Champagne
Karen Champenois
Christophe Champod
David Champredon
Walee Chamulitrat
Agnes Chan
Amy Chan
Andrew Chan
Annie Chan
Antoni Chan
Cheong Xin Chan
Chi-Ming Chan
Christian Chan
Cliffton Chan
David Chan
Edward Chan
Elsa Chan
George H. M. Chan
Heng Chan
Hsun-Liang Chan
Jane Chan
Jasper Chan
Juliana Chan
Kei Hang Katie Chan
Keith C. C. Chan
Kevin Chan
Kung-Sik Chan
Lawrence Chan
Man Ngai Chan
Michael Chan
Ming-Cheng Chan
Patrick Chan
Pei-Ying Chan
Pek-Lan Chan
Philip Chan
Poemen Chan
Pui Ying Chan
Raquel Chan
Shu Jing Chan
Siu Ming Chan
Thomas Y. K. Chan
Timothy C. Y. Chan
Ting-Fung Chan
Wai Sum Chan
Wallace Chan
Wen-Ching Chan
Wing Chan
Wood Yee Chan
Y. Chan
Yat Hin Chan
Yee-Ming Chan
Zhulong Chan

Jacinta Chan Phooi M'ng
Vaibhav Chand
Sinha Chanda
Shweta Chandak
Hersh Chandarana
Ajit Chande
Anuj. K. Chandel
S. S. Chandel
Harish Chander
James Angus Chandler
Julia Chandler
Mark Chandler
Paula Chandler-Laney
Ramesh Chandolia
Satish Chandolu
Goutam Chandra
Joya Chandra
Nagasuma Chandra
Namas Chandra
Ronil Chandra
Subhash Chandra
Rajesh Chandramohanadas
Venkatraman Chandra-Mouli
Divya Chandran
Vinod Chandran
Pratik Chandrani
Pranantharthi Chandrasekar
Bharath Chandrasekaran
Natarajan Chandrasekaran
Nilmini Chandrasena
Arun Chandrashekar
S. Chandy
Robert Chaney
Guillaume Chanfreau
Andrew Chang
Anne Lynn Chang
Anne-Marie Chang
Charles Chang
Cheng Chang
Chen-Wei Chang
Chia Ming Chang
Chia-Chuan Chang
Chia-En Chang
Chi-Chang Chang
Chih Chang
Chih-Ching Chang
Chih-Hsiang Chang
Chih-Hung Chang
Chi-Ru Chang
Chiz-Tzung Chang
Chuen-Chau Chang
Chun Chang
Chun-Yen Chang
Darby Tien-Hao Chang
Dong-Jo Chang
Eric Chang
Esther Chang
Eugene Chang
Fengshui Chang
Franklin Chang
Guobin Chang
Hang Chang
Hsiao-Yun Chang
Hsing-Yi Chang
Huibin Chang
Hung-Chi Chang
Hyesang Chang
Jeng Chang
Jian Chang
Jinfeng Chang
Jun Chang
Jun-Dong Chang
Jung-Chen Chang
Kai-Hsiung Chang
Ken-Lin Chang
Ke-Vin Chang
Kuan-Cheng Chang
Kwanghyeon Chang
Kyong-Mi Chang
Larry Chang
Li-Hung Chang
Melinda Chang
Min Jung Chang
Ming-Ling Chang
Ming-Shi Chang
Moon Jong Chang
Nan-Shan Chang
Pei-Chann Chang

Peter Chang
Sandra Chang
Seon Hee Chang
Sha Chang
Shan Chang
Shang-Tzen Chang
Shirley Chang
Shunjen Chang
Song-Bin Chang
Stanley Chang
Steven Chang
Su-Chin Chang
Su-Hsin Chang
Suhua Chang
Sui-Yuan Chang
Sun-Young Chang
Suyin Chang
Theresa Chang
Ting-Tsung Chang
Tzu-Pu Chang
Ung Kyu Chang
Ung-Kyu Chang
Victor Chang
W. Chang
W. L. William Chang
Wei-Chiao Chang
Wei-Jen Chang
Weiping Chang
Wei-Tang Chang
Wei-Ting Chang
Wen-Chi Chang
Wen-Han Chang
Wen-Ruey Chang
Wen-Tsan Chang
Xiaohui Chang
Yao-Feng Chang
Yeun-Chung Chang
Yu-Fan Chang
Yuh-Shin Chang
Yu-Jen Chang
Yu-Sun Chang
Yu-Wei Chang
Zhihui Chang
Tang Changbing
Zhang Changhua
Du Changwen
Keith Channon
Gérald Chanques
Sunee Chansangpetch
Ekktet Chansue
Paul Chantler
Estelle Chanudet-van den Brick
Pithi Chanvorachote
Ariana Chao
Dennis Chao
Elizabeth Chao
Jie Chao
Lingling Chao
Nelson Chao
Yin Kai Chao
Ari Chaouat
Shashi Chaoudhary
Claudine Chaouiya
Durga Chapagain
Orlando Chaparro Garzón
Alain Chapel
Brian Chapin
Julius Chapiro
Christina Chapman
Ed Chapman
Elaine Chapman
Jason Chapman
Matthew Chapman
Nora Chapman
Sarah Chapman
Rene Chapot
Daniel Chappell
Joe Chappell
John Chappell
M. Chappell
Mark Chappell
Phoebe Chappell
Maryse Chappin
Vivianne Chappuis
Guillaume Chapron
Kevin Chapuis
Benoit Chaput
Brahim Chaqour

Rana Charafeddine
James Charbonneau
Catherine Charcosset
Karen Chardon
Alain Charest
Safa Charfeddine
Aswin Chari
Sudarshan Chari
A. Charidimou
Ratthaphol Charlermroj
Cyril Charles
Kenneth Charles
Nora Charles
Trevor Charles
Wisler Charles
Elin Charles-Edwards
Sonia Charleston-Villalobos
Keyne Charlot
John Charlson
W. Neil Charman
Phapanin Charoenphol
Helene Charreire
Remi Charrel
Marie-Therese Charreyre
Christiane Charriaut-Marlangue
Gregory Charrier
Leigh Charvet
Cathy Chase
P. Chase
Susan Chase
Alex Chaskopoulou
Benoit Chassaing
Kaywalee Chatdarong
Suman Chaterjee
Mark Chatfield
Sheryl Chatfield
John Chatham
Dan Chatman
Rabih Chattat
Katherine Chatten
Aniruddha Chatterjee
Anirvan Chatterjee
Aritrick Chatterjee
Arjun Chatterjee
Debanjana Chatterjee
Indira Chatterjee
Ishita Chatterjee
Kunal Chatterjee
Mitali Chatterjee
Nabanita Chatterjee
Nirmalya Chatterjee
Rima Chatterjee
Seshadri Sekhar Chatterjee
Soumya Chatterjee
Sudipto Chatterjee
Suvro Chatterjee
Victor Chatterjee
Minki Chatterji
James Chatters
Mary Lou Chatterton
Robert Chatterton
Siriporn Chattipakorn
Anshuman Chattopadhyay
Debasis Chattopadhyay
Pratip Chattopadhyay
Saurabh Chattopadhyay
Dhruba Chattoraj
Iti Chaturvedi
Sarika Chaturvedi
Sonali Chaturvedi
Sudha Chaturvedi
Ilenia Chatziandreou
Marios Chatzigeorgiou
Stylianos Chatzimanolis
Kimon Chatzistamatiou
Ioannis Chatzistefanou
K. Chatzistefanou
Yiannis Chatzizisis
Gar-Yang Chau
Gyaneshwer Chaubey
Bhupendra Chaudhary
Kumardeep Chaudhary
Raj Chaudhary
Sarika Chaudhary
Ujwal Chaudhary
G. Chaudhry
Shehzad Chaudhry
Ajay Chaudhuri

Minu Chaudhuri
Rishidev Chaudhuri
Zia Chaudhuri
Arun Chaudhury
Anil Chauhan
Kamlesh Chauhan
Narsigh Chauhan
Prakram Singh Chauhan
Santosh Chauhan
Subhash Chauhan
Boris Chaumette
Akhilanand Chaurasia
Stella Chaushu
Alienor Chauvenet
Sylvain Chauvette
Sai Chavala
Leonardo Chavane
Jean-Paul Chavas
Jerome Chave
Leonardo Chaves
M. Chaves
Óscar Chaves
Raquel Chaves
Francisco Chavez
Luis Chavez de Paz
Gilberto Chavez-Gris
Carlos Chavez-Olortegui
Mikulas Chavko
Suhash Chavva
Bhavna Chawla
Louise Chawla
Michael Chazan
M. Chazapis
Lixiao Che
Pulin Che
Charissa Cheah
Phaik Yeong Cheah
Yong Cheah
Mun Hong Cheang
Carol Cheatham
Tomas E. Cheatham
Jennifer Cheavens
Yassmine Chebaro
Daniel-Robert Chebat
Ricardo Chebel
Fouad Chebib
Antonio Checa
Sara Checa
Devon Check
David Checkley
Marcio Chedid
Frédéric Chédin
Grace Chee
Kee Chee Cheong
Amrita Cheema
Ian Cheeseman
Rosa Chefaoui
Hassib Chehade
Adam Chehouri
Ira Cheifetz
Anthony Cheke
Adam Chekroud
Claude Chelala
Cosimo Chelazzi
Ravi Chella
Kalaivani Chellappan
Giulia Cheloni
Baptiste Chemin
Matthieu Chemin
Jonathan Chemouny
Abel Chemura
Aizheng Chen
Alice Chen
Allen Chen
Aoju Chen
Bang-Bin Chen
Benjamin Chen
Bijun Chen
Bin Chen
Bing Chen
Biyu Chen
Bo Chen
Bolin Chen
Caiyan Chen
Celine Chen
Chang Chen
Chang-Hsun Chen
Chao Chen

Chaoyang Chen
Chao-Ying Chen
Chen Chen
Cheng-Sheng Chen
Cheng-Yu Chen
Chen-Yun Chen
Chi Chen
Chia-Jeng Chen
Chian-Feng Chen
Chi-Der Chen
Chien-Chou Chen
Chien-Chung Chen
Chien-Hung Chen
Chien-Liang Chen
Chien-Yi Chen
Chih-Yu Chen
Ching-Hsein Chen
Ching-Yuan Chen
Chi-Wen Chen
Christopher Chen
Chuan-Yu Chen
Chun Chen
Chun Chen Chen
Chun-An Chen
Chun-Chi Chen
Chunhua Chen
Chun-Li Chen
Chyi Chen
Cong Chen
Congying Chen
Cunjian Chen
Dahua Chen
Dandan Chen
Dar-Ren Chen
David Chen
Dayue Chen
Dechang Chen
Delphine Chen
Der-Yuan Chen
Dong Chen
Dongbao Chen
Dongmei Chen
Duan-Bing Chen
Edward Chen
Ee Sin Chen
Elizabeth Chen
Eric Chen
Faju Chen
Fa-Jun Chen
Fang Chen
Fanguo Chen
Feier Chen
Felicia Chen
Feng Chen
Gang Chen
Gaojian Chen
Geng Chen
George Chen
Grace Chen
Guan Chen
Guang Chen
Guangjie Chen
Guangsheng Chen
Guoan Chen
Guo-Qiang Chen
Guoxiong Chen
Guoxun Chen
Guo-Zhong Chen
Haijun Chen
Hai-Long Chen
Haiquan Chen
Hao Chen
Haodong Chen
Haoyu Chen
Harn-Shen Chen
Hexin Chen
Heyu Chen
Homer Chen
Hong Chen
Hongda Chen
Hongsong Chen
Hongwei Chen
Hou-Zao Chen
Hsin-Wei Chen
Hu Chen
Huabang Chen
Huai Chen
Huaiwen Chen

Huaiyong Chen
Huan Chen
Huey-Ling Chen
Huhui Chen
Hui Chen
Huichao Chen
Hui-Ling Chen
Huiqin Chen
Hui-Tzu Chen
Hung Chun Chen
Hung-Chi Chen
Hungwen Chen
Hung-Yu Chen
J. Chen
Jack Chen
James Chen
James M. Chen
Jason Chen
Jau-Yuan Chen
Jean Chen
Jeff Yi-Fu Chen
Jiandong Chen
Jiangang Chen
Jiang-Xing Chen
Jianhua Chen
Jiann-Chu Chen
Jianping Chen
Jianquan Chen
Jiansheng Chen
Jianwu Chen
Jianxin Chen
Jian-Ye Chen
Jiayu Chen
Jidang Chen
Jie Chen
Jieyin Chen
Ji-Hua Chen
Jin-Bor Chen
Jing Chen
Jitong Chen
Jiwang Chen
Juine-Ruey Chen
Julian Chen
Jun Chen
Junjie Chen
Jun-Qiang Chen
Jyh-Cheng Chen
Kai Chen
Kai-Yi Chen
Kang Chen
Ke-Neng Chen
Keping Chen
Keyang Chen
Kuan Chou Chen
Kuang-Chi Chen
Ku-Chung Chen
Kuei-Min Chen
Kun Chen
Kun-Ming Chen
Kuo-Hu Chen
Kuo-Yu Chen
Lanying Chen
Lee-Wei Chen
Lei Chen
Lei-Shih Chen
Li Chen
Liang Chen
Liang-Kung Chen
Lidian Chen
Lifei Chen
Lihong Chen
Li-Jun Chen
Li-Li Chen
Limei Chen
Limeng Chen
Limin Chen
Liming Chen
Lin Chen
Ling-Chia Chen
Linyi Chen
Li-Qing Chen
Li-Sheng Chen
Li-Song Chen
Li-Wei Chen
Long Chen
Long-Qi Chen
Mei-Jou Chen
Mengqian Chen

Michael Chen
Min Chen
Ming Chen
Minglong Chen
Ming-Shun Chen
Min-Huey Chen
Minyong Chen
Mo Chen
Nigel Chen
Ning Chen
Ning-Hung Chen
Oliver Chen
Pei-Lung Chen
Peng Chen
Peter Chen
Ping Chen
Ping-Jen Chen
Pin-Yu Chen
Po-Chi Chen
Po-Yueh Chen
Qi Chen
Qiang Chen
Qinghe Chen
Qinglin Chen
Qing-Xi Chen
Qiushi Chen
Qiusong Chen
Quan Chen
Qun Chen
Rita Chen
Ritchie Chen
Roger Chen
Ronald Chen
Rong Chen
Rong-Jane Chen
Rongsheng Chen
Ruei-Ming Chen
Ruey-Yu Chen
Senyu Chen
Shaojie Chen
Shaoxing Chen
Shao-Yu Chen
Sheng Chen
Shengdong Chen
Shih-Jen Chen
Shih-Kuo Chen
Shiou-Lan Chen
Shixi Chen
Shuai Chen
Shuangyan Chen
Shu-Chuan Chen
Shuiping Chen
Shuisen Chen
Shuo Chen
Shuyuan Chen
Shu-Zhen Chen
Siyu Chen
Songbi Chen
Songbiao Chen
Ssu-Han Chen
Suying Chen
Szu-Chia Chen
Szu-Tah Chen
Taolin Chen
Thomas Chen
Tian Chen
Tien-Hsing Chen
Trevor Chung-Ching Chen
Tsai-Yun Chen
Tse-Ching Chen
Tsong Chen
Tsung-Yu Chen
Vincent Chen
Vivian Hsueh Hua Chen
Wan Tao Chen
Wanping Chen
Wan-Qing Chen
Wei Chen
Wei R. Chen
Weihong Chen
Weile Chen
Wei-Ta Chen
Wei-Ting Chen
Weiwei Chen
Wen Chen
Wenhao Chen
Wenpin Chen
Wen-Shiang Chen

Wentao Chen
Xi Chen
Xiangdong Chen
Xiang-Jun Chen
Xiaobo Chen
Xiaochao Chen
Xiaodong Chen
Xiaofeng Chen
Xiaohu Chen
Xiaojie Chen
Xiao-Lin Chen
Xiaoming Chen
Xiaowu Chen
Xiaoxiang Chen
Xin Chen
Xin Jie Chen
Xinchun Chen
Xing Chen
Xingjuan Chen
Xinhua Chen
Xinjun Chen
Xinqiang Chen
Xiqun Chen
Xiyuan Chen
Xu Chen
Xuehao Chen
Xuelong Chen
Xueni Chen
Xuequn Chen
Xuhai Chen
Yajun Chen
Yan Chen
Yang Chen
YangQuan Chen
Yaw-Chung Chen
Ya-Whei Chen
Yee-Chun Chen
Yen-Fu Chen
Yen-Hao Chen
Yen-Tyng Chen
Yen-Yu Chen
Yet-Ran Chen
Yi Chen
Yi-Cheng Chen
Yi-Chuan Chen
Yi-Chun Chen
Yi-Guang Chen
Yiheng Chen
Yihua Chen
Yi-Min Chen
Yin Chen
Ying Chen
Ying-Ping Chen
Yi-Tien Chen
Yi-Wen Chen
Yixin Chen
Yixiong Chen
Yong Chen
Yongjun Chen
You Chen
Young-Mao Chen
Youxin Chen
Yu Chen
Yuanbo Chen
Yuche Chen
Yu-Cheng Chen
Yu-Ching Chen
Yu-Chun Chen
Yue-Hwa Chen
Yuhan Chen
Yuh-Lien Chen
Yun Chen
Yunching Chen
Yung Chen
Yunjie Chen
Yuping Chen
Yushu Chen
Yvonne Chen
Zhaowei Chen
Zhen Chen
Zheng Chen
Zhenlong Chen
Zhenyue Chen
Zhe-Sheng Chen
Zhichang Chen
Zhijian Chen
Zhiming Chen
Zhi-Nan Chen

Zhiyuan Chen
Zhong Chen
Zhonghua Chen
Zhongzhou Chen
Zhu Chen
Zhuo (Adam) Chen
Zigui Chen
Zijiang Chen
Zuozhou Chen
Ronald Chenail
Benoit Chenais
Benoît Chénais
Liu Chenchen
Huang Chencheng
Ezhilarasi Chendamarai
Waqtola Cheneke
Kate Cheney
Marshall Cheney
Paul Cheney
Andrew L. Cheng
An-Lin Cheng
Anny Cheng
Bin Cheng
Cheng-I Cheng
Chia-Hsiung Cheng
Chi-Hui Cheng
Chin Cheng
Ching-Yu Cheng
Chi-Tsun Cheng
Chun-Chia Cheng
Deborah Cheng
Dominic Cheng
Erdong Cheng
Fang-Min Cheng
Feng Cheng
Han Cheng
Hans Cheng
Heung-Chin Cheng
Hewei Cheng
Hong Cheng
Hui Cheng
Hung-Chi Cheng
I-Jiunn Cheng
Jason Chia-Hsien Cheng
Jia Cheng
Jin Cheng
Jin-Wei Cheng
Jiuqing Cheng
Jun Cheng
Kai-Chun Cheng
Ke Cheng
Kuan-Chen Cheng
Lei Cheng
Leo Cheng
Li Cheng
Liang Cheng
Lily Cheng
Lingyun Cheng
Liqin Cheng
Lisa Cheng
Louise Cheng
Lu Cheng
Ni Cheng
Oumei Cheng
Pin-Nan Cheng
Qiang Cheng
Qing Cheng
Robert Cheng
Rui Cheng
Shibin Cheng
Shih-Ping Cheng
Shu-Hsing Cheng
Tina Cheng
Tong Cheng
Tuck Seng Cheng
Xi Cheng
Xiaodong Cheng
Xiao-Wen Cheng
Xinlai Cheng
Xuanhong Cheng
Xue Cheng
Xuzhen Cheng
Y. Cheng
Ya-Jung Cheng
Yanwei Cheng
Yi-Bang Cheng
Yinhe Cheng
Yi-Sheng Cheng

Yong Cheng
Yongzhong Cheng
Yuanping Cheng
Yuanzhi Cheng
Yuchung Cheng
Yufeng Cheng
Yujie Cheng
Yuqi Cheng
Zhangrui Cheng
Zhiyong Cheng
Zhonghua Cheng
Zhongjian Cheng
Ziqiang Cheng
Zhang Chengcui
Ye Chen-Izu
Bruno Chenuel
Anne Chenuil
Bobby Cheon
Yong-Pil Cheon
Hui Ting Cheong
Siew Ann Cheong
Ying Cheong
Kartikeya Cherabuddi
Zahra Cheraghi
Bobby Cherayil
C. Cherbuy
Daniel Haile Chercos
Dmitry Cherepanov
Gita Cherian
Mathew Cherian
Erik Cheries
Ameur Cherif
Mehdi Cherif
Vino Cheriyan
Linda Cherkassky
Yijuang Chern
Natalie Chernets
Debbie Cherney
Jerry Cherney
Jonathan Chernoff
Ekaterina Chernyaeva
Anatoly Chernyshev
P. V. Chernyshov
Thomas G. V. Cherpanath
Thomas Cherpes
Gemma Cherry
James Cherry
Katie Cherry
Matthew Chersich
Mark Chertoff
Beata Chertok
Aravind Cherukuri
Peter Cherutich
Frank Chervenak
Inna Chervoneva
Arik Cheshin
Dana Chesney
Alessandro Chessa
Marie-Françoise Chesselet
Russell Chess-Williams
Adrian Chester
Douglas Chesters
Banthit Chetsawang
Candice Chetty-Makkan
Ellen Chetwynd
Alice Cheung
Allen Cheung
Ching-Lung Cheung
Chi-Wai Cheung
Chui Ming Gemmy Cheung
Gordon Cheung
Lydia Cheung
Margaret Cheung
Martin Cheung
Po-Yin Cheung
William Cheung
Wisit Cheungpasitporn
Bernadette Chevalier
M. Chevalier
Mathieu Chevalier
N. Chevalier
Timothy Chevassut
Christophe Chevillard
Anne-Marie Chèvre
Pascale Chevret
Emily Chew
Lock Chew
Deepak Chhabra

Puneet Chhabra
Prashant Chhajed
Alexander Chi
Congwu Chi
Hsin Chi
Iris Chi
Kai-Jung Chi
Lin-Yang Chi
Oak Chi
Sung-Do Chi
Tom Chi
Yuling Chi
Jean-San Chia
Nicholas Chia
F. Chiacchio
Ri-Cheng Chian
Chen-Yuan Chiang
Chien-Hsieh Chiang
Chun-Ju Chiang
Chun-Pin Chiang
John Chiang
Kai-Yang Chiang
Ming-Chang Chiang
Wen Chih Chiang
Yao-Chang Chiang
Yao-Yi Chiang
Yin-Ru Chiang
Yu-Chih Chiang
Yu-Chung Chiang
Yung-Hsiao Chiang
Maria Vincenza Chiantore
Mariachiara Chiantore
Elizabeth Chiao
Michela Chiappalone
Eugenia Chiappe
Annalisa Chiappella
Joshua Chiappelli
Ciro Chiappini
Franck Chiappini
Marilo Chiara
Matteo Chiara
Ferdinando Chiaradonna
Agostino Chiaravalloti
Laurent Roberto Chiarelli
Giuseppe Chiarenza
Mary Ann Chiasson
Giorgia Chiatante
Hirofumi Chiba
Sotaro Chiba
Dixon Chibanda
Adam Chicco
Laurent Chiche
Havovi Chichger
David Chichka
Alain Chichom-Mefire
R. Matthew Chico
Vidya Chidambaran
Ann Chidgey
Andy Chien
Cheng Yu Chien
Jung-Ting Chien
Ling-Chu Chien
Yin-Hsiu Chien
Yi-Wen Chien
Scott Chiesa
Francesca Chiesi
Munashe Chigerwe
An-Hsuan Chih
Maria Chikina
Michael Chikindas
Usuf Chikte
Robert Chilcott
Stephanie Child
Emma Childs
Gwen Childs
Yves Chilliard
Cristiane Chillof
John Chilton
Moses Chimbari
Isotta Chimenti
Rodney Chimner
Alvin Chin
Andrew Chin
Kelly Chin
Kok-Yong Chin
Josephat Chinawa
Zaida Chinchilla-Rodriguez
Vernon Chinchilli

Prinya Chindaprasirt
Leonid Chindelevitch
Shinung Ching
Hector Chinoy
Jugun Prakash Chinta
Sumana Chintalapudi
Orawee Chinthakanan
Cosmin Chintoan-Uta
Adriano Chiò
Susanna Chiocca
Carlo Chiorri
Giuseppe Chiossi
Pinwen Peter Chiou
Yi-You Chiou
Luca Chiovato
Enrico Chiovetto
Chiara Chiozzini
Brian Chiputwa
Monique Chireau
Giovanni Battista Chirico
Witness Chirinda
Robert Chirinko
Diana Chirinos
Mayel Chirinos
Maurizio Chiriva-Internati
Carmelo Chisari
Caroline Chisenga
Andrew Chisholm
Mohammod Chisti
Walter Chitarra
Kenny Chitcholtan
Alexandra Chittka
Anitha Chitturi
Daniel Chitwood
Albert Chiu
Chih-Chiang Chiu
Chung-Yi Chiu
Han-Mo Chiu
Hao Chiu
Hui-Wen Chiu
Joanna Chiu
Kuo-Hsun Chiu
Liang-Da Chiu
Li-Chung Chiu
Nai-Chi Chiu
Nan-Tsing Chiu
Pei Chiu
Philip Chiu
Wilson Chiu
Yen-Cheng Chiu
Yuan-Shyi Peter Chiu
Miguel Chiurillo
Meredith Chivers
Venkat Keshav Chivukula
Faraja Chiwanga
Remigius Chizzola
Katerina Chlichlia
Gail Chmura
Wee Joo Chng
Arthur Cho
Byungchul Cho
Chunghee Cho
Clara Cho
Dong-Hyung Cho
Eun Ju Cho
Hyunsan Cho
Michael Cho
Mi-La Cho
Moonjae Cho
Nam Hoon Cho
Seungryong Cho
Sihyun Cho
Sohee Cho
Ssang-Goo Cho
Sungchan Cho
Yonglae Cho
Zhang-He Cho
Koh Cho Yeow
Keith Choate
Nachiappan Chockalingam
James Chodosh
Keith Choe
Christiane Borges do Nascimento
Chofakian
Bong-Kyu Choi
Bum-Rak Choi
Chang Won Choi
David Choi

Donghoon Choi
Dongseok Choi
Dongsu Choi
Eue-Keun Choi
H. Choi
Horace Choi
Hyong Woo Choi
Hyung-Kyoon Choi
Hyunmi Choi
Inchul Choi
Inho Choi
Ji Woong Choi
Jin A. Choi
Jin Young Choi
Jin-Ho Choi
Jongmoo Choi
Joong Sub Choi
Kwang-Wook Choi
Kyuha Choi
Marcelo Choi
Mi-Kyeong Choi
Min-Ho Choi
S. W. Choi
Sang-Bong Choi
Seul Choi
Soyoung Choi
Tae Young Choi
Tae-Jin Choi
Tammie Choi
William Choi
Wonil Choi
Won-Il Choi
Woonsup Choi
Yang Do Choi
Yong Seok Choi
Young Ki Choi
Young-Jun Choi
Youngshim Choi
Yu Suk Choi
David Chojnacky
Rubens Chojniak
Augustine Choko
Sylvie Chokron
Richard Chole
Frank Cholette
Joana Cholin
Sylvie Chollet
Evangelos Cholongitas
Michael Choma
Solomon Simon Ramphai Choma
Guillaume Chomicki
Jacques Chomilier
Andrea Chomistek
Niki Chondrogianni
Maria Chondronikola
Jo Chong
Karen Chong
Nelson Chong
Karen Chong-Seng
Esther Choo
Su-Pin Choo
Mahesh Choolani
Krongkarn Chootip
Lisa Chopin
Louise Chopinet
Meheriar Chopra
Rajiv Chopra
Jessica Chopyk
Nikos Chorianopoulos
Elizabeth Chornesky
J. Chorostowska
Alex Chortos
Kesinee Chotivanich
Suthat Chottanapund
An-Kuo Chou
Cheng-Yang Chou
Che-Yi Chou
Chi-Chung Chou
Chien-Chen Chou
Hung-Chieh Chou
James Chou
Joshua Chou
Kang-Ju Chou
Kun-Hsien Chou
Kun-Ta Chou
Lien-Siang Chou
Li-Wei Chou
Michelle Chou

Mitch Chou
Pei-Hsin Chou
Shih-Wei Chou
Tzu-Chieh Chou
Wen-Chi Chou
Y. H. Chou
Ya-Chang Chou
Yi-Yu Chou
Hichem Chouayekh
Divaker Choubey
Ruplal Choudhary
Mashkoor Choudhry
Soumitra Choudhuri
Anindo Choudhury
Moumita Choudhury
Naseem Choudhury
Ramprasad Choudhury
Robin Choudhury
Haroon Choudry
Manil Chouhan
Abderrahim Choukchou-Braham
Valerie Choumet
Danai Chourmouzi
Kapil Chousalkar
Eric Chow
Joan Chow
Ka Lai Chow
Keng-See Chow
Louis Chow
Tam-Lin Chow
Shibashish Chowdhary
Fazle Chowdhury
Kaushik Roy Chowdhury
Mahbub Chowdhury
Muhammad Abdul Baker Chowdhury
Nityananda Chowdhury
Raeed Chowdhury
Rajdeep Chowdhury
Samik Chowdhury
Sanjib Chowdhury
Sona Chowdhury
Souma Chowdhury
Soumitra Chowdhury
Sukanta Chowdhury
Tamjid Chowdhury
Vishwajit Chowdhury
Gerardo Chowell
Patricia Chow-Fraser
Wing-Yiu Choy
Mohcine Chraibi
Elizabeth Chrastil
Matthew Chrisman
Jonathan Chrispin
Sophocles Chrissobolis
Alexander Christ
Gregor Christa
Efterpi Christaki
Eirini Christaki
Sylvia Christakos
Cederberg Christel
Beat Christen
Nicholas Christenfeld
Daniel Christensen
Gitte Hedermann Christensen
Hannah Christensen
Henrik Christensen
Janne Christensen
John Christensen
Jørn Christensen
Joseph Christensen
Kenneth Christensen
Neil Christensen
Cory Christenson
Henrik Christesen
Daan Christiaens
Olivier Christiaens
Michael Christian
Sherri Christian
Richard Christiana
Blaine Christiansen
Bo Christiansen
Casper Christiansen
Christian Christiansen
Gunna Christiansen
Heather Christiansen
Soren Christiansen
Julie Christianson
Graham Christie

Nicola Christie
Jw Christle
Cynthia Christman
Gregory Christman
Olivier Christmann
Colleen Christmas
Matthew Christmas
Nicholas Christodoulides
Dimitrios Christodoulou
Diego Christofaro
Vincent Christoffels
Michael Christoffers
Thea Christoffersen
Nicola Christofides
Jan Christoph
Lay Christophe
Kenneth Christopher
Seldev Christopher
Christopher Fenno Christopher Fenno
Richard Christopherson
George I. Christopoulos
Vasileios Christopoulos
Ivaylo I. Christov
Petya Christova
Leonardo Christov-Moore
Annette Christy
Dorota Chrobak
Michael Chrobok
Pawel Chrom
Veronika Chromikova
Zissis Chroneos
Angeliki Chroni
George Chrousos
Alyson Chroust
Maksymilian Chruszcz
Hamdi Chtourou
Amanda Chu
Anne Chu
C. H. Chu
Chengjin Chu
Daniel Chu
Fang-Hua Chu
Hiutung Chu
In-Sun Chu
Jackson Chu
Jhih-Wei Chu
Ka Hou Chu
Kon Chu
Min Kyung Chu
Mingxing Chu
Wenying Chu
Wuying Chu
Xi Chu
Xiang-Ping Chu
Xiaogang Chu
Yiwei Chu
Zhaoqing Chu
Arlene Chua
Jacqueline Chua
Jennifer Chua
Jeroen Chua
Marcus Chua
Melvin L. K. Chua
Melvin Lee Kiang Chua
Sharon Chua
Streamson Chua
Seng-Kee Chuah
Swee-Hoon Chuah
Ts Chuah
Jiin-Haur Chuang
Kai-Hsiang Chuang
Kuo-Hsiang Chuang
Ming-Chin Chuang
Ming-Feng Chuang
Pao-Tien Chuang
Shuang-En Chuang
Shu-Chun Chuang
Wen-Po Chuang
Doan Huu Chuc
Anderly Chueh
Sumant Chugh
Celine Chui
Karin Chumbimuni-Torres
Kyung Ah Chun
Liu Chun
Sung Wook Chun
Tae-Hwa Chun
Rumi Chunara

Brian Chung
Chee Yeun Chung
Cheng-Shiu Chung
Chih-Ping Chung
Dai Chung
Emma Chung
Eun Jee Chung
Frances Chung
Gyuhwa Chung
Hee Jung Chung
Hsiao-Wen Chung
Jay Chung
Jayong Chung
Jen-Ping Chung
Jin-Wook Chung
Jong Woo Chung
Kevin Chung
Kian Chung
Moo Chung
Peter Chung
Raymond C. K. Chung
Raymond Chung
Ren-Hua Chung
Seon-Yoon Chung
Seung Ho Chung
Soonkyu Chung
Sung Wook Chung
Tae-Wook Chung
Taijoon Chung
Wen-Yu Chung
Yong Eun Chung
Young-Hwa Chung
Cecilia Chung-Nakandakari
Cheng-Ming Chuong
Anna Chuprin
Austin Church
Tyler Churchward-Venne
Daniel Churgin
Wanlop Chutipong
Michael Chvanov
André Chwalibog
Charng-Cherng Chyau
Igor J. Chybicki
Lily Chylek
Maciej Chylenski
Marcello Ciaccio
Laura Ciaffi
Lynn Cialdella-Kam
Giovanni Luca Ciampaglia
Michael Ciancanelli
Aurelio Ciancio
George Cianciolo
Giuseppe Cianciolo
Renaud Ciappuccini
Antonella Ciaramella
Gianfranco Ciaramitaro
Andrea Ciaranello
Constance Ciaudo
Fabio Cibella
Jolanda Cibere
Merih Cibis
Cinzia Ciccacci
Maria Ciccarelli
Renata Ciccarelli
Francesco Ciccia
Marco Cicciù
Marco Ciccone
Patrick Ciccotto
Kerim Cicek
Fabio Ciceri
Arrigo Cicero
Theodore Cicero
Giancarlo Cicolini
Giuseppe Ciconte
Flavia Cicuttini
Christopher Cielo
Izabela Ciesielska-Wróbel
Kristina Ciesielski
Małgorzata Cieśla
Adam Cieslak
Richard Cifelli
Ignacio Cifre
David Cifu
Víctor Cifuentes
Secundino. Cigarran Guldris
Roberto Cilia
Andrea Ciliberto
Corrado Cilio

Gustavo Cilla
Anthony Cillo
Catia Cilloniz
Giulia Cimarelli
Thomas Cimato
Mario Cimino
Megan Cimino
Ruben Cimino
Amelia Cimmino
Anca Maria Cimpean
Ali Cinar
Resat Cinar
Luca Cindolo
Tereza Cindrova-Davies
Scott Cinel
Antonella Cingolani
Gino Cingolani
Mathieu Cinier
Gilda Cinnella
E. Cinosi
Michela Cinquini
Luciano Cintra
Luca Cioccolanti
Patricia A. Cioe
Iacopo Cioffi
Jessica Cioffi
Marcelo Cioffi
Luminita Ciolacu
Erika Cione
Matteo Cioni
Alexander Ciota
Ana Cipak Gasparovic
Battal Çıplak
Marilyn Cipolla
Carlo Cipolli
Angela Cipollone
Giorgio Ciprandi
Andrea Cipriani
Paolo Cipriani
Matheus Cipriano
Laura Cirelli
Samuel Cires
Alessandro Ciresi
Rastko Ciric
Daniela Cirillo
Giovanni Cirillo
John Cirillo
Ivana Cirkovic
Tanja Cirkovic Velickovic
David Ciro
Duško Ćirović
Elizabeth Cirulli
Francesca Cirulli
Gerardo Cisneros
José Miguel Cisneros
Guéladio Cissé
Daniel Cisterna
Luca Cistrone
Daniel Citron
Francesca Citron
Antonio Cittadini
Christine Citti
Michelle Ciucci
Paolo Ciucci
Philippe Ciuciu
Marina Ciuffo
Stefano Ciurli
Anca Ciurte
Simone Ciuti
Pinar Civicioglu
Nico Claassens
Kasey Claborn
Beatrice Clack
Kasper Claes
Peter Claes
Frank Claessens
Lena Claesson-Welsh
Jérôme Clain
Audrey Claing
Claire Claire Terhune
Jesse Clanton
Thomas Clanton
Carmen Clapp
Elizabeth Clare
Jordi Clarimon
Abbot Clark
Alice Clark
Alys Clark

Andrew Clark
Anne B. Clark
Bronwyn Clark
C. Graham Clark
Carolyn Clark
Chase Clark
Clifford Clark
David Clark
Deborah Clark
Ian Clark
Imogen Clark
J. Alan Clark
James Clark
Jeffrey Clark
Jennifer Clark
Jonathan Clark
Julie Clark
Kim Clark
Meredith Clark
Nathan Clark
Ruth Clark
Sam Clark
Samuel Clark
William Clark
Laine Clark-Balzan
Anthony Clarke
Caroline Clarke
Christopher Clarke
Damian Clarke
David Clarke
Duncan Clarke
Emily Clarke
John Clarke
Laurence J. Clarke
Naomi Clarke
Sharon Clarke
Andrew Clarkson
Jenny Clarkson
Rafael Claro
M. Gonzalo Claros Diaz
Philip Clatworthy
Erika Claud
Julien Claude
Thierry Claudel
Joachim Claudet
Pier Claudio
Pioli Claudio
Sylvio Claudio-Neto
Ilene Claudius
Rolando Claure-Del Granado
Fredrik Clausen
Louise Clausen
Eric Clauser
Sylvain Clauser
Czerny Claus-Peter
Marcus Clauss
Matthias Clauss
Simon Clavagnier
Alexandra Clavarino
Marie-Annick Clavel
Jean-Michel Claverie
Katrina Claw
Gary Clawson
Michael Clawson
Alex Claxton
Keith Clay
Nicole Clay
John Claydon
Nicholas Claydon
Marla L. Clayman
Christine Clayton
Janine Austin Clayton
P. Clayton
Jane Cleal
Sophie Cleanthous
Christine Cleghorn
John Cleland
James Clelland
Mark Clemens
Christophe Clement
Deanna Clement
Nathan Clement
Romain Clement
Yves Clément
Christofer Clemente
Diego Clemente
Filipe Clemente
Maria Clemente

Nicolò Clemente
Maysa Clementino
C. Clements
Dennis Clements
Dylan Clements
Judith Clements
Meredith Clements
Robert Clements
Francesco Clemenza
Damien Cleret
Aldo Clerico
Justine Cléry
Beth Cleveland
Anthony Clevenger
Dylan Cliff
G. D. Clifford
Gari Clifford
Gary Clifford
Susan Clifford
Martin Clift
Andreu Climent
Brandon H. Cline
Christopher Cline
Peta Clode
Gisele Clofent-Sanchez
Alex Clop
Daniel Closa
Sergi Clotet
Geoff Cloud
Helen Clough
James Clough
Kate Clouse
Ronald Clouse
Guy Cloutier
Jasmin Cloutier
Robert Clubb
Kim Cluff
Robin Clugston
Nathan Clumeck
Laura Clusa
William Clusin
Marlon Coan
Nicolas Coant
Thomas Coate
Brad Coates
James Coates
Brittany Coats
Joel Coats
Joshua Cobb
Sarah Cobey
James Cobley
Teresa Cobo
Martyn Cobourne
Justine Coburn
Lori Coburn
Natalie Coburn
Phillip Coburn
Eliana Coccia
Christoph Coch
Blake Cochran
Gerald Cochran
James Cochran
Jesse Cochran
Kevin Cochran
Kyla Cochrane
Silvia Cocito
Ian Cock
Brian Cockburn
Ian Cockburn
Anne Cockcroft
Charles Cockell
Fiona Cocker
Paul Cockwell
Sergio Cocozza
Giuseppe Cocuzza
Jonathan Codd
Terence Coderre
Jordi Codony
Robert Coe
Denis A. Coelho
Gilberto Coelho
Lara Coelho
Marcel Coelho
Nuno Coelho
Manuel Coelho-E-Silva
Otavio Coelho-Filho
Tatiana Coelho-Sampaio
Yann Coello

Marieke Coenen
Pieter Coenen
Volker Coenen
Ricardo Coentre
Bronwyne Coetzee
Lindi-Marie Coetzee
Martin Coetzee
Maureen Coetzee
Vinet Coetzee
David Coeurjolly
John Coffey
M. Justin Coffey
Sean Coffey
James Coffman
Giancarlo Coghe
Massimo Cogliati
Anthony Cognato
Annalisa Cogo
Alasdair Cohen
Alicia Cohen
Amy Cohen
Andrew Cohen
Anna Cohen
Arnon Cohen
Bevin Cohen
Camille Cohen
David Cohen
Débora Cohen
Elizabeth Cohen
Jessica Cohen
Jonathan Cohen
Jonathon Cohen
Jordana Cohen
Joseph Paul Cohen
Joshua Cohen
Juliana Cohen
Juliana F. W. Cohen
Justus Cohen
Karen Cohen
Lauren Cohen
Michael Cohen
Mickaël M. Cohen
Mitchell Cohen
Myron Cohen
Netta Cohen
Nir Cohen
Noah Cohen
Oded Cohen
Philippa Cohen
Rebecca Cohen
Robert Cohen
Ronald Cohen
Sarah Cohen
Ted Cohen
Yigal Cohen
Zoe Cohen
Aaron Cohen-Gadol
Wendie Cohick
Claudia Cohn
Jeffrey Cohn
Lauren Cohn
Neil Cohn
Raul Coimbra
Frederic Coin
Patrick Coit
Ana Coito
Ann Coker
Darren Coker
Rogério Colaço
Stefano Colagrande
Cemil Colak
Serdar Colak
Tugba Colak
Paolo Colantonio
Elizabeth Colantuoni
Luc Colas
Joe Colasanti
Robert Colautti
Stefano Colazza
Nathalie Colbach
Angela Colbers
Jim Colbert
Tim Colbourn
Fred Colbourne
Robb Colbrunn
Jessica L. Colburn
Jeffrey Colby
M. A. Colchero

Teodora Emilia Coldea
Ian Colditz
Alexander Cole
Alison Cole
Jacqueline Cole
James Cole
Kelly Cole
Kimberly Cole
Megan Cole
Selina R. Cole
Stephen Cole
Tim Cole
Victoria Cole
James Colebatch
Esi Colecraft
Annette Coleman
Cynthia Coleman
David Coleman
Heather Coleman
Jenell Coleman
Jessica Coleman
Kim Coleman
Leon Coleman
Nichola Coleman
Nicholas Coleman
Richard Coleman
Stephen Coleman
Devin Coleman-Derr
Pietro Coletti
Niall Colgan
Lejla Colic
Andrew Colin
Sean Colin
Monica Colitti
Olympia Colizoli
Enrico Sandro Colizzi
Mar Coll
Aurélie Collado
Anna Collar
Rose Collard
Gabriel Coll-de-Tuero
Bill Colledge
Flora Colledge
Hilary Coller
Heidi Colleran
Bertrand Collet
Gavin Collett
Jason Collett
Matthew Collett
Tom Collett
Rosane Collevatti
Helen Colley
Andrea Colli
Licia Colli
Susanna Colli
David Colliaux
Alex Collie
Glen Collier
Lara Collier
Anne Collignon
Bertrand Collignon
Peter Collignon
Anne Collin
Peter Collings
Paul Collini
Alison Collins
Allen Collins
Anne Collins
Catherine Collins
Cg Collins
Colin Collins
Hal Collins
James Collins
Jeannie Collins
Kelsey Collins
Liam Collins
Marissa Collins
Natalie Collins
Nick Collins
Patricia Collins
Richard Collins
Samuel Collins
Steven Collins
Mark Collinson
Robert Collinson
Luana Colloca
Nathalie Colloc'h
Nicolas Collongues

Michael Collyer
Ben Colman
Ewan Colman
Jordi Colmenero
Lotte Colmorn
Celine Colnot
Holly Colognato
Claudia Colomba
Ds Colombari
Daniele Colombaroli
Marina Colombi
Lucio Colombi Ciacchi
Ana Paula Colombo
Barbara Colombo
Emanuela Colombo
Maria Laura Colombo
Matteo Colombo
Sonia Colombo
Wilfredo Colon
Fabiola Colone
Felipe Colón-González
Bianca Colonna
Giuliano Colosimo
Vittoria Colotta
Tonya Colpitts
David Colquhoun
Thomas Colquhoun
Gregory Colson
Natalie Colson
Erminia Colucci
Emma Colucci-Guyon
Christopher Colwell
Edoardo Colzani
Manuel Comabella
Josielli Comachio
Iñaki Comas
Mercè Comas
Yves Combarnous
Christian Combe
Jean-Damien Combes
Hendrik Combrink
Luc Comeau
Joan Comella
Alejandro Comellas
Iain Comerford
Nicholas Comerford
Paul Comfort
Giancarlo Comi
Giuseppe Comi
Hannie Comijs
Cinzia Comino
Pierre Comizzoli
Laura Comley
Ulrich Commandeur
Róisín Commane
Yvonne Commodore-Mensah
John Common
Didier Communi
Concetta Compagno
Susan Compton
Tanya Compton
Wilson M. Compton
Iulia Comsa
Philippe Comtois
Warren Comulada
Mary Ann Comunale
Clara Comuzzi
Kevin Conallen
Gavin Conant
Katherine Conant
Fabricio Conceição
Katia Conceição
Teresa Conceição
Beatrice Concepcion
Luis Concepcion
Lucie Conchou
Daniela Concolino
Jean-Paul Concordet
Laurent Condat
Artur Conde
Bruno Conde
João Conde
Renaud Conde
Silvia Conde
Miguel Ángel Conde González
John Condeelis
Giancarlo Condello
Guy Condemine

Lea Condon
Carles Conejero
J. Alberto Conejero
David Conesa
Anthony Confer
Anthony Confera
Renata Conforti
Lin Cong
Rihuan Cong
Xiaomei Cong
Yingzi Cong
Scott Conger
Leonardo Congiu
Emilio Congregado
Alice Conigliaro
Salvatore Coniglio
Rudi Coninx
Annalijn Conklin
Andrew Conlan
Dalton Conley
Terri Conley
Elizabeth Conlon
Michael Conlon
Thomas Conlon
John Conly
Cath Conn
Jan Conn
Kristen Conn
David Connell
Paul Connell
Sean Connell
Terry Connell
Kim Connelly
Linda Connelly
Alex Conner
Caitlin Conner
Mark Conner
Melinda Conners
Grant Connette
Andrew Connolly
Chris Connolly
Christopher Connolly
Deirdre Connolly
Eric Connolly
Thomas Connolly
E. Sander Connolly Jr.
Erin Connor
Jason Connor
Thomas H. Connor
Lawreen Connors
Steve Conolly
Cheryl Conover
Claudius Conrad
Daniel Conrad
Kirk Conrad
Steven Conrad
Barbara Conradt
Franz Conraths
Amy Conroy
Andrea Conroy
Robert Considine
Paul Consigny
Massimiliano Conson
Andrew Constable
Christian Constán-Aguilar
Daniel-Eduard Constantin
Niel Constantine
Stefan Constantinescu
Christos Constantinidis
Paul Constantino
Anthony Constantinou
Annamaria Conte
Maria Conte
Pierfranco Conte
Alfredo Conti
Luciano Conti
Barbara Contiero
Cristina Contreras
G. Andres Contreras
Lydia Contreras
David Contreras-Loya
Jose Clemente Contreras-Naranjo
Atilano Contreras-Ramos
Jose Contreras-Vidal
Philippe Conus
Gabriel Converse
Matteo Convertino
Baqiyyah Conway

Jessica Conway
Kevin Conway
Paul Conway
Tenley Conway
Klaus Conzelmann
Andrew Coogan
Alex Cook
Atlanta Cook
Bryan Cook
Chad Cook
Chelsea Cook
Dane Cook
Elizabeth Cook
Gary Cook
James Cook
Joe Cook
Leah Cook
Lyn Cook
Mark Cook
Samantha Cook
Martin Cooke
Matt Cooke
R. Cooke
Jessica Cooke Bailey
Amanda Cooklin
David Cool
Crystal Coolbaugh
Brian C. Cooley
Marion Cooley
Sarah Cooley
Skye Cooley
Tim Coolong
Nathalie Cools
Piet Cools
Roshan Cools
Brooke Coombes
Jason Coombes
Stephen Coombes
Matthew Coombs
Robert Coombs
David Coomes
David Coope
Andrea Cooper
Antony Cooper
Chris Cooper
Claudia Cooper
Curtis Cooper
Cynthia Cooper
David Cooper
Diane Cooper
Duncan Cooper
Eve Cooper
Geoffrey Cooper
George Cooper
James Cooper
Laurel Cooper
Lee Cooper
Nicholas Cooper
Paul Cooper
Richard Cooper
Robin Cooper
Sue Cooper
Trevor Cooper
W. Cooper
Ziva Cooper
Johnathan Cooper-Knock
Craig Coopersmith
Alex Cope
Andrew Cope
Vicki Cope
Kirstie Cope-Farrar
Brittney Copeland
John Copeland
Laurel Copeland
Les Copeland
Diego Copetti
Tanya Copley
Calina Copos
Stacy Copp
Isabelle Coppens
Michiel Coppens
Alan Copperman
Elisabetta Coppi
Ken Coppieters
Mauricio Coppo
Giangennaro Coppola
Mariangela Coppola
Jacques-Olivier Coq

Teresa Coque
Raildo Coqueiro
Daniele Coraci
Ramón Mauricio Coral Vázquez
Giuseppe Coratella
Kitty Corbet
Alexandra Corbett
Amanda Corbett
Bradley Corbett
John Corbett
Antoine Corbin
Joseph Corbo
Jason Corburn
Vanessa Corby-Harris
Nicolae Corcionivoschi
Brandan Corcoran
Cheryl Corcoran
Cm Corcoran
Michael Corcoran
Marcos Cordeiro
Yraima Cordeiro
Ann Cordell
Susan Cordell
Francesca Cordella
Paul Cordero
Radames Cordero
Jose Manuel Cordero-Ferrera
Adolfo Cordero-Rivera
Erik Cordes
Jose Cordoba-Chacon
Giada Cordoni
Rita Cordovil
Cynthia Core
Enoka Corea
David Corey
Lawrence Corey
Antonio Corgos
Marie France Corio Costet
Harold Corke
Ammon Corl
Alexandru Corlan
A. Corlateanu
Philip Corlett
Anne Corlu
Brendan Cormack
Steven Corman
Victor Corman
Gabriela Cormick
David Cormode
Mireille Cormont
Alyssa Cornall
Brian Corneil
Carlos Cornejo
Bruno Cornelis
Elise Cornelis
Marilyn Cornelis
Pierre Cornelis
Christopher Cornelison
Dawn Cornelison
Gert Cornelissen
Laura Cornelissen
Marion Cornelissen
Piers Cornelissen
Robin Cornelissen
Laura Cornelsen
Francois Cornet
Jennifer Cornick
Charlotte Cornil
Emmanuel Cornillot
Astrid Cornils
Erwin Cornips
Elisa Cornish
Peter Cornish
Rosie Cornish
Athel Cornish-Bowden
Christopher Cornwall
Thibaud Coroller
Angela Corona
Benjamin Corona
Giovanni Corona
Miguel Corona
Eva Corpeleijn
Sinead Corr
Francesco Corradi
Nicola Corradi
Flavio Corradini
Chiara Corrado
Pierre Corre

Edgar Correa
Alberto Corrêa
Franciso Correa-Aranada
Carlos Corredor
Bruno Correia
Manuela Correia
Ricardo Correia
Stephen Correia
Vanda Correia
Sara Correia Carreira
Maria Correia de Oliveira
Margarida Correia-Neves
André Correia-Santos
Xavier Correig
Carl Correll
Simon Corrie
Matthew Corriere
Giuseppe Corriero
Colleen Corrigan
Jay Corrigan
Luis Corrochano
Sherryse Corrow
Ben Corry
Maria Salvatore Corsello
Daniel J. Corsi
Betina Corsico
Gaetano Corso
Simonetta Corsolini
Guilherme Corte
Andrea Cortegiani
Claudia Cortes
Claudio Cortes
Daniel Cortes
Yamnia Cortes
Jorge Cortés
Liliana Cortés
M. C. Côrtes
E. Cortés-Castell
Antonio Cortese
Samuele Cortese
Michael Cortez
Manuela Corti
Olga Corti
Lilia Cortina
Soledad Cortina
Cristina Cortis
J. Corton
Gino Cortopassi
Stephane Corvec
Maria Corvi
Gerardo Corzo
S. Cosby
Carmine Coscia
Michele Coscia
Theodore Cosco
Theodore D. Cosco
Sandro Cosconati
Alessandro Cosentino
Claudia Cosio
Ahmet Coskun
Devrim Coskun
Alina Paula Cosma
Marco Cosme
Benilde Cosmi
Erich Cosmi
Lorenzo Cosmi
Andrea Cossarizza
Stephanie Cossette
Jacky Cosson
Davide Cossu
Albert Costa
Aldo Costa
Alex Costa
Amine Farias Costa
Angelo Brandelli Costa
Anna Costa
Barbara Costa
Catarina Costa
Fabrizio Costa
Felipe Costa
Fernando Costa
Francesco Costa
Hugo Costa
Joana Costa
Joao Costa
Leodante Costa
Luis Costa
Madalena Costa

Manuela Costa
Marcello Costa
Marcos Costa
Mário Costa
Paulo Costa
Pedro Costa
Robson Costa
Rúben Costa
Rui Costa
Sandra Costa
Sofia Costa
Tommaso Costa
Valerio Costa
Vitor Costa
Wilson Costa
Luiz Costa E Silva
Pedro Costa Lima
Miguel Costa-Gomes
Leticia Costa-Lotufo
Carlo Costantini
Federica Costantini
Maria Costantini
Susan Costantini
Todd Costantini
Robert Costanza
Andrea Costanzo
Erin Costanzo
Paola Costanzo
Pierluigi Costanzo
Raul Costa-Pereira
Rodrigo Costas Comesaña
João Henrique Costa-Silva
Bertrand Coste
Paola Costelli
James Costello
Kaitlin Costello
Mark Costello
Richard Costello
Kelly Costello Allison
Wendy Coster
Loïc Costeur
Maria Costi
Xurxo Costoya
Victor Coswig
Michel Cot
Luís Cota
Douglas Cotanche
Mary Frances Cotch
Kimberly Cote
Richard Cote
Francine Côté
Isabelle Côté
E. Cothran
Justin Cotney
Antonella Cotoia
Cheryl Cott
Gianluca Cotta Ramusino
Laurent Cotte
Aoife Cotter
James Cotter
Peggy Cotter
Elisabetta Cotti
Alberto Cottica
Fabien Cottier
François Cottin
Clémentine Cottineau
Bridget Cotti-Rausch
Linda Cottler
Jennifer Cotton
John Cotton
Pietro Cottone
Emmanuel Couacy-Hymann
Claire Couch
Yvonne Couch
John Couchman
Cecile Couchoud
Evelyne Coudrier
R. Coudroy
William Couet
Brian Couger
Christina Coughlan
Lynda Coughlan
Melinda Coughlan
Celine Cougoule
Jennifer Coull
Sion Coulman
Graeme Coulson
Vivien Coulson-Thomas

Xavier Coumoul
Jacqueline Counotte
Brian Counterman
Scott Counts
David Couper
Kevin Couper
Gwennou Coupier
Carol Coupland
Christian Couppe
Elliot Coups
P. Courand
Paul Courant
Alexis Courbet
François Courchesne
Albert Courey
Valerie Courgnaud
Sylvie Coursol
Donald Court
Gaston Courtade
Thibault Courtheoux
Jesse Courtier
Emmanuelle Courtiol
Elodie Courtois
Jose Courty
Aurélien Courvoisier
Helene Courvoisier
Peter Cousins
Stella Cousins
Diana Cousminer
Julien Coussement
Anna Coussens
Paul Coussens
Yohann Couté
Sheryl Coutermarsh-Ott
Diogo Coutinho
Felipe Coutinho
Julia Coutinho
Renato Coutinho
Marcos Coutinho Schechter
Iliano Coutinho-Abreu
Antoine Coutrot
Christopher Coutts
Jean-Philippe Coutu
Francis Couturaud
Berengere Couturier
Aude Couty
Alain Couvineau
Christine Covell
David Covell
Maureen Covelli
Adrian Covic
James Covington
Stephen Covington
Andrew Cowan
Don Cowan
Zara-Louise Cowan
Richard Cowden
Patricia Cowell
Stephen Cowen
Christina Cowger
Allen Cowley
Victoria Cowling
Seamus Cowman
Andrew Cox
Brian Cox
Brittney Cox
Daniel Cox
Eric Cox
Erin Cox
Helen Cox
James Cox
Laura Cox
M. Christina Cox
Marc Cox
Michael Cox
Miles Cox
Ruth Cox
Simon Cox
Timothy Cox
C. R. Coxon
Douglas Coyle
Seamus Coyle
Mark Coyne
David Coynel
Giorgio Cozza
Bruno Cozzi
David Crabb
Aurélie Crabbé

Margaux Crabtree
Brenda Crabtree-Ramırez
Timothy Crack
Daina Crafa
William Cragg
Jamie Craggs
Max Craglia
Ashley Craig
Bruce Craig
Maria Craig
Maureen Craig
Michael Craig
Paulig Craig
Timothy Craig
Tom Craig
Laila Craighero
Fergus Craik
Joseph Craine
Noel Craine
Ciprian Crainiceanu
James Crall
Erin Cram
Daniel W. Cramer
Holger Cramer
Joel Cramer
William Cramer
Peter Crampton
William Crampton
Aliceann Crandall
Sonia Crandall
Barbara Crane
Benjamin Crane
Jeff Crane
John Crane
Nicole Crane
Todd Crane
Tracy Crane
John Cranfield
Giuseppe Craparo
Fabien Crauste
Rolf Craven
Scott Craver
Joseph Cravero
Gale Craviso
Fiona Crawford
Forrest Crawford
Jeffrey Crawford
Lindsey Crawford
Nigel Crawford
Rebecca Crawford
Sue Crawford
Sybil Crawford
Heaven Crawley
Jacqueline Crawley
Carolyn Cray
Elise Crayton
Francesco Crea
Jenette Creaney
Seth Creasy
Melanie Cree-Green
Esther Creemers
Sarah Creem-Regehr
Bob Creese
Byron Creese
Melissa Cregger
Malte Cremer
Sylvia Cremer
Henk Cremers
Sylvain Cremoux
Thomas Crenshaw
Tiziana Crepaldi
Pascal Crépey
Pascale Crepieux
Elvira Crescenzi
Mark Crescenzi
Gail Cresci
Giada Crescioli
Emmanuele Crespan
Jose Crespo
Marcos Crespo
John Cressman
Sonya Cressman
James Cresswell
Jacob Creswell
Daniele Cretella
Benjamin Cretin
Silvia Cretoiu
Fabienne Crettaz von Roten

Armel Cretual
Felix Creutzig
Joseph Crew
David Crews
Kristine Crews
Leslie Crews
Menno P. Creyghton
Paulo Criado
Ernesto Criado-Hidalgo
Bronwen Cribb
Bart Criel
Cassandra Crifasi
Courtney Crim
Francis Crinella
Giuseppe Cringoli
Tawni Crippen
Michael Cripps
Richard Cripps
Alessandra Crisa
Antonio Crisafulli
Ernesto Crisafulli
Samuel Crish
William Crisler
Alastair Crisp
Martina Crispo
Matteo Cristani
Ioana Cristea
Matthieu Cristelli
Simona Cristescu
Alejandrina Cristia
Elena Cristiano
Riccardo Cristofani
Jean-Paul Cristol
Simone Cristoni
Christine Critchley
David Critchley
Julia Critchley
Elena Critselis
Faith Critzer
Leandro Crivellenti
Anna Croce
Davide Croce
Jenifer Croce
Roberta Croce
Emanuele Crocetti
John Crockarell Jr.
Jonny Crocker
Alessandro Crocoli
Leonard Crocombe
Tatiana Croenlein
Julie Croff
Cara Croft
Darin Croft
Darren Croft
Holly Croft
Kevin Croft
Ana-Maria Croicu
Keneth Croitoru
Rogier Crolla
Virginie Crollen
Howard Cromwell
Randy Cron
Candace Croney
Aidan Cronin
Owen Cronin
Tom Cronin
Alice Cronin-Golomb
Ruth Cronkite
Nigel Crook
Jim Crooks
Valorie Crooks
Wendell Cropper
David Cros
Marie-Josée Cros
David A. Crosby
Paolo Crosetto
Alan Cross
Paul Cross
Troy Cross
Robert Crossler
Merlin Crossley
Elena Crotti
Christophe Croux
Renato Crouzeilles
Christian Crouzet
Karen Crow
Scott Crow
Wade Crow

Larry Crowder
David Crowe
Sally Crowe
Trevor Crowell
Karin Crowhurst
Jennifer Crowley
Kevin Crowley
Cynthia Crowson
Michele Crozatier
Alyson Crozier
Sarah Crozier
Eric Crubézy
Aline Crucello
Tania Crucitti
Valeria Crudele
Mark Cruickshank
Steven Crum
Clyde Crumpacker
Rustin Crutchley
Anne-Marie Crutz-Le Coq
Maarten Cruyff
Adriano Cruz
Albert Cruz
Christian Cruz
Fernanda Cruz
Israel Cruz
Leonardo Cruz
Tania Cruz
Penélope Cruzado-Caballero
Vinicius Cruzat
Monica Cruz-Lemini
Jesús Cruz-López
Zobeida Cruz-Monserrate
Juan Cruz-Motta
Ariovaldo Cruz-Neto
Edwin Cruz-Rivera
Sally Cryan
Colin Cryer
Howard Crystal
Miklos Csala
Gabor Csanyi
Elmar Csaplovics
David Csercsik
Arpad Csernetics
Valér Csernus
Anita Cservenka
Gergő Csete
Gergely Csibra
Gabor Csifcsak
J. Csiszár
Laszlo Csonka
Sándor Csősz
Adrienne Csutak
Csaba Csuzdi
Lucas Cuadra
Francisco Javier Cubero
Francisco Cubillos
Susana Cubillos
Victor Cubillos
Esther Cubo
Jorge Cubo
T. Cubo
Esther Cubo Delgado
Belen Cubria
Alessandro Cucchetti
Brett Cucchiara
Francis Cucinotta
Luca Cucullo
Mihai Cucuringu
Patrick Cudahy
Gary Cuddeback
Brenda Cude
Gabriel Cuellar Partida
Luis Cuello
Nicolás Cuenca
Pablo Cuenca
Manuel Cuenca-Estrella
José Cuervo
Alberto Cuesta
Carlos Cueva
Raúl Cueva del Castillo
Daniel Cuevas
Andrew Cuff
Lucia Cugusi
Emmanuelle Cugy
Chang-Yi Cui
Chuyi Cui
F. Cui

Feng Cui
Guiyun Cui
Helen Wei Cui
Hongjuan Cui
Jie Cui
Jing Cui
Jin-Jie Cui
Jiujie Cui
Kehui Cui
Li Cui
Liang Cui
Liwang Cui
Mengchao Cui
Rutao Cui
Shangjin Cui
Taixing Cui
Wei Cui
Wenguo Cui
Xiangrong Cui
Xiang-Shun Cui
Xiaobing Cui
Xing Cui
Yuehua Cui
Yuhai Cui
Yunfu Cui
Yuqi Cui
Yuxin Cui
Zhaoxia Cui
Ziyou Cui
Frederic Cuisinier
Ivan Cuk
Adam Cuker
Engin Cukuroglu
Oana Culache
Susan Culican
Bernadette Cullen
Kevin Cullen
Paul Cullen
Alexander Culley
Fiona Culley
Christophe Cullin
Nyssa Cullin
Stephen Cullum
Susan Culos-Reed
Maxime Culot
Valeria Culotta
William Culp
David C. Culver
Bob Cumming
Douglas Cumming
Jennifer Cumming
Jonathan Cumming
Oliver Cumming
Anthony Cummings
Bethany Cummings
Brian Cummings
Linda Cummings
Sherry Cummings
C. Cummins
Carole Cummins
Carolyn Cummins
Eoin Cummins
Theodore Cummins
Ivan Cundrle Jr.
Thomas Cundy
Ana Cunha
Diana Cunha
Diogo Cunha
Joice M. Cunha
Leonardo Cunha
Sergio Cunha
Joana Cunha-Cruz
Marilia Cunha-Lignon
Barbara Cuniberti
Adam Cunningham
Charles Cunningham
Evan Cunningham
Kyle Cunningham
Lee Cunningham
Lisa Cunningham
Marc Cunningham
Melissa Cunningham
Scott Cunningham
Susan Cunningham
David Cunnington
Christina Cuomo
Ornella Cuomo
Gerald Cupchik

Rocío Cupeiro
Andrea Cupp
John Curci
Gary Curhan
Giulia Curia
Giuseppe Curigliano
Belinda Curley
Marcel Curlin
Maria Curotto de Lafaille
Bart Currie
Jane Currie
Rob Currie
David Currow
Joseph Curry
Marina Cursino
Ray Cursons
Ian Curthoys
Daniela Curti
Catherine Curtin
John Curtin
Richard Curtin
David S. Curtis
Jeffrey Curtis
Kathleen Curtis
Paul Curtis
Ryan Curtis
Thomas Curtis
Tim Curtis
Wayne Curtis
Roy Curtiss
Carina Curto
Vivian Cury
Fábio Cury de Barros
Davide Curzi
Melanie Cushion
Maria Grazia Cusi
Sarah Cusick
Rosa Cusido
Christine Cuskley
Cesare Cuspidi
Michel Cusson
Michelle Custead
Brian Custer
Estefania Custodio
Adele Cutignano
Chris Cutler
Teresa Cutright
Court Cutting
Simon Cutting
Carrie Cuttler
Olivier Cuvier
Robert Cuzner
Salvatore Cuzzocrea
Maria Cvach
Ales Cvekl
Uros Cvelbar
Marina Cvetkovska
Barbara Cvikl
Mariusz Cycoń
Melissa Cyders
Boguslaw Cyganek
Praveen Cyriac
Dirk Cysarz
Tomer Czaczkes
K. Czaja
Laszlo Czako
Michael Czaplik
Michael Czech
Peter Czermak
Joseph Czerniecki
Istvan Czigler
Michael Czihal
Mirjam Czjzek
Michał Czopowicz
Henryk Czosnek
Marek Czosnyka
Paul Czoty
Michael Czubryt
Peter Czuppon
Anna Czupryna
Heloisa D´Ávila
Angelo D’Alessandro
Riccardo D’Ambrosi
Francesca D’Errico
T. D’Orazio
Manoranjan D’Souza
Chaojiu Da
Feipeng Da

Wendi Da
Xing Da
Bruno da Costa
Milton da Costa
Thais da Costa
Rute da Fonseca
Bernardo da Gama
Daniele Da Lio
Felipe Da Luz
Raquel I. da Luz
Rubia da Rocha Valente
A. S. Da Silva
Aline da Silva
André da Silva
Anne Da Silva
Diane Da Silva
Edson da Silva
Eufranio da Silva
Gabriela Jorge da Silva
Hugo Plácido da Silva
José Maria Cardoso da Silva
Joyce Kelly da Silva
Julimar Da Silva
Marcelo da Silva
Marcelo José da Silva
Natal da Silva
Rafaela da Silva
Tânia Mara Da Silva
Tatiane Da Silva
Geraldo da Silva Junior
Itabajara Da Silva Vaz Jr.
Juliano da Silveira
Ronis Da Silveira
GV da Veiga
Shareef Dabdoub
Sandrine Dabernat
Dana Dabiri
Jesse Dabney
Wojciech Dabrowski
Ralph Dacey
Jordi Dachs
James Dachtler
Joel Dacks
Guy Daculsi
Ekaterina Dadachova
Trifone D'Addabbo
Stijn Daenekindt
Katherine Dafforn
Amrita Daftary
Noa Dagan
Stephanie d'Agata
Tisja Daggers
Matteo Daghio
Mohsen Daghooghi
John Dagle
Gislin Dagnelie
Lina Dagnino
Fortune Dagnon
Gregorio D'Agostino
Albert Dahan
Michael Dahan
Niroshana Dahanayake
Yael Dahan-Moss
Harel Dahari
Elizabeth Daher
Navdeep Dahiya
Satinder Dahiya
Sunita Dahiya
Audun Dahl
Christiane Dahl
Björn Dahlbäck
Gunnar Dahlen
Joseph Dahlen
Craig Dahlgren
Maik Dahlhoff
Lars Dahlin
James Dahlman
Kimberly Dahlman
Annegret Dahlmann-Noor
Kam Dahlquist
Geraldine Dahlqvist
Kristina R. Dahlstrom
Maznah Dahlui
Darren Dahly
Souhayl Dahmani
Hans-Uwe Dahms
Bachar Dahro
Boyi Dai

Chang-Feng Dai
Chia-Yen Dai
Chuanchao Dai
Chunfu Dai
Dajun Dai
Jianfeng Dai
Jihong Dai
Junhu Dai
Lisheng Dai
Qiang Dai
Wei Dai
Xiaoxia Dai
Xi-Jian Dai
Xinbin Dai
Xingui Dai
Yang Dai
Yi Dai
Yong Dai
Zen-Kong Dai
Zhengjia Dai
Zhi-Cong Dai
Zhifeng Dai
Zhiyu Dai
Zhuang Dai
Andreas Daiber
Adam Daigneault
Frank Dailey
Harry Dailey
Michael Daily
Masao Daimon
Stephen Dain
Antoine Daina
Benjamin Dainat
Cori Daines
Singla Daisy
Fidelia Dake
Sadoun Fahad Dakhil
Helen Dakin
Roslyn Dakin
Fabien Dal Maso
Juliano Dal Pupo
Swayamprava Dalai
Sushila Dalal
Jelle Dalenberg
Emmanuelle d'Alencon
Julio Daleprane
Fredrik Dalerum
Yuri D'Alessandra
Giuseppe Dalessandro
Evan D'Alessandro
Allison Daley
Brian Daley
Louise Dalgaard
Ertugrul Dalgic
Ankur Dalia
Philip Ayizem Dalinjong
Mehmet Dalkilic
Teresa Dalla Costa
Dinara Dallaeva
Cecilia Dall'Aglio
Houman Dallali
Robert Dallapiazza
Enrico Dall'Ara
Viviana Dallard
George Dallas
Tad Dallas
Chiara Dall'Asta
Antonella Dalle Zotte
Olivia Dalleur
Geesje Dallinga-Thie
Reinhard Dallinger
Mario Dalmaso
Guillaume Dalmasso
Claudia Dalmastri
Christine Dalmazzone
Jean-Charles Dalphin
Jade d'Alpoim Guedes
Kirsten Dalrymple
Søren Dalsgaard
Véronique Dalstein
Danyel Dalto
Annamarie Dalton
Desiré Dalton
Patricio Dalton
Anne Daltveit
Mohammad Dalvi-Esfahani
Christopher Daly
Daniel Daly

Kimberly Daly
Lisa Daly
Martin Daly
Mary Daly
Marymegan Daly
Kim Dalziel
Maurizio D'Amario
Oriana Damas
Benito Damasceno
Jeziel Damasceno
Nadyr Damasceno
M. Damasiewicz
Gennaro D'Amato
Angelo D'Ambrosio
Pauliina Damdimopoulou
Stefano D'Amelio
Catherine Damerval
Christian Damgaard
Francesco Damiani
Rachel Damico
Reid D'Amico
David Damiens
Priyadarshan Damle
Philip Dammann
Jesper Dammeyer
Chendil Damodaran
Gaëlle Damour
Fredrick Damron
Timothy Damron
Christian Damsgaard
Reza Dana
Teodor Danaila
Ali Danandeh Mehr
Antoine Danchin
Abhaya Dandekar
Chethan Dandin
Paul Dando
Samantha Dando
Rakhi Dandona
Ivana d'andrea
Marco Maria D'Andrea
Mariasilvia D'Andrea
Fabio D'Andreagiovanni
Antonio D'Andrilli
Andrew Dane
Marcelle Danelon
Nick Daneman
Elisa Danese
Silvio Danese
P. Danesi
Cathy Danesin
Hong Dang
Linh Dang
Shaonong Dang
Cuong T. Dang
Tran Ngoc Dang
Tuan Dang
Xin Dang
Nabin Dangal
Daniela D'Angelo
Livia D'Angelo
Sadikshya Dangi
Ziyaad Dangor
Volodia Dangouloff-Ros
Thanh Dang-Vu
Carlo Dani
Sven Dänicke
Ebenezer Daniel
Hans-Dieter Daniel
Jean-Baptiste Daniel
Ramiro Daniel
Richard Daniel
Van Santen Daniëla K.
Antonio Daniele
Soares Danielle
Salvatore D'Aniello
David Danielpour
Jaret Daniels
Jo Daniels
Joseph Daniels
Karen Daniels
Megan Daniels
Rachel Daniels
Ranjit Daniels
Savel Daniels
Robert Danka
Scott Dankel
Heidi Danker-Hopfe

Petra Dannapfel
Camille Danne
Michael Dannemann
Astrid Dannenberg
Ng Danny Wang-Kit
Sonye Danoff
Olivier Danot
Ina Danquah
Jan Danser
Emily Dansereau
Jayne Danska
Jaya Dantam
Ana García Dantas
Paulo Cesar Dantas Silva
Robert Dante
Matthew Danter
Subrahamanyam Dantham
Matteo D'Antonio
Nico Dantuma
Antonietta D'Antuono
László Dányi
Heather Danysh
Karin Danzer
Michael Danziger
Zhang Daopei
Nihaya Daoud
Dimitrios Daoussis
Fredrick Dapaah-Siakwan
Elena Daprati
Richard D'Aquila
Nawab Dar
Osman Dar
Radbod Darabi
Faryad Darabi Sahneh
Cinzia Daraio
Konstantinos Daras
Benjamin Darbro
Ivan Darby
Philip Darby
Nicolas Darcel
Céline Dard
Anuja Darekar
Mohammad Daremipouran
Raellene Dare-Smith
Peter Dargaville
Emmanuelle d'Argent
Marco D'Arienzo
Pawlak Dariusz
Michael Dark
Victor Darley-Usmar
Douglas Darling
Eric Darling
Katharine Darling
Cynthia Darling-Fisher
Anne-Sophie Darlington
Pierre Darlu
Dominique Darmaun
Michael Darmon
Simon Darnell
Wesley Darocha
Karin Darpel
Margot Darragh
Sébastien Darras
Michelle Darrieux
Eric Darrouzet
Benjamin Darter
Thomas Darton
Richard Darveau
Brian Darvell
Hosain Darvishi
Hala Darwish
Zbigniew Darzynkiewicz
Amit Das
Anindita Das
Anindya Bijoy Das
Anupam Das
Bhaskar Das
Bhudev Das
Chittaranjan Das
Dibash Das
Dipak Das
Gitanjali Das
Kali Das
Krishna Das
Madhurima Das
Mahasweta Das
Malay Das
Nabanita Das

Raibatak Das
Reena Das
Saikat Das
Sampa Das
Sanchita Das
Sindhupriya Das
Subir Das
Suman Das
Sunit Das
Undurti Das
Krishna Das Saha
Gautam Dasarathy
Srinivasan Dasarathy
Kiran Dasari
Mihai Dascalu
Fabrizio D'Ascenzo
Ben Dascombe
Partha Dasgupta
Piyali Dasgupta
Ramanuj Dasgupta
Subham Dasgupta
Susmita Dasgupta
Hirak Dash
Paban Dash
Padmanava Dash
Pradyot Dash
Srikanta Dash
Behdad Dashtbozorg
Hassan Dashti
Luis Dasilva
Regilane daSilva
Stella Daskalopoulou
Nina Daskalovska
Asok Dasmahapatra
Jayati Das-Munshi
Ruth Dassonneville
Syed Dastagir
Mehran Dastmalchi
Ravi Dasu
Daniel Datiko
Manuel Datiles
Alexandre N. Datta
Animesh Datta
Kasturi Datta
Malabika Datta
Pratik Datta
Subhojit Datta
Mehul Dattani
Michael Dattilo
Raymond Dattwyler
Diane Daubert
Ivana Daubnerova
Luc Dauchet
Aizati Daud
Nor Shidrah Mat Daud
Larry Dauer
Margery Daughtrey
John Daugirdas
Martine Daujat-Chavanieu
Robert Daum
Bruno D'Aurea Furquim
Giuseppe D'Auria
Marco Dauriz
Bertrand Dautzenberg
Brigitte Dauwalder
Rafael Davalos
Veronica Davalos
Kinjal Dave
Shruti Dave
Vachik Dave
Emily Davenport
Miles Davenport
Ross Davenport
Christopher Davey
Gavin Davey
Mary Ann Davey
Mary Davey
Della David
Dryden David
Jean-Pierre David
Mariana David
Marilia David
Meckes David
Michael David
Richard David
Stephen David
Tim David
Tony David

Valentin David
Sandra Davidge
Brett Davidheiser-Kroll
Nickolai Davidovich
Eugene Davids
Malika Davids
Alec Davidson
Colin Davidson
Giana H. Davidson
Jennifer Davidson
Lance Davidson
Silas Davidson
Alun Davies
Andrew Davies
Brandon Davies
Bryan Davies
Christopher Davies
Claire Davies
Helen Davies
Kay Davies
Kelvin Davies
Louise Davies
Michael Davies
Nathan Davies
Noel Davies
Shireen Davies
Stella Davies
Stephen Davies
Stuart Davies
Tim Davies
Rob Davies-Colley
Miranda Davies-Tuck
Anne-Marie Davila
Marco Davila
Marivel Davila
Monica Davila
Claudia d'Avila-Levy
Sergio Davinelli
Aaron Davis
Adam Davis
Alan Davis
Alexandra N. Davis
Andrew Davis
Anthony Davis
Brian Davis
Cristina Davis
Daniel Davis
Edward Davis
Greg Davis
Gregg Davis
James Davis
James J. Davis
Jason Davis
John Davis
Justin Davis
Kathleen Davis
Kelliann Davis
Mark Davis
Matthew Davis
Meghan Davis
Melinda Davis
Melissa Davis
Meryl Davis
Michael Davis
Paul Davis
Philip Davis
Phillip Davis
R. Eric Davis
Randall Davis
Richard Davis
Roger Davis
Thomas Davis
Tyler Davis
William Davis
Jon Davison
Vincent Davisson
Christopher Davitt
Bazbek Davletov
Horvatic Davor
Jean Davoust
Hasan Davulcu
Anurag Daware
Adriana Dawes
Colin Dawes
Diana Dawes
Piers Dawes
Igor Dawid
Buddhadeb Dawn

Lowe Dawn
Adekunle Dawodu
Mahmoud Dawood
Dalia Dawoud
Rajinder Dawra
Alison Dawson
Dolph Dawson
Jeremy Dawson
Julie Dawson
Lisa Dawson
Liza Dawson
Michael Dawson
Brad Day
Craig Day
Jo Day
Regina Day
Sanjana Dayal
Franck Dayan
Victor Dayan
D. A. Dayde
Maher Dayeh
Hu Dayong
Carolyn Dayton
Paul Dayton
Alvar Daza
Ghassan Dbaibo
Jonathan D'Cunha
Debashree De
Prithwiraj De
Josiane de Almeida
Tiago de Almeida
Guillermo De Anda-Jaúregui
Daniel de Andrade
Paola De Angelis
Gabriel de Araujo
Juliana de Araújo
Lucilla de Arcangelis
Vania Regina de Assis
Ole De Backer
Marcos de Bastos
J. de Beer
Maria Elena de Bellard
Anna De Benedetto
Giulia Maria De Benedictis
Dott De Berardis
Edwin de Beurs
Esther de Beus
Dario de Biase
Filomena De Biasio
Claire De Bisschop
Jessica de Bloom
Geertruida de Bock
Anneloes de Boer
Bart de Boer
Hugo de Boer
Jetske De Boer
Pieter de Boer
Sietse de Boer
Xavier De Bolle
Raquel De Boni
Mario de Bono
Nicola de Bortoli
Karolien De Bosscher
Remco de Bree
Anna de Breij
Savitha De Britto
Marc de Broe
Irene de Bruijn
Angela De Bruin
Eling D. de Bruin
Marijn de Bruin
Patrícia de Brum Vieira
Frederik De Buck
Eliana de Camargo
Gregorio de Camargo
Michel Leandro de Campos
Paola de Candia
Elisabetta De Cao
Juliana Ribeiro de Carvalho
Leandro de Carvalho
Mamede de Carvalho
Marco Antonio de Carvalho-Filho
Gabriela de Castro
Loris De Cecco
Benito de Celis Alonso
Annarosaria De Chiara
Francesco De Chiara
Eva De Clercq

Monaca De Cola
Valentina De Cosmi
Bruna de Costa Alberton
Barbora de Courten
Valerie de Crécy-Lagard
Dileepa de Croos
Marco de Curtis
Simon De Deyne
Carsten De Dreu
Tony De Falco
Roseane De Fátima Guimarães
Italia De Feis
Francesca De Felice
Francesca De Filippis
Stefan de Folter
Davide De Francesco
Ernestina De Francesco
Vincenzo de Francesco
Pilar de Frutos
Vidya de Gannes
Vincent de Gardelle
Bart De Geest
Beatrice de Gelder
Luigi De Gennaro
Brechje De Gier
Ugo De Giorgi
Marco De Giovanni
Maria R. C. de Godoy
Dirk de Graaf
Miranda De Graaf
Pablo de Gracia
Armando De Gracia Nieto
Cesare de Gregorio
Eliana De Gregorio
Bert De Groef
Arjan de Groot
Imelda de Groot
Friedl De Groote
David De Haan
Adam de Havenon
Jan De Houwer
Cornelis de Jager
Saskia de Jager
Magdia De Jesus
Vinicio de Jesus Perez
Cornelis De Jong
Hans de Jong
Peter de Jong
Pim de Jong
Hugo de Jonge
Jørgen de Jonge
Ronnie de Jonge
Jos F. M. de Jonghe
Cyrille de Joussineau
Silvia de Juan
Rob de Keizer
Annette de Kloet
Henrik J. de Knegt
Peter De Knijff
Harry de Koning
Lawrence de Koning
Mariken de Koning
Laetitia de Kort
Ronald de Krijger
Jorge de la Calleja
Albert de la Chapelle
Irene De la Cruz Pavía
Christelle de la Fouchardiere
Mónica De la Fuente
Fernando de la Gándara
Horacio de la Iglesia
Rodrigo de la Iglesia
Maria de la Iglesia Vaya
Aurelien de la Lande
Manuel De La Mata
Nicole De La Mata
Luis de la Maza
Suzanne de la Monte
Carol De La Motte
Ivan de la Providencia
Patricia De la Riva
Laura de la Rosa
Pilar De la Rua
Claire de La Serre
Jimmy de la Torre
Laura de la Torre
Isabel de la Torre-Diez
Jan de Laffolie

D. W. (Dylan) De Lange
Thomas de Lange
Lisa de las Fuentes
Vincenzo De Laurenzi
Paul De Lay
Laura De Lellis
Letícia Celia de Lencastre Novaes
Giulio De Leo
Francesco De Leonardis
Nico De Leu
Laurence de Leval
Kalil De Lima
Jan Willem de Lind van Wijngaarden
Stephen De Lisle
Esther De Loof
Mónica De los Reyes
Maxy De los Santos
Teresa De Los Santos
Emilia De Luca
Maia De Luca
Maria De Luca
Paul De Luca
Manuela de Lucas
Malson de Lucena
Ruud de Maagd
João Pedro de Magalhães
Paolo De Marco
Yang De Marinis
Luigi De Martino
Luisa De Martino
Maurizio de Martino
Gianluca De Masi
Quirijn de Mast
Maria Antonietta De Matteis
Sara De Matteis
Ingrid De Meester
Tim de Meij
Marco de Mello
Paulo de Mello Affonso
Fernanda de Mello Malta
Marc De Meyer
Simon De Meyer
Pierre De Meyts
Giuseppe De Michele
Carlos de Miguel
Joachim de Miranda
Leonardo de Miranda
Mariane de Montalembert
Valentina De Monte
Leandro de Morais Cardoso
Miguel de Mulder
Kim de Mutsert
Gert-Jan De Muynck
Pasquale De Negri
Robin de Nijs
Mariana De Niz Hidalgo
Carlos de Noronha
Angela de Oliveira
Camila de Oliveira
Cassia de Oliveira
Elaine de Oliveira
Guilherme de Oliveira
Leandro de Oliveira
Leonardo Pestillo de Oliveira
Lucia de Oliveira
Natasha de Oliveira
Rodrigo de Oliveira
José de Oliveira Filho
Paola de Pablo
Alessandra De Pádua Bueno
Simone de Padua Teixeira
Nick de Palma
Emanuele De Paoli
Donatella de Pascale
Roberto De Pascalis
Eneida de Paula
Francisco de Paula
Heitor de Paula Neto
Vanessa De Pietri
Gleiciani de Queiros Silveira
Nick De Regge
K. De Reu
Hilde de Reuse
Anik de Ribaupierre
Denise De Ridder
Raffaele De Risi
Giuseppe De Rosa
Giusseppe De Rosa

Maria Cristina De Rosa
Michael De Rosa
Nicoletta De Rosa
Alessandro de Rosis
Nicolas de Roux
Philiphi de Rozari
Godard C. W. de Ruiter
Renata De Sá
Luc de Saint Martin
Tainá de Sandes-Freitas
Giovambattista De Sarro
Caroline De Schacht
Maarten De Schryver
Angela de Silva
Nisansa de Silva
Giovanni de Simone
Charles De Smet
Lina De Smet
Patrícia de Soárez
Paul De Sousa
Maria Elizabeth de sousa Rodrigues
André de Souza
Arnaldo de Souza
Heitor de Souza
Robson de Souza
Matheus de Souza Barbosa
Luís Carlos de Souza Ferreira
Anthony De Soyza
Marco De Spirito
Alfonso De Stefano
Hans De Steur
Bart De Strooper
Benoit de Thoisy
Mario De Tullio
Sarah De Val
Carolina de Vargas Nunes Coll
Jaira de Vasconcellos
Anke De Veer
Manuel de Vega
Miguel de Vega
Fabrizio De Vico Fallani
Dominique de Vienne
Jill De Vis
Richard de Visser
Teresa De Vita
Andrea De Vito
Albert De Vries
Ap de Vries
Bradley de Vries
Henry de Vries
Jan de Vries
Jeanne de Vries
Marieke de Vries
Paul de Vries
Sjoerd de Vries
Taco De Vries
Vivian de Waard
Damien De Walque
Nicole De Weerd
Stephan De Wekker
Ksander de Winkel
Heleen de Wit
Lee de Wit
Lenore de Wit
Massimiliano de Zambotti
Roeland J. G. De Moor
Martin de Santibañes
Chatsri Deachapunya
Amy Deacon
Andras Deak
Chris Dealwis
Caroline Dealy
Dennis Dean
Harlan K. Dean
Matthew Dean
Michael Dean
Nathan Dean
Nathan P. Dean
Philip Dean
David Deane
Rashid Deane
Don DeAngelis
Jennifer Dearborn-Tomazos
Kirk Dearden
Clare Death
Philippe Debaeke
Pati Debananda
Florence Debarre

Vincent Debat
Bryann DeBeer
Lucie Debeffe
Rudolf Debelak
Željko Debeljak
Matt DeBerge
Alberto Debernardi
Stephanie Debette
Mélanie Debiais-Thibaud
He Debiao
Hanna Debiec
Thierry Debillon
Waldemar Debinski
Marijke DeBock
Jane Debode
Marta Debolini
Miguel Debono
Hilda Deborah
Nathan Debortoli
Agata Debowska
Didier Debroas
Jennifer Debruyn
Igor Debski
Charlotte Debus
Zeger Debyser
Nicola Decaro
Ana deCarvalho
Simon Décary
François-Xavier Dechaume-Moncharmont
Alexander Dechene
Alan W. Decho
Paul Dechow
Volodymyr Dechyporuk-Zloy
Jared Decker
Scott Decker
Hans Deckmyn
Mathieu Declerck
Guillaume Decocq
Caroline Decosta
Heather Decot
Wilfrida Decraemer
Lieselot Decroix
Etienne Decroly
Tom Decroo
Adeline Decuyper
Simon DeDeo
Dan Dediu
Keith Dedonder
Fabrice Dédouit
Nicolas Dedy
Michael Dee
Scott Dee
Patrick Deegan
Shelley Deeks
Dinesh Deelchand
Suha Deen
Shashank Deep
Amanda Deering
Leslie DeFalco
Luc Defebvre
G. Defeudis
Xavier Deffieux
Guillaume Deffuant
Paola Defilippi
Dieter Deforce
Mariam Degani
Abraham Degarege
Marc Degelaen
Allan Degen
Bernd Degen
Howard Degenholtz
Hans Degens
Cyril Degletagne
Patrick Degnan
Francesca Degola
Bertrand Degos
Donald DeGracia
Timothy DeGrado
David DeGraff
James DeGregori
Juno Deguchi
Shinji Deguchi
Joe DeGutis
Christian DeGuzman
L. R. DeHaan
Wim Dehaen
Kong Dehan

Mark DeHaven
Benjamin Dehay
Mohammed Dehbi
Tahereh Dehdarirad
Mahshid Dehghan
Maziar Dehghan
Cirous Dehghani
Morteza Dehghani
Roozbeh Dehghannasiri
Christine Dehlendorf
Carola Dehler
Jamshid Dehmeshki
Nathalie Dehne
Ahmad Dehpour
Ranjit Dehury
Omid Dehzangi
Scott Deibel
Marie Deiber
Andrew M. Deines
Chad Deisenroth
Nina Deisig
Helmut Deissler
Kirk Deitsch
Jörg Deiwick
Sintayehu Dejene
Benjamin Dekel
Nava Dekel
Aldo Dekker
Andre Dekker
Jasja Dekker
Jack Dekkers
Koen Dekkers
Brandon Dekovsky
Rosa del Campo
Ignacio del Castillo
Rafael del Castillo
Kleber Del Claro
Maite Del Collado
Juan Del Coso
Silvia Del Din
Marco Del Giudice
Liana Del Gobbo
Stephen Del Grosso
Jesus del Mazo
Annarosa Del Mistro
Federica del Monte
Luis del Papa
Luis del Peso
Marta Del Pino
Miguel A. del Pozo
Olga del Pozo
Jesús Del Pozo Cruz
Andrew Del Pozzi
Dominic Del Re
Alberto Del Rio
Silvia Del Ry
Lorenzo Del Sorbo
Marc Esteve del Valle
Michela Del Vicario
Delphine Delacour
Vincent Delafont
Patrice Delafontaine
Robin Delahay
Eamonn Delahunt
Paul Delamater
Pierre Delanaye
Aogán Delaney
John Delaney
Kevin Delaney
Meghan Delaney
Peter Delaney
Thomas DeLaney
Joris Delanghe
Sabine Delannoy
Paul Delano
Alexandre Delanoe
John P. Délano-Frier
Pierre Delaplace
Cecile Delarasse
Edson Delatorre
Eo Delatorre
Ronald Delaune
Philippe Delavault
Eugene Delay
Tobi Delbruck
Sebastien Delcasso
Kleber Del-Claro
Candice Delcourt

Louise Deldicque
Henri-Jacques Delecluse
Krisine DeLeon-Pennell
Philippe Delespaul
Emilie Deletre
Jade Delevaux
Alessandro Delfanti
Carolina Delgado
Mario Delgado
Rafael Delgado
Roberto Delgado-Bolton
Jesus Delgado-Calle
José Delgado-García
David Delgado-Hernández
Santiago Delgado-Tirado
Benoit Delhaye
Jan Delhey
Kaspar Delhey
Alexander Delides
Mario Milco D'Elios
Matthew DeLisi
Brian Delisle
Helene Delisle
Jonathan Delisle
Sylvain DeLisle
Alessandro Delitala
Devy Deliyanti
Silvia Della Bella
Chiara Della Libera
Maria Elena Della Pepa
Giorgia della Rocca
Sara Della Torre
Graham Dellaire
Dean DellaPenna
Emanuela Dell'Aquila
Dominick DellaSala
Robert Dellavalle
Sergio Dellepiane
Christian Delles
Antonio Dello Iacono
Evan Dellon
Aurelien Delluc
Volker Dellwo
Mario Delmar
Allison DeLong
Jordan DeLong
Ralph DeLong
Sabrina Delong
Alejandro Delorenzi
François Delori
Stefan Delorme
Vincent Delorme
Tristan Delory
L. DeLott
Regine Delourme
Cédric Delporte
Flavia Delporto
Ruth Del-Prado
Marcos Delprato
Constantinos Deltas
Angelo DeLucia
Christophe Delye
Ayako Demachi-Okamura
George Demakis
Sandrine Demaneche
Peter Demant
Francesca Demarchi
Gianpaolo Demarchi
Cristiano Demarchis
Ricardo DeMarco
Vincent DeMarco
Wendy Demark-Wahnefried
Emilie Demarsy
Eleonora Demartin
Vlad Demartsev
Marilene Demasi
Ronald DeMatteo
Nicolas Demaurex
Deeptankar DeMazumder
Simona Demel
Sébastien Dementin
Marika Demers
Michael DeMers
Athena Demertzi
Stefanos Demertzis
Panagiotis Demestichas
Lloyd Demetrius
Zsolt Demetrovics

Christian Demeure
Simon DeMeyer
Nele Demeyere
Libertario Demi
Eugene Demidenko
Ilya Demidyuk
Marie Demion
Claire Demiot
Aydeni Demir
Ihsan Ekin Demir
F. Yesim Demirci
Nazli Demirel
Nikos Demiris
Mukaddes Sakalli Demirok
Murat Demirtas
Kürşat Demiryürek
Serkalem Demissie
Ryan Demmer
Fabio Demontis
Sharon DeMorrow
Evangelia Demou
Nathalie Demoulin
Bruce Demple
Paddy Dempsey
Satoru Demura
Henk den Bakker
A. M. den Harder
Daan den Hollander
Dirk den Ouden
Jaap Den Toonder
Avram Denburg
Kara Denby
Christy Denckla
Vincent Denef
Adam Denes
Caroline Denesvre
Fei Deng
Fenglin Deng
Glenn Deng
Han-Bing Deng
Hong Deng
Hongbing Deng
Huiping Deng
Jianming Deng
Jie Deng
Jun Deng
Linhong Deng
Qi Deng
Qiang Deng
Sophie Deng
Tao Deng
Tianhu Deng
Wen-Tao Deng
Win-Ping Deng
Xiaojuan Deng
Xin Deng
Xutao Deng
Ye Deng
Yongqiang Deng
Zhenhua Deng
Zhiying Deng
Julius Dengler
Tim Denham
Emiel A. DenHartog
Justin Denholm
Srdjan Denic
Amedeo DeNicolo
Cecile Denis
Dan Denis
Gerald Denis
Jean-Louis Denis
Max Denis
Vianney Denis
Baudouin Denis de Senneville
Pierre Denise
Rachel Denison
Evgeny Denisov
Michelle Dennehy
Robin Dennell
Jack Dennerlein
David Denning
James Dennis
Jonathan Dennis
Louise Dennis
Peter Dennis
Rodolfo Dennis
Todd Dennis
Mark Dennison

Mark Denny
Lise Denoeud-Ndam
Eileen Denovan-Wright
Béatrice Denoyes
Tobias Denskus
James A. Densley
Nancy Denslow
Llewellyn Densmore
Jessica Dent
Micheal Dent
Christopher Denton
Marianne Denton
Robert Denver
Martin Denvir
Annette Denzinger
Makarand Deo
Sapna Deo
Atul Deodhar
Anil Deolalikar
C. Deom
Sarah Depallens
Ralph DePalma
Claire Depardieu
Antoine Depaulis
Nicolas Depauw
Marcel Deponte
Colin Depp
Stephen Deppen
Scotty DePriest
Bertrand Deputte
Clio Der Sarkissian
Anne Derache
Hooman Derakhshani
Charles Derby
Nina Derby
Fabio Dercole
Maria Chiara Deregibus
Sebastien Deregnaucourt
Enrico Derenzini
Christopher DeRenzo
Tekalign Deressa
Laurent Derex
Mykola Dergai
Amare Deribew
Maxime Derisbourg
Rachel Derita
P. Derksen
Valerian Derlega
Veronique Dermauw
Ryan Dermot
Birgit Derntl
Andrew Derocher
Laurie Derose
Robert DeRose
Mindy DeRouen
Philippe Derreumaux
Steven Derrick
Ainslie Derrick-Roberts
Nikolaos Dervisis
Marc-Andre Dery
Rik Derynck
Elena Deryugina
Tatyana Deryugina
Don Des Jarlais
Lauren Des Marteaux
Ankit Desai
Madhavi Desai
Moreshwar Desai
Tejas Desai
Ruel Z. B. Desamero
Jean-Francois Desaphy
Jane Desborough
Diane Descamps
Sébastien Descamps
Alexis Descatha
Thibault Deschamps
James Deschner
Tobias Deschner
Yves Desdevises
Kobe Desender
Jyoti S. Deshmukh
Rupesh Deshmukh
Sachin Kumar Deshmukh
Gaurang Deshpande
Maithili Deshpande
Nandini Deshpande
Raamesh Deshpande
Smita Deshpande

Vikram Deshpande
Giovambattista Desideri
Vincenzo Desiderio
Udaya DeSilva
Taseen Desin
Suzanne Desire
Camille Desjonquères
Berthony Deslouches
Tom Desmet
Michel Desmurget
Lucía DeSoto
Guillaume Desoubeaux
Sanda Despa
Pierre-Yves Desprez
Mahalia Desruisseaux
Tigestu Desse
Ulrich Desselberger
Yadeta Dessie
Francesco Dessì-Fulgheri
Shimelis Dessu
Jeffrey Destefano
Roger Detels
Megan Detloff
Ilario DeToma
Pieter deTombe
Allan Detsky
Agnes Dettai
Jon Detterich
Laura Detti
Ulf Dettmer
Gabriella d'Ettorre
Svenja Deuchler
Jordi Deu-Pons
Cornelius Deuschl
Jan Deussing
Arielle Deutsch
Eric Deutsch
Josef Deutscher
Murray Deutscher
Heinz Deutschmann
Krishnakumar Devadas
Sakamon Devahastin
Guillaume Devailly
K. Devaki
Jerome Deval
Nicholas Devaney
Trevor DeVaney
Niranjali Devaraj Sivasitambaram
Rachayya Devarumath
Niveditha Devasenapathy
Carole Devaux
Pedro Develey
Christian Devenish
Francisco Devescovi
Leticia Deveza
Pradip Devhare
Nithya Devi
Rukumani Devi
Sundru Devi
Raul Devia Rodriguez
Vincent Devictor
Chiara Devirgiliis
Elise DeVito
Pratap Devkota
Angela Devlin
Maureen Devlin
Nele Devoogdt
Marcella Devoto
Trevor DeVries
Wiebe deVries
Kevin Dew
Rebecca Dew
Carolyn Dewa
Michel DeWaard
Howard Dewald
Adam Dewan
Amielle DeWan
Ashraf Dewan
Alex Dewar
Michaela Dewar
Megan Dewdney
Grant Dewell
Youssef Dewer
Alexander Dewerth
Kathryn Dewey
Michael Dewey
Fred DeWinter
Jamie DeWitt

Sharon DeWitte
Walter Dewitte
Arthur Dewolf
Grant Dewson
Jeroen Dewulf
Franklin Dexter
Abhishek Dey
Aditi Dey
Ayan Dey
Madhusudan Dey
Nilanjan Dey
Sandeepa Dey
Sohini Dey
Satoshi Deyama
Farzad Deyheim
Randy DeYoung
Cameron Dezfulian
Nicole Deziel
Linda Dezsö
Sangappa M. Dhaded
Ajay Dhaka
Santosh Dhakal
Harcharan Dhaliwal
Mukesh Dhamala
Navdeep Dhami
Sonam Dhamija
Klodian Dhana
Danny Dhanasekaran
Dharumadurai Dhanasekaran
Renumathy Dhanasekaran
Saravana Mohan Dhanasekaran
Perundurai Dhandapany
Subramanian Dhandayuthapani
Nirav Dhanesha
Gunanidhi Dhangadamajhi
Amreeta Dhanoa
Animesh Dhar
Deepanshi Dhar
Dolly Wattal Attal Dhar
Neeraj Dhar
Ratan Dhar
Suman Dhar
Shalmali Dharmadhikari
Guha Dharmarajan
Madushini Dharmasena
Sunethra Dharmasiri
Rajat Dharr
Devesh Dhasmana
Mohan Dhawan
Punita Dhawan
Nolwenn Dheilly
Gurjeet Dhesi
Ashwat Dhillon
Haryana Dhillon
Preet Dhillon
Meghnath Dhimal
Sunil Dhiman
Sanjiv Dhingra
Rolina Dhital
Jan D'hooge
Sahajal Dhooria
Raja Ram Dhungana
Mengyang Di
Zhao Di
Mauro Di Bari
Belinda Di Bartolo
Vincenzo Di Bartolo
Gianluca Di Bella
Claudia Di Bene
Antonio Di Biagio
Lazzaro di Biase
Maria Grazia Di Bono
Alessandra di Cagno
Barbara Di Camillo
Dino Di Carlo
Giuseppe Di Caro
Enrico Di Cera
Lorenzo Di Cesare Mannelli
Maikon Di Domenico
Jeremy Di Domizio
Francesco Di Dona
Paola Di Donato
Mirko Di Febbraro
C. Di Francesco
Fabrizio Di Francesco
Antonio Di Franco
Mauro Di Giancamillo
Pierpaolo Di Giminiani

Sibilla Di Guida
Anne-Marie Di Guilmi
Mauro Di Ianni
Concetta di Iorio
Di Di Jin
Silvia Di Legge
Giuseppe Di Leo
Vito Di Lernia
Gabriele Di Lorenzo
Nicolas Di Lorenzo
Rosaria Di Lorenzo
Marco Di Luca
Eric di Luccio
Giulia Di Lullo
Luca Di Lullo
Massimo Di Maio
Fabiano Di Marco
Sergio Di Marco
Giovanni Di Matteo
Michele Di Mauro
Giulio Di Minin
Matteo Nicola Dario Di Minno
Fabrizio Di Muro
Antonello Di Nardo
Antonello Di Paolo
Cinzia Di Pietro
Giovanni Di Pino
Antonella Di Pizio
Gennaro Di Prisco
Elisa Di Rosa
Mirko Di Rosa
Silvia Di Santi
Dario Di Silvestre
Attilio Di Spiezio Sardo
Marco Di Stefano
Marilena Di Valentin
Francesco Di Virgilio
Valerio Di Vittori
Erika Di Zazzo
Ndongo Dia
Yasser Diab
Vakaramoko Diaby
Andreea Diaconescu
Anastasia Diakou
Diawo Diallo
Jean-Simon Diallo
Karidia Diallo
Mawlouth Diallo
Themistoklis Diamantopoulos
L. Diambra
David Diamond
Kevin Diamond
James Diana
Matteo Diano
Sabrina Diano
Honglu Diao
Qiyu Diao
Xianmin Diao
Moussa Diarra
Isabel Dias
Murilo Dias
Patricia Dias
Patrick S. Dias
Rafael Dias
Wilmar Dias da Silva
Newton Dias Filho
Emmanuel Dias Neto
Marcus Dias-Souza
Francisca Diaz
George Diaz
James Diaz
Jose Diaz
Keith Diaz
Maureen Diaz
Olivier Diaz
Antonio Crego Díaz
Clara Díaz
David Díaz
Julio Díaz
Mario Díaz
Eva Carolina Diaz Fuentes
Alvaro Diaz Gonzalez
Rochellys Diaz Heijtz
Isabel Díaz Reviriego
Adrian A. Diaz-Faes
Patricia Diaz-Gimeno
Desiree Díaz-Jiménez
Jordi Díaz-Manera

Javier A. Díaz-Ochoa
Antonio Diaz-Quintana
Miguel Díaz-Rodríguez
Paula Diaz-Valencia
Kamran Diba
Daniel Dibaba
Mahmoud Dibas
Michele Dibattista
Pauline Dibbets
Charna Dibner
Wolfgang Dichtl
Fred Dick
Ivy Dick
Thomas Dick
Leah Dickens
Rochelle Dicker
Allan Dickerman
Kathryn Dickerson
Stephan Dickert
James Dickey
Jeffrey Dickhout
Erin Dickie
Melanie Dickie
Tom Dickins
Alexander Dickinson
J. Dickinson
Janis Dickinson
Patsy Dickinson
Sally Dickinson
Christopher Dickman
Petra Dickmann
Thomas Dickmeis
Matt Dicks
James Dickson
Phillip Dickson
Robert Dickson
Daniel Dickstein
Yaakov Dickstein
Finn Diderichsen
A. Didier
Alain Didier
Giulio Didiodato
Sean Didion
Diego Didona
Madeleine Didsbury
Sandra Diebold
Claire Diederich
Kai Diederich
Axel Diederichsen
Amy Diedrich
Duane Diefenbach
Vincent Diego
Alessandra Diehl
Philipp Diehl
Amanda Diekman
Fritz Diekmann
Stephan Diekmann
Igor Diemberger
Bruce Dien
Ed Diener
Kerrilyn Diener
Benjamin Dieplinger
Rudi Dierckx
Ellen Dierenfeld
Susan Dierickx
Marta Dies
Peter Dieser
Susanne Diesner
Rhett Diessner
Ulrike Diesterbeck
Kevin Dieter
Angela Dieterich
Douglas Dieterich
Kai Diethelm
Gregory Dietl
Christopher Dietrich
Daniela Dietrich
Hans Dietrich
Johannes Dietrich
Lars Dietrich
Muriel Dietrich
Susanne Dietrich
Thomas Dietrich
Alia Dietsch
Karl-Josef Dietz
Mathias Dietz
Noella Dietz
Thomas Dietz

Volker Dietz
Mark Dietzko
Antje Dietz-Pfeilstetter
Carmen Diez
J. Diez
Maria Diez-Cirarda
Francisco Diez-Gonzalez
Belén Diezma
Salvatore Difalco
Stephen Difazio
Rosa diFelice
Jay Diffendorfer
Joseph Roland Difranza
Abhijit Dighe
Gabriele Digiammarco
Mariacristina Digilio
Raymond Digiuseppe
David Dignath
Gianni Diguglielmo
Marcel P. Dijkers
Pascale Dijkers
Rick Dijkhuizen
Remco Dijkman
Bauke Dijkstra
Jennifer Dijkstra
Sergey Dikalov
Jimmy Dikeakos
Suzanne Dikker
Serdal Dikmen
Lisabeth Dilalla
Ralph Dileone
Dario Dilernia
Tina Dillahunt-Aspillaga
Joseph Dillard
Kenneth Diller
Norbert Dillier
Daniel Dilling
Angel Dillip
Christina Dillon
Jo-Anne Dillon
Anna Dimberg
Fred Dimenna
Paul Dimilla
Nicola Dimitri
Slavica Dimitrieva
Gabriel Dimitriou
Michelle Dimitris
Gina Dimitropoulos
Kiril Dimitrov
Henrik Dimke
Simon B Dimmitt
Meletios A. Dimopoulos
Leda Dimou
Niki Dimou
Mariapaola D'Imperio
Maria D'Império Lima
A. P. Dimri
Goberdhan Dimri
Andrew Dinardo
Hasan Dincer
Astra Dinculescu
Furkan Dindaroğlu
Kelly Dineley
Bi-Sen Ding
Chuan Ding
Chuanqing Ding
Cody Ding
Dale Ding
Di Ding
Ding Ding
Feng-Hua Ding
Guofang Ding
Guowei Ding
Guoxian Ding
Hongyuan Ding
Hu Ding
Huangen Ding
Jingzhong Ding
Jiu Ding
Jun Ding
Kele Ding
Keyue Ding
Li Ding
Liang Ding
Lin Ding
Ming Ding
Mingzhou Ding
Nenggen Ding

Ning Ding
Ninming Ding
S. G. Ding
Shifei Ding
Shuai Ding
Shumao Ding
Tao Ding
Wei Ding
Wei-Qun Ding
Weixin Ding
Wen-Xing Ding
Xavier Ding
Xiangdong Ding
Xiaopan Ding
Xiaoqiang Ding
Xiaoyu Ding
Xiu-Hao Ding
Yali Ding
Yezhang Ding
Yi Ding
Ying Ding
Yu-Bin Ding
Yuqiang Ding
Yu-Shin Ding
Zhaojun Ding
Zhaotang Ding
Zhengfeng Ding
Zhide Ding
Zili Ding
Mark Dingemanse
Calinda Dingenouts
V. Dinglas
Hugh Dingle
Jonathan Dingwell
Linh Dinh
Quang Toan Dinh
Thu-Ha Dinh
Luciana Dini
Francisco Dini-Andreote
Miriana Dinic
Teresa Dinis
J. A. F. Diniz-Filho
José Alexandre Diniz-Filho
Andreas Dinkel
Randy Dinkins
Gizem Dinler Doganay
Ener Cagri Dinleyici
José Dinneny
Girmaye Dinsa
Mihaela Dinsoreanu
Monica Dinu
Stephen Dinwiddie
Rui Diogo
Francesca Diomede
Marina Diomedi
Michel Dione
Carlo Dionisi Vici
Daniele Dionisio
Francisco Dionisio
Giuseppe Dionisio
George Dionne
Ginette Dionne
Jodie Dionne-Odom
Nicolas Diotel
Franco Dipadova
Richard Dipaolo
John Dipersio
Luisa Dipietro
Ludovico Dipineto
Joanna Dipnall
Sebastian Dippold
Mohamed Dirani
L. Dirix
Marlou Dirks
Ellen Dirkx
Ulrich Dirnagl
Ermias Diro
David J. Disabato
Michelle Discacciati
Dennis Discher
Richard Discipio
Tang Disheng
Nicola Disma
Salvatore Disomma
Francesco Dispenza
Angela Dispenzieri
Sahan Dissanayake
Sunanda Dissanayake

Gaetano Distefano
Assaf Distelfeld
Steven Distelhorst
Sinee Disthabanchong
Ottmar Distl
Luciano Ditacchio
Michael Ditiatkovski
Prudence Ditlopo
Massimiliano Ditroilo
Simon M. Dittami
Ryan Dittamore
Achim Dittler
Elke Dittmann
Emily Dittmar
Manuela Dittmar
Dirk Dittmer
Jessica Dittmer
Keren Dittmer
Neal Dittmer
Anna Dittrich
Ralf Dittrich
Sabine Dittrich
Darshan Divakar
Ajit Divakaruni
Senad Divanovic
Adeline Divoux
A. K. Dixit
Manjusha Dixit
Narendra Dixit
Elijah Dixon
Jenna Dixon
Laura J. Dixon
Peter Dixon
Simon Dixon
Thomas Dixon
Barnaby Dixson
Pedro Diz
Nishtman Dizeyi
Mustafa Djamgoz
Zakia Djaoud
Therese Djarv
Valentin Djonov
Marko Djordjevic
Steven Djordjevic
Luc Djousse
Arif Djunaidy
Mats Djupsjöbacka
Sinisa Djurasevic
Jens Djurhuus
Marija Djuric
Zora Djuric
Thandiwe Dlamini
Darryl D'Lima
Daniel Dlugolenski
Tymoczko Dmitri
Roger Dmochowski
Wojciech Dmuchowski
Bao Do
Lien Anh Ha Do
Loc Do
Mai Do
S. H. Do
Tuyen Do
Ronaldo Do Amaral
Jussara do Carmo
Frederico Simoes do Couto
Fernanda Dotti do Prado
Thomas Doak
Michael Doane
Carlota Dobano
Sina Dobaradaran
Kevin Dobbin
G. Dobbins
Suzanne Dobbinson
Drena Dobbs
Britta Dobenecker
Aneta Dobierzewska
Bruce Dobkin
Bradley Doble
Susanne Dobler
Monika Doblin
Sigita Doblyte
Árpád Dobolyi
Fulufhelo Doboro
Karen Dobos
Anca Dobrean
Sergey Dobretsov
Hana Dobrovolny

Gabriella Dobrowolny
Grażyna Dobrowolska
Radoslaw Dobrowolski
Solomon Dobrowski
Małgorzata Dobrzyńska
Claire Dobson
Fabian Docagne
Guillermo Docena
Virginie Doceul
Gerard Docherty
Paul Docherty
Fernando Docobo-Pérez
Pilar D'Ocon
Tamas Doczi
Ravi Doddapaneni
Mark Dodding
David Dodell-Feder
Laura Dodge
Dejan Dodig
Erich K. Dodson
Matthew Dodson
Steven Dodsworth
Philipp Doebler
John Doelman
Torsten Doenst
Thorsten Doeppner
Adrien Doerig
Johanna Doerr
Paul Doetsch
Tom Doetschman
Jean d'Offay
Eyup Dogan
Hakan Dogan
Aleksandar Dogandzic
Norman Doggett
Alain Doglio
Anthony Dohan
Preeti Dohare
Mohamed Fahmy Doheim
Karen Doherty
Martin Doherty
Thomas Doherty-Bone
Shinya Dohgu
Yasuaki Dohi
Simone Dohle
Katsumi Doi
Kent Doi
Kento Doi
Toshiki Doi
Michel Dojat
Tansel Dokeroglu
Nikolay Dokholyan
Maxim Dokukin
Martin Dokulil
Sara Dolan
Leila Dolatshahil
Marina Dolbeth
Sanda Dolcos
C. Dold
Leona Dold
Barbara Dolegowska
Agnès Doligez
Oleg Dolkart
Andrew Doll
Kemi Doll
Pascal Dolle
Peter Dolman
Vincenza Dolo
Annette Dolphin
Geert Dom
Joanna Domagala-Kulawik
Abraham Domb
Julia Dombrowski
Nina Dombrowski
Mirian Domenech
Leslie Domier
P. B. Dominelli
Xavier Domingo
Michael Domingue
Andre Domingues
Rosa Domingues
Francisco Dominguez
Jose Domínguez
Raúl Domínguez
Gabriel Dominguez-Maldonado
Mev Dominguez-Valentin
Alberto Dominguez-Vicent
Alberto Domínguez-Vicent

Dominik Dominik
Tanja Dominko
Robert Domitrovic
Jacqueline Domjanic
Sarah Domoff
Eric Domyan
Jorge Doña
Morgane Donadel
Marco Paolo Donadini
Carlo Donadio
Márcio Donadio
Kristine Donahue
Timothy Donahue
Erwan Donal
Alex Donaldson
Maria Donaldson
Michael Donaldson
Paul Donaldson
Tiffany Donaldson
Carlos Donascimiento
Pietrella Donatella
Lars Donath
Marc Donath
Abele Donati
Giacomo Donati
Simone Donati
Anthony Donato
Celeste Donato
Silvia Donato
Julian Donaubauer
José Donázar
Lydia Doncarlos
Frank Dondelinger
Don Donderi
Gilbert Donders
Max Donelan
Jennifer Donelson
Elena Donetti
Bi-Cheng Dong
Bin Dong
Chuanjiang Dong
Chunjiaon Dong
Daming Dong
Di Dong
Fei Dong
Feng Dong
Guangheng Dong
Guanghui Dong
Hao Dong
Hao-Ming Dong
Hongxin Dong
Jian Dong
Jianli Dong
Jing-Fei Dong
John Qi Dong
Li Dong
Liang Dong
Lingli Dong
Na Dong
Qiwen Dong
Ruoyu Dong
Shengzhang Dong
Shikui Dong
Shuang-Lin Dong
Shuangling Dong
Siping Dong
Tingting Dong
Wei-Guo Dong
Wenjuan Dong
Xiaoguang Dong
Xiaoli Dong
Xiao-Qiao Dong
Xin Dong
Yan Dong
Yan-Jun Dong
Yanping Dong
Yi Dong
Yun-Wei Dong
Zaijie Dong
Zheng Dong
Zhenying Dong
Zhicheng Dong
Zhijie Dong
Zhongyun Dong
Paola Dongiovanni
Guo Dongliang
Yashad Dongol
Wang Dongshu

Marco Donia
Lorenzo Donini
Mieke Donk
Dirk Donker
Liesje Donkin
Deborah Donnell
Martin Donnelley
Eve Donnelly
Ryan Donnelly
Tam Donnelly
Albert Donnenberg
Kristian Donner
Josef Donnerer
Sandra Donnini
Stacey Donofrio
Alberto d'Onofrio
Brian D'Onofrio
Annie Donoghue
Phil Donoghue
Maura Donohue
Nikos Donos
David Donoso
Brittney Donovan
Graham Donovan
Mary Donovan
Michael Donovan
Curtis Donskey
Frede Donskov
Miae Doo
Steve Doo
Doug Doohan
William Dooley
Hugo Dooner
Mark Dooner
Amin Doosti Irani
Abolfazl Doostparast
Gabriel Dorado
Cristina Dorador
Kavitha Dorai
Sathya Doraiswamy
Elizabeth Doran
Neal Doran
Simon Doran
Todd Doran
Mathieu Doray
Robert Dorazio
Emilie Dordet-Frisoni
Fernanda Dorea
Paula Dore-Duffy
Haxhiademi Dorela
Audrey Dorélien
Kevin Dorfman
Cristian Doria
Alan Dorin
Dusana Dorjee
Thomas Dorlo
Franziska Dorn
Tim Dornan
Mathew Dornbush
Jo Dorning
Salvatore D'Oro
Dan Dorobantu
Loredana Dorobantu
Olga V. Dorofeeva
Augustine Doronila
Shirin Doroudgar
Jan Dörr
Marcus Dörr
Anne Dorrance
Jan Dörrie
Caroline Dorsen
G. D'Orsi
Richard Dortch
Laurent Dortet
Pemra Doruker
David Dorward
Izabel dos Reis
Erika dos Santos
Julio dos Santos
Laurita dos Santos
Odelta dos Santos
Patricia Dos Santos
Serge Dos Santos
Jair Guilherme dos Santos Junior
J. Dose
Bhavya Doshi
Jimit Doshi
Reena Doshi

Brian Doss
Michael Xavier Doss
Ines Dossa
Marcos Dossantos
Michelle Dossett
Ejovwoke Dosunmu
Dror Dotan
Keri Dotson
Richard Doty
Lingling Dou
Q. Ping Dou
Qi Dou
Benoît Doublet
Vincent Doublet
Mikhail Doubrovin
Jay Doucet
Florence Doucet-Populaire
Karim Douch
Anastassios Doudoulakakis
Daniel Douek
Dawn Dougherty
Eric Dougherty
Patrick Dougherty
Paul Dougherty
Jamie Douglas
Margaret Douglas
Marlis Douglas
Peter Douglas
Richard Douglas
Ross Douglas
Timothy Douglas
Trevor Douglas
Dominique Douguet
Ilias Doulamis
Agapi Doulgeraki
Claudie Doums
Mojca Doupona-Topic
Linda Douw
Katja Douze
Prusky Dov
Sinisa Dovat
Guy Dove
Andraž Dovnik
Constantine Dovrolis
Alan W. Dow
Dorothy Dow
Runjun Dowarah
Patrick Dowd
Marsha Dowda
Steven Dowdy
Floyd Dowell
Nicholas Dowell
Arnaud Dowkiw
Dar Dowlatshahi
Damian Dowling
Jennifer Dowling
Ryan Dowling
Soo Downe
Brian Downer
Laura Downie
Colleen Downs
Joni Downs
Christopher Dowrick
Nicholas Dowson
Margaret Doyle
Sean Doyle
Vinson Doyle
Irini Doytchinova
Ann Dozier
Mikhail Dozmorov
Charles Dozois
Tiina Sairanen
Tomas Drabek
Anna Drabik
Harry Drabkin
Emmanuel Drabo
Maxime Drabo
Peter Dracatos
Arlene Drack
Giulio Draetta
Marcin Drag
Tommaso Dragani
Ioannis Dragatsis
Luciano Drager
Lorenzo Drago
Silvano Dragonieri
Georgia Drakakaki
Alison Drake

Bettina Drake
Brandon Drake
Isabel Drake
Nick Drake
Robert Drake
Shaynoor Dramsi
Mark Dransfield
Jarosław Drapała
Pierre Drapeau
Catherine Draper
Heather Draper
Elena Dreassi
Didier Dreau
Anna Dreber
Maik Drechsler
Sven Drefahl
Dayna Dreger
Theo Dreher
Michele Drehmer
Robert Dreibelbis
Michael C. Dreiling
Dimitri Drekonja
Thomas Drepper
Martin Dres
Hannah Drescher
Falko Dressler
Michael Dretsch
Joshua Drew
Kevin Drew
Paul Drew
Robert Drew
Chris Drewell
Yvonne Drewes
Daniel Drewniak
Florian Dreyer
Rachel Dreyer
Jean-Michel Drezen
Patrik Drid
Djamel Drider
Michael Drielsma
Arnold Driessen
Pete Driezen
William Driggers
Reza Drikvandi
Timothy Driscoll
Adel Driss
Barbara Dritschel
Ian Driver
Jane Driver
David Drobes
Anna Drohomirecka
Marc Drolet
Liv Drolsum
Casey Droscha
Elefterios Drosinos
William Drosopoulos
Elena Drosopoulou
Régen Drouin
Bogna Drozdowska
Stanislaw Drozdz
Dorothee Drucker
Martin Drucker
Tilman Drueke
Thomas Druetz
Marjan Drukker
Todd Druley
Zuzanna Drulis-Kawa
Heidi Drummer
Eleanor Drummond
Marcela Drummond
Shelly Druyan
Maurice Druzin
Jonathan Dry
Nick Drydakis
Matthew Dryden
Rachel Dryer
Julia Drylewicz
J. Drymon
Linda D'Silva
Nisha D'Silva
Bin Du
Chenyu Du
Hai-Ning Du
Hewei Du
Huahua Du
Huaidong Du
Jianyang Du
Jing Du

Jinming Du
Juan Du
Jun Du
Ke-Lin Du
Lei Du
Meng Du
Muqing Du
Ning Du
Peng Du
Ping Du
Pufeng Du
Qingzhang Du
Rose Du
Ruofei Du
Shaofu Du
Shufa Du
Weihua Du
Wenbin Du
William Du
Xiao-Bo Du
Xiaoming Du
Xuye Du
Yan Du
Yanzhi Du
Yi Du
Yongjun Du
Yuchuan Du
Zhangliu Du
Zhibin Du
Zunfeng Du
Stefan du Plessis
Jean Baptist du Prel
Patrick Du Souich
Joss Du Toit
Nur Duale
Fenghai Duan
Guangcai Duan
Jingyue (Ellie) Duan
Jizhou Duan
Lei Duan
Lide Duan
Qiang Duan
Shiwei Duan
Wenzhen Duan
Xuewu Duan
Zhisheng Duan
Aida Duarte
Caro Duarte
João Valente Duarte
Regina Duarte
Simone Duarte
Jan Henk Dubbink
Caroline Dubé
Karine Dubé
Caroline Dubertret
Ashok Dubey
Sunil Chandra Dubey
Venketesh N. Dubey
Michael Dubick
Anna Dubiel
Steven Dubinett
David Dubnau
Margarita Dubocovich
Eric Dubois
Grant Dubois
Jessica Dubois
Nicolas Dubos
Julie Duboscq
A. Dubovi
Sarah Dubrac
Bertrand Dubreucq
Alexandra Dubrovina
Stephen Dubsky
Constance Dubuc Dubuc
Jean Dubuisson
Laurent Dubuquoy
Frank Duca
Mariette Ducatez
Fulvio Ducci
Jordi Duch
Caroline Duchaine
Jacques Duchateau
Simon Ducheix
Johan Duchene
Sebastian Duchene
Kyle Duchman
Jonathan Duckett
Susan Duckett

Rachel Duckham
John Duckitt
Julien Duclay
Frédéric Ducongé
Christian Ducrot
Andrew Ducruet
Cesar Ducruet
Lorenzo Ductor
Dan Duda
Ernö Duda
Amel Dudakovic
Jozsef Dudas
Travis Dudding
William Duddy
Vikas Dudeja
Chris Dudgeon
Omaya Dudin
Dawn Dudley
Edward Dudley
Edward G. Dudley
Jaquelin Dudley
Robert Dudley
Morten Dueholm
Ej Duell
Martin Duennwald
Andreas Duenser
Nathalie Duerinckx
Macro Duering
Penelope Duerksen-Hughes
Adam Duerr
Ann Duerr
Felix Duerr
Ralf Duerr
Rebecca Duerr
Daniel Duerschmied
Stephan Duetzmann
David Dufeau
Janet Dufek
Martina Düfer
Jacques Duff
Thomas Duff
Tyler C. Duffield
Paul Duffin
Charles Duffy
Darragh Duffy
David Duffy
Kevin Duffy
Michael Duffy
Laurence Dufourny
Mike Dugan
Lara Dugas
Martin Dugas
Tammy Dugas
Vivienne Duggan
Nick Duggett
Eva Dugoff
Melissa Duhaime
Radboud Duintjer Tebbens
Ashley Duits
A. Duittoz
D. C. Duives
Gwendal Dujardin
Jean-Claude Dujardin
Bernard Dujon
Clifford Duke
Dawn C. Duke
Stephen Duke
Jonathan Duke-Cohan
Sean Dukelow
Rajendra Dulal
Thierry Dulermo
Ana Dulgheroff
Angela Dulhunty
Jennifer Dulin
Arianna D'Ulizia
Robin Dullaart
Lisa Dulli
Aidan Dumaisnil
Pierre-Yves Dumas
William Kwadwo Dumenu
Korsi Dumenyo
Jerome Duminil
Roger Dumke
Reinhard Dummer
Krishna Dummula
Remi Dumollard
Julie Dumond
Georgette E. Dumont

Jerome Dumortier
Dorothea Dumuid
Jon Andoni Duñabeitia
Joshua Dunaief
Christophe Dunand
Murielle Dunand
Neil Dunavin
Johanna Dunaway
Ewan Dunbar
James Dunbar
Miranda Dunbar
Alison Duncan
Kent Duncan
Margaret Duncan
Neil Duncan
Randall Duncan
Robert Duncan
Bumin Dundar
Ekrem Dundar
Ebru Dündar Yenilmez
Ivan Dungan
Robert Dungan
Huzefa Dungrawala
Ian Dunham
Yarrow Dunham
David Dunigan
Zackary Dunivin
Caroline Dunk
Christine Dunkel-Schetter
Jason Dunlop
Adam Dunn
Alexander Dunn
Dell P. Dunn
Elizabeth Dunn
Graham Dunn
Heather Dunn
James Dunn
Julia Dunn
Kelly Dunn
Louise Dunn
Peter Dunn
Eileen Dunne
John Dunne
Matthew Dunne
Simon Dunne
Jake Dunning
Kylie Dunning
Gary Dunny
Christopher Dunsby
Joseph Dunsmoor
Carl Dunst
Ken Dunton
Nancy Dunton
Veasna Duong
Vu Duong
Sy Duong-Quy
Stéphane Dupas
George J. Dupaul
Elisabeth Dupin
Anne Duplouy
Emmanuel Dupont
Nicolas Dupont
Sylvain Dupont
William Dupont
Cydney Dupree
Thierry Dupressoir
Luc Dupuis
Juan Manuel Dupuy
Lionel Dupuy
Jazmin Duque
Tina Dura
Salvador Dura-Bernal
Mithila Durai
Josefina Duran
Miguel A. Duran
Nicholas Duran
Benoit Durand
Jean-Baptiste Durand
Manuela Durando
Miquel Duran-Frigola
Ana Duran-Pinedo
Mario Durán-Prado
Ana Durán-Quesada
Szonya Durant
Flore Duranton
Paulo Durao
Marcelino Durão Junior
Francesca Duraturo

Madeleine Durbeej
Sushmitha Durgam
Flavia Durgante
David Durham
Emily Durham
Michael Durheim
David Durica
Olivier Duriez
Rita Marqueti Durigan
Heinjo During
Madeleine Durkee
Magdalena Durlik
Zoey Durmic
Dion G. Durnford
Robert Durrant
David Durrheim
Klaus Dürrschmid
Erin Dursa
Lisa Durso
Pierpaolo D'Urso
Andrea Durst
Mehtap Dursun
Georg Duscher
Albert Duschl
Adam Dušek
Robert Dusenka
Jonathan Dushoff
Christian Dussault
Stephane Dussert
Olivier Dussurget
Janine Dutcher
Martine Duterque-Coquillaud
Geraldine Duthe
Heverton Dutra
Lauren Dutra
Tania Dutra
Walderez Dutra
Yago Dutra
Manjul Dutt
Arijita Dutta
Debargh Dutta
Dipankar Dutta
Haimonti Dutta
Pradip Kumar Dutta
Rinku Dutta
Samikshan Dutta
Trishna Dutta
Atanu Duttaroy
James Dutton
Mary Dutton
Raimund Dutzler
Benjamin Duval
Cedric Duval
Dawn Duval
Mathieu Duval
Romain Duval
Melvin Duvall
Bernard Duvivier
Bram Duyx
Petr Dvorak
Anna Dvorkin-Gheva
Nam Dwangho
Melinda Dwinell
Michael Dwinell
Alok Dwivedi
Hari Dwivedi
Puneet Dwivedi
Rinshu Dwivedi
Sadhana Dwivedi
Shailendra Dwivedi
Sourabh Dwivedi
Upendra Dwivedi
Yogesh Dwivedi
Duah Dwomoh
Shari Dworkin
Peter C. Dworschak
Amy Dworsky
Andrew Dwyer
Dominic Dwyer
Julie Dwyer
Noelle D. Dwyer
Simon Dyall
Andrzej Dybus
Amalie Dyda
Andrew Dyer
David Dyer
Rodney Dyer
William Dyer

Emily Dykhuizen
Martin Dyrba
Lars Dyrskjot
Tomasz Dysarz
Ben Dyson
Louise Dyson
Nicholas Dyson
Paul Dyson
Sue Dyson
Rhonda Dzakpasu
Yvonne Dzal
Jaroslaw Dziadek
Rosemary Dziak
Mohd Dziauddin
Marta Dziedzicka-Wasylewska
Katarzyna Dziendzikowska
Thomas Dziubla
Anastase Dzudie
Mohd Dzulkipli
Laura Dzurec
Brian Dzwonkowski
Bogdan Dzyubak
