# Supplementary material for: PLOS ONE 2017 Reviewer and Editorial Board Thank You
Source: PLoS One. 2018 Mar 15;13(3):e0194158. doi: 10.1371/journal.pone.0194158 (PMC5854357; doi:10.1371/journal.pone.0194158)
Supplement: S2 Reviewer List — Alphabetical list of 2017 PLOS ONE peer reviewers (E-J). (PDF) [file pone.0194158.s004.pdf]

PLOS ONE would like to thank all those who reviewed on behalf of the journal in 2017:

Emily E.
Aidin E. Pour
Michael Eadon
Anne Eady
Kyle Eagen
George Eapen
Bertrand D. Eardly
Gammon Earhart
Julie Earl
Scott Earley
Kate Earlt
Julie Early
Dietrich Earnhart
Brian Earp
Christine East
Marion East
Alan Eastman
Alison Eastman
Charmane Eastman
Roger Easton
Gillian Eastwood
Narelle Eather
Jeffrey Eaton
Jennifer Eaton
Kathryn Eaton
Nicholas Eaton
Simon Eaton
William Eaton
Julian Eaton-Rye
Daniel Eaves
Ada Eban-Rothschild
Mitsuhiro Ebara
Cathrine Ebbing
Gregory Ebel
Mark Ebell
Hermann Eberl
Bernd Eberle
Kyle Eberlin
Livia Eberlin
Jeffrey Ebersole
Isaac Eberstein
David Ebert
Dieter Ebert
Natalie Ebert
Thomas Ebert
Timothy Ebert
Tom Ebert
Kris Ebi
Aya Ebihara
Jeff Eble
Scott Eblen
Kevin Eboigbodin
Quteba Ebrahem
Somayeh Ebrahimi-Barough
Mohammad R. Ebrahimkhani
David Eby
Sarah Eby
Caniglia Ec
Arnaud Echard
Manuel Echarte
Blas Echebarria
Karen Echeverri
Pablo Echeverria
Craig Echt
Wolfgang Ecke
Matthew Eckelman
Maryellen Eckenhoff
Roderic Eckenhoff
John Ecker
Frank Eckerdt
Beate Eckes
Benjamin Eckhardt
Hans Eckstein
Torsten Eckstein
Anastassios Economou
Nicholas-Tiberio Economou
Heath Ecroyd
Hanie Edalati

Chandrakanth Edamakanti
Kazuya Edamura
Saadia Eddahibi
Brian Eddie
Thomas Eddinger
David Eddins
Michael Eddleston
Tyler Eddy
Brad Edelman
Elazer Edelman
Shimon Edelman
Leonard Edelstein
Guinevere F. Eden
Kristin Eden
Howard Edenberg
Anne Eder
Klaus Eder
Rosy Edey
Graham Edgar
Albert Edge
Gudrun Edgren
Nina Edin
Neranjan Edirisinghe
Myriam Edjlali
Melissa Edler
Suzanne Edmands
Gary Edmond
Andrew Edmonds
Kyle Edmunds
Peter Edmunds
Alemayehu Edo
Norbert Edomah
Sheikh Edrisi
Rolf Edvardsen
David Edvardsson
Nils Edvardsson
Alexis Edwards
Amy Edwards
Autumn Edwards
David Edwards
Frances Edwards
James Edwards
Jeffrey D. Edwards
Jeremy Edwards
John Edwards
Kate Edwards
Nathan Edwards
Paul Edwards
Robert Edwards
Sandra Edwards
Amanda Edworthy
Carolyn Ee
Jonathan Peter Ee
Burak Efe
Alejo Efeyan
Felix Effenberger
Suhendry Effendy
Charles Efferson
Thomas Efferth
Jimmy Efird
Nathan Efron
Nikolaos Efstathiou
Eftekhar Eftekharpour
Dimitrios Efthymiou
Dilfuza Egamberdieva
Jonathan Egan
Patrick Egan
Thomas Egan
V. L. Egarter
Naohiro Egawa
Catherine Egbe
Jan Egberts
Chinaza Egbuta
Markus Ege
Grace Egeland
Paul Egeler
Agneta Egenvall
Tammy Eger
Uzochukwu Egere
Monika Egerer
Louise Egerton-Warburton
Ulrika Egertsdotter
Torbjørn Eggebø
Lothar Eggeling
Roger Eggen
Bernhard Egger
Garry Egger
Karl Egger

Matthias Egger
Erika Eggers
Lori Eggert
Thomas Eggert
Stephen Eggins
Ian Eggleston
Karen Eggleston
Paul Eggleton
Rosalind Eggo
Shahin Eghbalsaied
Andrea Egizi
Markus Egli
Doris Eglseer
Tobias Egner
Tewodros Eguale
Akiko Eguchi
Hisashi Eguchi
Kazuo Eguchi
Takashi Eguchi
Yawara Eguchi
Yutaka Eguchi
Luís Enrique Eguiarte
Víctor Eguíluz
Hiroshi Egusa
Thomas Egwang
Tatsuya Ehara
N. D. B. Ehelepola
Kaylena Ehgoetz Martens
Johannes Ehinger
Jens Ehmcke
Sabrina Ehnert
Måns Ehrenberg
Ann Ehrenhofer-Murray
Hannelore Ehrenreich
Miriam Ehrensaft
Deborah Ehrenthal
Dana Ehret
Georg Ehret
Anja Ehrhardt
Marissa Ehringer
Frédéric Ehrler
Laurent Ehrlich
Samantha Ehrlich
Lutz Eichacker
Patric Eichelberger
Patrick Eichenberger
Ursula Eichenlaub-Ritter
Susan Eicher
Anne Eichmann
Naomi Eichorn
Catherine Eichwald
Olivier Eichwald
Michael Eickermann
Olaf Eickmeier
Shaker Eid
Arne Eide
Ivar Eide
Thijs Eijsvogels
M. Eikermann
Matthias Eikermann
Ichihara Eiki
Alexander Eiler
Eric Eils
Johannes Eimer
Elisabet Einarsdottir
Gisli Einarsson
Susan Dana Einbinder
Wolfgang Einhäuser
Hassan Eini-Zinab
Shahram Eisa-Beygi
Avraham Eisbruch
Damon Eisen
Judith Eisen
Jonathan Eisenback
Dan Eisenberg
Tobias Eisenberg
Emily Eisenhart
Michael Eisenhut
Tory Eisenlohr-Moul
Andreas Eisenreich
Wolfgang Eisenreich
David Eisenstat
Sarah Eisenstein
Andreas Halgreen Eiset
Yoshinobu Eishi
Alvin Eisner
Manuel Eisner
David Eissenstat

Alexander Eitel
Eduardo Eizirik
Katrine Ejlerskov
Weronica Ek
Asif Ekbal
Andrew Ekelem
Damian Ekiert
Marc Ekker
Stephen Ekker
Anders Eklund
Aron Eklund
Elizabeth Eklund
Björn Ekman
Francis Eko
Mohsen Ekramian
Torbjorn Ekrem
Mirjam Ekstedt
Obi Ekwenna
Oi Ekwunife
Safwan El Assad
Ikbale El Ayachi
Jamila El Baghdadi
Regina El Dib
Magdy El Ekiaby
Ahmed El Hady
Ihab El Hajj
Maguy El Hajj
Nady El Hajj
Nour El Helou
Thaqif El Khassawna
Hisham El Moaqet
Issam El Naqa
Mohamed Réda El Ochi
Rashika El Ridi
Faten El Sayed
Maha El Tantawi
Bishoy El-Aarag
Dina El-Agamy
Shokrollah Elahi
Kit Elam
M. Elamin
Jonathan E-Lanao
Ramu Elango
Vijaikrishnah Elango
Ravikrishnan Elangovan
Hosam Elansary
Hosam O. Elansary
Afaf El-Ansary
Martyna Elas
Saeed El-Ashram
Tatiana El-Bacha
Mohamed El-Badry
Husni Elbahesh
Lama Elbahlawan
Mohamed Elbanhawi
Musa El-Barghouthi
Heba Elbasiouny
Batya Elbaum
Alexis Elbaz
Ayman El-Baz
Hossam El-Beltagi
Jorina Elbers
Tarek El-Bialy
Michal Elboim-Gabizon
Diana Elbourne
Vasco Elbrecht
L. Mark Elbroch
Adrian Elcock
Ebtehal El-Demerdash
Benjamin Elder
James Elder
Rudi Van Eldik
Felice Elefant
John Elefteriades
Ilias Elefterohorinos
Klaus Elenius
Mohamed El-Esawi
Elis Cristina Eleutherio
Hazem Elewa
Karen Eley
Muhammed Elfaituri
Mohammed El-Faki
Ali El-Far
S. Elfekih
J. Elfenbein
Achim Elfering
Malin Elfstrand
Deena El-Gabry

Mohamed Elgendi
Ahmed El-Geneidy
Bernice Elger
Christian Elger
Maged El-Ghannam
Mona El-Ghazaly
Lynda Elghazi
Mona El-Ghoneimy
Tarik Elhadd
Mohamed Elhadidy
Nazira El-Hage
Jon Elhai
Ahmed Elhakeem
Hamdy El-Hakim
Sarah Elhamiani Khatat
Salah El-Hendawy
Heithem El-Hodiri
Khaled Elhosiny
Leonardo Elia
Maurizio Elia
S. Eliades
Daniel Elias
Harold Elias
Marlene Elias
Rosilene Elias
Erika Eliason
Okan Elibol
Brian Eliceiri
Fernando Elijovich
Andrey Eliseyev
Stella Elkabes
Samer El-Kadi
Charles Elkan
Ardith El-Kareh
Sherif El-Khamisy
Ziad El-Khatib
Che Elkin
Peter Elkin
Robert Elkin
Mitchell Elkind
Marwan El-Koussy
Shawn Ell
R. Ellahi
Ratnavalli Ellajoysula
Abd El-Latif
Elizabeth Elle
Mai-Britt Bjørklund Ellegaard
Ingrid Ellen
Clare Ellen Edge
Susan Ellenberg
David Ellenberger
Franziska Eller
Michael Eller
Sandrine Ellero-Simatos
Christina Ellervik
Stacey Ellery
Lesley Ellies
O. Ellingsen
Kristian Ellingsen-Dalskau
Helga Ellingsgaard
Sally Ellingson
Sascha R. Ellington
Digby Elliott
Gillian Elliott
Ivo Elliott
James Elliott
Kyle Elliott
Mark Elliott
Michael Elliott
Roberrt Elliott
Ross Elliott
Steve Elliott
Timothy Elliott
Alissa Ellis
Ben Ellis
Jessica Ellis
Jonathan Ellis
Katherine Ellis
Rachel Ellis
Robert Ellis
Steve Ellis
Amanda Ellison
Christopher Ellison
Richard Ellison
Stephan Ellmann
Walid Elloumi
Ranjith Ellur
Nicholas Ellwanger

Elizabeth Ellwood
Mai Elmallah
Peggy El-Mallakh
Ahmed Elmarakby
Mahmoud El-Mas
Moamen Elmassry
Johan Elmberg
Eva-Maria Elmenhorst
Greg Elmer
Kathryn R. Elmer
Jocelyn Elmes
Mostafa El-Mokadem
Andrew Elmore
Donald Elmore
Khalil Elmorjani
Khalifa Elmusharaf
Reem Elnaga
Mohamed El-Naggar
Arne Elofsson
Marc Eloit
Sunny Eloot
Fernando Elorriaga-Verplancken
Roberto Elosua
Catarina Eloy
Mona Elrefaey
Rasha El-Rifai
Wael El-Rifai
Orna Elroy-Stein
Mayada Elsabbagh
Marwan El-Sabban
A. A. Elsadany
Mattijs Elschot
Tobias Else
Corinna Elsenbroich
Sigrid Elsenbruch
Ruth Elsey
Tracy Elsey-Quirk
Hatem Elshabrawy
Mostafa Elshahed
Hossam Elshamaa
Samer Elshikh
Philip Elsinga
Ann Elsner
Ahmed El-Sohemy
Ari Elson
Malte Elson
Richard Elson
J. Elster
Brendan Elsworth
John Elsworth
Mohamed El-Tahan
E. Eltahir
Maha El-Tantawi
Mahmoud El-Tarabany
Khaled El-Tarabily
Mohamed El-Tholoth
Moataz Eltoukhy
Arif Elvan
Rune Elvik
Rachel Elward
Jean Elwing
Bert Ely
Tobias Elze
Theo Elzenga
Abdel-Naser Elzouki
Ashkan Emadi
Nicholas Embleton
Marina Emborg
Mildred Embree
Livinus Emebiri
Pinar Emecen-Huja
Ben Emery
Sarah Emery
Venereau Emilie
Amanda Emke
Patricia Emmanuel
Koilmani Emmanuvel Rajan
Johannes Emmerling
Michael Emmert-Buck
Susan D. Emmett
Jennifer Emond
Patrick Emond
Scott Emrich
Eve Emshwiller
Michael Emslie
Richard Emslie
Gideon Emukule
Abdul-Hamid Emwas

Francis Enane
E. Enato
Elena Enax-Krumova
John Encandela
Gabriele Ende
Jeffrey Endelman
Joyce Endendijk
Heiko Enderling
Eva Enders
Marie-Jose Enders
Nancy Endersby
Carola Endes
Yvonne Ende-Verhaar
Masayuki Endo
Tomoko Endo
Jérôme Endrass
Johann Endres
Egrinya Eneji
Ulrika Enemark
Cordula Enenkel
Soeren Enge
Ingunn Engebretsen
Knut Engedal
Florian Engel
Jerome Engel
Marion Engel
Scott Engel
Tatiana Engel
Christopher Engeland
Hanna Engelberg-Kulka
Jürgen Engele
Georg Engelhard
Martin Engelhard
Brabara Engelhardt
Maren Engelhardt
Monika Engelhardt
Sacha Engelhardt
Simon Engelhart
Daniel Engelman
Peter Engelmann
Tim Engels
Yvonne Engels
David Engelthaler
Bevin Engelward
Denis-Alexander Engemann
Kristine Engemann
Richard Engh
Ephrem Engidawork
Bryant England
Patrick England
Sarah England
S. Walter Englander
Elizabeth Engle
Kevin Englehart
William English
Erin Englund
David Engman
Raymond Enke
Walther Enkerlin
Doyo Enki
Jost Enninga
Irene Ennis
Roland Ennos
Atsushi Enomoto
Hirayuki Enomoto
Yoshimi Enose-Akahata
Francesco Enrichetti
Ipek Ensari
David Ensing
Alexander Ensminger
Emilia Entcheva
Andreas Entenmann
Alireza Entezari
Vahid Entezari
Sonja Entringer
Sabrina Epiphanio
G. Epler
Elliot Epner
Michael Epperly
Timo Eppig
Michel Eppink
Meira Epplein
Julien Epps
Sacha Epskamp
Jonathan I Epstein
Richard Epstein
Jérôme Epsztein
Francois Erard

Liesl Erb
Tobias Erb
Belkis Erbas
Chistopher Erbes
Nülüfer Erbil
Eyup Ercan
Daniel Erces
Ozgur Ercetin
Laura Ercoli
Ayse Ercumen
Fabian Erdel
Sevilay Erden
W. Scott Erdley
Erland Erdmann
Friederike Erdmann
Jason Erdmann
Ks Erdmann
Laszlo A. Erdodi
Nelzo C. Ereful
Metin Eren
Zehra Eren
Hakan Erenel
Ali Kemal Erenler
Ramazan Erenler
Alexander Ereskovsky
Gisela Erf
Amir Erfani
Holger Erfle
Sarina Ergas
Bülent Ergönül
Gul Ergör
Gulfem Ergun
Osman Ergun
Koray Ergunay
Hans Erhard
Hall Eric
Salas Eric Ariel L.
Mary Erich
Andrew Erickson
Benjamin Erickson
Loren Erickson
Mary Erickson
Michelle Erickson
Nick Erickson
Peter Erickson
Richard A. Erickson
Simon Erickson
Todd Erickson
Aaron Ericsson
Daniel Ericsson
Göran Ericsson
Leonardo Erijman
Erik Eriksen
Jason Eriksen
Anneli Eriksson
Henrik Eriksson
Kimmo Eriksson
Maria Eriksson
Marie Eriksson
Niclas Eriksson
Per-Olof Eriksson
Ann-Kristin Eriksson Wiklund
Albert Erives
Mert Erkan
Tulay Erkan
Omer Erkuner
Armin Erlacher
Jon Erlandson
Martin Erlandson
Arvid Erlandsson
David Erle
Adrian Erlebacher
Judith Erlen
Silvio Erler
Deborah Erlich
Batu Erman
Luca Ermini
Jochen Ernst
Kacey Ernst
Monique Ernst
Oliver Ernst
Raffael Ernst
Udo Ernst
Jussi Eronen
Dirk Erpenbeck
Youssef Errami
Gian Erre
Enzo Errichetti

Thomas Errico
Roberto Erro
Ali Ersen
Klaus Ersfeld
Andrea Ertani
Özlem Ertekin
Cesim Erten
Michael Ertl
Ömer Faruk Ertuğrul
Ali Erturk
Gizem Ertürk
Jan Jaap Erwich
Heather Erwin
Hanno Erythropel
Isabel Escada
Ricardo Escalante
Tania Escalante
Arthur Escalas
Kevin Escandón-Vargas
Ricardo Escarcega
Gabriel Escarela
Christine Escargueil
Lukas Esch
Joerg Eschweiler
Alejandro Escobar
Carolina Escobar
Gabriel Escobar
Juan Escobar
César Escobedo-Bonilla
Cam Escoffery
C. Escolano
Gines Escolar
Javier Escudero
Chiedu Eseadi
J. A. Esfahani
Dani Eshel
Gidon Eshel
Adiren Eshraghi
Mehrzad Eskandari
Mona Eskandari
Farsad Eskandary
Antoine Eskander
Anu Eskelinen
Anne Eskild
Mohammed Eslam
Amy Esler
Hanif Esmail
Eric Espagne
Edgar Espana
Alberto Espay
Luca Espen
Eric Espensen
Toomas Esperk
Manuel Esperon-Rodriguez
Daniel Espes
Antonio Espín
Andres Espindola
Carlos Espinel
Ana Espino
Alexander Espinosa
Claudia Espinosa
Victor Espinosa
Alejandro Espinosa de los Monteros
Francisco J. Espinosa-Garcia
Carmen Espinosa-Gongora
Manuel Espinosa-Urgel
Kim Espinoza
Mario Espinoza
Russ Espinoza
Edward Esplin
Bruno Esporcatte
Alfonso Esposito
Douglas Esposito
Elga Esposito
Felice Esposito
Giulia Esposito
Irene Esposito
Lauren Esposito
Simone Esposito
Johan Espunyes Nozières
Jeremy Esque
Antonio Esquinas
M. Paz Esquivias
Kobina Essandoh
Soha Essawy
Karyn Esser
Jeroen Essers

M. Essex
Michael Essington
Tara Essock-Burns
Beverley Essue
Samuel Estabrooks
Jennifer Estall
Elizabet Estallo
Irene Esteban
Jaime Esteban
Nicole Esteban
Irene Esteban-Cornejo
Jean-Pierre Estebe
Lianne Estefan
Matt C. Estep
Irina Esterlis
Zachary Estes
Pierre-Olivier Esteve
Maria Esteve-Gasent
Pedro Esteves
Satu Estlander
Alba Estrada
Vicente Estrada
Paulina Estrada-de los Santos
Agustin Estrada-Peña
Dieferson Estrela
Nicole Estrella
Ramon Estruch
Vicente Estruch
Alex Estudillo
Catherine Etchebest
Jason Etchegaray
Paula Eterovick
Steen Ethelberg
Tim Etheridge
Nicole Etherington
Josee-Lyne Ethier
Tadele Eticha
Lucie Etienne
Olivier Etienne
Xiaoli Etienne
Ilker Etikan
Nilay Etiler
Masumi Eto
David Eton
Gertjan Ettema
Andres Etter
Ron Etter
Matthew Etterson
Stephanie Etting
Jaione Etxeberria
Amsalem Etya
Anthony Etyang
Joset Etzel
Ohad Etzion
Harald Euler
Carola Eva
C. Éva
Andrew Evan
Pavlos Evangelidis
Adriane Evangelista
Laura Evangelista
Alex Evans
Andrew Evans
Annette Evans
Bruce Evans
Charlesnika Evans
Charlotte Evans
Christopher Evans
Colin Evans
Holly Evans
Jason Evans
Jennifer Evans
John Evans
Jon Evans
M. Blair Evans
Marie Evans
Mark Evans
Megan Evans
Melissa Evans
Peter Evans
Richard Evans
Robert Evans
Roger Evans
Scott Evans
Tanya Evans
Tim Evans
Tom Evans
Ayamdooh Evans Nsoh

Tshibangu Evariste Kabamba
Lori Evarts
Kimberley Evason
E. Didem Evci (K)
Nikolai M. Evdokimov
Patrick Even
Sergine Even
Andrew Evens
Stephanie Everaerts
Lis Evered
Paul Everest
Jessica Everett
Thomas Everett IV
Sydney Everhart
Andrea Evers
Jochem Evers
Richard Evershed
Regula Everts
Elena Evguenieva-Hackenberg
Leslie Evinger
Serge Evrard
G. Evtugyn
Arne Ewald
Hannah Ewald
Sarah Ewald
Victoria Ewan
William Eward
Heidi Ewen
Joshua Ewen
Katie Ewer
Michael Ewers
Christine Ewers-Saucedo
Alan Ewert
Marcela Ewert
Temitope Ewete
Louise Ewing
Rob Ewing
Filippos Exadaktylos
Daniel Exeter
Christopher Exley
Paul Expert
Cassandra Extavour
Myron Exum
Nir Eyal
Willard Eyestone
Thomas Eykyn
Florent Eymard
Isobel Eyres
Exequiel Ezcurra
Ikenna Eze
Lara Ezquerra
Absalom Ezugwu
Yoichi Ezura
Sayeh Ezzikouri
Kay Faaberg
Marijke Faas
Kate Faasse
Cornelius Faber
Irene Faber
Mieke Faber
L. W. Faberowski
Alessandra Fabi
Francois Fabi
Carol Fabian
Daniel Fabian
Gianluca Fabiano
Mario Fabiilli
Marchesoni Fabio
Christelle Fablet
Pierre-Henri Fabre
Stephane Fabre
Emma Fabrega
Claude Fabre-Nys
Marc Fabri
Gemma Fabriàs
Marek Fabrika
Linda Fabris
Luca Fabris
Fabrizio Fabrizi
Lorenzo Fabrizi
A. T. Fabro
Sebastian Faby
Arnoldo Facanha
Francesco Facchiano
Luca Facchinelli
Gaetano Facchini
Andrea Luigi Facci
Antonion Facciorusso

Shelley Facente
Daniele Fachinetti
Antonio Faciola
Frank Fackelmayer
Andrea Facoetti
Pam Factor-Litvak
S. Fadda
Mitchell Faddis
William Fadel
Luciano Fadiga
Mario Faenza
Camille Faes
Christel Faes
Luca Faes
Pierre Fafournoux
Jeffrey Fagan
Susan C. Fagan
Susan Fagan
Adeniyi Fagbamigbe
Marisa Faggini
Shiva Faghih
Faraz Faghri
Lorenzo Fagotti
Francois Fagotto
Nelson Fagundes
Christina Faherty
Muhammad Fahim
Mia Fahlen
Richard Fahlman
Umi Fahmida
Ahmed S. Fahmy
Aly Fahmy
Dalia Fahmy
Sebastian Fähndrich
Nicole Fahner
René Fahr
Susan Fahrbach
Christoph Fahrni
Chris Fahs
Ahmed Faik
Michel Failing
Mark Failla
Marco Faimali
Sean Fain
Abraham Fainsod
Cynthia Fair
Trudee Fair
Kirsty A. Fairbairn
Nadia Fairbairn
Jeremy Fairbank
Carolyn Fairbanks
Kimberly Fairchild
Merle Fairhurst
Lee Fairlie
Kathleen Fairman
Kate Fairweather-Schmidt
Muhammad Faisal
Edward Faison
Raphael Faiss
Andreas Faissner
Francesco Faita
Jamila Faivre
Md. Faiyazuddin
Fadi Fakhouri
Micol Falabella
Peter Falck
Nicola Falco
Luisa Falcon
Alberto Falcón
Andrew Falconar
Francisco Falcone
Marco Falcone
Marika Falcone
R. Falconer
April Falconi
Juan Falcon-Perez
Martin Faldyna
Emily Falk
Jamie Falk
Johannes Falk
Julien Falk
Markus Falk
Ronald Falk
Laura Falkenberg
Gerhard Falkenhorst
Torbjörn Falkmer
Michael Falkowski
Saeideh Fallah Fini

Hossein Fallahi
Monica Fallon
Lisandro Falomir Lockhart
Stefano Falone
Benedetto Falsini
Paul Falso
Gloria Falsone
Elisabet Fält
Elizabeth Falwell
Barbara Fam
Nicola Fameli
Mary Familari
Itziar Familiar
Michael Famulare
Bao Jian Fan
Chenguang Fan
Chuanzhu Fan
Daidi Fan
Fan Fan
Guichao Fan
Guoqiang Fan
Haojun Fan
Hongchao Fan
Hongjie Fan
Hongkuan Fan
Hsien-Yu Fan
Hui Wen Fan
Huimin Fan
Jean Fan
Jianglin Fan
Jibiao Fan
Jie Fan
Jingfang Fan
Jun Fan
Junfeng Fan
Longling Fan
Peilei Fan
Pengxian Fan
Qin Fan
Qing-Hai Fan
Ruzong Fan
Stanley Fan
Vincent Fan
Weiguo Fan
Wenyi Fan
Xianqun Fan
Xiaoduo Fan
Xiao-Hui Fan
Xing-Jun Fan
Yong Fan
Youran Fan
Yun Fan
Zhaofei Fan
Zhi Fan
Zhi-Jin Fan
Zhiqiang Fan
Zhun Fan
Bronwyn Fancourt
Joachim Fandrey
Camilla Fanelli
Vito Fanelli
Angela Fang
Carolyn Fang
Di-An Fang
Fude Fang
He Fang
Hezhi Fang
Hongbin Fang
Hua-Chang Fang
Kai Fang
Leyuan Fang
Li Tai Fang
Lin Fang
Liqun Fang
Liwei Fang
Pu Fang
Qiang Fang
Qianqian Fang
Qiaojun Fang
Shao-You Fang
Shubo Fang
Shuhuan Fang
Te-Chao Fang
Weijia Fang
Xing Fang
Xiuqi Fang
Ya Fang
Ye Fang

Yuan Fang
Zaojun Fang
Zhixiang Fang
Zhuo Fang
Nann A. Fangue
Christopher Fang-Yen
Meng Fanli
Philip Fanning
Manoussa Fanny
Katherine Fantauzzo
Federico Fanti
Juliana Fantinelli
Bernardino Fantini
Maria Pia Fantini
Wendy Fantl
Giamila Fantuzzi
Alessandro Fanzani
Jessica Fanzo
Franco Faoro Faoro
Philip Farabaugh
Mary Farach-Carson
Fernando Farache
Nauder Faraday
Mohamed Farag
S. Farahani
David Faraoni
Iman Farasat
Silvia Faravelli
Melissa Farb
Masood Fard
Olivier Fardel
David Fardon
Jasmine Fardouly
Dominic Fareri
Souha Fares
Wassim Fares
Silvia Fargion
Hesso Farhan
Walid Farhat
Nuno Faria
Ronaldo Faria
Weslem Faria
Miguel Faria-Ribeiro
Leonardo Farias
Michael Farias
Miguel Farias
Jorge Farías
María Farías
Huma Farid
Almo Farina
Cinthia Farina
Nicholas Farina
Walter Farina
Damien Farine
Giovanni Farinella
Daniel Farinotti
Reza Farivar
Attila Farkas
Michael Farkas
Andrew Farke
Jessica Farley
Tim Farley
Martin Farlow
Bhupinder Farmaha
Theodora Farmaki
Doyne Farmer
George Farmer
Harry Farmer
Ruth Farmer
Richard Farndale
Greg Farnell
Peggy Farnham
Jose Faro
Omid Farokhzad
Matthew Faron
Anthony Farone
Lêda Rita Faroni
Muhammad Farooq
Ammad Farooqi
Omaral Farooque
Habib Farooqui
Ahmed Farouk
Samira Farouk
Stephaie Farquhar
Sherry Farr
Tracy Farr
Katayoun Farrahi
Tom Farrar

Dorothy Farrar-Edwards
Albert Farre
Núria Farré
Aidan Farrell
Brenda Farrell
Michelle Farrell
Paul J. Farrell
Ken Farrington
Susan Farrington
A. Farris
Alton Farris
Dominic Farris
Ryan Farris
Shannon Farris
Andrew Farrow
David Farrow
John Farrow
Brooke Farrugia
Cher Farrugia
Nicolas Farrugia
Konstantinos Farsalinos
Juliane Farthmann
Jean Farup
Mortazavi Farzad
Mohammad Hosein Farzaei
Alessio Fasano
Stefania Fasano
Olufunke Fasawe
Peter Fasching
Folorunso Fasina
Yewande Fasina
Marianna Fasoli
Matteo Fassan
Hannah Fassel
Secondo Fassino
Mark Fast
Rosamaria Fastuca
Oyebola Fasugba
Fadi Fathallah
Hassan Fathallah-Shaykh
Marzieh Fathi
Sumana Fathima
Yaghoub Fathipour
Charles Fathman
Razia Fatima
Maria Fátima Lucas
Jaume Fatjo
Mar Fatjo-Vilas
Timur Fatkhudinov
Gerardo Fatone
George Fatseas
Quek Fatt
Julien Fattebert
Lucrezia Fattobene
Patrizia Fattori
Mirek Fatyga
Celine Faubladier
Jérémy Fauconnier
Mark Faul
Alex Faulkner
Melinda Faulkner
Sarah Faulwetter
Victor Faundez
Régis Faure
Bart Fauser
Sascha Fauser
Caitlin Fausey
Michel Fausther
Massimiliano Faustino
Pedro Faustmann
François Fauteux
Maarten Fauvart
Giammarco Fava
Emmanuel Favaloro
Graziella Favarato
Livio Favaro
Marcelo Faveri
David Favero
Sergio Favero
Isabella Favia
Oleg Favorov
Geraldine Favrais
Adrien Favre
Guillaume Favre
Colin Favret
Angela Fawcett
Christine Fawcett
Ashraf Fawzy

Manal Fawzy
David Fay
Nicolas Fay
Zahi Fayad
John Fayyad
Kiavash Fayyaz
János Fazakas
Christian Fazekas
Xavier Fazio
Marco Fazzari
Mark Fear
Nicola Fear
Howard Fearnhead
Rachel Fearns
Ian Fearon
Jeffrey Fearon
Catherine Feart
Nicholas Feasey
Alexandra Feast
Allen Featherstone
Gerardo Febres
Daniela Fecht
David Fedele
Katya Feder
Francesca Federico
Paolo Federico
Gero Federkeil
Peter Federolf
Roman Federov
Andrea Federspiel
Stacey Fedewa
Evelina Fedorenko
Inna Fedorenko
Victor Fedorenko
Ludwik Fedorko
Oleg Fedorov
Vadim V. Fedorov
David Fedson
Dennis Feehan
Brian T. Feeley
Frank Feeley
Ann Feeney
Eoin Feeney
William Feeney
W. Gregory Feero
Gregory Fegan
Corey R. Fehnel
Anthony Fehr
Jill Fehrenbacher
Boris Fehse
Jingyi Fei
Juntao Fei
Kezhen Fei
Shumin Fei
Zheng-Hua Fei
James Feigenbaum
Maria Feijoo
Mark Feinberg
Michele Feinberg
Wiebke Feindt
Kenneth Feingold
Jeffrey Feinstein
Matthew Feinstein
Zachary Feinstein
Ana Carla Feio Dos Santos
Zhu Feipeng
Adam Feist
Richard Feist
Rodrigo Feitosa
Guillem Feixas
Gedefaw Fekadu
Donna Fekete
Barbara Felber
Gordon Feld
Paul Feldblum
Vincent Felde
Guido Felder
Jennifer Felder
Marie-Paule Felder-Schmittbuhl
Heike Feldhaar
Christoph Feldhaus
Brian Feldman
Inna Feldman
Justin Feldman
Mario Feldman
Steven Feldman
Yuri Feldman
Stephan Feldmeier

Barbara Feldmeyer
Joram Feldon
David Feliciano
Onelkis Feliciano
Isidoro Feliciello
Antonio Felicioli
Denis Feliers
Martha Felini
A. Felipe-Silva
Thomas Felis
Georg Felix
Juan Felix
Luiz Felix
John Felkner
James Fell
Vanda Elisa Andres Felli
Tommaso Fellin
Martin Fellner
Deborah Fels
Joseph Felsenstein
Pedro Femia Marzo
Tom Fenchel
Anja Feneberg
Andrew Fenelon
Bang Feng
Bin Feng
Chao Feng
Chaolu Feng
Chen Feng
Congjing Feng
Gu Feng
Hailan Feng
Hai-Zhong Feng
Hongqiang Feng
Hualong Feng
Jiannan Feng
Jianwen Feng
Jia-Yih Feng
Jie Feng
Jun Feng
Kelly (Yuchen) Feng
Lei Feng
Lin Feng
Ling Feng
Qiushi Feng
Rao Feng
Shi-Ting Feng
Su-Wen Feng
Tingyong Feng
Wen-Hai Feng
Xiao Feng
Xin Feng
Xueyang Feng
Yan Feng
Yaoyu Feng
Ye Feng
Yibin Feng
Yuanjing Feng
Yuanming Feng
Zhongkai Feng
Zongdi Feng
Concettina Fenga
Joelle Fenger
William Fenical
Bridget Fenn
Fiona Fennessy
Albert J. Fenoy
Andre Fenton
Tanis Fenton
Abbey Fenwick
Alan Fenwick
Craig Fenwick
Francesco Feo
Eric Feraille
Maryam Ferdousi
Mahboobeh Fereidan-Esfahani
Sasan Fereidounit
David A. Ferenbach
Peter Ferenci
Thomas Ferenci
Gyorgy Ferenczy
Adam Ferguson
Christopher Ferguson
Deborah Ferguson
Eilidh Ferguson
Elaine Ferguson
Hugh Ferguson
Kristi Ferguson

Sarah Ferguson
Stephen Ferguson
Malcolm Ferguson-Smith
Onur Ferhanoğlu
Bart Ferket
Russell Ferland
Edoardo Ferlazzo
Gustavo Fermin
Pierre Olivier Fernagut
A. Fernandes
Alexandra R. Fernandes
Ana Paula Fernandes
Fátima Fernandes
Maria José Fernandes
Maria Luiza Fernandes
Michelle Fernandes
Natalia Fernandes
Olavo Fernandes
Paula Fernandes
Pedro Fernandes
Ricardo Fernandes
Rômulo Fernandes
Rosa Fernandes
Sabrina Fernandes
Sofia Fernandes
Timothy Fernandes
Adria L. Fernandez
Agustin Fernandez
Carmen Fernandez
Cristina Fernandez
Emilio Fernandez
Ephrem Fernandez
Gerardo Fernandez
Ivan Fernandez
Luis Fernandez
Matthew Fernandez
Miguel Fernandez
Ricardo Fernandez
Esteve Fernández
Helena Fernández
Jose Fernández
Liliana Fernández
Selene Fernández
Octavio Fernandez Limia
Maria S. Fernandez-Alfonso
Monica Fernandez-Aparicio
Daniel Fernandez-Ayala
Ana Fernandez-Bustamante
M. Milagro Fernández-Carrobles
Marcos Edgar Fernandez-Cuadros
Sandra Fernández-Fernández
Elena Fernandez-Fueyo
Marta Fernández-García
Anabel Fernandez-Iglesias
Francisco Fernández-Klett
Fernando Fernandez-Llimos
Arsenio Fernández-López
Javier Fernández-López de Pablo
Carlos Fernandez-Lozano
Mario Fernández-Mazuecos
Elisa Fernández-Millán
D. Fernández-Nóvoa
Lindsay Fernandez-Rhodes
Javier Fernández-Ruiz
Mario Fernández-Ruiz
Manuel Fernández-Sánchez
Ana Fernandez-Sesma
Enrique Fernández-Taboada
Gloria Fernández-Tilapa
Rodrigo Fernandez-Valdivia
José Ignacio Fernández-Vigo
Nilesh Fernando
Bo Fernhall
David Fernig
Gordon Ferns
Paul Fernyhough
Jack Ferracane
Chiara Ferracini
Paula Ferrada
Carla Ferragut
Diodato Ferraioli
Giovanna Ferraioli
Aude Ferran
Jose Ferran
Ida Ferrandino
Irene Ferrando
Pasquale Ferrante
Aloysio Ferrao-Filho

Carlo Ferrarese
Cleber Ferraresi
Mariano Ferraresso
Chiara Ferrari
Fiorenza Ferrari
Guido Ferrari
Paola Ferrari
Renata Ferrari
Sergio Ferrari
Marina Ferrarini
Carrie Ferrario
Manuela Ferrario
Valerio Ferrario
Angelo Ferraro
Donatella Ferraro
Giuseppe Ferrauto
Carolina Ferraz
Guilherme Ferraz
Fulvia Ferrazzi
Paolo Ferrazzi
Adilson Kleber Ferreira
Arthur Ferreira
Carmen Ferreira
Cristine Ferreira
Daniela Ferreira
Fabio Luiz Ferreira
Fernando Ferreira
Isabel Ferreira
Jacielle Ferreira
Joao Ferreira
Jorge Ferreira
Jose Carlos Ferreira
Laerte Ferreira
Leonardo Ferreira
Marcelo Ferreira
Paula Ferreira
Rafael Ferreira
Victor Ferreira
Cristiana Ferreira Alves de Brito
Ilce Ferreira da Silva
Silvio Ferreira Jr.
M. Alexandra Ferreira-Valente
Alejandro Ferreiro
Mercedes Ferrer
Miguel Ferrer
Rebecca Ferrer
Jose Ferreras
Carles Ferrer-Boix
Jose Ferrer-Paris
Fabrizio Ferretti
Francesco Ferretti
Luca Ferretti
Nicola Ferri
Gary Ferrier
John Ferrier
Silvano Ferrini
Martin Ferris
Steven Ferris
William Ferris
Charles Ferro
Manuel Ferro
Matteo Ferro
Ilario Ferrocino
John Ferron
Frank Ferrone
John Ferry
Colin J. Ferster
Andrzej Fertala
Fernando Fervenza
Howard Fescemyer
Michael Fessler
Sabrina Festa
Mario Philip Festin
Lawrence Feth
Sergueï Fetissov
Chris Fettig
Stefan Feuerriegel
Giora Feuerstein
Seth Feuerstein
Jean Feuillard
Marc Feuilloley
Paul Feustel
Rachel Fewster
Laura Feyrer
Enrico Fiaccadori
Michael Fialkow
Alison Fiander
D. B. Ficco

Marta Fichna
Yann Fichou
Harlan Fichtenholtz
Walter Fick
Fernanda Fidalgo
Rivka Fidel
Paul Fidel Jr.
David Fidock
Mark Fiecas
Adam Fiedler
Konrad Fiedler
Paul Fiedler
Pawel Fiedor
Jess Fiedorowicz
Jessica Fiege
Jennifer Fiegel
Jens Fiehler
Andrew Field
David Field
Gregory Field
John Field
Kenneth Field
Loren Field
Mark Field
Vanessa Field
Alistair Fielding
Eric Fielding
James Fielding
Richard Fielding
Aaron Fields
Ryan Fields
Paola Fierabracci
Casey Fiesler
Rainer Fietkau
Laurence Fiette
Daniela Fietz
Ingo Fietze
Bruno Figadare
Anthony Figajji
William Figg
Chase R. Figley
Tomasz Figlus
Michela Figorilli
Amanda Figueiredo
Daniela Figueiredo
Luisa Figueiredo
Patricia Figueiredo
Mariana Figueiro
Albert Figueras
Maria-Jose Figueras
Alvaro Figueroa
Carmen Figueroa
Olga Filatova
Marilène Filbet
Elaine Fileman
János Filep
Andrew Filer
Marxa L. Filgueiredo
Edivaldo Filho
Melanie Filiatrault
Francesco Filiciotto
Viachaslau Filimonau
Kristian Filion
Vassilis Filiopoulos
Szymon Filip
Luca Filipas
Gabrielle Filip-Crawford
Anna Filipek
Nikolay Filipov
Joanna Filipowska
Panagis Filippakopoulos
Massimo Filippi
José Filippini Alba
Alan Filipski
George Filis
Gerda Fillenbaum
Guido Filler
Tobin Filleter
Myriam Fillion
Sophie Fillon
Hannah Filmer
Jessica Filosa
Olivier Finance
Phil Finch
Christine Finck
Jonathan Finder
Oliver Findl
Michael Fine

Noah Fine
Paul Fine
Thomas Fine
Milton Finegold
Ellen Fineout-Overholt
John Finer
Neil Finer
Nick Finer
Janet Finer-Moore
Vittorio Fineschi
Robert Finger
Abe Fingerhut
Ralph Fingerhut
Barbara Fingleton
Jean-Baptiste Fini
Mehdi Fini
Viviana Finistrella
David Fink
Ericka Fink
Jim Fink
Rok Fink
Laruen Finka
Elena Finkbeiner
Stefan Finke
Deborah Finkel
Omri Finkel
Richard Finkel
David Finkelhor
David Finkelstein
Fredric Finkelstein
Myra Finkelstein
Barbara Finlay
Malcolm Finlay
A. Finlayson
Kathleen Finlayson
Benjamin Finley
Stacey Finley
Russell Finley Jr.
Brian Finlin
Aloke Finn
Amy S. Finn
Deborah Finn
Nigel Finn
Daniel Finnegan
Rick Finnell
Silvia Finnemann
John Finney
Christine Finnie
Ryan Finnigan
Mikko Finnilä
Sarah Finocchario Kessler
Francesca Finotello
Susetta Finotto
Walter Finsinger
Josef Finsterer
Serena Fiocchi
Alexandra Fiocco
Xavier Fioramonti
Enrico Fiore
Kathrn Fiorella
Logan Fiorella
Francesca Fiorentino
Gabriella Fiorentino
Niccolo Fiorentino
Teresa Vanessa Fiorentino
Maria Fiorenza
Valentina Fiorilli
Lisa Fiorini
Romina Fiorotto
Anne-Sophie Fiorucci
Sebastien Fiorucci
Francesca Fiory
Mehmet Firat
Michael Firbank
Bonnie Firestein
Lauren (Arnesen) Firestein
Chaz Firestone
Rebecca Firestone
Horacio Firmino
Arnaud Firon
Denis Fischbacher-Smith
A. Fischer
Andreas Fischer
Corinne Fischer
Dania Fischer
Egil Fischer
Florian Fischer
Gabriela Fischer

Heinz Fischer
Jan Fischer
Janet Fischer
Katja Fischer
Krista Fischer
Lutz Fischer
Naomi Fischer
Peter Fischer
Reinhard Fischer
Roman Fischer
Sebastian Fischer
Silvia Fischer
Susanne Fischer
Ulrike Fischer
Valentin Fischer
William Fischer
Wolfgang Fischer
Arne Fischmann
Rodolphe Fischmeister
Cene Fišer
John Fiset
Alexandra Fish
Kenneth Fish
Andrew Fisher
B. A. Fisher
Christopher Fisher
Colleen Fisher
Daniel Fisher
David Fisher
Derek Fisher
Edwin Fisher
Jason Fisher
John Fisher
Jonathan Fisher
Lee Fisher
Lynn Fisher
Rebecca Fisher
Rory Fisher
Steven Fisher
Ayelet Fishman
Ezra Fishman
Jay Fishman
Mayer Fishman
John Fisk
Ane Fisker
David Fisman
Christoph Fisser
Giovana Fistarol
Gordon Fitch
Tecumseh Fitch
William Fitt
Stephen Fitter
Daniel Fitzgerald
Devon Fitzgerald
Felicity Fitzgerald
Gerry Fitzgerald
Jamie Fitzgerald
Jennifer Fitzgerald
Melinda Fitzgerald
Tj Fitzgerald
Clare Fitzpatrick
Courtney Fitzpatrick
Margaret Fitzpatrick
Meghan Fitzpatrick
Merry Fitzpatrick
Tracy Fitzsimmons
Hans Flaatten
Alicia Flach
Andreas Flache
Daniel Flaherty
Elizabeth Flaherty
Alan Flake
Claude Flamand
Véronique Flamand
Frederic Flamant
Nicolas Flament
Constance Flanagan
Virginia Flanagin
Corey Flanders
Philippe Flandre
Timothy Flanigan
Brendan Flannery
John Flannery
Jason Flannick
Klas Flardh
Kjersti Flatmark
Andrew Flavell
David Flavell

Valerie Flax
Abraham Flaxman
Brian R. Flay
Sandra Flechas
James Fleckenstein
James M. Fleckenstein
Paul Flecknell
Joan Fledelius
John Fleeger
Jaroslav Flegr
Ilya Fleidervish
Shelby Fleischer
Angela Fleischman
Albert Fleischmann
Andreas Fleischmann
Bobbi Fleiss
Aaron Fleming
Braden Fleming
Evan Fleming
Ingrid Fleming
Jason Fleming
Jodie Fleming
Padhraig Fleming
Ronan Fleming
Sherry Fleming
Stephen B. Fleming
Thomas Fleming
Timothy Fleming
Simon Fleminger
Kelly D. Flemming
Vicki Flenady
Michelle Flenniken
Alison Fletcher
Hansel Fletcher
Helen Fletcher
Jacqueline Fletcher
Patrick Fletcher
Reginald Fletcher
Stephanie Fletcher
Laurence Flevaud
Arthur Flexer
Leon Flicker
Ulrike Flierl
Mark Flint
Paul Flint
Steve Flint
Tina Flisar
Krzysztof Flisikowski
Rebecca Flitcroft
J. Floege
Ulrich Flögel
Nicolas Floquet
Alexander Flor
Ewa Florek
F. B. Vincent Florens
Alan Flores
Andrew Flores
Carlos Flores
Francisco Flores
Gilberto Flores
Joel Flores
Laura Flores
Micah Flores
Oscar Flores
Samuel Flores
Fernando Ivan Flores-Perez
Lluvia Flores-Renteria
Mario Floria
Luis Mario Floría
João Batista Florindo
Tullio Florio
Esther Florsheim
Jean-Francois Flot
Christian Flotho
Terry Flotte
Darren Flower
Paul Flowers
Kory Floyd
Martin Flück
James Fluckey
Gerard G. Fluet
Simon Flury
Nina Fluschnik
Lena Flyckt
Brian Flynn
Cormac Flynn
Harry W. Flynn
Jamie Flynn

Jennifer Flynn
Kevin Flynn
Matt Flynn
Matthew Flynn
Suzanne Flynn
Katherine Flynn-O'Brien
Tamara Flys
Michael Flythe
Laura Fochtmann
Niels Focke
Andreas Focks
Stelios Fodelianakis
Rob Foels
John Foerster
Paolo Fogagnolo
Michael Fogarty
Quentin Fogg
Bruno Foggi
Laura Fogg-Rogers
Anne Fogli
Marta Fogolari
Donovan Fogt
Mauro Fois
A. Foisy
Tito Fojo
Pelagia Foka
Eric Fokam
Joseph Fokam
Emmanouil Fokas
Kunuwo Fokong
Jorge Folch-Mallol
Alessandra Folci
Alejandra Folco
Gianluca Folesani
Brian Foley
Geraldine Foley
Melissa Foley
Nicole Foley
William Foley
Joao Folgado
Annelise Folie
James Folk
Jonathan Folland
David Followill
Gunnar Folprecht
Brian Folt
Bashir Fomda
Ben Fong
Kenneth Fong
Lau Seng Fong
Mun Yik Fong
Noah Fongwen
Virginia Fonner
Erich T. Fonoff
Carme Fons
Bruna Fonseca
Isabel Fonseca
Kevin Fonseca
Mark Fonseca
Márlon de Freitas Fonseca
Paula Fonseca
Eduardo Fonseca-Pedrero
Jean Fontaine
Magali Fontaine
Pirmin Fontaine
Adriano Fontana
Gianluca Fontana
Marianna Fontana
Roberto Fontana
Luca Fontanesi
Diego Fontaneto
Gabriella Fontanini
Francesc Font-Clos
Michaela Fontenay
Alain Fonteneau
Carlos Frederico Fontes
Cor Jesus Fontes
Lisa Fontes
Natacha Fontes
António Paulo Fontoura
Beatriz Fontoura
Jaime Foong
Rachel Foong
Dustin Foote
Lee Foote
Simon Foote
Robert Foottit
Chad Foradori

Ronja Foraita
Christy Foran
Petter Forander
Ni Forbang
Andrew Forbes
Larry Forbes
Paul Forbes
Adam Ford
Adam T. Ford
Amanda Ford
Antonia Ford
Catherine Ford
Elizabeth Ford
Eric C. Ford
H. Ford
James Ford
Jane Ford
Julian Ford
Laura Ford
Matthew Ford
Michael Ford
Nathan Ford
Nancy Forde
Diego Forero
Jean-Claude Forest
Cristian Forestan
Catherine Forestell
Gabor Forgacs
Fabricio Forgerini
Patrice Forget
Alison Forhead
Francesca Forini
Marie-Amélie Forin-Wiart
Megan Fork
Gerald Forkuor
Ester L. Forlales
Gregory Forlenza
Maria Forlenza
Harry Forman
Fabio Formenti
Luigi Formisano
Pietro Formisano
Michele Fornaciai
Christen Fornadel
Lorenzo Fornaro
Michele Fornaro
Astrid Fornek
Anna Fornell
Joachim Forner
Miguel Fornés
Arianna Fornili
Erick Forno
Alessia Fornoni
Cyrus Foroughi
Vivienne Foroughirad
Christopher Forrest
Douglas Forrest
J. Forrest
John Forrester
Lesley Forrester
L. Forsberg
Karin Forsberg-Nilsson
Joana Forsea
Allan Forsman
Christopher Forsmark
Maria Forsner
Julia Forstenpointner
Rodney Forster
Elisabeth Förster-Waldl
Asta Försti
Wolfgang Forstmeier
Jitka Forstova
K. Forsyth
Laura Forsythe
Steven Forsythe
Jérôme Fort
Patrice Fort
Giovanni Forte
Consuelo Fortes-Dias
Fabio Forti
Tomaso Fortibuoni
Anne-Marie Fortier
Lisa Fortier
Elise Fortin
Jennifer Fortin
Renee Fortner
Elizabeth Fortunato
Talita Fortunato-Tavares

Claire Fortunel
Jarrod Fortwendel
Maria Forzan
Marie Fosbol
Claudio Foschi
Federico Foschi
Maria Pia Foschini
Andrew Foskett
Carlos Fossati
Claudio Fossati
Silvia Fossati
Becky Foster
Byron Foster
Carl Foster
Derek Foster
Diana Foster
Emma Foster
James Foster
Jane Foster
Jeffrey Foster
Joshua Foster
Michael Foster
Monique Foster
Rick Foster
Simon Foster
Tina Foster
Anne Fostier
Daniela Patrizia Foti
D. Fotiadis
Jerome Foucaud
Eva Fouka
Ali Fouladi-Nashta
Mitra Fouladirad
Nicholas Fountain-Jones
Elise Fouquerel
Yoan Fourcade
François Fourchet
Alexandre Fouré
Pierre Fouret
Maralyn Foureur
Hendrika Fourie
Claudia Fournier
Elisabeth Fournier
Pierre-Edouard Fournier
Georgia Fousteri
Ashley Fowler
Cynthia Fowler
Patrick Fowler
Veronica Fowler
Barry Fox
Brian Fox
David Fox
Donald Fox
Erin Fox
Glen Fox
Gregory Fox
James Fox
Karin Fox
Keith Fox
M. Fox
Molly Fox
Neil Fox
Samuel Fox
Spencer Fox
Stanley Fox
Stephen Fox
Christine Foyer
Elizabeth Fozo
Claudio Fozza
Marco Fraaije
Magdalena Frąc
Anna Ludovica Fracanzani
Alessandra Fracasso
Daniela Fraccarollo
Amanda Frade
Karl Frafjord
Deborah Fraga
Helder Fraga
Isabel Fraga
Luis Gerardo de la Fraga
Gabriele Fragasso
Tiziana Fragasso
Dorothy Fragaszy
Filippo Fraggetta
Sotirios Fragkostefanakis
Enric Frago
José Fragoso
Rodrigo Rocha Fragoso

Daniel Fraher
Gail Fraizer
Michael Fraker
Garrett Frampton
Piotr Franaszczuk
Marcondes França Jr.
Paolo Francalacci
Rubén Francés
Silvana Franceschetti
Francesco Franceschi
Silvia Franceschi
Guido Franceschini
Nora Franceschini
Sandro Franceschini
Di Gennaro Francesco
Giallauria Francesco
Mirko Francesconi
Francesco Franchi
Fulvio Franchi
Martino V. Franchi
Martina Franchini
Gianluigi Franci
Emerson Francini
M. Pilar Francino
Diego Franciotta
Brian Francis
Charles Francis
Chris Francis
Diane Francis
Gordon Francis
Gregory Francis
Isolde Francis
Jean Francis
Merlin Franco Francis
Michael Francis
Nicholas Francis
Peter Francis
Richard Francis
Stephen Francis
Romeu Francisco
Sara Francisco
Francisco Francisco Abad-Santos
Robert Franciscus
Pierre Franck
Daniel Franco
Jorge Franco
Nuno Henrique Franco
Ricardo Franco
Rose Franco
Valentina Franco
Heitor Franco de Andrade
Helene Francois
Jean Francois
Patrice Francois
Sylvie Francois
Sandrine François-Souquière
Liliana Franco-Lara
Renata Françoso
Alison Frand
Rafael Frandoloso
Ellen Frandsen Lau
Arthur Frank
Dara Frank
Eibe Frank
Joachim Frank
Kevin Frank
Kristyn Frank
Linda Frank
Matthew Frank
Rachel Frank
Leonora Franke
Paul Frankel
Nicolaas Franken
Philipp Franken
Julius Frankenbach
Benjamin Frankfort
Greg Frankfurter
W. Anthony Frankino
Anna Franklin
Bryony Franklin
Erik Franklin
Gary Franklin
Jeremy Franklin
Patricia Franklin
Robert G. Franklin
Jonathan Franklyne
Kevin Frankowski
Charles Fransen

Joseph Franses
Thomas Franssen
Maria Frantzi
Alfred Franz
Marion Franz
Nico Franz
Sandra Franz
Scott Franzblau
Silvia Franzellitti
Daniel Franzen
Giovanni Franzo
Lori Frappier
Angelisa Frasca
Manfred Frasch
Martin Frasch
Simonetta Fraschetti
Matteo Fraschini
B. Fraser
David Fraser
Gilles Fraser
James Fraser
Jennifer Fraser
Sarah Fraser
Steve Fraser
Stuart Fraser
Nicole Fraser-Hurt
Tim Frasier
Elisa Frasnelli
Francesca Frassinetti
Roberta Frass-Kriegl
Renato Prata de Moraes Frasson
Piero Fraternali
Sara Fratini
Lucilla Frattura
Philippe Fraunie
Michael Fray
David Frayer
Katherine Frayman
Bodvaël Fraysse
Francois Fraysse
David Frazer
Maurizio Fraziano
Emilie Frealle
Erik Fredenberg
Gaboyer Frederic
Kendra Frederick
Peter Frederikse
Anja Frederiksen
Henrik Frederiksen
Yoon Frederiksen
Britta Frederiksen-Møller
Gabrielle Fredman
David Fredricks
Per Morten Fredriksen
Maria Fredriksson-Ahomaa
Caroline Free
Todd Freeberg
Michael Freedberg
Benjamin Freedman
Mark Freedman
Morris Freedman
Sara Freedman
Seth Freedman
Kelle Freel
Benjamin Freeman
Brian Freeman
Jennifer Freeman
Professor Freeman
Steven Freeman
Sylvie Freeman
Theresa Freeman
William Freeman
Maxine Freeman-Robinson
Michael Freemark
Jeremy Freese
Dean Freestone
Megan Freeth
Rosa Fregel
José Fregnani
Britta Frei
Ursula Frei
Brian Freibaum
Ellen Freiberger
Tom Freiberger
Esteban Freidin
Navid Freidoonimehr
Caroline Freiermuth
Richard Freifelder

Diana Freitag
Nancy Freitag
Cintia Freitas
Sandro Freitas
Carolyn Freiwald
Thibaut Fréjaville
Lee Frelich
Marie-Laure Frelut
Véronique Frémeaux-Bacchi
Stephen Fremes
Brent French
Katherine E. French
Martyn French
Varghese Frency
Matthias Frentzen
Harald Frenz
Inéz Frerichs
Leah Frerichs
David Fresco
Paula Fresco
Libera Fresiello
Jonathan Fresnedo-Ramírez
Shane Fresnoza
Catherine Freudenreich
John Freudenstein
Conrad Freuling
Jean-Noël Freund
Patrick Freund
Paul A. Frewen
Andreas Frey
Georgia Frey
Mark Frey
Sandra Frey
Ana Friães
Vinicius Frias de Carvalho
Jorge Frias-Lopez
Anders Friberg
Hanna Friberg
Oddgeir Friborg
Sebastien Fribourg
Radek Frič
Ulrich Frick
Florian Fricke
Lloyd Fricker
Tyler Fricker
Hagen Frickmann
Leonid Fridlyand
Gene Fridman
Bernd Friebe
Hermann Frieboes
Eiko Fried
Maud Frieden
Christine Friedenreich
Alan Friedlander
Marc Friedlander
Alan Friedman
Alexander Friedman
Antony Friedman
Dan Friedman
Deborah Friedman
Eitan Friedman
Jacob Friedman
Jennifer Friedman
Joseph Friedman
David Friedmann
Anne Friedrich
Markus Friedrich
Nele Friedrich
Thomas Friedrich
David Friel
Robert Friesel
Amanda Friesen
Maren Friesen
Megan Friesen
Melissa Friesen
Helmut Friess
Jan Friess
Antonio Frigeri
Lorenzo Frigerio
Nicolas Friggens
Johan Frijns
Andrea Frilling
Andreas Frings
Lars Frings
Stephan Frings
Gaelle Friocourt
Christophe Frippiat
Jean-Pol Frippiat

Matthias Frisch
Karl Friston
Jens Frisvad
David Frisvold
Xavier Fritel
Jessica Frith
Lucy Frith
Susannah Fritton
Sarah Fritts
Uwe Fritz
Marvin Fritzler
Ray Frizzell
Filippo Frizzi
Kerstin Fröber
Jörg Fröbisch
Celine Frochot
Halley Froehlich
Tanya Froehlich
Michael Froehner
Christian Froekjaer Jensen
Robert Froemke
Helmut Frohnhofen
Pascal Froissart
Claudia Frola
Andreas Frölich
Steve Frolking
Roman Frolov
Arvid Fromberg
Pascal Froment
Larry Fromm
Jörg Frommer
Ingo Frommholz
Karl-Heinz Frommolt
Edward Frongillo
Ines Fronteira
Walter Frontera
Juhana Frosen
Laurent Frossard
Chris Frost
David Frost
Jeffrey Frost
Karl Frost
Ram Frost
Steven Frost
Johan Frostegård
Marine Frouin
Jan Frouz
Guy Froyen
Josef Fruehwald
Giovanna Frugis
Gema Frühbeck
Sascha Früholz
Rebecca Frum
Davide Frumento
Mario Fruzangohar
Donald Fry
Jessica Fry
William Fry
Judith Frydman
Richard Frye
Victoria Frye
Jonathan Fryer
Louise Fryer
Tim Fryer
Daniel Frynta
Bai-Ping Fu
Bowen Fu
Chao Fu
Chi-Yu Fu
Chunyun Fu
Da Fu
Dehao Fu
Geng-Feng Fu
Guo Fu
Haiyan Fu
Hai-Yan Fu
Hongtuo Fu
Hongyun Fu
Jianliang Fu
Jihua Fu
Junjiang Fu
Junjie Fu
King-Wa Fu
Li Fu
Lianwu Fu
Liping Fu
Qi Fu
Qian-Jie Fu

Qing-Ling Fu
Qiushi Fu
Sau Nga Fu
Shu Man Fu
Shujun Fu
Siu Ngor Fu
Susie Fu
Xiang-Dong Fu
Xiaodong Fu
Xingbing Fu
Yang-Chieh Fu
Yong-Bi Fu
Yongshuo Fu
Zhen Fu
Zhengwei Fu
Todsaporn Fuangrod
Zhou Fu-Bao
Lorenzo Fuccio
Dietmar Fuchs
Joerg Fuchs
Marc Fuchs
Sandra Fuchs
Stephen Fuchs
Ernst-Martin Fuchtbauer
Karolina Fucikova
Tobias Fuehrmann
María Fuentes
Sigfredo Fuentes
Enrique Fuentes-Mattei
Isabel Fuentes-Santos
César Fuentes-Yaco
Peter Fuerst
Jordi Fuertes Manuel
Juan Fueyo
Kristina Fuhr
Jeffry Fuhrmann
Masahiko Fujihara
Yoshitaka Fujihara
Kohtaro Fujihashi
Hiroshi Fujii
Naoaki Fujii
Satoshi Fujii
Takaaki Fujii
Takao Fujii
Tomoko Fujii
Tsutomu Fujii
Noriyuki Fujima
Ko Fujimori
Takahito Fujimori
Koichi Fujimoto
Koji Fujimoto
Nobukazu Fujimoto
Yoshinori Fujimoto
Shiki Fujino
Yoshihisa Fujino
Ken Fujioka
Hiroko Fujisato
Tomoyuki Fujisawa
Yasuko Fujisawa
Hiroshi Fujita
Hisashi Fujita
Katsuhide Fujita
Kazutoshi Fujita
Koki Fujita
Masayuki Fujita
Satoshi Fujita
Toshiro Fujita
Yasuyuki Fujita
Yukihiro Fujita
Yuko Fujita
Katsuhito Fujiu
Esther Fujiwara
Ken Fujiwara
Manabi Fujiwara
Yasuhiro Fujiwara
Yoshiyuki Fujiwara
Mikihiro Fujiya
Akira Fujiyoshi
So-Ichiro Fukada
Naomi Fukagawa
Kakuhiro Fukai
Kei Fukami
Keiichi Fukaya
Masashi Fukayama
Takeo Fukuchi
Kazuhiko Fukuda
Keitaro Fukuda
Ken Fukuda

Noboru Fukuda
Sanae Fukuda
Shinichi Fukuda
Atsushi Fukui
Hiroyuki Fukui
Kiichi Fukui
Yasuyuki Fukukawa
Heidge Fukumasu
Manabu Fukumoto
Kenji Fukunaga
Ryuya Fukunaga
Hideoki Fukuoka
Akihisa Fukushima
Atsushi Fukushima
Takuya Fukushima
Masamitsu Fukuyama
Daniel Fulford
Gianluca Fulgenzi
Domenico Fulgione
Richard Fullagar
Douglas Fullen
Aaron Fuller
Barry Fuller
Douglas Fuller
Maria Fuller
Sebastian Fuller
Tony Fuller
Thomas Fuller-Rowell
Susan Fullerton-Shirey
Attila Fülöp
Tibor Fülöp
Brent Fulton
David Fulton
Erika Fulton
Elena Fumagalli
Matteo Fumagalli
Luca Fumarco
Tulio Fumian
Katsu Funai
Hiromasa Funato
Tomas Funda
Hartmut Fünfgeld
Megan Fung
Sin-Yee Fung
Thomas Fung
Tung Fung
Vicki Fung
Juergen Funk
Birgit Funke
Jan Funke
Paul Funston
Suzanne Fuqua
Giulia Furfaro
Jennifer Furin
Carlo Furlan
Karina Furlan
Cesare Furlanello
John Furler
Kevin Furlong
Laura I. Furlong
Michael Furlong
Bradley Furman
Christine Fürst
Axel Fürstberger
Alexandre Fürstenberg
Tamzin Furtado
Maximilian Fürthauer
Martin Furtner
Kengo Furuichi
Tatsuya Furuichi
Luzia Furukawa
Shinya Furukawa
Yoshiaki Furukawa
Yutaka Furukawa
Fumikazu Furumi
Yuki Furuse
Shunsuke Furuta
Masahiko Furutani
Toshi Furuuchi
Fumihiko Furuya
Mitsuko Furuya
Shinichi Furuya
Tatiane Furuya
Yoichi Furuya
Luis Furuya-Kanamori
Tomoyuki Furuyashiki
Morgan Furze
Bonnie Furzer

Paolo Fusar-Poli
Nicole Fusco
Massimo Fusconi
Yasutaka Fushimi
Marco Fusi
Andrea Fuso
Johannes Fuß
Martin Fussenegger
Daniel Fuster
Jean-Michel Fustin
Mitsuru Futakuchi
Anthony Futerman
Richard Futrell
Lisieux Fuzessy
Abby Fyer
Jackson Fyfe
Dmitry Fyodorov
Joseph G.
Madhu G. M.
Tamas Gaal
Diann Gaalema
Francois Gaascht
Douglas Gaasterland
Marçal Gabalda-Sagarra
Ahmed Gaballa
Kamis Gaballah
Gillie Gabay
Belinda Gabbe
Tim J. Gabbett
Alessandro Gabbiadini
Christopher Gabel
Davide Gabellini
Thomas Gable
Nicholas Gabler
Rafael Gabriel
Robert Gabriel
Sonya Gabrielian
Charlotte Gabrielsen
Kathleen Gabrielson
Alessandra Gabutti
Giovanni Gabutti
John Gaby
Anthony Gaca
Emma Gachomo
Frédéric Gachon
Márta Gácsi
Ahmed Gad
Mohamed Gad
Bharathi Gadad
Shahinaz Gadalla
Reuma Gadassi
Emily Gadbois
Michelle Gadd
Giovanni Gadda
Sumanlatha Gaddam
Vasanthi Gaddi
Kristen Gaddis
Dana Gaddy
Jennifer Gaddy
Rikke Gade
F. Gadea
Gilles Gadea
Joaquin Gadea
Bart Gadella
Sylvie Gadeyne
Massimo Gadina
Ivana Gadjanski
Anne Gadomski
Inae Gadotti
Klaus Gadow
Jeffrey Gadsden
Jochen Gaedcke
Lowell Gaertner
Matthias Gaestel
William Gaetz
Holly Gaff
Michelle Gaffey
Patrick Gaffney
Angelo Gaffo
Phillip Gage
Yann Gager
Tomaso Gaggero
Hanna K. Gaggin
Andrea Gaggioli
Manjusha Gaglani
Michael Gaglia
Antonina Lisa Gagliano
Sarah Gagliano

Teresa Gagliano
Maria Cristina Gaglianone
Stella Gagliardi
Anna Gagliardo
Amandine Gagneux-Brunon
Dominique Gagnon
Jorge Gago
Manuel Gahete
Carl Gahmberg
Forest Gahn
Sarah Gaichas
Gabriel Gaidosh
Virginija Gaigalaite
Michael Gaihede
Nilesh Gaikwad
Swapnil Gaikwad
Ujjwala Gaikwad
Carlo A. J. M. Gaillard
Dany Gaillard
Jean-François Gaillard
María Emilia Gaillard
Robert W. Gaines
Todd Gaines
Kristin Gainey
Inna Gaisler-Salomon
Hernando Gaitán-Duarte
George Gaitanis
Luis Gaitero
Sabrina Gaito
Thomas Gaj
Karina Gajardo
Marzena Gajecka
Pawel Gajer
Byron Gajewski
Patrick Gajewski
Zdzislaw Gajewski
Krzysztof Gajowniczek
Max Gakh
Diane Gal
Katya Galactionova
Serge Galam
Csaba Galambos
Claire Galand
Maria Galang-Boquiren
Raquel Galante
Theodore Galanthay
Hashini Galappaththi-Arachchige
M. Galar-Martinez
Juan Galarza
Mark Galatowitsch
Christine Galavotti
Martyna Galazka
James Galbraith
Esther Galbrun
Fabio Galbusera
Maurizio Galderisi
Jose Carlos Galduroz
Cheryl Gale
Christopher Gale
Daniel Gale
Jerome Galea
Liisa Galea
Pascale Galea
Eugenia Galeota
Caroline Galeotti
Wolfgang Galetke
Mauro Galetti
Luciana Galetto
Giovanni Galfano
Richard Galgon
Ricardo Galhardoni
Victor Galiano
Pierre Galichon
Jonah Galicia
Piero Galieni
Andrea Galimberti
Elisa Galimberti
Alfiia Galimzianova
Cristi Galindo
Rene Galindo
L. Galindo-González
Caridad Galindo-Romero
Jorge Galindo-Villegas
Jayne Galinsky
Leonarda Galiuto
Gennaro Galizia
Andrew Gall
Christine Gall

Seana Gall
Catherine Gallagher
Colin Gallagher
Matthew Gallagher
Tom Gallagher
Daniel Gallaher
Paul Galland
Belinda Gallardo
Karine Gallardo
Rodrigo Gallardo
Jaime Gallardo Alvarado
Leticia Gallardo-Estrella
Monica Galleano
Aina Gallego
Francisco Gallego
Juan Álvaro Gallego
M. Gallego
Angel Gallego Lázaro
Danielle Gallegos
Luca Gallelli
Caleb Gallemore
Armel Gallet
Maura Galletta
Elena Galli
F. Galli
Massimo Galli
Richard Galli
G. Gallicano
Claudio Gallicchio
Emily Gallichotte
Daniel Gallie
Arianna Galliera
James Galligan
Sabina Gallina
Chiara Gallione
Randy Gallistel
Antonio Gallo
Bill Gallo
Carmen Gallo
Diego Gallo
Gianluca Gallo
James Gallo
Pedro Gallo
Valerio Gallotta
Riccardo Gallotti
Marie Galloux
Nathan Galloway
Maurizio Gallucci
Silvano Gallus
David Gally
Fabienne Gally
Anat Galor
Michael Galperin
Sophie Galson
George Galster
Adriana Galvan
Immaculada Galvan-Sanchez
Sergio Gálvez
Ana Gama
Clarissa Gama
Marcelo Gama de Abreu
Miguel Gama Sosa
Alyssa A. Gamaldo
Margarita Gamarra
Pascal Gamas
Valentina Gambardella
Orsola Gambini
Giorgio Gambino
Amandine Gamble
Stephanie Gamble
T. Gamblin
Freddy Gamboa
Jorge Gamboa
Claudia Gamboa-Gómez
Lucia Gambuzza
Daniel Gamermann
Anna Gamero
Manuel Gámez-Guadix
David Gamm
Hilary Gammill
Don Gammon
John Gamon
Sonja Gamsjaeger
Fei Gan
Gregory Gan
Guojun Gan
Han Ming Gan
Lu Gan

Samuel Ken-En Gan
Seng-Ian Gan
Shi-Rui Gan
Yong Gan
Yunn-Hwen Gan
Sabha Ganai
Shabir Ganai
Arsheed Ganaie
Andy Ganapathi
Sindhu Ganapathy
Vadivel Ganapathy
Togoobaatar Ganchimeg
Paolo Gandellini
Fabian Gander
Ravi Kumar Gandham
Chandrashekhar Gandhi
Manisha Gandhi
Mihir Gandhi
Monica Gandhi
Robin Gandley
Isabella Gandolfi
Marialuisa Gandolfi
Ilaria Gandolfini
Roghayeh Gandomkar
Emmanuel Gandouin
Kumaresan Ganesan
Subramaniam Ganesan
Timothy Ganesan
Venkat Ganesan
Suvarna Ganesh Kini
Muralikrishna Gangadharan Komala
Silvia Gangemi
Daniel Ganger
Steve Gangestad
Umesh Gangishetti
David Gangitano
Sophie Gangloff
Bhushan Gangrade
Abantika Ganguly
Auroop Ganguly
Avishek Ganguly
Koustav Ganguly
Tridib Ganguly
Rita Gangwani
Linsey Gani
Alexander Ganin
Douglas Ganini da Silva
Davood Ganji
Neil Ganju
Ian Ganley
Sreenivas Gannavaram
Maureen Gannon
Melissa Gannon
Victor Gannon
Ioannis Ganopoulos
Ziv Gan-Or
Fraide Ganotice
Hayley Gans
Andre Ganswindt
Benjamin Gantenbein
Philip Ganter
Pierre Gantner
Brett Gantt
Daniel Ganu
Vitaly Ganusov
Melanie Ganz
Michael Ganz
Peter Ganz
Tomas Ganz
Yoav Ganzach
Ron Ganzevoort
Jörg Ganzhorn
Michael Gänzle
Baoyu Gao
Bin Gao
Bo Gao
Bu-Lang Gao
Caiqiu Gao
Changshou Gao
Chunji Gao
Congfen Gao
Dawen Gao
Daxing Gao
Fan Gao
Fei Gao
Feng Gao
Guangyao Gao
Guimin Gao

Hongbo Gao
Hongjiang Gao
Huijiang Gao
Huijun Gao
Jianbo Gao
Jianwei Gao
Jiejun Gao
Jin Gao
Jingxia Gao
Jinlong Gao
Ju Gao
Junling Gao
Kunshan Gao
Lei Gao
Ling Gao
Lu Gao
Mingming Gao
MingZhong Gao
Nan Gao
Qianying Gao
Qingzhu Gao
Sheng Gao
Shengjie Gao
Shiqing Gao
Shuhong Gao
Shunji Gao
Song Gao
Tianchong Gao
Tianshun Gao
Tianxiang Gao
Ting Gao
W. Gao
Wei Gao
Wei Wei Gao
Wenhong Gao
Xiang Gao
Xiangyun Gao
Xian-Shu Gao
Xiaohui Gao
Xiaoxue Sherry Gao
Xin Gao
Xing Gao
Xinyan Gao
Xuejun Gao
Yang Gao
Ying Gao
Zheng-Nan Gao
Zhiqiang Gao
Zhong-Ke Gao
Zhong-Shan Gao
Vadim Gaponenko
Olga Garaschuk
Sophiya Garasia
Andrea Garatti
Enrico Garattini
Livia Garavelli
Jessica Garb
Rayyan Garba
Sergio Garbarino
Patrizia Garbati
Corrado Garbazza
Gary Garber
John J. Garber
Håvard Garberg
Amel Garbou
A. Garbuno-Inigo
Frank Garcea
Ana Garcés
Henri-Jean Garchon
Andre Garcia
Beate Hennie Garcia
Brandon Garcia
Danilo Garcia
David Garcia
Erin Garcia
Fernando Garcia
Flávio Garcia
Graciela Garcia
Irene Garcia
Jonathan Garcia
K. Christopher Garcia
Katherine Garcia
L. Rene Garcia
Linda Garcia
Maricarmen Garcia
Mark Garcia
Meritxell Garcia
Michael Garcia

Miriam Garcia
Montse Garcia
Oreto Garcia
Ricardo Garcia
Sandra Garcia
Sebastian Garcia
Veronica Garcia
Beatriz García
Federico García
Rosario García
José García de la Asunción
Daniel Garcia de la serrana
Jose Garcia de la Torre
Patricia García de Olalla
Julian Garcia Feijoo
Jose Garcia Fernandez
Hector Garcia Garcia
David Garcia Juan
Jorge García Molinos
Cristina Garcia Perez
Adolfo García Sainz
Axel Garcia y Garcia
Javier Garcia-Alba
Luis Garcia-Alonso
Luz Garcia-Alonso
Jaime García-Añoveros
Clemente Garcia-Avila
Victoria Garcia-Cardenas
Fabio García-Castro
Francisco García-del Portillo
Marcos García-Diez
Aurora Garcia-Dorado
Guillermo Garcia-Effron
Ramon Garcia-Escudero
David García-Fresnadillo
Guillermo Garcia-Garcia
David García-Gudiño
Claudio Garcia-Herrera
Ricardo García-Herrera
Naroa Garcia-Ibaibarriaga
Núria Garcia-Jacas
Vanessa Garcia-Larsen
Luisjoaquin Garcia-Lopez
Selene Garcia-Luna
Miguel Á. García-Martínez
Jordi Garcia-Mas
Xavier García-Massó
Carlos García-Mata
Mauricio García-Mateu
Fernando Garcia-Menendez
José María García-Mina
Vicente Garcia-Navas
Africa Garcia-Orad
Daniel García-Ovejero
Azucena Garcia-Palacios
Abel Garcia-Pino
Sara Garcia-Ptacek
María Isabel García-Real
Julian Garcia-Rejon
Gerardo García-Rivas
Noemi Garcia-Romero
Carmen Garcia-Ruiz
Pedro Garcia-Ruiz
Sonia Garcia-Salicetti
Tomas Garcia-Sanchez
F. García-Sánchez
Javier García-Sancho
Ibai Garcia-Tabar
I. F. Garcia-Tejero
Ignacio Garcia-Verdugo
Carolina Garcia-Vidal
Cristina García-Viguera
Angel Garcimartín
Christophe Garcion
Loic Garçon
Adrian Garda
Gwenn Garden
David Gardiner
Elizabeth Gardiner
James Gardiner
Katheleen Gardiner
Matthew Gardiner
Natalie Gardiner
Philip Gardiner
Stuart Gardiner
Joseph Gardinier
Andrew Gardner
Beth Gardner

Caleb Gardner
Candice Gardner
David Gardner
Dianne Gardner
James Gardner
Janet Gardner
Jillian Gardner
Kyle Gardner
Lauren Gardner
Lytt Gardner
Paul Gardner
Thomas Gardner
Jason Gardosi
Mahdi Garelnabi
Lim Gareth
Zeiler Gareth
Richard Garfein
Sara Garfield
Dana Garfin
A. Garg
Harish Garg
Himanshu Garg
Rekha Garg
Sunir Garg
Paolo Gargiulo
Daniela Garib
Valentina Garibotto
Venkata Garikipati
Eduardo Garin
Larisa Garkava-Gustavsson
Cedric Garland
Christopher Garland
Emily Garland
Patrick Garland
Theodore Garland
Taryn Garlock
Joshua Garn
Jeff Garnas
Karine Garneau
Nicole Garneau
Allen Garner
Amanda Garner
Stephen Garnett
Diana Garnica
Gil Garnier
Maeva Garnier
Carlo Garofalo
Franco Garofalo
Germana Garofalo
Anastasia Garoufi
Patxi Garra
M. Garratt
Olivier Garraud
James Garrett
Timothy Garrett
Carlos Garrido
Jose Garrido
Marcial Velasco Garrido
Pm Garrido
Rg Garriga
Patrick Garrigan
Patton Garriott
Deborah Garrity
George Garrity
Marie-Line Garron
Jose A. Garrote
Maïté M. G. Garrouste-Orgeas
Colin Garroway
David Garry
David Garshelis
Eric Garshick
Antje Garten
Anton Gartner
Coral Gartner
Marieke Gartner
James Garvey
W. Garvey
Jeffrey Garvin
Michael Garvin
Thomas Gary
Eleftherios Garyfallidis
Anthony Garza
Jennifer Garza
Manuel Garza León
Jessica Garzke
Carol Garzon Lopez
Francesco Garzotto
Janvier Gasana

Antonella Gasbarri
Simani Gaseitsiwe
Danijela Gasevic
Milica Gasic
Agata Gasiorowska
E. Gaskell
Peter Gaskill
James Gaskin
Francisco Gaspar
Héléna Gaspar
John G. Gaspar
Nicolas Gaspard
Anthony Gaspari
Flavio Gaspari
Zoltán Gáspári
Francesca Gasparini
Davi Gasparini Fernandes Cunha
Roberto Gasparri
Philippe Gasque
Natalia Gass
Susan Gasser
T. Christian Gasser
Thomas Gasser
Vanessa Gasser-Steiner
Natalie Gassman
Max Gassmann
Thomas Gast
Mikel Gastaca
Eduardo Gastal
Laura Gastaldi
Pablo Gastaminza
Paul Gastanaduy
Amandine Gastebois
Miguel Gastelurrutia
Amelia Gaston
Jsh Gaston
Xavier Gasull
Jose Gatell
Josep Maria Gatell
Deanna Gates
Gail Gates
Terry A. Gates
John Gatesy
Albert Gatt
Luigi Gatta
Valentina Gatta
Carolina Gattei
Hannes Gatterer
Jean-Luc Gatti
Stefano Gatti
Thiago Gatti Pianca
Mariana Gatto
Thomas Gattringer
Justin Gatwood
Peter Gaube
Philippe Gaubert
Susana Gaudencio
R. Gaudin
Mario Gaudino
Pierrette Gaudreau
Francis Gaudreault
Rene Gaudreault
Sylvie Gaudron
Bertrand Gauffre
Julia Gauglitz
Dominique Gauguier
James Gauld
Christopher Gaulke
Victor Gault
Bruno Gaume
Robert Gaunt
Rajeeva Gaur
Rohit Gaurav
Sunil Gautam
Vibhav Gautam
Yadu Gautam
Mathias Gautel
Benoit Gauthier
Pascale Gauthier
Sylvie Gauthier
Aude Gautier
Lara Gautier
Anne Gautier-Bouchardon
Joanna Gautney
Jacques Gautrais
Jean-Philippe Gauvin
Laetitia Gauvin
Neda Gavarić

Gaëtan Gavazzi
Maria Gaviao
Felicity Gavins
Jose A. Gavira
Elizabeth Gavis
Nirit Gavish
Andrey Gavrilov
Beata Gavurova
Indika Gawarammana
Matthias Gawlitza
Stanislaw Gawronski
Noellie Gay
Surya Gayet
Christine Gaylarde
Irina Gaynanova
Andrea Gaynor
Jeffrey Gaynor
Eve Gazave
Frédéric Gazeau
U. Gazi
Tom Gaziano
Tatjana Gazibara
Yoav Gazit
Ievgeniia Gazo
Antonio Gazol
Angelo Gazzano
Gus Gazzard
Sonia Gazzarrini
Elisabetta Gazzerro
Mattia Gazzola
Yvetta Gbelska
Francis Gbogbo
Uwe Gbureck
Joanna Gdula-Argasinska
Changzi Ge
Gaoxiang Ge
Hao Ge
Hu Ge
Lei Ge
Li Ge
Lixia Ge
Shaoyu Ge
Tian Ge
Wei Ge
Xiachun Ge
Xianhong Ge
Xin Ge
Xinmin Ge
Xue-Jun Ge
Ying Ge
Yongpeng Ge
Yubin Ge
Zhaolong Ge
Zhi-Dong Ge
Guillermo Gea-Izquierdo
Po-Wu Gean
Nicholas Geard
David Geary
Fatima Gebauer
Nicole Geberzahn
Ille Gebeshuber
Sabine Gebhardt
Daniel Gebler
Abel Gebre
Berihu Gebrekidan
Assefaw Gebremedhin
Samuel Gebremedhn
Berhanu Gebremeskel
Yonas Geda
Dawit Gedamu
Gabrielle Geddes
James Geddes
Rastafa Geddes
Odrun Gederaas
Kenan Gedik
Melaku Gedil
Naomi Geeraert
Andrew Geering
Audrey Geffen
Maria Gehne
Jennifer Geib
John Geibel
Adam Geiger
Alain Geiger
James Geiger
Jonathan Geiger
Sonja Geiger
Steven Geiger

Steffen Geis
Rolf Geisen
Fritz Geiser
Marianne Geiser
Eldon Geisert
Rodney Geisert
Edward Geisinger
Benjamin Geisler
Corinna Geisler
Matt Geisler
Tobias Geisler
William Geisler
Quentin Geissmann
Andreas Geist
Nick Gekakis
A. Gelb
William Gelbart
Shari Gelber
Stefan Gelcich
Hubrecht Gelderblom
Mathias Gelderblom
Hans Gelens
Mikhail Gelfand
Roselle Gélinas
David Gell
Marie-Paule Gelle
Kapuaola Gellert
Malleswari Gelli
Jean-Christophe Gelly
Julien Gelly
Carlo Gelmetti
Guido Gelpi
Josep Gelpí
Dmitry Geltman
Moti Yohannes Gemechu
Marlene Gemelon
Alison Gemmill
Suzuki Gen
Becky Genberg
Yilmaz Emre Gencay
Caroline Genco
Takuya Genda
Fernand-Pierre Gendron
Igor Generalov
Joseph Genereux
Elke Genersch
Blaise Genest
Pierre Genevaux
Anne-Marie Genevière
Janice Genevro
Bin Geng
Lei Geng
Qing Geng
Xin Geng
Nicolas Gengler
Zhao Gengmao
Olga Genilloud
Guy Genin
Stéphane Genin
Luciana Génio
Douglas Genna
Andrew R Gennery
Lisa Gennetian
Jean-Luc Gennisson
Helen Genova
Giuilo Genovese
Andrea Genre
John Gensel
David Gent
Fernando Genta
Dane J. Genther
Paulo Gentil
Francesco Gentile
Giorgio Gentile
Ivan Gentile
Piergiorgio Gentile
Salvatore Gentile
Rodolfo Gentili
Matthew Gentle
Kornelia Gentsch
Maurizio Genuardi
Franziska Genzel
Lisa Genzel
Steve Geoffrion
Cedric Geoffroy
Jemma Geoghegan
Katerina Georganta
Elizabeth George

Graham George
Joby George
Michael George
Nithin George
Paul George
Peter George
Arthur Georges
Karla Georges
Adriana Georgescu
Carmen Georgescu
Anastasia Georgiades
Andreas Georgiadis
Millie Georgiadis
Nicholas Georgiadis
Panagiotis Georgianos
Tihomi Georgiev
Harry Georgiou
Panayiotis Georgiou
Tryphon Georgiou
Stamatis Georgopoulos
Jessica Gephart
Yftach Gepner
Pierangelo Geppetti
Geetanjali Gera
Patrick Geraghty
Murilo Geraldo
Jennifer Gerard Ball
Jaline Gerardin
Patrick Gérardin
Nicole Gerardo
Rita Gerardy-Schahn
Maria Gerbase-Delima
Brian Gerber
Brian L. Gerber
David Gerber
Marcus Gerber
Jasper Gerbers
Susan Gerbi
Solweig Gerbier-Colomban
Kenn Gerdes
Norbert Gerdes
Victor Gerdes
Paul Gerdhem
Björn Gerdle
Marco Gerdol
Sharon Gerecht
Gernot Gerger
Andre Gergs
Glenn Gerhard
Thomas Gerhard
James Gerhart
Ingo Gerhauser
Richard Gerhold
Gabriele Gerlach
Robin Gerlach
Philip Gerlee
Roberto Gerli
Gregory Gerling
Francois Gerlotto
Pierre Germain
Philipp Germann
Ana Germano
Anastasios Germenis
Raphaele Germi
Pierre Germon
Giuseppe Gerna
Alison Gernand
Ramkishore Gernapudi
Morton Ann Gernsbacher
Ioannis Gerothanassis
Ioannis Gerothnasis
Grigoris Gerotziafas
Bruce Gerratt
Richard Gerraty
Karin Gerrits
Karin Gerritsen
Elizabeth Gershoff
Elliot Gershon
Michael D. Gershon
Michael Gershon
Timothy Gershon
Laurel Gershwin
Lisa-Ann Gershwin
Sarah Gerson
Aleeza Gerstein
Eitan Gerstner
Michael Gerth
William Gerthoffer

Alban Gervaise
Francesco Gervasio
William Gerwick
Russ Gesch
Jeff Geschwind
Antoine Gessain
Carla Gestich
Manuel Gesto
Belete Getahun
Godfrey Getz
Wayne Getz
Changiz Geula
Aldo Geuna
Aron Geurts
Michael Geusz
Reint H. Geuze
David Gewirtz
Milan Geybels
Felipe Geyer
Joachim Geyer
Julia Turbiner Geyer
Matthias Geyer
Ugur Gezer
Kate Gfeller
Sawsen Ghabri
Seyyed Abolghasem Ghadami
Nesreene Ghaddar
Ebrahim Ghaderi
Samad Ghaffari
Siddhesh Ghag
Arezou Ghahghaee
Shahram Ghanaati
Hamid Reza Ghanavi
Shahla Ghanbari
Shahram Ghandeharizadeh
Roopaly Ghandy
Aghareed Ghanim
Khaled Ghannam
Oula Ghannoum
Anita Ghansah
Mahdi Gharabaghi
Burhan Gharaibeh
Abdorasoul Ghasemi
Ali Ghasemzadeh
Yaser Ghavami
Majid Ghayour-Mobarhan
D. Ghazali
Arjumand Ghazi
Irfan Ghazi
Rahel Ghebre
Musie Ghebremichael
Mufaddal Gheewala
Maria Gheggi
Damien Gheldof
Dario Ghersi
Michelle Ghert
Pol Ghesquiere
Tullio Ghi
Gita Ghiasi
Mohammad Ghiasi
Soheil Ghiasi
Massimo Ghidinelli
Alessandro Ghidini
Michele Ghidini
Gian Marco Ghiggeri
Ada Ghiggia
Stefano Ghignone
Dirgha Ghimire
Massimiliano Ghinassi
Razvan Ionut Ghinea
Davide Ghinolfi
François Ghiringhelli
Stefano Ghirlanda
Rodolfo Ghirlando
Sara Ghiselli
Tahar Ghnaya
Anish Ghodadra
Arash Ghoddousi
Monali Ghodke
Yogita Ghodke-Puranik
Reza Ghodssi
Ali Gholami
Hassan Ghomrawi
Ahmed Ghoneima
Mohammad Ghorbanhoseini
Kaju Ghosal
Bishwajit Ghose
Asit Ghosh

Avik Ghosh
Dhruba Ghosh
Dipankar Ghosh
Ilora Ghosh
Indranil Ghosh
Kingshuk Ghosh
Manosij Ghosh
Pamela Ghosh
Paramita Ghosh
Rajarshi Ghosh
Rakesh Ghosh
Romi Ghosh
Ruby Ghosh
Sanjoy Ghosh
Santu Ghosh
Shyamasree Ghosh
Souvik Ghosh
Srikanta Ghosh
Srimoyee Ghosh
Sumantra Ghosh
Vijayalakshmi Ghosh
Gourab Ghoshal
Kalpana Ghoshal
Uday Ghoshal
Umesh Ghoshdastider
Abduwasit Ghulam
Prachi Ghule
Norbert Ghyselinck
Mauro Giacca
Andrea Giaccari
Matilde Giaccherini
Andrea Giachetti
Gabriele Giachin
Daniele Roberto Giacobbe
Peter Giacobbi
Roberto Giacomelli
Romina Giacometti
Daniele Giacoppo
Vito Giagulli
Angela Gialamas
Marcia Giambiagi-Demarval
Antonino Giambona
Francesco Giammarchi
Philip Giampietro
Luca Gianaroli
Elisabetta Gianazza
Ermete Giancipoli
Michele Gianella
Sara Gianella
Claudia Gianelli
Umberto Gianelli
Francesco Gianfagna
Fernando Gianfrancesco
Carmen Gianfrani
Felice Giangaspero
Sara Giangiobbe
Adriana Giangrande
Elisa Giani
Silvia Gianì
Emilio Antonio Luca Gianicolo
Gino Giannaccini
Giorgos Giannakakis
Konstantinos Giannakis
Anastasia Giannakopoulou
George Giannakoulas
Sergio Giannattasio
Alessio Giannelli
Ilias Giannenas
Elisa Giannetta
Maria Gianni
Riccardo Giannini
Gregory Giannone
Alberto Giannoni
Eric Giannoni
Peter Giannoudis
Alexia Giannoula
Nicola Gianotti
Francesco Giansantii
Andros T. Gianuca
Elena Gianulis
Efstathios Giaouris
Antonino Giaquinta
Maria Giardini
William Giardino
Cristina Giaroni
Filippo Giarratana
Benoit Giasson
Martin Gibala

Brian Gibbens
Emma Gibbin
Karen Gibbins
Christopher Gibbons
Sean Gibbons
Simon Gibbons
Adrian Gibbs
Allen Gibbs
Bethany Gibbs
Jo Gibbs
Karine Gibbs
Kenneth Gibbs
Roz Gibbs
Davide Gibellini
Robin Giblin-Davis
Katherine Gibney
Richard Gibney
Laure Gibot
Britton Gibson
C. Gibson
Deanna Gibson
Dustin Gibson
Gerusa Gibson
Jacob Gibson
Joshua Gibson
Mhairi Gibson
Oliver Gibson
Phil Gibson
Wendy Gibson
Jeremy Gibson-Brown
Stephen Gichuhi
Jonatha Giddens
Samuel Gidding
Hannah Gideon
Nicholas Gidmark
Clarissa Giebel
Motuzaite-Matuzeviciute Giedre
Katrin Giel
Henk Giele
Roben Gieling
Anne Giersch
Ed Giesbrecht
Ivone Giffard-Mena
Brian Gifford
Elizabeth Gifford
Miriam Gifford
Francesco Giganti
U. Giger
Katie Giger Seu
Gerd Gigerenzer
Jason Gigley
Anita Giglio
Daniel Giglio
Francis Gigliotti
Christopher Gignoux
Jacques Gignoux
Sylvain Gigout
Pierre Giguere
Marion Gijbels
Gabriel Gijon-Nogueron
Frank Gijsen
Helen G. Gika
Achilleas Gikas
Aristofanis Gikas
Anna Gil
Emilio Gil
Fernando Gil
Susana Gil
Yolanda Gilaberte
Kambiz Gilany
Moran Gilat
Amy Gilbert
Annie Gilbert
Christopher Gilbert
Clare Gilbert
Francis Gilbert
Hélène Gilbert
Joël Gilbert
Marius Gilbert
Martin Gilbert
Nicolas Gilbert
Paul Gilbert
Richard Gilbert
Robert Gilbert
Rodney Gilbert
Susan Gilbert
Diane Gilbert-Diamond
Edward Gilbert-Kawai

Suzanne Gilboa
Ian Gilby
Mercedes Gil-Campos
Sabine Gilch
Elizabeth Gilchrist
Gail Gilchrist
Michael Gilchrist
Gillian Gile
Amber Giles
Carla Giles
David E. Giles
Emma Giles
Lee Giles
Sam Giles
Wayne Giles
Andrew W. Gilg
Darren Giling
Renske Gilissen
Vanja Giljaca
Stacey Gilk
Bikram Gill
Christopher Gill
David Gill
Harvinder Gill
Jonathan Gill
Prit Gill
Sean Gill
Simone Gill
Sudeep Gill
Thomas Gill
Tiffany Gill
Morgane Gillard
Nathalie Gillard
Linn Gillberg
Yvonne Gilleece
Catherine Gillespie
Mark Gillespie
Joris Gillet
Pierre Gillet
Patrick Gillevet
Melissa Gilliam
Muriel Gillick
Glenda Gillies
Conor Gilligan
Beatriz Gil-Lopez
J. Gilman
Joanna Gil-Mohapel
José Antonio Gil-Montoya
Anna Gilmore
Camilla Gilmore
Ian Gilmore
Michael Gilmore
Stephen Gilmore
Heather Gilmour
Eustaquio Gil-Pelegrin
Nicholas Gilpin
Vicente Gilsanz
Paul Gilson
Erik Giltay
Jangwon Gim
Michael L. Gimbel
Fabricia Gimenes
Luis Giménez-Lirola
Suely Gimeno
Daniel Ginat
Maria Giné-Garriga
Juan Giner-Casares
Cosmina Gingaras
Anne Gingery
Rosa Gini
M. Ginja
Samantha Ginn
Yehuda Ginosar
Jean-Marc Ginoux
Joshua Ginsberg
Dan Ginsburg
Victor Ginsburgh
Filip Ginter
Donna Ginther
Karni Ginzburg
Yelena Ginzburg
David Giofrè
Tania Gioia
Michael Gionfriddo
Lara Giongo
Philippe Giordanengo
Paolo Giordani
Arturo Giordano

Emanuele Giordano
Luana Giordano
Ricardo Giordano
Silvia Giordano
A. Giorgetti
Sophie Giorgetti-Peraldi
Francesco Giorgianni
Paolo Giorgirossi
Nicholas Giori
Giacomo Giorli
Marcos Giovanetti
Marta Giovanetti
Manuela Giovannetti
Gavin Giovannoni
Roberto Giovannoni
Sonia Giovinazzi
Marco Giovine
Jessica Gipson
Henrique Girao
Benoît Girard
Christiane Girard
Pierre Marie Girard
Pierre-Marie Girard
Sylvie Girard
Raquel Girardello
Enrico Girardi
Thibaut Girardot
Matthieu Giraud
Enrico Giraudo
Hélène Giraudo
Jose Girela
Ayush Giri
Mona Giri
Pankaj Giri
S. S. Giri
Gonzalo Giribet
Thomas Girke
Alan Girling
Tsinuel Girma
Rossano Girometti
José-Antonio Girón-González
Julien Giron-Michel
Albert Girotti
Michael Giroux
Etienne Giroux Leprieur
Nicolas Gisch
Sebastian Gisder
David Gisen
Guro Giskeødegård
Angela Spontelli Gisselman
Magnus Gisslen
Mark Colin Gissler
Mika Gissler
Katja Gist
Daniel Gitaí
I. Z. Gitas
Michael Gitcho
Berenice Gitomer
Adriana Gittenberger-de Groot
Rachel Gittman
Dennis Gitz
Anna Giudetti
Linda Giudice
Oliver Giudice
Giuseppina Giulia-Mari
Alessandra Giuliani
Alessandro Giuliani
Bernal Giuliano
Rodolfo Giunchetti
Dimitri Giunchi
Paola Giussani
Emanuele Maria Giusti
Alain Givaudan
Nicolas Givaudan
Michael Givel
Charles Given
Babak Givi
Aleksander Giwercman
Dana Elena Giza
Belachew Gizachew
Agnieszka Gizak
Sotiria Gizani
Elke Gizewski
Alessio Gizzi
Leonardo Gizzi
Svein Gjelstad
Bergljot Gjelsvik
Erida Gjini

Nikolaos Gkantidis
Amanda Gladics
Angela Glading
Melissa Gladstone
Stefanie Glaeser
Mark Glaister
Olivier Glaizot
J. Glantz
Stanton Glantz
Thomas Glanville
Martin Glas
Tim Glasby
Jack Glaser
Sarah Glaser
Shannon Glaser
Jennifer Glass
Kathryn Glass
Marilyn Glassberg
Allison Glasser
Matthew Glasser
Adam Glassman
Amanda Glassman
Richard Glassock
Sarah Glastras
Torben Glatz
Clemens Glaubitz
Dominique Glauser
Courtney Glavis-Bloom
Amanda Glaze
Norbert Gleicher
Andrew Glencross
Lisa Glenk
Thomas Glenn
Joseph Glessner
Isaias Glezer
Patricia Glibert
Adam Glick
David Glick
Sara Glick
Joseph Glicksohn
Sabine Glienke
Elizabeth Glisky
William Glisson
Ros Gloag
Daniel Globisch
Ruth Globus
Sabine Glock
Gernot Glöckner
Rainer Gloeckl
Griet Glorieux
Martin Glos
Mary Glover-Amengor
Alexey Glukhov
Olena Glushakova
Nancy Glynn
W. H. Gmeiner
Fred G. Gmitter Jr.
Bruce Gnade
Timo Gnambs
Aswini Gnanasekaran
Agostino Gnasso
Vadim Gnatkovsky
Thomas J. Gniadek
Sedami Gnidehou
Danijela Gnjidic
Surya Gnyawali
Michael Go
Pål Goa
Emanuela Gobbi
Marco Gobbi
M. Gobbini
Hartmut Göbel
Denise Gobert
Eric Goberville
Ivana Gobin
Michael Gock
Swetha Godavarthi
Anuradha Godavarty
Amelia Goddard
Ben Godde
Pascal Godefroit
Julien Godet
H. Charles Godfray
Jacques Godfroid
Marco Godi
Gerardo Díaz Godínez
Susan Godlonton
Brian Godman

Justyna Godos
Thea Godschalk
P. Charles Goebel
Jeremy Goecks
Robert Goeder
James Goedert
Akshat Goel
Rahul Goel
Rajeev Goel
Reeta Goel
Sanjay Goel
Shailendra Goel
Craig Goergen
Stacy Goergen
Sandra Kathrin Goericke-Pesch
Matthew Goering
Holger Goerlitz
Paula Goes
Peter Goethals
Mark Goettel
Nicolai Goettel
Claudia Goettsch
Michael Gofeld
Laura Goff
Louise Goff
Eric Goffin
Richard Goffin
Christine Goffinet
Giovanni Battista Goffredo
Ameena Goga
Johann Peter Gogarten
M. Gögenur
Michael Goggins
William Goggins
Radhika Gogoi
Venkateswara Gogulamudi
Vladimir Gogvadze
George Goh
Hoe-Han Goh
Hui-Ting Goh
Kian Mau Goh
Suril Gohel
Antje Gohla
Polina Goichberg
Pablo G. Goicoechea
Pedro Gois
Jun Gojobori
Koichi Goka
Fulya Dilek Gökalp
Markus Göker
Moneisha Gokhale
Vladimir Gokhman
Nilüfer Goknar
Philippe Golay
Ilker Golcuk
Abby Gold
Barry Gold
David Gold
Jason Gold
Morris Gold
Stefan Gold
Slawomir Golda
Jeremy Goldbach
Daniel Goldberg
Elizabeth Goldberg
Gary Goldberg
Hanan Goldberg
Joanna Goldberg
Lynette Goldberg
Marcia Goldberg
Matthew Goldberg
Tony Goldberg
Christopher Golden
Sara Golden
Amit Goldenberg
Daniel Goldenberg
Robert Goldenberg
Norbert Goldfield
Joshua I. Goldhaber
Stephen J. Goldie
Basil Golding
Michael Golding
Nick Golding
Monica Goldklang
Alicia Goldman
Alyssa Goldman
Dana Goldman
Daniel Goldman

James Goldman
Jeremy Goldman
Roberta Goldman
Mary Goldring
Imeke Goldschmidt
Jeff Goldsmith
Paul Goldspink
Abraham Goldstein
Allan Goldstein
Bradley Goldstein
Gerald Goldstein
Harris Goldstein
Itamar Goldstein
Jenny Elaine Goldstein
Lara Goldstein
Neal Goldstein
Rebecca Goldstein
Risë Goldstein
Stephen Goldstone
Justyna Gołębiewska
Blanka Golebiowski
Edyta Gołembiewska
Nikola Golenhofen
Nady Golestaneh
Delia Goletti
Srinivas Goli
Abolghasem Golian
Priscila Goliatt
Michael Goligorsky
Mark Golitko
Ali Golizadeh
Lauren Gollahon
Yulia Golland
Carlos Goller
Juergen Goller
Sandra Gollnick
Erica Gollub
Sarah Gollust
Russell Golman
Beatrice Golomb
Julie Golomb
Maryna Golovchenko
Daniel Golparian
Rieta Gols
Jonathan Golub
Sarah Golüke
Pawel Golusinski
John Golz
Asmaa M. S. Gomaa
Huda Gomaa
Noha Gomaa
Adele M. Goman
Jesús Gomar
Monica Gomaraschi
Richard Gomer
Aldrin Gomes
Andreia Gomes
Beatriz Gomes
Cynthia Gomes
Edgar Gomes
Gabriela Gomes
João Gomes
Karina Gomes
Matheus Gomes
Nelson Gomes
Samirah Gomes
Sérgio Gomes da Silva
Mansueto Gomes Neto
Angelica Gomes Ueltschy
Maria Gomes-Solecki
Andres Gomez
Delphine Gomez
Esther Gomez
Felicia Gomez
Francisco Gomez
Gregorio Gomez
Hector Gomez
Hernando Gomez
Juan Gomez
Keith Gomez
Michael Gomez
Ricardo Gomez
Ángel Gómez
Juan Esteban Martínez Gómez
Karina Gómez
Magdalena Gómez
May Gómez
Ricardo Gómez

Rodolfo Gomez Bahamonde
Diego Gomez Baya
Hipólito Gómez Couso
Pedro José Gómez de Nova
Filippo Gomez Paloma
Elisa Gomez Perdiguero
Beatriz Gomez Perez-Nievas
Javier Gomez-Ambrosi
Jose Luis Gomez-Ariza
Nicolas Gomez-Banoy
Maria Jose Gomez-Benito
Juana Gómez-Benito
David Gomez-Cabrero
Alberto Gómez-Carballa
Marta Gomez-Chiarri
Bruno Gomez-Gil
Andres Gomez-Lievano
E. Gomez-Lucia
Alex Gomez-Marin
Fernando Carlos Gómez-Merino
Diego Gomez-Nicola
Antonio Gómez-Plana
Paulino Gomez-Puertas
Carola Gómez-Rodríguez
Paulina Gomez-Rubio
Jose Antonio Gomez-Sanchez
Eduardo Gómez-Sánchez
Stasha Gominak
F. Xavier Gomis-Rüth
Stephen Gomperts
Arthur Gomtsyan
Ludvik Gomulski
Joao Goncalves
Jorge Gonçalves
José Manuel Gonçalves
Juliane Gonçalves
Luzia Gonçalves
Rui Gonçalves
Sebastián Gonçalves
Teresa Gonçalves
Viviane Gonçalves
Mercia Aurelia Goncalves Leite
Rafael Gonçalves-Araujo
Gil Gonçalves-Miranda
Rodrigo Gonçalves-Oliveira
João Gonçalves-Pereira
Elena Goncharova
Thomas Gonda
Christopher Gondi
Florence Gondret
Elena Gonella
Sherif Gonem
Serap Gonen
Chengliang Gong
Diankun Gong
Erick Gong
Fangqi Gong
Hong-Yi Gong
Hui Gong
Jie Gong
Lei Gong
Liang Gong
Mengyan Gong
Michelle Gong
Minglun Gong
Neng Gong
Peng Gong
Qiang Gong
Tao Gong
Xiaohua Gong
Xu Gong
Xun Gong
Yanyan Gong
Yiping Gong
Yu-Shun Gong
Zhenwei Gong
Zhiyun Gong
Francesco Gongolo
Marisol Goni
Steven Gonias
Takehisa Gono
François Gonon
Tanja Gonska
Nihan Gonulol
Gustavo Gonzales
Luis Gonzales
Catherine Gonzalez
Cesar Gonzalez

Elena Gonzalez
Frank Gonzalez
Guadalupe Gonzalez
Javier Gonzalez
Jeffrey Gonzalez
Jorge Gonzalez
Manny Gonzalez
Maria Gonzalez
Mirna Gonzalez
Nathalie Gonzalez
Oscar Gonzalez
Sonia Gonzalez
Stevan Gonzalez
Suzanne Gonzalez
Camila González
Mercedes González
Laura González Acebrón
Anne Gonzalez de Peredo
Elisa Gonzalez Dominguez
Arlyng González Vázquez
Gregorio González-Alcaide
Carlos Gonzalez-Alcon
Carlos González-Alcón
Isidoro González-Álvaro
Pedro Gonzalez-Añover
Maria Gonzalez-Baro
Lucia Gonzalez-Buendia
Rafael Gonzalez-Cano
César González-Cantón
Aaron Gonzalez-Castro
M. C. González-Chávez
M. Gonzalez-Colaço Harmand
Humberto González-Díaz
Jorge González-Domínguez
Jesús González-Feliu
E. González-García
Miguel A. González-Gay
Mario Gonzalez-Gronow
José Ramón González-Juanatey
Rosaura Gonzalez-Mendez
Fernando Danilo Gonzalez-Nilo
Gemma González-Ortiz
Emilio Gonzalez-Parra
Gilberto Gonzalez-Parra
Rolando de Jesús Gonzalez-Peña
Mariano González-Pérez
Amelia González-Porto
Diego Gonzalez-Rivas
Ana Gonzalez-Roldan
Nestor Gonzalez-Roldan
Francisco Gonzalez-Scarano
Jara González-Silva
María Cristina González-Torres
Jose Gonzalez-Valdez
Juan P. González-Varo
Sixto González-Víllora
Gianluca Gonzi
Benjamin Good
Erin Good
Mary-Jo Good
Misty Good
Brian J. Goodacre
Sara Goodacre
Russell Goodall
Stuart Goodall
Mohammad Goodarzi
Farshad Goodarzi Boroojeni
Rose Goodchild
Karen Goodell
Dudley Goodhead
Eugene Goodilin
Kate Goodin
Alan Goodman
Barbara Goodman
Chelain Goodman
Christopher Goodman
David Goodman
James Goodman
Joel Goodman
Kenneth Goodman
Michael L. Goodman
Robert L. Goodman
Simon Goodman
Steven Goodman
Philip Goodney
John Goodpaster
Laurie Goodrich
Amador Goodridge

Helen Goodridge
David Goodsell
J. Max Goodson
Nicola Goodson
Patricia Goodson
Robert Goodspeed
Adam Goodwill
Anthony Goodwin
James Goodwin
John Goodwin
Renee Goodwin
Jonathon Gooi
Jeroen Goos
Victoria Goosey-Tolfrey
Evy Goossens
Brij Gopal
Satish Gopal
Natarajan Gopalakrishnan
Vidya Gopalakrishnan
Chaitra Gopalappa
Rafael Ruiz Gopegui
Vijayaprasad Gopichandran
Bamini Gopinath
Krishnamoorthy Gopinath
Anupama Gopisetty
Ruwan Gopura
Artur Gora
Dhruman Goradia
Valentin Gordeliy
Leonel Gordo
Ana Gordoa
Brett Gordon
Catherine Gordon
Christopher Gordon
Cristopher Gordon
David Gordon
Deborah Gordon
Evan Gordon
John Gordon
Joseph Gordon
Keith Gordon
Marion Gordon
Nicola Gordon
Noah Gordon
Reyna Gordon
Ulla Gordon
Vanessa Gordon-Dseagu
Aleksandra Górecka-Bruzda
Daniel Gorelick
Itamar Goren
O. Goren
Larry Gorenflo
Alemayehu Gorfe
Ashraf Gorgey
Frederic Gorin
Jean-Baptiste Gorin
Alfredo Gorio
Michael Gorman
Shelley Gorman
Matthew Gormley
German Gornalusse
Guy Gorochov
Elena Gorokhova
Jorg Goronzy
Philippe Gorphe
Josef Gorres
Andrea Gorrini
José Luis Gorriz
Anna Gorska
Andrzej Górski
Jonathan Gorstein
Florien Gorter
G. Gorton
Benjamin Gory
Yevgeniy Goryakin
Jessica Gorzo
Lara Goscè
Kirk Gosik
Anna Gosling
Becky Gosling
Yvan Gosmain
Grace Gosmann
Mary Gospodarowicz
Amy Goss
Dixie Goss
Donald Goss
Erica Goss
John Goss

Nathalie Gosselin
Sophie. Gosselin
Manfred Gossen
Philippe Gosset
Toni Gossmann
Sanchita Goswami
Santonu Goswami
Sumanta Goswami
Veena Goswami
Neha Gothe
Ruth Gotian
Akiteru Goto
Atsushi Goto
Eiji Goto
Katsumasa Goto
Masuo Goto
Rie Goto
Tadahiro Goto
Tsuyoshi Goto
Hiroki Gotoh
Tomomi Gotoh
Vincente Gotor-Fernandez
Alexander Gotschy
Riccardo Gottardi
Nicole Gottdenker
Martin Gotte
Matthias Gotte
Joel Gottesfeld
Ilan Gottlieb
Jacqueline Gottlieb
Joshua Gottlieb
Marcelo Gottschalk
Stephen Gottschalk
William Gottschalk
Carsten Gottschlich
Friedrich Götz
Timo Götzelmann
Mehdi Goudarzi
Steven Goudy
Aurélie Gouel-Chéron
Piebiep Goufo
Riadh Gouider
Daniel Goujet
Marilia Goulart
Dinah Gould
Francois Gould
Tom Gould
Tristan Goulden
Prabhu Gounder
M. J. Gounis
Samuel Gourion-Arsiquaud
R. M. Gous
Alexander Goushcha
Nadege Goutagny
Romain Goutagny
Myrofora Goutaki
Sandra Goutte
Joao Diogo Gouveia
Nélia Gouveia
Ricardo Gouveia
Anouk Gouvras
Jean Gouzi
Christian Gov
Hugh Govan
Muthusamy Govarthanan
Art Gover
Andrea Govetto
Dipti Govil
Chhabi Govind
Periyasamy Govindaraj
Annette Govindarajan
T. Govindaraju
Vijayendran Govindasamy
Govindjee Govindjee
Kristen Govoni
Elena Govorkova
Megan Gow
Neil Gow
Anilkumar Gowda
Chandrika Gowda
N. K. S. Gowda
Kymberly Gowdy
Brian Gowen
Adam Gower
Emily Gower
Jim Gower
Humaira Gowher
Josh Gowin

Rebecca Gowland
John Gowlett
Santhana Gowri
Félix Goyache
Amit Kumar Goyal
Aparajita Goyal
M. Goyal
Neeraj Goyal
Pankaj Goyal
Sachin Goyal
Sharad Goyal
Surendra Goyal
Swati Goyal
Jana Goyens
Holly Goyert
Bernard Goyette
Maira Goytia
Evelyne Gozal
Pedro Gozalo
Jeremy Grabbe
Michael Grabchak
Ed Grabczyk
Magnus Grabe
Michael Grabe
Adam Grabell
Fabian Grabenhorst
Judith M. Graber
Manfred Grabherr
Bryan Grabias
Urszula Grabiec
Chad P. Grabner
Roland Grabner
Desiree Grabow
Maggie Grabow
Przemyslaw Grabowicz
Timothy Grabowski
Krystyna Grabski
Gonçalo Graça
Vincent Gracco
James Grace
Mary Grace
Peter Grace
Elena Gracheva
Maarten Grachten
Luis Gracia
Carlos Gracia-Lazaro
Carolina Gracitelli
Emily Graczyk
Sibylle Grad
Sergio Gradilone
Luigi Gradoni
Marco Grados
Jaimie Gradus
Sue Grady
Cedric Graebin
Jennifer Graef
Shanna Grafeld
Lesley Graff
Stéphanie Graff-Dubois
Elizabeth Grafton-Cardwell
Loren Gragert
Sara Gragg
Andrew Graham
Annette Graham
Belinda Graham
Bruce Graham
David Graham
Delyth Graham
James Graham
Jay Graham
Kerr Graham
Mark Graham
Nick Graham
Rachel Graham
Reiko Graham
Ryan B. Graham
Stephen Graham
Stewart Graham
Wendy Graham
John Graham-Brown
Jennifer Graham-Engeland
Marc Graille
Benoit Graillot
Alessandro Grainer
Matthew Grainge
Jonathan Grainger
Tess Grainger
Harald Grallert

Lone Gram
Lu Gram
Hugo Gramajo
Maria Gramatges
Thomas Gramberg
Eliza Grames
Marina Gramiccia
William Gramlich
Paola Grammatico
Paul Grams
Jan Bert Gramsbergen
Julio Granados
Daniel Granato
David Granatstein
Sarah Granberg
Maria Grandahl
David Grande
M. Grande
Stuart Grande
Anjana Grandhi
Eleonora Grandi
Temple Grandin
Louis Grandjean
Marcus Granegger
Carlos Granell
Michael Graner
Roser Granero
Antonio Granero Gallegos
Cristina Grange
Thierry Grange
Christophe Grangeasse
Lotta Granholm-Bentley
Clémence Granier
Xavier Granier
Caterina Grano
Heather Gransee
Andrew Grant
Barth Grant
Denis Grant
Greg Grant
Ian Grant
Janet Grant
Jon Grant
Lafaine Grant
Lindsay Grant
M. J. Grant
Marie Clare Grant
Simon Grant
Struan Grant
W. Grant
William Grant
Lelede Grantina-Ievina
Ana Granville-Garcia
Alessandro Grapputo
Florian Gras
Jorge Grasa
Susanne Gräslund
Beatriz Gras-Miralles
Gregor Grass
Elena Grasselli
Giacomo Grasselli
Fabio Grassi
Fabrizio Grassi
Luigi Grassi
Massimo Grassi
Guntram Grassl
Nicholas Grassly
Andre Grassmann
Sandrine Grasteau
Jonathan Gratch
Aurelien Graton
Dave Grattan
Enrico Gratton
Maud Gratuze
Jim Grau
María Grau
Oriol Grau
Stacy Landreth Grau
Veronika Grau
Marit Graue
M. Graupera
Heinrich Grausgruber
Francielle Graus-Nunes
Stavros Gravas
Claudia Gravekamp
Sarah Gravem
Dana Graves
Lee Graves

Stephen Graves
Steven Graves
Michael Gravett
Giovanni Luca Gravina
Janko Gravner
Nathan Grawe
Andrew Gray
Christine L. Gray
David Gray
Debra Gray
Elin Gray
Emilie Gray
Gregory Gray
Jason Gray
Joshua C. Gray
Lincoln Gray
Nicola Gray
Peter Gray
Richard Gray
Robert Gray
Ryan Gray
Stephen Gray
Steven Gray
Peter Graystock
Francesca Graziano
Serge Grazioli
Helena Grcman
John Greany
Molly Grear
K. Leigh Greathouse
Thomas Greb
Carola Grebitus
Jacqueline Grebmeier
Luca Greci
Alberto Greco
John Grecula
Barry Green
Dannielle Green
Eric Green
James Green
Jamison Green
Janet Green
Jennifer Green
Jessica Green
Judith Green
Lucy Green
Mark Green
Michael Green
Nancy Green
Paul J. Green
Robert Green
S. Green
Sara Green
Sherril Green
William Green
Alan Greenberg
Andrew Greenberg
Arthur Greenberg
David Greenberg
Jared Greenberg
Josh Greenberg
Robert Greenberg
Joel Greenberger
Catherine Greene
Ciara Greene
Daniel Greene
Jeremy Greene
Justin Greene
Lois Greene
Mark Greene
Michael Greene
Nathaniel Greene
Scott Greene
Sharon Greene
William Greene
Ben Greenfield
David Greenhalgh
Neil Greening
Justin Greenlee
C. Greenlief
Sarah Greenman
Penelope Greenslade
Penelope J. M. Greenslade
John Greenspan
Shane Greenstein
Aaron Greenville
Roby Greenwald
Scott Greenwald

Matthew Greenwold
Brian Greenwood
Iain Greenwood
J. Greenwood
Jennifer Greenwood
Nan Greenwood
Sue Greenwood
Susan Greenwood
Dennis Greer
Joseph Greer
Paul Greer
Giuliana Gregato
Justin R. Gregg
Michael Gregg
Marie-Claude Grégoire
Ingo Gregor
Gustavo Gregoracci
Giovanni Gregori
Matjaž Gregorič
Carol Gregorio
Chris Gregory
Emily F. Gregory
James Gregory
Laura Gregory
Richard Gregory
Thomas Greig
Steffen Greilich
Andreas Greinacher
Stefan Greisenegger
Tobias Greitemeyer
Sasha Grek
Emily Grekin
Philippe Grellier
Hendrik Gremmels
Ryszard Grenda
James Grenert
Karen Grepin
Mark Gresnigt
Tanya Gressley
Ian Grettenberger
Norbert Gretz
Douglas Greve
Mark Greve
Dennis Grevenstein
James Greville
Felix Grewe
Christoph Grewer
Margaret Grey
Bruce Greyson
Ingeborg Grgurina
Paul Gribben
Irena Gribovskaja-Rupp
Eva Maria Griebeler
Yannick Griep
Pauline Grierson
Gerhard Gries
Uta Griesenbach
Roderick Grieves
Klaus Griewank
Blaine Griffen
Amy Griffin
Brandon Griffin
Donal Griffin
Greg Griffin
James Griffin
Jamie Griffin
Joan Griffin
LaToya M. Griffin
Leanne Griffin
Lewis Griffin
Lisa Griffin
Philip Griffin
Susan Griffin
Jack Griffith
James Griffith
Mike Griffith
Patrick Griffith
Thomas Griffith
Todd Griffith
Anthony Griffiths
Bryan Griffiths
Ewen A. Griffiths
Kristi Griffiths
Richard Griffiths
Seren Griffiths
William Griffiths
Marieke Griffoen
Tomás Grigera

John Grigg
Ryan Griggs
Stefano Grignolio
Michel Grignon
Maria Grigoriou
Boyan Grigorov
Larissa Grigoryan
Maria Eugenia Grillet
Renato Grillo
Shellee Grim
D. Grimaldi
Salvatore Grimaldi
Gabriel Grimaldi Jr.
Francisco Grimaldo
Katerina Grimani
David Robert Grimes
Bernhard Grimm
Dirk Grimm
Giso Grimm
Herwig Grimm
Jan Grimm
Kevin J. Grimm
Michael Grimm
Paul Grimm
Sabine Grimm
Tobias Grimm
Karen Grimmer
Franco Grimolizzi
Eleni Grimpampi
Jerome Grimplet
Anna Grimsrud
John Grindeland
Kelly Grindrod
Mariusz Grinholc
Evelina Griniene
Frederick Grinnell
Jonathan Grip
Cristina Grippaudo
Paul Grippo
Denis Gris
Michael Grisham
Isabella Grishkan
Olaf Grisk
Lee Grismer
Laura Grisotto
Valeriya Gritsenko
Nikolaos Grivas
Petros Grivas
Fabio Grizzi
Raymond Grizzle
Mirjam Grobbel
Nadja Grobe
Guy Groblewski
Michelle Grocke
Lucie Grodecká
Semyon Grodsky
Jürgen Groeneveld
Peter Groenewegen
Rolf Groenwold
Matthias Groeschel
Dorothy Grogono
Ursula Grohmann
Yrjö Gröhn
Guillaume Grolez
M. Michael Gromiha
Eric Grommon
Kirill Gromov
Kurt Gron
Alessandro Gronchi
Kirsten Grond
Dieter Groneberg
Karsten Gronert
Petra Gronholm
Scott D. Gronlund
Ulrika Gronlund
Gloria Gronowicz
Emily Gronseth
Thomas Grönthal
Wolfram Gronwald
Astrid Groot
Christopher Groot
Maria Groot
Tijl Grootswagers
Michael Gropper
Frédéric Gros
Marie-Hélène Grosbras
Chester Grosch
Antje Grosche

Stefan Grosek
Brigitte Grosgogeat
Fabrizio Grosjean
Yael Grosjean
Julie Gros-Louis
Sidney Grosprêtre
Aaron Gross
Alan Gross
Andrew Gross
Briana Gross
Elena Gross
Eric Gross
Jeferson Gross
Jörg Gross
Josef Gross
Joshua Gross
Julia Gross
Volker Gross
Jonathan Grossberg
Robert Grosse
Scott Grosse
Laís Grossel
Jean-Francois Grosset
Moritz Grosse-Wentrup
Ewald Grosse-Wilde
Nicola Grossheinrich
Dave Grosshuesch
Gerarda Grossi
Ari Grossman
Ashley Grossman
Daniel Grossman
Ruth Grossman
Steven Grossnickle
Giuseppe Grosso
Rüdiger Grote
Karin Groten
David Groth
Axel Grothey
Hanna Grothmann
Oliver Grottke
Andrea Grottoli
Huw Groucutt
Cyril Grouin
Mathieu Groussi
Bill Groutas
Abhinav Grover
Martha Grover
Neeraj Grover
Renaud Grover
Sandeep Grover
Sonam Grover
Andy Groves
Mari Groves
Lars Grøvle
André Grow
Edward Grow
Christina Grozinger
Agnès Gruart
Robert Grubb
Allison Gruber
Jens Gruber
Karl Gruber
Ludwig Gruber
Reinhard Gruber
Sabine Gruber
Tony Grubesic
Lisa Grubisha
Sergei Grudinin
Catherine Grueber
Oliver Gruebner
Sonja Gruen
Hannah Gruffudd
Ireneusz Grulkowski
Holger Grüll
Anete Sevciovic Grumach
Rebecca Grumet
Warren Grundfest
Oliver Grundmann
Anne Grundy
John Grundy
Quinn Grundy
Scott Grundy
Tim Grüne
Andrea Gruneir
Cariline Grunenwald
Gabriele Grunig
Robert Gruninger
Daniel Grunspan

Gerald Grunwald
Jocelyn Grunwell
Philip Gruppuso
Christof Grüring
Alexandra Gruss
Daniel Gruss
Matthew Gruwell
John Gruzelier
Jonathan Gryak
Elena Gryazina
Mark Grygier
Marc Grynpas
Ouriel Grynszpan
Lukasz Grzeczkowski
Lukasz Grzeskowiak
Andrzej Grzybowski
Can Gu
Changgui Gu
Dangen Gu
Dongfeng Gu
Dongsheng Gu
Gao-Feng Gu
Guoying Gu
Huaxi Gu
Jianguo Gu
Jie Gu
Junjuan Gu
Keyu Gu
Lianfeng Gu
Linlin Gu
Liqun (Andrew) Gu
Min Gu
Peili Gu
Qilin Gu
Qiong Gu
Runxia Gu
Tongjun Gu
Weifeng Gu
Weikuan Gu
Wenyi Gu
Xiaomei Gu
Xiaowu Gu
Y. Gu
Yan Jun Gu
Yang-Guang Gu
Yangnan Gu
Yanhong Gu
Ye Gu
Yihua Gu
Yu Gu
Zhen Gu
Rodrigo Guabiraba
Antonio Guaita
Maria Gualano
Victor Guallar
Diego F Gualtero
Fabio Gualtieri
Roberta Gualtierotti
Donghai Guan
Min Guan
Qingmei Guan
Shengxi Guan
Tao Guan
Wenjian Guan
Yi Guan
Yongqiang Guan
Riccardo Guanà
Frederico Guanais
Du Guangwei
Giovanni Guaraldi
Pietro Guaraldi
Fabio Guarany
Luca Guardabassi
Ornella Guardamagna
Giulia Guarguaglini
Manuel Guariguata
Vladimiro Guarnaccia
Jeannette Guarner
Fay Guarraci
Fabio Guarracino
Eduard Guasch
Leonardo Guasti
Luigina Guasti
Debbie Guatelli-Steinberg
Carmen Guaza
Marco Guazzone
Ricardo Gucci
Enza Gucciardi

Ivana Gudelj
Severin Gudima
Esayas Gudina
Carlota Gudiol
Eyjolfur Gudmundsson
Joachim Gudmundsson
Wallentin Gudrun
Guilherme Guedes
Thaís Guedes
Vivian Guedes
Eric Guedj
Jeremie Guedj
Laurence Guédon-Moreau
Erwan Gueguen
Cecile Gueidan
Isabelle Guellec
Anis Guelmami
María Guembe
Cagatay Guenes
Line Guénette
Dominik Guensch
Andreas Guenther
Sebastian Guenther
Onur Guentuerkuen
Federico Guercilena
Pierre Gueriau
Greg Guerin
Philippe Guerin
Raphaël Guérois
Angel Guerra
Carmen Guerra
Concettina Guerra
Javier Guerra
Rudy Guerra
Sylvia Guerra
José Afonso Guerra-Assunção
Lucia Guerra-Reyes
Susana Guerreiro
Ida Chiara Guerrera
Elián Guerrero
Antonieta Guerrero-Plata
Alfredo Guerreros
David Guerrero-Setas
Michele Guerreschi
Uliano Guerrini
Dominique Guerrot
Claude Guertin
Michael Guertin
Romain Guery
Benjamin Guesdon
Andrew Guess
James Guest
Julian F. Guest
Richard Guest
Timothy Guetterman
Laetitia Guevel
Ndeye-Aicha Gueye
Zbigniew Gugala
Felix Gugerli
Matthias Guggenmos
Muriel Gugger
Paul Gugger
William Guggino
Valeria Guglielmi
Jean Guglielminotti
Bernard Guglielmo
Letterio Guglielmo
Chandan Guha
Prajna Guha
Shion Guha
Sonia Guha
Huan Gui
Jian-Fang Gui
Jie Gui
Kai Gui
Christelle Guibert
Edgardo Guibert
Bertrand Guidet
Paolo Guidetti
Giancesare Guidi
Rafael Guido
Bruno Guigas
Brice Guignard
Yann Guiguen
Beatriz Guijarro
Francisco Guijarro
Maria Guijarro
William Guilford

Olivier Guillaume
Fabienne Guillaumond
Karen Guillemin
Marie-Laure Guillemin
Nicolas Guillemin
Christian Guilleminault
Laurent Guilleminault
George Guillen
Nancy Guillen
Karina Guillen Navarro
Thomas Guillerme
Maude Guillier
Benoit Guillon
Quentin Guillon
Casey Guillot
Jacques Guillot
Sophie Guillot
Hervé Guillou
Jean-Philippe Guilloux
Daniel Guimarães
Fernando Guimarães
Murilo Guimarães
Nuno Guimarães-Camboa
Michele Guindani
Sarah Guindre-Parker
Celine Guinez
Benjamin Guinhouya
Pierre-Grégoire Guinot
Jose Guinot Saporta
Ruth Guinsburg
J. Guiot
Marianne Guiral
Faheem Guirgis
Amélie Guitart
Pascale Guitera
Stéphane Guitet
Rachel Guiton
Matthieu Guitton
Sergi Guixe-Muntet
Marina Guizetti
Katell Guizien
Shashi Gujar
Muhammet Gul
Noor Gul
Taza Gul
Omer Faruk Gulban
Jacqui Gulbis
Şaban Gülcü
Per Guldberg
Robert Guldemond
Christian Güldner
Ali Guler
Aysegul Selcen Guler
Emre Guler
Marco Gulewitsch
Monika Gulia-Nuss
Andrew Gulick
Annamaria Gullà
Rao Gullapalli
Margaret Gulley
Martin Gulliford
Jan Gulliksen
Matthew Gullo
Martin Gullstrom
Hamza Güllü
Amber Gum
James Gumbart
Freedom Gumedze
Sathyanarayana Gummadi
Jonathan Gumucio
Pinar Gumus
Michelle Gumz
Karthigayan Gunalan
Manjula Gunawardana
Cengiz Gunay
Mehtap Günay
Konstantin Gunbin
Gregor Guncar
Resign Gunda
Venugopal Gunda
Rohit Gundamaraju
Craig Gundersen
Fusun Gundogan
Anil Gundu
Mehmet Gunes
Ece Güneş
Monica Gunetti
Alistair Gunn

Michael Gunn
Ingibjorg Gunnarsdottir
Gunnar Gunnarsson
Stefan Gunnarsson
Ulf Gunnarsson
Sarah Gunnery
Amy Gunning
Peter Gunning
Raf Guns
Catrin Gunther
Orlando Guntinas-Lichius
Jenny Gunton
Arthur Gunzl
Anyuan Guo
Baocheng Guo
Benchang Guo
Boliang Guo
Chao Guo
Chao-Yu Guo
Cheng-Xian Guo
Chuanyong Guo
Chunming Guo
Dan Guo
Daqing Guo
Fei Guo
Feiye Guo
Feng Guo
Fengjie Guo
Grace Guo
Guo Guo
Haitao Guo
Haixun Guo
Hongbin Guo
Hua Guo
Huijun Guo
Jianhua Guo
Jianping Guo
Jian-You Guo
Jiasong Guo
Jiguang Guo
Jing Guo
Jingyu Guo
Jingzhe Guo
Jong-Long Guo
Junke Guo
Min Guo
Ming Guo
Minglei Guo
Nick Guo
Ning Guo
Pengfei Guo
Qian-Hui Guo
Qunying Guo
Rey-Ting Guo
Rongwei Guo
Shanshan Guo
Shuangzhuang Guo
Song Guo
Songchang Guo
Sophie Huey-Ming Guo
Teng Guo
Tiankui Guo
Wei Guo
Weichao Guo
Wenbin Guo
Wenshan Guo
Wenwu Guo
Xiao Guo
Xiaoya Guo
Xiaoyi Guo
Xinxing Guo
Xiong Guo
Xiuqing Guo
Yadong Guo
Yanrong Guo
Yanyong Guo
Yingnan Guo
Yiting Emily Guo
Yongjun Guo
Yuanming Guo
Yunfei Guo
Yunliang Guo
Yuqing Guo
Yuxuan Guo
Zaihua Guo
Zhanhu Guo
Zhenheng Guo
Zhenhua Guo

Zhigang Guo
Zhiqiang Guo
Zhongmin Guo
Shen Guozhen
Liu Guozhong
Christopher Guppy
Aditi Gupta
Akash Gupta
Anuj Gupta
Bhagwati Gupta
Bhavna Gupta
Deepika Gupta
Devanand Gupta
Dipika Gupta
Dorin Gupta
Ekta Gupta
Kalpna Gupta
Manveen Gupta
Nabanita Gupta
Navin Gupta
Nimesh Gupta
Nishant Gupta
Pankaj Gupta
Phalguni Gupta
Rachna Gupta
Rajeev Gupta
Rangan Gupta
Rishein Gupta
S. D. Gupta
Sabhyata Gupta
Sanjay Gupta
Sanjeev Gupta
Sanjiv Gupta
Shamba Gupta
Shishir Gupta
Snehasish Gupta
Subash Gupta
Subodh Gupta
Swati Gupta
Tripti Gupta
Umesh Gupta
Vikas Gupta
Vivek Gupta
Vk Gupta
Yogesh Gupta
Purnananda Guptasarma
Komal Gupte-Singh
Gozde Gur
Tamar Gur
Bala Gur Dedeoglu
Candan Gürakan
Elie Gurarie
Yogesh Gurav
Filiz Gürel
Eugenia Gurevich
Michael Gurevich
Artur Gurgul
Cemal Gurkan
Umut Gurkan
Tami Gurley-Calvez
James Gurney
Olga Gursky
Ozge Gursoy-Yuzugullu
Rajendra Gurung
Kotambylu Gururaja
Michael Gurven
Caroline Gurvich
Diego Gurvich
David Gurwitz
Nikolai Gusev
Michael Gusmano
Leonor Gusmão
Mitchell Guss
Emanuela Gussoni
Alison Gustafson
Christopher Gustafson
Clay Gustafson
Jan-Ake Gustafsson
Kenth Gustafsson
Ian Gut
Adrian Guta
David Gute
Elan Guterman
Yoram Gutfreund
Martina Guthoff
Rudolf Guthoff
Joanne Guthrie
Robson Gutierre

Fernando Gutierrez
Jose Gutierrez
Antonio Gutierrez de Blume
Alessandra Gutierrez Oliveira
Alfonso Gutierrez-Adan
Martha Gutiérrez-Aguirre
Mario Gutiérrez-Bedmar
Julián Gutiérrez-Escobar
Braulio Gutiérrez-Medina
José Gutiérrez-Pabello
Antonio Gutiérrez-Pizarraya
Mario Gutiérrez-Roig
Gonzalo Gutiérrez-Tobal
Annelie Gutke
Jessica Gutknecht
Ivan Gutman
K. Gutschow
Vishwesha Guttal
Michael Gutter
Michael Guttery
David Guttman
Emma Guttman-Yassky
Esin Guven
Emine Guven-Maiorov
Franck Guy
Anna Guyatt
Gordon Guyatt
Bernard Guyer
Christophe Guyeux
Melissa Guynn
Stephane Guyot
David Guyton
Nihal Guzel
Tomasz J. Guzik
Christian Guzman
David Sanchez-Migallon Guzman
Frank Guzman
Monica Guzman
Raul Guzman
Camilo Guzmán
José Guzmán
Rocío Guzmán
Alerie Guzmán de la Fuente
Maria Guzman-Castillo
Ernesto Guzman-Novoa
Caterina Guzman-Verri
Iko Guzulgen
Goran Gužvica
Mariangela Guzzardi
Goude Gwenaëlle
Thando Gwetu
Kimberly Gwinn
O. Scott Gwinn
Anna Gyarmathy
Brian Gygi
Marian Gyongyosi
Sandor Gyorke
Wilfried Gyselaers
Miklós Gyuranecz
Chien Ha
Chul-Won Ha
Chunfang Ha
Hojin Ha
In-Hyuk Ha
Kwon-Soo Ha
Nam-Chul Ha
Patrick Ha
Sang-Jun Ha
Seung Ha
Shang Ha
Su Ha
Ya Ha
Yong-Chan Ha
Youn Doh Ha
Robert Haack
Austin Haag
David Haaga
Juanita Haagsma
Daniel Haag-Wachernagel
Christine Haaland
Markus Haapasalo
Birger Haarbrandt
Alexis Haas
Andreas Haas
Fernando Haas
Tara Haas
Astrid Haase
Elaine Haase

Hajo Haase
Kristina Haase
Nicolai Haase
Volker Haase
Carolina Haass-Koffler
José Haba-Rubio
Michael Habeck
Antje Habekuss
Noah Haber
Paul Haber
Helene Häberle
Cory Haberman
Abdulrazaq Habib
Farhat Habib
Nagy Habib
Ghader Habibi
Maryam Habibi
David Habiel
Mounira Habli
Pascal Hablützel
Kassahun Habtamu
Gokce Hacioglu
Daniel Hackett
Ruth Hackett
Troy Hackett
T. J. Hackmann
Anthony Hackney
David Hackney
Ted Hackstadt
Farzad Hadaegh
Yitzhak Hadar
Pierre Haddad
Rodrigo Haddad
Parham Haddadi
Ram Haddas
James Haddow
Lewis Haddow
Sandile Hadebesta
Khaled Hadhri
Muhammad Hadi
Ahmed Hadidi
Fadila Hadj-Bouziane
Christos Hadjichristodoulou
Maria Hadjiconstantinou-Neff
Maria Hadjifrangiskou
George Hadjigeorgiou
Michalis Hadjikakou
Maria Claret Hadler
Daniela Hadorn
Niels Hadrup
Denita Hadziabdic
Rainer Haeckel
Danny Haelewaters
Gregory Haenel
Marisa Haenni
Corinna Haenschel
Hadi Haeri
Sonja Haertle
Thomas Haertle
Thomas Haertlé
Frédéric Haesebaert
Tessa Haesevoets
Daniel Haeusser
Julius Clemence Hafalla
Christof Haffner
Michael Haffner
Melanie Haffner-Luntzer
Brian Hafner
Patricia Hafner
Michael Häfner
Andrew Hafs
Gertrud Hafstad
Yuki Haga
Christopher Hagan
Kaitlin Hagan
Mats Hagberg
Chadi Hage
Steffen Hage
Paulien Hagedoorn
Linda Hagedorn
Gregor Hagelueken
Jesper Hagemeier
Marne Hagemeijer
Bjoern Hagen
Ellen Merete Hagen
Ferry Hagen
Scott Hagen
Bruno Hagenbuch

Mette Hagensen
S. J. Haggarty
Olle Häggström
Reza Haghighi
Akihito Hagihara
Daisuke Hagiwara
Nao Hagiwara
Shinichiro Hagiwara
York Hagmayer
Franz Hagn
William Hague
Torsten Hahmann
Amanda Hahn
Andreas Hahn
Daniel Hahn
Federico Hahn
Ingo Hahn
Ji-Sook Hahn
Judith Hahn
Juergen Hahn
Mariah Hahn
Matthias Hahn
Michael Hahn
P. Richard Hahn
Philip Hahn
Richard Hahn
Tim Hahn
Jennifer Hahn-Holbrook
Michael Hahsler
Debbie Hahs-Vaughn
Lan Hai
Rong Hai
Shi Hai
Yong Hai
Hossam Haick
Malak Haidar
Ch. Md. Rakin Haider
Haula Haider
David Haig
Candace Haigler
Shalin Hai-Jew
Demewoz Haile
Luke Haile
Frank Hailer
Asrat Hailu
Jess Haines
Atticus Hainsworth
M. R. Razean Haireen
Fariza Hairi
Zied Haj-Amor
Eric Hajduch
Milan Hajek
Shahin Hajibandeh
Mohammadhossein Hajiebrahimi
Mohammad Hajihosseini
George Hajishengallis
Inaya Hajj Hussein
Waseem Hajjar
Rejab Hajlaoui
Gyorgy Hajnoczky
Mihaly Hajos
Jussi Hakala
Antoine Hakim
Djelouah Hakim
Hakon Hakonarson
Carmen Halabi
Harry Haladjian
Eliran Halali
A. Halaris
Natasha Halasa
Heli Halava
Hadeel Halaweh
Demetrios Halazonetis
Richard Halberg
Patrick Halbur
Yaroslav Halchenko
Gert Hald
Chandana Haldar
Devyani Haldar
Rolf Halden
Genevieve Hale
Jack Hale
Laura Hale
Lauren Hale
Rebecca Hale
Claire Hales
Derek Hales
Andrew Halestrap

Guillaume Halet
Andreana Haley
Connie Haley
Robert Haley
William Haley
Jochen Halfar
Peter Halfmann
Oday Halhouli
Nuray Haliloglu
Nafisa Halim
Sofia Halin Bergström
Lukasz Halinski
Fabien Halkett
Arja Halkoaho
Amanda Hall
April Hall
Bradley Hall
Brian Hall
Bruce Hall
Chad Hall
Deborah Hall
Emily Hall
Eric Hall
H. Irene Hall
Jean Hall
John Hall
Joshua Hall
Judith Hall
Kathleen Hall
Laurie Hall
Lawrence Hall
Marissa Hall
Mark C. Hall
Matthew Hall
Nigel Hall
Ralph Hall
Robert Hall
Wayne Hall
Nadim Hallab
Brian Hallahan
Dean Hallam
Jeffrey Hallam
Mryka Hall-Beyer
Brynjar Halldorsson
Gustaaf Hallegraeff
Joannes Hallegraeff
Heather Hallen-Adams
Florian Haller
Maria Haller
Sebastian Haller
Toomas Haller
Kadri Haller-Kikkatalo
Michael Hallett
Penelope Hallett
Richard Hallett
John Halley
Kevin Hallgren
Mats Hallgren
William Halliday
Erik Hallin
Lauren Hallion
Tyler Hallman
William Hallman
Johannes Hallmann
Sonja Hall-Mendelin
Jason Hall-Spencer
Seth Hallstroem
Michael Halmagyi
John Halperin
Andrea Halpern
Scott Halpern
Jamie Halsall
Eric Halsey
Kimberly Halsey
Lewis Halsey
Brian Halstead
Scott Halstead
Mary Halter
Arda Halu
Karl Halupka
Bente Halvorsen
Kathy Halvorsen
Halvor Halvorson
Hikabasa Halwiindi
Jong Hyun Ham
Won Sik Ham
Aldric Hama
Rita Hamad

Fumika Hamada
Kazunori Hamamura
Gen Hamanaka
Elena Hamann
Mark Hamann
Michael Hamann
Silke Hamann
Steffen Hamann
Laurence Hamard
Yoshifumi Hamasaki
Kei Hamazaki
Peter Hamback
Anna-Karin Hamberg
Ridha Hambli
Shaheen Hamdy
Ali Hamedani
Wolfgang Hamel
Davidson Hamer
Gabriel Hamer
Geert Hamer
Mark Hamer
Petra Hamerlik
Karim Hamesch
Pavel Hamet
Saeed Hamid
Syed Hamid Sherazi
Mohammad Hamidaddin
Mohammadreza Hamidi
Amir Ali Hamidieh
Josiph Hamill
Bruce Hamilton
Christopher Hamilton
Cyd Hamilton
David Hamilton
Douglas Hamilton
Erin Hamilton
Joshua Hamilton
Kerry A. Hamilton
Louise Hamilton
Mark Hamilton
Nick Hamilton
William Hamilton
Andrew Hamilton-Wright
Elissa Hamlat
J. Kiley Hamlin
Jeff Hamm
Robert Hamm
Karen Hammad
Sarah Hamm-Alvarez
Harald Hammarström
Marie-Louise Hammarström
K. Larry Hammell
Daniel Hammenfors
Gary Hammer
Joseph Hammer
Niels Hammer
Sabine Hammer
Heinz Hammerlindl
Alexander Hammers
Margaret Hammerschlag
Neil Hammerschlag
Kurt Hammerschmidt
Wolfgang Hammerschmidt
Richard Hammersley
Anette Hammerum
Theodore Hammett
Edd Hammill
Mike Hammill
Bruce Hammock
Harald Hammon
Ashley Hammond
Constance Hammond
Rosemarie Hammond
S. Hammond
Talisin Hammond
William Hammond
Ahmad Hammoud
Mohamed Hammoud
Melanie Hamon
Thierry Hamon
Yannick Hamon
Ayman Hamouda
Jochen Hampe
Wolfgang Hampe
Daniela Hampel
Ken Hampel
Axel Hamprecht
Fred Hamprecht

David Hampson
Katie Hampson
Lynne Hampson
Robert Hampton
Pedram Hamrah
Harald Hamre
James Hamrick
Zoe Hamstead
Melvyn Hamstra
Jawad Hamza
Lisa Hamzah
Beth Han
Bing Han
Byung Woo Han
Cheng-Gui Han
Dale Han
Daphne Han
Dunzheng Han
Eugene Han
Eun-Taek Han
Fei Han
Guangxuan Han
Hahrie Han
Haiyong Han
Hongyan Han
Jeffrey Han
Jian-Lin Han
Jianping Han
Jianyong Han
Jing Han
Jinguang Han
Jingyan Han
Jing-Yan Han
Julianna Han
Jun Han
Jung-Soo Han
Ke-Li Han
Kiho Han
Kyudong Han
Lanzhi Han
Leng Han
Liebao Han
Longzhi Han
Oksoo Han
Peng Han
Rui-Qi Han
Sang-Sun Han
Sang-Uk Han
Seung Hyeok Han
Seungyeup Han
Seung-Yong Han
Shengtong Han
Shihui Han
Sileny Han
Song-I Han
Sushan Han
Wei Han
Xiao-Pu Han
Xiaozeng Han
Xu Han
Xuesong Han
Yeon Soo Han
Ying Han
Yingying Han
Yong Han
Yonghua Han
Yuchi Han
Yu-San Han
Zhenhai Han
Zongchao Han
Zongfu Han
Ahmad Ali Hanafi-Bojd
Norio Hanafusa
William Hanage
Ken-Ichi Hanaki
Ali El Hanandeh
Hironari Hanaoka
Stefania Hanau
Merlin Hanauer
Allan Hanbury
Pascale Hancart Petitet
C. Nathan Hancock
Jeff Hancock
Lynn Hancock
Meaghan Hancock
Peter Hancock
Robert Hancock
William Hancock

Ewa Hanczakowska
Martin Hanczyc
Nicholas Hand
Timothy Hand
Avtar Handa
Rajash Handa
Garry Handelman
Janine Handforth
Elizabeth Handorf
Sara Handy
Susan Handy
Stefan Haneder
Romana Haneef
Ulf Hanefeld
Dominik Hangartner
Jürgen Hänggi
Priscila Hanisch
Laura Hanish
Andrew Hanks
John Hanks
Kathryn Hanley
Torrance Hanley
Charlotte Hanlon
Ana Hanlon-Dearman
Patrick Hanly
Hie-Won Hann
Alex Hanna
David Hanna
Jandy Hanna
Louise Hanna
Philip Hanna
Anthony Hannan
Kelly Hannan
S. Hannan
S. Hannawi
Yousef Hannawi
Abdelfattah Hanno
Breffni Hannon
Erin Hannon
Abdelali Hannoufa
Jarna Hannukainen
Yaniv Hanoch
Salem Hanoun
Jorg Hanrieder
Chetan Hans
Franziska S. Hanschen
Donna Hansel
Allison Hansen
Benjamin Hansen
Camilla Hansen
Christian Hansen
Egon Hansen
Gail Hansen
Gorm Hansen
Jason Hansen
Jeffrey Hansen
Jonathan Hansen
Keith Hansen
Klavs Hansen
Kristian Hansen
Lise Lotte Hansen
Marc Hansen
Marie-Luise Hansen
Peter Hansen
Rikke Hansen
Ronald Hansen
Solveig Hansen
Thor Willy Ruud Hansen
Tine Hansen
Ulla Hansen
Wiebke Hansen
Simon Hanslmayr
Florian Hansmann
J. Hansmann
Ulrich Hansmann
Franck Hansmannel
Claudia Hanson
Corrine Hanson
Eric Hanson
Erik Hanson
Gregory Hanson
Johannes Hanson
Lorelei Hanson
Nora Hanson
Thomas Hanson
Bhakti Hansoti
Jarkko Hantula
Brigitte Hantusch

Mark Hanudel
Adriana Hanulíková
Krishna Prasad Hanumanthappa
Prasada Rao Hanumanthu
Chenyang Hao
Chong-Qing Hao
Han Hao
Lu Hao
Pengying Hao
Shiying Hao
Shuang-Li Hao
Tianyong Hao
Wei Hao
Wenrui Hao
Y. B. Hao
Yang Hao
Yangyang Hao
Zhixin Hao
Alexander Hapfelmeier
Siegfried Hapfelmeier
Denise Happ
Christine Happle
Hapuarachchige Chanditha
Hapuarachchi
Rizwan Ul Haq
Andrea Haqq
Haris Haqqani
Azizul Haque
Mainul Haque
Md. Aminul Haque
Md. Ehtesamul Haque
Shabirul Haque
Shafiul Haque
Sulsal-Ul Haque
Ubydul Haque
Azusa Hara
Hideaki Hara
Manami Hara
Noriko Hara
Takafumi Hara
Takeshi Hara
Alice Harada
Bryan Harada
Hisashi Harada
Mamoru Harada
Naoki Harada
Rie Harada
Kristin Haraldsdottir
Hulda Haraldsdóttir
Guttorm Haraldsen
Tomohiro Harano
Harapan Harapan
Oscar Harari
Simon Harbarth
Matthew Harber
Philip Harber
Tomeka Harbin
Jeremy Harbinson
Alf Harbitz
Poya Hård af Segerstad
Sveinn Hardarson
Karen Hardee
Stuart Hardegree
Rachel Hardeman
Avril Harder
Jens Harder
Timm Harder
Andrew Hardie
William Hardie
Jeffrey Hardin
Eleanor Harding
Juliana M. Harding
Keith Harding
Scott Harding
Shannon Harding
Jenny Hardingham
Tim Hardingham
Kyle Hardman
Jo L. Hardstaff
Laurel Harduar Morano
Anne Harduin-Lepers
Dale Hardy
Giles Hardy
Liselotte Hardy
Louise Hardy
Sheila Hardy
Steve Hardy
Dominic Hare

Gregory Hare
James Hare
Jonathan Hare
Timothy Hare
Roi Harel
Alan Hargens
Levi Hargrove
Stacy Hargrove
Bodduluri Haribabu
Mohie Haridy
Christopher Harig
Nirmala Hariharan
Prasanna Hariharan
Anantha Krishnan Harijith
K. B. Harikumar
Bernhard Haring
Robin Haring
G. Hariprasad
Roopa Hariprasad
Nader Hariri
Anil Kumar Haritash
Kenneth Harkin
John Harkness
Alexandra Harlander-Matauschek
Naomi Harley
Guy Harling
David Harmon
Derek Harmon
Karen Harmon
Thomas C. Harmon
Amy Harms
Peter Harms
Bart Harmsen
Dag Harmsen
Martin Harmsen
H. Harn
Lisa Harnack
Dominique Harnicek
Chris Harnish
Jay Haron
Simon Haroutounian
C. Mark Harper
Christopher Harper
G. Harper
Lorie Harper
Nathan Harper
Thomas Harper
Dörte Harpke
Adrian Harpold
Bettina Harr
Balázs Harrach
Paul Harrell
Trisheena Harricharran
Deirdre Harrington
Kerry Harrington
Laura Harrington
Stephen Harrington
Anette Harris
Caroline Harris
Claire Harris
Courtenay-Jane Harris
David Harris
Edward Harris
Holly Harris
Hugh H. Harris
J. Berton Harris
Janna Harris
Jenine Harris
Kari Harris
Karleah Harris
Kelley Harris
Laurence Harris
Linda Harris
Marion Harris
Mark Harris
Matthew Harris
Michael Harris
Norman Harris
Patrick Harris
Rachel Harris
Rayna Harris
Rebecca Harris
Reuben Harris
Richard Harris
Robert Harris
Ruth Harris
Stephen Harris
Steven Harris

Taylor Harris
Tim Harris
Tony Harris
William Harris
Helen Harris-Fry
Anthony Harrision
Abby Harrison
Andrew Harrison
David Harrison
Glenn W. Harrison
Ian Harrison
Jennifer Harrison
Jonathan Harrison
Laura Harrison
Margo Harrison
Mark Harrison
Neil R. Harrison
Rhett Harrison
Robert Harrison
Steve Harrison
Lisa Harrison-Bernard
Elinor Harrisson
Katie Harron
Clare Harrop
Emily Harrop
Pam Harrop
Elizabeth Harry
Laura Harsan
Jolan Harsfalvi
James Harsh
David Hart
Jo Hart
Joanna Hart
Lynette Hart
Prue Hart
Robbie Hart
Sarah Hart
Trevor Hart
Steven Harte
Tibor Hartel
Andreas Härtel
Volker Hartenstein
Patrick Harter
Klaus Hartfelder
Olaf Hartig
Hanna Hartikainen
Rebekka Harting
Carol Hartley
John Hartley
Rebecca Hartley
Brett Hartman
Nadia Hartman
Sheri Hartman
Alain Hartmann
D. Hartmann
Daniel Hartmann
Daniela Hartmann
Erik Hartmann
Jessica Hartmann
Marcus Hartmann
Petra Hartmann
Rune Hartmann
Tom Hartmann
Sonja Hartnack
Christiane Hartog
Peter Hartsough
Adam Hartstone-Rose
Miriam Hartveit
Matthew Hartwig
H. Hartzell
Bram Hartzema
Megumi Haruna
Yoshiaki Harushima
Ovidiu Hâruta
Yasuo Haruyama
Alan Harvey
Ben Harvey
Brian Harvey
Campbell Harvey
Danielle Harvey
Mark Harvey
Nigel Harvey
Philip Harvey
Daniel Harvie
Sailesh Harwani
Joseph Harwell
Colin Harwood
Lois Harwood

Baktiar Hasan
Fariha Hasan
Irtiza Hasan
Md. Kamrul Hasan
Samiul Hasan
Tayyaba Hasan
Uzma Hasan
Zahra Hasan
Mirza Hasanuzzaman
Ferdinand Haschke
Naoyuki Hasebe
Rie Hasebe
Muhammad Haseeb
Hiroshi Hasegawa
Junichi Hasegawa
Kunihiro Hasegawa
Masahiro Hasegawa
Masami Hasegawa
Midori Hasegawa
Paul Hasegawa
T. Hasegawa
Michael Haselhuhn
Jan Hasenauer
Timothy Hasenoehrl
Karl Hasenstein
Kirstern Haseyama
Mohamed Hashem
Masoud Hashemi
Rowshanak Hashemiyoon
Dana Hashim
Michio Hashimoto
Motomu Hashimoto
Muneaki Hashimoto
Naohiro Hashimoto
Shoji Hashimoto
Takeshi Hashimoto
Tomoki Hashimoto
Hikaru Hashitani
Toshikazu Hashizume
Mohammad Hashmi
Syed Hashsham
Ayshamgul Hasim
Noor Hasima Nagoor
Sinan Hasirlioglu
Marie Haskell
Catherine Haslam
S. Alexander Haslam
Chris Hass
Richard Hass
Ahmed Hassan
Areej Hassan
Bruna Hassan
Elham Hassan
Hosni Hassan
Mahmoud Hassan
Mohamed Hassan
Mohammad Hassan
Ranya Hassan
Reem Hassan
Sahar Hassan
Shahzad Syed Hassan
Syed Hassan
Tamoor Hassan
Tidi Hassan
Waseem Hassan
Wassim Hassan
Mehdi Hassanshahian
Gholamreza Hassanzadeh
David Hassel
Eliza Hasselquist
Esteban Hasson
Y. Hassona
Magdy Hassouna
Hur Hassoy
Michael Hast
Katherine Hastings
Philip Hastings
April Hastwell
Alyssa Hasty
Tomohisa Hasunuma
Akito Hata
Nobuhiro Hata
Shoji Hata
Toshimichi Hata
Ernieda Hatah
Dai Hatakeyama
Kevin Hatala
Etsuro Hatano

Jun Hatazawa
Elizabeth Hatch
Grant Hatch
Maureen Hatch
Abigail Hatcher
Abigail M Hatcher
Patrick Hatcher
Todd Hatchette
Peter Hatemi
Emma Hatfield
Jerry Hatfield
Ronald Hatfield
Timothy Hatfield
Jesse Hatgis
Marcia Hathaway
Erez Hatna
Yuta Hatori
Asma Hatoum
Mathieu Hatt
Taku Hatta
Waku Hatta
Theresa Hattenrath
John Hattersley
Maximilian Hatting
Bart Hattink
Gabriel Hattori
Naoko Hattori
Tsuyoshi Hattori
István Gábor Hatvan
Emmanuel Hatzakis
Mechthild Hatzfeld
Georgia Hatzivassiliou
Maria Hatzoglou
Julia Hatzold
Karin Hatzold
Stavros Hatzopoulos
Dietrich Haubenberger
Christina Haubrich
Ruediger Hauck
Stefanie Hauck
Dominik Haudenschild
Martin Hauer-Jensen
Alexander Tobias Haug
Jon Haug
Håkon Haugaa
Kristina Haugaa
Asger M. Haugaard
Ellen Hauge
Arvid Haugen
Thomas Haugen
Alison Haughton
Jason Haukoos
Markus W. Haun
Robin Haunschild
Steffen Hauptmann
Narayan Haurasiya
Michael Hauschild
Jeffrey Hausdorff
Ben Hause
Felix Hauser
Michael Hauser
Peter Hauser
Christine Hauskeller
Dorothy Hausman
Anna Hausmann
Axel Hausmann
Niklas Hausmann
Georg Hausner
Thomas Hausner
Bettina Haussmann
Stefanie Haustein
Jarkko Hautala
Denis Hauw
Mária Havašová
John Havel
Emmanuel Haven
Joaquim M. Havens
Robert Havey
Jan Havlicek
Shlomo Havlin
Stepan Havranek
James Hawdon
Joseph Hawes
Stephen Hawes
Nael Hawi
Ljac Hawinkels
Steven Hawken
David Hawkes

Michael Hawkes
Alexander T. Hawkins
Amy J. Hawkins
Anthony Hawkins
Brent Hawkins
Christopher Hawkins
Clare Hawkins
Claudia Hawkins
Guy Hawkins
Meredith Hawkins
Stephen Hawkins
Andrea Hawkins-Daarud
Adrian Hawley
Bob Hawley
Nicola Hawley
Kristina Haworth
Claudie Haxaire
Coline Haxaire
Bruce Hay
Doug Hay
Eric Hay
John Hay
Phillipa Hay
Yoichi Hayakawa
Kohsuke Hayamizu
Ryugo Hayano
Abdallah Hayar
Atsushi Hayashi
Kanako Hayashi
Kaori Hayashi
Kazohiko Hayashi
Kei Hayashi
Mirian Hayashi
Shigehiko Hayashi
Shigeo Hayashi
Shin-Ichi Hayashi
Shinichiro Hayashi
Toshio Hayashi
Yukako Hayashi
Yukiko Hayashi
Morihiro Hayashida
Tomoko Hayashida
Kathleen Hayden
Salim Hayek
Andrew Hayen
Heiko Hayen
Juliette Hayer
Manajit Hayer-Hartl
Alan Hayes
Alison Hayes
Christopher T. Hayes
Daniel F. Hayes
Dexter Hayes
Doug Hayes
Galina Hayes
Jerrard Hayes
John Hayes
Kelly Hayes
Lawrence Hayes
Marie Hayes
Patrick Hayes
Richard Hayes
Scott Hayes
Graham Haylor
Laura Hayman
Jean-Philippe Haymann
Morey Haymond
Alex Haynes
Kathleen Haynes
Laura Haynes
Winston Haynes
Sohan Hayreh
William Hayton
Adam Hayward
Bruce Hayward
Catherine Hayward
Nicholas Hayward
Steven Hayward
Shoichi Hazama
Florette Hazard
Mehdi Hazari
Anupama Hazarika
Manjil Hazarika
Surovi Hazarika
Kaoru Hazeki
Jon Hazeldine
Leslie Hazell
Eric Hazelton

William Hazelton
Kevin Hazen
Lori Hazlehurst
David Hazlerigg
Rohan Hazra
Hossein Hazrati
Aibin He
Angela Xiaoxue He
Bin He
Chongbo He
Daihai He
Dalin He
Daqing He
Debiao He
Dongsheng He
Fei He
Feng He
Fenglei He
Guoguang He
Haibin He
Hongbo He
Hong-Di He
Jiangping He
Jiankui He
Ji-Huan He
Jin He
Jinbo He
Jing He
Jingrui He
Jinhan He
Jin-Sheng He
Jun He
Junxian He
Kaiyu He
Lin He
Long He
Maoxian He
Miao He
Mingzhu He
Qiang He
Qing He
Qiushui He
Rui He
Shaozhen He
Shuangba He
Songqing He
Tao He
Tianyu He
Wei He
Weilue He
Wei-Qi He
Wenqian He
Wenrong He
Xiang He
Xiaoming He
Ximiao He
Xingshi He
Xuesong He
Yajun He
Yan He
Yanling He
Yiping He
Yong He
Yonghan He
Yueping He
Yungang He
Zangdong He
Zhe He
Zhi He
Zhonghu He
Zhongming He
Zhuohao He
Steven Head
Stewart Head
Simon Header
Derek Headey
Christopher Healey
Sean Healey
Genevieve Healy
Kristen Healy
Rosaria Healy
Alexandra Heaney
Marshall Heap
Christopher Heaphy
Stephen Heard
Kristine Hearld
Vanessa Hearnden
Tamara Heartsill

Caroline Heary
Daniel Heath
Gregory Heath
Lenwood Heath
Paul Heath
Sonya Heath
James Heaton
Lisa Heaton
Robert Heaton
Scott Hebbring
Frank Heberling
James Hebert
Lee Hebert
Sébastien Hébert
Kim Hébert-Losier
Antje Hebestreit
Clair Hebron
Silke Hechinger
Jory Hecht
Michael Hecht
Daniel W. Heck
Nicolas Heck
David Heckel
Andrew Heckert
Carolyn Heckman
Jesse Heckman
Robert Heckman
Anne Hecksteden
Mark Hector
Stefanie Hectors
Mehdi Hedayati
Nosratollah Hedayatpour
Craig Hedberg
Salim Heddam
C. Hedegaard
Mette Hedemann
Peter Hedera
Sandeep Hedgire
Richard Hedley
Johannes Hedman
Rainer Hedrich
Sabrina Hedrich
Ann Hedrick
Brandon Hedrick
Sarah Hedtrich
Alvin Kah-Wei Hee
Philipp Heeb
Tobias Heed
Hannelore Heemers
Adel Heenan
Paul Heerdt
Alexandre Heeren
Ashley Heers
David Heery
Kristiann Heesch
Abraham Hefetz
Neil Heffernan
Petra Heffeter
Renee Heffron
Katrin Hefler-Frischmuth
Joseph Hefner
Ahmed Hefny
Kelsey Hegarty
Matthew Hegarty
Rehab Hegazy
Yamen Hegazy
Mahabaleshwar Hegde
Pushpa Hegde
Shrilakshmi Hegde
Shubhada Hegde
Adriana Hegedus
Tamás Hegedűs
Ute Hegenbart
Michal Heger
Ulrike Heger
Ulrich Hegerl
Colleen Hegg
Daniel Hegglin
K. W. Hegland
Robert Hegna
Reinhard Hehl
Eric Hehman
Tom Hei
Iris Heid
Bahram Heidari
Behzad Heidari
Mohammad Heidarinejad
Warren Heideman

Rebecca Heidkamp
Timo Heidt
Peter Heiduschka
Christoph Heier
Irene Heijink
Anders Heijl
Jordi Heijman
Cobi Heijnen
Michel Heijnen
Ahmed Heikal
John Heiker
Oskari Heikinheimo
Katriina Heikkila
Jeanne Heil
Martin Heil
Leonie Heilbronn
Lance Heilbrun
C. Heilig
Albert Heim
Ronald Heimark
Harry Heimberg
Thomas Heimburg
Douglas Heimburger
J. Heimel
Robert Heimer
David Hein
Irma Hein
Jerry Heindel
Bjo¨Rn Heindryckx
Markus Heine
Martin Heine
Lutz Heinemann
Torsten Heinemann
Udo Heinemann
Joel Heinen
Sara Heinert
Kujala Heini
Stefan Heinl
Krista Heinonen
Anje Heinrich
Joachim Heinrich
Katie Heinrich
Mattias P. Heinrich
Jud Heinrichs
Elizabeth Heinrichs-Graham
Bradley Heins
Rainer Heintzmann
Adrienne Heinz
Werner Heinz
Jürgen Heinze
Katharina Heinzelmann
Robert Heinzen
Daniel Heise
Verena Heise
Kazuki Heishima
Michele Heisler
Michelle Heisler
Christian Heiss
Gerardo Heiss
Jeremy Heit
John Heit
Marlène C. H. Hekman
Sophie Helaine
Harri Helajärvi
Ilkka Helanterä
Karla Helbig
Manuel Helbig
Fabian Held
Jason Held
Kathryn Held
Coy Heldermon
Helena Helena Rabie
Camie Heleski
Tobias Helfen
Fabrice Helfenstein
Véronique Helfer
Simon Helfgott
Valeska Helfinger
Kristofer Helgen
Frederik Helgstrand
Hossein Heli
Moritz Helias
Sébastien Hélie
Tomáš Helikar
Diane Heliker
Ritva Heljasvaara
Dan Hellberg
Francois Helle

Karen Helle
Christopher Hellen
Daliah Heller
Nicole Heller
Richard Heller
Stefan Heller
Stephane Helleringer
Susan Hellerstein
Adam Helles
Jon Hellin
Britney Helling
Emily Helliwell
Jens Hellmann
Marcin Hellmann
Jacklyn Hellwege
Kerstin Hellwig
Nathan Hellyer
Bryan Helm
Rebecca Helm
Robert Helm
Karl Helmer
Terhi Helminen
Dominique Helmlinger
Jill Helms
My N. Helms
Volkhard Helms
Brian Helmuth
Yosra Helmy
Claudia Helou
Liat Helpman
Frederik Helsen
Werner Helsen
Jeffrey Helton
Otto Helve
Nathaniel Helwig
Joris Hemelaar
Katie Hemer
Robert Hemke
Farhid Hemmatzadeh
Bernhard Hemmer
Moritz Hempen
Andrew Hemphill
Tessa Hempson
Corinne Henak
Marcela Henao-Tamayo
Yann Henaut
Yves Henchoz
Marloes Henckens
Brad D. Hendershot
Amanda Henderson
Andrew Henderson
Beric Henderson
Cassandra E. Henderson
Claire Henderson
Elizabeth Henderson
Gail Henderson
Graeme Henderson
Gregg Henderson
Janet Henderson
Karla Henderson
Neil Henderson
Paul Henderson
Victor Henderson
Arun Hendi
Nathan Hendricks
Friederike Hendriks
Marja Liisa Hendriks
Rudolf Hendriks
Niels Bohse Hendriksen
Peter Hendriksen
Harmen Hendriksma
David Hendrix
Nathaniel Hendrix
Nicola Heneghan
Hong-Qiang Heng
Zhang Heng
Ulrich Hengst
Li Hengzhe
Martin Heni
Sigrun Henjum
Oliver Henke
Peter Henke
John Henley
William Henley
Marie Hennebelle
Julie Hennegan
Peter Henneman
Christophe Hennequin

Erin Hennes
Ian Hennessee
Annemarie Hennessy
Dwight Hennessy
Ellis Hennessy
Timo Hennig
Stephen Hennigar
Marcus Henning
Susan Henning
Heath Henninger
Nils Henninger
Anna Henningsson
Mark Hennon
Eileen Hennrikus
Michael Henrich
Lena Henriksen
Hanna Henriksson
Alexandre Henriques
Catarina Henriques
Joana Henriques
Romina Henriques
Sofia Henriques
Flavio Henrique-Silva
Oswaldo Henriquez
Juan Pablo Henríquez
Bernard Henrissat
Charles Henry
Chuck Henry
Clarissa Henry
Doug Henry
Hugh Henry
Kenneth Henry
Kevin Henry
Laurence Henry
Mary Henry
Michael Henry
Olivier Henry
Robert Henry
Stephen Henry
Yves Henry
Devon Hensel
Goetz Hensel
Yvonne Henskens
Michael Henson
Stephanie Henson
Ian Hentall
Benjamin Henwood
Dietrich Henzler
Hye-Young Heo
Jeong Heo
Ji Hoe Heo
Nilay Hepgul
Jeff Hepinstall
Luiz U. Hepp
Thomas J. Heppner
Stan Heptinstall
Matthew Hepworth
Delphine Héquet
Chandana Herath
Catherine Herba
Joshua Herbeck
Radu Herbei
F. A. Herbella
Debby Herbenick
Samuel Herberg
Holly B. Herberman Mash
Marie Herberstein
Amanda Herbert
Cristan Herbert
James Herbert-Read
Alexander Herbig
Peter Herbison
Bruce Herbold
Maikel Herbrink
Andreas Herbst
Sophie Herbst
Melissa Herbst-Kralovetz
Michael Herce
David Hercher
Trent Herda
Maria Teresa Herdeiro
Piet Herdewijn
Anthony Herdman
M. Herdman
Michael Herdman
Roberto Heredia
Levente Herenyi
S. Herese

Tamila Heresztyn
Safa Herfat
Kristina Herfert
Alan Herford
Melinda Hergert
Matthias Heringlake
Nicolae Herisanu
Kathleen Herkommer
P. W. Herlina
James Herman
Lule Herman
Michael Herman
P. M. Herman
Patricia Herman
Pawel Herman
Péter Hermán
Anna Herman-Antosiewicz
Brian Hermann
Sara Hermann
Norbert Hermanns
Are Hermansen
Robert Herman-Smith
Jean-Michel Hermel
Dora Hermes
Gerben Hermes
Priscila Hermida
John Hermos
Sabrina Hermosilla
Joachin Hermsdorfer
Pablo Hernáiz Driever
Imma Hernan
Thiago Rocha Hernandes
Alexis Hernandez
Arturo E. Hernandez
Brenda Hernandez
Christopher Hernandez
Emili Hernandez
Gonzalo Hernandez
Hector M. Hernandez
Isabel Hernandez
Joselin Hernandez
Laura Hernandez
Luis Hernandez
Marta Hernandez
Mauro Hernandez
Oscar Hernandez
Malva Hernández
María Aurora Armienta Hernández
Maria-Angeles Hernández
Moisés Hernández
Lorna Hernandez Santin
Roberto Hernandez-Alejandro
Alina Hernandez-Bark
Juan Hernández-Barrios
Angel A. Hernández-Borges
Carlos Hernandez-Castillo
John Hernandez-Castro
Juan Hernandez-Fernaud
Ana Hernández-Hernández
Daniel Hernandez-Huerta
Luis Hernández-Ibarra
Aurora Hernández-Machado
Pablo Hernández-Marrero
Salvador Hernandez-Martinez
Salvador Hernández-Martínez
Cesar Hernandez-Rodriguez
Maria Hernandez-Valladares
Esteban Hernandez-Vargas
Hector Hernandez-Vargas
José L. Hernández-Verdejo
Roberto Hernan-Gonzalez
Dennis Hernaus
Leon Herndon
Michel Hernould
Alfred Hero
Morgan Herod
Nikolas Herold
Martin Héroux
Deron Herr
R. Herráez-Hernández
Daniel Herranz
Anthony Herren
Antonio Herrera
Bernardo Herrera
Carolina Herrera
Juan Herrera
Manuel Herrera
L. Gerardo Herrera M.

Maria Isabel Herrera-Montes
José Herrero
Lara Herrero
Miguel Herrero
Gabriel Herrero-Beaumont
John Herring
David Herrington
William Herrington
Harald Herrmann
Matthew Herron
Karl Herrup
Christophe Herry
Kurt Hersberger
Jason Herschkowitz
Peter Hersey
Eitan Hersh
Alexendra Hershberger
Carlton Hershner
Mette Herskin
Adam Hersperger
Jens Hertel
Johannes Hertel
Megan Herting
Ingo Hertrich
Stefan Hertwig
Daniel Hertz
Jens Michael Hertz
M. Hertzberg
Alexis Hervais-Adelman
Frederic Hervant
Antonio Hervás
Ramón Hervás
Jactel Hervé
Vincent Hervé
Arnau Hervera
Baptiste Hervier
Daniel Herwartz
Christian Herweh
Genevieve Héry-Arnaud
Osnat Herzberg
Julia Herzen
Mark Herzik
Charlotte Herzmann
Christine Herzog
Erica Herzog
Gregoire Herzog
Harold Herzog
Roland Herzog
Stefan Herzog
O. Hes
Kathryn Hesketh
Cornelius Hess
Jeremy Hess
Jochen Hess
Lisa Hess
Michael Hess
Philip Hess
Yanine Hess
Constanze Hesse
Matthijs Hesselink
Guido Hesselmann
Amanda Hessels
Dag Hessen
Hugo Hesser
Deike Hesse-Wilting
David Hessl
Martin Hessner
Robert Hester
Warren Heston
Matthew Hethcoat
Kasper Hettinga
Attila Hettyey
Rainer Heuchel
Andreas Heuer
Gregory Heuer
Holger Heuer
Klaus Heuner
Martin Heur
Julia Heuritsch
Nicole Heussen
Marjolein Heuvelmans
Henri Heuven
Andrea Hevener
Kirk Hevener
Mark Hew
Tamara Hew-Butler
Angela Hewett
James Hewett

Marion Hewicker-Trautwein
A. J. Mark Hewison
Martin Hewison
Alex Hewitt
Richard Hewitt
Erik Hewlett
Patricia Hewston
Mehdi Heydari
Isabel Heyde
Robert Heyderman
Karolina Heyduk
Leonard Heyerdahl
Dominik Heyers
Jason Heyes
Beate Heym
Gail Heyman
Samuel Heyman
Leo Heyndrickx
Marc Heyndrickx
Frankie Heyward
Wendy Heywood
Natasha Hezelgrave
Yoichi Hiasa
Paul Hibbard
Carina Hibberd
Benjamin Hibbert
David Hibbett
Matthew Hibbing
Michael Hibbing
Margaret Hibbs
Masahiko Hibi
Margaret Hicken
Robyn Hickerson
Anthony J. Hickey
Aodhan Hickey
Clayton Hickey
Lee Hickey
William Hickey
Graham Hickling
Deb Hickman
J. Hickman
James Hickman
Zachary Hickman
Kyle Hickmann
Robert Hickner
Noreen Hickok
Jason Hicks
Lanis Hicks
Richard E. Hicks
Yasuhiro Hida
Eiji Hidaka
Cesar Hidalgo
José Miguel Hidalgo
Julia Hidalgo
Carmen Hidalgo Tenorio
Balázs Hidasi
Mallorie Hide
Bettina Hiemer
Martin Hieronymi
Jari Hietanen
Peter Hietz
Elisa Higa
Yukihito Higashi
Haruhiro Higashida
Norihisa Higashihori
Matt Higger
Gavin Higgins
Kristin Higgins
Lucy Higgins
Andrew Higginson
Jill Higginson
Elizabeth Higgs
Phil Higham
Kate Highfield
Scott Highhouse
James Highsmith
Lawrence Hightower
Lisa Hightow-Weidman
Gustavo Higuera
Diego Hijano
Farrukh Hijaz
Hiroyuki Hikichi
Taiichi Hikichi
Leena Hilakivi-Clarke
David Hilbert
Friederike Hilbert
Martin Hilbert
Benjamin E. Hilbig

Deana Hildebrand
Falk Hildebrand
Michael Hildebrand
Niko Hildebrandt
David Hildeman
Stanley Hileman
Roger Hilfiker
Joseph Hilgard
Ingo Hilgendorf
Ralf-Dieter Hilgers
Monika Hilker
Andrew Hill
April Hill
B. J. Hill
Bradford Hill
Catherine Hill
David B Hill
David Hill
Elisa Hill
Emmeline Hill
James Hill
Jessica Hill
Kathleen Hill
Matt Hill
Matthew Hill
Richard Hill
Russell Hill
Suzanne Hill
Terence Hill
Tessa Hill
Uta Hill
Wendy Hill
Kathleen Hill Gallant
Georgios Hillas
Russ Hille
Andreas Hillenbrand
John Hillier
Susan Hillier
Sophie Hillion
Frederique Hilliou
Argye Hillis
Susan Hillis
Kristin Hillman
Magnus Hillman
Noah Hillman
Falk Hillmann
Claire Hills
Danny Hills
Nancy Hills
Ronald Hills
Susan Hills
Thomas Hills
Nathan Hillson
Julian Hillyer
Sarah Hilmer
Robert Hilsden
Susan Hilsenbeck
Peter Hilsenrath
Lara Hilton
Zoë Hilton
Markus Hilty
Max Hilz
Tommi Himberg
Gary Hime
Seiichiro Himeno
Wolfgang Himmel
Heinz Himmelbauer
David Himmelgreen
Hubertus Himmerich
Thomas Hinault
Charlotte Hinault-Boyer
Doracelly Hincapie
Dirk Hincha
Alan Hinderliter
Sajedah Hindi
Rikkert Hindriks
Pieter Hindryckx
David Hinds
Andrew Hines
Brooke Hines
O. Hines
Stephanie Hing
A. Hiniker
Rheyda Hinlo
Jason Hinman
Takao Hinoi
Silvia Hinojosa
Gustavo Hinojosa Arango

Peter Hinow
Jan Hinrichs
David Hinton
Joseph Hinton
Kerry Hinton
Henning Hintzsche
Rainer Hinz
Sebastian Hinz
Lori Hinze
David Hipgrave
Noriyuki Hirahara
Daniel Hirai
Hiroki Hirai
Osamu Hiraike
Hidehiko Hirakawa
Hiroshi Hirakawa
Yoshihisa Hirakawa
Jade Hiramoto
Kajal Hirani
Shigeru Hirano
Yoji Hirano
Yukinori Hirano
Pornpan Hiranpradit
Hiroyuki Hirasawa
Tadashi Hirasawa
Takashi Hirasawa
Hajime Hirase
Shotaro Hirase
Akihiro Hirashiki
Shintaro Hirata
Yasuhisa Hirata
Yoshito Hirata
Nobuhito Hirawa
Yoshitaka Hirayama
Jibril Hirbo
Sarah Hird
Swapnil Hiremath
Noboru Hiroi
Keiju Hiromura
Keiichi Hirono
H. Hirooka
Kazuyuki Hirooka
Yoshitaka Hirooka
Kiichi Hirota
Simon Hirota
Masako Hirotani
Alec Hirsch
Annemarie Hirsch
Candice Hirsch
Christian Hirsch
Elizabeth Hirsch
Gary Hirsch
Joy Hirsch
Lawrence Hirsch
Oliver Hirsch
Gilad Hirschberger
Jon Hirshon
Kathy Hirsh-Pasek
Andrew Hirst
Claire Hirst
Myriam R. Hirt
Robert Hirt
Frank Hirth
Kalle Hirvonen
Toru Hisabori
Shin-Ichi Hisanaga
Koji Hisatake
Harriet Hiscock
Alexandra Hiscox
Tomohito Hishikawa
Christophe Hissler
Francisco Hita Garcia
Geeta Hitch
Peter Hitchcock
Paul Hitchcott
Jessica Hite
Mary Hitt
Chris Hittinger
V. Hiuzar
Jacob Hjelmborg
Mathis Hjelmsø
Jesper Hjortdal
Carsten Hjorthoej
Tim Hla
Wolfgang Hladik
Myron Hlynka
Amic Ho
Andrew Ho

Brian Ho
Cheng-Maw Ho
Chen-Hsun Ho
Chi-Lai Ho
Ching-Sung Ho
Chuan-Kai Ho
Derek Ho
Emily Ho
Harvey Ho
John W. S. Ho
Mengfei Ho
Ming-Chih Ho
Shin-Lon Ho
Shinn-Ying Ho
Sunita Ho
Thien Ho
Tiffany Ho
Tuan-Hua Ho
Vincent Ho
Walter Ho
Yuh-Shan Ho
Katherine Hoadley
Nhat-Duc Hoang
Quyen Hoang
Erin Hoare
Anders Hobæk
Marcia Hobbs
Robin Hobbs
Ryan Hobbs
William Hobbs
Alistair Hobday
Gerlinde Höbel
Charles Hobson
Keith A. Hobson
Jody Hobson-Peters
Roman Hobza
Peter Hoch
Jacob Hochard
Natasha Hochberg
Alexander Hochdorn
Berthold Hocher
Ute Hochgeschwender
Edith Hochhauser
Alejandro Hochkoeppler
Gal Hochman
Guy Hochman
Zvi Hochman
George Hochmuth
Sandra Hochscheid
Mark Hochstrasser
Andreas Hochwagen
Barry Hock
Philip Hockberger
Adam Hockenberry
Joel Hockensmith
Darren Hocking
David P. Hocking
Samantha Hocking
Marc Hockings
Martina Höckner
Jean-Francois Hocquette
John Hoddinott
Christopher Hodge
Sarah Hodge
Jo Hodgekins
Matthew Hodges
Carolyn Hodges-Simeon
Jamie Hodgkins
James Hodgkinson
Abraham Hodgson
David Hodgson
Kelly Hodgson
Chris Hodkinson
Claire Hodson
Mark Hodson
Martin Hodson
Peter Hodson
C. W. Hoedemaekers
Julia Hoefer
Guenter Hoeglinger
Barbara Hoehle
Mathias Hoehn
Thomas Hoehn
Wieteke Hoeijmakers
Gerard Hoek
Janet Hoek
Berthold Hoelldobler
Timothy Hoellein

Sarah Hoelscher
Ludwig Hoelzle
Caroline Hoemann
Markus Hoenicka
Martin Hoenigl
Dirk Hoeper
Lori A. Hoepner
Luke Hoeppner
Michael Hoerger
Stephane Hoerle
André Hoerning
Marisa Hoeschele
Konrad Hoetzenecker
Andrew Hoey
Anouschka Hof
At Hof
Benedikt Hofauer
Gunther Hofbauer
Angelika Hofer
Geir Hoff
Peter Hoff
Kenneth Hoffer
Barbara Hoffman
Gabriel Hoffman
Geoffrey Hoffman
Irving Hoffman
Joel Hoffman
Joseph Hoffman
Lindsay Hoffman
M. Hoffman
Martin Hoffman
Paul Hoffman
Rene Hoffman
Rhonda Hoffman
Robert Hoffman
Stanley Hoffman
William Hoffman
Adrian Hoffmann
Andreas Hoffmann
Ary Hoffmann
Boris Hoffmann
Celine Hoffmann
Christian Hoffmann
Dirk Hoffmann
Donata Hoffmann
Frauke Hoffmann
Hanne Hoffmann
Janine Hoffmann
Johannes J. M. L. Hoffmann
Markus Hoffmann
Robert Hoffmann
Sandra Hoffmann
Stefan Hoffmann
Anna-Maria Hoffmann-Vold
Cynthia Hoffner
Ulrich Hoffrage
Florian Hofhansl
Johan Hofkens
Johannes Hofland
Florence Hofman
Sebastian Hofman
Winni F. Hofman
Alexander Hofmann
Franz Hofmann
Maike Hofmann
Richard Hofmann
Volker Hofmann
Marion Hofmann Bowman
Tim Hofmeester
Wayne Hofstetter
Robert Hofstra
Herman Hofte
Ellizabeth Hogan
Harry Hogan
Mary-Ellen Hogan
Natacha Hogan
Simon Hogan
Lee Hogarth
Claus Høgdall
Michel Hogenes
John Hogenesch
Eef Hogervorst
Hans Hogerzeil
Paulien Hogeweg
Claire Hogg
Katherine J. Hoggatt
Cerissa Hoglander
Wolfgang Högler

Jacob Hoglund
Richard Hoglund
Marieann Hogman
Jens Högström
Brenda Hogue
Ron Hoham
Stefan Hohaus
Christian Hohenstein
Jörg Höhfeld
Corinne Hohl
Johannes Hohlbein
Gottfried Hohmann
Stefan Hohmann
Martin Hohmann-Marriott
Aleta Hohn
Lars Øivind Høiseth
Himan Hojat Jalali
Ole Hojberg
Aki Hoji
Akira Hokama
Jessica Hokamp
Jo Høkedal
Akishige Hokugo
Wim Hol
Matthew Holahan
V. Holan
John Holbein
Amber Holbrook
Beth Holbrook
Joseph Holbrook
Luke Holbrook
Michael Holbrook
David Holcman
Randall Holcombe
Mateusz Hołda
Arun Holden
John T. Holden
Karen Holden
Stein Holden
Benjamin Holder
Beth Holder
Mark Holder
Mary Holder
Chris Holderman
Ricardo Holdo
Thao Ho-Le
Jan Holeksa
Theodore Holford
Francisco Holguin
Nilsson Holguin
Michael Holick
Toril Holien
Ninoslav Holjevac
Linda Holland
Maggie Holland
Martin Holland
Nicholas Holland
Peter Holland
William Holland
John Hollander
Kristen Hollands
Thomas Hollemann
Andrew Hollenbach
Jill Hollenbach
Peter Hollenbeck
Yvonne Höller
Charvonne Holliday
Gemma Holliday
Shannon Holliday
Bruce Hollis
Chris Hollis
M. E. Hollmen
Gábor Holló
Kurt Hollocher
Alison Holloway
Graham Holloway
Jeremy Holloway
Alfreda Holloway-Beth
Edward Hollox
Jan Holly
Charlotte Holm
Johanna Holm
Lars-Erik Holm
Niels Bjerre Holm
Soren Holm
Devin Holman
Scott Holmberg
Thomas Holme

Hampus Holmer
Emma Holmes
Gregory Holmes
Jacinta Holmes
Jonathan M. Holmes
Mark Holmes
Arne Holmgren
Yurij Holovatch
Jayna Holroyd-Leduc
R. M. Damian Holsinger
Stephanie Holst
Thomas Holstein
Martin Holt
Natalie Holt
Robert Holt
Stephen Holt
Tim Holt
Harald Holte
Jan Holter
Philipp Holter
Andreas Holtermann
Silva Holtfreter
Knut Holthoff
Derald Holtkamp
Avery Holton
Tom Holtz
Nick Holtzman
Jana Holubcikova
Inga Holube
Paul Holvoet
David A. Holway
Olaf Holz
Gerhard Holzapfel
Simon Holzapfel
Michael Holzel
Michaela Holzem
Andreas Holzinger
Dana Holzinger
A. Holzkaemper
Iris Holzleitner
Nusrat Homaira
Philipp Homan
Dominique Homberger
Akira Homma
Kazuaki Homma
Susanne Homolka
Rami Homsi
Jaco Homsy
Ellis Hon
Akira Honda
Kohsuke Honda
Shigeru Honda
Trenton Honda
Robert Hondal
Hubert Hondermarck
David Hondula
Thomas Hone
Jonathan Honegger
Rodney Honeycutt
Peter Honeyman
Jordi Honey-Rosés
Chen Hong
Chien-Chong Hong
Chientai Hong
Chun-Ming Hong
Chwan-Yang Hong
Daojun Hong
Huifen Hong
Hyun Ju Hong
Jae Taek Hong
Jae-Young Hong
Jin Tae Hong
Jinkee Hong
Jong Ha Hong
Jun Hong
Keun-Sik Hong
Mee Young Hong
Miao Hong
Robert Hong
Sang-Bum Hong
Seok Jun Hong
Shuangsong Hong
Xinru Hong
Yang Hong
Yeong Ho Hong
Yoonki Hong
Zhiyong Hong
Zhou Hong

Bo Hønge
Simone Honikman
Juliane Honisch
Ken Honjo
Julia Honneffer
Patrick Honore
Stéphane Honore
Jean Honorio
Nildimar Honorio
Raquel Hontecillas
Jan Hontelez
Anna Hood
Julia Hood
Wendy Hood
Bart Hoogenboom
Jacob Hoogenboom
Michelle Hoogenhout
Rudolf Hoogenveen
Jurian Hoogewerff
Wouter Hoogkamer
Carlijn Hooijmans
Tomasdr Höök
Angelo Hooker
Shelley Hooks
Edward Hookway
Mark Hoon
Cornelia Hooper
D. Craig Hooper
Marcus Hooper
Ewout Hoorn
David Hoos
Stephen Hooser
Babak Hooshmand
Steven Hooton
Dallas Hoover
Donald Hoover
Edward Hoover
Kelli Hoover
Andrew G. Hope
Steven Hope
Sarah Hope Lincoln
Philip Hopewell
Lan T. Ho-Pham
Merle Höpken
Jacob Hopkins
Kristine Hopkins
Melanie Hopkins
Richard Hopkins
Will Hopkins
Sarah Hopp
Matthias Hoppe
Trevor Hoppe
Lydia Hopper
Jessica Hoppstädter
Mohammad Mainul Hoque
Ricardo Hora
Micha Horacek
Yasushi Horai
Peeter Hõrak
Michael Horberg
Brenton Hordacre
Joost Hordijk
Peter Hordijk
Tal Hörer
Dell Horey
Ai Hori
Chiaki Hori
Hiroki Hori
Kiyosumi Hori
Masatoshi Hori
Yusuke Hori
Yuta Hori
Masayuki Horie
Rie Horie
V. Horigan
Arata Horii
Tomoyasu Horikawa
Nobuo Horikoshi
Daniel Horinek
Takeshi Horio
Nobuyuki Horita
Hisanori Horiuchi
Keisuke Horiuchi
Takahiko Horiuchi
Pavel Horky
Sahand Hormoz
David Hormuth
David Horn

Harald Horn
Patrick Horn
Paul Horn
John Hornberger
David Horne
Mary Horne
Rosemary Horne
Jennifer Horney
Gwo-Jiun Horng
Yu-Tze Horng
Claudia Horn-Hoffmann
Amnon Horovitz
Dafne Horovitz
Alice Horowitz
John Horowitz
Michal Horowitz
Julia Horrocks
Tor Horsberg
Karen Horsburgh
Robert Horsburgh
Alex Horsley
William Horsnell
Jeremy Horst
Olaf Horstick
Bernardo Horta
Marco Horta
Joaquín Hortal
Ruud Hortensius
Marcus Hortmann
Tibor Hortobagyi
Tibor Hortobágyi
John Horton
Jonathan Horton
Julie Horton
Simon Horton
Susan Horton
Robert Horvath
Tamas Horvath
Gergely Horváth
Marcela Horvitz Lennon
Leora Horwitz
Danielle Horyniak
Miroslava Horynová
Ramegowda Hosahally
Venkategowda
Sean Hosein
Sybil Hosek
Naoto Hoshi
Rosangela Akemi Hoshi
Takashi Hoshiba
Junichi Hoshino
Tomonori Hoshino
Yoichiro Hoshino
Annmarie Hosie
Bernhard Hosius
Sherria Hoskins
Paul Hoskisson
Peter Hosner
Manar Hosny
Misa Hosoe
Nobuko Hosokawa
Shuhei Hosomi
Ahmed Hossain
Faisal Hossain
Firoz Hossain
Iqbal Hossain
Mohammad Hossain
Muhammad Hossain
Yeamin Hossain
Zahid Hossain
Syed Hossainy
Seyed Jalal Hosseinimehr
Seyyed-Mohammadreza
Hosseininasab
Leila Hosseinzadeh
Saeid Hosseinzadeh
Abasalt Hosseinzadeh Colagar
David Hostler
Morten Hostrup
Dechamma Hosur
Ramakrishna Hosur
Swetansu Hota
Cara Hotchkin
Richard Hotchkiss
Ibrahim Hoteit
Thilo Hotfiel
Kreshnik Hoti

Benoît Ho-Tin-Noé
Hak Hotta
Maria Hötzel
Bob Hou
Deyi Hou
Dianyun Hou
Hongying Hou
Jian Hou
Jianghui Hou
Lin Hou
Maolin Hou
Maomao Hou
Peng Hou
Shaoping Hou
Sheng Hou
Shengping Hou
Wei Hou
Wen-Hsuan Hou
Wensheng Hou
Xiangyang Hou
Xinguo Hou
Youming Hou
Yuxia Hou
Zhaoyuan Hou
Zhiyuan Hou
Eline Houben
Marlies Houben
Michael Houbraken
Cécilia Houdelier
Han Houdijk
Josselin Houenou
Karin Hougaard
Peter Houghton
Peter Houk
Annwyne Houldsworth
Sherilyn Houle
Joseph Houmard
Laetitia Houot
Eric Houpt
Katherine Houpt
James Houran
Walid Houry
Benjamin Housden
John House
Michael House
Reniqua House
J. E. Householder
Gregory Houseman
Tambri Housen
Alexandra Houssaye
Brian Houston
Charles Houston
Denise Houston
J. Brian Houston
James Houston
Carl Houtman
Jan Hovanec
Michael Hove
Rachel Hovel
Melbourne Hovell
Nadine Hövelmeyer
Jason Hoverman
Daniel Hovey
Tapani Hovi
Torre Hovick
Jeffery Hovis
Charla Howard
Christina Howard
Eric Howard
Jonathan Howard
Louise Howard
Matt Howard
Scarlett Howard
Catherine Howarth
Dianella Howarth
Gordon Howarth
Jennifer Howcroft
Alan Howe
Anita Howe
Cheryl Howe
Andrew J. Howell
Brian Howell
David Howell
Gareth Howell
Kate Howell
Michael Howell
Roger Howell
Cindy Howells

Ella Howes
R. Nicole Howie
Brooke Howitt
Mohammad Sajid Howlader
Brett Howland
Philip Howles
Jonathan Howlett
Robert Howlin
Andrew Hoy
Dirk Hoyer
Zsófia Hoyk
Cathrine Hoyo
Richard Hoyos
Joseph Hoyt
Peter Hoyt
Heba Hozyen
Jaroslav Hrabak
Thomas Hrabik
Bronwyn Hradsky
Alexander Hramov
Susan Hrapcak
Tomas Hrbek
Paulo Hrihorowitsch Moreno
Adriana Hristea
Desislava Hristova
Mariya Hristova
Carrie Hritz
Richard Hrivnak
Victor Hrkac
Maria Hrozanova
Ralph Hruban
Keith Hruska
Jana Hruskova
Paul Hruz
Krzysztof Hryniewicz
Connie Hsia
Allen Hsiao
Amber Hsiao
Ansel Hsiao
Chun-Yuan Hsiao
Elaine Hsiao
Hui-Hua Hsiao
Jong-Kai Hsiao
Kuei-Yang Hsiao
Li-Li Hsiao
Nei-Yuan Hsiao
Victoria Hsiao
W. L. Wendy Hsiao
Yaling Hsiao
Yi-Han Hsiao
Cheng-Yang Hsieh
Ching-Hua Hsieh
Ching-Lin Hsieh
Chiu-Lan Hsieh
Chiwan Hsieh
Chiying Hsieh
I-Chang Hsieh
Meng-Hsuan Hsieh
Michael Hsieh
Po-Shiuan Hsieh
Ru-Lan Hsieh
Sen-Yung Hsieh
Shi-Tong Hsieh
Shun-Chieh Hsieh
Teng-Fu Hsieh
Yi-Chen Hsieh
Yi-Fang Hsieh
Yu-Hsiang Hsieh
Yu-Wei Hsieh
Lisa Hsin
Wang Hsiu-Feng
Bor-Kai Hsiung
Annie Hsu
Bang-Gee Hsu
Cheng-Lung Hsu
Chia-Lin Hsu
Chih-Chien Hsu
Chih-Chung Hsu
Chih-Hung Hsu
Ching-Sheng Hsu
Chung-Yao Hsu
David Hsu
Han-Shui Hsu
Hui-Chuan Hsu
Jason C. Hsu
Joe Hsu
Jue-Liang Hsu
Jung-Yu Hsu

Kean J. Hsu
Lewis Hsu
Li-Chi Hsu
Ming-Lun Hsu
Pang-Hung Hsu
Peter Hsu
Ping-Ching Hsu
Ping-I Hsu
Shen-Mou Hsu
Steven Hsu
Wei-Chung Hsu
Wen-Lian Hsu
Yao-Chun Hsu
Yau Heiu Hsu
Ya-Wen Hsu
Yi-Chiang Hsu
Yu-Hsun Hsu
Yu-Yin Hsu
Yi-Jen Hsueh
Yuan-Yu Hsueh
Tai-Chiu Hsung
Nay Htun
Bang-Chuan Hu
Bin Hu
Bo Hu
Changwei Hu
Chaur-Jong Hu
Che-Ming Hu
Cheng Hu
Cheng-Jun Hu
Chenhui Hu
Chun Hu
Chung-Yi Hu
Dan Hu
Dan-Ning Hu
Dongcai Hu
Emily Hu
Fengjiao Hu
Fupin Hu
Guochang Hu
Guoqiang Hu
Gwo-Chi Hu
Hai-Yang Hu
Han Hu
Han-Chung Hu
Han-Hwa Hu
Hao Hu
Hongli Hu
Howard Hu
Huanhuan Hu
Hui Hu
Huirong Hu
Jia Hu
Jiafen Hu
Jiafu Hu
Jian Hu
Jiang Hu
Jian-Kun Hu
Jianxing Hu
Jianzhong Hu
Jie Hu
Jiehui Hu
Jiming Hu
Jing Hu
Junguo Hu
Junjie Hu
Junmei Hu
Kaijin Hu
Ke Hu
Li Hu
Liang Hu
Longhua Hu
Lu Hu
Mao-Bin Hu
Min Hu
Ming Chang Hu
Ming Hu
Peisong Hu
Ping Hu
Po Hu
Qian-Nan Hu
Qinghua Hu
Qingwei Hu
Rongbin Hu
Rongrong Hu
Rui Hu
Rui-Ping Hu
Shiang Hu

Shijia Hu
Songlin Hu
Susie Hu
Tianhui Hu
Tsung-Hui Hu
Wanglai Hu
Wan-Hsiang Hu
Wei Hu
Wei-Syun Hu
Wen-Pin Hu
Wenxiang Hu
Wenxing Hu
Wuhua Hu
Wuyang Hu
Xiang-Qun Hu
Xiaomeng Hu
Xiaoxiang Hu
Xin Hu
Xinyu Hu
Xudong Hu
Xuebo Hu
Yan Hu
Yanmei Hu
Yanqing Hu
Yaowu Hu
Yi Hu
Yi-Bo Hu
Yifei Hu
Yihong Hu
Ying Hu
Yingang Hu
Yong Hu
Yongli Hu
Yu Hu
Yu-Chen Hu
Yueh-Chiang Hu
Yufeng Hu
Yun Hu
Yunzhao Hu
Yuxin Hu
Zanmin Hu
Zhaonong Hu
Zhenghui Hu
Zhenhua Hu
Zhiguo Hu
Zhongli Hu
Zhongyuan Hu
Bao-Zhen Hua
Changchun Hua
Guoqiang Hua
Hongbo Hua
Jian Hua
Jingyu Hua
Jinlian Hua
Kuo-Feng Hua
Li Hua
May Hua
Susan Hua
Yuchao Hua
Hua Kuang Hua Kuang
Jeannette Huaman
Shitong Huan
Xiujia Huan
Yanjun Huan
Ailing Huang
Allen Huang
Baojun Huang
Carol Huang
Changling Huang
Chang-Ming Huang
Changsheng Huang
Chang-Wen Huang
Chao Huang
Chao-Li Huang
Chen Huang
Chi-Chen Huang
Chih-Yang Huang
Chung-Feng Huang
Chu-Ren Huang
Congxin Huang
Cui Huang
Danqiong Huang
Danwei Huang
David Huang
Ding-Wei Huang
Faqing Huang
Fu-Chen Huang
Gao Huang

Greg Huang
Guan-Hua Huang
Guo-Hua Huang
Guomin Huang
Haiming Huang
Haiqing Huang
Hairong Huang
Han-Chang Huang
Hao Huang
Hao-Jen Huang
Hongwen Huang
Hsiang Huang
Hsiang-Wen Huang
Hsiao-Ying Shadow Huang
Hsien-Da Huang
Hsuan-Ming Huang
Huawei Huang
Huei-Ling Huang
Huey-Chun Huang
Hui Huang
Hui-Chun Huang
Jian An Huang
Jian Huang
Jihong Huang
Jin Huang
Jing Huang
Jingfang Huang
Jingyi Huang
Jinyan Huang
Jiun-Yan Huang Huang
Ji-Yuan Huang
Jun Huang
Junming Huang
Kai Huang
Kai-Wen Huang
Kun Huang
Kuo-Chuan Huang
Le Huang
Lei Huang
Li Huang
Lihan Huang
Lili Huang
Li-Ling Huang
Linfang Huang
Ling Huang
Liquan Huang
Li-Rung Huang
Liusheng Huang
Maoyi Huang
Mark Huang
Meng-Chuan Huang
Min Huang
Ming-Chun Huang
Ming-Chyi Huang
Mu-Hsuan Huang
Ngan Huang
Ning Huang
Peng Huang
Penghui Huang
Pintong Huang
Po-Chin Huang
Po-Hsien Huang
Possu Huang
Qiaobing Huang
Qiaoqiao Huang
Qifang Huang
Qihong Huang
Qingming Huang
Qingxu Huang
Qiong Huang
Qiongyu Huang
Qiu Huang
Qiuying Huang
Qunying Huang
Renhua Huang
Ruby Yun-Ju Huang
Ruyi Huang
San-Yuan Huang
Shaobin Huang
Shengfeng Huang
Shiang Fen Huang
Shiang-Fu Huang
Shih-Feng Huang
Shile Huang
Shiu-Feng Huang
Shuaibo Huang
Shuang Huang
Shuning Huang

Songyin Huang
Steven Huang
Suhua Huang
Tai-Huang Huang
Tao Huang
Ting-Ting Huang
Tzu-Chou Huang
Weidong Huang
Wei-Jen Huang
Weini Huang
Weishan Huang
Wenjing Huang
Wenjuan Huang
Wenjun Huang
Wen-Sheng Huang
Xianghua Huang
Xiang-Run Huang
Xianzheng Huang
Xiaolin Huang
Xiao-San Huang
Xiaoxi Huang
Xin Huang
Xinyi Huang
Xuefei Huang
Xuehui Huang
Xuemei Huang
Xueying Huang
Yadong Huang
Yafan Huang
Yan Huang
Yanbo Huang
Yanjun Huang
Yao-Wei Huang
Yehong Huang
Yhu-Chering Huang
Yin Huang
Ying Huang
Ying-Hsien Huang
Yingze Huang
Yi-Shin Huang
Yi-Wen Huang
Yong Huang
Youjun Huang
Yu-Jei Huang
Yuji Huang
Yunhui Huang
Yuqing Huang
Yu-Tung Huang
Yvonne Huang
Zhaohui Huang
Zhen Huang
Zhihuan Huang
Zhipeng Huang
Zhu Huang
Zhuoyi Huang
Zirui Huang
Zunnan Huang
Zu-Shi Huang
Wu Huan-Gan
Isabel Huang-Doran
Caroline Huart
Marcela Huart
François Huaux
Jaroslav Hubacek
David Hubbard
Penny Hubbard
Peter Hubbard
Rebecca Hubbard
Timothy Hubbard
Alex Hubbe
Stephen Hubbell
Carl Hubel
Djuro Huber
Gregory Huber
Katharina Huber
Ludwig Huber
Manuel Huber
Sally Huber
Stefan Huber
Veronica Huber
Veronika Huber
Jan Hubert
Anita Hubley
Dirk Hubmacher
Megan Huchko
Bernhard Huchzermeyer
Cord Huchzermeyer
Christian. Huck

Mark Huckvale
Sophie Huddart
Michael Hudecek
Kristin Hudock
Kristelle Hudry
Amanda Hudson
Damien Hudson
James Hudson
Olivier Hue
Christian Huebbers
Peter Huebener
Axel Hueber
Wolfgang Hueber
Robert Huebert
Erwin Huebner
Jutta Huebner
Kay Huebner
Karen Huen
Graciela Huerta
José María Huerta
Margarita Huesca
Yvette Huet
Alfredo Huete
Robert Hueter
Markus Huettel
Fabian Huettig
Falk Hüettmann
Ann Marie Huffenberger
Sonya Huffman
Joachim Hüffmeier
Gary Huffnagle
Kristina Hufford
Andreas Hug
Christina Hugenschmidt
Stefan Huggenberger
Jane Huggins
Alicia Hughes
Amanda Hughes
Charles Hughes
David Hughes
James Hughes
Jane Hughes
Kevin Hughes
Samantha Hughes
Stephen Hughes
Thomas E. Hughes
Timothy Hughes
Martin Hugh-Jones
Frederick Hughson
Christoph Hugi
Christian Hugo
John Huguenard
Pil-Woo Huh
Winston Huh
Ilpo Huhtaniemi
Ben Hui
Francis Hui
Kar Hoou Hui
Kwai Fung Hui
Pak Ming Hui
Pun Hui
Sheng (Tony) Hui
Tan Ai Hui
Xuan Hui
Jon Huibregtse
Barbara Huijgen
Robbert Huijsman
Barbara Huisamen
Anders Huitfeldt
Philippe Hujoel
Maciej Huk
Monica Hulbert
Jiri Hulcr
Mahmoud Huleihel
Todd Hulgan
Elaine Hull
Kerry Hull
Pam Hull
Rebecca Hull
Vanessa Hull
Esther Hulleman
John Hulleman
Philipp Hüllemann
Anke Hüls
Ina Hulsegge
Catherine Hulsof
Eva Hulstaert
Kristina Hulten

Nilantha Hulugalle
Matthew Hulver
Lucienne Human
Atul Humar
Sajid Humayun
Richard Humber
Clifford Hume
David Hume
Anne Humeau-Heurtier
Sandra Hummel
Edith Hummler
Helmut Hummler
Christian Humpel
Hans-Ulrich Humpf
Louise Humphrey
Parris Humphrey
Thomas Humphrey
David Humphreys
Kate Humphreys
Mike Humphreys
Debbie Humphries
Holly Humphries
Mark Humphries
Song-Lih Hunag
Martin Hund
Gadissa Hundie
Lena Hünefeld
Chao-Hung Hung
Chi-Chih Hung
Chien-Ching Hung
Ching-I Hung
Galen Chin-Lun Hung
I-Kuai Hung
Ivan F. N. Hung
Jia-Jang Hung
Keng-Lou Hung
Kuan-Yu Hung
Kuo-Hsiang Hung
Ming-Jui Hung
Ming-Yow Hung
Rayjean Hung
Sandy Hung
Shao-Min Hung
Szu-Chun Hung
Ya Ching Hung
Daniel Hungerford
Carol Hunja
Gary Hunninghake
Stacie Hunsaker
Mauricio Hunsche
Alice Hunt
Barnaby Hunt
Beverley Hunt
C. Anthony Hunt
Chris Hunt
Edmund Hunt
Greg Hunt
James Hunt
John Hunt
Kevin Hunt
Peter Hunt
Stephen Hunt
W. Hunt
George Hunt Jr.
Julia Huntenburg
Abby Hunter
Catherine Hunter
Christopher Hunter
Donald Hunter
Fiona Hunter
Gary R. Hunter
James Hunter
John Hunter
Jonathan Hunter
Kendall Hunter
Kent Hunter
Lisa Hunter
Margaret Hunter
Mitchell Hunter
Rachael Hunter
Robert Hunter
William Hunter
Jason Huntley
Bo Huo
Hong Huo
Li-Jun Huo
M. Q. Huo
Yuankai Huo

Zhiguang Huo
Nicolas Huot
Philippe Huot
Dan Hupalo
Philippe Hupé
David Hupin
Stacey Huppert
Muhammad Huq
Nafisa Lira Huq
Md. Hamidul Huque
Rumana Huque
Gang Min Hur
Ji-Won Hur
Mirna Hur
Pilwon Hur
Soojung Claire Hur
Yera Hur
Yoonkang Hur
Christelle Hureau
Juan Hurle
Emily Hurley
James Hurley
Katrina Hurley
Robert Hurley
Jennifer Hurlow
Karl Michael Hurn
Douglas Hurst
Samia Hurst
Thomas Hurst
Aeron Hurt
Barry Hurwitz
Mary Hurwitz
Shahid Husain
Bettina Husebo
Martin Husemann
Azamal Husen
Lili Husniati Yaacob
Michaëla Huson
Afzal Hussain
Anwar Hussain
Maria Hussain
Muzaffar Hussain
Sabah Hussain
Shamima Hussain
Tabish Hussain
Tanweer Hussain
Ahmed Hussein
Islam Hussein
Khaled Hussein
M. Hussein
Shereen Hussein
Sophia Hussen
Michael Hust
Søren Husted
Roland Hustinx
Carla L. Huston
Christopher Huston
Joseph Huston
Laura Huston
Wilhelmina Huston
Willa Huston
Rolf Hut
Joshua Hutcheson
B. Hutchins
David Hutchins
Angela Hutchinson
Brian Hutchinson
Elizabeth Hutchinson
Mark Hutchinson
Raymond Hutchinson
Lauren Hutchison
Paul Hutchison
William Hutchison
Jeffrey Hutsler
Alan Hutson
Paul Hutson
Maik Huttemann
Benedikt Huttner
David Hutton
Raymond Hutubessy
Claudio S. Hutz
Bianca Huurneman
Andrea Huwiler
Sharon Huws
David Huyben
Roger Huybrechts
Mark Huycke
Kris Huygen

Flavia Huygens
Mary Huynh
Thanh-Tuan Huynh
Vân Anh Huynh-Thu
Christian Hvas
Lars Magnus Hvattum
Ai-Wen Hwang
Andrew Hwang
De-Kuang Hwang
Dong-Uk Hwang
Eileen Hwang
Han-Jeong Hwang
Hyisung Hwang
Jeeseong Hwang
Jenn-Kang Hwang
Jongnam Hwang
Juen-Haur Hwang
Jun-Eul Hwang
Jungwook Hwang
Ming-Jing Hwang
Peter H. Hwang
Pung-Pung Hwang
Rosa Hwang
Thomas Hwang
Tsong-Long Hwang
Y. S. Hwang
Wuh-Liang Hwu
Hyacinth Hyacinth
Christian Hyde
R. Katherine Hyde
John Hyett
Catherine Hyland
Philip Hyland
Ole Hyldegaard
Anthony Hyman
James Hyman
Richard Hyman
Weston Hymas
Noreen Hynes
Wayne Hynes
Kullervo Hynynen
Jung Hyosun
Tuulia Hyötyläinen
Thomas Hyphantis
Elina Hypponen
Karice Hyun
Sang-Hwan Hyun
Seung-Jae Hyun
Robert Hyzy
Guido Iaccarino
Chiara Iacconi
Gianluca Iacobellis
Roberto Iacone
Claudio Iaconetti
William Iacono
Fortunata Iacopino
Massimo Iacoviello
David Iaea
Andrei Iagaru
Fabiola Iannarilli
Hernan Iannella
Danilo Iannetta
Pietro Iannetta
Micaela Iantorno
Ines Ibáñez
Alejandro Ibáñez-Costa
Noelia Ibanez-Escriche
Jorge Ibanez-Gijon
Şenol Ibanogˇ Lu
Paul Ibbotson
Bas Ibelings
Adriana Ibelli
Akachukwu Ibezim
Samad Ibitokou
Wafa Ibn Saied
Salvador Iborra
Guilhem Ibos
George Ibrahim
Marwa Ibrahim
Maryanne Ibrahim
Nadine Ibrahim
Nizar Ibrahim
Salam Ibrahim
Nabil Ibtehaz
Yoko Ibuka
Larry Icard
Parul Ichhpujani
Atsuhiro Ichihara

Daisuke Ichikawa
Atsuhiko Ichimura
Toshiharu Ichinose
Ronaldo Ichiyama
Stefanie Ickert-Bond
Takashi Ida
Michiru Ida-Eto
Soichiro Ide
Evgeny Idelevich
Olubukola Idoko
Iskandar Idris
Salim Idriss
William Idsardi
Ferdinando Iellamo
Riccardo Ientile
Marianthi Ierapetritou
Michele Iester
Wangshuxing Ieu
Francesca Ieva
Giandomenica Iezzi
Christina Ifrim
Shafia Iftekhar
Mohsin Iftikhar
Jun-Ichi Iga
R. Ariel Igal
Jotaro Igarashi
Katsuhide Igarashi
Kazuei Igarashi
Kiyohiko Igarashi
Michihiro Igarashi
Toru Igarashi
Michiya Igase
Satoshi Igawa
Lionel Igaz
E. O. Igbinosa
Suleiman Igdoura
José Roberto Iglesias
Mar Iglesias
P. Iglesias
Cifre Ignacio
Zuleide Ignácio
Anita Ignatius
Vera Ignjatovic
Igor Prudovsky
Naoya Iguchi
Taisen Iguchi
Tokuichi Iguchi
Toni Ihalainen
Kazushige Ihara
Masafumi Ihara
Atsushi Ihata
Espen Ihlen
Anna Ihnatowicz
Festus Ihwagi
Masato Iida
Tomoya Iida
Kyo Iigaya
Hideki Iijima
Noriaki Iino
Shahzada Ijaz
Hiroyuki Ijima
Yoko Ijima
Shigeho Ijiri
Auke Ijspeert
Tetsuro Ikebe
Andrea Ikeda
Kei Ikeda
Keita Ikeda
Koji Ikeda
Kota Ikeda
Masaaki Ikeda
Nayu Ikeda
Satoshi Ikeda
Shigaku Ikeda
Hidetoshi Ikegami
Kohta Ikegami
Koji Ikegami
Naoki Ikegami
Toru Ikegami
Mitsunori Ikeguchi
Melanie Ikeh
Takehito Ikejiri
Mai Ikemura
Hidetoshi Ikeno
Mhd Ikhwanuddin
Karen Ikin
T. Alp Ikizler
Amy Ikui

Toshikazu Ikuta
Govindasamy Ilangovan
Federica Ilardi
John Ilekis
Will Iles
Mikas Ilgunas
Dusko Ilic
Jugo Ilic
Sanja Ilic
Jasminka Ilich-Ernst
Marius Ilie
Kostantinos Iliodromitis
César Ilizaliturri-Hernández
Hazlee Illias
Svenja Illien-Junger
Björn Illing
Nicholas Illsley
Titilayo Ilori
Onaedo Ilozumba
Kiho Im
Enyu Imai
Hideaki Imai
Hiroo Imai
Hiroyuki Imai
Hissei Imai
Shin-Ichiro Imai
Takao Imai
Takeo Imai
Tsuyoshi Imai
Kazuhiko Imakawa
F. Imamura
Fumihiko Imamura
Hiromi Imamura
Hiroshi Imamura
Minako Imamura
Toshihiko Imamura
Yuka Imamura
Yuichi Imanaka
Kyoko Imanaka-Yoshida
Alex Imas
Toshiyuki Imasawa
Pascal Imbeault
Anne Imberty
Bruno Imbimbo
Carol Imbriano
Paul Imhoff
Roland Imhoff
John D. Imig
Stephan Immenschuh
Genji Imokawa
Issei Imoto
Valeria Imperato
Michael Imperiale
Soren Impey
Konstantinos Imprialos
Giovanni Improta
M. Imtiaz Hussain
Yoji Inaba
Yutaka Inaba
Caio Inácio
Ryo Inada
Toshiya Inada
M. Inada Kim
Takayuki Inagaki
Reiko Inagi
Hüseyin Ince
Robin Ince
Ümit Incekara
Sara Incera
Pablo Inchausti
Cesare Indiveri
Ciro Indolfi
Sabarish Indran
Abhaya Indrayan
Frank Infurna
Brian Ingalls
Roberto A. Ingaramo
Pablo Ingelmo
Martin Ingelsson
Victoria Ingham
Kanika Inglett
Vijendra Ingole
Armann Ingolfsson
Holly Ingraham
James Ingram
Maia Ingram
Travis Ingram
Erin Ingvalson

Pilar Iniesta
Alena Iñiguez
Steve Innes
Hamish Innes-Brown
Bernardo Innocenti
Naohiro Inohara
Masafumi Inomata
Andras Inotai
Daisuke Inoue
Ituro Inoue
Jun Inoue
Kazushi Inoue
Makoto Inoue
Nariaki Inoue
Nozomu Inoue
Satoshi Inoue
Seiji Inoue
Shiji Inoue
Takanari Inoue
Yoshio Inoue
Yosuke Inoue
David Inouye
Giuseppe Insalaco
Heribert Insam
E. Inskeep
Marcos Intaglietta
Paula Intasqui
Kiao Inthavong
Atsushi Into
Nobuyuki Inui
Paul Salvador Inventado
Alessandro Invernizzi
Pietro Invernizzi
Kris Inwood
Thomas Inzana
Andreea Ioan-Facsinay
Panagiotis Ioannidis
Christos Ioannou
Stephanos Ioannou
Pier Paolo Iodice
Allyson Ion
Egidio Iorio
Raffaele Iorio
Ruben Iosif
Bryan Iotti
Mirco Iotti
Juan Iovanna
Dennis Ip
Giovanbattista Ippoliti
Rodolfo Ippoliti
Danielle Ippolito
Matthew Ippolito
Anindya Iqbal
Farkhund Iqbal
Hafiz Iqbal
Javeed Iqbal
Jawed Iqbal
M. Bilal Iqbal
Md. Shahrear Iqbal
Mohammad Perwaiz Iqbal
Syed Iqbal
Srinivas Iragavarapu
Yazad Irani
Rabah Iratni
Shahriar Iravanian
Robert Ireland
Abreuire Rodríguez Irene
Leonid Irenge
Maria Jose Iriarte-Chiapusso
Sarah Iribarren
Nagore Iriberri
Ignis Iribhogbe
Kaoru Irie
R. Irie
Maria-Claudia Irigoyen
Daniel Irimia
Brian Iritani
Ryosuke Iritani
Özgür Irmak
Pui-Ying Iroh Tam
Maria Ironside
Andres Iroume
Azeem Irshad
Joseph Irudayaraj
Helen Irving
Hyacinth Irving
Amy Irwin
Andrew Irwin

Tracy Irwin
Adam Isaac
Clement Isaac
Vivian Isaac
Kim Isaacs
Stuart Isaacs
Laurie Isacco
Olena Isaeva
Ioannis Isaias
Michael Isakoff
Brant Isakson
Carlos Isales
Deanna Isaman
Stamatina Isari
Christina Isaxon
Till Ischebeck
Irene Iscla
Kohtaro Iseki
Sachiko Iseki
Claudio Isella
Sylvain Iseni
Joerg Isensee
Anuar Ishak
Waguih Ishak
Suzanne Ishaq
Kenichi Ishibashi
Kenji Ishibashi
Akihiro Ishibazawa
Fumihiro Ishida
Hideki Ishida
Tatsuhiro Ishida
Masaji Ishiguro
Yoshihito Ishihara
Haruyuki Ishii
Hiroshi Ishii
Kenichi Ishii
Kunihiko Ishii
Shunsuke Ishii
Akira Ishikawa
Hiroto Ishikawa
Naoko Ishikawa
Naoto Ishikawa
Yasuyuki Ishikawa
Yoshiki Ishikawa
Takatsugu Ishimoto
Chikashi Ishioka
Gail Ishiyama
John Ishiyama
Nobukazu Ishizaka
Jane Ishmael
Tauheed Ishrat
Andrea Isidori
Ciro Isidoro
Chinwoke Isiguzo
Ahmet Isik
D. Robert Iskander
Ihsan Iskesen
Maria Ines Isla
Aminul Islam
Farahnaz Islam
M. Mazharul Islam
M. Mofizul Islam
Mainul Islam
Md. Islam
Md. Shahidul Islam
Md. Shofiqul Islam
Mohammad Islam
Munia Islam
Rafiq Islam
S. K. Hafizul Islam
Saiful Islam
Salim Islam
Shaheen Islam
Shamsul Islam
Shofiq Islam
Towhidul Islam
Suryadi Ismadji
Ayesha Ismail
Hanafy Ismail
Helene Ismail
Mohannad Ismail
N. A. Ismail
Niveen Ismail
Shehab Ismail
Onur Ismi
Richard Isnard
Kikuo Isoda
Nina Isoherranen

Tsuyoshi Isojima
Clay Isom
Lori Isom
Michael Ison
Shuji Isotani
Theocharis Ispoglou
Iaroslav Ispolatov
Dawn Israel
Mark Israel
Salomon Israel
Naim Issa
David Issadore
Natalia Issaeva
Surapol Issaragrisil
Taghrid Istivan
Toth Istvan
Michio Itabashi
Ryo Itabashi
Tadashi Itagaki
Jun Itakura
Salvatore Italia
Yuval Itan
Keiichi Itatani
Sujay Subbayya Ithychanda
Daisuke Ito
Hiroki Ito
Hiroshi Ito
Kiyoaki Ito
Marcia Ito
Masaoki Ito
Minami Ito
Nobuaki Ito
Satoru Ito
Takashi Ito
Takuo Ito
Tsuyoshi Ito
Yasuhiro Ito
Yoko Ito
Yutaka Ito
Hideomi Itoh
Hiroaki Itoh
Hiroshi Itoh
Kanako Itoh
昌広 伊藤
Yasushi Itoh
Yoshifumi Itoh
Eiji Itoi
Lars Ittner
Unyime S. Ituk
Ursula Iturrarán-Viveros
Iñaki Iturrate
Jagoba Iturri
Michal Itzhaki
Steven H. Itzkowitz
Jennifer Iudicello
Jorge Iulek
Gerardo Iuliano
Sandra Iuliano-Burns
Joseph B. Moore IV
Giovanni Ivaldi
Silvina Ivalo
Hajek Ivan
Mircea Ivan
Lacramioara Ivanciu
Renata Ivanek
Viatcheslav Ivanenko
Julijana Ivanisevic
Andrey Ivankin
Natalia Ivankova
Alex Ivanov
Alexander Ivanov
Andrei Ivanov
Andrey Ivanov
Ivan Ivanov
Vadim Ivanov
Natalia Ivanova
Nevena Ivanovic
Tatyana Ivanovska
Andreas Ivarsson
Dmitriy Ivashchenko
Angela Ivask
Kaisa Ivaska
Domagoj Ivastinovic
Richard Ivell
Ann-Charlotte Iversen
Ljiljana Ivesa
Alan Ivkovic
Masato Iwabu

Toshiki Iwabuchi
Masao Iwagami
Toshiyuki Iwahori
Leo Iwai
Rtyichi Iwakiri
Yuriko Iwakura
Masahiro Iwamoto
Ryoichiro Iwanaga
Masayuki Iwano
Shingo Iwano
Kuni Iwasa
Laura Iwasaki
Masanori Iwasaki
Yasumasa Iwasaki
Yuka Iwasaki
Atsushi Iwasawa
Satoshi Iwase
Yoshio Iwashima
Urszula Iwaszczuk
Hisataka Iwata
Junichi Iwata
Masaaki Iwata
Minoru Iwata
Yasunori Iwata
Yusuke Iwata
Aiko Iwata-Otsubo
Keiichi Iwaya
Benson Iweriebor
Anthony Iwuafor
Collins Iwuji
Jean-Marie Iyamba
Arpana Iyengar
Chaaya Iyengar
Abhirami Iyer
Aditya Iyer
Aparna Iyer
Arun Iyer
Jyoti Iyer
Sriya Iyer
Saeed Izadi
Zohreh Izadifar
Kazuhiro Izawa
Shingo Izawa
Shuhei Izawa
Tetsuya Izawa
Aaron Izenberg
Jacques Izopet
Eduardo Izquierdo
Luis Izquierdo
David Izquierdo-Garcia
Nuria Izquierdo-Useros
Evgeny Izumchenko
Tadahide Izumi
Yukitoshi Izumi
Yasuhiro Izumiya
Ayako Izuno
Ken-Ichi Izutsu
Valerio Izzi
Pascal Izzicupo
Fairuz Izzudin
Holst J.
Ramalingam J.
Bradley J. Roth
William Ja
Rola Jaafar
Kelly Jaakkola
Laura Jaakola
Satu K. Jääskeläinen
Abdul Jabbar
Reza Jabbari
Ramazan Jabbarli
Ehsan Jabbarzadeh
Jad Jabbour
Zahra Jabeen
Jose Raduan Jaber
Tarek Jaber
Sybille Jablonka
Ewa Jablonska
Daniel Jablonski
Michal Jablonsky
A. Jabor
Khawar Jabran
Rodrigo Jacamo
Hugo Jachmann
Allison Jack
Rachael Jack
Markus Jäckel
Sebastian Jäckle

Paul Jacklin
Gemma Jacklyn
Jane Jackman
William Jackman
Conrad Jacksich
Benjamin Jackson
Brian Jackson
Charlene R. Jackson
Charlotte Jackson
Claire Jackson
Colin Jackson
Edwin Jackson
Heide Jackson
Ian Jackson
Isabel Jackson
Jamie Jackson
Jeffrey Jackson
Jonathan Jackson
Joshua Jackson
Kristina Jackson
Mandy Jackson
Margaret Jackson
Marie Jackson
Michael Jackson
Philip Jackson
Robert J. Jackson
Robert Jackson
Shelley Jackson
Stephen Jackson
Thomas Jackson
Walker Jackson
Mark Jackwood
Christopher Jacob
Francis Jacob
Gunnar Jacob
Jens Jacob
John Jacob
Joseph Jacob
Ralf Jacob
Richard Jacob
Shomona Jacob
James Jacobberger
Frank Jacobi
Choolwe Jacobs
Daniel Jacobs
David Jacobs
Elizabeth Jacobs
Jeremy Jacobs
Jesse Jacobs
Maarten Jacobs
Maartje Jacobs
Michael Jacobs
Robin J. Jacobs
William Jacobs
David Jacobs Jr.
Ilse Jacobsen
Joyce Jacobsen
Karsten Jacobsen
Stine Jacobsen
Thomas Jacobsen
Marcelo Jacobs-Lorena
David Jacobson
Jeffrey Jacobson
Joseph Jacobson
Sandra Jacobson
Deborah Jacobs-Sera
Marc Jacofsky
Carolina Jacometo
Alain Jacot
Vincent Jacquemet
Hans Jacquemyn
Frederic Jacques
Frédéric Jacques
Marie-Agnès Jacques
Mario Jacques
Suzanne Jacques
Laloe Jacques-Olivier
Herve Jacquier
Emmanuelle Jacquin-Joly
Maude Jacquot
Evelyne Jacqz-Aigrain
Janusz Jadasz
Sudarshan Jadcherla
Karin Jäderlund
Gopal Jadhav
Murtuza Jadliwala
Saad Jadoo
David Jaeger

Gerhard Jaeger
Matthias Jaeger
Anne Jaerve
Aino Jaervelin
Jaewan Jaewan Choi
Tazeen Jafar
Amin Jafari Sojahrood
Mohammad Jafarnejad
Amir Jafarnezhad
Zeina Jaffar
David Jaffe
Eileen Jaffe
Iris Jaffe
Klaus Jaffe
Bahram Jafrasteh
Syed Jafri
Santosh Jagadeeshan
Guru Jagadeeswaran
Ramasamy Jagadeeswaran
Madan Jagasia
Jared Jagdeo
Benedikt Jäger
Pamela Jagger
Preeti Jaggi
Krzysztof Jagla
Jagnoor Jagnoor
Adam Jagodinsky
Reinhold Jagsch
Sujit Jagtap
Grazyna Jagura-Burdzy
Ismat Jahan
S. Jahanfar
Leila Jahangiry
Rahman Jahanian
Tom Jahans-Price
Zeinab Jahed
Inge Jahn
Willi Jahnen-Dechent
Peter Jahns
Babak Jahromi
Sébastien Jaillon
Rider Jaimes-Reategui
Amita Jain
Ankur Jain
Chirag Jain
Deepak Jain
Deepika Jain
Diwakar Jain
Felipe Jain
Gaurav Jain
Kavita Jain
Maneesh Jain
Pawan Jain
Priyesh Jain
Rajan Jain
Rajeev Jain
Shalu Jain
Sushil K. Jain
Vikas Jain
Yogesh Jain
Stephanie Jainta
Jiten Jaipuria
Teele Jairus
Dinesh Jaishankar
Amit Kumar Jaiswal
Aruna Jaiswal
Vandana Jaiswal
Cheedy Jaja
Andras Jakab
Pratibha Jakhu
Birgit Jaki
Bogdan Jakiela
Mark Jakiela
Martina Jakob
Peter Jakob
Stephan Jakob
Tatjana Jakobs
Lasse Jakobsen
Jan Jakobsson
Asgeir Jakola
Vladimir Jakovljevic
Valdas Jakštas
Daniela Jakubowicz
Aleksandra Jakubowski
Claudia Jakubzick
Garg Jalaj
Diana Jalal
Mohammad Jalali

Sarika Jalan
Isabelle Jalbert
Somayeh Jalilvand
Sirpa Jalkanen
Archana Jalligampala
George Jallo
Ngoneh Jallow
Isabel Jalon Rojas
Mohammad Jamal
Syed Jamal
L. Jameel
Alex James
Alexander James
Andrew James
Arthur R. James
Chloe James
David James
Gary James
Harvey James
Helen James
John T. James
Judith James
Louisa James
Michael James
Sherman A. James
Simon James
Laurie James-Hawkins
Geoffrey Jameson
Mary Jameson
Tamara James-Todd
Elie Jamie
Nigel Jamieson
Kaiser Jamil
Muhammad Jamil
Keith Jamison
Henry Jampel
Shazia Jamshed
Ankur Jamwal
Deepa Rana Jamwal
Arif Tasleem Jan
Plelaert Jan
Ren-Long Jan
Soumya Jana
Karolina Janacsek
Manochehr Janagard
Malvin Janal
Colleen Janczewski
Tibor Janda
Karin Jandeleit-Dahm
Georg Jander
Moody Jandhyala
Robert Jandl
Stefan Janecek
Štěpán Janeček
Jan Janecka
James Janetka
Sandra Janezic
Anna Jang
Byoung Kuk Jang
Eun Sun Jang
Hee-Chang Jang
Hee-Jin Jang
Hyewon Jang
Jae Eun Jang
Jae Young Jang
Jong Wook Jang
Jyan-Chyun Jang
Mi-Kyeong Jang
Sang-Hun Jang
Sehwan Jang
Seogjoo Jang
Seonghoe Jang
Seongsoo Jang
Tae-Sik Jang
Woncheol Jang
Wooyoung Jang
Kamlesh Jangid
Hanna Jangö
Nrupa Jani
Elizabeth Janiak
Vincent Janik
Zorica Janjetovic
Stacey Jankelowitz
Anuradha Jankiraman
Dragana Jankovic
Matthew Jankowich
Agnieszka Jankowicz-Szymanska
Marta Jankowska

Joachim Jankowski
Konrad Jankowski
Fernanda Janku
Piyapong Janmaimool
Paul Janmey
Amynah Janmohamed
Dietmar Jannach
Franziska Jannasch
Jim Jannes
Vincent Jannin
Emmanuele A. Jannini
Claire Janoir
Amol Janorkar
Jan Janouškovec
Miroslaw Janowski
Xanne Janse De Jonge
Bernard J. Jansen
Floor Jansen
Florian Jansen
Jacobus Jansen
Jesse Jansen
Katrien Jansen
Laura Jansen
M. J. A. Jansen
Nicolaas Jansen
Petra Jansen
Ray Jansen
Stefanie Jansen
Petrus Jansen van Vuren
Pidder Jansen-Dürr
Emiel Janssen
Heido Janssen
Paul Janssen
Ralf Janssen
S. Janssen
Saskia Janssen
Yvonne Janssen-Heininger
Barbara Janssens
Geert Janssens
Steven Janssens
Johan Jansson
Lauren Jansson
Janis Jansz
Thijs Jansz
Prévost Jantchou
Jonathan Jantsch
Michael Jantz
Fraser Januchowski-Hartley
Patrick Janulis
Magdalena Janus
Kai Januschowski
Aleksandra Januszewska
Philippe Janvier
Tavan Janvilisri
Daniel Janzen
Sebastien Jaquemet
Antoine Jaquet
Zaira Jara
Abdullah Jaradat
Ainhoa Jaramillo-Gutiérrez
Laura Jarboe
Anthony Jarc
Peter Jarcuska
Aaron Jarden
Meg Jardine
Narissara Jariyapan
David P. Jarmolowicz
Kamil Jaron
Andrew Jarosz
Andrzej Jaroszyński
Mohamed Jarraya
Paige Jarreau
Kendall Jarrett
Edward Jarroll
Michael Jarstfer
Hanna Jarva
Kati Järvelä
Kajsa Jarvholm
Jeffrey Jarvik
Joseph Jarvis
Paul Jarvis
Jason Jarzembowski
Wolfgang Jaschinski
Grazyna Jasienska
Jaroslaw Jasiewicz
Bernard Jasmin
Cynthia Jasper
Pawel Jastreboff

Kamil Jastrzebski
Rajneesh Jaswal
Ram Jat
Ralf Jauch
Frank Jauker
Elita Jauneikaite
Sabzali Javadov
Marco Javarone
Amjad Javed
Farrukh Javed
Mohammad Javed
Mustafa Javed
Zeeshan Javed
Babak Javid
Etienne Javouhey
Veronika Javůrková
Mohammed Jawad
Coline Jaworski
Chadwick Jay
E. Jaya
Amal Jayakumar
Elamparithi Jayamani
Harikrishnan Jayamohan
Ciriyam Jayaprakash
G. Jayaprakasha
Rama Jayaraj
B. Jayaram
Vinay Jayaram
Dhileepkumar Jayaraman
Jayakumar Jayaraman
Sudha Jayaraman
Thottala Jayaraman
Vasanthi Jayaraman
Sampath Jayarathna
Saroj Jayasinghe
Chantal Jayat-Vignoles
Ranil Jayawardena
Lewis Jayes
Lee-Ann Jaykus
R. S. Jayshree
Mehrdad Jazayeri
Mohammad-Ali Jazayeri
Janez Jazbec
Christopher Jazwa
Anna Jaźwińska
S. Jazwinski
Aurelie Jeandron
Angela Jeanes
Tiphaine Jeanniard-Du-Dot
Pascale Jeannin
Frank Jeanplong
Jamel Jebali
David Jebb
Mulusew Jebena
Andreas Jechow
Jean-Marc Jeckelmann
Behrouz Jedari
Katarzyna Jednoróg
Włodzimierz Jędrzejewski
Elima Jedy-Agba
Maciej Jedynak
Rajesh Jeewon
William Jeffcoate
Beth Jefferies
Caroline A. Jefferies
Jana Jeffers
Thomas Jefferson
Kate Jeffery
Nicholas Jeffery
Nick Jeffery
Stephen Jeffery
Brendan Jeffrey
David Jeffries
Marlo Jeffries
Lianne Jeffs
R. Jegadeeshwaran
Sinthujan Jegaskanda
Feyisayo Jegede
Jean-François Jegou
George Jeha
Frederic Jehan
Robert Jehle
Shireen Jejeebhoy
Elissa Jelalian
Jaroslav Jelinek
Kurt Jellinger
W. Jellish
Tarun Jeloka

Albert Jeltsch
Michael Jeltsch
Jennifer Jemc
Gregor Jemec
Katalin Jemnitz
Julie Jemutai
Bhanu P. Jena
Naresh Jena
Mohammad-Ali Jenabian
Eric Jenczewski
Verena Jendrossek
Jiann-Shing Jeng
Jiiang-Huei Jeng
Mei-Jy Jeng
Wei Jeng
Wen-Juei Jeng
Yung-Ming Jeng
Rajesh Jeniton
Arthur Jenkins
Cathy Jenkins
David Jenkins
Frank Jenkins
Helen Jenkins
Nathaniel Jenkins
Richard Jenkins
Rob Jenkins
Stuart Jenkins
Todd M. Jenkins
Maria Jenmalm
Dieter Jenne
Christopher Jennelle
Malgorzata Jennes
Domhnall Jennings
Garry Jennings
Jackson Jennings
Jacky Jennings
Skyler Jennings
Michael Jennions
Amy Jennison
Andreas Jenny
Anders A. Jensen
Anders Jensen
Andrew Jensen
Asther Agnete Jensen
Boye Jensen
Frank Jensen
Frants Jensen
Gitte Jensen
Helen Jensen
Henrik Jensen
Jamie Jensen
Kirk Jensen
Kristian Kiim Jensen
Kristin Jensen
Murray Jensen
Natasja Jensen
Olaf Jensen
Paul Jensen
Poul Jensen
Ralph Jensen
Sigmund Jensen
Signe Jensen
Todd M. Jensen
William Jensen
Ulf Jensen-Kondering
Sebastian Jentschke
J. Jeon
Jong Jeon
Joo Jeon
Justin Jeon
Kwon Jeon
Woojin Jeon
Chang Jeong
Dong-Hoon Jeong
Hyundoo Jeong
Hyunyoung Jeong
Jeong-Won Jeong
Kwangcheol Jeong
Kyungah Jeong
Soung Won Jeong
Unyong Jeong
Yong Jeong
Young-Sik Jeong
Jin Wook Jeoung
Sunil Jeph
Marieke Jepma
Per Jeppesen
Niels Jepsen

Mark Jepson
Peter Jeranyama
Travis Jerde
Barbara Alicja Jereczek-Fossa
Wander Jeremias
Young Jeremy
Igor Jerkovic
Johannes Jermakka
Igor Jerman
Ashley Jermusyk
Jih-Shuin Jerng
Robert Jernigan
Last Jerold
Ana Jeroncic
Carmen Jerónimo
Carsten Jers
Barbara Jeršek
Gunnar Jeschke
Udo Jeschke
Thomas Jespersen
Niels Jessen
Sarah Jessen
Ellen Jessouroun
David Jessup
Dilip Jeste
James Jester
Rachel Jesudasan
Preeti Jethwa
Kanika Jetmalani
Marc Jeuland
Patrick Jeurissen
Monika Jevšnik
Carl Jewell
Derek Jewell
Nicholas Jewell
Rachel Jewkes
Jeyaraman Jeyakanthan
Shanmugam Murugaiha Jeyakumar
Nitthiyah Jeyaratnam
William Jeynes
Maria Jeziorska
Tereza Jezkova
Dhruva Kumar Jha
Jay Jha
Nayansi Jha
Prabhat N. Jha
Rajesh Jha
Rajesh Kumar Jha
Uday Jha
Yadvendradev Jhala
Meena Jhanwar-Uniyal
Niyati Jhaveri
Mahima Jhingan
Min A. Jhun
Chen Ji
Chunyan Ji
Conghua Ji
Dong-Feng Ji
Fuhai Ji
Hong Ji
Hongbin Ji
Hongtao Ji
Junliang Ji
Kefeng Ji
Kun Ji
Lei Ji
Lili Ji
Linqin Ji
Shuiwang Ji
Tian Ji
Xiang Ji
Xiaoxi Ji
Yanyan Ji
Zhiwei Ji
Dongya Jia
Dongyao Jia
Fujun Jia
Huijue Jia
Jianhua Jia
Junjing Jia
Liting Jia
Rong Jia
Ruixue Jia
Shu Jia
Shuqin Jia
Tao Jia
Tong Jia
Wangcun Jia

Weiping Jia
Wenkai Jia
Xiuqin Jia
Xiuyi Jia
Yanbin Jia
Yilin Jia
Yong Jia
Zhengping Jia
Zhiyun Jia
Zongchao Jia
Awachana Jiamsakul
Zhen Jian
Tang Jianbo
Baoguo Jiang
Binyan Jiang
Chunsun Jiang
Cunmei Jiang
Dong Jiang
Dongyun Jiang
Fang Jiang
Guihua Jiang
Guoxing Jiang
Hanlin Jiang
Heng Jiang
Hong Jiang
Hongen Jiang
Honglin Jiang
Hua Jiang
Huawu Jiang
Hui Jiang
Jack Jiang
Jean Jiang
Jian-Li Jiang
Jianmin Jiang
Jicai Jiang
Jinjin Jiang
Junjun Jiang
Lei Jiang
Libo Jiang
Lili Jiang
Liwen Jiang
Lurong Jiang
Maorong Jiang
Peiyong Jiang
Pengyao Jiang
Ping Jiang
Qi Jiang
Qin Jiang
Qing Jiang
Qiyan Jiang
Rays Jiang
Shancheng Jiang
Shengyao Jiang
Shijin Jiang
Shuguang Jiang
Shulong Jiang
Siwen Jiang
Tao Jiang
Wei Jiang
Wen Jiang
Xi Jiang
Xiang-Chen Jiang
Xiaoming Jiang
Xing Fu Jiang
Xingpeng Jiang
Xinyin Jiang
Xujie Jiang
Xuntian Jiang
Yangsheng Jiang
Yanliang Jiang
Yannan Jiang
Yawen Jiang
Yi Jiang
Yichuan Jiang
Yinghua Jiang
Yinlai Jiang
Yiwei Jiang
Yizhang Jiang
Yong Jiang
Yu Jiang
Yuchao Jiang
Yue Jiang
Yueyang Jiang
Yun-Jin Jiang
Zhe Jiang
Zhiqiang Jiang
Zhi-Qiang Jiang
Zhongli Jiang

Zhongyuan Jiang
Zide Jiang
Zongliang Jiang
Wu Jianmin
Li Jianping
Chen Jiao
Mingli Jiao
Shuliang Jiao
Ting Jiao
Wei-Wei Jiao
Xiadong Jiao
Xiaoguo Jiao
Yu-Yong Jiao
Guli Jiapaer
Luo Jiaquan
Lai Ji-Ching
John Jifon
Hannah Jilani
Robert Jilka
Veronika Jílková
Masamine Jimba
David Jimenez
Francisca Jimenez
Joaquin Jimenez
Jose Jimenez
Juan de la Cruz Jimenez
Eduard Jiménez
Gerardo Jiménez
Natalia Jiménez
María Victoria Jiménez Franco
William Jimenez Leal
Veronica Jimenez Ortiz
Alejandro Jiménez Sosa Jiménez Sosa
Francesc Jiménez-Altayó
Javier Jiménez-Candil
Jose Jimenez-Chillaron
David Jimenez-Gomez
José Jiménez-Heffernan
Eijiro Jimi
Beck Jimikaye
Chunyuan Jin
Dakai Jin
Dezhe Jin
Di Jin
Eonseon Jin
Faguang Jin
Fengliang Jin
Fengyi Jin
Fulai Jin
Guangfu Jin
Guangze Jin
Hao Jin
Hongmei Jin
J. P. Jin
Jennifer Jin
Jia-Min Jin
Jian Jin
Jie Jin
Jing Bo Jin
Jing Jin
Jiyong Jin
Jun-O Jin
Lei Jin
Li Jin
Long Jin
Meiyan Jin
Minghao Jin
Qingwei Jin
Sheng Chih Jin
Wen Jin
Wenchi Jin
Xia Jin
Xiaohang Jin
Xiao-Hua Jin
Xiaowei Jin
Xin Jin
Xiuliang Jin
Yan Jin
Yanfei Jin
Yi Jin
Zhao Jin
Zhe Jin
Zhijun Jin
Zi-Bing Jin
Young Jin Tak
Irena Jindrichovska
Goodwin Jinesh

Huaiqi Jing
Wei-Xin Jing
Wu Jing
Xiaoyuan Jing
Zhao Jing
Zhaoqian Jing
Tsung-Luo Jinn
Masatoshi Jinnin
Umesh Jinwal
Paulin Jirkof
Kim Jiseon
Tina Jiwatram-Negrón
Hang-Hyun Jo
Helen Jo
Insu Jo
Jun-Ichiro Jo
Min-Woo Jo
Robinson Jo
Seongbong Jo
Yonghwa Jo
Stephanie Joachim
Michal Joachimiak
Michael Joannidis
Andrei Joaquim
Taufique Joarder
Dominique Job
Malcolm Jobling
Alexander Jobs
Simon Jobson
Samia Joca
Simon Jochems
Ralf Jockers
Elizabeth Jockusch
Tim Joda
Meritxell Jodar
Patrick Jodice
William Joe
Elin Joergensen
Rolf Joerger
Ari Joffe
Mireia Jofre-Bonet
Amod Jog
Mandar Jog
Tania Jogesh
Annika Jögi
Gerwald Jogl
Anand Jogota
Charles Johannessen
Suzanne Johanningsmeier
Leif Johannsen
Anders Johansen
Kristen Johansen
Martin Johansen
Michelle Johansen
Ragnhild Elise Johansen
Steinar Johansen
Terje Johansen
Zerina Johanson
Anders Johansson
Björn Johansson
Emma Johansson
Hanna Johansson
Ingegerd Johansson
Mattias Johansson
Unn-Britt Johansson
Kirpa Johar
Amit Joharapurkar
Nor Johdi
Eric Johlin
Alison John
Ann John
Annie John
Esther John
Mike John
Rosalind John
Steven John
Thomas John
Neha John-Henderson
Benjamin Johns
Jennifer Johns
Michelle Johns
Murray Johns
Eric Johnsen
Sonke Johnsen
Alexander Johnson
Allison Johnson
Asal M. Johnson
Bruce Johnson

Candice Johnson
Casey Johnson
Casonya Johnson
Cheryl Johnson
Chris Johnson
Christopher Johnson
Curtis Johnson
Daniel Johnson
David Johnson
Derek Johnson
Eric Johnson
Evan Johnson
Glyn Johnson
James Johnson
Jason Johnson
Jay Johnson
John Johnson
Joseph Johnson
Judith Johnson
Judy Ann Johnson
Julene Johnson
K. Ian Johnson
Katherine Johnson
Kathy Johnson
Keith Johnson
Kenneth Johnson
Liam Johnson
Marc Johnson
Maribeth Johnson
Marilyn Johnson
Mark Johnson
Matthew Johnson
Miriam Johnson
Natalie Johnson
Nessa Johnson
Newell Johnson
Owen Johnson
Patricia Johnson
Paul Johnson
Peter Johnson
Philip Johnson
Rabia Johnson
Richard Johnson
Rodney Johnson
Russell Johnson
Ruth Johnson
Scott Johnson
Sindhu Johnson
Steven Johnson
T. Johnson
Teresa Johnson
Theron Johnson
Timothy Johnson
Tom Johnson
Valerie Johnson
Warren Johnson
Wayne Johnson
William Johnson
Andrew Johnston
Carol Johnston
Christopher Johnston
Craig Johnston
David Johnston
Fabian Johnston
James Johnston
Jodie Johnston
Randal Johnston
Daniel Johnstone
Jeanette M. Johnstone
Stuart Johnstone
Manu Johny
Sören Johst
Wilsaan Joiner
Vijaya Jojo
Jesse Jokerst
Asbjorn Jokstad
Darukeshwara Joladarashi
Alain Joliot
Genevieve Jolivet
Jukka Jolkkonen
Daniel Jolley
Ann Jolly
Clare Jolly
Emmitt Jolly
Kate Jolly
Saira Jolly
Carlos Joly
Jean-Stéphane Joly

Aurélie Joly-Amado
Godwin Jombo
Marie Jo-Moutin
Clare Jonas
Dorn Jonas
Elizabeth Jonas
Rene Jonas
Jennifer Jonason
Peter Jonason
Inge Jonckheere
Nicolas Jonckheere
Rafal Jończyk
Erik Joner
Andrew Jones
Arwel Jones
Barbara Jones
Ben Jones
Benjamin Jones
Brad Jones
Byron Jones
Cassandra Jones
Christopher Jones
Clinton Jones
D. Jones
Daniel Jones
Darryl Jones
David Jones
Edward Jones
Edward Samuel Jones
Elena Jones
Elizabeth Jones
Eric Jones
Gareth Jones
Gary Jones
Gregory Jones
Harrison Jones
Ian Jones
Jay Jones
Jeffery Jones
Jessica Jones
Jim Jones
Jonathan Jones
Julia Jones
Kathryn Jones
Landon Jones
Lara Jones
Lee Jones
Lesley Jones
Lisa Jones
Martin Jones
Meaghan Jones
Michael Jones
Neil Jones
Nev Jones
Nicole Jones
Nina Jones
Oliver Jones
Patrick Jones
Paul Jones
Rachel Jones
Rebecca Jones
Reese Jones
Richard Jones
Rodney Jones
Salene Jones
Simon Jones
Stephanie Jones
Stephen Jones
Steven Jones
Stuart Jones
Wendell Jones
Valerie Jones Taylor
Gwo-Ping Jong
Monica Jong
Jan Jongbloed
Aldo Jongejan
Eelke Jongejans
Allard Jongman
Danny Jonigk
Donatas Jonikaitis
Ilse Jonkers
Karen Jonscher
Johanna Jonsdottir
Svava Jonsdottir
Ian Jonsen
Bengt-Harald Jonsson
Bert Jonsson
K. Jönsson

Sven Jonuscheit
Chulmin Joo
Deokjin Joo
Dong Jin Joo
Heesoo Joo
Jorge Joo-Nagata
Marie Joossens
Tinbergen Joost
Leo Joosten
Michel Joosten
Robbie Joosten
Rosa Joosten
Simone Joosten
Pavol Joppa
Kaveh Jorabchi
Bjarte Henry Jordal
Daniel Jordan
David Jordan
Eric Jordan
Helen Jordan
Irmgard Jordan
Mark Jordan
Meggan Jordan
Michael Jordan
Nancy Jordan
Philip Jordan
Rebecca Jordan
Robert Jordan
Susan Jordan
Ferenc Jordán
Jordi Jordana
Rolf Jorde
Stephan Jörg
Fatima Jorge
Rey Jorge
Rodrigo Jorge
Veronique Jorge
Dana R. Jorgensen
Jens Erik Jorgensen
Joan Jorgensen
Pernille Jorgensen
Peter M. Jorgensen
Terrence Jorgensen
Christian Jørgensen
Hanne Skou Jørgensen
Silje Jørgensen
Vanda Jorgetti
F. Jori
Olaf Jöris
Anthony Jorm
Arul Jose
Ricardo Jose
Djalma José Fagundes
Jami Josefson
Anders Josefsson
Kristina Josefsson
Antonio Joseph
Benesh Joseph
Jane Joseph
K. Joseph
Lawrence Joseph
Robert Joseph
Shanthi Joseph
Dvora Joseph Davey
Emily Josephs
Abhilasha Joshi
Anagha Joshi
Atul Joshi
Bharat Joshi
Bimba Joshi
Hiren Joshi
Manali Joshi
Manisha Joshi
Nirav Joshi
P. C. Joshi
Pheroze Joshi
Rajendra Joshi
Rohit Joshi
Suresh Joshi
U. N. Joshi
Srijana Joshi Rijal
Vasna Joshua
Grace Joshy
Djuro Josic
Quentin Jossart
Yves Jossin
Christian Jost
John Jost

Nancy Joste
Zdravko Jotanovic
Deepika Jothinathan
Valérie Jotterand Drepper
Yuh-Shan Jou
Emilie Jouanjus
Laurent Journot
Céline Jousse
Olivier Jousson
Grégory Jouvion
Miljana Jovandaric
Aleksandar Jovanovic
Dejana Jovanovic
Nikolina Jovanovic
Slavisa Jovanovic
Mariona Jové
J. Joven
Rodrigo Jover
Miguel Jover Cerdá
Ljiljana Jowitt
Anita Joy
Andrea Joyce
Walter Joyce
Chester Joyner
Vink Joy-Sarah
Damian Józefiak
Pawel Jozkow
Alicja Jozkowicz
Sergiusz Jozwiak
Yasmina Jraissati
Imane Jroundi
Bin Ju
Cynthia Ju
Feng Ju
Ji Hyeon Ju
Jyh-Cherng Ju
Wenjun Ju
Xiang Ju
Xiangqun Ju
Yang Ju
Bolea Juan
Chi-Chang Juan
Manel Juan
Yi-Je Juang
Edwin Juarez
Miguel Juarez
Sol Juárez
Ali Judd
Walter Judd
Howard Judelson
Kevin Judge
Pedro B. Júdice
Marc Judson
Shannon Juengst
Verena Juergens
M. Jugulam
Csaba Juhasz
Gabor Juhasz
Levente Juhasz
Christofer Juhlin
Catherine Juillard
Yvon Julé
Ryan Julian
Esther Julián
V. Julián
Stuginski-Barbosa Juliana
Mendes Juliana Frossard Ribeiro
Berry Juliandi
Margarida Julià-Sapé
Andrew Jull
Janet Jull
Donald Jump
Gyungah Jun
Hee-Sook Jun
Jin Hyun Jun
JinWoo Jun
Liu Jun
Howard Junca
Romina Juncos
Sandeep Juneja
Andreas Jung
Bernd Jung
Franziska Jung
Hans Jung
Jae Hwan Jung
Jae Won Jung
Jinkyu Jung
Keun-Hwa Jung

Ki-Hong Jung
Klaus Jung
Kyu Sik Jung
Mary Jung
Michaela Jung
Simon Jung
Su Yon Jung
Sukgeun Jung
Susanne Jung
Wi Hoon Jung
Won-Kyo Jung
Woo-Sung Jung
Young Hoon Jung
Youngmi Jung
Anne Jungblut
Harald Junge
Harald Jungnickel
Wolfgang Jungraithmayr
Antonio Junior
Kerstin Junker
Robert Junker
Stephane Junot
Andre Junqueira
Juliana Junqueira
Juhani Junttila
Bianca Jupp
Beatriz Jurado
Kohta Juraku
Ivan Juran
Rytis Juras
Anamarija Jurcev-Savicevic
Michael Jurczak
Raja Jurdak
Russell Jurenka
Sarah Jurick
Alo Juriloo
Janine Jurkowski
Giuseppe Jurman
Mario Juruena
Peter Jurutka
Elizabeth Jury
Todd Jusko
William Jusko
Lee Jussim
Lothar Just
Steffen Just
Silvia Justi
Anne Justice
Sheryl Justice
Jean-Lou Justine
Giselle Justo
Marko Jusup
Brandon Jutras
Bernhard Juurlink
Parijat Juvale
Risto Juvonen
Garima Juyal
Asta Juzeniene
Gaurav Jyani
