# Supplementary material for: PLOS ONE 2017 Reviewer and Editorial Board Thank You
Source: PLoS One. 2018 Mar 15;13(3):e0194158. doi: 10.1371/journal.pone.0194158 (PMC5854357; doi:10.1371/journal.pone.0194158)
Supplement: S3 Reviewer List — Alphabetical list of 2017 PLOS ONE peer reviewers (K-N). (PDF) [file pone.0194158.s005.pdf]

PLOS | ONE

PLOS ONE would like to thank all those who reviewed on behalf of the journal in 2017:

Chandrashekar K.
Dinesh Kumar K.
Sangeetha K.
Vinod K. K.
Hakhyun Ka
Shuk-Man Ka
Anne Marije Kaag
Rudolf Kaaks
Niina E. Kaartinen
Vesa Kaartinen
Tea Kaartokallio
Valtteri Kaasinen
Martin Kaatz
Tamon Kabata
Mirjam Kabel
Enamul Kabir
Muammar Kabir
Russell Kabir
Parijat Kabiraj
Stefan Kabisch
Sandor Kabos
Yasushi Kaburagi
Wieslaw Kaca
Asli Kacar
Malika Kachani
Markus Kächele
George Kachergis
Vasiliki Kachrimanidou
Priyanka Kachroo
Rachel Kachur
S. Patrick Kachur
Niko Kaciroti
Rachel Kacmar
Bogumil Kaczkowski
Irena Kaczmarska
Tadeusz Kaczorowski
Tejas Kadakia
Prashant Kadam
Shilpa Kadam
Deepak Kadayakkara
John Kaddis
Anil Kadegowda
Claus Kadelka
Rene Kaden
Didem Kadihasanoglu
Sudarsana Reddy Kadiri
Krishna Kadirvelu
Kristina Kadlec
Mikiko Kadohisa
Hiroya Kadokawa
Keiko Kadono-Okuda
David Kadosh
Takashi Kadowaki
Tatsuhiko Kadowaki
Bashkim Kadriu
Igor Kaefer
Raymond Kaempfer
Bernhard Kaess
Michael Kaess
Jaranit Kaewkungwal
Thammarat Kaewmanee
Wafaa Kaf
Jafar Kafaie
Vivian Kafantaris
Salih Kafkas
Senay Kafkas
Şenay Kafkas
Tal Kafri
Kimitaka Kaga
Yoshimi Kaga
Kotaro Kagawa
Masaharu Kagawa
Tatehiro Kagawa
Tetsushi Kagawa
Anna Kågesten
Daisuke Kageyama
Shinichi Kageyama
Georg Kägi

Bashar Kahaleh
Shoshana Kahana
Maria Kahar Bador
Gila Kahila Bar-Gal
Barbara Kahl
Erin Kahle
Waltraud Kahle
J. Michelle Kahlenberg
Charlene Kahler
Ulf Kahlert
Andrea Kahn
Ashraful Kahn
Daniel Kahn
Itamar Kahn
Jeremy Kahn
Shulamit Kahn
Kathrin Kahnert
Sevim Kahraman
Guoyin Kai
Hirofumi Kai
Hisashi Kai
Zhao Kai
Angela Kaida
Daisuke Kaida
Toshimi Kaido
Georgia Kaidonis
Darnell Kaigler
Ville Kaila
Andrzej Kaim
David Kaimowitz
Danielle Kain
Kevin Kain
Morgan Kain
Bernd Kaina
Angela Kaindl
Alexander Kainz
Benny Kaipparettu
Daniel Kaiser
Elias Kaiser
German Kaiser
Laure Kaiser
Thorsten Kaiser
Tu'uhevaha Kaitu'u-Lino
Omprakash Kaiwartya
Marina R. Kaizer
Kleineberg Kaj Kolja
Amala Kaja
Murali Kaja
Andrey Kajava
Aiko Kaji
Hiroshi Kaji
Izumi Kaji
Tomonari Kaji
Hiroshi Kajihara
Yukie Kajita
Hiroshi Kajiya
Robert Kajobe
Adriana Kajon
Łukasz Kajtoch
Kavita Kakarala
Aarti Kakatkar
Akinobu Kakigi
Yoshihiko Kakinuma
Terutaka Kakiuchi
Masako Kakizaki
Shigeyuki Kakizawa
Nadira Sultana Kakoly
Ken Kakudo
Byron Kakulas
Ritsuko Kakuma
Madhavi Kakumanu
Kenichiro Kakutani
László Kalabay
Evangelos Kalaitzakis
Hazem Kalaji
Theodoros Kalampokas
Spyros Kalams
Lindsay Kalan
Raj K. Kalapatapu
Raj Kalaria
Marina Kalashnikova
Siripen Kalayanarooj
Sita Kalayanarooj
Corey Andrew Kalbaugh
Miriam Kalbitz
Hans Robert Kalbitzer
Lee Kalcsits
Niilo Kaldalu

J. A. Kalef-Ezra
Amy Kaleita
Alexis Kalergis
Satish Kalhan
Amy Kalia
Lorraine Kalia
Priya Kalia
Maximos A. Kaliakatsos-Papakostas
Jérôme Kalifa
Andre Kalil
Sahir Kalim
Kyriaki Kalimeri
Hannu Kalimo
Latif Kalin
Tomas Kalincik
Laura Kaliniene
Natalia Kalinina
Olga Kalinina
Ulrich Kalinke
Antigoni Kaliontzopoulou
Richard Kalisa
Tobias Kalisch
Jatin Kalita
Jayantee Kalita
Sanjib Kalita
Emma Kalk
Satish Kallappagoudar
Toivo Kallas
Zein Kallas
Sven Kalle
Kimberly Kalli
Asha Kallianpur
Sakari Kallio
Elisa Kallioniemi
Staci Kallish
Teemu Kallonen
Michael Kalloniatis
Nicolai Kallscheuer
Ulf Kallweit
David Kalmbach
Martin Kalmokoff
Uldis Kalnenieks
Eleftheria Kalogera
Andreas Kalogeropoulos
Ameeta Kalokhe
Isgouhi Kaloshian
Dimitris Kaloudas
Oliver Kalpak
Ganjam Kalpana
Bhawna Kalra
Dinesh Kalra
Lalit Kalra
Rajat Kalra
Aino Kalske
Daniela Kalthoff
Jonathan Kaltman
Marco Kaltofen
Christian Kaltschmidt
Brian Kaluf
Sanjeeva Kalva
Carlos Kalva Filho
Ramki Kalyanaraman
Ramaswamy Kalyanasundaram
Jonghun Kam
Julia Kam
Yiu Wing Kam
Masamitsu Kamada
Ayeesha Kamran Kamal
Faisal Kamal
Maud Kamal
Susan Kamal
Mohammadreza Kamali
Masood Kamali-Moghaddam
Dilara Kaman
David Kamara
Prashant Kamat
Rujvi Kamat
Takaaki Kamatani
Nivedita Kamath
Pauline Kamath
Vasudeva Kamath
Beena Kamath-Rayne
Konstantinos Kambas
Neeraja Kambham
Robert Kambic
Hideto Kameda
Asuka Kamei

Daigo Kamei
Nozomu Kamei
Yasutomi Kamei
Nick Kamenos
Petra Kameritsch
Maarten Kamermans
Hidehiro Kamezaki
Taro Kamigaki
Takehiko Kamijo
Daisuke Kamimura
Hiroteru Kamimura
Kenya Kamimura
Shinji Kamimura
Hironori Kaminaka
Bozena Kaminska
Gwenael Kaminski
Henry Kaminski
Atsushi Kamiya
Hajime Kamiya
Haruyuki Kamiya
Hiroyuki Kamiya
Kazutaka Kamiya
Kentaro Kamiya
Takehiro Kamiya
Toshiya Kamiyama
Yoshimi Kamiyama
Madhu Kamle
Björn-Ole Kamm
Thomas Kammer
Sara Kammerer
Peer Kämmerer
Leonie Kamminga
Michael Kammüller
Olga Kamneva
Marcel Kamp
Astrid Kamperman
Gueneter Kampf
Lars Kamphuis
Sandra Kamping
Jatupol Kampuansai
Md. Kamruzzaman
Bernard Kamsu-Foguem
Andrey Kan
Biao Kan
Haiming Kan
Kees Jan Kan
Man Shan Kan
Weiwei Kan
Rahul Kanadia
John Kanady
Motoi Kanagawa
Yoshihiro Kanai
Ioannis Kanakis
Oleg Kanakov
Jill Kanaley
Mariano Kanamori
Satoru Kanamori
Christopher Kanan
Rornald Kananura
Batur Kanar
Alexandre Kanashiro
Kenichi Kanatani
Naoki Kanayama
Ippei Kanazawa
Masato Kanazawa
Rajashekhar Kanchanapally
Vijaya Kancherla
Waldemar Kanczkowski
Tatsuo Kanda
Tomonori Kanda
Yoshinobu Kanda
Ngianga Kandala
Kirthevasan Kandasamy
Saravanakumar Kandasamy
Eugene Kandel
Rita Kandel
Thomas Kander
Sami Kanderian
Farid Kandil
Raju Kandimalla
Ramesh Kandimalla
Mustapha Kandouz
Sasikiran Kandula
Christina Kandutsch
Adam Kane
Corinne Kane
Evan Kane
Jeremy Kane

Lawrence Kane
Patricia Kane
Robert L. Kane
Suzanne Kane
Katsuyuki Kaneda
Kazue Kanehara
Masao Kaneki
Akira Kaneko
Hiroto Kaneko
Kotaro Kaneko
M. Kaneko
Shuji Kaneko
Yoshikatsu Kaneko
Yuji Kaneko
Yuko Kaneko
Masato Kanemaki
Simon Kaner
Ravindran Kanesvaran
Carla Kaneto
Byoung-Cheorl Kang
Byungkyu Kang
Chang Hyun Kang
Chang-Keun Kang
CongBao Kang
Dan Kang
Dongchon Kang
Eun Kang
Fei Kang
Gyeong Hoon Kang
Hakmook Kang
Hang-Bong Kang
Hong-Gu Kang
Hongpu Kang
Houxiang Kang
Hunseung Kang
Hye Won Kang
Hyojung Kang
Hyoung Jin Kang
Hyun Kang
Jaewoo Kang
Jidong Kang
Kai Kang
Keon Wook Kang
Kun-Tai Kang
Kyungsu Kang
Min Suk Kang
Mo-Yeol Kang
Pauline Kang
Sang-Hee Kang
Sanghoon Kang
Seungcheol Kang
Shin-Wook Kang
Si-Yong Kang
Sona Kang
Su Yun Kang
Sun Kang
Sung Wook Kang
Tae Wook Kang
Xian-Jiang Kang
Young-Hee Kang
Yun Kang
Yunhee Kang
Zhensheng Kang
Saijaliisa Kangasjarvi
Shreya Kangovi
P. Kangueane
Lovney Kanguru
Yuri Kaniovski
Jean Kanitakis
Hannu Kankaanranta
Phyllis Kanki
Tomotake Kanki
Srinivasaraghavan Kannan
T. R. Kannan
Vijay Kannan
Ramaswamy Kannappan
Stephen Kanne
Yoshihiko Kannno
Yosuke Kanno
Pekka Kannus
Junko Kano
Rui Kano
Yasunori Kano
Deepak Kanojia
Michael Kanost
Hatice Kansu-Celik
Ami Kantar
Melda Kantar

Alpdogan Kantarci
Konstantinos Kantartzis
Eva Kantelhardt
Julie Kanter
Nidhi Kantharia
Jimut Kanti Ghosh
Tatsuya Kanto
Elizabeth Kantor
Ivan Kantor
E. Kantorová
Ira Kantrowitz-Gordon
Suman Kanungo
Jagat Kanwar
Stefan Kanzok
Albert Kao
Cheng-Yuan Kao
Chen-Yu Kao
Chien-Min Kao
Chih-Chin Kao
Chuan-Liang Kao
Hao-Yun Kao
Jia-Horng Kao
Lillian Kao
Peter Kao
Rowland Kao
Tze-Wah Kao
Cyrus Kapadia
Farzana Kapadia
Kostas Kapellas
Christoph Kapeller
Dinesh Kaphle
Saidi Kapiga
Cameron Kaplan
Cevdet Kaplan
Daniel Kaplan
David Kaplan
Jennifer Kaplan
Jonathan Kaplan
Richard Kaplan
Warren Kaplan
Zane S. Kaplan
Dimitrios Kapogiannis
Sunil Kapoor
Vaishali Kapoor
Andras Kaposi
Pál Kaposi
Madhu Kappagantu
Ilias Kappas
Carrie Kappel
David Kappel
János Kappelmayer
Elisabeth Kappos
Bill Kapralos
Maxim Kapralov
Jagadis Kapuganti
Gaurav Kapur
Reuben Kapur
Andras Kapus
Aurélie Kapusta
Alexander Kapustin
Larry Kapustka
Jarmo Käpylä
Partha Kar
Rajiv Kar
Abdul Qadar Kara
Melik Kara
Umit Karabiyik
Andromachi Karachaliou
Marianna Karachaliou
Niki Karachaliou
Bulent Karadag
Mevlude Karadag
Ömer Karadağ
Roxani Karagiannis
Pinar Karagoz
Aki Käräjämäki
Andrea Kara-Jose
Mahir Karakas
Ioannis Karakis
Giorgos Karakousis
Daniela Karall
Ahmed Karam
Chafic Karam
Alexandros Karamanlidis
Yannis Karamanos
Konstantina Karamanou
Spyridoula Karamanou
Dimitrios Karampinos

Anup Karan
Narasimha Kumar Karanam
Anjali Karande
Robin Karanek
Emily Karanges
Evangelos Karanikas
Panagiotis Karanis
Ioannis Karapanagiotis
Akihiro Karashima
Ece Karatan
Ilia Karatsoreos
Christina Karatzaferi
Jennifer Karberg
Michael Karbiener
Dan Stieper Karbing
Bozena Karbowska
André Karch
François Karch
Daniel Kardefelt Winther
Gabor Kardos
József Kardos
Marty Kardos
Joseph Kardouni
Andrew Karduna
Isabella Kardys
Hassen Kared
Antony Karelis
Dana Karelus
Fatemeh Kargarfard
Segraves Kari
Thomas Karikari
Ali Karim
F. Karim
Lamya Karim
Mohd Karim
Nasiara Karim
Rashed Karim
Rezaul Karim
Shahid Karim
Shamsul Karim
Zubair Karim
Alireza Karimi
Fariba Karimi
Hamid Reza Karimi
Javad Karimi
Maryam Karimi
Samuel Kariuki
Sofia Karkampouna
Abhilasha Karkey
Shakun Karki
Lisa Karlov
Erik Karlsson
Fredrik Karlsson
Ida Karlsson
Linn Karlsson
Patrik Karlsson
Magnus Karlsteen
Rakesh Karmacharya
Chandan Karmakar
Parimal Karmakar
Faisal Karmali
Wilfried Karmaus
Christof Karmonik
S. L. Rajasekhar Karna
Jaanika Kärner
A. E. Karnoub
Thomas Karnowski
Seth Karol
Golnaz Karoubi
Evgenia Karousou
Marisa Karow
Krishna Karpe
George Karpouzas
Elena Karpova
Phillip Karpowicz
Nancy Karraker
Johan Karremans
Katherine Karriker-Jaffe
George Karris
Marton Karsai
Andrey Karshikoff
Stephanie Karst
Bettina Karsten
John Karstensen
Michael Karsy
Yuri Kartavtsev
Vilvapathy Senguttuvan
Karthikeyan

Mikko Karttunen
Max Karukivi
S. Karumanchi
Devarajan Karunagaran
Panduka Karunanayake
Indrani Karunasagar
Shunmugiah Karutha Pandian
Simon Kärvemo
Diana Karwan
Maciej Karwowski
Waldemar Karwowski
Anastasios Karydis
Hideko Kasahara
Kiyoto Kasai
Takashi Kasai
Nongyao Kasatpibal
Karina Kasawara
D. P. Kasbekar
Annemarie Käsbohrer
Michael Kaschak
Elena Kaschina
Sabine Kasel
Benjamin Kasenda
Joseph Kaser
Thomas Kash
Kianoush Kashani
Nasim Kashef
Sumbul Kashif
Hirofumi Kashii
Hideaki Kashima
Hirokazu Kashiwagi
Shinichiro Kashiwagi
Elena Kashuba
M. P. Kashyap
Rajpal Kashyap
Vasundhra Kashyap
Anup Kasi
Anna Kasielska-Trojan
Ramanathan Kasimanickam
Andrea Kasinski
Konstantinos Kasiotis
Diego Kaski
Jan Kaslin
Enkelejda Kasneci
Martin Kasny
Ursula Kaspar
Tomas Kasparovsky
Dennis Kasper
Michael Kasper
Daniel Kass
David Kass
Getachew Kassa
Aliya Kassam
Amir Kassam
Modar Kassan
Stavros Kassinos
Zamaneh Kassiri
Amin Kassis
Andrea Kassner
Gerald Kastberger
Florian Kasten
Jude Kastens
Monica Kasting
Randee Kastner
Panagiotis Kastritis
Shoko Kasuga
Stefanie Kasurinen
Masatoshi Katabuchi
I. Katageri
Sayaka Katagiri
Takenobu Katagiri
Yoshinori Katakura
Ramesh Katam
Robab Katani
Hiroaki Kataoka
Rajesh Katare
Ranjit Kataria
Ayumi Katayama
Naoki Katayama
Lorig Kate
Michael Katehakis
David Katende
Kandasamy Kathiresan
Roger Kathol
Lurain Kathryn
Peiman Kathryn
Moritz Kaths
Neerja Katiyar

Santosh Katiyar
Gopal Katna
Aki Kato
Fumihiro Kato
Hideki Kato
Hironori Kato
Johji Kato
Junji Kato
Katsuhiko Kato
Kazuhisa Kato
Ken Kato
Koichi Kato
Makoto Kato
Masaru Kato
Mitsuo Kato
Satoshi Kato
Saul Kato
Shin Kato
So Kato
Taichi Kato
Takahiro Kato
Yuki Kato
Akira Katoh
Hironori Katoh
Midori Kato-Maeda
Michael Katovich
Christos Katris
Katerina Katsani
Konstantinos Katsanos
George Katsaros
Dimitrios Katsavelis
Konstantinos Katsoulis
Panagiotis Katsoulos
Maki Katsuhara
Ayako Katsumata
Yasuhiro Katsumata
Tomohiro Katsuya
Daniel Kattnig
Karen Katula
Kalyan Katuri
Hugo Katus
Sucharit Katyal
Joanne Katz
Lois Katz
Mark Katz
Patricia Katz
Robert Katz
Sherri Katz
Hans Katzberg
Judith Katzenellenbogen
David Katzenstein
Rita Katznelson
Todd Katzner
Rachel Katz-Sidlow
Massimiliano Kaucic
Carol Kauffman
David Kaufman
Elizabeth Kaufman
James Kaufman
Jim Kaufman
Kenton Kaufman
Tessa Kaufman
Christopher Kaufmann
Ines Kaufmann
Scott Kaufmann
Kaarina Kauhala
Marcus Kaul
Barbara Kaup
Soujanya Kaup
U. Benjamin Kaupp
Joonas Kauppila
Anu Kauppinen
Chamandeep Kaur
Gunjeet Kaur
Gurjinder Kaur
Gurvinder Kaur
Harparkash Kaur
Harpreet Kaur
Jaspinder Kaur
Manmeet Kaur
Prabhdeep Kaur
Sukhbir Kaur
Sukhwinder Kaur
Tejbeer Kaur
Christina Kauschke
Antti Kause
Margarita Kaushanskaya
Azad Kaushik

Gaurav Kaushik
Jai Kaushik
Nagendra Kaushik
Kevin Kaut
Markus Kautz
Servet Kavak
Ioannis Kavakiotis
Martin Kavaliers
Andrius Kavaliunas
Nickolas Kavallieratos
John Kavanagh
Shane Kavanagh
Saeid Kaviani
Zahra Kavosi
Jennifer Kavran
Saneyuki Kawabata
Jun-Ichi Kawabe
Soichiro Kawabe
Takumi Kawabe
Jun-Ichi Kawada
Manabu Kawada
Shigeo Kawada
Yasushi Kawaguchi
Genri Kawahara
Shigenori Kawahara
Ryouka Kawahara-Miki
Kimito Kawahata
Masanobu Kawai
Motofumi Kawai
Takayuki Kawai
Toshi Kawai
Koichi Kawakami
Ryoko Kawakami
Yasuhiko Kawakami
Tetsuya Kawakita
Kazumichi Kawakubo
Takakazu Kawamata
Dan Kawamori
Atsuhiko Kawamoto
Elisa Mitiko Kawamoto
Shin-Ya Kawamoto
Tetsuya Kawamoto
Yoshi Kawamoto
Makoto Kawamukai
Kazuhiro Kawamura
Daiji Kawanami
Hiroo Kawarazaki
Wakako Kawarazaki
Hideki Kawasaki
Yuichi Kawase
Masahiro Kawashima
Shinya Kawashiri
Akihiro Kawauchi
Takeshi Kawauchi
Naoki Kawazoe
R. Kawtummachai
Jennifer Kawwass
Adam Kay
Alan Kay
Chris Kay
Jerald Kay
Robin Kay
Cengiz Kaya
Mehmet Kaya
Yılmaz Kaya
Aya Kayali
Ghazi Kayali
Guven Kayaoglu
Jane Kaye
Gilles Kayem
Kassoum Kayentao
Afsin Kayipmaz
Jean Kayitsinga
Harvey Kayman
Gbenga Kayode
George Kaysen
Jürgen Kayser
Klaus Kayser
Lars Kayser
Tomo Kazama
Yusuke Kazama
Ludmila Kazdova
Hossein Kazemi
Yukinori Kazeto
Andrius Kazlauskas
Dhouha Kbaier
Santosh Kc
Hengning Ke

Po-Ju Ke
Po-Yuan Ke
Ruian Ke
Ruimin Ke
Yang Ke
Zun-Ji Ke
Paul Keall
Leslie Kean
Florence Keane
J. Ben Keane
Orla Keane
Robert Keane
Terence Keane
Graham Kearn
Alison Kearney
Anne Kearney
David Kearney
John Kearney
Michael Kearney
Benjamin Kearns
William Kearns
Mary Kearns-Jonker
Nancy Keating
Peter Keating
Jacob Keaton
Emily Keats
Bereket Kebede
Mihiretu Kebede
Moritz Kebschull
Zied Kechaou
Paulo Kechele
Bastian Keck
James Keck
Mira Keddis
Natalia Kedishvili
Monika Kedra
Lukasz Kedzierski
Chea-Su Kee
Kwong-Ming Kee
Mee-Kyung Kee
Younghoon Kee
Youngwook Kee
Dong Kee Jeong
Andrew Keech
Edward Keedwell
Douglas Keefe
Carol Keefer
Lucas Keefer
Jill Keeffe
Ken Keefover-Ring
Brian Keegan
Kevin Keegan
Kirk Keegan
Stuart Keel
Dave Keele
Courtney Keeler
John Keeling
Patricia Keely
Simon Keely
Jeremy Keenan
Rodney Keenan
Sarah Keenan
Tiarnan Keenan
Danielle Keenan-Miller
Alex Keene
Justin Keene
Marcus Keep
Nalina Keeran
Shivakumar Keerthikumar
James Keesling
Marijke Keestra-Gounder
Mark Keezer
Deborah Kegelmeyer
Michelle Kegler
Angie Kehagia
Izhak Kehat
Corinna Kehrenberg
Joyce Keifer
Jaap Keijer
Jessica Keim-Malpass
Hiroshi Keino
Peter Keir
David Keire
Lisa Keister
Jonathan Keith
Anouk Keizer
Eduard Kejnovsky
Peter Kekenes-Huskey

Uddhav Kelavkar
Almut Kelber
Olaf Kelber
Saskia Kelders
Linda Kelemen
Theodoros Kelesidis
Abdellali Kelil
Roya Kelishadi
Ian Kelleher
Rodney Kellems
Esther Kellenberger
Anna Keller
Bettina Keller
Charles Keller
E. Keller
Kathleen Keller
Klaus Keller
Maria Keller
Peter Keller
Manda Keller-Ross
Brigitte Keller-Stanislawski
Maureen Keller-Wood
Sergio Varela Kellesarian
Darcy Kelley
Darshan Kelley
George Kelley
Jennifer Kelley
Michael Kelley
Patricia Kelley
Stephen Kelley
Walter Kelley
Eleftherios Kellis
Elias Kellner
James Kellner
Kristell Kellner
Frauke Kellner-Weldon
Angela Kelly
Emer Kelly
Helen Kelly
J. Kelly
Kimberly Kelly
Luke Kelly
Maggi Kelly
Michael Kelly
Paul Kelly
Robert Kelly
Ryan Kelly
S. J. Kelly
Terra Kelly
Thomas Kelly
William Kelly
Malte Kelm
Karl Kelsey
David Kelso
Joel Kelso
David Kelton
Skye Kelty
Damian Kelty-Stephen
Emma Kelty-Stephen
Martin Kemler
Andre Kemmling
Joanne Kemp
Matthew Kemp
Melissa Kemp
William Kemp
Isabelle Kempf
Tibor Kempf
Barbara Kemp-Harper
Aleksander Kempny
Peter Kempster
Elisabeth Kemter
Soleakhena Ken
Shifra Ken Dror
Richard Kenagy
Justin Kenardy
Brian Kendall
Emily Kendall
Jacob Kendall
Matt Kendall
Neala Kendall
Sev Kender
Keith Kendrick
István Kenessey
Dror Kenett
Michael Keng
Shian-Ling Keng
Lin Keng-Hui
Andre Kengne

Jacques Kengne
Bojan Kenig
Paul-Ariel Kenigsberg
Miran Kenk
Ralph Kenna
Tony Kenna
Carrie-Ann Kennedy
Casey Kennedy
Chinaro Kennedy
David Kennedy
George Kennedy
Jessica A. Kennedy
Kristen Kennedy
Lindsey Kennedy
Malcolm Kennedy
Martyn Kennedy
Maureen Kennedy
Nabla Kennedy
Neil Kennedy
Oran Kennedy
Peter Kennedy
Rachel Kennedy
Ryan Kennedy
Sean E. Kennedy
Tay Kennedy
Timothy Kennedy
Steve Kennelly
Niall Kenneth
Douglas Kennett
Laurence Kenney
Rachel Kenney
Dianna Kenny
Glen Kenny
Nathan Kenny
Tsuyoshi Kenri
Andreana C. Kenrick
Julia M. Kensbock
David Kent
Erin Kent
George Kent
Shia Kent
Ben Kenward
Gary Kenward
Judson Kenworthy
Nora Kenworthy
Zoltan Kenyeres
Chris Kenyon Kenyon
Johanna Kenyon
Nicholas Kenyon
Phouthone Keohavong
Stella Kepha
Ryan Kepler
Thomas Kepler
Franz Keplinger
Hannah Keppler
Julia Keppler
Robert Kerbel
Leonardo Kerber
Jeffrey Kerby
Halima Kerdjoudj
Gyorgy Kerekes
Manjunath Keremane
Nir Keren
Margit Keresztes
Alan Kergunteuil
Hugo Kerhervé
Sam Kerk
Bonnie Kerker
Anthony Kermagoret
Kagan Kerman
Yvon Kermarrec
Elsa Kermorvant-Duchemin
Aurelie Kern
Christine Kern
Timothy S. Kern
Nancy Kerner
James Kerns
Jacqueline Kerr
Kathleen Kerr
Mickey Kerr
Peter Kerr
Deanna Kerrigan
Kamel Kerrouche
Inge Kersbergen
Mônica Kersch-Becker
Daniel Kerschensteiner
Oliver Kerscher
Pamela Kerschke-Risch

Ngaire Kerse
Gilbert Kersh
Arik Kershenbaum
Phil Kersten
Sander Kersten
David Kerstetter
Uwe Kersting
Peter Kertes
Vilmos Kertesz
A. F. Kertz
Jean Kerver
Laura Kervezee
Jason Kerwin
Jothydev Kesavadev
Lakshmyya Kesavalu
James Kesby
Ingrid Keseler
Zafe Keser
Shashank Keshavmurthy
Sailaja Kesiraju
Emre Keskin
Muhammed Emre Keskin
Tuulikki Keskitalo
Karen Keslar
Kenneth Kesler
Shelli Kesler
David Kessel
Dominique Kessel
Miriam Kesselmeier
Jurg Kesselring
Bradley Kesser
Christian Kessler
Harald Kessler
M. Kessler
Michael Kessler
Viktor Kessler
Thomas Kesteman
Dorothea Kesztyüs
James Ketchum
Daniel Ketelhuth
Kirsi Ketola
Maria Kett
Markus Ketteler
Robin Ketteler
Ellen Ketterson
James Ketudat Cairns
Oliver Keuling
Florian Keusch
Ajay Kevat
Anne Kever
Jess Kevill
Ioannis Kevrekidis
Alastair Key
Nigel Key
Anthony Keyburn
Linda Keyes
Nemat Keyhani
Yoav Keynan
Roxanne Keynejad
Gholamreza Khademi
Karim Khader
Jyoti Khadka
Anmar Khadra
Alka Khaitan
Bijay Khajanchi
Bacem Khalele
Syma Khalid
Michael Khalil
Mohammed Rohi Khalil
Mohd Ibrahim Khalil
Davood Khalili
Kamel Khalili
Amir Khaliq
Farhzad Khalvati
Mohammad Khami
Bavornlak Khamnamtong
Adnan Khan
Aisha Khan
Ajmal Khan
Ali Khan
Arif Khan
Arshad Khan
Asif Khan
Asma Khan
Gausal Khan
Ilyas Khan
Imran Khan
Khalid Khan

Khan Bahadar Khan
Mahmood Khan
Masood Khan
Matiullah Khan
Mazhar Khan
Meraj Khan
Mishal Khan
Mohammad Aslam Khan
Mohhamad Tahir Khan
Mohsin Khan
Muhammad Kasib Khan
Naim Khan
Naiman Khan
Nilofar Khan
Palwasha Khan
Raees Khan
Rasel Khan
Rashid Khan
Saeed Khan
Sami Ullah Khan
Samiullah Khan
Seema Khan
Shahid Khan
Shehroz Khan
Shujaul Khan
Suleman Khan
Tauseef Khan
Umar Khan
Wahid Khan
Waliul Khan
Wasif Khan
Zeyaur Khan
Zia Khan
Zohaib Khan
Samir Khanal
Arjun Khandare
Himanshu Khandelia
Farzaneh Khani
Yusef Khaniabadi
Larisa G. Khanina
Aditya S. Khanna
Rajiv Khanna
Sahil Khanna
Sunil Khanna
Garima Khare
Sangeeta Khare
Sunil Khare
Ritu Kharer
Nawwaf Kharma
Heather Kharouba
Ali Khashan
Reza Khasheir
Manar Khashram
Ali Khasseh
Camilo Khatchikian
Fabian Khateb
Hasan Khatib
Dipendra Khatiwada
Mehar Khatkar
Hafeeza Khatoon
Mahesh Khatri
Manisha Khatri
Resham Khatri
Vishal Khatri
Gaurav Khatwani
Anthony Khawaja
Saeed Khayatzadeh
Ali Khazaee
Maryam Khazaee-Pool
Maher Khdour
Farid Kheloufi
Naziha Khen-Dunlop
Mary Khetani
Farhad Khimani
Asem Khmag
Joel Khobondo
Fariba Khodagholi
Cyrus Khodani
Ahmad Khodayari Rostamabad
Konstantin Khodosevich
Mina A. Khoei
Samannaaz Khoja
Shara Kholia
Alan Soo-Beng Khoo
Edmund Khoo
Yasamin Khorramzadeh
Sabra KhorsandAhmadi
Habibeh Khoshbouei

Ali Reza Khosravi
Christine Khosropour
Tayebi Khosroshahi
Ali Khosrowbeygi
Farzin Khosrow-Khavar
Igor Khovanov
Kareeann Khow
Zaza Khuchua
Sadik Khuder
Tural Khudiyev
Surapan Khunamornpong
Budhachandra Khundrakpam
Dhanachandra Khuraijam
Atika Khurana
Satish Khurana
Seema Khurana
S. Kiami
Richard Kiang
Narsis A. Kiani
Reza Kiani
Ata Kiapour
Abdul-Ghani Kibbi
Simon Kibira
Muhammad Kibriya
Biniam Kidane
Kenneth Kidd
Mark Kidd
Thomas Kidd
Weerawat Kiddee
Steven Kiddle
Mallory Kidwell
Michael Kiebler
Marion Kiechle
Florian Kiefer
Jennifer Kielczewski
Margaret Kielian
Jan Kielstein
Michele Kiely
Francois Kiemde
Lambertus Kiemeney
Sabine Kienesberger
Dorothe Kienhues
Marie-Cécile Kienitz
Pascal Kienlen-Campard
Justyna Kierat
Uwe Kierdorf
Amy Kiernan
Dale Kiesewetter
Matthias Kieslinger
Aaron Kiess
Silke Kiessling
Thomas Kietzmann
Rogier Kievit
N. G. Kigozi
Ronald Kiguba
Juliet Kiguli
Takanori Kihara
Yasuki Kihara
John Kiiru
Takahiro Kikawada
Elise Kikis
Eiji Kikuchi
Haruhito Kikuchi
Iwaho Kikuchi
Jun Kikuchi
Mitsuru Kikuchi
Yoshitomo Kikuchi
Motoi Kikusato
Takefumi Kikusui
Mariana Kikuti
Zaal Kikvidze
John Kilbane
Angela Kilby
Thomas Kilduff
Mergerditch Kiledjian
James Kilgour
Julia Kilgour
Robert Kilgour
Reinhold Kilian
Fusun Kilic
Korhan Kilic
Ilker Kılıç
Oguz Kilinc
Saija Kiljunen
Monique Kilkenny
Clemens Kill
Edward Killan
Gerry Killeen

Shaun Killen
Mary Killilea
Timothy Killingback
Nabil Killiny
C. Kilpatrick
Laurie Kilpatrick
Michelle Kilpatrick
Anne Kiltie
Annice E. Kim
Beom Kyung Kim
Beom-Gi Kim
Bong-Hyun Kim
Bong-Sung Kim
Bongyoung Kim
Brian Kim
Bum Jung Kim
Bum-Joon Kim
Bw Kim
Byoungsoo Kim
Byung-Seok Kim
Byung-Wook Kim
Chang Hun Kim
Changhwan Kim
Chi Heonn Kim
Chul-Hong Kim
Dae Kim
Dae Woo Kim
Daegyoum Kim
Daehyun Kim
Daniel Kim
David Kim
Deaho Kim
Do Han Kim
Do Kyun Kim
Do-Gyoon Kim
Dohyeong Kim
Dong-Eog Kim
Dong-Hoon Kim
Dong-Yun Kim
Doo-Sub Kim
Dukjoon Kim
Eugene Kim
Eui-Jin Kim
Eunjoo Kim
George Kim
Grace Kim
Hae Won Kim
Hail Kim
Hak Jun Kim
Hee Jin Kim
Heebal Kim
Heejung Kim
Heui-Soo Kim
Heung Dong Kim
Hun Kim
Huy Kang Kim
Hwa-Jung Kim
Hwi Young Kim
Hye Kyong Kim
Hyeun Bum Kim
Hyo-Cheol Kim
Hyun Kim
Hyun Koo Kim
Hyun Soo Kim
Hyun-Duck Kim
Hyunil Kim
Il-Jin Kim
Il-Man Kim
Isaac Kim
Jae Heon Kim
Jae J. Kim
Jaeil Kim
Jae-Jin Kim
Jae-Sung Kim
James Kim
Jane Kim
Jangwon Kim
Jay Kim
Jeffrey Kim
Jennifer Kim
Jeong Do Kim
Jeong Hun Kim
Jieun Kim
Jihun Kim
Jin Hyoung Kim
Jin-Cheol Kim
Jin-Hwan Kim
Jinmi Kim

Jinoh Kim
Jinsoo Kim
Jinwoo Kim
Jiwon Kim
Jiyeun Kate Kim
John Kim
Jong Kim
Jonghwan Kim
Jong-Myong Kim
Jongphil Kim
Jonny Kim
Joohan Kim
Joo-Hwan Kim
Joomyeong Kim
Joosup Kim
Joseph Kim
Jun Su Kim
Jung Hoon Kim
Jungsu Kim
Jung-Whan Kim
Junheon Kim
Jun-Mo Kim
Juyong Kim
Kee-Hong Kim
Kevin Kim
Ki Hong Kim
Ki Woo Kim
Kwang Pyo Kim
Kwang Soo Kim
Kyeong Kim
Kyeong Soo Kim
Kyeong-Man Kim
Kyoungtae Kim
Kyujung Kim
Kyung Bo Kim
Kyungho Kim
Kyung-Jin Kim
Kyungmin Kim
Leo Kim
Lou Kim
Man-Su Kim
Man-Sun Kim
Mee Kim
Mi Na Kim
Michelle M. Kim
Min Hwan Kim
Min Su Kim
Mincheol Kim
Minjae Kim
Moon Young Kim
Moon-Soo Kim
Myeung Kim
Nam Chul Kim
Nam-Gyoon Kim
Namhee Kim
Nayoung Kim
Peter Kim
Philip Kim
Pil Joo Kim
Rakhyun Kim
Reuben Kim
S. T. Kim
Sang Gyune Kim
Sang Kim
Sang Wan Kim
Sang Woo Kim
Sang-Jin Kim
Sangyun Kim
Sejoong Kim
Seok-Jo Kim
Seong-Gon Kim
Seongho Kim
Seonghwan Kim
Seong-Jang Kim
Seong-Kyu Kim
Seul Ki Kim
Seung-Jae Kim
Shin-Hee Kim
Simon Kim
Soh Kim
Soo Young Kim
Soyeon Kim
So-Youn Kim
Soyoung Kim
Stephen Kim
Suil Kim
Sun Kim
Sun Tae Kim

Sun Wook Kim
Sung Gyun Kim
Sung Joon Kim
Sung-Hoon Kim
Sung-Jin Kim
Sung-Kun Kim
Sunhong Kim
Sunjung Kim
Sunmi Kim
Susan Kim
Suwon Kim
Tae Gwan Kim
Tae Hoon Kim
Tae Il Kim
Tae Jin Kim
Tae Kim
Tae Kyoung Kim
Taeho Kim
Taeyoon Kim
Tai-Gyu Kim
Ungsoo Kim
W. Kim
Wangdo Kim
Won H. Kim
Won Kim
Wonjoon Kim
Woo Ho Kim
Wooju Kim
Yangha Kim
Yanghee Kim
Yeonjoo Kim
Yong Jin Kim
Yong Soo Kim
Yonggyun Kim
Youngchul Kim
Young-Mee Kim
Youngseek Kim
Yun Gi Kim
Yun Kyung Kim
Yungil Kim
Yuri Kim
Peter Kima
Miho Kimachi
Jeffrey Kimbrel
Kevin Kimbro
Chava Kimchi-Sarfaty
Ismael Kimirei
Yoshifumi Kimizuka
Bruce Kimler
Alan Kimmel
Martin Kimmel
Joachim Kimmerle
Gretchen Kimmick
Jin Kim-Mozeleski
Michelle Kimple
Katrina Kimport
Atsushi Kimura
Dai Kimura
Daisuke Kimura
Fuminori Kimura
Hideo Kimura
Hiroaki Kimura
Hiroshi Kimura
Kazuhiro Kimura
Koutarou Kimura
Li Kimura
Nobuyuki Kimura
Shinya Kimura
Tadashi Kimura
Takeshi Kimura
Tomoki Kimura
Tsuyoshi Kimura
Yasumasa Kimura
Shinichi Kinami
Rebecca Kinaston
Paul Kinchington
Robert Kinders
Joseph Kindler
Bethia King
Brett King
Carina King
Charlotte King
Daniel King
David King
George King
Georgina King
Gwendalyn King
James King

Jean-Rémi King
Jonas King
Kylie King
Marco-Felipe King
Mark King
Meagan King
Tamara King
Tania King
Turi King
Fred Kingdom
Mila Kingsbury
Richard Kingsford
David Kingsley
J. Derek Kingsley
Michael Kingsley
Robert Kingsley
Tigga Kingston
Takeshi Kinjo
Tim Kinnaird
Allison Kinney
Ulla-Mari Kinnunen
Noriyuki Kinoshita
Takamasa Kinoshita
Kenichiro Kinouchi
Osame Kinouchi
J. Kinscher
Joanna Kinsey
Rochelle Melina Kinson
Tamara Kinzer-Ursem
Markus Kipp
Franz Király
Orsolya Király
Tara Kiran
Amy Kirby
Edward Kirby
James Kirby
John Kirby
Lynn Kirby
Russell Kirby
Sarah Kirby
Tyler Kirby
Martin Kircher
Karin Kirchgatter
Anne Kirchhoff
Ann Kirchmaier
Miranda Kirchner
Theresa Kirchner
Mehmet Kirgiz
Mikhail Kirillin
Diana Kirilovsky
Girish Kirimanjeswara
Kohtaro Kirimura
Yohei Kirino
Christian Kirisits
Adam Kirk
Jonathan Kirk
Mark Kirk
Steve Kirkby
Robert Kirkcaldy
Carsten Kirkeby
Karla Kirkegaard
Grant Kirker
Lea-Ann Kirkham
M. Kirkham
Pertti Kirkinen
Emrys Kirkman
Jamie B. Kirkpatrick
Andrea Kirmaier
Antonis Kirmizis
Knut Kirmse
Thomas Kirn
Reinhard Kirnbauer
Gilbert Kirouac
George Kirov
Ilya Kirov
Ivan Kirov
Yonal Kirsal
Kirill Kirsanov
Alexander Kirsch
David Kirsch
Jordan M. Kirsch
Louise Kirsch
Max Kirsch
Thorsten Kirsch
Dan Kirschner
Lawrence Kirschner
Joseph L. Kirschvink
Gary Kirsh

Frank Kirstein
Janine Kirstein
Lars Kirsten
Lech Kirtiklis
Olivia Kirtley
Anna Kis
Kai Kisand
Vladimir Kiselev
Katja Kiseljak-Vassiliades
Kirill Kiselyov
Laszlo Kish
Amar U. Kishan
Takuya Kishi
Taro Kishi
Yoshimi Kishimoto
Dmitry Kishkinev
Nand Kishore
Sandeep Kishore
Roman Kislov
Andor Kiss
Anna Karolina Kiss
Antal Kiss
Erzsébet Kiss
Hubert Kiss
János Kiss
Levente Kiss
Zelma Kiss
David Kissane
William Kisseberth
Alexei Kisselev
Grace Kissling
Dinant Kistemaker
Peter Kistler
Anna Kistner
Zoltan Kisvarday
Anthony Kiszewski
Gabor Kiszler
Hirohito Kita
Yosuke Kita
Hiroshi Kitagawa
Kaori Kitagawa
Masashi Kitagawa
Noriyuki Kitagawa
Yoshiaki Kitaguchi
Cari Kitahara
H. Kitahata
Keiichi Kitajo
Kenichiro Kitamura
Shumpei Kitamura
Yoshihisa Kitamura
Akio Kitao
Azusa Kitao
Yasushi Kitaoka
Yuichi Kitasako
Mark Kitchen
Scott Kitchen
James Kite
Maja Kitic
Elizabeth A. Kitsis
Sebastian Kittelmann
Rick Kittles
Mark Kittleson
Otomar Kittnar
Christopher Kitts
Nupur Kittur
Osmo Kivinen
Yogev Kivity
Noah Kiwanuka
Nam Kiwoong
Charles Kiyaga
Eugene A. Kiyatkin
Takako Kizaki
Erhan Kiziltan
Merel Kızıltan
Laura Kofoed Kjær
Anette Kjeldsen
Barbro Kjellstrom
Olav Sigurd Kjesbu
Rasmus Kjobsted
Rasmus Kjøller
Corné Klaassen
Rianne Klaassen
Z. Klaassen
Eline Klaassens
Cheryl Klaiman
Riina Klais
Jeffrey Klann
Oya Klanten

Sarah Klapproth
Eva Klaric
Philipp Klaritsch
George Klarmann
Zachary Klase
Lisa Klasson
Nichole Klatt
Nancy Klauber-DeMore
Jeffery Klauda
Katy Klauenberg
Christoph Klauer
Jeffrey Klausner
Richard Klavans
Sonja Klebe
Marcus Kleber
J. Klećkowska-Nawrot
Leszek Kleczkowski
Pawel Kleczynski
Silke Klee
Jorg Kleeff
Onno Kleen
Melvin Klegerman
Evan Kleiman
Dana Kleimeier
Arnaud Klein
Cameron Klein
Carina Klein
David M. Klein
Emily Klein
Hannah Klein
Jens Klein
Kerstin Klein Klein
Laura Klein
Marlise Klein
Nadav Klein
Nicholas J. Klein
Peter Klein
Richard Klein
Teresa Klein
Wilfried Klein
Christoph Kleineidam
David Kleiner
Aleysia Kleinert
Miriam Klein-Flügge
Marisa Klein-Gitelman
Adam Kleinman
Hynda Kleinman
Mark Kleinman
Iivari Kleino
Sarah Klein-Radukic
Christoph Kleinschnitz
Kristin Kleisner
Christian Kleist
Ingo Kleiter
Daniel Klem
Zalika Klemenc-Ketis
Reija Klemetti
Samuel Klempner
Riku Klen
Paulina Kleniewska
Aleksandr Klepinin
Ekaterini Klepousniotou
Joerg Klepper
Elizabeth Klerman
Hilit Kletter
Arnulf Kletzin
Thomas Kleyman
Reinhold Kliegl
Vaclav Klika
Harvey Kliman
Jan Klimas
Peter Klimek
Yann Klimentidis
Thomas Klimkait
Sophie Klimscha
Christopher Kline
Daniel Kline
David Kline
David Kling
Juliana Kling
Matthew Kling
Karsten Klingberg
Torkel Klingberg
Petra Klinge
Karin Klingel
Jon Klingensmith
Martin Klingenspor
Elizabeth Klings

Don Klinkenberg
Dennis Klinman
Anna Klintsova
Amira Klip
Alexander Klishko
Brian Kliszczewicz
Janne Klitgaard
Bruce Klitzman
Marko Klobucar
Michael Klockmann
Kimberly Klock-Moore
Andrzej Kloczkowski
Casey Klofstad
Houke Klomp
Bernard Klonjkowski
Kimberly Klonowski
Włodzimierz Klonowski
Helmut Kloos
Robert Klopfleisch
Christophe Klopp
Karl Klose
Steven Klosterman
Luisa Klotz
Daniel Kluess
Bryan Kluever
Achim Klug
E. Q. Klug
Stefanie Klug
Tejs Klug
Michael Kluge
Avraham Kluger
Michael D. Kluger
Georg Klump
Katja Klumpp
Stefan Klumpp
Glenn Klute
Igor Klyubin
Julie Kmec
Livia Kmetzsch
Eric Kmiec
Sebastian Kmiecik
Antti Knaapila
Christine Knabe
Guido Knapp
Michael Knapp
Stefan Knapp
Susanne Knappe
Steven Knapper
Peter Knappett
Ewelina Knapska
Brian Knarr
Shirley Knauer
Claude Knauf
Beat Knechtle
Matthias Knefel
Henderikus Knegtering
Mark A. Knepper
Paul Knepper
Jeff Kneteman
Wayne Knibb
Ulrich Knief
Kali Kniel
Benjamin Knier
Alec Knight
Alissa Knight
David Knight
Joseph Knight
Julia Knight
Marian Knight
Robert Knight
Vincent Knight
Cathy Knight-Agarwal
Chris Knightes
Jennifer Knight-Madden
Brian Knights
Maria Knikou
Mikael Knip
Duleeka Knipe
Peter Knipe
Corina Knipper
Uwe Knippschild
Andrea Knittel
Matthias Knobe
Jürgen Knobloch
Greg Knock
Michael Knoflach
Mirjam Knol
Fabien Knoll

Maximilian Knoll
Thomas Knopfel
Aliah Knopff
Michael Knopp
Craig Knott
Nathan Knott
Verner Knott
William Knowler
Helen Knowles
John Knowles
Joshua Knowles
Jessie Knowlton
Justin Knox
Ron Knox
Susan Knox
Anders Knudby
Jakob Knudsen
Lars Knudsen
Thomas Knudsen
Cory Knudson
Kathleen Knudson
Andrew Knutsen
Benjamin Ko
Chang-Yong Ko
Ching-Chang Ko
Fung Chi Ko
Gladys Ko
Hsin-Kuo Ko
Hyang Mi Ko
Hyun-Jeong Ko
Ja Kyong Ko
Kisung Ko
Mei-Chuan Ko
Po-Yen Ko
Seong-Gyu Ko
Wen-Chien Ko
Young-Gyu Ko
Hiroyuki Koami
Robert Kob
Jarosław Kobak
Hitoshi Kobata
Donald Kobayashi
Ichiro Kobayashi
Katsuhiro Kobayashi
Lindsay Kobayashi
Masahiro Kobayashi
Masaki Kobayashi
Susumu Kobayashi
Tamaki Kobayashi
Tatsuya Kobayashi
Tetsuo Kobayashi
Tetsuro Kobayashi
Stefan Köberich
Rudi Kobetic
Tomasz Kobiela
Christopher Kobierzycki
Gary Kobinger
Stephan Koblmüller
Dylan Kobsar
Lester Kobzik
Atakan Koc
Cetin Koc
Mustafa Koc
Yavuz Koca
Rüya-Daniela Kocalevent
Husniye Kocasarac
Jan Kocbach
Simon Kocbek
Ayhan Kocer
Zeynep Kocer
Alexander Koch
Flávia Koch
Frank Koch
Holger Koch
Jennifer Koch
Marcus Koch
Marianne Koch
Michael Koch
Paul Koch
Philipp Koch
Sabine Koch
Sheryl Koch
Thea Koch
Wayne Koch
Jacob Kocher
Thomas Kocher
Sonali Kochhar
Lazaros Kochilas

Karen Kochis-Jennings
Bose Kochupurakkal
Juraj Koci
Matthew Koci
Robin Köck
Samuel Kocoshis
Chris Koczar
Yusaku Kodaka
Takahiro Kodama
Rahul Kodgule
Jordan Kodner
Dalibor Kodrik
Krithika Kodumudi
Smitha D. Koduru
Srinivas Koduru
Anson Koehler
Karl Koehler
Karsten Koehler
Linda Koehler
Till Koehne
Michael Koelch
Bobby Koeleman
Thomas Koelewijn
Katja Koelkebeck
Philipp Koellinger
Gijsje Koenderink
Stephanie Koenemann
Alexander Koenig
Amanda Koenig
Christoph Koenig
Christopher Koenig
Renate Koenig
Sven Koenig
Nikolaus Koeniger
Marcel Koenigkam Santos
Constantianus Koenraadt
Cristian Koepfli
Roger Koeppe
Arnulf Koeppen
Katja Koeppen
Alessandro L. Koerich
Leonardo Koerich
Christian Koerner
Oliver Koerner
Sally Koerner
Henk Koerten
Juergen Koessler
Benjamin Koester
Ad Koets
Joris Koetsveld
Matthew Kofron
Paulo Kofuji
Fumitaka Koga
Hironori Koga
Jun-Ichiro Koga
Kazuo Koga
Kenichi Koga
Masatoshi Koga
Alexis Coulourideskogan Kogan
Felix Kogan
Donat Kögel
Michael H. Kogut
Michael Kogut
Tehila Kogut
Dong-Hee Koh
Hee-Jong Koh
Jae-Young Koh
Mariko Koh
Sehwon Koh
Michel Kohl
Michael Köhl
Florent Kohler
Pamela Kohler
Ole Kohler-Forsberg
Richie Kohli
Sebastian Kohlmann
Kevin Kohlstedt
Rachel Kohn
Taylor Kohn
Thomas Kohnen
Takashi Kohno
Bruce Kohorn
Susy Kohout
Mark Kohr
Kenjiro Kohri
David Kohrman
Stephen Kohut
Ayato Kohzu

Suehiro Koichi
Masanori Koide
Roger Koide
Masato Koike
Shinsuke Koike
Takahiko Koike
Tatsuya Koike
Teruhiko Koike
Rajeshwari Koilkonda
Diwa Koirala
Matti Koivula
Jussi Koivumäki
Antti Koivusalo
Hisashi Koiwa
Akio Koizumi
Tomonobu Koizumi
Georg Kojda
Milan Kojic
Hideto Kojima
Soichi Kojima
Takashi Kojima
Ilpo Kojola
Maria Kojoukhova
Ellen Kok
Rob Kok
Sang-Heng Kok
Victor Kok
Wouter Kok
Eric Koka
Lauren Kokai
Juline Koken
Visnja Kokic
Tarja Kokkola
Gyöngyi Kökönyei
George Kokotos
Hiroki Kokubo
Daisuke Kokuryo
Gerasimos Kolaitis
Andrew Kolarik
Joseph Kolars
Alexander Kolb
Andreas Kolb
Bryan Kolb
Rosana Kolb
Michael Kolber
Roman Kolcun
Jeppe Kolding
Martin Kolditz
Ryszard Kole
John Kolega
Evangelos Kolettas
Hemanta Koley
Nitin Kolhe
Angelos Kolias
Vassilis Koliatsos
George Kolios
Roxy Koll
Anup Kollanoor Johny
M. H. Kollef
Martin Koller
Barbara Kollerits
Florian Kollert
Panagoula Kollia
Giorgos Kollias
Laxmikanth Kollipara
Kenneth Kollman
Markus Kollmann
Martin Kollmar
Marlene Kollmayer
Kathrin Kollndorfer
Afsal Kolloli
Bradley Kolls
María de los Angeles Kolman
Jane Kolodinsky
Alex Kolodkin
Michalina Kołodziejczak
Anatoly Kolomeisky
Dmitry Kolomenskiy
Attila Kolonics
Efi Koloverou
Allison Kolpas
Anne-Brit Kolstø
Tomas Koltai
Ismail S. Koltas
Ida Kolte
Walter Koltun
Aleksandra Kołtuniuk
Y. Komai

Volodymyr Koman
Marcus Komann
John Komar
Paul Komesaroff
Barry Komisaruk
Andrey Komissarov
Takefumi Komiya
Ricardo Komotar
Tom Kompas
Michihiro Kon
Valentina Kon
Koffi Konan
Jürgen Konczalla
Kelika Konda
Ganesh Kondabattula
Divya Kondaveeti
Elias Kondilis
Kazuhiro Kondo
Naoshi Kondo
Rumi Kondo
Takahisa Kondo
Takashi Kondo
G. Kondolf
Daniel Kondor
Boris Kondratieff
Krishnamurthy Konduru
Nagarjun Konduru
Evangelos Kondylis
Apurba Koner
Richard Kones
Suma Konety
Amanda Kong
Anthony Kong
Chung Yin Kong
Dexing Kong
Eunjung Kong
Fabian Kong
Feng Kong
Heidi Kong
Il-Keun Kong
In Deok Kong
Jiang-Ti Kong
Jianqiang Kong
Ka-Yiu Edwin Kong
Lan Kong
Lingrang Kong
Lisheng Kong
Qingke Kong
Qiusheng Kong
Shengchun Kong
Shu Kong
Wei Kong
Weijun Kong
Xiangfeng Kong
Xiangjie Kong
Xiangrong Kong
Yazhuo Kong
Ying Kong
Yingzhen Kong
Ane Konglund
Alice Kongsted
Jakob König
Matthias König
John König Walles
Stefan Konigorski
Ruud H. Koning Koning
Hirotaka Konishi
Hiroyuki Konishi
Yutaka Konishi
Dean Konjević
Prasad Konkalmatt
Kotaro Konno
Satoshi Konno
Masahiro Kono
Reiko Kono
Tadeusz Kononowicz
Agneszka Konopka
Witold Konopka
Marina Konopleva
Nicolai Konow
Daniel Konrad
Franziska Konrad
Thomas Konrad
Ilze Konrade
Tania Konry
Mary Konsolaki
Eleni Konstantinou
George Konstantinou

Georgios Konstantinou
Aris Konstantopoulos
Tsuneo Konta
Maria Konte
Eleftherios O. Kontis
Evangelos Kontopantelis
Panagiota Kontou
Dimitris Kontoyiannis
Irina Kontsevaya
Kevin Konty
Ozlen Konu
Kishori Konwar
Brian Koo
Hyeon-Sook Koo
Hyung-Jun Koo
Malcolm Koo
Shahriar Koochekpour
Elisabeth Kooi
Katherine Kooij
Bas Kooijman
Lammert Kooistra
Wouter Kool
Sebastiaan Koole
Jeroen Kooman
Hon Wai Koon
Karanjit Kooner
Gerrit Koopman
Heather Koopman
Peter Koopman
René Koopman
Klaas Pieter Koopmans
Maarten Koornneef
Krisztián Koós
Tibor Koós
Gerritt Jan Kootstra
Neeltje Kootstra
David Kooyman
Dara Koozekanani
Ian Kopacka
Doris Kopahnke
Raphael Kopan
Daniel Kopans
Alexander Kopatz
Ashley Kopec
Karl Kopiske
Benjamin Kopp
Marcel Kopp
Matthias Kopp
Robert Kopp
Steven Kopp
Ulla Kopp
Zoe Kopp
Erling Koppang
Janna Koppe
Kadri Koppel
Daniël Koppenol
Germán Kopprio
Dorota Kopycka-Kedzierawski
Bato Korac
Abhilash Koratala
Ioanna Korbila
Christopher Korch
Irina Koreen
Ludek Koreny
Bruce Korf
Martina Korfei
Dieneke Korfker
Mayuresh Korgaonkar
Netanel Korin
Prakash Koringa
Gilly Koritzky
Mustafa Korkmaz
Selcuk Korkmaz
James Korkola
Christoph Korn
Anna Kornadt
Thomas Kornberg
Diana Kornbrot
Joe Kornegay
Jelena Kornej
Mirjam Körner
Daniel Kornitzer
Birgitte Kornum
Igor Korobiichuk
Andrey Korotayev
Konstantin Korotenko
Konstantin Korotkov
Irina Korshunova

Ron Korstanje
Sharina Kort
Insa Korten
Raine Kortet
Todd Korthius
Ronald Korthuis
Trond Kortner
Marcin Kortylewski
Christian Korunka
M. Korzeniowska
Viktor Korzun
Bor Kos
Ivan Kos
Randall Kosaki
Yutaka Kosaki
Tiffany Kosch
Jessica Koschate
Kivanc Kose
Samet Kose
Klemen Koselj
Igor Kosevich
Sentaro Koshida
Shigeyuki Koshikawa
Taka-Aki Koshimizu
Maciej Kosilo
Mary Kosir
Jarkko Koskela
Sanna Koskela
Alissa Koski
Renee Koski
Sonja Koski
Markku Koskinen
Sanna Koskiniemi
Paul Kosma
Ralf Kosma
Arkadiusz Kosmala
Daniel Kosman
Cassandra Kosmidou
Georgia Kosmopoulou
Klára Kosová
Catherine Koss
Anthony Kossiakoff
Michael Kossmeier
Eric Kossoff
Manoelle Kossorotoff
Gerald Kost
Martin Košťák
Zvonko Kostanjcar
Anna Kostareva
Thomas Kosten
Andreas Koster
Moritz Köster
Oleg Kosterin
Mirjana Kostic
Deliana Kostova
Petya Kostova
Viviane Kostrubiec
Alexei Kostygov
Maxim Kostylev
Lidia Kostyniuk
Alla Kostyukova
Ondřej Košulič
Pallav Kosuri
Jacek Kot
Vitas Kotagal
S. Kotake
Hema Kotamarthi
Hafsat Kotangora
B. Kotchoubey
Ekaterina Kotelnikova
Atul Kothari
Rashmi Kothary
Karl Kothbauer
Ute Kothe
Jerzy Kotlinowski
Norihiko Kotooka
Giorgios Kotoulas
Alexander Kotov
Georgios Kotsakis
Kornelia Kotseva
S. Kotsiantis
Joanne Kotsopoulos
Nikolaos Kotsopoulos
Dimitra Kotsougiani
Nilwala Kottegoda
Mark Kotter
Shyamasundaran Kottilil
Jan Kottner

Jack Kottwitz
Katarzyna Kotulska
Dimitrios Kotzinos
Gang Kou
Junping Kou
Yongping Kou
Zhaohui Kou
Marek Kouba
Georgios Koubouris
George Koufos
Ohnuki Kouichirou
M. Koukourakis
Hira Koul
Albert Koulman
Markku Koulu
Lefteris Koumakis
Ilias Kounatidis
Natalay Kouprina
Taxiarchis Kourelis
Stella Kourembanas
Lambrini Kourkouta
Valerie Kouskoff
Konstantin Kousoulas
Yiannis Koutalos
Michael Koutsilieris
Aikaterini Koutsou
Kostas Koutsoumanis
Tanja Kouwenhoven
Roger Kouyos
Fiona Kouyoumdjian
Abdellah Kouzou
Andrej Kovac
Dejan Kovac
Lubomir Kovac
Borut Kovačič
Andrea Kovacs
Gabor Kovacs
Laszlo Kovacs
Norbert Kovacs
Peter Kovacs
Richard Kovacs
Sandor Kovacs
Stephanie Kovacs
Mihály Kovács
Andriy Kovalenko
Ilona Kovalszky
Petri Kovanen
Martina Kovarova
Yulia Kovas
Zsuzsanna Kövi
Gennadiy Kovtunovych
Irina Kovyazina
Marek Kowal
John Kowalchuk
Piotr Kowalczuk
Michał Kowalczyk
Michal Kowalewski
Bernd Kowall
Justyna Kowalska
Luiz Paulo Kowalski
Alicia Kowaltowski
Markus Kowarik
M. Kox
Daisuke Koya
Eiki Koyama
Hiroshi Koyama
Sachiko Koyama
Tatsuki Koyama
Teruhide Koyama
Tomotsugu Koyama
Toshihiro Koyama
Yoshimasa Koyama
Kazuo Koyanagi
Robert Koza
Sergeja Kozar
Richard Kozarek
Thomas Kozel
Iryna Kozeretska
Tamas Kozicz
Jacek Koziel
Joanna Koziel
Dariusz Koziorowski
Kasia Kozlowska
Hanna Kozłowska
Dariusz Kozlowski
Lynn Kozlowski
Piotr Kozlowski
Steve Kozlowski

Steven Kozlowski
Jeff Kozma
Ivan Kozyatnyk
Anita Kozyrskyj
Vivian Kraaij
A. O. Kraaijeveld
Wilbur Kraak
Casper Kraan
Claudine Kraan
Sören Krach
Joep Kraeima
Fredric Kraemer
Friederike Kraemer
Roland Kraemer
Nicolle Kraenkel
Oliver Kraff
Peter Kraft
Stephanie Kraft
Gordon Kraft-Todd
Christian Krägeloh
Birthe Kragelund
Kasper Kragh
Rüdiger Krahe
Thomas Krahe
Scott Krahl
Andrew Krahn
Thomas Krahn
Peter Kraiczy
Dimitri Krainc
Maja Krajinovic
Joel Krajl
John Krakauer
Michelle Krakowski
Miroslav Králik
Martina Kralinger
Susan Kralisch
Simona Kralj-Fiser
Rodger Kram
Andreas Kramer
Andrew Kramer
Daniel Kramer
H. Kramer
Ina Kramer
Jill Kramer
Joel Kramer
John Kramer
Jos Kramer
Mark Kramer
Patricia Kramer
Robin Kramer
Sarah Kramer
Susanne Kramer
Wolfgang Kramer
Bernhard Krämer
Irene Krämer
Stefanie Krämer
Ursula Krämer
Andrei Kramerov
Ralf Krampe
Günter Krampen
Kamil Kranc
Olve Krange
Branka Kranjc
Katharina Kranzer
Sven Krappmann
Allan Krasnik
Irina Krasnova
Agata Krasny-Pacini
Tatiana Krasova Wade
Tal Krasovsky
Ines Krass
Marco Krasselt
Andrei Krassioukov
Lukas Kratochvil
Jadel Kratz
Robert A. Kratzke
Helen Kraus
Johann Kraus
Kornelius Kraus
Thomas Kraus
Virginia Kraus
Annalinda Krause
Daniel Krause
Douglas Krause
Duncan Krause
Jens Krause
Jesse Krause
Mauricio Krause

Niklas Krause
Roland Krause
Allen Kraut
Stefanie Krauth
Andra Krauze
Richard Krauzlis
Len Kravitz
Richard Kravitz
Katarzyna Krawczyk
Marcin Krawczyk
Emanuel Krebs
Paul Krebs
Robert Krebs
Ruth Krebs
Lazlo Kredics
Cory Krediet
Paul Krediet
Pamela Kreeger
Jan-Ulrich Kreft
Richard B. Kreider
Richard Kreider
Vladik Kreinovich
Barry Kreiswirth
Martyna Krejmer-Rabalska
David Kreling
Peter Krell
Claire Kremen
Colin Kremer
Patricia Kremer
Jan Kremers
Christian Kremser
Waldemar Kremser
Mira Krendel
Oliver Krenkel
Miroslav Krepl
Clemens Krepler
Eckhard Krepper
W. John Kress
Dieter Kressler
Irene Kretchy
Alexander Kretschmer
Dorothee Kretschmer
Alexandra Kretz
Colin Kretz
Markus Kretz
Jutta Kretzberg
Benno Kreuels
Gunter Kreutz
Rolf Kreutz
Carin Kreutzer
Susanne Kreuzer-Redmer
Robert Kreuzig
Jürgen Kreuzwieser
Sebastian Krey
Skirmantas Kriaucionis
Alison Kriegel
Nancy Krieger
Johannes Krietsch
Steven Krilis
Michael Krings
Helga Krinzinger
Murali Krishna
Sitaraman Krishna
Sudhir Krishna
K. Krishnakumar
Mathangi Krishnakumar
Janarthanan Krishnamoorthy
Raghu Krishnamoorthy
Srikanth Krishnamoorthy
Vijay Krishnamoorthy
Narayanan Krishnamurthi
Karthik Krishnamurthy
Krishna Krishnamurthy
Prasanna Krishnamurthy
S. L. Krishnamurthy
Ananthanarayan Krishnan
Jaya Krishnan
Krishnamoorthy Krishnan
Natraj Krishnan
Nithya Krishnan
Rathinasamy Krishnan
S. S. Nagamuthu Krishnan
Shilpa Krishnan
Unni Krishnan
Suchitra Krishnan-Sarin
Rathika Krishnasamy
Aparna Krishnavahjala
Ghattu Krishnaveni

Peter Krisper
Amy Krist
Kristian Kristansen
Anders Kristensen
Bjuul Kristensen
Gunnar B. Kristensen
Hanne Kaae Kristensen
Michael Kristensen
Peter Kristensen
Solvejg Kristensen
Søren Kristensen
Magnús Kristjánsson
Katalin Kristof
Ladislav Kristoufek
Tamas Krisztin
Leonard Kritharides
Michael Kritzer-Cheren
Jürgen Kriwet
Olga Krizanova
Istvan Krizbai
Pavla Krizova
Magdalena Krochmal
Aleksandra Kroemeke
Marijn Kroes
Silke Kröger
Jesper Krogh
Paul-Henning Krogh
Michelle Krogsgaard
Regina Krohn
Matthias Kroiss
Fernanda Kroker
Miltiadis Krokidis
Jessica Krok-Schoen
A. Krol
Mariusz Król
Alejandro Krolewiecki
Greg Kroliczak
Hans Kromhout
Volker Krömker
Berhard Kromp
Irving Kron
Michael Kron
Andreas Kronbichler
Michael Krone
Pat Krone
Madelon Kroneman
Amy Kronenberg
Thales Kronenberger
Ian Kronish
Chanoch Kronman
Brent Kronmiller
Erna Kroon
Jan Kroon
Heather Kropp
Leonie Krops
Emily Kroshus
Aleksandra Krstic
Milena Krstić
Sonja Krstin
Konstanze Krueger
Martina Krueger
Thilo B. Krueger
Jan Kruetzfeldt
Andrew Krug
Laurie Krug
Annie Kruger
Karen Kruger
Peter Kruger
Ruan Kruger
Alejandra Krüger
Nadine Krüger
Frithjof Kruggel
Alexey G. Kruglov
Andrey Kruglov
Tamar Krugman
Nicole Kruh-Garcia
Anne-Wil Kruijt
Boudewijn Kruithof
Marcos Krull
Katrin Krumbholz
Carol L. Krumhansl
Kira Krumhansl
Ralf Krumkamp
Joseh Krummenache
Zuzana Krumpalova
Tanja Krumpe
Martin Krupa
Yanna Krupnikov

Daniel Krupp
Joshua Krupp
Anthony Krus
Kevin Kruse
Dean Krusienski
Germán Kruszewski
Jean-Pierre Kruth
Christina Kruuse
Frank Kruyt
Konstantin Krychtiuk
Annedie Krygsman
Angelos-Miltiadis Krypotos
Andriy Kryshtafovych
Alexey Kryukov
Urszula Krzych
Wirginia Krzysciak
Varun Kshettry
Krzysztof Ksiazek
Aneta Książek
Cheng Ku
David Ku
Kang Mo Ku
Leighton Ku
Li-Jung Ku
Namkug Ku
Chee Sian Kuan
Garry Kuan
Khing Boon Kuan
Hua Kuang
Rui Kuang
Shihuan Kuang
Yiqun Kuang
Michael Kuba
Karen Kubena
Jakub Kubiak
Lucy Kubiak Martens
Milan Kubiatko
Christian Peter Kubicek
John Kubie
Lubica Kubikova
Leszek Kubin
Terrance Kubiseski
Eri Kubo
Takeo Kubo
Takuo Kuboki
Jennifer Kubota
Kazuo Kubota
Kengo Kubota
Naoto Kubota
Ryuji Kubota
Toshiaki Kubota
Yoshiki Kubota
Stan Kubow
Haruo Kubozono
Flavia Kubrusly
Roman Kuc
Gregory Kucera
Michal Kucera
Ladislav Kuchar
Philip Kuchel
Roman Kuchta
Rachel Kuchtey
Jennie Kuckertz
Can Kucuk
Can Küçük
Fatih Küçükdurmaz
Ozlem Kucukoglu
Umut Kücüksezer
Krzysztof Kuczera
Anetta Kuczyńska
Rajat Kudchadker
Kristina Kudelko
Fumitaka Kudo
Isao Kudo
Masataka Kudo
Toru Kudo
Yasusei Kudo
Mahesh Kudrimoti
Nina Kudryashova
Alexey Kudryavtsev
Indira Kudva
Sara Kuebbing
Stefan Kuechler
Christian Kuehn
Sarah Kuehne
Claudia Kuehni
Georg Kuenze
Joshua Kuethe

Irina Kufareva
Nuworza Kugbey
Dimitris Kugiumtzis
Scott Kuhl
Hilmar Kühl
Susanne Kühl
Angela Kuhla
Björn Kuhla
Thomas Kühlein
Stella L. Kuhlmann
Tim Kuhlmann
Cynthia Kuhn
Jens Kuhn
Joachim Kuhn
Matthias Kuhn
S. Kuhn
Taylor Kuhn
Christina Kühn
Ingolf Kühn
Jens-Peter Kühn
Sonja Kühn
Nikolai Kuhnert
Gunter Kuhnle
Lucia Kuhn-Nentwig
P. P. F. M. Kuijer
Hugo Kuijf
Jonas Kuiper
Bea Kuiperij
Ernest Kuipers
M. Kuipers
Urho Kujala
Elizabeth Kujawinski
Jennifer Kuk
Akiko Kukita
Jyrki Kukkonen
Clayton Kuklick
Veerapol Kukongviriyapan
Vandna Kukshal
Maria Kukuruzinska
Peter Kulakow
Dominik Kulakowski
Anna Kula-Pacurar
Kulamakan Kulasegaram
Michel Kulbicki
Ivana Kulhánová
Alexander Kulik
Margarete Kulik
Sofya Kulikova
Goran Kuljanin
Abhaya Kulkarni
Chinmay Bhimaji Kulkarni
Mangesh Kulkarni
Manisha Kulkarni
Manjiri Kulkarni
Nachiket Kulkarni
Ritwij Kulkarni
Sanjay Kulkarni
Subhash Kulkarni
Tejaswini Kulkarni
Matthew Kulke
Skadi Kull
Sven Kullander
Sven O. Kullander
Christopher Kullenberg
Brian Kullin
Emily Kullman
Aura Kullmann
Bernard Kulohoma
John Kulp
Rebecca Kulp
Ambar Kulshreshtha
Ritu Kulshreshtha
Erik Kulstad
Arman Kulyyassov
Hye-Chung Kum
Hiromichi Kumagai
Koshi Kumagai
Naoki Kumagai
Eiichi Kumamoto
Abhishek Kumar
Addanki Kumar
Ajay Kumar
Alex Kumar
Amit Kumar
Amrendra Kumar
Anil Kumar
Arun Kumar
Ashok Kumar

Ashutosh Kumar
Ashwini Kumar
Avnish Kumar
Bijender Kumar
Chandra Kumar
Deepak Kumar
Devarai Kumar
Dhananjay Kumar
Dhiraj Kumar
Dinesh Kumar
Gulshan Kumar
Gunasekaran Kumar
Haribalan Kumar
Himanshu Kumar
Indrajit Kumar
Jatin Kumar
Kishlay Kumar
Lalit Kumar
Madhan Kumar
Manish Kumar
Manoj Kumar
Munesh Kumar
Nalin Kumar
Narender Kumar
Nathella Pavan Kumar
Neeraj Kumar
Neha Kumar
Nithin Kumar
Pankaj Kumar
Parameet Kumar
Parveen Kumar
Pradeep Kumar
Rahul Kumar
Raj Kumar
Rajesh Kumar
Ram Kumar
Ravinder Kumar
Rishikesh Kumar
Rohit Kumar
Sai Kumar
Sandeep Kumar
Sanjay Kumar
Santhosh Kumar
Santosh Kumar
Saravanan Kumar
Saurabh Kumar
Shailesh Kumar
Shaji Kumar
Shekhar Kumar
Shivesh Kumar
Soora Naresh Kumar
Subodh Kumar
Sudhir Kumar
Suresh Kumar
Sushil Kumar
Ujendra Kumar
Uttam Kumar
V. Bharath Kumar
Vijay Kumar
Vikas Kumar
Vinay Kumar
Vipan Kumar
Vivek Kumar
Vivekanandan Kumar
Yugal Kumar
Ashesh Kumar Das
Santosh Kumar T. R.
Soundar Kumara
Sangaralingam Kumaran
Dinakantha Kumararatne
Anbarasu Kumarasamy
Deepika Kumari
Neelam Kumari
Saru Kumari
Sharma Kumari
Snehlata Kumari
Giriraj Kumawat
S. Kumazaki
Lily Kumbani
Lalathaksha Kumbar
Atsushi Kume
Shinji Kume
George Kumi
Nyoman Kumiawan
Shivaani Kummar
Benjamin Kummer
Wolfgang Kummer
Emily Kumpel

Karol Kumpfer
Li Kun
Yang Kun
Jitka Kuncova
Nitesh Kunda
Kousik Kundu
Madan Kundu
Manikuntala Kundu
Pallob Kundu
Rakesh Kundu
Rita Kundu
Shilajit Kundu
Tanja Kunej
Norbert Kunert
Richard Kunert
Chih-Chun Kung
Justin Kung
Nina Kung
Sam Kung
Te-Lung Kung
Yutaka Kunimatsu
Shiho Kunimatsu-Sanuki
Masaru Kunimoto
Mie Kunio
Ken Kunisaki
Mervi Kunnasranta
Muthusamy Kunnimalaiyaan
Ajaikumar Kunnumakkara
Anton Kunst
Michael Kunst
Hans-Jörg Kunte
Krushnamegh Kunte
Emmanuel Kuntsche
Setor Kunutsor
Ripu Kunwar
Nicolas Kunz
Wolfram Kunz
Micha Kunze
Sarka Kunzova
Tina Kunz-Plapp
Anthony Kuo
Arthur Kuo
Caroline Kuo
Chao-Yang Kuo
Chian-Jue Kuo
Chi-Yun Kuo
Hann-Chorng Kuo
Han-Yueh Kuo
Hsiao-Che Kuo
I Fan Kuo
Jinn-Rung Kuo
Ko-Lin Kuo
Li-Wei Kuo
Patty Kuo
Yan-Fu Kuo
Yong-Hong Kuo
Yuan-Hung Kuo
Yu-Ting Kuo
Ling-Long Kuo-Huang
Denise I. T. Kuok
Marcelo Kuperman
Leanne Küpers
Roland Kupka
Peter Kuppens
Sundaram Kuppu
Elena Kupriyanova
Harley Kurata
Jiro Kurata
Shoichiro Kurata
Shigeru Kuratani
Gael Kurath
Jonathan Kurche
Benedek Kurdi
Lauri Kurdziel
Boris Kurganov
Biji Kurien
Thomas Kurien
Hidemi Kurihara
Walid Kuri-Harcuch
Yuichi Kurita
Daniel Kuritzkes
Satoru Kuriyama
Y. Kuriyama
Ajay Kuriyan
Brenda Kurland
Carolyn Kurle
James Kurle
Magdalena Kurnik

Marcelo Kuroda
Masahiko Kuroda
Tatsuhiko Kuroda
Yasuhiro Kuroda
Kenji Kurokawa
Manae Kurokawa
Mineo Kurokawa
Shun Kurokawa
Hiroshi Kuroki
Makoto Kuro-O
Masayuki Kurosaki
Shinichiro Kuroshima
Takeshi Kurosu
Hidehito Kuroyanagi
Shekar Kurpad
Deborah Kurrasch
Fina Kurreeman
Florian Kurschus
Bollmann Kurt
Margaret Kurth
Timothy Kurtti
Murat Kurtuncu
Ira Kurtz
Kenneth J. Kurtz
Isaac Kurtzer
Shree Kurup
Tagried Kurwie
Patrick Küry
Barret Kurylyk
Oliver Kurzai
Mateusz Kurzawski
Eric A. Kurzrock
Josip Kusak
Takehiro Kusakabe
Yuko Kusakabe
Kengo Kusano
T. Kusanthan
Marion Kusche-Gullberg
Kristina Kusche-Vihrog
Elizabeth Küsel
Akifumi Kushiyama
Azadeh Kushki
Kostadin Kushlev
Ariel Kushmaro
David Kushner
Sidney Kushner
Vitaly Kushnir
Manish Kushwaha
William Kuskin
Claudia Kusmic
Christine Kusminski
Linda Kusner
Oliver Kuss
Nataliia Kussul
Adam Kustka
Kenro Kusumi
Mariusz Kusztal
Vijay Kutala
Zoltán Kutalik
Mzia Kutateladze
Jason Kutch
Anton Kutikhin
Sebla Kutluay
Ryszard Kutner
Tiit Kutser
Christina Kuttler
Raman Kutty
Marta Kutwin
Lotta Kuuliala
Kadri Kuusemae
Maria Kuusmaa
Jelena Kuvač Kraljević
Rahul Kuver
Koichiro Kuwabara
Masanari Kuwabara
Takashige Kuwabara
Tomoyuki Kuwaki
Kazuyoshi Kuwano
Masaki Kuwatani
Thom Kuyper
Gaston Kuzamunu
Fiona Kuziga
Jakub Kuzilek
Ekaterina Kuzmina
Susan Kuzminsky
Alexey Kuznetsov
Andrey Kuznetsov
Nikolay Kuznetsov

Tatiana Kuznetsova
Duygu Kuzum
Kiyotaka Kuzushima
Charlotta Kvarnemo
Charlotte Kvasnovsky
Kristina Kvile
Laura Kvist
Sebastian Kvist
Tarja Kvist
Brian Kvitko
Bernard Kwabi-Addo
Bongseok Kwak
Cheol Kwak
Ho-Young Kwak
Jaeyoung Kwak
Sang-Soo Kwak
Shin Kwak
Suryang Kwak
Youn-Sig Kwak
Joanne Kwak-Kim
Kelvin Kwan
Lorinda Kwan
Paul Kwan
Y. H. Kwan
Awewura Kwara
Eliningaya Kweka
David Kwiatkowski
Anne Kwitek
Richard Kwizera
Boon-Chong Kwok
Hang Fai Kwok
Man Ki Kwok
Roland Kwok
Seung-Ki Kwok
Brian Kwon
Douglas Kwon
Goo-Rak Kwon
Guim Kwon
Janice Kwon
Minhyuk Kwon
Minjung Kwon
Ronald Kwon
Sung Woo Kwon
Tae-Hwan Kwon
Young Jik Kwon
Young Min Kwon
Yung-Keun Kwon
Allison Kwong
Jeff Kwong
Waldan Kwong
Yok Lam Kwong
Emmanuel Kyagaba
Nang Thu Thu Kyaw
Peter Kyberd
Samuel Kyei
Rosina Kyerematen
Line Anker Kyhn
Leena Kylanpaa
Dean Kyne
Tomita Kyoko
Natasha Kyprianou
Chris Kypridemos
A. Kyriacou
Kyriacos Kyriacou
Constantinos Kyriakis
Vasileios Kyttaris
Evan Kyzar
Ricard L. Fogues
Rosemary L. Walzem
Christian La
Jun-Ho La
Giancarlo La Camera
Antonio La Cava
Valentina La Corte
Alessio La Manna
Angela La Neve
Megan La Peyre
Barbara La Scaleia
Albert La Spada
Giuseppe La Torre
Michael Laakasuo
Anne Laarman
Majid Laassri
Luciana Labanca
Mary Labato
Vincent Labatut
John Labavitch
Jessy Labbe

Catherine Labbé
Bernard Labedan
Jeffrey Labelle
Maude Laberge
Niklaus Labhardt
Vinod Labhasetwar
Jessica Labonte
Aurore Labourel
Arthur J. Labovitz
Michel Labrecque
Nicholas Labriola
Nikolaos Labrou
Pascal Labrousse
Travis Labrum
Marcelo Labruna
Elisabeth Labruyere
Luminita Simion Labusca
Daniel Laby
Andrew Lac
J. Lacal
Pierre Lacasse
John Lacava
Norman Lacayo
Michelle Lacey
Simon Lacey
Joseph Lachance
Marc-André Lachance
Alain Lachaux
Dirk Lachenmeier
Thomas Lacher
Salil Lachke
Kenneth Lachlan
Nathan Lachowsky
Lubica Lacinova
Thomas Lacoue-Labarthe
Terri Lacoursiere-Zucchero
Christelle Lacroix
Jacques Lacroix
Sébastien Lacroix-Desmazes
María Lacruz
Lukasz Laczmanski
Nandan Lad
Hania Lada
Hanif Ladak
Dominick Laddy
Zach Ladin
Ana Ladio
Fred Lado
Istvan Ladunga
Oliver Laeyendecker
Jennifer Lafemina
Eileen Lafer
Shawn Laffan
Kevin Lafferty
Francois Lafond
Clement Lafon-Placette
Eric Lafontaine
Richard Laforest
Jean-Philippe Lafrance
William Lafuse
Robert Lafyatis
Erwann Lagabrielle
Neil Lagali
Alessandro Laganà
Antonio Simone Laganà
Jérôme Laganière
Julien Lagarde
Alfonso Lagares
Joao Lagarto
Linda Lagasse
Laurel Lagenaur
Anton Lager
Kelly Lager
Ilias Lagkouvardos
Francesco Lagona
Anand Lagoo
Carlos Lago-Pena
Mark Lagrimini
James Lagro
Mateus Laguardia-Nascimento
Pablo Laguna
D. Lagzdins
Tamara Lah
Chandrakant Lahariya
Ian Lahart
Thomas Lahaye
Ismail Laher
Vicente Lahera

Amiya Kumar Lahiri
Tanaya Lahiri
Esmah Lahlah
Glenn Lahodny
Chloé Lahondère
Lies Lahousse
Leo Lahti
Marius Lahti
Chengguang Lai
Chien-Chen Lai
Chih-Cheng Lai
Chih-Ho Lai
Ching Jung Lai
Ching-Huang Lai
Ching-Shu Lai
Daniel Lai
Edward Lai
Florence Lai
Gabriel Lai
Hong-Chang Lai
Hung-Cheng Lai
Hung-Wen Lai
Ivan Lai
J. M. Lai
Jennifer T. Lai
Jung-Nien Lai
Kefang Lai
Kuan-Lin Lai
Lucy Lai
Ming-Derg Lai
Nai Ming Lai
Olimpia Lai
Peggy S. Lai
Rachel Lai
Roberto Lai
Shih-Wei Lai
Tai-Shuan Lai
Te-Jen Lai
Timothy Lai
Xinan Lai
Xudong Lai
Yen Chun Lai
Yen-Chein Lai
Yongxiu Lai
Yu-Shu Lai
Yu-Wen Lai
Evagelia Laiakis
Deborah Laible
Kaitlin Laidlaw
Samantha Lain
Elodie Laine
Ingrid Laing
Richard Laing
Luigi Laino
Ines Lains
Alessandro Laio
Barry Laird
David Laird
Yvonne Laird
Richard Laird IV
Ite Laird-Offringa
Triin Laisk-Podar
Elmar Laistler
Outi Laitinen-Vapaavuori
Hannele Laivuori
Daniel Lajeunesse
Dennis Lajeunesse
Kate Lajtha
Timo Lajunen
Seema Lakdawala
Douglas Lake
Evelyn Lake
Janice A. Lake
Jennifer Lake
Jessica Lake
Stephanie Lake
Daniël Lakens
Yihunie Lakew
Laila Lakhal
Shaheen Lakhan
Sundeep Lakhtakia
Steven Lakin
Cynthia Lakon
Imayavaramban Lakshmanan
Karthik Lakshmanan
Premkumar Lakshmanane
Kamakshi Lakshminarayan
Anisha M. Lal

Aparna Lal
Ashish Lal
Chandra Shekhar Lal
Geeta Lal
Giridhari Lal
Hind Lal
Rup Lal
Shailesh Lal
Sham Lal
Rahmi Lale
Maria Lalioti
Kristi Lall
Marek Lalli
Marie Lallier
Andrew Lalo
Jacques-Olivier Laloë
Patricia Lalor
Alexis Lalouette
Samuel Lalronunga
Carson Lam
Daniel Tai Pong Lam
Grillo Lam
Hilaire Lam
Jessica Lam
Kathy Lam
Metthew Lam
Phung Lam
Shu Lam
Tai Ning Lam
Tommy Lam
Wei-Haur Lam
Dilraj Lama
Angela Lamarca
Nathalie Lamarche-Vane
Laurent Lamarque
Alain Lamarre
Bruno Lamas
Audrey Lamb
Benjamin Lamb
Clayton Lamb
Don Lamb
Roos Lambalk
Simon Lambden
Barrot H. Lambdin
Fernand Lambein
Anne-Marie Lambeir
Amy Lambert
Ian Lambert
James Lambert
Lauren Lambert
Olivier Lambert
Shea Lambert
Sidonie Lambert-Niclot
Poppy Lamberton
Pia Lamberty
Lauren Lambie-Hanson
Philippe Lambin
Christine Lambkin
Marcel Lambrechts
Petar Lambrev
David Lambright
Pablo Lamelas
Regina Lamendella
Mariusz Lamentowicz
Daniel Lametti
Raphael Lami
Gyanu Lamichhane
Saneer Lamichhane
Santosh Lamichhane
Sangeet Lamichhaney
Todd Lamitina
Gert Jan Lammers
Joris Lammers
Adriaan Lammertsma
Patrick Lammie
Dudley Lamming
Elise Lamont
Anthony Lamontagne
Elizabeth Lamos
Frederic Lamoth
Valerie Lamour
Ecosse Lamoureux
Francois Lamoureux
Paul Lampe
Kathrin Lampert
Margarita Lampo
Constanze Lamprecht
James Lamsdell

Andre Lamurias
Dominique Lamy
Sébastien Lamy
Chou-Chin Lan
Ethan Lan
Keng-Hsin Lan
Lan Lan
Luhong Lan
Ping Lan
Ruiting Lan
Tsuo-Hung Lan
Weizhong Lan
Wenzhi Lan
Xiangyuan Lan
Xianyong Lan
Yue Lan
Raquel Lana
Lauren Lanahan
Angel Lanas
Claudio Lanata
Graeme Lancaster
Kathryn Lancaster
Marcus Lance
Rachel Lance
Hovirag Lancioni
Rüdiger Land
Premysl Landa
Kristin Landau
Günter Landeck
David Landeira
Gabriel Lander
Natalie Lander
Karin Landerl
Grant Landers
James Landers
Cornelia Landersdorfer
Robert Landewé
Stefan Landgraeber
Dominic Landgraf
Kathrin Landgraf
Alessandro Landi
Francesco Landi
Dawn Landis
Wil J. M. Landman
Nadia Kancheva Landolt
Kristen Landreville
Jean-Francois Landrier
Leslie Landrum
Oriane Landry
Samuel Landry
Shane Landry
Steven Landry
Daniel Landsburg
Andrew Landstrom
Brian Lane
Chad Lane
Darius Lane
Deirdre Lane
Jacqueline Lane
Justin Lane
Nancy Lane
Nicholas Lane
Pierre Lane
Steven Lane
Taylor Lane
Courtney Lane-Donovan
Luisa Lanfranco
Andrew Lang
Catherine Lang
Iain Lang
Ingeborg Lang
James Lang
Martin Lang
Rongling Lang
Steven Lang
Zhihong Lang
Eike Langbehn
Lorrie Langdale
Chris Langdon
Dawn Langdon
Angela Lange
Christian Lange
Claudia Lange
Denise Lange
Tobias Lange
Ana Langer
Harald Langer
Nicolas Langer

Marlyn Langford
Arnulf Langhammer
Simone Langhans
Peter Langhorne
Aaron Langille
Dewi Langlet
Michelle Langley
Ryan Langlois
Timothy Langlois
Soenke Langner
Helio Langoni
L. Langouche
Hilde Langseth
Lisa Langsetmo
David Langsford
Christian Langton
Gabriella Lania
Hayley Lanier
Joanne Lannigan
Donald Lannin
Delphine Lannuzel
Joel Lanoix
Cheryl Lans
Fabio Lansac-Tôha
M. G. Lansberg
Van Lansingh
Alfons Lansink
József Lanszki
Daniele Lantagne
Paul Lantos
Eva Lantsoght
Maria Lantz
Alessandra Lanubile
Árpád Lányi
Gaetano Lanza
Anders Lanzén
Rupert Lanzenberger
Michael Lanzer
Robin Lanzi
Vanessa Soares Lanziotti
Oscar Lao
Wongsa Laohasiriwong
Kamolwish Laoprasopwattana
Yael Laor
Yasmina Laouar
Olav Lapaire
Bruno Lapauw
Pascal Lapébie
Sylvie Lapegue
Olivia Lapenta
Anne Laperche
Syria Laperche
Maryse Lapeyre-Mestre
Suzanne Lapi
Oren Lapid
Moshe Lapidot
Tracey Lapiere
Pascal Lapierre
Katharina Lapin
David Laplante
Andre Laplume
Stephen Lapointe
Robert Laport
Gabriel Laporta
Jimena Laporta
Rosaria Laporta
Sebastien Laporte
Rosamaria Lappano
Joan Lappe
Therese Lapperre
Abbot Laptook
Carlos Lara
Leonel Democrito Lara-Estrada
Matthew Laramie
Liliana Laranjo
Carlos Lara-Romero
Muriel Larauche
Oscar Lara-Velasco
Anis Larbi
Marco Lardies
Beatriz Lardiés-Sánchez
Maximilian Larena
Ismael Lares-Asseff
Jean-Jacques Lareyre
Roddy Large
Stephen Large
Etienne Larger
Raquel Largo

Martina Lari
Bagher Larijani
Sara Lariviere
Sally Lark
Bob Larkin
Celine Larkin
F. Larkin
James Larkin
John Larkin
Michael Larkin
Paul Larkin
Nicolas Larkins
Harry Larman
Jan Larmann
Joseph Larmarange
Maarten Larmuseau
S. Larney
Cécilia Larocca
Francesco Larocca
Kathryn Laroche
Ignacio Larrabide
Aitor Larrañaga
Isabel Larridon
Antoine Larrieu
Bruno Bernard Larrivée
Luis Larrondo
Chris Larsen
David Rolf Larsen
Eva Larsen
Mark Larsen
Martin Larsen
Morten Larsen
Peter Larsen
Ryan Godsk Larsen
A. Noelle Larson
Elizabeth Larson
Elysia Larson
Eric Larson
Helen Larson
Jamie Larson
Julie Larson
Lincoln Larson
Peder Larson
Shawn Larson
Thornton Larson
Kathryn Larson-Johnson
Anders Larsson
Christer Larsson
Jonas Larsson
Lars Larsson
Margareta Larsson
Mattias Larsson
Lillebeth Larun
Tiziana Larussa
Arrate Lasa
Laura Lasagni
Cesar Lascala
Melanie Laschinger
Victor Lasebikan
Amy Lasek
Robert Lash
L. D. Lashev
Kameran Lashkari
Matthias Laska
Tamás Laskay
Judith Lasker
Kristofer Lasko
Paul Lasko
M. Laskowska
Edwin Lasonder
Achim Lass
Laurent Lassabatere
Alvaro Lassaletta
Amandine Lassalle
Florent Lassalle
Cornelia Lass-Flörl
Hans Lassmann
Gorka Lasso
Elena Lasunskaia
Daniele Laszlo
Charu Lata
Maria Ujue Latasa
Emily Latch
Rosa Laterza
M. S. Latha
Kenzie Latham-Mintus
Justin Lathia
Kira Lathrop

Seemab Latif
Gimi Latifi
Andrew Latimer
Kenneth Latimer
Carl Latkin
Donald Latner
Jessica Latorre
Ramon Latorre
Pedro Ángel Latorre Román
Neus Latorre-Margalef
Agnieszka Latosinska
Sylvain Latour
Augustin Latourte
Perle Latré de Laté
Mathieu Latreille
Ardashel Latsuzbaia
Robert Latta
David Lattanzi
Gregory Lattanzi
Loredana Latterini
Benoit Lattuca
Maxime Latulippe
Alexander Latzka
Albert Lau
Billy Lau
Bonnie Lau
Colleen Lau
Erica Lau
Gee Lau
Geoff Lau
John Lau
Ken Lau
Kenneth Lau
Kwok-Fai Lau
Laurie Lau
Phyllis Lau
Stephan Lau
Susanna K. P. Lau
Terrence Chi Kong Lau
Tze Kin Lau
Vi Lau
Wayne Bond Lau
Wei Ling Lau
Yan Kwan Lau
Victor Laubach
Reinhard Laubenbacher
Chris Lauber
Mark Lauchs
Luca Laudani
Augustinus Laude
George Lauder
Tsai-Ling Lauderdale
Vincent Laudet
Alice Laudisio
Dagna Laufer
Kathryn Laughon
Pascal Laugier
Petri Laukka
Elise Launay
Katherine Launer-Felty
Thomas Launey
Sandrine Launois
Ben Laurel
Bishop-Fitzpatrick Lauren
Pintor Lauren
Cato Laurencin
Jean Laurens
Matthew Laurens
Arnaud Laurent
Louise Laurent
Sandra Laurentino
Douglas Laurents
Michael Laurenzano
Matthew Lauretta
Giuseppe Lauria
Claudio Laurido
Henrik Lauridsen
Jørgen Lauridsen
Andrew Laurie
Gordon Laurie
Nikia Laurie
Nicola Laurieri
Arvydas Laurinavicius
Kenneth Laurita
Chiara Lauritano
Lotte Lauritzen
Bjarne Laursen
Richard A. Laursen

Volker Lauschke
Ludwig Lausser
Christoffer Laustsena
Tim Lautenschlaeger
Sylvie Lautru
Lena Lautscham
Dale Lauver
Gunter Laux
Benjamin Lauzier
Anne-Marie Lauzon
Sebastiano Lava
Luca Lavagnino
Sergio Lavandero
Patricia Lavand'homme
Afsaneh Lavasanifar
Gary Laver
Kate Laver
Michael Laver
Bonnie Laverock
Jean-Noel Laverriere
Gareth Lavery
Enrico Lavezzo
Hugo Laviada
Alessandro Laviano
Michal Lavidor
Carl Lavie
Eric Lavigne
Pierre Lavigne
Gaute Lavik
Maurice Laville
Dimitri Lavillette
Joel Lavine
Cristina Lavini
Victor Lavis
Sofia Lavista-Llanos
Robert Lavker
Les Lavkulich
Justin A. Lavner
Claude Lavoie
Jean-Pierre Lavoie
Kim Lavoie
Patrick Lavoie
Suzie Lavoie
Tera Lavoie
Anne-Violette Lavoir
Iris Lavon
Élise Lavoué
Harish Lavu
Amanda Law
Benedict Law
Brian Law
Catherine Law
Cliff Law
Lawrence Law
Mansun Law
Matt Law
Yuen Kwan Law
Oladipupo Lawal
Alfons Lawen
Timothy Lawes
Katherine Lawler
Elizabeth Lawlor
Agnieszka Lawniczak
Alistair Lawrence
B. Paige Lawrence
Dana J. Lawrence
David Lawrence
Elizabeth Lawrence
John Lawrence
Michael Lawrence
Kate Lawrenson
Nathan Lawrentschuk
Barbara Lawrenz
Stephen Lawrie
Daniel Lawson
David Lawson
James Lawson
Julia Lawson
Lucinda Lawson
Michelle Lawson
Victoria Lawson
Sunil Laxman
Michael Laxy
Brendan Lay
Chih-Ying Lay
Michael Layden
Mehdi Layeghifard
Charles Layne

J. Bradley Layton
Kara Layton
David Layzell
Alina Lazar
Veronica Lazăr
Allison Lazard
Iosif Lazaridis
Maria Lazarina
Stergios Lazarinis
Amparo Lazaro
Ester Lázaro
Orly Lazarov
J. Lazarus
Cristina Lazcano
Helen Lazear
Gweneth Lazenby
Deann Lazovich
Ahmed Lazrak
Lambros Lazuras
Giulia Lazzarini
Douglas Lazzaro
Marzia Lazzerini
Anh Le
Hanna Le
Hoa Le
Lu Le
Qihua Le
Quan Le
Quang Bao Le
Thuy Le
Trung Le
Yun Le
Rozenn Le Berre
Yann-Ael Le Borgne
Raphael Le Bouc
Ronan Le Bouffant
Eric Le Bourg
Yoann Le Breton
Edward Le Brun
Yann Le Cunff
Christophe Le Dantec
Kirsty Le Doare
Joost le Feber
Gaetan Le Floch
Christelle Le Foll
Sylvain Le Gall
Gaelle Le Goff
Ronan Le Goffic
Renaud Le Goix
Vadim Le Joncour
Alix Le Marois
Cédric Le May
Vincent Le Moing
Gilles Le Moullac
Fabienne Le Provost
Jean Yves Le Reste
Virginie Le Rolle
Stanzi Le Roux
O. Le Rouzic
Olivier Le Saux
Marion Le Texier
C. Suzanne Lea
David Lea
Amanda Leach
Avi Leader
Erin Leahey
Patricia Leahy-Warren
A. Leal
Ermelindo Leal
Fabio Leal
Manuel Leal
Francisco Leal-Soto
Michael Lean
Christopher Lear
Alberto Leardini
Vijittra Leardkamolkarn
Alexandra Leary
Christopher Leary
Eric Leas
Andrew Leask
Richard Leask
Simon Leather
David Leavesley
Silas Leavesley
Breiffni Leavy
Simon Lebaron
Luc Lebeau
David Lebeaux

J Leblanc
Judith Leblanc
Hakan Leblebicioglu
Arthur Leblois
Florent Lebon
Christophe Leboulanger
Stan Lebow
Benedicte Lebret
Carlito Lebrilla
Ivo Lebrun
Jean-Baptiste Leca
Michel Lecendreux
Margaret Lech
André Lechel
Monique Léchenne
Walter Lechner
Jeannette Lechner-Scott
Michael Lecholop
Aron Lechtig
Beata Lecka-Czernik
Brian Leckie
Estelle Leclerc
Martin Leclerc
Melen Leclerc
Nicole Leclerc
Suzanne Leclerc-Madlala
Alexandre Leclercq
Sophie Leclercq
Julie Lecomte
Magalie Lecourtois
Timothy Lecras
Valérie Lecureur
Helmut Leder
Karin Leder
Eleanor Lederer
Debora Ledergerber
Frank Lederle
José Ledesma
Lola Ledesma
Rodrigo Ledesma Amaro
Timothy Ledgeway
John Lednicky
Alicia Ledo
Justin Ledogar
Aaron Lee
Alan Lee
Alfred Lee
Allison Lee
Amy Lee
An-Sheng Lee
Arier Chi Lun Lee
Bang Yeon Lee
Barry Lee
Benhur Lee
Benjamin Lee
Beth Lee
Boreom Lee
Brian Lee
Bryan Lee
Byung Il Lee
Byung Ro Lee
C. K. Lee
Cecilia Lee
Ch Lee
Chan Joo Lee
Chang Hyun Lee
Chang Lee
Changhan Lee
Changjoon Justin Lee
Chang-Soo Lee
Chanhui Lee
Charles Tzu-Chi Lee
Cheng-Chi Lee
Cheng-Chung Lee
Cheng-Kang Lee
Cheng-Shiuan Lee
Cheuk Kwong Lee
Chia Lee
Chia-Hwa Lee
Chii-Ming Lee
Choong-Eun Lee
Chul Hee Lee
Chun Yi Lee
Chung-Lin Lee
Chung-Ta Lee
Chun-Woo Lee
Chu-Yu Lee
Clara Lee

D. Lee
Dae-Hee Lee
Dakeun Lee
Darren Lee
David Lee
Dawn Lee
De-Hyung Lee
Derek Lee
Do Yup Lee
Dong Gun Lee
Dong Hoon Lee
Dong Hun Lee
Dong Wan Lee
Dong-Gun Lee
Dong-Yup Lee
Douglas Lee
Eun Ji Lee
Eun Jin Lee
Eun Kyung Lee
Eun Young Lee
Eunsong Lee
Eun-Young Lee
Gabsang Lee
Gap Ryol Lee
Geraldine Lee
Hane Lee
Hee Lee
Hee Young Lee
Herbert Lee
Ho Joung Lee
Hookeun Lee
How-Jing Lee
Hsiao-Hui Lee
Hsien-Ming Lee
Hsin-Chen Lee
Hsin-Chien Lee
Hui Lee
Hye Seung Lee
Hye Won Lee
Hye Yeon Lee
Hye-Ja Lee
Hyojung Lee
Hyongki Lee
Hyun Soo Lee
Hyun Woong Lee
Hyunglae Lee
Hyunju Lee
I-Cheng Lee
Ik Jae Lee
In-Kyu Lee
Iris Lee
I-Te Lee
J. S. Lee
Jae Eun Lee
Jae Kwan Lee
Jae Lee
Jae-Seong Lee
Jaewon Lee
Janet Lee
Je Chul Lee
Jeffrey Lee
Jen-Ai Lee
Jeongae Lee
Jeong-Chae Lee
Jeonghwan Lee
Jia-Jung Lee
Jih-Hsiang Lee
Jihye Lee
Jin Lee
Jin-A Lee
Jinhee Lee
Ji-Woong Lee
Ji-Young Lee
Ji-Yun Lee
John Lee
Jonathan Lee
Jong-Soo Lee
Joo Hyoung Lee
Joon Lee
Joon-Hee Lee Lee
Joo-Yong Lee
Joung-Hun Lee
Joy Lee
Juhyun Lee
Julian Lee
Jun Ho Lee
Jundae Lee
Jungchul Lee

Jung-Jae Lee
Jungkwan Lee
Jung-Seok Lee
Junsoo Lee
Juwon Lee
Kangjae Lee
Kangjoo Lee
Kangwon Lee
Kang-Yun Lee
Kathryn Lee
Kelly Lee
Kern Lee
Kerry Lee
Keun Hwa Lee Lee
Kichun Lee
Kuei-Chuan Lee
Kun Chang Lee
Kuo-Jung Lee
Kwang Pum Lee
Kyu Eun Lee
Kyu-Ho Lee
Kyung-A Lee
Kyungwon Lee
Le Ye Lee
Lewina Lee
Li-Ang Lee
Lois K. Lee
M. Catherine Lee
Matthew. Lee
Mei-Hsuan Lee
Mei-Yueh Lee
Mi Ji Lee
Mi Suk Lee
Michelle Lee
Ming-An Lee
Ming-Been Lee
Ming-Ching Lee
Ming-Shinn Lee
Ming-Shyue Lee
Minsun Lee
Myeongwoo Lee
Namki Lee
Natuschka Lee
Ni-Chung Lee
Nikki Lee
Nora Lee
P. Lee
Paul Lee
Pei-Chang Lee
Phyllis Lee
Po-Huang Lee
R. K. Lee
R. Lee
Robert Lee
Robin Lee
Royce Lee
Samuel Lee
Sang Haak Lee
Sang Joon Lee
Sang Lee
Sang Wook Lee
Sang-Hie Lee
Sang-Kyu Lee
Sangmi Lee
Sang-Myeong Lee
Sangun Lee
Seung-Goo Lee
Seung-Jae Lee
Shyh-Jye Lee
So Hee Lee
Song Lee
Soo Chan Lee
Soo-Chin Lee
Soonam Lee
Suk Keun Lee
Sun Eun Lee
Sun Hee Lee
Sun Lee
Sung Chul Lee
Sung Lee
Sung-Bau Lee
Sun-Ho Lee
Sunkyung Lee
Suzanne Lee
T. T. Lee
Tae-Jin Lee
Teng-Yu Lee
Tien-Wen Lee

Todd Lee
Tsong-Hai Lee
Tsung-Han Lee
Tzong-Yi Lee
Victor Ho-Fun Lee
Vivian Lee
W. Lee
W. M. Steve Lee
Walter Lee
Wei-Chen Lee
Wei-Chieh Lee
Wei-Ju Lee
Wei-Ning Lee
Wen-Chung Lee
Wk Lee
Won Ki Lee
Won Woo Lee
Won-Joon Lee
Wooin Lee
Yeon-Ah Lee
Yeon-Hee Lee
Yi-Chia Lee
Yi-Chung Lee
Yie Hou Lee
Yi-Jang Lee
Ying-Chou Lee
Yong Lee
Yong-Ho Lee
Young Hen Lee
Young-Ae Lee
Young-Jae Lee
Young-Sam Lee
Youngseok Lee
Yueh Lee
Edwin Leeansyah
Martin Leeb
Tosso Leeb
Mariska Leeflang
Peter Leegwater
Surbhi Leekha
Pimlapas Leekitcharoenphon
Kristen Leeman
Luke Leenen
Aviva Lee-Parritz
Jessica Leers
Michael Lees
Paul Leeson
W. Matthew Leevy
Chen Lee-Wei
Louis Lefaucheur
Carmen Lefevre
Christopher Lefevre
Sarah Lefevre
Sebastien Lefevre
Sjannie Lefevre
Daniel Leff
Todd Leff
Lisa Leffert
Jonatan Leffler
Elliot Lefkowitz
Thomas Lefort
Anne-Marie Lefrançois-Martinez
Cecile Legallais
Marc-André Legault
Nicolas Legay
Frédéric Legendre
Lucas Legendre
Leecole Legette
Joscha Legewie
David Legge
Kevin Legge
Kristina Legget
Leroy Leggitt
Daniel Legler
Benoit Legoff
Jacopo Legramante
Jean-Luc Legras
B. Legros
Anne-Sophie Leguern
V'yacheslav Lehen'kyi
Aleksi Lehikoinen
Tanya Lehky
David Lehman
Michael Lehman
R. Lehman
Arne Lehmann
Helmar C. Lehmann
Hugo Lehmann

Jörg Lehmann
Laurent Lehmann
Mick Lehmann
Philipp Lehmann
Rainer Lehmann
Tomasz Lehmann
Klaus Lehmann-Horn
Ricarda Lehmitz
Markus Lehmkuhl
Lukas Lehner
Sigrid Lehnert
Steven Lehotay
Stephanie Lehoux
Edgar Lehr
Jonathan Lehrfeld
Johann Lehrner
Esko Lehtonen
Sanna Lehtonen
Mikko Lehtovirta
Agnes Lehuen
Chao-Liang Lei
Chengwei Lei
Elissa Lei
Hansheng Lei
Hao-Jan Lei
Hong Lei
Hongxing Lei
Jikai Lei
Mingxing Lei
Peng Lei
Qinghua Lei
Tie-Chi Lei
Xia Lei
Xiaoying Lei
Xin Gen Lei
Xin Lei
Xinglin Lei
Xiujuan Lei
Yaguo Lei
Yun Lei
Zhenmin Lei
Margarita Leib
Raz Leib
Rudolph Leibel
Jessica Leibler
Elizabeth Leibold
Didier G. Leibovici
Emily Leibovitch
Arleen Leibowitz
Avshalom Leibowitz
Rachel Leibu
David Leiby
Jami Leichliter
Christof Leicht
Alexander Leichtle
Sebastian Leidel
Jennifer Leiding
Samantha Leigh
P. Leighton
Julie Leignadier
Jolien Leijenaar
Arne Leijon
Emmerik Leijten
George Leikauf
Pamela Lein
Mallorie Leinenger
Olof Leinhard
Gina Leinninger
Michael Leipold
Enrique Leira
Tanya Leise
Daniel Leisman
Christoph Leisser
Jorge Leitão
Carlinda Leite
Gabriela Leite
Hercules Leite
Marcio Leite
Nuno Leite
Gerd Leitinger
Juan Leiva
Laura Leiva
Mārcis Leja
Tomás Lejarraga
Fabrice Lejeune
Charlotte Lekieffre
Marilena Lekka
Jesse Leland

Pushkar Lele
Margareta Lelea
Neal Leleiko
Bruno Leles
Frederik Leliaert
Vincent Lelievre
Lisette Leliveld
Michael Lell
Patrick Lelliott
Cécile Lelong
Christophe Lelong
Pavlos Lelovas
A. Titia Lely
Isabel Lema
Edward D. Lemaire
Patrick Lemaire
Claire Lemaitre
Jean-François Lemaitre
Rafael Lemaitre
Edward Lemay
Guy Lemay
Martin Lemay
Valerie Lemay
Giuseppe Lembo
Rosalba Lembo
Erwin Lemche
Frances Lemckert
Cynthia Lemere
Raphael Lemes Hamawaki
Gilles Lemesle
Srdjan Lemez
Isabelle Lemieux
Michael Lemke
Justin Lemkul
Kevin Lemley
Eshetu Lemma
Robin Lemmens
Niels Lemmermann
Marike Lemmers
Helge Lemmetyinen
Frederic Lemoine
Sandrine Lemoine
Sakari Lemola
Christian Lemon
Francois Lemonnier
Artur Lemonte
Elkin Lemos
Francine Lemos
Marcos Lemos
Nucelio Lemos
Pedro Lemos
Mathieu Lempereur
Willem Lems
Luis Lemus
Brigitte Lemyre
Agatha Lenartowicz
Heather Lench
Pierre-Pascal Lenck-Santini
Lonneke Lenferink
Gareth Leng
Ping Leng
Roger Leng
Theodore Leng
Csaba Lengyel
Imre Lengyel
Michael Lenhard
Colin Lenihan
Gustavo Lenis
Guy Lenk
Narendra Lenka
Balint Lenkei
Frances Lennon
Rachel Lennon
Maxime Lenormand
Eelco Lens
Eef Lentjes
Nathan Lents
Tomas Lentz
Frank Lenze
Marco Leo
Ming Di Leom
Javier Leon
Lisa Leon
Lorenzo Leon
Lydia Leon
Anne Leonard
Carly Leonard
Christopher Leonard

Hayley Leonard
Jack Leonard
Timothy Leonard
Guillaume Léonard
Antonio Leonardi
Matilde Leonardi
Lea Leonardis
Roberto Leonarduzzi
Lorenzo Leoncini
Alessandro Leone
Aurelio Leone
Marc Leone
Sebastiano Leone
Darryl Leong
Fong Yew Leong
Kelvin Leong
Howard Leong-Poi
James M. Leonhardt
Giovanna Leoni
Andreas Leonidou
Isabel Leonor
Antonio Leon-Reyes
Vladimir Leon-Salazar
Fidias E. Leon-Sarmiento
Marco Leonti
Christine Leopold
Jane Leopold
Jeannie-Marie Leoutsakos
Li-Ann Leow
Martin Lepage
Nathalie Lepage
Jesse Lepak
Alain Lepape
Malgorzata Lepczynska
Antonio Lepedda
Snjezana Lepej
Didier Lepelletier
Romuald Lepers
Edwin D. Lephart
Fabio Lepori
Christian Leporini
Andrew Lepp
Jaana Leppalammi-Kujansuu
Jenni Leppanen
Jimmie Leppink
Bruno Lepri
Jerome Leprince
Sebastian Lequime
Isaac Lera
Emmanuelle Leray
Alexander Lerchl
Maria Lerm
Kristina Lerman
Lilach Lerman
Aaron Lerner
Dan Lerner
Ethan Lerner
Nancy Lerner
Michel Leroux
Shawn Leroux
Boris Leroy
Celine Leroy
Gregoire Leroy
Jef Leroy
Karelle Leroy
O. Leroy
Sandrine Leroy
Valeriane Leroy
Leticia Lery
Elise Lesage
Florian Lesage
Suzanne Lesage
Sylvie Lesage
Claude Lesaux
David Lesbarrères
Roxana Lescano
Félice Lê-Scherban
Sandrine Lesellier
Micah Leshem
Clémence Lesimple
Emily Leskinen
Katarzyna Leskinen
Andrew Leslie
Elizabeth Leslie
Frances Leslie
Hannah Leslie
John Leslie
Monica Leslie

William Leslie
Elena Lesma
Sylvain Lesne
Edward J. Lesnefsky
Philippe Lesnik
Saskia Lesnik Oberstein
Carolyn Lesorogol
E. Lesovaya
Leonardo Lessa
Heidi M. B. Lesscher
Richard Lessells
Iris Lesser
Mary Lesser
Harilaos Lessios
Susan Lessner
Robert Lester
Sarah Lester
Lionel Leston
Olivier Lesur
Jurate Lesutiene
Roman Lesyk
Scott Letendre
Sébastien Leteneur
Sophie Leterme
Jean-Jacques Letesson
Craig Leth-Steensen
Nicole L. Letourneau
Ariadne Letra
Christophe Lett
Jun-Yi Leu
Tzeng-Horng Leu
Eleonora Leucci
Philipp Leucht
S. Leuci
Stefan Leuko
Nicolas Leulliot
Ada W. S. Leung
Ann Leung
Doris Leung
Hon-Chiu Leung
Janni Leung
June Leung
Justin Leung
Kenneth Leung
Kwok Leung
Maggi Leung
Nicki Leung
Patrick Leung
Richard Leung
Sharon Leung
Susan Leung
Ting Fan Leung
W. K. Leung
William Leung
Ying Ying Leung
Yuet-Kin Leung
Florian Leuschner
Hartmut Leuthold
Henri Leuvenink
Christoph Leuze
Danielle Levac
Patrick Levallois
Pierre LeVan
Beth Levant
Stephen Levas
Romain Levayer
Thierry Léveillard
Gabriel Leventhal
Michael Leveridge
Jean Pierre Levesque
Maxime Levesque
Mitch Levesque
Simon Lévesque
Florian Levet
Kate Levett
Nicolas Leveziel
Amnon Levi
Edi Levi
Mark S. Levi
Moshe Levi
Sonia Levi
Taal Levi
Scott Levick
Shaun Levick
Anna Levin
Carol Levin
L. Scott Levin
Michael Levin

Mindy Levin
Oron Levin
Roy Levin
Anna Levina
Vera Levina
Aaron Levine
Adam Seth Levine
Edward Levine
James Levine
Jon Levine
Jonathan Levine
Mark Levine
Martin Levine
Min Levine
Steven Levine
Todd Levine
Zachary Levine
Marika Leving
Louis Levinger
David Levinson
Francesca Levi-Schaffer
Irena Levitan
Jonathan Levitt
Michael Levitt
Alexander Levitzki
Liat Levontin
Jacques Levraut
Elena Levtchenko
Hadar Lev-Tov
Amit Levy
Ariel Levy
Becca Levy
Cynthia Levy
Dalit Levy
David Levy
Jerrold Levy
Jonathan Levy
Karen Levy
Matthew Levy
Michael Levy
Richard Levy
S. Levy
Daniel Levy-Bercowski
Jason Kar Sheng Lew
Jie-Bin Lew
Jörg Lewald
Beata Lewandowska
Eva Lewandowski
Piotr Lewczuk
Jan Lewerenz
Astrid Lewin
Amanda Lewis
Andrew Lewis
Cara Lewis
Cathryn Lewis
Dale Lewis
Daniel Lewis
David Lewis
Evan Lewis
Felicia Lewis
Gary Lewis
Jani Lewis
Jennifer Lewis
Jerome Lewis
Jesse Lewis
Jill Lewis
Jonathan Lewis
Kemper Lewis
Kristy Lewis
L. Kevin Lewis
Lawrence Lewis
Levi Lewis
Lucy Lewis
Margaret Lewis
Matt Lewis
Michael Lewis
Nathaniel Lewis
Peter Lewis
Richard Lewis
Sara Lewis
Simon Lewis
Vernard Lewis
Zachary Lewis
Zenobia Lewis
Grant Lewison
Jarrod Lewis-Peacock
Joan Lewis-Wambi
Alan Lewitus

Ala Lew-Tabor
Sonia Lewycka
Alexandra Ley
Benedikt Ley
Eric Ley
Jakob Ley
Loet Leydesdorff
Luc Leyns
Misti Leyva
Victor Leyva-Grado
Timothy Lezon
Frederic Lezot
Marco Lezzerini
Samden Lhatoo
Erwan Lher
Ludovic Lhermitte
Thibault Lhermusier
Yvan L'Homme
Lenka Lhotska
Karl Lhotta
An Li
Anthony Li
An-Wei Li
Baikun Li
Baolan Li
Baoxin Li
Benyi Li
Bibo Li
Bin Li
Bingshan Li
Bing-Tao Li
Bingyun Li
Bo Li
Bofeng Li
Boqiang Li
Changgui Li
Changwei Li
Changzhi Li
Chenghe Li
Chenghua Li
Chengtao Li
Chengwei Li
Chenzhao Li
Chia-Cheng Li
Chia-Lin Li
Chong Li
Chuandong Li
Chun Wei Li
Chunbo Li
Chung-Hsing Li
Chung-Yi Li
Chunhao Li
Chunhui Li
Chunjie Li
Chunlian Li
Chunliang Li
Chunming Li
Chunxiao Li
Chunyan Li
Conggang Li
Da Li
Dan Li
Danielle Li
Dawei Li
Dayong Li
Dechang Li
Defang Li
Deguang Li
Dejun Li
Der-Chiang Li
Dongye Li
Duo Li
Erchao Li
Fagen Li
Fa-Jun Li
Fali Li
Fan Li
Fanbiao Li
Faqiang Li
Fei Li
Feng Li
Fengna Li
Fuguang Li
Fuhong Li
Fuhua Li
Fuzhen Li
G. Li
Gang Li

Ganghua Li
Ge Li
Gen Li
Genghui Li
Guang Li
Guanglin Li
Guangqi Li
Guangye Li
Guibing Li
Gui-Hua Li
Guiying Li
Guoan Li
Guojun Li
Guoping Li
Guoqi Li
Guoshi Li
Haihong Li
Haiqi Li
Haiyun Li
Hanzeng Li
Hao Li
He Li
Henghong Li
Hengzhe Li
Hong Li
Hongbo Li
Hongjie Li
Hong-Li Li
Hongmin Li
Hongwei Li
Hongyang Li
Hu Li
Hua Li
Huabin Li
Huaixing Li
Huang Li
Huating Li
Hui Li
Huiguang Li
Huihui Li
Huijia Li
Hui-Quan Li
Huiying Li
Hung-Yuan Li
James J. Li
Ji Li
Jia Li
Jiale Li
Jiali Li
Jian Li
Jiang-Tao Li
Jianhong Li
Jiani Li
Jian-Jun Li
Jianping Li
Jianquan Li
Jianwei Li
Jianwu Li
Jianxiong Li
Jianxun Li
Jiaqi Li
Jie Li
Jieliang Li
Jiguo Li
Jin Li
Jinchen Li
Jing Li
Jingyuan Li
Jinnian Li
Ji-Su Li
John Li
Joule Li
Juan Li
Jun Li
Kaiming Li
Ke Li
Kexue Li
Kui Li
Kun Li
L. Li
Laigeng Li
Laixing Li
Lan Li
Lang Li
Le Li
Leida Li
Lennon Li
Leping Li

Le-Qun Li
Li Li
Lian Li
Liang Li
Liangcheng Li
Liangzhu Li
Lianhui Li
Lihua Li
Li-Hua Li
Lihui Li
Li-Ming Li
Lin Li
Lin-Feng Li
Ling Li
Ling-Hui Li
Linna Li
Liping Li
Lixiang Li
Longchuan Li
Longfeng Li
Longhui Li
Lu Li
Lu-Lin Li
Luowei Li
Luping Li
Lying Ryan Li
Mai Suan Li
Man-Wah Li
Mao Li
Maoteng Li
Meili Li
Mei-Ling Li
Meng-Hua Li
Miao-Xin Li
Min Li
Ming Li
Mingcheng Li
Ming-He Li
Mingjun Li
Ming-Tao Li
Ming-Xia Li
Mingyu Li
Mingzhou Li
Minqiang Li
Mo Li
Mu Li
Munan Li
Muwang Li
Na Li
Nan Li
Ningdong Li
Pan Li
Peipei Li
Peiyang Li
Peng Li
Ping Li
Qi Li
Qian Li
Qiao Li
Qiaoli Li
Qifeng Li
Qinfen Li
Qing Li
Qingguo Li
Qinglei Li
Qingsheng Li
Qingyang Li
Qiu Li
Qiutang Li
Qiwei Li
Qun Li
Ran Li
Renfeng Li
Renhao Li
Renhui Li
Robert Li
Rong Li
Rongjian Li
Rui Li
Sai-Ping Li
Sam Li
Shan Li
Shanshan Li
Shao Li
Sheng Li
Shenghui Li
Shengyu Li
Sheyu Li

Shihua Li
Shijia Li
Shi-Ming Li
Shiri Li
Shi-Weng Li
Shiying Li
Shou Li
Shouli Li
Shuang Li
Shundai Li
Shunping Li
Shunzhen Li
Shuping Li
Shyh-Dar Li
Song Li
Spencer Li
Su Li
Sung-Chou Li
Tao Li
Tiangang Li
Tianhong Li
Tianliang Li
Tianlu Li
Tingqiang Li
Tingting Li
W. P. Li
Wan-Feng Li
Wei Bo Li
Wei Li
Weidang Li
Weigang Li
Weikai Li
Wei-Wei Li
Weixi Li
Wen Li
Wenchen Li
Wen-Cheng Li
Wenhui Li
Wenjing Li
Wenqing Li
Wentian Li
Wen-Xue Li
Wenyuan Li
Wu Li
Xi Li
Xia Li
Xiande Li
Xiang Li
Xiangcheng Li
Xianghong Li
Xiangkai Li
Xiangmin Li
XiangRui Li
Xianhong Li
Xianyue Li
Xiaobai Li
Xiaobing Li
Xiaobo Li
Xiaohong Li
Xiaohua Li
Xiaojiang Li
Xiaojuan Li
Xiaojun Li
Xiaokun Li
Xiaoli Li
Xiaoling Li
Xiaomin Li
Xiaoming Li
Xiaoqing Li
Xiaoshu Li
Xin Li
Xingfeng Li
Xing-Hui Li
Xingpu Li
Xinhu Li
Xinli Li
Xinwu Li
Xinyuan Li
Xiong Li
Xiuqing Li
Xiuzhen Li
Xu Li
Xuan Li
Xue Li
Xuechao Li
Xuejie Li
Xuewei Li
Xue-Yan Li

Xueyong Li
Xun Li
Xuri Li
Xuyan Li
Yan Li
Yang Li
Yanhong Li
Yanming Li
Yanping Li
Yanqing Li
Yan-Ruide Li
Yansong Li
Yao Li
Ye Li
Yi Li
Yihong Li
Yimin Li
Yin Li
Yinchuan Li
Ying Li
Ying Zhen Li
Yiwen Li
Yong Li
Yongfang Li
Yonghe Li
Yonghua Li
Yonghui Li
Yongjun Li
Yongqing Li
Yongxin Li
You Li
You-Ming Li
Youyong Li
Yu Li
Yuan Li
Yuan-Yuan Li
Yuchao Li
Yue Li
Yuezhou Li
Yuhuan Li
Yuju Li
Yuling Li
Yumeng Li
Yuncong Li
Yung-Hui Li
Yunhe Li
Yunmei Li
Yunqi Li
Yunzi Li
Yupeng Li
Yuqing Li
Yuzhan Li
Zaiyun Li
Zechao Li
Zenglu Li
Zengning Li
Zengyong Li
Zhan Li
Zhaozhu Li
Zhe Li
Zhen Li
Zhenlin Li
Zhenqing Li
Zhi Li
Zhibin Li
Zhigang Li
Zhiming Li
Zhiqiang Li
Zhiru Li
Zhi-Yong Li
Zhong-Guang Li
Zhonghui Li
Zhongjun Li
Zhongming Li
Zhongwei Li
Zhongwu Li
Zhuoyu Li
Zhuyun Li
Zibo Li
Zichong Li
Zihai Li
Zixuan Li
Zongyun Li
李妙龄 Li
Bertrand Liagre
Vassilios Liakopoulos
Ie-Bin Lian

Jane Lian
Qizhou Lian
Zhanghua Lian
Lei Liancheng
Bin Liang
Carol Liang
Chih-Chia Liang
Feng Liang
Guodong Liang
Guohua Liang
Hao Liang
Hejun Liang
Hualou Liang
Ja-Der Liang
Leng Liang
Li Liang
Lingyi Liang
Mike Liang
Mingxiang Liang
Nu-Chu Liang
Po-Huang Liang
Qiming Liang
Qiu-Xia Liang
Sai Liang
Sharon Liang
Wanjie Liang
Xi Liang
Xiaoming Liang
Xin Liang
Yan Liang
Ying Liang
Yuanxin Liang
Yue Liang
Zhifeng Liang
Georgios Lianos
Alexandra Lianou
Chen Liao
Fuyuan Liao
Ge Liao
Jan-Ray Liao
Jiahn-Haur Liao
Jun Min Liao
Junming Liao
Jyh-Fei Liao
Kuan-Fu Liao
Li Liao
Lili Liao
Limin Liao
Lin Rong Liao
Lingjie Liao
Ping Liao
Q. Vera Liao
Shang-Chih Liao
Sumei Liao
Vivian Liao
Wei Liao
Wei-Chih Liao
Wenbo Liao
Xin Liao
Xing Liao
Xuejun Liao
Yaping Joyce Liao
Ying-Yi Liao
Yong-Hong Liao
You-Di Liao
Zhihua Liao
Zhuan Liao
Hannan Liaqat
Ayalon Liat
Stavros Liatis
Shwu-Jen Liaw
Cleiton Libardi
Ben Libberton
Stephen Libby
Geneviève Libeau
Karsten Liber
Alessio Liberati
M. Charles Liberman
Mark Liberman
Zoe Liberman
Sergiy Libert
Micaela Liberti
Michelle Liberton
Josie Libertucci
Alexander Liberzon
Israel Liberzon
Zamir Libohova
Alexandre Liborio

Massimo Libra
Daniel Libraty
Lísa Libungan
Anna Licata
Frederick Licciardi
Paul Licciardi
Rodríguez Licel de los Angeles
Alexander Lichius
Christoph Licht
Michael Lichtenauer
Kenneth Lichtenstein
Glen Lichtwark
Robin Lickley
Valerio Licursi
Belinda Liddell
Shane Liddelow
Alexander Liddle
Sarah Lidstone
Charles Lidz
John Lie
Roselind Lieb
Stefan Liebau
Harris Lieberman
Jay Lieberman
David Liebeskind
Jürgen Liebig
Andrea Liebl
Susan Liebman
Thoralf Liebs
George Liechti
Edward Liechty
Hiltrud Liedgens
Wolfgang Liedtke
Justin Liefer
Franziskus Liem
Judit Lienert
Christian Lienhardt
Sascha Liepelt
John Lieske
Daniel Lietha
Woei Chang Liew
Pernilla Lif Holgerson
Jan Lifjeld
Alan Lifson
Alessadro Ligas
Douglas Light
Jessica Light
Johnson Lightfoote
Nichole Lighthall
Joseph Lightner
Owen Lightsey
Giovanni Ligresti
Wilco Ligterink
Claudio Liguori
Raphael Lihana
Jaan Liira
Diego Lijavetzky
Willem Lijfering
H. Roger Lijnen
Dario Lijtmaer
Walter Lilenbaum
Ann Liljas
Jerker Liljestrand
Sarah Lillie
Cathrine Lillo
John Lillvis
Brenda Lilly
Albert Lim
Bora Lim
Burton Lim
Changwon Lim
Ching Jou Lim
Chunghun Lim
Daniel Lim
David Lim
Faye Lim
Heon Lim
Ilhan Lim
James Lim
Jung Yul Lim
Juyun Lim
Kean Lim
Lena Lim
Lina Lim
Megan Lim
Mi Young Lim
Renly Lim
Robert Lim

Sang Min Lim
Seng Gee Lim
Shen-Yang Lim
Sian Yik Lim
Sin How Lim
Su-Ho Lim
Sungwoo Lim
Un Taek Lim
Unhee Lim
Yoong Ping Lim
Young Woon Lim
Young-Suk Lim
Yun Ping Lim
Yun-An Lim
Alexandre Lima
Carla Lima
Danielly Lima
José Bento Lima
Kledoaldo Lima
Luis Mauricio Lima
Marina Lima
Walter de Paula Lima
Josué Lima Junior
Jan Liman
N. Liman
Adriana Limansky
Matheus Lima-Ribeiro
Adriano Lima-Silva
Svetlana Limborska
David Limbrick Jr.
Karin Limburg
Kirsten Limesand
Thawornchai Limjindaporn
Direk Limmathurotsakul
Charles Limoli
Patricia Limousin
Thanawut Limpanitgul
Teresa Limpo
Athena Lin
Bai-Quan Lin
Been-Ren Lin
Brenda Lin
Brian Lin
Chang-Shen Lin
Chen Lin
Chen-Chun Lin
Cheng-Li Lin
Cheng-Wen Lin
Chen-Yong Lin
Chi Lin
Chia-Ho Lin
Chia-Hua Lin
Chi-Chien Lin
Chieh-Hsin Lin
Chien-Chi Lin
Chien-Chung Lin
Chien-Liang Lin
Chih-Che Lin
Chih-Ching Lin
Chih-Peng Lin
Chi-Hung Lin
Chih-Wen Lin
Ching-Yeh Lin
Chin-Hsien Lin
Chuan Lin
Chun Chieh Lin
Chung-Jung Lin
Chung-Yen Lin
Chun-Liang Lin
Chun-Mao Lin
Chun-Wang Lin
Chun-Yen Lin
Chun-Yu Lin
Daniel Lin
Dingbo Lin
Dong-Qiang Lin
Dung-Ying Lin
Fang-Tsyr Lin
Feng Lin
Feng-Chang Lin
Gen-Min Lin
Guiting Lin
Hai Lin
Haijiang Lin
Han-You Lin
Hao Lin
Haotian Lin
Ho-Hsiung Lin

Hsiang Yu Lin
Hsi-Hsun Lin
Hsing-Juh Lin
Hsueh-Kung Lin
Hualiang Lin
Hugo Lin
Hui-Long Lin
Hung-Du Lin
Hung-Yun Lin
Jen-Der Lin
Jennifer Lin
Jiaping Lin
Jiaxing Lin
Jie Lin
Jin Lin
Jin-Ching Lin
Jing Lin
Jiu-Jenq Lin
Jules Lin
Jun Lin
Jung-Chun Lin
Jung-Hsin Lin
Keh-Chung Lin
Kun-Der Lin
Kun-Ju Lin
Kwang-Huei Lin
Lei Lin
Li Lin
Liang-Ching Lin
Li-Chan Lin
Lifeng Lin
Li-Mei Lin
Lin Lin
Liqiang Lin
Longhui Lin
Mao Lin
Meifang Lin
Michael Lin
Ming-Hwai Lin
Ming-Tai Lin
Ming-Tsan Lin
Ming-Tsung Lin
Ming-Tzer Lin
Ming-Yen Lin
Nan Lin
Ning Lin
Pan Lin
Ping-I Lin
Rongcheng Lin
Ruo-Kai Lin
Shan Lin
Shankung Lin
Sheng Lin
Sheng-Hsuan Lin
Shih-Hsien Lin
Shih-Hua Lin
Shin Lin
Si-Min Lin
Suewei Lin
Susan Lin
Sze-Kwan Lin
T. Y. Lin
Tao Lin
Tingting Lin
Tzu-Hsin Lin
Tzung-Jin Lin
Wei Lin
Weichun Lin
Wei-Ning Lin
Wenwei Lin
Wenxiong Lin
Wenyi Lin
Win-Li Lin
Xiaochen Lin
Xiaopei Lin
Xiaoxi Lin
Xin Lin
Xinfan Lin
Xiuyun Lin
Y. C. Lin
Yan-Ren Lin
Yansong Lin
Yanwei Lin
Yen-Chung Lin
Yenn-Jiang Lin
Yen-Sheng Lin
Yi Lin
Yi-Ching Lin

Yi-Chung Lin
Yih-Jyh Lin
Ying-Chung Lin
Ying-Ting Lin
Yi-Ting Lin
Yi-Tsen Lin
Yi-Tsung Lin
You-Min Lin
You-Shuei Lin
Yuan Lin
Yu-Hsuan Lin
Yu-Hung Lin
Yu-Kai Lin
Yusen Lin
Yu-Shan Lin
Yu-Ting Lin
Z. Ping Lin
Zhang Lin
Zhao Lin
Zhongxu Lin
Zu-Yau Lin
Sera Linardi
Cristina Linares
Marco Linari
Benjamin Linas
Naomi Lince-Deroche
Pasquale Linciano
Marcus Lincoln Smith
Nilton Lincopan
Pedro Lind
Anna Linda
Anders Lindahl
Iris Lindberg
Laura Lindberg
Annika Lindblom
Klaus Linde
Martin Lindegren
Katja Lindel
Catherine Lindell
Michael Lindell
Jan Lindeman
Ulrich Lindemann
Dan Linden
Katrin Lindenberg
David Lindenmayer
Thomas Lindenstrøm
Thies Lindenthal
Jeffrey Linder
Joshua Linder
Maria Linder
Stig Linder
Marc Linderman
Jutta Lindert
Cecilia Lindestam Arlehamn
Helena Lindgren
Jonatan Lindh
Jacob Lindheimer
Bengt Lindholm
Daniel Lindholm
Amanda Lindholm-Perry
Philip Lindner
John Lindo
Catherine Lindon
Kresten Lindorff-Larsen
Diego Lindoso
Steve Lindow
Steven Lindow
Derick Lindquist
John Lindquist
Moritz Lindquist Liljeqvist
Roland Lindqvist
Alec Lindsay
Angus Lindsay
Dhugal Lindsay
Stuart Lindsay
Merry Lindsey
Annika Lindskog
Marcus Lindskog
Maria Lindskog
Carita Lindstedt
Sandra Lindstrom
Miia Lindström
Bernt Lindtjørn
Claes Mikael Lindvall
Antonio Linero
Tara Lineweaver
Chen Ling
Erjun Ling

Kai-Shu Ling
King Hwa Ling
Man Ho Ling
Morris Ling
Shuyang Ling
Xuefeng Ling
Annett Linge
James Lingeman
Christina Ling-Hsing Chang
Chris Lingle
Susan Lingle
Apichart Linhananta
Robert Linhardt
Daniel Linhares
Sultan Linjawi
A. James Link
Alexander Link
Andrew Link
Emma Link
Andrew M. Linke
Claudia Linker
Matt Linkie
Wayne Linklater
Omer Linkovski
Gary Linn
Kristin Linn
Vesa Linnamo
Michael Linnebank
Sebastian Linnemayr
Andreas Linninger
Adriana Lino-Dos-Santos-Franco
Teresa Lino-Neto
Sara Linse
Jakob Linseisen
Anja Linstädter
Anna Lintern
MacRae Linton
François Lintz
Simone Linz
Jjulius Liobikas
Vincenzo Lionetti
Clifford Liongue
Chian-Jiun Liou
Chia-Wei Liou
Jyh-Ming Liou
Ruey-Fen Liou
Ming Han Lincoln Liow
Gregory Lip
Tomislav Lipic
Patrycja Lipinska
Malgorzata Lipinska-Gediga
Brian Lipinski
Robert Lipinski
Peter Lipke
Roger Lippé
Adam Lippert
Frank Lippert
Giordano Lippi
Marco Lippi
Christopher Lippitt
Jocelyn Lippman-Bell
Ethan Lippmann
John Lippolis
John Lipscomb
Leah Lipsky
David Lipson
Ellen Lipstein
Wendy Lipworth
Fabio Lira
Rosalia Lira
Andres Lira-Noriega
Fabrizio Lirer
Stefanie Lis
Robert Lisak
Patricia Lisboa
Sabrina Lisboa
Thiago Lisboa
Andrey Lisitsa
Ton Lisman
Andrey Lissovsky
James Lister
Stephen Lister
Aaron Liston
Ulf Liszkowski
Karen Lithgow
Gianni Liti
Geert Litjens
Antonios Litke

Dzianis Litskevich
Alan Litsky
Jeremy Littell
Chris Little
Christopher Little
Dianne Little
Jonathan Little
Peter Little
Randie Little
Susan Little
Taras Litvin
Sheldon Litwin
Jacqueline Litzgus
Aimin Liu
Aizhong Liu
Angela Liu
Arron Liu
Bang Liu
Bao Liu
Baodong Liu
Bei Liu
Biao Liu
Bin Liu
Bindong Liu
Bing Liu
Binghang Liu
Binghe Liu
Bingqiang Liu
Bingquan Liu
Bingya Liu
Biyue Liu
Bo Liu
Can Liu
Canran Liu
Catherine Liu
Chang Liu
Changwei Liu
Chao Liu
Cheng Liu
Chengfei Liu
Chengshuai Liu
Cheng-Tzu Liu
Chenguang Liu
Chengyu Liu
Chen-Hua Liu
Chen-Yu Liu
Chien-An Liu
Chi-Hsiu Liu
Ching-Ti Liu
Chuan Fa Liu
Chuang Liu
Chuanhe Liu
Chun Liu
Chun-Hung Liu
Chunjiang Liu
Chunlin Liu
Cun-Zhi Liu
Da Liu
Dali Liu
Dazhi Liu
Dengcai Liu
Dianzhi Liu
Dong Liu
Dongcheng Liu
Dongfei Liu
Dongjun Liu
Dongmin Liu
Dun Liu
Enqi Liu
Fabao Liu
Fang Liu
Fei Liu
Feng Chi Liu
Feng Liu
Feng-Bin Liu
Fenyong Liu
G. H. Liu
Gaifen Liu
Gang Liu
Gangcai Liu
Gang-Yu Liu
Guangxu Liu
Guansheng Liu
Gui Liu
Guiqiong Liu
Guodong Liu
Guokun Liu
Haijiang Liu

Haijun Liu
Haipei Liu
Hai-Peng Liu
Haitao Liu
Hao-Li Liu
Haoping Liu
Helene Minyi Liu
Hong Liu
Hongbin Liu
Hongfang Liu
Honglei Liu
Hongling Liu
Hongmei Liu
Hongmin Liu
Hong-Xia Liu
Hongyan Liu
Hongyou Liu
Hongyu Liu
Hu Liu
Huai Liu
Huaiju Liu
Hu-Chen Liu
Hui Liu
I-Hsin Liu
Jeff Liu
Je-Ruei Liu
Jesse Liu
Jian Liu
Jianghua Liu
Jiang-Qin Liu
Jiangtao Liu
Jianhua Liu
Jianv Liu
Jianxing Liu
Ji-Bin Liu
Jie Liu
Jigang Liu
Jihe Liu
Ji-Liang Liu
Jin Liu
Jinfen Liu
Jing Liu
Jingbin Liu
Jinhua Liu
Jinliang Liu
Jinping Liu
Jintao Liu
Jiying Liu
John Liu
Jonathan Liu
Jufen Liu
Jun Liu
Jun-E Liu
Junling Liu
Junting Liu
K. Liu
Kai Liu
Kaidong Liu
Kaiyan Liu
Ke Liu
Kenneth Liu
Ke-Zhi Liu
Kun Liu
Lawrence Liu
Lei Liu
Leyuan Liu
Li Liu
Liang Liu
Liezhao Liu
Li-Kuo Liu
Li-Lian Liu
Lin Liu
Ling Liu
Linghui Liu
Liquan Liu
Lu Liu
Luxiang Liu
Manhua Liu
Manqiang Liu
Maoxing Liu
Meihua Liu
Mei-Lin Liu
Mengyuan Liu
Min Liu
Ming Liu
Ming-Jung Liu
Mingliang Liu
Mingming Liu

Mingxia Liu
Mingyao Liu
Ming-Yie Liu
Minhui Liu
Minmin Liu
Peide Liu
Ping Liu
Pingsheng Liu
Qi Liu
Qian Liu
Qiang Liu
Qiaoquan Liu
Qiji Liu
Qin Liu
Qing Liu
Qingchang Liu
Qing-Feng Liu
Qinghang Liu
Qing-Jie Liu
Qing-Lin Liu
Qingming Liu
Qingsong Liu
Qing-Song Liu
Qiong Liu
Qiuning Liu
Qiu-Ning Liu
Quan Liu
Quanhui Liu
Quanzhong Liu
Renyu Liu
Richard Liu
Rong Liu
Rui Liu
Rui-Tian Liu
Run-Ran Liu
Shan Liu
Shaojun Liu
Shao-Lun Liu
Shelan Liu
Shiguang Liu
Shih Hui Liu
Shikai Liu
Shing Liu
Shiu-Mei Liu
Shuchang Liu
Shu-Wei Liu
Shuyu Liu
Shuzhen Liu
Sichao Liu
Silvia Liu
Song-Mei Liu
Song-Tao Liu
Stanley Liu
Sumei Liu
Suyang Liu
Tao Liu
Teng Liu
Teresa Liu
Thomas Liu
Tian-Liang Liu
Tiffany Ting Liu
Ting Liu
Tong-Xian Liu
Tsunglin Liu
Wansheng Liu
Wei Liu
Weibo Liu
Weiguo Liu
Wei-Jie Liu
Weishu Liu
Weizhi Liu
Wen Liu
Wen-Bin Liu
Wenjun Liu
Wentai Liu
Wen-Tso Liu
Wenzhe Liu
X. Sherry Liu
Xia Liu
Xiang Dong Liu
Xianshuang Liu
Xiao Liu
Xiaodong Liu
Xiaojun Liu
Xiaolei Liu
Xiaolin Liu
Xiao-Long Liu
Xiaoqun Liu

Xiaoyue Cathy Liu
Xiaoyun Liu
Xiaozhong Liu
Xibao Liu
Xinwang Liu
Xinyi Liu
Xiqiang Liu
Xiufan Liu
Xiuwei Liu
Xiuwen Liu
Xiyang Liu
Xuan Liu
Xuhui Liu
Xun Liu
Xu-Ping Liu
Xuyang Liu
Y. Liu
Yajun Liu
Yan Liu
Yang Liu
Yanhong Liu
Yan-Jun Liu
Yanyun Liu
Yaou Liu
Ye Liu
Yen-Wen Liu
Yi Liu
Yi-Chien Liu
Yichuan Liu
Yifan Liu
Yifei Liu
Yilin Liu
Yilun Liu
Yimin Liu
Ying Liu
Ying-Bin Liu
Yitao Liu
Yi-Wen Liu
Yong Liu
Yongchao Liu
Yongfeng Liu
Yongxin Liu
Yong-Zhong Liu
Youjun Liu
Yu Liu
Yuan Liu
Yuanhui Liu
Yu-Chi Liu
Yudong Liu
Yue Liu
Yuelin Liu
Yuhe Liu
Yuhong Liu
Yuhuan Liu
Yushi Liu
Yuzhou Liu
Zaiyi Liu
Zewen Liu
Zhanqiang Liu
Zhen Liu
Zhenhua Liu
Zhenyu Liu
Zhi-Hai Liu
Zhihong Liu
Zhihua Liu
Zhijie Liu
Zhi-Jin Liu
Zhijun Liu
Zhipeng Liu
Zhi-Ping Liu
Zhiqin Liu
Zhiyong Liu
Zhiyuan Liu
Zhongbo Liu
Zhonghao Liu
Zhonghua Liu
Zhongyuan Liu
Zhuangzhuang Liu
Ziyi Liu
Zonghua Liu
Zu-De Liu
Feng Liu-Smith
Eli Liverani
Helen Liversidge
Marco Livesu
Ido Liviatan
Kimberly Livingston
Michael Livingston

Gregory Livshits
Zhu Liwu
Lisa Lix
Gerard Lizard
Jose Lizarraga
Karlo Lizarraga
Maria Giovanna Lizio
Eva Liznarova
Mila Ljujic
Britt-Marie Ljung
Lotta Ljung
Maria Ljungberg
Mats Ljungman
Olle Ljungqvist
Bastien Llamas
Daniel Llano
Alejandro Llanos-Cuentas
Carmen Llena
John Llewelyn
Jorge Llibre
Rafael Llinas
Joaquim Llisterri
Nùria Lloberas
Juan Llopis
Oscar Llopis
Cristina Llopis-Belenguer
Joel Llopiz
Belinda Lloyd
Charlie Lloyd
James Lloyd
Joyce Lloyd
Richard Lloyd
Thomas Lloyd
Peter Lloyd-Sherlock
Mari Lloyd-Williams
Sara Llufriu
Bernice Lo
Camilla Lo
Christopher Lo
Chu-Fang Lo
Clement Lo
Clive Lo
Janet Lo
Jeng-Fan Lo
Mentzung Lo
Michael Lo
Ronson Sze Long Lo
Shyh-Ching Lo
Warren Lo
Yen-Li Lo
Ying-Ru Lo
Alessandra Lo Presti
Vincent Lo Re
Hugo A. Loaiciga
Daniel Lobato
Elizabeth Lobb
Steven Lobello
Gerald Lobley
Dileep Lobo
Eunice Lobo
Francisco José Lobo
Jorge Lobo
Jose Lobo
Michelle Lobo
Ronstan Lobo
Sonja Lobo
Susana Lobo
Malgorzata Lobocka
Agnieszka Loboda
Donald Lobsien
Sergio Locaputo
Joseph Locascio
Francesco Locatelli
Ken Locey
Carolina Loch
Robert Lochhead
Adam Lock
Brittanie Lockard
Robert Locke
Caryl Lockhart
Joey Lockhart
Nicole Lockhart
Sarah Lockie
Shahin Lockman
Michael Lockwood
Patricia Lockwood
Jocelyn Lockyer
Médéa Locquet

Robert Locy
Elisabeth Lodder
Andrew Lodge
Nilanjan Lodh
Alessia Lodi
Sara Lodi
Melissa Lodoen
Lorenzo Lodola
Gregory Lodygensky
Stacy Loeb
Susan Loeb
Nicolas Loebel
Mike Loeffelholz
Willie Loeffen
Diane Loeffler
Ivonne Loeffler
Henry Loeffler-Wirth
Jeremy Paul Loenneke Loenneke
Adrian Loerbroks
Nils Loewen
David Loewenstein
Zvi Loewy
Eric Lofgren
Jennifer Lofgren
Sarah Lofgren
Antonio Loforte
Christer Lofstedt
Erika Löfström
Pooja Loftus
James Logan
Malcolm Logan
Ryan Logan
Sreemathi Logan
Timothy Logan
Nanthakumar Loganathan
Tharani Loganathan
Carmen Logie
Margaux Lognoul
Giancarlo Logroscino
Craig Logsdon
Brian Logue
Catherine Logue
Mark Logue
Liyen Loh
Siew Yim Loh
Thomas Loh
Tze Ping Loh
Vanessa Loh
Y. Peng Loh
Jayant Lohakare
Monika Lohani
Gertrud Lohaus
Rolf Lohaus
Ebba Lohmann
Theobald Lohmueller
Max Löhning
Michael Lohoff
Benedikt Lohr
Nicole Lohr
Matthias Löhr
Andrew Lohrer
Ansgar Lohse
Barbara Loi
Claire Loiseau
Nicolas Loiseau
Julie Loisel
Alayna Loiselle
Bette Loiselle
Rocio Loizaga de Castro
Joseph Loizzo
Fernando Lojudice
Judith Lok
Mun Fai Loke
Seng Cheong Loke
Belur Lokesh
Yuliya Lokhnygina
Stephen Lokitz
Anders Lokke
Ann-Kristina Løkke
Anna Lokshin
Irakli Loladze
Donald Lollar
André Lollert
Vincent Lomabardi
Niklas Loman
Richard Lo-Man
Elizabeth Lomas
Silvia Lomascolo

Antonella Lomauro
Dean Lomax
Ermelinda Lomazzo
Carl Lombard
Catherine Lombard
Fabien Lombard
Marlize Lombard
Angela Lombardi
Jason Lombardi
Raúl Lombardi
Fabrizio Lombardo
Franklin Lombardo
Michael Lombardo
Umberto Lombardo
Alejandro Lomniczi
Karen Lomond
A. Lonardo
Amedeo Lonardo
Natasa Loncarevic-Vasiljkovic
Nir London
Stephanie London
Maria Londoño
Anna Mari Lone
Deborah Long
Elizabeth Long
Elliot Long
Hai Long
Hong Long
Jeremy Long
Jinyi Long
Jun Long
Lawrence Long
Mark Long
Matthew Long
Meixiao Long
Michael Long
Nathan Long
Quanshan Long
Richard Long
Robert Long
Sara Long
Scott Long
Wenxing Long
Xiaochun Long
Yicheng Long
Ying Long
Yong Long
Zaiyang Long
Mariachiara Longarzo
Adhemar Longatto-Filho
Joshua Longbottom
Todd L. Longbottom
P. W. Longest
Ana Leda Longhini
Cédric Longin
Nicky Longley
Rhea Longley
Cai Longman
Patricia Longmuir
Benedetto Longo
Katie Longo
Larissa Longo
Laura Longo
Matthew Longo
Patrizia Longone
Nicholas Longrich
Francesco Longu
John R Lonks
Evelyn Loo
Keatwei Loo
Siaw-Chuing Loo
Yueh-Ming Loo
Rolf Lood
Torey Looft
Loren Looger
Kevin Looi
Wilhelmina Mijntje Looman
Bruno Loos
Anne Loosen
Gary Lopaschuk
Kristina Lopatiene
Anatoli Lopatin
Ana Lopes
Ana Patrícia Lopes
Carina Lopes
Coeli Lopes
Graciliana Lopes
John Lopes

José Lopes
Luciane Lopes
Perdita Lopes
Vanessa Lopes
Vítor Lopes
Fernando Lopes da Silva
Rodrigo Lopes Ferreira
Leila Lopes-Bezerra
Manuel Lopes-Lima
Camila Lopes-Ramos
Bernard Lopez
Christophe Lopez
Jose Hector Lopez
Michael Lopez
Susana Lopez
Vanessa Lopez
María López
Saioa López
Guadalupe Lopez Contreras
Evangelina López de Maturana
Juan Lopez Gappa
Pilar López Larrubia
Marcelo Lopez Lastra
Amanda Lopez Picado
Monserrat López Sanmartín
Julio Lopez-Aban
Juan Lopez-Alvarenga
Rafael Lopez-Andujar
Adria Lopez-Baucells
Jose Luis López-Campos
Andrés López-Cortés
Jose Lopez-Escamez
J. López-García
Hugo Lopez-Gatell
Mariana Lopez-Gongora
Patricio Lopez-Jaramillo
Liliana López-Kleine
Marcelo López-Lastra
Lucia Lopez-Lopez
Marisol López-López
Cuauhtémoc López-Martín
Joel Lopez-Meza
V. Lopez-Miranda
Horacio Lopez-Nicora
Luisa Lopez-Ochoa
Jorge Ramón López-Olvera
Roberto López-Píriz
Jordi López-Pujol
Andrés López-Rubio
Laura María Lopez-Sanchez
Emlia Lopez-Solanilla
Jorge Lopez-Tello
Francisca López-Torrecillas
Carlos López-Vaamonde
Beatriz López-Valcárcel
Yolanda López-Vidal
Leonardo Lopiano
Benjamin Lopman
Giovanni Loprete
Irene Lorand-Metze
Havard Loras
J. Lora-Tamayo
Heidi Lord
Sue Lord
Karin Lore
Kimberly Lorenz
Timo Lorenz
Stephan Lorenzen
Silvio Lorenzetti
Valentina Lorenzetti
Guillermo Lorenzo
Oscar Lorenzo
Justo Lorenzo Bermejo
Alicia Lorenzo Figueiras
Massimo Lorenzoni
Laura Lorés-Motta
Cristian Loretelli
Elgion Lucio Loreto
Vittorio Loreto
Analia Loria
Francesco Loria
Stephanie Loria
Rik Lories
Dénes Lőrinczy
Sophie Lorioux
Pedro Lorite
Gauthier Loron
Thomas Lorscheid

Joannie Lortet-Tieulent
Hugues Lortie-Forgues
Alessio Lorusso
Robert Lorway
Marek Los
Giovanni Losano
Emily Loscalzo
Annabel Losecaat Vermeer
Molly Losh
Aba Losi
Victoria Losinski
Scott Loss
Kristina Lossow
Tamar Lotan
Jörn Lötsch
William Lott
Fabien Lotte
Hannelore Lotter
Matt Lotter
Joost Lötters
Guilherme Lotufo
Irineu Loturco
Martin Lotze
Edmond Lou
Fangyuan Lou
Hua Lou
Jianlin Lou
Min Lou
Peian Lou
Qingqing Lou
Stina Lou
Yixue Lou
Yonggen Lou
Celine Louapre
Karine Louati
Christian Loubert
Paul Loubet
Remi Louf
John Loughlin
Kevin Loughlin
Thomas Loughran Jr.
Alexander Louie
Kenway Louie
Alain Louilot
Julien Louis
Petra Louis
Valère Lounnas
Karen Lounsbury
Marcelo Loureiro
Barbara Lourenco
Anália Lourenço
Flávio Loures
Ricardo Louro
Rita Louro Guerreiro
Diana Lousa
Maria Lousada-Ferreira
Mona Loutfy
Charalampos Loutradis
Heleni Loutrari
Rogier Louwen
Shavahn Loux
Sandro Lovari
Francesca Lovat
Luigi Lovato
Cara Love
Emma Love
Marian Loveday
Gabor Lövei
David A. Lovejoy
Cheryl Lovelady
Jasmine Loveland
John Lovell
Ric Lovell
Andrew Lover
Marlina Lovett
Mark A. Lovich
Kathryn Loving
Julie Lovshin
Andrea Low
Bobbi Low
Boon Low
Daren Low
Karen Low
Lian Leng Low
Liying Low
Anna Lowe
Brian Lowe
Dawn Lowe

Jean Lowe
Lynn Lowe
Rachel Lowe
Robert Lowe
Siegrid Löwel
Clifford Lowell
Mark Lowenberg
Hubert Löwenheim
Brian Lower
Susan Lowerre-Barbieri
Eric Lowet
Christopher Lowry
Paul Lowry
Andrew Lowther
Andre Loxton
Deborah Loxton
Anna Loy
Bryan Loy
Clement Loy
Yossi Loya
Prashant Loyalka
Kerrie Anne Loyd
Sofie M. M. Loyens
Rodrigo Loyola Muñoz
Francisco Lozano
Juan Manuel Lozano
Oscar Lozano
Pedro Lozano
Santiago Lozano-Calderon
Rosa Lozano-Duran
Lorena Lozano-Sufrategui
Kristina Loziene
Anrui Lu
Baisong Lu
Bingwei Lu
Catherine Lu
Chaofu Lu
Chia-Chen Lu
Chih-Hao Lu
Chun Lu
Chunming Lu
Chun-Yi Lu
Congming Lu
Daru Lu
Fei Lu
Feng Lu
Fung-Jou Lu
Gang Lu
Ganghong Lu
Guang Ming Lu
Guangquan Lu
Gui Lu
Guoquan Lu
Guoyun Lu
Han Lu
Hong Lu
Hongfei Lu
Hongzhou Lu
Hsiangchia Lu
Hsuan-Hsuan Lu
Huchuan Lu
Jenny Lu
Jianquan Lu
Jianxin Lu
Jing Lu
Jinsong Lu
Jun Lu
Ke Lu
Ko-Hsiu Lu
Kun Lu
Kuo-Cheng Lu
Lei Lu
Limin Lu
Lingeng Lu
Liqun Lu
Lu Lu
Mengzhu Lu
Ming Lu
Minghui Lu
Ming-Wei Lu
Ning Lu
Peiou Lu
Qing-Bin Lu
Qiulun Lu
Rongwen Lu
Rongxing Lu
Rui Lu
Ting Lu

Wei Lu
Xia Lu
Xiaochun Lu
Xiaowei Lu
Xiaoyin Lu
Xiaoying Lu
Xing Lu
Xiyuan Lu
Xuyang Lu
Yanhui Lu
Yanrong Lu
Yanxia Lu
Ya-Peng Lu
Yi Lu
Yihe Lu
Ying-Jie Lu
Yingli Lu
Yingmin Lu
Yong Lu
Yongbo Lu
Yonggang Lu
Yongke Lu
Yun Lu
Zheng Lu
Zheng-Rong Lu
Zhentai Lu
Zhiqiang Lu
Zhongxian Lu
Ziyu Lu
Zuhong Lu
Ling Lü
Zhi-Chuang Lü
Sen Luan
Yun-Xia Luan
David Lubell
Mark Lubell
Farah Lubin
Yael Lubin
Gitta Lubke
Zelmina Lubovac-Pilav
Valter Lubrano
Jim Luby
Joan Luby
Stephen Luby
Angius Luca
Corneliu Luca
Di Marco Luca
Mark Lucanic
Claudia Lucarelli
Alexander Lucas
Ana Lucas
Cathy Lucas
Christopher Lucas
Emma Lucas
Gregory Lucas
Joseph Lucas
Richard Lucas
Spencer Lucas
Stuart Lucas
Dani Lucas-Barbosa
Elena Lucchetta
Giancarlo Lucchetti
Maristella Lucchini
Reinaldo Lucena
Ersilia Lucenteforte
Sean Lucey
Adriano Lucheta
Martina Luchetti
Claudio Luchini
Nestor Luchini
Fabio Luciani
Alberto Maria Luciano
Olivier Lucidarme
Vanessa Lucieer
Daniela Lucini
Marília Lucisano
Katharina Lückerath
Tim Luckett
Tracy Luckhardt
Marcin Luckner
Tyrone Lucon-Xiccato
Serena Lucrezi
Marian Luctkar-Flude
Dominic Luc-Webb
Artur Luczak
Jasmina Luczo
Julia Ludewig
Markus Lüdi

William Ludington
O. Ludka
Stuart Ludsin
Richard Luduena
Björn Ludwar
Andreas Ludwig
Arne Ludwig
Mike Ludwig
Roland Ludwig
Lih-Fen Lue
Neal Lue
Aaron Luebbe
Torben Luebbe
Georg Luebeck
Justin Luebke
Hartmut Lueddens
Steffen Luedeke
Maximilian Luehr
Ramon Luengo-Fernandez
Claus Lüers
Darron Luesse
Friedrich Luft
Thomas Luft
Micah Luftig
Emanuele Lugato
Aurelia Lugea
Dror Luger
Edwin Lughofer
Isaac Luginaah
Marc Luginbühl
Gustavo Lugo Goytia
Dih-Ling Luh
Chi-Wai Lui
Kelvin Fai Hong Lui
Sylvia Lui
Marco Luigetti
Maartje Luijten
Diana Luise
Nagendra Luitel
Osmar Luiz
Thomas Luiz
Anderson Luiz-Ferreira
Nathan Lujan
Rafael Lujan
Kelvin Luk
Martin Lukac
John Lukacs
Alexander Lukashev
Katarzyna Lukasiuk
Henry Lukaski
David Lukatsky
Nancy Luke
Ryan Lukeman
Julius Lukes
Tomas Lukes
Suryani Lukman
Vimoksalehi Lukoschek
Magdalena Lukowiak
Wolfgang Lukowitz
Angela Lukowski
Deus Lukoye
Francois Luks
Jolanda Luksenburg
Gedi Luksys
Pavel Lukyanov
Dorothée Lulé
Matteo Lulli
Fok Moon Lum
Julian J. Lum
Jean-Pierre Lumaret
Bruno Lumbroso
Serge Lumbroso
Stefano Luminari
Mark Lumley
Zhao-Rong Lun
Brian Luna
José D. Luna
Jose D. Luna Pinto
Sandra Luna-Fineman
Klaus Lunau
Hilda Luna-Záizar
Amie Lund
Hannah Lundberg
Kristjen Lundberg
Patric Lundberg
Thorbjörn Lundberg
Thomas Lundeberg
Fredrik Lundell

Taina Lundell
Iben Lundgaard
Benjamin Lundgren
Marjorie Lundgren
Andreas Lundh
Morten Lundh
Torbjorn Lundh
Daniel Lundin
Knut Lundin
Richard Lundmark
Christofer Lundqvist
Mikael Lundqvist
Kenneth Lundstrom
David Lundy
Sebastian Lunemann
Jan Lünemann
Chi-Wen Lung
Anusorn Lungkaphin
Codrin Lungu
Dan Lunney
Mariia Lunova
Biquan Luo
Chaoxi Luo
Chen Luo
Chunling Luo
Daya Luo
Dehong Luo
Fang-Li Luo
Feijun Luo
Gang Luo
Guojie Luo
Hao Luo
Haoxiang Luo
Jake Luo
Jianhua Luo
Jiawei Luo
Jiebo Luo
Jincai Luo
Jing Luo
Jingqin Luo
Jinming Luo
Jon Luo
Juhua Luo
Jun Luo
Junhai Luo
Kaijun Luo
Li Luo
Linlin Luo
Lixin Luo
Ming Luo
Min-Hua Luo
Minnan Luo
Pei Luo
Qian Luo
Qingwei Luo
Renfu Luo
Rong Luo
Tao Luo
Ting Rong Luo
Tingjin Luo
Wanjun Luo
Wei Luo
Weijun Luo
Wenting Luo
Xiangang Luo
Xianghua Luo
Xianguo Luo
Xiaofeng Luo
Xiao-Guang Luo
Xiaolan Luo
Xiaoping Luo
Xiaozhou Luo
Xin Luo
Xiong-Jian Luo
Yan Luo
Yawei Luo
Yi Luo
Yi-Bo Luo
Yi-Jyun Luo
Yong Luo
Yongfeng Luo
Yu Luo
Yuan Luo
Yu-Heng Luo
Zhehui Luo
Zhenyao Luo
Zhibin Luo
Zisheng Luo

Livio Luongo
Oleg Lupan
Angela Lupattelli
Graziana Lupattelli
Antonella Lupetti
Pietro Lupetti
Christopher Lupfer
Darío Lupiáñez
Blanca Lupiani
Gabriella Lupo
Amelie Lupp
Fabrizio Luppi
Ivan Luptak
Capilla Luque
Ignacio Luque
Juan Luque-Larena
Amandine Luquiens
Nell Lurain
Shai Luria
Christoph Lürig
John Lurquin
Christian Luschnig
Francis Luscinskas
Elizabeth Lusczek
Peter Lush
Sofya Lushchekina
Bethany Lusk
Matthew Luskin
Katherine Lust
Maryam Lustberg
Glen Lutchman
May Lutfiyya
Marc Lütgehetmann
Joe Lutkenhaus
Evan Lutkenhoff
Pavlo Lutsik
Erika Lutter
Randy Luttrell
Frederick Lutz
Manfred Lutz
Michael Lutz
Chi Luu
R. Luwor
Christine Luxemburger
Wang Luya
Carlos Luz
Paula Luz
Daniela Luzi
Livio Luzi
Katherine Luzuriaga
Roberto Luzzati
Chen Lv
Jiale Lv
Peng Lv
Xuewei Lv
Yanfeng Lv
Ying Lv
Yingjun Lv
Zhao Lv
Zhaozhi Lv
Cheng Ly
Donald Lyall
Ruth Lyck
David Lydall
Deirdre Lyell
Anne Lyerly
David Lyerly
Michael J. Lyerly
Annmarie Lyles
Christopher Lyles
Lee Lyman
Stephen Lyman
Petros Lymberakis
Anastasios Lymperopoulos
Despoina Lymperopoulou
Antony Lynam
Chris Lynch
Christopher Lynch
Conor Lynch
David Lynch
Lauren Lynch
Lori Lynch
Michael Lynch
Penelope Lynch
Ryan Lynch
Scott Lynch
Shalini Lynch
Shannon Lynch

Robert Lynd-Stevensen
Heidi Lyng
Elsebeth Lynge
Gholson Lyon
Richard Lyon
Antonia Lyons
David Lyons
Ian Lyons
Jeri-Anne Lyons
Keith Lyons
Leslie Lyons
Nick Lyons
Traci Lyons
Kateryna Lypetska
Divaldo Lyra Junior
Philippe Lyrer
Inna Lysnyansky
Błażej Łyszczarz
Theodore Lytras
Lyubomyr Lytvynchuk
Jiankun Lyu
Nengchao Lyu
Ping-Chiang Lyu
Qifeng Lyu
Shijie Lyu
Yuanyuan Lyu
Kalle Lyytinen
Corry M.
Krishna Priya M.
Li M.
M. Ebrahim M. Mashat
Bexci M. S.
Anthony Ma
Baohua Ma
Bin Ma
Bing Ma
Bo Ma
Changqi Ma
Chang-Sheng Ma
Changxi Ma
Changyan Ma
Chaoran Ma
Che Ma
Chuanxi Ma
Chunhua Ma
Dan Ma
Dan-Dan Ma
David Ma
Deqin Ma
Dong Ma
Dongyun Ma
Duan Ma
Enlong Ma
Fei Ma
Fengyun Ma
Guojia Ma
Haiting Ma
Hang Ma
Hao Ma
Haoli Ma
Hongming Ma
Jian Ma
Jianbing Ma
Jianhua Ma
Jian-Hua Ma
Jiantao Ma
Jihui Ma
Jilin Ma
Jinjin Ma
Jixiang Ma
Jun Ma
Jun-Yu Ma
Ka Yan Ma
Keming Ma
Kesen Ma
Keyi Ma
Li Ma
Lijun Ma
Linna Ma
Long Ma
Maode Ma
Meihu Ma
Ning Ma
Pengda Ma
Qi Ma
Qing Ma
Qingguo Ma
Qingyan Ma

Qingyi Ma
Rong Ma
Ruijuan Ma
S. N. Ma
Sai Ma
Teng Ma
Thomas Ma
Wan-Li Ma
Weihua Ma
Weimin Ma
Weiqiang Ma
Wen Ma
Wenjun Ma
Wenming Ma
Xiao Ma
Xiaobing Ma
Xiaogang (Marshall) Ma
Xiaohong Ma
Xiaojing Ma
Xiao-Kai Ma
Xiaoxiao Ma
Xiaoyin Ma
Xin-Rong Ma
Xuelei Ma
Yalong Ma
Yaoming Ma
Yina Ma
Ying Ma
Yitong Ma
You-Zhi Ma
Yuan Ma
Yuehui Ma
Yuqin Ma
Yushui Ma
Yutao Ma
Zhe Ma
Zhengqiang Ma
Zhen-Qiang Ma
Zhiwei Ma
Zhiyong Ma
Zhonghua Ma
Selma Maacha
Steffen Maak
Thomas Maal
Amy Maas
Angela Maas
Bea Maas
H. A. Maas
Miriam Maas
Renke Maas
Rutger Maas
Anne Maass
Karen Maass
Imad Maatouk
Ulaganathan Mabalirajan
Musa Mabaso
Angela Mabb
Neil Mabbott
Katsuhiro Mabe
Nicola Mabjeesh
Jon Mabley
Tomotaka Mabuchi
Yo Mabuchi
Micheál Mac Aogáin
Pádraig Mac Carron
Caoimhin Mac Giolla Phadraig
Neil Mac Parthaláin
John Mac Sharry
J. Maca
Emiliano Macaluso
Kevin Macaluso
Aline Macarevich
Donia Macartney-Coxson
Richard Macatee
Nanna MacAulay
Rebecca MacAulay
Valentine Macaulay
Angus MacBeth
Marco Maccaferri
Beth MacCallum
Donna MacCallum
Laura Maccari
Anita MacDonald
Chancey MacDonald
Clinton MacDonald
James MacDonald
John MacDonald
Marjorie MacDonald

Niall Macdonald
Noni MacDonald
Robert Macdonald
Ross MacDonald
Stuart Macdonald
Corrie Macdonald-Wallis
C. W. MacDonell
Frederick MacDonnell
Judith MacDonnell
Colin MacDougall
Scott MacDougall-Shackleton
Emma Mace
Mark MacEachern
Alexandre Macedo
Antonio Macedo
Derrick MacFabe
Bruce MacFadden
Sarina Macfadyen
Jenny Macfie
Ian MacGregor-Fors
François Mach
John Mach
Nuria Mach
Khaled Machaca
Diana Machado
Elisabete Machado
Elizabeth Machado
Fabiana Machado
Glauco Machado
Gustavo Machado
Heather Machado
Ivana Machado
J. Tenreiro Machado
Jorge Machado
José Machado
Mariana Machado
Nathália Machado
Pedro Machado
Ricardo Machado
Roberto Machado
Rosangela Machado
Zurab Machaidze
Zdenko Machala
Dorothy Machalek
William Macharia
Melissa Mache
Randy Machemehl
Terry Machen
Janet Macher
Olivier Macherey
Peter Macheroux
Edouard Machery
Laurent Machet
Venkata Machha
Kiego Machida
David Machin
Maria Teresa Machini
Juan Machin-Mastromatteo
Mercilene Machisa
Kazuyo Machiyama
Katerina Machova Polakova
Kelly Machovec
Gabriel Machovsky
Fernando Macian
Emma del Carmen Macías-Cortés
Léa Maciel
Manoela Maciel
Rafael Maciel-de-Freitas
Janet MacInnes
Catriona Macinnis-Ng
Andrew Macintyre
David MacIntyre
Michael Maciosek
Salvador Macip
Hugh MacIsaac
Rachael MacIsaac
Nancie MacIver
J. Scott MacIvor
Isabelle Mack
Katya Mack
Alex Mackay
Alexander Mackay
Clare Mackay
D. J. G. Mackay
Dylan MacKay
James MacKay
John MacKay

Anna MacKay-Brandt
Emilie Macke
Joreintje Mackenbach
Darryl MacKenzie
Derek Mackenzie
Grant Mackenzie
Lisa Mackenzie
Sasho James Mackenzie
Tippi MacKenzie
Sarah Jane Mackenzie Ross
Alexander MacKerell
David Mackey
Tim Mackey
Ken Mackie
William Mackin
Alexander Mackinnon
Steve Mackinson
Shylie Mackintosh
Heath Mackley
Stephen Macko
Erich Mackow
Julian Maclaren
Shannon MacLaughlan David
James Maclean
Mary Maclean
Calman MacLennan
Steven MacLennan
Amy MacLeod
Malcolm Macleod
Megan MacLeod
Norman MacLeod
Stuart MacLeod
Amanda MacLoed
Katie MacLure
Clare MacMahon
Heath MacMillan
Lee Ann MacMillan-Crow
Andrew Macnab
Áine MacNamara
Brooke Macnamara
Katherine MacNamara
S. MacNeil
Jill Macoska
Sonya MacParland
Iain MacPhee
Ross MacPhee
Enrique Macpherson
Peter MacPherson
Rebecca MacPherson
Thomas MacRae
Christopher MacRaild
Nathalie Macrez
Erin Macri
Francesco Macri
John MacSharry
Jason MacTaggart
Jean-Marc Mac-Thiong
Thomas MacVittie
Aaron Macy
Jonathan Macy
Michael Macy
Srideshikan Madabushi
Syra Madad
Vince Madai
Barbara Madaj
Claudia Madampage
Karan Madan
Taruna Madan
Ali Madani
Kaveh Madani
Rupalatha Maddala
Rohan Maddamsetti
Ros Madden
Victoria Madden
Ted Maddess
Joseph Dylan Maddox
Paul Maddox
Mohan Maddur
Jean-Yves Madec
Yoann Madec
Giordano Madeddu
Joao Madeira
Luciana Madeira da Silva
Pascal Madeleine
Malcolm Maden
Frank Madeo
Simone Mader
Geraldo Mäder

Patrick Mäder
Stephan Madersbacher
Panagiotis Madesis
A. Madhankumar
Guruprasad Madhavan
Sangeetha Madhavan
Kayalvizhi Madhivanan
Neha Madhiwala
Nilesh Madhu
Rama Sashank Madhurapantula
Michael Madigan
Jilian Madine
Gabriel Madirolas
B. Madison
Diane Madlon-Kay
Rosalinda Madonna
Fahima Madouri
Jorge Fonseca Madrigal
Grasiele Scaramal Madrona
Christian Madry
Anne-Mette Madsen
Joseph Madsen
Ole Madsen
Steen Madsen
Darin Madson
Francisco Maduell
Parthasarathy Madurantakam
Akiko Maeda
Eduardo Maeda
Guilherme Jorge Maeda
Isseki Maeda
K. Maeda
Ken Maeda
Naoyuki Maeda
Shin Maeda
Yasuhiro Maejima
Silje Maeland
Daniel D. Maeng
Pil Jae Maeng
Sumiaki Maeo
Steven Maere
Michael Maerker
Miet Maertens
Charline Maertens de Noordhout
Dominiek Maes
Dries Maes
J. H. R. Maes
Michael Maes
Peter Maes
Burkhard Maess
Martijn Maessen
Francesca Maestrelli
Michelangelo Maestri
Renan Maestri
Elena Maestrini
Arno Maetens
Arianna Maffei
Andrea Mafficini
Tania Maffucci
Nicola Maffulli
Nyashadzaishe Mafirakureva
Denise Mafra
F. Mafra
Isabel Mafra
Elizabeth Maga
Pedro Magalhaes
Tereza Magalhaes
Fernão Magalhães
Lizandra Magalhães
Ernesto Magallon-Neri
Everett Magann
Srihari Maganti
Jules Magda
Ahmed Maged
Dixie Mager
Markus Magerl
Aude Magerus-Chatinet
Adriana Maggi
Fabrizio Maggi
Mario Maggi
Pietro Maggi
Alberto Maggiani
Roberto Maggio
Marcello Maggiolini
Umberto Maggiore
Jennifer Maggs

Kesson Magid
Arthur Magill
Francis Magilligan
Thomas Magin
Melissa Maginnis
Marcelo Magioli
Emmanouil Magiorkinis
Gkikas Magiorkinis
Paolo Magistri
Lucia Magis-Weinberg
Adriano Magli
Dangelo Magliano
Alessio Maglio
Nicholas Magliocca
Karl Magnacca
Christophe Magnan
Renee Magnan
Rodrigo Magnani
Paola Magnano
Elodie Magnanou
Marcelo Magnasco
Jean-Francois Magnaval
Nicola Magnavita
Ronald Magness
Paolo Magni
Laio Magno
Claire Magnon
Brooke Magnus
Carsten Magnus
Maria Magnus
C. G. Magnussen
Steen Magnussen
Mans Magnusson
Nils Magnusson
Ulf Magnusson
Gesham Magombedze
Dimitrios E. Magoulioti
Lorenzo Magrassi
Donatella Magri
Sara Magrini
Rafael Magris
Fernando Magro
Emmanuelle Maguin
Kaitlin Clare Maguire
Lynn Maguire
Mahon Maguire
Tibor Magura
Amir Mahabadi
Sanjiv Mahadeva
Abhishek Mahajan
G. R. Mahajan
Harshal Mahajan
Supriya Mahajan
Yatin Mahajan
Prathapkumar Mahalingaiah
Ashwin Mahalingam
Michael Mahan
Wahib Mahana
Michael Mahande
M. Mahapatra
Rajanikanta Mahapatra
Saugata Mahapatra
Sidharth Mahapatra
Munazzam Ali Mahar
Rohan Maharaj
Surakameth Mahasirimongkol
Sushil Mahata
Preeti Mahato
Mahboobeh Mahdavinia
Hamid Mahdiuni
Fréderic Mahé
Liam Mahedy
Rathi Mahendran
Bathsheba Mahenge
Brady Maher
Jaclyn Maher
Sean Maher
L. Maher III
Zaynah Maherally
M. S. Mahesh
Ankit Maheshwari
Mathieu Maheu-Giroux
Ahmed Mahfouz
Magdy Mahfouz
Mohamed Mahfouz
Amal Mahfoz
Rifat Mahfuza
Lenin Mahimainathan

Ata Mahjoubfar
Mersiha Mahmic-Kaknjo
Salman Mahmood
Bakhtawar Mahmoodi
Zohreh Mahmoodi
A. Mahmoodpoor
Ahmed Mahmoud
Haytham Mahmoud
Mahdi Mahmoudi
Tokameh Mahmoudi
Jalal Mahmud
Nadim Mahmud
Mehmet Mahmut
Andrea Mahn
John Mahoney
Megan Mahoney
Patrick Mahoney
James Mahony
Jennifer Mahony
Shaun Mahony
Timothy Mahony
Zahra Mahoor
Amin Mahpour
Claudius Mahr
James Mahshie
Chun-Wai Mai
Xiaoqin Mai
Cristiane Maia
Lucianne Maia
Inbal Maidan
Daniel Maidana
Shachar Maidenbaum
Ekaterina Maidji
Ian Maidment
Cornelia Maier
Hannes Maier
Jeanette Maier
Johanna Maier
Katharina Maier
Werner Maier
Abdoulaye Maïga
Nicolas Maillard
Arnaud Mailleux
Lisa Mailleux
Ryan Mailloux
Dennis Maiman
Michal Maimaran
Eric Maimela
Anson Main
Bradley Main
Mercy Maina
John Maingi
Jacques Mainil
Joel Mainland
James Mainprize
Richard Mains
P. Stanely Mainzen Prince
Vittorio Maio
Francesco Maione
Rafael Maior
Luigi Maiorano
Maria Maiorino
Johannes Mair
Patrick Mair
Valerio Mais
Tim Maisch
Fiona Maisels
John Maisey
Carine Maisse
Lara Maistrelleo
Hiroshi Maita
Shishir Maithel
George Maiti
Mrinal Maiti
Panchanan Maiti
S. Maiti
Arindam Maitra
Pushkar Maitra
Ranjan Maitra
Nathalie Maitre
Jean-Léon Maître
Luigi Maiuri
Antonio Majdandzic
Barbara Majello
Vladimir Majerciak
Jennifer Majersik
Matthias Majetschak
Wojciech Majewski

Dewan Majid
Farhan Majid
Shideh Majidi
Laleh Majlessi
Ignazio Majolino
Edith Majonga
Marian Major
Laszlo Majoros
Shannon Majowicz
Muhammad Majrooh
Ireneusz Majsterek
Adhip Majumdar
Subeer Majumdar
Maimuna Majumder
Mrinmoyee Majumder
Rupamanjari Majumder
Kwok-Kei Mak
Kepher Makambi
Prestige Makanga
Prestige Tatenda Makanga
Agnieszka Makara
Mariano Makara
Vladimir Makarov
George Makedonas
Juho-Antti Mäkelä
Miia Mäkelä
Pirjo Mäkelä
Benjamin Makepeace
Issam Makhoul
Alexander Maki
Hisataka Maki
James Maki
Takakuni Maki
Oluwole Daniel Makinde
Markus Makinen
Shigeki Makino
Tomoko Makishima
Kaouthar Makni
Yoshida Makoto
Elena Makovac
Peter Makovicky
I. Joanna Makowska
Carolina Makowski
László Makra
Christos Makris
Stergios Makris
Grigory Maksaev
Hernán Makse
Elira Maksuti
Alexey Makunin
Maria Makuwa
Luiz Malabarba
Juan Malacara
Magdalena Malachowicz
Leonel Malacrida
Laurence Malandrin
Andrea Malandrino
Umberto Malapelle
Laurent Malaquin
Yegor Malashichev
Davide Malatesta
Francesco Malatesta
Paolo Malatesta
E. M. Malathy
Lorenzo Malatino
Nuria Malats
Luc Malaval
Marco Malavolta
Hamish Malcolm
Rosalind Malcolm
Frank Maldarelli
Alpeshkumar Malde
Maricela Maldonado
Rosa Maldonado
Carmen Maldonado-Bernal
Amanda Male
Victoria Male
Kristen Malecki
Jacek Malejczyk
Goldis Malek
Sami Malek
Žiga Malek
Michael Malek-Ahmadi
Soheila Maleki
Saman Maleki Vareki
Mohsen Malekinejad
Katarzyna Malenczyk Malenczyk
René Malenfant

Giovanni Malerba
Mario Malerba
Paola Malerba
Chrisovalantis Malesios
Jozsef Maleth
Matthew Maley
Anne-Marie Malfait
Anita Malhotra
Chetna Malhotra
Neil Malhotra
Pankaj Malhotra
M. C. Malicdan
Daniela Malide
Afshan Malik
Ashar Malik
Danish Malik
Marek Malik
Meenakshi Malik
Muhammad Malik
Rainer Malik
Shaihan Malik
Shaily Malik
Shalie Malik
Tanu Malik
Uzma Urooj Malik
Yps Malik
Bradley Malin
Steven Malin
Marjo Malinen
Daniel Maliniak
Vesna Malinova
Agnieszka Malinowska
Marcin Malinowski
Radek Malinowski
Manuel Maliqueo
Jessica Malisch
Karim Malki
Sebastian Malkusch
Nancy Malla
Francesca Mallamaci
Dirk Mallants
Jihad Mallat
Carine Malle
Jennie Mallela
Yassine Mallem
Benoit Malleret
James Mallet
Robert Mallet
Chris Malley
Tauqeer Mallhi
Luca Mallia
Prabodhika Mallikaratchy
Pavan Mallikarjun
Garladinne Mallikarjuna
Sagar Mallikethi-Reddy
Karthik Mallilankaraman
Fedirico Mallo
Jordan Mallon
Izaskun Mallona
Nuria Mallorquí-Bagué
Hanspeter Mallot
Elizabeth Mallott
Anders Malmendal
Judith Malmgren
Noah Malmstadt
Vivianne Malmström
Nilusha Malmuthuge
Bettina Malnic
Alexander Malogolovkin
Ailish Malone
Brendan Malone
David Malone
Donald Malone
Ian Malone
Jacob Malone
John Malone
Shane Malone
Shane Maloney
Heidi Maloni
Ryan Malosh
Maria Luisa Malosio
Daniel Malouli
Alina Maloyan
Norberto Malpica
Mats Målqvist
Maira Malta
Ferruccio Maltagliati
Helen Maltezou

Victor Maltsev
Marcos Malumbres
E. Malusa
João Malva
Amir Malvandi
Paolo Malvezzi
Claude Malvy
Monica Maly
Robert Malyapa
Dariya I. Malyarenko
Boris Malyugin
Adamantios Mamais
Ioannis Mamais
Maria Mamani-Matsuda
Mamas Mamas
Fahimeh Mamashli
Edson Mambuque
Piotr Mamczur
Chiara Mameli
Sujan Mamidi
Irene Mammarella
Tadanori Mammoto
Tomasz Mamos
Cyril Mamotte
Charalampos Mamoulakis
Emanuelle Mamroud
Hadii Mamudu
Zissis Mamuris
Maoqiang Man
Si Ming Man
Xiao-Yong Man
Mark Manago
Denise Manahan-Vaughan
Celia Manaia
Sudheesh Manalil
Diwas Manandhar
Mark Manary
Miriam Manary
George Manayath
Fulvia Manchado-Gobatto
Vinaya Manchaiah
Paula Manchon-Walsh
Francesca Mancianti
Giorgio Mancinelli
Susanna Mancinelli
Elena Mancini
Marcello Mancini
Maria Vittoria Mancini
Nicasio Mancini
L. Manco
Massimo Mancone
Mauro Manconi
Cesare Mancuso
Patrizia Mancuso
Malgorzata Manczak
Chandi C. Mandal
Narayan C. Mandal
Paramita Mandal
Supreet Mandala
Maurizio Mandalà
Sundhiya Mandalia
Carlos Mandarim-de-Lacerda
Antonio Mandarino
Pitchaiah Mandava
Rishi Mandavia
Jess Mandel
Michael Mandel
Jennifer Mandelbaum
Luke Mander
Vera Manders
Danilo Mandic
Vanja Mandic-Maravic
Pragati Rao Mandikal Vasuki
Pierre Mandin
Aliye Mandiracioglu
Judith Mandl
Kenneth Mandl
Giuseppe Mandolino
Mattias Mandorfer
Pietro Mandracci
Giuseppe Mandraffino
Pranoti Mandrekar
William Mandy
Chitra Mandyam
Nathan Manes
Zoi Manesi
Nicholas Maness
Yefim Manevich

Pooja Maney
Juan Mañez
Giovanni Manfredi
Piero Manfredi
Roberto Manfro
Vidya Mangala Prasad
Ashutosh Mangalam
Michael Mangan
Andrea Mangano
Kelsey Mangano
Amee Manges
Harald Mangge
Anna Lisa Mangia
Fabio Mangiacapra
Luisa Mangialajo
Brigitte Mangin
Jean-François Mangin
Gerald Mangine
Martin Mangino
Massimo Mangiola
Marco Mangone
Arduino Mangoni
Maria Luisa Mangoni
Sara Mangsbo
Neelam Mangwani
Alex Manhães
Rajeswaran Mani
Jayanti Mania-Pramanik
Alagu Manickavelu
Anne Manicone
Irena Maniecka-Bryla
P. Manikandan
Jayadev Manikkam Umakanthan
Lydia Manikonda
Deene Manikprabhu
Clement Manineng
Todd Manini
George Manis
Maria-Luisa Manis
Magna Manjareeka
Elias Manjarrez
Janna Manjelievskaia
Karim Manji
Abu Manju
S. Manjunath
Paul Manka
Justin Mankin
Kezia Manlove
Alan Mann
Brian Mann
Elizabeth Mann
John Mann
Laura Mann
Paul Mann
Ritse Mann
Robert Mann
Roger Mann
Dipak Manna
Prasenjit Manna
Adnan Mannan
Ilaria Mannazzu
Sharon Manne
Tiina Manne
Jörg Männer
Lucia Mannetti
David Manning
H. Manning
John Manning
Keefe Manning
Laurens Manning
Louise Manning
Victoria Manning
David Mannino
Peter Mannon
Braden Manns
Michael Mannstadt
Pier Mannucci
Pier Vittorio Mannucci
Dinesh Manocha
S. Manochantr
Kooroush Manochehri
Murli Manohar
Muthu Saravanan Manoharan
Laurence Manolakakis
Elias Manolakos
Charlie Manolis
Morris Manolson
Ayumi Manome

Carrie Manore
Ali Manouchehrinia
Nicholas Manoukis
Jessica Manousakis
Arjun Manrai
Angela Manrique-Alba
Betty Manrique-Espinoza
Anton Mans
C. Mans
Rachael Mansbach
Aaron Mansfield
Avril Mansfield
Elise Mansfield
Sarah Mansfield
Joanna Manson
Michael Manson
Steven Manson
Jacques Mansourati
Marjan Mansourian
Mohammad Ali Mansournia
Alf Månsson
Sven Månsson
Pascal Mansuelle
Ahmad Rois Mansur
Jean-Michel Mansuy
Angel Manteca
James Mantell
Aukje Mantel-Teeuwisse
Kalpana Manthiram
Brian Mantilla
César Mantilla
Tara Mantler
David Manton
Alessandro Mantovani
Roberto Mantovani
Konstantinos Mantsopoulos
Sarah Mantwill
Dimitra Mantzouka
Alexander Manu
Kanjoormana Manu
Manu Manu
Remy Manuel
Jennifer Manuzak
James Manwell
Antonio Manzaneda
Ann Manzardo
Enzo Manzato
Anatole Manzi
Aldo Manzin
Gianluca Manzo
Laura Manzo
Claudia Manzoni
Nikhat Manzoor
Chuanbin Mao
Gaowei Mao
Hui-Fen Mao
J. Mao
Jianjun Mao
Kezhi Mao
Limin Mao
Lingfeng Mao
Long Mao
Peisheng Mao
Weiming Mao
Wenhui Mao
Xiangbing Mao
Xu-Hu Mao
Ying-Bo Mao
Yuting Mao
Elad Maor
Rudo Mapanga
Mkhululi Maphosa
Jessica Maples-Keller
Desmond Mapps
Mark Mapstone
Salwan Maqdasy
Claudio Marabotti
Armelle Marais
Mohlopheni Marakalala
Sandro Marana
Ramona Marasco
Sayed Marashi
Sayed-Amir Marashi
M. Maraun
Kenneth Maravilla
Jose Luis Maravillas
Aniko Maraz
Francesca Marazzi

Maria Cristina Marazzi
Ketan Marballi
Aiello Marc
Ana Marçalo
Matteo Marcantonio
Rafael Marcé
François Marceau
Maurizio Marceca
Luisa Marcelino
Machteld Marcelis
La Noce Marcella
Sylvain Marcellini
Denis Marcellin-Little
Andrea Marcellusi
Marina Marcet-Houben
Axel Marchal
Andrée-Anne Marchand
Benoît Marchand
Daniel Marchant
David Marchant
Gary Marchant
Kevin Marchbank
Mercedes Marchese
Isabella Marchesi
Giulio Marchesini
Nathan Marchetti
Stefano Marchetti
Anita Marchfelder
Nicola Marchi
Régis Marchiano
Renee M. Marchin
Timoteo Marchini
Caterina Marchiò
Michele Marchioni
Davide Marchiori
Virginia Marchman
Petr Marcián
Francine Marciano-Cabral
Arkadiusz Marciniak
Stefan Marciniak
Martin Marcinkiewicz
Francisco Marco
Ignasi Marco
Francisco Marco-Jiménez
Milena Marcolino
Vanessa Aparecida Marcolino
F. K. Marcondes
Crystal Marconett
Luis Marco-Ruiz
Ingrid Marcq
Ralph Marcucio
Gonzalo Mardones
František Marec
Laëtitia Maréchal
Isabelle Marechaux
Francois Maree
Daniel Marenda
Marc Marenda
Olga Mareninova
Julie Marentette
Giancarlo Marenzi
Andrea Maresca
Marc Maresca
Vittoria Maresca
Petra Maresova
Lavinia Margarit
Nikos Margaritelis
Paris Margaritis
Claire Margerison-Zilko
Marta Margeta
Milica Margeta
Peter Margetts
Martin Margittai
William Margolin
David Margolis
Abelardo Margolles
Mark Margres
Christophe Marguet
David Margulies
Paola Margutti
Karol Marhold
Zoltan Mari
Doaa Maria
Durvanei Maria
Babu Mariadoss
Carlotta Marianecci
Francesca Mariani
Maria Mariani

Massimiliano Mariani
Patrizio Mariani
Sara Mariani
Thomas Mariani
Patrick Mariano
Vladimir Marianov
Vanitha Mariappan
Heather Mariash
Eric Marietta
Christophe Mariette
Miguel Marigil
Geneviève Marignac
Peričić Salihović Marijana
Eloi Marijon
Keith Marill
José Maria Marimon
Catherine Marimoutou
Kalisvar Marimuthu
Bóris Marin
Ethan Marin
Jose Marin
Marie-France Marin
Luis Marín
Anne Marinan
Enzo Marinari
Michela Marinelli
Iván Marín-Franch
Patrícia E. M. Melo Marinho
Thatiany Marinho
Andrea Marini
Carla Marini
Elisabetta Marini
Francesco Marini
Frank Marini
Giovanni Marini
Serge P. Marinkovic
Rodrigo Marín-Navarrete
Bruno Marino
Nicholas Marino
Antonella Marino Gammazza
Atanaska Marinova-Petkova
Welber Marinovic
Luis Marins
Marta Mariotti
Raffaella Mariotti
Paolo Mariottini
Christine Maritz-Olivier
Stefan Marius
Lilian Mariutti
Arthur Marivin
Alan E. Mark
Bryan Mark
Christie Mark
Jonathan Mark
Kristen Mark
Patrick B. Mark
Anil Markandya
Doug Markant
Julie Markant
Ana Markelz
Sebastian Markett
Kate Markey
Maria Markiewicz-Keszycka
Niina Markkula
Francis Markland
Melissa Markofski
Maria Markoulli
Gabriel Markov
Mariya Markova
Dimitrije Markovic
Nikola Markovic
Svetomir Markovic
Amanda Markovitz
Ezra Markowitz
Georgios Markozannes
Angharad Marks
Bonita Marks
Donald Marks
Haley Marks
Michael Marks
David Markusic
Thomas Markussen
Michal Markuszewski
Philipp Markwerth
Ferdinand Marlétaz
Gifty Marley
Laura Marlow
Srinivas Marmamula

Vivien Marmelat
Marta Marmiroli
Nelson Marmiroli
Sandra Marmiroli
Fernando Marmolejo-Ramos
Naomi Marmorstein
Michael J. Marmura
Alexander Marneros
Jeanine Marnewick
Hanna Marno
Cassandra Marnocha
Adriano Marocco
Tal Marom
Daniela Marone
J. Marostica
Luc Maroteaux
Hubert Marotte
Juliana Marotti
Richard Marottoli
Leonard Maroun
David Marpaung
Nicola Marples
Quentin Marquant
Nicole Marquardt
A. Marques
Adilson Marques
Alexandre Marques
C. Marques
Elisa Marques
Fabio Marques
Joaquim Marques
Marcia Marques
Mário Marques
Mathew Marques
Miriam Marques
Luis Marques Dos Santos
Pierre Marquet
Roland Marquet
Manlio Márquez
Johann Marquez-Barja
Hélène Marquis
Nico Marr
Carlo Marra
Christina Marra
Fawziah Marra
Francesco Marra
Paolo Marra
Roberta Marra
Camillo Marra Chiara
Vannina Marrachelli
Philippa Marrack
Luciano Marraffini
Hedia Marrakchi
Annarita Marrano
Jeanne Marrazzo
Helia Marreiros
Daniele Marrelli
Mauro Marrelli
M. Marrese
J. Marron
Giusi Marrone
Kristen Marrone
Oreste Marrone
Fabio Marroni
Norma Marroni
Laura Marroquí
Hélène Marrou
James Marrs
M. Mars
Martin Marsala
Petr Marsalek
Peter Marschang
Alison Marsden
Anthony Marsh
Elisabeth Marsh
Eric Marsh
Heidi Marsh
Helene Marsh
James Marsh
John Marsh
Kerry Marsh
Phil Marsh
Samantha Marsh
Samuel Marsh
Terence Marsh
Tim Marsh
Aaron Marshall
Andrew Marshall

Ian Marshall
Jennifer Marshall
Karen Marshall
Katie Marshall
Lynn Marshall
Maria Marshall
Michael Marshall
Nathaniel Marshall
Paul Marshall
Tara Marshall
Zoe Marshman
Letizia Marsili
Benjamin Marsland
Frederic Marsolais
Lukas Eugen Marsoner
Steinkasserer
Amelie Marsot
Denise Marston
John Marston
Kieran Marston
Carlos Marta
Marco Martalo
Enzo Martegani
An Martel
Jan Martel
Sebastian Martel
Joseph Martel-Foley
Vito Martella
Paolo Martelletti
Francesca Martelli
Giovanna Martelli
Marialuisa Martelli
Meredith G. Marten
John Martens
Martin Martens
Sander Martens
Hans-Peter Marti
Joachim Marti
Joan Marti
Josep Marti
Matthias Marti
Maria Jose Martí
Mercè Martí
Ricardo Marti-Arbona
Cristian Martignani
Luis Martim
Alastair Martin
Alberto Jesus Martin
Alberto Martin
Allan Martin
Andrew Martin
Anne-Celine Martin
Ben Martin
Benjamin Martin
Bernard Martin
Carlos Martin
Chris Martin
Christian Martin
Christina Martin
Claire Martin
Clara Martin
Craig Martin
Debra Martin
Elizabeth Martin
Erika Martin
Estelle Martin
Fabiola Martin
Franck Martin
Graeme Martin
Jack Martin
James Martin
Janet Martin
Javier Martin
Jérémy Martin
Jessica Martin
Jodi Martin
Joseph Martin
Juan Martin
Karen Martin
Kathleen Martin
Kendall Martin
Laura Martin
Lee Martin
Lisa Martin
Loren Martin
Lorna Martin
Luci Martin
Miguel Martin

Natalia Martin
Oliver Martin
Randi Martin
Richard Martin
Samuel Martin
Sandra Martin
Scott Martin
Seth Martin
Simone Martin
Stephanie Martin
Tammy Martin
Thomas Martin
Wayne Martin
Margarita Martín
Rebeca Martín
Verónica Martín
Kathleen Martin Ginis
Maria Teresa Martin Gomez
Adriane Martin Hilber
Ángela Martín Jiménez
Sofia Martin Suarez
Luigi Martina
Valeria Martin-Albarracin
Sanda Martincic-Ipsic
Lluís Martin-Closas
Noa Martin-Cofreces
Robert Martindale
Tim Martineau
Elena Martinelli
Ombretta Martinelli
Paola Martinelli
Vittorio Martinelli
Marianne Martinello
Nadine Martinet
Alfredo Martinez
Andres Martinez
Andy Martinez
Ari Martinez
Arturo Martinez
Esteban Martinez
Henry Martinez
Jessica Martinez
Julien Martinez
Leandro Martinez
Leonardo Martinez
Luis Martinez
M. Luisa Martinez
Manuel Martinez
Maria Carmen Martinez
Miguel Martinez
Omar Martinez
Pedro Martinez
Agustín Martínez
Ana Martínez
Luis Carlos Martínez
María Martínez
Miguel Angel Martínez
Sergio Martínez
Alejandra Martinez Berdeja
Alejandro Martinez Bueno
Moisés Martínez Velázquez
Claudia Martínez-Anaya
Beatriz Martínez-Azúa
M. Martínez-Calderon
Jimmy Martínez-Correa
Josué Martínez-de la Puente
Ana Martinez-Donate
Flor Martinez-Espinosa
Vanessa Martínez-Fernández
Daniel Martinez-Fong
Fernando Martinez-Garcia
Marina Martinez-Garcia
Blanca Martinez-Garriga
Luis Martinez-Gil
Pedro Martínez-Gómez
Antonio Martínez-Graña
José Armando Martínez-Guarneros
Jose-Luis Martinez-Guitarte
Sergio Martinez-Herrera
David Martínez-Íñigo
Antonio Martínez-Laborda
Maria Martinez-Larrad
Pablo Martinez-Legazpi
Luis Martinez-Lemus
Emilio Martínez-López
Pilar Martinez-Martinez
Ismael Martínez-Martínez

Karina Martínez-Mayorga
Ignacio J. Martinez-Moyano
Juan Martínez-Murillo
J. Martinez-Padilla
Marcel Martinez-Porchas
Javier Martínez-Reina
María Angeles Martinez-Rivera
M. D. Martínez-Rodrigo
Alejandro Martinez-Rodriguez
Carolina Martínez-Ruiz
Luis Martínez-Sobrido
Ataulfo Martínez-Torres
M. Martínez-Trujillo
Javier Martinez-Useros
Eduardo Martinez-Valdes
Manuel Martin-Flores
Irene Martín-Forés
Raquel Martín-Hernández
Ana Carolina Martini
Enrico Martini
Katharina Martini
Matteo Martini
Rino Martini
Ludmila Martínková
Alejandro Martin-Montalvo
Carlos Martino
Davide Martino
Elena Martino
Giovanni Martino
Maria Elena Martino
Sabata Martino
Tami Martino
Kimberly Martinod
Jean Benoît Martinot
Giovanni Martinotti
Carmen Martin-Ruiz
Ademir Martins
André Martins
Bruno Martins
Catarina Martins
Cesar Martins
Cyro Martins
Elisabete Martins
Elizabeth Martins
Fabiane Martins
Fernandaalves Martins
Gustavo Martins
José Luiz Martins
Leonardo Martins
Luis Martins
Regina Martins
Renato Martins
Ricardo Martins
Roberta Martins
Samuel Martins
Tiago Martins
Viviana Martins
Wellington Martins
Yuri Martins
Ana Martins Sequeira
Egil Martinsen
Francisco Rogerlândio Martins-Melo
Jann Martinsohn
Jeremy Martinson
Sebastian Martinuzzi
Jose-Luis Martin-Ventura
Lynn Martire
Tamas Marton
Domenico Martone
Zsolt Mártonfalvi
Francesca Martora
Lourdes Martorell
Miquel Martorell
Reynaldo Martorell
Katherine Martucci
Charles Marty
Robert Marty
C. Martyniuk
Anatoly Martynyuk
Anthony Martyr
Shruti Kochar Maru
Shigeru Marubashi
Paul Maruff
Takeshi Marumo
James Maruniak
Antonello Maruotti
Maija Marushchak

Petr Marusic
Ana Marušić
Jun Maruta
Shinsaku Maruta
Haruhiko Maruyama
Jun-Ichi Maruyama
Keisuke Maruyama
Shoichi Maruyama
Toru Maruyama
Yukio Maruyama
Mohsen Marvibaigi
Juan Carlos Marvizon
Andreas Marx
Brian Marx
Florian Marx
Hannah Marx
Philip Marx Stoelting
Um Maryse
Manja Marz
Caren Marzban
Lucy Marzban
S. Marze
Alex Marzel
Emanuele Marzetti
Laura Marzetti
Jan Marzinek
Ezequiel Marzinelli
Martin Pierre Marzloff
Vincenzo Marzolla
Massimo Marzorati
George Marzouka
Paolo Marzullo
Michael Mäs
Oku Masahide
Johnson Masaka
Giovanni Batista Masala
Kazuto Masamoto
Kei Masani
Mario Masarone
Masuda Masashi
Engin Masazade
Laurent Mascarell
Sandra Mascarenhas
Françoise Mascart
Giuseppe Mascia
Luca Mascitelli
Masaji Mase
Michela Masè
Mario Masellis
Bernd Masepohl
Omar Masera
Rosalinde Masereeuw
Biancaelena Maserti
Frank Masese
Linnet Masese
Matteo Masetti
Aikaterini Masgala
Khathutshelo Mashige
George Mashour
Saidur Mashreky
Alessio Masi
Anne Masi
Antonio Masi
Marco Masi
Lorenzo Masia
Simona Masiero
Madhan Masilamani
Daniel Masison
Felix Masiye
Juan Masjoan
Shiraz Maskatia
Nick Maskell
Brian Maskery
Cheryl Maslen
Edward Masler
Sharmila Masli
Dmitri Maslov
Gary Maslow
Isabelle Masneuf
Cristina Masoller
Alexina Mason
Dyana P. Mason
Hugh Mason
Jeffrey Mason
Linda Mason
Nicole Mason
Paul Mason
Peggy Mason

Ralph Mason
Suzanne M. Mason
William Mason
Winter Mason
Mohamed Masoud
Samar Masoumi-Moghaddam
Bruno Masquelier
Helen Massa
Stephen Massa
Diana Massai
A. Massaia
Marta Massanella
Rodrigo Massara
Judy Massare
Paola Massari
Emanuele Massaro
Carlo Massaroni
Suleiman Alfred Massarweh
Jana Masselink
Leah Masselink
Nurulkamal Masseran
Michael Massett
Peter Massey
Ruth Massey
Daniel Massicotte
Lourdes Massieu
Chandirasegaran Massilamany
Guerda Massillon J. L.
Dario Massimino
Pierre Massion
Alessandro Massolo
Jean-Baptiste Masson
Serge Masson
Sebastien Massoni
Ivana Massud
Sarabjit Mastana
Simon Mastbergen
Forrest Masters
William Masters
Grant Mastick
Aikaterini Mastoraki
Francesca Mastorci
Sharon Mastracci
Lucy Mastrandrea
Simona Mastrangeli
Salvatore Mastrangelo
Arianna Mastrofrancesco
Claudio Mastroianni
Patricia Mastroianni
Salvatore Andrea Mastrolia
Hirotaka Masuda
Shinobu Masuda
Gwinyai Masukume
Kenji Masumoto
Howard Masuoka
Tumishang Maswime
Nor Ashidi Mat Isa
Ignacio Mata
Rui Mata
David Mataix-Cols
David Matallanas-Gomez
Anna Matamala
Joan Matamalas
Miriam Matamales
Oranart Matangkasombut
Tetsuro Matano
Marios Mataragas
Valerie Matarese
Bashir Matata
Farai Matawa
David Matchar
Gr Matcuk
Victor Mateevitsi
José Luis Maté-Muñoz
Ana Mateos
Magdalena Materniak-Kornas
Silvia Matesanz
Guillermo Mateu
Marcos Mateus
Matthews Mathai
Michael Mathai
Prince Mathai
Susan Mathai
Domokos Máthé
Hugo Mathé-Hubert
Anthony Mathelier
Barun Mathema
Ashley Matheny

Alison Mather
Jennifer Mather
Kieren Mather
Jonathan Mathers
Kate Mathers
Flora Matheson
Megan Matheson
Anuja Mathew
Linu Mathew
Jeffrey Mathews
Michael Mathews
Shanaaz Mathews
Klaus Mathiak
Clinton Mathias
Candace Mathiason
Alistair Mathie
Elspeth Mathie
Joachim Mathiesen
William Mathieson
Patrick Mathieu
Erik Mathijs
Alexandros Mathioudakis
Roger Mathisen
Bertrand Mathon
Sebastiaan Mathôt
Sameer Mathur
Vanessa Mathys
Margarida Matias
Alexis Matikas
Miguel Angel Matilla
A. Matin
Hanna-Maria Matinolli
Janos Matko
Jordan Matley
Nobuyuki Matoba
Satoaki Matoba
Dinka Matošević
Natalie Matosin
Andres Matoso
Joseph Matovu
Takashi Matozaki
Khalid Matrougui
Michael Matschiner
Franz M. Matschinsky
Gladys Matseke
Tandi Matsha
Hidenori Matsubara
Kazuki Matsubara
Keiichi Matsubara
Nagahide Matsubara
Shigeki Matsubara
Takeshi Matsubara
Fumio Matsuda
Hiroshi Matsuda
Junko Matsuda
Kazuyuki Matsuda
Koichi Matsuda
Masaru Matsuda
Naoyuki Matsuda
Shinichi Matsuda
Shinji Matsuda
T. Matsuda
Yasuyuki Matsuda
Ko Matsudaira
Yuya Matsue
Tetsuya Matsuguchi
Kazuaki Matsui
Mari Matsui
Reiko Matsui
Yoshizo Matsuka
Chiaki Matsukura
K. Matsukura
Wataru Matsumiya
Chisa Matsumoto
Haru Matsumoto
Hiroyuki Matsumoto
Joseph Matsumoto
Kazumasa Matsumoto
Masaki Matsumoto
Naoko Matsumoto
Osamu Matsumoto
Ryusaku Matsumoto
Noboru Matsumura
Shigenobu Matsumura
Tsuyoshi Matsumura
Yasufumi Matsumura
Hisato Matsunaga
James Matsunaga

Masahiro Matsunaga
Hiroaki Matsuo
Koji Matsuo
Masafumi Matsuo
Ryu Matsuo
Yukinori Matsuo
Junji Matsuoka
Masaaki Matsuoka
Satoshi Matsuoka
Taiji Matsusaka
Toshinori Matsushige
Akihiro Matsuura
Cristiane Matsuura
Eiji Matsuura
Isao Matsuura
Keita Matsuura
Motokazu Matsuura
Tohru Matsuura
Yu Matsuura
Hideyasu Matsuyama
Ryota Matsuyama
Shigemi Matsuyama
Takatoshi Matsuyama
Tasuku Matsuyama
Yusuke Matsuyama
Jaime Matta
Mario Matta
Antonella Mattana
Alberto Matteelli
Josiemer Mattei
Giorgi-Pierfranceschi Matteo
Magali Matteodo
Gianluca Matteoli
Michelle Matter
Thomas Mattern
Gail Matters
William Mattes
Mario Matthaei
Franziska Matthaus
Harry Matthews
James Matthews
Judy Matthews
Luke Matthews
Lynn Matthews
Lynsay Matthews
Tony Matthews
Stephen Matthey
Schiemann Matthias
Swantje Matthies
Maurizio Mattia
Silvana Mattiello
Joshua Mattila
Olli Mattila
William Mattingly
Emanuela Mattioli
Simona Mattioli
Stefano Mattioli
Monica Mattioli-Belmonte
Julie Mattiske
Christopher Mattison
Jochen Mattner
Calum Mattocks
Seema Mattoo
Claudia Mattos
Robert Mattrey
Mark Mattson
Johanna Mattsson
Radim Matula
Daumantas Matulis
Michael Matunis
David Matus
Francisco Matus
Radek Matušů
Zenon Matuszak
Szymon Matuszewski
Carlos Matute
Karl Matz
Christina Matz-Costa
Maor Matzrafi
Felix Mauch
Carmelo Maucieri
Eric Mauerhofer
Andrea Maugeri
Jeff Maughan
Aaron Maule
Maximilian Mauler
Nilanjana Maulik
Subir Maulik

Robert Maunder
Steffen Maune
Pierre Maurage
Cedric Maurange
Joan Maurel
Andrew Maurer
Tobias Maurer
Mauro Mauri
Pierluigi Mauri
Roberto Mauri
Fadel Maurice
Madelon Maurice
Dídac Mauricio
Thomas Maurin
Natasha Maurmann
Wendy Maury
Ramanuj Maurya
Josephine Mauskopf
Fabienne Mauxion
Ernest William Mauya
Barbara Mauz
Vidya Mave
Fabio Mavelli
Uroš Maver
Laurent Maveyraud
Aigul Mavletova
Clio Mavragani
Georgia Mavria
Demetra Mavri-Damelin
Konstantinos Mavridis
Eleni Mavrogonatou
Ludo Max
Maxime Maxime Tarabichi
Nigel Maxted
Anthony Maxwell
Carl May
Clive May
Jody May
Margaret May
Philip May
Susanne May
Daisy May Constantino-Santos
Juan Maya
Maria Mayan
Shekhar Mayanil
Yunia Mayanja
Simnikiwe Mayaphi
Oleg Mayboroda
Alexander Maye
Manasi Mayekar
Audrey Mayer
Brooke Mayer
Deborah Mayer
Erik Mayer
Larry Mayer
Leonard Mayer
Lukas Mayer
Marius Mayer
Michael Mayer
Uwe Mayer
Katrin Mayer-Barber
Marius Mayerhoefer
David Mayerich
Mariana Mayer-Pinto
Patrick Mayeux
Anderson Mayfield
Dean Mayfield
R. Mayfield
Christopher Mayhorn
Erin Maynard
Jennifer Maynard
Jonathan Maynard
Olivia Maynard
Steven Maynard-Moody
Benjamin Mayne
Darren Mayne
Richard Mayne
Charles Mayo
Christie Mayo
Leah Mayo
Lindsey Mayo
Karla Mayolo-Deloisa
Thibault Mayor
Chrispus Mayora
Gisela Mayora
Marcus Mayorga
Evan Mayo-Wilson
Eva Mayr

Gerald Mayr
Patrick Mayr
Philipp Mayr
Winfried Mayr
Helen May-Simera
Holger Mayta
Edward Maytin
Nishi Mayumi
Elie Maza
Shali Mazaki-Tovi
Sylvie Mazan
Antonios Mazaris
Séverine Mazaud-Guittot
Mervyn Maze
Stephanie Mazerolle
Jonna Mazet
Noemí Mazía
Julien Mazières
Stéphane Mazières
Sarkis Mazmanian
Manuel Mazo
Tessa Mazor
Yariv Mazor
Papiya Mazumdar
Allan Mazur
Marcelina Mazur
Wojciech Mazur
David Mazurais
Agnieszka Mazur-Bialy
Nathalie Mazure
Alexey Mazus
Barbara Mazza
Paulo Mazzafera
Sandro Mazzaferro
Enzo Mazzaro
Andrea Mazzatenta
Pierluigi Mazzei
Maria Mazzitelli
Antonio Mazzocca
Fulvio Mazzocchi
Mario Mazzocchi
Michele Mazzola
Paolo Mazzola
Carlotta Mazzoldi
Maurizio Mazzoleni
Emanuela Mazzon
Andrea Mazzone
Emanuele Mazzoni
Cosimo Mazzotta
Diego Mazzotti
Frank Mazzotti
Sara Mazzucco
Ikechukwu Innocent Mbachu
Yamikani Mbilizi
Justice Mbizo
Anthony Mbonye
Gerald Mboowa
Sam Mbulaiteye
Lazarous Mbulo
Gitau Mburu
Lars Mc Naughton
Scott McAdam
David McAdams
Tom McAdams
S. McAdoo
Luke McAlary
Jan McAllister
Lisa McAllister
Scott McArt
J. McArthur
Julie McAuley
Kimberley McAuley
Priscilla McAuliffe
Heather McAuslane
Alan McBride
Anne McBride
Joseph McBride
Kevin McBride
Orla McBride
Richard McBride
Shonna McBride
William McBride
Kerry McBrien
Emma McBryde
Christopher McCabe
Kira McCabe
Laura McCabe
Mark McCabe

Ryan McCabe
Jeanne McCaffery
Peter McCaffery
Luke Mccaffrey
Anthony McCall
Cade McCall
Chad McCall
Grant McCall
Jamie McCall
Matthew McCall
Maureen McCall
Scott McCallum
Timothy McCalmont
Alana McCambridge
Dennis McCance
Honour McCann
Joshua McCann
Stewart McCann
Meg McCarron
D. P. McCartan
Antonio Desmond McCarthy
Avril D. McCarthy
Cathal McCarthy
Colin McCarthy
Elizabeth McCarthy
John McCarthy
Mary McCarthy
Matthew McCarthy
Neil McCarthy
Noel McCarthy
Ona McCarthy
Peter McCarthy
Curt McCartney
Christopher McCarty
Dennis McCarty
Douglas McCarty
Eric McCarty
Kristofer McCarty
Owen McCarty
Stacy McCarty
Michael Alexander McCaskey
Jennifer McCaughan
Mary McCauley
Affette McCaw-Binns
Penn McClatchey
Dan McClatchy
Linda McClean
William McCleary
Gregory McClelland
R. Scott McClelland
Robyn McClelland
Beverly Mcclenaghan
Tim McClintock
Karen McCloskey
Christopher J. W. McClure
Elizabeth McClure
Kirk McClure
Sarah McClure
Matt McCluskey
Stuart McCluskey
Suzanne McCluskey
Sue McCoard
Mark McComb
Pamela McCombe
Jonathan McConathy
Kim McConkey
Eleanor McConnell
Gail McConnell
Michael McConnell
Michelle McConnell
Patrick A. McConnell
C. McConville
Abby McCormack
Erin McCormack
M. Luke McCormack
Meredith McCormack
Joy McCorriston
Mark McCourt
Matthew McCoy
Sandra McCoy
J. Philip McCoy Jr.
Shawn McCracken
Vance McCracken
Malcolm McCrae
Nicky McCreesh
Rory McCrimmon
Jean McCrory
Andrew G. McCubbin

Marshall McCue
Charlotte McCullagh
Karl McCullagh
Paul McCullagh
Christopher Ag McCulloch
Michael McCulloch
Jeffrey McCullough
Mark Mccullough
Matthew McCullough
Megan McCullough
Robert McCullumsmith
Bruce McCune
Matthew McCurry
Christine McCusker
J. McCutchan
Robert McCutcheon
Vivia McCutcheon
Thomas McDade
Lisa McDaid
Tara McDaneld
Kelly McDaniel
Marshall McDaniel
Michael McDaniel
Troy McDaniel
Michael McDermott
Paul McDermott
Rose McDermott
Ross McDevitt
Tracey McDole
Birgitte McDonald
Christine McDonald
Daniel McDonald
Elizabeth L. McDonald
Ellie McDonald
Emily McDonald
Fiona McDonald
James McDonald
Jay McDonald
Laura McDonald
M. McDonald
Michael McDonald
Michelle McDonald
Scott McDonald
Liam McDonnell
P. McDonnell
Timothy McDonnell
Alicia McDonough
Ian McDonough
Suzanne McDonough
Carmel McDougall
David McDowall
Andy McDowell
Colleen McDowell
Gary McDowell
Michael McEachern
Ken McElreavey
Kenneth Mcelreavey
Anita McElroy
Noel McElvaney
John McEvoy
Brad McEwen
Scott McEwen
Brandon McFadden
Bradford J. McFadyen
J. Ricardo McFaline-Figueroa
Braden McFarland
Daniel McFarland
David McFarland
Karen McFarland
Malcolm McFarland
Nikolaus McFarland
Richard McFarland
Wayne McFee
Quinn McFrederick
Patrick McGah
Forbes McGain
Patrick McGann
Kevin McGarigal
Anthony McGarry
Conor McGarry
Arlene McGarty
Craig McGarty
Stephen McGarvey
Suzanne McGaugh
Kerry McGawley
Michael McGeachie
Corey McGee
Lesley McGee

Monnie McGee
Richard G. McGee
Meghan McGee-Lawrence
Paul McGeoch
William Mcgeown
James McGettigan
David McGhie
Milton McGiffen
Fiona McGill
Janet McGill
Jodi McGill
Ed McGinley
James McGinty
David McGivern
Gerry McGivern
C. Jane McGlade
Elise McGlashan
Daniel McGlinn
Matthew McGlone
Chris McGlory
Andrew McGovern
Mark McGovern
Thomas Mcgovern
C. M. McGowan
Courtney McGowan
Eileen McGowan
Jennifer McGowan
Laura McGowan
Maura McGrail
Andrew McGrath
Catherine McGrath
Margaret McGrath
Patrick McGrath
Hillary McGraw
Kathy McGraw
Rose McGready
Ailsa McGregor
Alison McGregor
Keith McGregor
Morgan McGregor
Mike McGrew
Anita McGrogan
Michael McGuckin
Rankin McGugin
Nicola McGuigan
William McGuiness
Owen McGuinness
Betty McGuire
Mark McGuire
William McGuire
Kenneth McGwire
James McHale
Amy McHenry
Matt McHenry
Andrew McHill
Joe McHugh
John McHugh
Elizabeth McHuron
Peter McIlveen
Charlton McIlwain
Stephen McIninch
Matthew McInnes
Opal McInnis
Kristina McIntire
Anthony McIntosh
J. Richard McIntosh
Kenneth McIntosh
Rebecca McIntosh
Stuart McIntosh
Jeremy McIntyre
Trevor McIntyre
Robert McKallip
Stuart McKamey
Dana McKay
Derek McKay
Fiona McKay
Kyla Mckay
Michael McKay
Andrew McKechanie
Kimberly McKee
Neil McKeganey
Duane McKenna
Gerald McKenna
James McKenna
Leanda McKenna
Megan Mckenna
Peter McKenna
Jennie McKenney

Cynthia McKenzie
Lara McKenzie
Marian McKenzie
Andrew McKeon
Charlotte McKercher
Luseadra McKerracher
Doyle McKey
Michael McKie
Shane Mckie
Aine McKillop
Caitlin McKillop
Daniel McKim
Blake McKimmie
Chris McKinlay
Danette McKinley
Sophia K. McKinley
James McKinnell
Michael McKinney
Richard McKinney
A. McKinnon
Britt McKinnon
John McKinnon
Lyle McKinnon
Kai McKinstry
Andrew Mckinstry-Wu
J. Chris McKnight
Thomas D. McKnight
Andrew McLachlan
Robert McLachlan
Sandra McLachlan
Alexander McLain
Jean McLain
Molly McLaren
Neil McLatchie
BethAnn Mclaughlin
Jacqueline McLaughlin
Margaret McLaughlin
Milena McLaughlin
Patricia McLaughlin
Patrick McLaughlin
Robert McLaughlin
Susan McLaughlin
Margaret McLaughlin-Drubin
Toni McLaurin
Anna McLean
Carmen McLean
Estelle McLean
G. McLean
Gary McLean
Robert McLean
Scott McLean
Mark McLelland
Eleanor McLellan-Lemal
Monica McLemore
Andrew McLennan
Conor McLennan
Damian McLeod
Graem McLeod
Ian McLeod
Julie McLeod
Kyle McLeod
Lynette J. McLeod
Linda McLoon
Caleb McMahan
Chris McMahan
James McMahon
Meagan McMahon
Robert McMahon
Roisin McMahon
James McManaman
Ryan McManamay
George McManus
Joel McManus
Conor McMeniman
Donald McMillan
Matthew Mcmillan
Matthew McMillin
Troy McMullin
Helene McMurray
Timothy L. McMurry
James McNair
Margaret McNairy
L. McNamara
Mairéad McNamara
Niamh McNamara
Robert McNamara
James McNamee
M. A. McNarry

Monica McNeal
Clea McNeely
Marie McNeely
Daniel McNeil
Eileen McNeill
Bairbre McNicholas
Paul McNicholas
Gavin McNicol
Louise-Anne McNutt
John McPeak
Michael McPhaden
Jamie S. McPhee
Bradley McPherson
David McPherson
Laura McPherson
Nicole Mcpherson
Robert McPherson
Lauren McPhillips
Amy McQueen
Jim McRoberts
Ronald McRoberts
Blakeley McShane
Heather McShane
Michael McShane
Daniel McSkimming
Brian McStay
Alistair McTaggart
F. McVerry
David McVey
Sean McWhinney
David McWilliams
Gabriela Meade
Kieran Meade
Philip Meade
Aimee Meader
Amanda Meadows
Richard Meadows
Rebecca Meagher
Robert Meagher
Judith Meakin
Lee Meakin
Calvin Meaney
David Meaney
Peter Meaney
Sarah Meaney
Anna Means
Arianna Means
Robert Means
Daniel Meara
Ellen Meara
Vivien Measday
G. Measey
Jared Mecha
Raphael Mechoulam
Karen Meckstroth
Mauro Mecozzi
Cristina Mecucci
Annamaria Mecuri
Carlinda Medeiros
Carlos Medeiros
Susanna Medellin-Garibay
Jan Medenbach
Roger Meder
Walaa Medhat
Antonio Medina
Jared Medina
Oula Medina
Rafael Medina
Enrique Medina-Acosta
María Del Pino Medina-Brito
Jose Medina-Inojosa
Carlos Medina-Torres
Nicholas Medland
Kathryn Medler
Steven Medley
Stephanie Medlock
Belinda Medlyn
Matus Medo
Marvin Medow
Hipólito Medrano
Sean Meehan
Paul Meek
Mark Meekan
Krai Meemon
Nagoor Meeran
Mariana Meerhoff
Geert Meermans
Annegret Meermeier

Kevin Meesters
Ivan Meeus
Delphine Meffre
Gholam Hossein Meftahi
Kesav Meganathan
Lynn Megeney
Sara Meger
Bindiya Meggi
Itamar Megiddo
Francis Mégraud
Timothy Megraw
Muthamilarasan Mehanathan
Saurabh Mehandru
Céline Méhats
Riffat Mehboob
Bano Mehdi
Seyed Mehdi Nouraie
Mahboobeh Mehdikhani
Elod Mehes
Mohamed Mehiri
Alexander Mehler
Camilla Mehlum
Obaid Mehmood
Abha Mehndiratta
Sarika Mehra
Adib Mehrabi
Masomeh Mehrabi
Vikram Mehraj
Ali Mehri
R. Mehrotra
Omid Mehrpour
Anahita Mehta
Atul Mehta
Dolly Mehta
Gautam Mehta
Geeta Mehta
Hemal Mehta
Jawahar Mehta
Minesh Mehta
Mitul Mehta
Neil Mehta
Priyanka Mehta
Ravindra Mehta
Sanjay Mehta
Satish Mehta
Shwetal Mehta
Stuti Mehta
Supriya Mehta
Han-Wei Mei
Jiaqin Mei
Jie Mei
W. Mei
Wenbin Mei
Xiangdong Mei
Yi Mei
Yiwen Mei
Andreas Meid
Beat Meier
Christoph Meier
Jeffery Meier
Thomas Meier
Anton Meijer
Karina Meijer
William Meikle
Dan Meila
Richard Meilan
Leo Meile
Richard Meindl
Felix Meinel
Rick Meinersmann
Natalia Meir
Lindolfoda Silva Meirelles
Pedro Meirelles
Noam Meiri
Susan Meiring
Yohai Meiron
Jill Meirte
Jacques Meis
Susanne Meis
Peter Meisel
Richard Meisel
Allison Meisner
Wassilios Meissner
Ramona Meister
Marina Meixner
Amanda Mejia
E. C. Mejia
Juan Manuel Mejia

Javier Mejía Cubillos
Raquel Mejías-Luque
Said Mekary
Elisa D. Mekler
Wubegzier Mekonnen
Giuseppe Melacini
John Melack
Haddish Melakeberhan
Oren Melamud
Marites Melancon
Philippe A. Melas
Roxanne Melbourne-Chambers
Roberto Melcangi
Ted Melcer
Thomas Melchardt
Karsten Melcher
Klaus Melchers
Ine Melckenbeeck
Marcello Mele
Maria Laura Mele
Paula Meleady
Alessia Melegaro
G. J. Melendez-Torres
Jorge Melendez-Zajgla
David Melero-Fuentes
César Melgar
Hassan Melhem
Nadine Melhem
Rosa Marina Melillo
Pierrette Melin
Alicia Melis
Giulia Melis
Michelle Melisko
C. Daniel Meliza
Isabelle Melki
Mulugeta Melku
Joshua Mell
Loren Mell
Jaime Mella
Mario Mellado
Melha Mellata
Lene Mellemkjær
Gerrit Melles
Emmanuel Mellet
Mehmet Melli
Valerie Melli
Alexander Mellmann
Luane Marques Mello
Marco Mello
Maria Luiza Mello
Michelle Mello
Lisa Mellon
Amine Mellouk
Dorra Mellouli
Yuliya Michailova Melnichenko
Nataliya Melnikova
Ana Melo
Bruno F. Melo
Eduardo Melo
Felipe Melo
Gustavo Melo
Hygor Piaget Melo
Lauro Melo
Manuel Melo
Nathalie Melo
Patricia Melo
Paulo Melo
José Melo-Ferreira
Gabriella Meloghisi
Joanna Melonek
Antonella Meloni
Bruno Meloni
Gabriele Meloni
Sandro Meloni
Luca Melotti
Peppa Melpomeni
Rebecca J. Melrose
Catharina M. C. Mels
Ambrose Melson
Laleh Melstrom
Phillip Melton
James Melton III
Angela Melton-Celsa
James Meltzer
Jed Meltzer
Kari Melve
Stephen Melville
Ryan Melvin

Ann Melzer
Rainer Melzer
Amir-Hossein Memari
Sanaz Memarzadeh
Mohamed-Mahmoud Memmah
Muhammed Memon
Qiulei Men
Nacho Mena
Didier Mena Aguilar
Ciro Menale
Jasmine Menant
Didier Menard
A. Menchaca
Cathy Mendelsohn
Robert Mendelsohn
Emily Mendenhall
Adelio Mendes
Fausto Mendes
Joana Mendes
João Mendes
Lucas William Mendes
Pedro Mendes
Renato Mendes
Armando Mendez
Carmen Mendez
Nina Mendez
Pedro Mendez
Christopher Mendias
Jessica Mendieta-Wejebe
Jaime Mendiola
Carlos Mendivil
Janine Mendola
Jorge Mendoza
Pilar Mendoza
Oliver Mendoza-Cano
Alexis Mendoza-León
Clara Mendoza-Lera
Victor Manuel Mendoza-Nuñez
German Mendoza-Silva
Rachel Menegaz
Chiara Meneghetti
Marco Meneguz
J. Carlos Menéndez
Margarita Menéndez
Manuel Menéndez González
Luis Menendez-Arias
Carlos Menendez-Castro
Pedro Menezes
Ritesh Menezes
Telmo Menezes
Rafael Menezes-Reis
Fanming Meng
Fanyin Meng
Guozhu Meng
Han Meng
Hao Meng
He Meng
Jianjun Meng
Jihua Meng
Kangpei Meng
Linghui Meng
Maxwell Meng
Ming Meng
Qingfeng Meng
Qinggang Meng
Qing-Jun Meng
Shu Meng
Songdong Meng
Weiyi Meng
Weizhi Meng
Xiang-Chen Meng
Xianghui Meng
Xianxin Meng
Xuhui Meng
Ying Meng
Yuan Meng
Zhao-Fu Meng
Zhaowei Meng
Zhiqiang Meng
Zibo Meng
Jonas Mengel-From
Catherine Mengelle
F. M. Menger
Alemayehu Mengesha
Hylemariam Mihiretie Mengist
Alessio Mengoni
Marlene Mengoni
Trevelyan Menheniott

Francesco Menichetti
Stefano Menichetti
Douglas Menke
Luiz Menna-Barreto
A. Mennander
Franck Mennechet
Anant Menon
Bijoy Menon
Jairam Menon
Manoj Menon
Ram Menon
Sonia Menon
Swapna Menon
Vijay Menon
Vikas Menon
David Menotti
Fiona Mensah
Jacob Mensah-Attipoe
Julian Mensch
Armand Mensen
Allen Mensinger
Gregorio Mentaberre
Sibel Mentesse
Stef Menting
Andreas F. Mentis
Dimitrios Mentis
Giovanni Mento
Karen Menuz
Timothy Menza
Claudia Menzaghi
Bjoern H. Menze
Kibret Mequanint
Serena Meraviglia
Nofel Merbahi
Lucile Mercadal
Sebastiano Mercadante
Diego J. Mercanti
Paolo Mercantini
Alison Mercer
Catherine Mercer
George Mercer
Jason Mercer
Paul Mercer
Stewart Mercer
Gildas Merceron
Gina Merchant
Ra Merchant
István Merchenthaler
Catherine Mercier
Frédéric Mercier
Saadet Mercimek-Andrews
Bryan Mercurio
Valentina Mercurio
Hussein Merdan
Mehmet Merdan
Andreas Merdes
Dorota Merecz
Marc Meredith
Rhiannon Meredith
Kim Meredith-Jones
Stephane Meresse
Irmgard Merfort
Judith Mergen
Dustin Mergott
Joe Merheb
Ashley Merianos
Celine Meriaux
Daniele Merico
Alberto Meriggi
Adalberto Merighi
Henry Meriki
Stephen Meriney
Diana Merino
Susana Merino
Marek Meristo
Ruth Merkatz
Béla Merkely
Matthias Merker
Katrina Merkies
Alexander Merkler
Rebecca Merkley
Arben Merkoçi
Daphne Merkus
Arcangelo Merla
Caterina Merla
Giampaolo Merlini
Andrea Merlo
Barbara Merlo

Juan Merlo
Lisa Merlo
Elodie Merlot
Robin Mermelstein
Aristide Merola
Dafna Merom
Pier Luigi Meroni
Valeria Meroni
D. Merrell
William Merrick
Evelyn Merrill
Scott Merrill
Tony Merriman
Edward Merritt
Martha Merrow
Diane Merry
Lisa Merry
Andrew Merryweather
Fathia Ahmed Mersal
Haydyn Mertens
Peter Mertens
Dominik Mertz
Thomy Mertzanidou
L. M. Mertz-Henning
Joshua E. Mervis
Frederic Mery
Erin Merz
Julian Merz
Maximilian Merz
Sinisa Mesarovic
Omar Mesarwi
Md. Mesbah-Uddin
Tiziana Meschi
Clement Meseda
Elena Meseguer
Bita Mesgarpour
Prafullachandra M. Meshram
Charles Meshul
Aner Mesic
C. G. M. Meskers
Aaron Meskin
Carine Meslot
Judi Mesman
Stephane Mesnage
Laurent Mesnard
Andrew Meso
Isabel Mesquita
Joao Mesquita
Rita Mesquita
António Mesquita Montes
Enrique Mesri
Werner Mess
Piergiorgio Messa
Amed Messaood
Evangelos Messaris
Louisa Messenger
Scott Messenger
William Messer
Martin Messerle
Daniel Messerschmidt
Andre Messias
Erick Messias
J. Messika
Giovanni Messina
Janey Messina
Joseph Messina
Pablo Messineo
Daniel Messinger
Gemma Mestre
Tiago Mestre
Nadine Mestre-Francès
Luisa Mestroni
Ana Mestrovic
Bruno Mesz
Danika Metallinos-Bannasch
Norman Metanis
Paul Metaxatos
Jessica Metcalf
Brian Metcalfe
Richard Metcalfe
Mutlu Mete
Mark Metersky
Lotte Meteyard
Andrew Metha
Apiwattanakul Metha
Linda Metheny-Barlow
Louise Metherell
Caroline Methling

Mathieu Métifiot
Sunil Metkar
Matteo Metruccio
Brian Metscher
E. Metting
Noelle Metting
Giovanni Mettivier
Jonathan Metts
Adam R. Metwalli
Christine Metz
Thomas Metz
Verena Metz
Silke Metzelthin
Michael Metzen
David Metzger
Florian Metzger
Jochen Metzger
Lisa Metzger
Bernhard Metzler
Ralf Metzner
Walter Metzner
Marco Meucci
Rodrigo Meucci
Sascha Meudt
Frederic Meunier
Jean Christophe Meunier
Katharina Meurer
William Meurer
Alicia Meuret
Vincent Meuric
Ralph Meuwissen
Meike Mevissen
Dik Mevius
Ricarda Mewes
Louise Mewton
Anne Mey
David Meya
Patrick Meybohm
Bernd Meyburg
Andreas Meyer
Antje Meyer
Christian Meyer
Diogo Meyer
Dixie Meyer
Eric Meyer
Everett Meyer
Gretchen Meyer
Harry A. Meyer
Joel Meyer
Justin Meyer
Katie Meyer
Keith Meyer
Marc Meyer
Marion Meyer
Markus Meyer
Martin Meyer
Nuala Meyer
Philippe Meyer
Sara Meyer
Stefan Meyer
Thomas Meyer
Timothy Meyer
Wieland Meyer
Franz-Josef Meyer-Almes
Christine H. Meyer-Frießem
Wolfgang Meyerhof
Hauke Meyerhoff
David Meyerholz
Hendrik Meyer-Lückel
Danie Meyer-Parlapanis
Gesine Meyer-Rath
A. Meyers
Stuart Meyers
Tammy Meyers
Catherine Meyer-Schwesinger
Beth Meyerson
Shari Meyerson
Delphine Meynard
Dominique Meynial-Denis
David Meyre
Pieter Meysman
Stephane Meystre
Cesar A. Meza-Herrera
Edward Mezones-Holguin
Delia Mezzanzanica
Rinaldo Mezzarane
Eleonora Mezzaroma
Marcello Mezzasalma

Maria Lina Mezzatesta
Maura Mezzetti
Andrew Mgaya
Maroun Mhanna
Gu Mi
Jianguo Mi
Jian-Xun Mi
Qing-Sheng Mi
Patrick Miailhes
Melissa Mialon
Nicholas D. Mian
Annemarie Miano
Silvia Miano
Chiyuan Miao
Hongmei Miao
Minmin Miao
Qiucheng Miao
Sheng Miao
Xiaoping Miao
Xuexia Miao
Tomasz Miazgowski
Luca Micci
Maura Miccò
Francesco Miceli
Rosalba Miceli
Corinne Miceli-Richard
Damian Michael
Davis Michael
John Michael
Tryba Michael
Ward Michael
Kai Michaelis
Thomas C. T. Michaels
Jakob Michaëlsson
Peter Michaely
Kyriaki Michailidou
Ewa Michalak
Izabela Michalak
Joel Michalek
Veronika Michalkova
Dominik Michalski
Rafał Michalski
Stefan Michalski
Beate Michalzik
Benjamin Michaud
Dominique Michaud
J. P. Michaud
Pierre-Andre Michaud
Chloé Michaudel
Magali Michaut
Johan Michaux
Olivier Micheau
Andrew Michel
Brady Michel
Clotaire Michel
Eric Michel
Marc Michel
Martin Michel
Sebastian Michel
Thomas Michel
Noseda Michela
Manuele Michelessi
Thomas Michelet
Davide Micheli
Paul Michelin
Katherine Michelmore
Paola Michelozzi
Aaron Michels
Monique Michels
Brigitte Michelsen
Hope Michelson
Christopher Michetti
Denis Michez
Annelies Michiels
Carine Michiels
Chris Michiels
Jan Michiels
Sarah Michiels
Toshimi Michigami
Ryoma Michishita
Gracjan Michlewski
Richard (Rick) Michod
Alan Mickelson
Kristin Mickelson
Karen Mickle
Elizabeth Micks
Vicente Micol
Jaap Middeldorp

John Middlebrooks
Frank Middleton
Michael Middleton
Adam C Midgley
Adrian Midgley
Claire Midgley
Nicholas Midgley
Rune Midjord
Anne Midwinter
Katarzyna Miekus
Alba Miele
Lucio Miele
Mara Miele
Michael Mielewczik
Gregore Mielke
H. Mielke
Martin Mielke
Andreas Mierau
Jochen Mierau
Gabry Mies
Miguel Mies
Nova Mieszkowska
Alexander Miething
Elizabeth Mietlicki-Baase
Markus Miettinen
Muhammad Miftahussurur
Egidia Miftode
Zoe Migicovsky
Masao Migita
Americo Migliaccio
Anna Rita Migliaccio
Chris Migliaccio
Silvia Migliaccio
Alessandro Miglietta
Maria Angelica Miglino
Lucia Migliore
Simone Migliore
Carolina Migliorell
Christine Migliorini
Paola Migliorini
Antonino Mignano
John E. Mignano
Olivier Mignen
Emmanuel Mignot
Antonio Mignucci
Véronique Migonney
Florence Migot-Nabias
Florencia Miguel
Irene Miguel-Aliaga
Jesus Miguez
Andrei Mihalca
Rada Mihalcea
Kata Miháltz
Jozsef Mihaly
Masahito Mihara
Takahiro Mihara
Kenichi Miharada
Marius Mihăşan
Carmen Mikacenic
Takashi Mikawa
Jeyhun Mikayilov
Constantinos Mikelis
Thomas Mikeska
Alexandra Mikhailova
Vladimir Mikheev
Takayuki Miki
Toshio Miki
Tsuyoshi Miki
Dariusz Mikielewicz
Ronni Mikkelsen
Torben Mikkelsen
Pika Miklavc
Benjamin Miko
Antonina Mikocka-Walus
Moïra Mikolajczak
Renata Mikstacka
Mario Mikula
Katarina Mikusova
Borja Milá
Joan Milam
Katrina Milaney
Giuseppina Milano
Vladimir Milanov
Mariofanna Milanova
Alexander Mildner
Michael Mildner
Luigi Milella
Olga Milenkaya

Vladimir Milenkovic
Oliver Miler
Anna Miles
Darryl Miles
Michael Miles
Susie Miles
Jennifer Miles-Chan
Andrés Milessi
Isabelle Miletich
Mila Mileva
Taciano Milfont
Catarina Milheiriço
Raffaele Milia
Yoamel Milián-García
Andreas Milias-Argeitis
Franco Milicchio
Pietro Milillo
Fabio Milioni
Konstantinos Milios
Piotr Milkiewicz
José Geraldo Mill
Javier Millan
Maria S. Millan
Carmen Millán
José Millán
Andrew Millar
Anthony Millar
Heather Millar
Jocelyn Millar
Robert Millar
Matthew Millard
Peter Millard
Douglas Millay
Magali Millecamps
A. Miller
Adam Miller
Andrea Miller
Anthony Miller
Arne Miller
Boris Miller
Brandon Miller
Bryan Miller
Christopher Miller
Claire Miller
Cory Miller
Craig Miller
Dana Miller
Daniel C. Miller
David Miller
Elizabeth Miller
Freeman Miller
Galen Miller
Geoffrey Miller
Grover Miller
Helen Miller
Ingrid Miller
J. Miller
Jason Miller
Jennie Miller
Jesse Miller
Jessica Miller
John Miller
Jonathan Miller
Katherine Miller
Kyle Miller
Laura Miller
Lawrence Miller
Luis Miller
Luke Miller
Lyndsey Miller
Mark Miller
Michael Miller
Michelle Miller
Patti Miller
Paul Miller
Rachel Miller
Richard Miller
Robert Miller
Ross Miller
Samantha Miller
Scott Miller
Shelley Miller
Steven A. Miller
Susan Miller
T. Miller
Veronica Miller
William Miller
Wolfgang Miller

Yifat Miller
Emily Miller-Cushon
Eydie Miller-Ellis
Elise Miller-Hooks
Kobe Millet
Maurice Millet
Oscar Millet
Glenn Millhauser
Pjm Milligan
George Millington
Jeffrey Millman
Aaron Mills
Caitlin Mills
Daniel Mills
David Mills
Edward Mills
Ian Mills
Judith Mills
Kingston Mills
Kristen Mills
L. Mills
Peter Mills
Suzanne Mills
Joshua Millstein
Jason Millward
Deborah Milly
Uzi Milman
Elizabeth Milne
Ginger Milne
George Milner
Joel Milner
Teresa Milner
Ron Milo
Staša Milojević
Jadranka Milosevic
Jean Milot
Tanja Milotic
Milan Milovancev
Natalia Milovantseva
William Milsom
Wolgang Miltner
Jana Milucka
Sophie Milward
R. Mimoto
Michael Mimouni
Booki Min
Jung-Joon Min
Kyungyoon Min
Paul Min
Wang Min
Masato Minabe
Alireza Minagar
Yuko Minami
Susumu Minamisawa
Kanshi Minamitani
Toshifumi Minamoto
Toshinari Minamoto
Harry Minas
Ioannis Minas
Shohei Minato
Kevin Minbiole
Thomas Minckley
Mark Minden
Jeroen Minderman
D. L. Mindry
Atsushi Mine
Rebecca Minehart
Y. Mineharu
Alessandra Minelli
Alessandro Minelli
Chieko Mineo
J. R. Mineo
Jeffrey Miner
Jonathan Miner
Giovanni Minervini
Antonio Minervino
Emmanuel Minet
Alberto Minetti
Lei Ming
Li-June Ming
Long Chiau Ming
Tsang Ming
Yue Ming
Ismael Mingarro
John Mingers
Karl Minges
Ma Mingguo
Matteo Minghetti

Paola Minghetti
Marco Minghini
Li Minghui
Jesus Mingorance
Lyi Mingyang
Pham Minh
Quang Bui Minh
Chris Minion
Richard Mink
Piotr Minkiewicz
Jessica Minnier
Giuseppe Minniti
Yoshihisa Mino
Subhash Minocha
Akiko Minoda
Mari Mino-Kenudson
Philip Minor
J. Minov
Silvia Minozzi
Katja Mintenbeck
Justine Mintern
Carolina Minte-Vera
Coilin Minto
Gianna Minton
Simone Minucci
Monika Mioduchowska
Lisa Miorin
Pierre Miossec
José Miotto
Marie-Christine Miquel
Sylvie Miquel
Johanna Miquet
Reyazul Rouf Mir
Behnud Mir Djawadi
Fatemeh Mir Najafi Zadeh
Lucia Mirabella
Massimiliano Mirabella
Maria Miragaia
Mariella Miraglia
Nandita Mirajkar
Emily Miraldi
Virginia Miraldi Utz
Rodolfo Miralles
Kildare Miranda
Rajesh Miranda
Roberto Miranda
Susan Miranda
Rafael Dreux Miranda Fernandes
Genaro Miranda-de la Lama
Juan Marcos Mirande
Ferdinando L. Mirarchi
Andrew Mirelman
Marta Miret
Haïtham Mirghani
Aboutaleb Miri
M. Miri
Mohammad Miri
Katherine Mirica
Gudrun Mirick Mueller
Domenico Miriello
Luca Mirimin
Aslan Miriyev
Gianluca Mirizzi
Dragan Mirkov
Bojan Mirkovic
Alemnesh Mirkuzie
Guadalupe Miro
Richard Miron
Talya Miron-Shatz
Paola Mirra
Takumi Misaka
Masaya Misaki
Harald Mischak
Giovanni Misciagna
Nicolas Misdariis
Naobumi Mise
Awoke Misganaw
Alexander Misharin
Masanori Mishima
Alexander S. Mishin
Yuji Mishina
Anjali Mishra
Bibhuti Mishra
Biswajit Mishra
Deepak Mishra
Dheerendra Mishra
Krishnaveni Mishra
Kumar Vijay Mishra

Manish Mishra
Manoj Mishra
Nagendra Mishra
Neerad Mishra
Neeti Mishra
Niranjan Mishra
Nishant Mishra
Puneet Mishra
Rakesh Mishra
Rupesh K. Mishra
Sandeep Mishra
Saroj Mishra
Seema Mishra
Sharmistha Mishra
Shashank Mishra
Shiraz Mishra
Sujata Mishra
Umakant Mishra
Vineet Mishra
John Misiaszek
Bratislav Misic
Cristina Misic
Jason Miska
Vladimir Miskovic
Diva Kant Misra
Hari Misra
Madhusmita Misra
Parimal Misra
Ravi Misra
Usha Misra
Anita Misra-Hebert
Cristina Missale
Gabriele Missale
Nabiha Missaoui
Paolo Missier
Stacey Missmer
Giovanni Mistraletti
Michele Mistri
Hiten Mistry
Ichiro Misumi
Justyna Miszkiewicz
Pawel Misztal
Damiano Mita
Delphine Mitanchez
Namiko Mitarai
Satoshi Mitarai
Josef Mitáš
Patrick Mitashi
William Mitch
Aaron Mitchell
Braxton Mitchell
Brett Mitchell
Cameron Mitchell
Carl Mitchell
David Mitchell
James Mitchell
Jonathan Mitchell
Pamela Mitchell
Patricia Mitchell
Piers Mitchell
Robert Mitchell
Rod Mitchell
Rowan Mitchell
Suzanne Mitchell
Thomas Mitchell
Bradley Mitchelmore
Deborah Mitchison
Axel Mithöfer
Tijana Mitic
Timur Mitin
Asako Mito
Mihail Mitov
Abhijit Mitra
Alok Mitra
Chiranjit Mitra
Devarati Mitra
Kalyan Mitra
Sophie Mitra
Suman Mitra
Sushanta Mitra
Achilleas Mitrakas
Tarik Mitran
Naim Mitre
Veronica Mitroi
Ragai Mitry
Dimos Mitsikostas
Georgios Mitsis
Charalampos Mitsonis

Dimitris Mitsouras
Tetsuya Mitsudomi
Satomi Mitsuhashi
Yozo Mitsui
Dai Mitsushima
Yoshiaki Mitsutake
Ashwani Mittal
Manish Mittal
Shubham Mittal
Sumeet Mittal
Aaron Mittel
Holger Mitterer
John Mittler
Thomas Mittlmeier
Sibylle Mittnacht
Luigi Mittone
Steffen R. Mitzner
Wayne Mitzner
Fuminari Miura
Kazuki Miura
Kazutoyo Miura
Shin-Ichiro Miura
Shinji Miura
Takashi Miura
Tanya Miura
Yuichiro Miura
Yuri Miura
Yutaka Miura
Koji Miwa
Kunihisa Miwa
Kyoko Miwa
Makoto Miwa
Yoshihiro Miwa
Franklin Mixon
Masaki Miya
Elen Miyabara
Hiroko Miyadera
Keiko Miyadera
Taku Miyagawa
Masaru Miyagi
Yoshihiro Miyahara
Kazunori Miyake
Kensaku Miyake
Sanae Miyake
David T. Miyamoto
Shigeki Miyamoto
Shimpei Miyamoto
Yohei Miyamoto
Kazuo Miyashita
Kazutoshi Miyashita
Atsuro Miyata
Kanjiro Miyata
Shingo Miyata
Takeshi Miyata
Makoto Miyazaki
Mariko Miyazaki
Shinya Miyazaki
D. Miyoshi
Eiji Miyoshi
Hideaki Miyoshi
Takemasa Miyoshi
Toru Miyoshi
Mark Mizee
Ivana Mizikova
Atsushi Mizoguchi
Koji Mizoguchi
Yaffa Mizrachi Nebenzahl
Toru Mizuguchi
Hiroki Mizukami
Kenji Mizumoto
Shuji Mizumoto
Kensaku Mizuno
Ryuichi Mizuno
Takamasa Mizuno
Takuya Mizuno
Masayuki Mizuri
Tsunekazu Mizushima
Katsumi Mizuta
Koji Mizutani
Giovanni Mizzi
Ger Mjoes
Amosy M'Koma
Bulukani Mlalazi
Agnieszka Mlynarska
Coceka N. Mnyani
Dorcas Mnzava
Delin Mo
Fuhao Mo

Ji-Hun Mo
Phoenix Mo
Xiumei Mo
Yin-Yuan Mo
Yuanhan Mo
Marocolo Moacir
R. Moaddel
Gila Moalem-Taylor
Hounaida Moalla
Eman Moawad
M. Moazen
Maryam Moazzam Jazi
A. Mobashsher
Saleh Mobayen
Jennifer Mobberley
Aaron Moberly
James Mobley
William Mobley
Andrei Mocan
Francesco Moccia
Marcello Moccia
Simone Mocellin
Shirra Moch
Koji Mochida
Takemi Mochida
Atsushi Mochizuki
George Mochizuki
Hiroyuki Mochizuki
Manabu Mochizuki
Daria Mochly-Rosen
Julie Mocklin
Fabio Moda
Ali Modarressirressi
Kathryn Modecki
Rugira Modeste
Pietro Modesti
Sean Modesto
Alessandra Modi
Gopesh Modi
Pranjal Modi
Shiva Kumar Modi
Surbhi Modi
Jaime Modiano
Giuseppe Modica
Sara Modig
Luzia Modolo
Martin Modriansky
Aaloke Mody
Angelica Moe
Luke Moe
Markus Moehlenbruch
Holly Moeller
Jamie Moeller
Marcus Moeller
Ralf Moeller
Michel Moenner
Stephen Moerlein
Cyril Moers
Caroline Moeser
Pim Moeskops
Søren Moestrup
Mariola Moeyaert
Lynne Mofenson
Jennifer Moffat
Scott Moffat
Luciano Moffatt
David Moffitt
Daniela Moga
Matthew Mogensen
Jacopo Moggi-Cecchi
Gert-Jan Moggre
Sheida Moghadamrad
Seyed Moghadas
Nuriel Moghavem
Abhay Moghekar
Sasan Moghimi
Jeffrey Mogil
Alex Mogilner
Axel Mogk
Baharia Mograbi
David Mogul
Hassan Mohabatkar
Niroj Mohalik
Jamaludin Mohamad
Amin Mohamadi
Ayman Mohamed
Junaith Mohamed
Mona A. Mohamed

Nadia Mohamed
Rozi Mohamed
Thahira Jamal Mohamed
Mohamed Abdelaziz
Firdaus Mohamed Hoesein
Khalid Mohamedali
Haroon Mohammad
Khalid Mohammad
Noor Mohammad
Ramzi Mohammad
Majid Mohammadhosseini
Abdolreza Mohammadi
Eesa Mohammadi
Ehsan Mohammadi
Gelareh Mohammadi
Hadi Mohammadi
Kasra Mohammadi
Mohsen Mohammadi
Manijeh Mohammadi
Dehcheshmeh
B. Mohammadi-Ivatloo
Asghar Mohammadpoorasl
Bedru Hussen Mohammed
Emad A. Mohammed
Kamal Mohammed
Kassim M. Mohammed
Amita Mohan
Ananda Sanagavarapu Mohan
Diwakar Mohan
Mahesh Mohan
Royce Mohan
Viswanathan Mohan
Nethia Mohana Kumaran
Manoj Mohanan
Venkat Mohanram
Tapan Mohanta
Bidyut Mohanty
Binayak Mohanty
Kuldeep Mohanty
Sanjay Mohanty
Smita Mohanty
Tushar Kumar Mohanty
Shyam Mohapatra
Subrajeet Mohapatra
Krishnaveni Mohareer
Sarah Moharem-Elgamal
Osama Mohawesh
Akif Mohd
Rosminah Mohd
Azmin Shakrine Mohd Rafie
Jayasilan Mohd-Azlan
Sherina Mohd-Sidik
Jeff Moher
Golam Mohi
Maura Mohler
J. Mohlman
Trine Moholdt
Vijayanand Moholkar
Alicia Mohr
Christian Mohr
Christine Mohr
Nicholas Mohr
Rachel M. Mohr
Volker Mohrholz
Yasuhiko Mohri
Pedram Mohseni
Nicholas Mohtadi
Syed Mohyud-Din
Susan Moir
Sionia Moisa
Kenneth Moise
Jennifer Moïsi
Steven Moisyadi
Elizabeth Mojica
Afsaneh Mojra
Nicola Mok
Pooi Ling Mok
Vincent Mok
Mathildah Mokgatle
Babak Mokhlesi
Parisa Mokhtari
Hossein Mokhtarzadeh
John Mokili
Maxim Mokin
Thabiso Mokotjomela
Marika Mokou
Daniela Mokra
A. Mokrowiecka

Michal Mokry
Suzanne Mol
Liliana Mola
Even Moland
Athanassios Molassiotis
Kåre Mølbak
Øyvind Molberg
Samat Moldakarimov
Alin Moldoveanu
Adrian Molenaar
Douwe Molenaar
Dylan Molenaar
Pascal Molenberghs
Marc Molendijk
Richard Molenkamp
Carla Molento
Marcelo Molento
Andrés Molero-Chamizo
Jordi Molgo
Vera Moliadze
Anthony Molina
Cristina Molina
David Molina
Esther Molina
George Molina
Gustavo Molina
Oscar Molina
Samuel Molina
Wagner Molina
Emanuela Molinari
Francesco Molinari
Jesús Molinari
Sabrina Molinaro
Ivan Molineris
Matthieu Molinier
L. Molins
Andreas Molisch
John Molitor
Juan Moliva
Joachim Molkentin
Yaroslav Molkov
Isabella Moll
Kristina Moll
Gianluca Molla
Vasiliki Mollaki
Guillaume Molle
Ann-Beth Moller
Kirsten Moller
Peter Moller
Luciana Möller
Ralf Möller
Linda Sevelsted Møller
Niels Møller
Peter Mollica
Faustino Mollinedo
Barbara Molnar
Julia Molnar
Zsolt Molnar
Viktor Molnár
Kirk Moloney
Elena Moltchanova
Massimo Molteni
Maria Molto
Philip Molyneaux
Paolo Momigliano
Abdulrahaman Momin
Henrique Momm
Franco Momoli
Ryo Momosaki
Anne-Mette Momsen
Fiammetta Monacelli
Claudia Monaco
Marie Monaco
James Monaghan
Sean Monaghan
Paul Monahan
Philip Monahan
Pascal Monasse
Roberto Monastero
Jean Claude Monboisse
Marie-Hélène Moncel
Darren Monckton
Jessica Moncrieff-Boyd
Abdul Mondal
Debasis Mondal
Dinesh Mondal
S. Mondal
Sukanta Mondal

Mauro Mondelli
Stefania Mondello
Bernardo Monechi
Pablo Monedero
Marc Monestier
Arthur Money
Amparo Monfort
Scott Monfort
Manuel Monfort-Pañego
Janet Monge
Philippe Mongeon
Gianluigi Mongillo
Juthathip Mongkolsapaya
Emmanuel Mongodin
Sébastien Mongrand
Francisco Moniche
Karine Monier
Mohammed Moniruzzaman
Marcin Moniuszko
Norihito Moniwa
Lucas Monje
M. Monje
David Monk
Gerald Monk
Cole Monnahan
Dominique Monnet
Tony Monnet
Xavier Monnet
Mollie Monnig
Cyril Monnot
April Monroe
Derek Monroe
Lynn Monrouxe
Claire Monroy
Maria Monsalve
Eduard Monso
Ken Monson
Rita Monson
Lluís Mont
Vincent Montade
Christian Montag
Maria Teresa Montagna
Lucille Montagne
Anelise Montagner
Federica Montagnese
Alberto Montagnoli
Brian Montague
Carolina Montanari
Erika Montanaro
Giuseppe Montanaro
Gaspard Montandon
Eduard Montanya
Ulisses Montarroyos
Mireille Montcouquiol
Andréa Monte-Alto-Costa
Martin Montecino
Carlomaurizio Montecucco
Monica Montefalcone
Mark Monteferrante
Alvaro Monteiro
Denise Monteiro
Douglas Monteiro
Gisele Monteiro
Henrique Monteiro
Mervyn Monteiro
Wuelton Monteiro
Thiago Monteiro de Paiva Fernandes
Matilde Monteiro-Soares
Denise Montell
Filippo Montemurro
Carlos Montenegro
Lucía Monteoliva
Daniel Montero
Javier Montero
Jesus Montero-Marin
Diego Montes
Rosa Montes
Diogo Montes Vidal
Giovanni Montesano
Pau Montesinos
Christophe Montessuit
Marco Montevecchi
Sandra Montezuma
Robert Montgomerie
Elizabeth Montgomery
Gw Montgomery
Paul Montgomery

Ruth Montgomery
Giulia Monti
Lucie Monticelli
Silvia Monticelli
Marco Monticone
Robert Monticone
Alonso Montiel-Luque
Natalia Montinari
Arnold Monto
Monica Montopoli
Raquel Montorio
Paola Montoro
Miguel Montoro Girona
Juan Carlos Montoy
José María Montoya
Maria Montoya
Ben Montpetit
Luke Montrose
Arnaud Monty
Chelsea Monty
Burrell Montz
Michael Monuteaux
Catalina Monzon
Eng Kuan Moo
Anthony Moody
Eric Moody
Heather Moody
Howard Mooers
Richard Moog
Frits Mooi
Simon Mooijaart
Marlon Mooijman
Changjong Moon
Hyeong-Gon Moon
Katherine Moon
Nathalie Moon
Richard Moon
Seok Jun Moon
Joshua Mooney
Robert Mooney
Scott Mooney
Ravi Moonka
Veelasha Moonsamy
Molly Moor
Arshag Mooradian
Jon Moorby
Albert Moore
Allen Moore
Andrew Moore
Anna Moore
Ashlee Moore
Bethany Moore
Brandon Moore
Bret Moore
Catrin Moore
Christopher Moore
Daniel Moore
Dirk F. Moore
Francis Moore
Ginny Moore
Hunter Moore
Jennifer Moore
John Moore
Julie Moore
Kathleen Moore
Kristen R. Moore
Lynne Moore
Michael Moore
Nathan Moore
Robert Moore
Sean Moore
Simon Moore
Steven Moore
Thomas Moore
Roger Moorehead
Carolyn Moores
Greg B. G. Moorhead
David Moorman
Thomas Moorman
Pieter Moors
R. N. Moorthi
Shhyam Moorthy
Johra Moosa
Mohammed Moosa
Mahmood Moosazadeh
Andreas Moosmann
Vamsi Mootha
Robert Moots

Orna Mor
Tsafrir Mor
Zohar Mor
Higinio Mora
Javier Mora
Juan Mora
Thierry Mora
Alessandro Morabito
Luis Moraes
Renato Moraes
Theo Moraes
Thyago Moraes
Flávia Moraes Silva
Ivana Moraes-Silva
Pedro Manoel Moraes-Vieira
Santiago Mora-Garcia
Concepció Moragrega
Christudas Morais
Gracyelle Morais
Carlos Morais de Alencar
Cleopatra Moraiti
Oyewale Morakinyo
Aixa Morales
Aythami Morales
Diana Morales
Hernan Morales
J. Alejandro Morales
Juan Morales
Narkis Morales
Patricio Morales
Rodrigo Morales
Jose Morales Gordon
Sara Luz Morales Lázaro
Maria Morales Suárez-Varela
Ana Morales-Arce
Antonio Morales-Artacho
Luis E. Morales-Buenrostro
Juan Mora-Macías
Hector Mora-Montes
Andrew Moran
Brendan Moran
Chris Moran
David Moran
Gary Moran
John Moran
Jose Moran
Julio Moran
Nancy Moran
Niamh Moran
Patrick Moran
Serge Morand
Anita Morandi
Maria Moran-Diez
Claire Morandin
Pierre-Emmanuel Morange
Jose Moran-Mirabal
Ingo Morano
Jamie Morano
Joan Moranta
Roser Morante
José Carlos Morante-Filho
Borja Mora-Peris
Raphael Morard
Rosario Moratalla
Manuela Morato
Gonzalo Moratorio
Stephan Moratti
Molly Moravek
Henning Morawietz
Roger Morbey
Umberto Morbiducc
Marcello Morciano
Douglas Morck
Clifford Morden
Dana Mordue
Viatcheslav Mordvinov
Pierre-Arthur Moreau
James Morehen
Alexandre Moreira
Catarina Moreira
Irina Moreira
Ivone Moreira
João Moreira
João Nuno Moreira
Luciana Moreira
M. Teresa Moreira
Miguel Moreira
Paula Moreira

Renata Moreira
Rodrigo Moreira
Wilfried Moreira
Bernardo Moreira Soares Oliveira
Carlos Moreira-Filho
Luis Moreira-Matias
Jean-Michel Morel
Nicole Moreland
Annamaria Morelli
Eugenio Morelli
Sara Morelli
Thiago Morelli
Fabio Morellini
Laura Morello
Kelley Moremen
Louis-Philippe Morency
Alejandra Moreno
Andrea Moreno
Diana Jennifer Moreno
Diego Moreno
Eduardo Moreno
Federico Moreno
Ignacio Moreno
Patricia Moreno
Renata Moreno
Silvia Moreno
Yolanda Moreno
Débora Lina Moreno Azócar
Claudia Ximena Moreno Herrera
Thiago Moreno L Souza
Max Moreno Madriñán
Andres Moreno-Estrada
Rubén Moreno-Opo
Rino Morent
Antonio Moretti
Fabiola Moretti
Marcelo Moretti
Marco Moretti
Rita Moretti
Rocco Moretti
Angelo Moretto
Amy Morey
Fernando Morgado-Dias
Andrei Morgan
Angela Morgan
Daniel Morgan
Ethan Morgan
Gareth Morgan
Gary Morgan
Ian Morgan
Kerri Morgan
Lucy Morgan
Matthew Morgan
Michael Morgan
Perri Morgan
R. G. Morgan
Richard Morgan
Stephanie Morgan
Thomas Morgan
Mary Morgan-Richards
Francesca Morgante
Susan Morgello
Daniel Morgensztern
Maria Grazia Morgese
Floriana Morgillo
Chisato Mori
Emiliano Mori
Kazuhiko Mori
Kiyoshi Mori
Marcelo Mori
Masahiro Mori
Noriko Mori
Shintaro Mori
Takefumi Mori
Trevor Mori
Brivaëla Moriceau
Mario Luca Morieri
Hitoshi Morikawa
Dimitrios Morikis
Maria Morilla
Lilian Morillo
Kozo Morimoto
Libby Morimoto
Mitsuru Morimoto
Naoki Morimoto
Satoshi Morimoto
Shauna Morimoto

Daniel Morin
Evelyn Morin
Jason Morin
Jean Benoit Morin
Olivier Morin
Jérôme Morinière
Béatrice Morio
Sho Morioka
Tomoaki Morioka
Marco Moriondo
Joaquin Moris
Naho Morisaki
Takayuki Morisaki
Anselmo Moriscot
Eriko Morishita
Donald Morisky
Ian Morison
Akimichi Morita
Hiroki Morita
Hironobu Morita
Takayoshi Morita
Teppei Morita
Toshisuke Morita
Charlotte Moritz
Orson Moritz
Kenta Moriwaki
Jun Moriya
Minoru Moriyama
Ignacio Moriyon
Yuki Morizane
Markus Morkel
Alexander Morley
Bruce Morley
Celeste Morley
Gregory Morley
James Morley
Kirsten Morley
Neil Morley
Peter Morley
Patrica Morley-Forster
Michael Morlock
Ana Moro
Loredana Moro
Pedro Moro
Ana Moro-Egido
Yoichi Morofuji
Francisco J. Morón
Renato Morona
Giovanni Morone
James Moroney
Francesco Moroni
Suzanne Morony
Lavinia Morosi
Andrea Morotti
Alireza Morovat
Giulia Morra
Nuria Morral
Michael Morran
Maureen Morrin
Adam Morris
Brian Morris
Claudia Morris
David Morris
Douglas Morris
Gareth A. Morris
Genela Morris
Gwilym Morris
James Morris
Jeff Morris
Jill Morris
Julie Morris
Kasey Lynn Morris
Katherine Morris
Margaret Morris
Martin Morris
Meg Morris
N. Morris
Nathan Morris
Susan Morris
Brett Morrison
Ciaran Morrison
Filomene Morrison
Helena Morrison
Janna Morrison
Katherine Morrison
Kathryn Morrison
Malcolm Morrison
Steven Morrison

Trudy Morrison
Richard Morriss
Anne Morrissey
Christy Morrissey
Ember Morrissey
Karyn Morrissey
Patrocinio Morrondo
Doralba Morrone
Doralisa Morrone
Juan Morrone
Alexandre Morrot
Adam Morrow
Bernice Morrow
Brenda M. Morrow
Christopher Morrow
Johnica Morrow
Kathleen Morrow
Melissa Morrow
Marco Morsch
Joachim Morschhäuser
C. Morsczeck
Caryn Morse
Christopher Morse
Gene Morse
Michael Morse
Patrick Morse
Tracy Morse
Alexander Mörseburg
John Morser
Bashir Morshed
Giulia Morsica
Bryce Morsky
Sara Morsy
Richard Mort
Niyousha Mortaza
Jennifer Mortensen
Laust Mortensen
Motazakker Morteza
Philippe Mortier
Ann Mortimer
Jeanne Mortimer
Alec Morton
Craig Morton
David Morton
Gregory Morton
Jude Morton
Richard Morton
Sarah Morton
Golam Mortuza
Anita Morzillo
David Moscatelli
Marco Moschetta
Michele Moschetta
Matthew Moscou
Michael Moseley
Michael Moser
Fausta Mosha
Solomon Moshe
Solomon Moshé
Deane Mosher
Sergei Moshkovskii
Dimitrios Moshou
Christian Mosimann
Jonathan Mosley
Anne Mosnier
Mildrey Mosquera Escudero
Alan Moss
Andrew Moss
Jennifer Moss
John Moss
Paul Moss
Richard Moss
Robert Moss
Samantha Louise Moss
Mahmud Mossa-Basha
Amr Mossad
Enrico Mossello
Michael Mossing
Barbara Mostacci
Ahmed Mostafa
Dalia Mostafa
Heba Mostafa
Habib Mostafaei
Giovanni Mostile
Serge Mostowy
Alejandro Mota
Luís Mota
Manuel Mota

Babu Motagi
Marzieh Motallebi
Kou Motani
Ramzi Ahmed Abdullah
Mothana
Bianca Mothé
Artur Motheo
Carmel Mothersill
Kathleen Motil
Lukas Motloch
Hideyuki Motohashi
Norio Motohashi
Tsutomu Motohashi
Goro Motomura
Chie Motono
Edith Motschall
Jean-Paul Motta
Philip Motta
Daisy Motta-Santos
Hans Motte
Roberto Motterlini
Denis Mottet
Michelle Mottola
Jeremy Mottram
Katherine Motyl
Haiwei Mou
Xiaozhen Mou
Zhonglin Mou
Sébastien Mouchet
Vítězslav Moudrý
Stavroula Mougiakakou
Fleur Mougin
Abdeslam Mouihate
Bernard Mouillac
Jean Francois Mouillet
Etienne Mouisel
David Moulin
Joanna Moullin
Lawrence Moulton
Peter Mount
Adrian Mountford
Giorgos Mountrakis
Nina Mounts
Felipe Moura
Claudia Moura Gallo
Hashem Mourad
Pierre Mourad
Gulherme Mourao
Miguel Mourato
Stefanos Mourdikoudis
Mourad Moursi
Dimitris Mourtzis
Shaaban Mousa
S. Jamshid Mousavi
Seyed Mohsen Mousavi
Mehdi Moussaïd
Aline Moussard
Khaled Moustafa
Mohamed Moustafa
Romana Moutelikova
Michele Mouttapa
Jamileh Movassat
Narine Movsisyan
Sujana Movva
Chishimba Mowa
Danielle Mowery
Lorna Moxham
Katherine Moxley
Rodney Moxley
Christopher Moxon
Sarah Moxon
F. M. Moy
Francisco Moyano
Brian R. Moyer
Cheryl Moyer
Craig Moyer
Joshua Moyer
David Moyes
K. Moyes
Rita Moyes
Cynthia Moylan
Gene Moyle
Meghan Moynihan
Ray Moynihan
Faith Moyo
Sabrina John Moyo
Sikhulile Moyo
Paul Mozdziak

Ioana Mozos
Pallab Mozumder
Raimondas Mozuraitis
David Mphuthi
Anastasios Mpotsaris
Jaroslav Mráz
Jakub Mrazek
Jan Mrazek
Kandadai Mridula
Markéta Mrkvová
Dan Mroczek
Dariusz Mrozek
Randall Mrsny
Saraswathi Ms
George Msalya
Rachel Msetfi
Ramadhan Mstafa
Thuli Mthiyane
Chang-Kao Mu
Dezhi Mu
G. Mu
Nan Mu
Shengyu Mu
Tingwei Mu
Wenbo Mu
Xingjiang Mu
Xiuqian Mu
Yanshuang Mu
Yifen Mu
Yuguang Mu
Chatchai Muanprasat
Thierry Muanza
Marco Mucciarelli
Cristina Muccioli
Giulio Muccioli
Dario Pasquale Mucciolo
Lev Muchnik
Thomas Mücke
Susan Mudambi
Onur Mudanyali
Giridhar Mudduluru
Poonam Mudgil
Rajini Mudhasani
Maria Mudryj
Thomas Muehleisen
Atis Muehlenbachs
Jochen Muehlschlegel
Anne Mueller
Charles Mueller
Christa Mueller
Christian Mueller
Christopher Mueller
Dagmar Mueller
Eugene Mueller
Geoffrey Mueller
Kathryn Mueller
Keith Mueller
Kim Mueller
Klaus Mueller
Rebecca Mueller
Ryan Mueller
Scott Mueller
Sebastian Mueller
Tara Mueller
Ute Mueller
William Mueller
Wolfram Mueller
Saskia Muellmann
Marcus Muench
Stephen Muench
Jannes Muenchow
Anne Muesch
Kim Mueser
Stefan Muetzel
Ilgiz Mufazalov
Stefanie Muff
Rachel Mugge
Lucia Muggia
Catrina Mugglin
Lapo Mughini-Gras
Iñaki Mugica
Andrew Mugler
Valdair Muglia
Cecilia Mugnai
Nelly Mugo
Peter Mugo
Nikolai Mugue
Geana Mugur

Irfan Muhammad
Taseer Muhammad
Abdul Kadar Muhammad Masum
Oumer Muhammed
Ghaffar Muharram
Mark Mühlau
Lawrence Muhlbaier
Ruben Mühl-Benninghaus
Kristin Mühldorfer
Paul Muhle-Karbe
Thomas Mühlenberg
Kristin Mühlenbruch
Timo Mühlhaus
Yvonne Mühlig
Sandra Mühlstädt
Andreas Muhs
Khitam Muhsen
Paolo Muiesan
Florian Muijres
Keith Muir
Peter Muir
Taulant Muka
Asumpta Mukabutera
Elizabeta Mukaetova-Ladinska
Kanae Mukai
Nyirenda Mukanthu
Manishi Mukesh
A. Mukherjee
Ashis Mukherjee
Debanjan Mukherjee
Jogeshwar Mukherjee
Nabanita Mukherjee
Pulok K. Mukherjee
Raju Mukherjee
Sandip Mukherjee
Satyam Mukherjee
Sayan Mukherjee
Shomen Mukherjee
Shrabani Mukherjee
Siddhartha Mukherjee
Sunil Mukherjee
Trena Mukherjee
Andrey Mukhin
Ashis Mukhopadhyay
Chinmay Mukhopadhyay
Debabrata Mukhopadhyay
Debashis Mukhopadhyay
Indranil Mukhopadhyay
Nitai Mukhopadhyay
Rupak Mukhopadhyay
Saikat Mukhopadhyay
Subhadip Mukhopadhyay
Tariq Mukhtar
Ivan Mukisa
Trilochan Mukkur
Masahi Mukoyama
Sharif Mukul
Harshini Mukundan
D. Mul
Joram Mul
Marloes Mul
Marco Mula
Himaya Mula-Michel
Marta Mulawa
Abby L. Mulay
Jean Mulcahy Levy
Christa Mulder
Mark Mulder
Pamela Mulder
Rosa Mulder
Tessa Mulder
Katherine Muldoon
Sarah Muldoon
Giuseppe Mule
Flavia Mulè
Miquel Mulero
Carly Muletz
James Mulholland
Padraig Mulholland
Kristine Mulhorn
Shai Mulinari
Stefan Muljo
Luke Mullany
Stephanie Mullen
William Mullen
Alexandre Muller
Christo Muller

Daniel Muller
K. Muller
Matthew Muller
Paulo Muller
Sylviane Muller
Yunhua Muller
Caroline Müller
Juliane Müller
Klaus-Robert Müller
M. C. Müller
Marcel Müller
Marcus Müller
Markus Müller
Rolf Müller
Tobias Müller
Wolfgang Müller
Jana Müllerová
Michaela Müller-Trutwin
John Mullet
Francois Mullier
Connie Mulligan
Desmond Mulligan
Megan Mulligan
James Mullin
Conrad W. Mullineaux
Benjamin Mullins
David Mullins
Ewen Mullins
James Mullins
Paul Mullins
Robert Mullins
Joaquim Mullol
Gerd Multhaup
Kristi Multhaup
Gabriele Multhoff
Wondemagegn Mulu
Surafel Mulugeta
Shawn Mulvaney
Matthew Mulvey
Michael Mulvey
John Mulvihill
Minal Mulye
Khalid Mumtaz
Marcus Munafo
Masanori Munakata
Hetron Munang'andu
Luca Munaron
Lankeshwara Munasinghe
Mohammed Munavvar
Cesar Munayco
Daniel Munblit
Jelena Muncan
Jan Munch
Martina Mund
Sibu Mundiyanapurath
Christopher Mundorf
Emily Mundorff
Filip Mundt
Christopher J. Mundy
John Mundy
Tomoatsu Mune
Alejandro Múnera
Kristen Munger
Imran Mungrue
Khátia Munguambe
Diego Munguía-Izquierdo
Givemore Munhenga
Carolina Munhoz
Daniel Munich
Nira Munichor
Maite Muniesa
Arasambattu Kannan Munirajan
David Munisi
Graciela Muniz-Terrera
Carmine Munizza
Heidi Munk
Marion Munk
S. Munnangi
Angel Munoz
Francisco Munoz
Ricky Munoz
Iker Muñoz
Irene Muñoz
Isabel Muñoz
Javier Muñoz
Luis Muñoz
Marta Muñoz
Nacira Muñoz

Roldan Muñoz
Samuel Muñoz
Marta Munoz Colmenero
Miguel Muñoz-Alía
Carmen Muñoz-Almagro
Maria Munoz-Amatriain
Carla Muñoz-Antoli
Juan Luis Muñoz-Bellido
Rosario Muñoz-Clares
Maider Muñoz-Culla
José M. Muñoz-Felix
Emma Muñoz-Moreno
Jose A. Muñoz-Moreno
Maria Teresa Munoz-Quezada
Carol Munro
Cindy Munro
Natalie Munro
Samantha Munroe
Michelle Munro-Kramer
Anjana Munshi
Kiraat Munshi
Jeffrey Munson
Mary Munson
Jordi Muntané
Thomas Münte
Tendai Munthali
Tim Muntinga
Murugan Munusamy
Kylie Munyard
Usha Muppirala
Lina Mur
Luis Mur
Maurizio Muraca
Melissa Muradian
Koji Murai
Akira Murakami
Kazuhiro Murakami
Makoto Murakami
Masami Murakami
Satoshi Muraki
Krishnamurthy Muralidhar
Vasant Muralidharan
Chisako Muramatsu
Giacomo Murana
Eduard Murani
Konstantin Muranov
Satoshi Murao
Daisuke Muraoka
Wayne Muraoka
Elena Muraro
Paolo Muraro
Jun Murase
Jonathan Muraskas
Dorothee Murat
Kazumoto Murata
Mika Murata
Takahisa Murata
Takayuki Murata
Claudia Muratori
Steve Murawski
Moses Muraya
Hiroshi Murayama
Roberto Murcio
Kyle Murdock
Giuseppe Murdolo
Ludovic Mure
M. Murea
Dafin Muresanu
Joseph Muretta
Walter Murfee
Darnel Murgatroyd
Peter Murgatroyd
Carla Murgia
Juan Murias
Delphine Muriaux
Anarina Murillo
Angela Murillo
R. Murillo
Juan Murillo-Maldonado
Bianca Murillo-Ortiz
Shelton Murinda
Ally Murji
William Murk
Silvia Muro
Sergio Murolo
Hiroyuki Murota
Stephen Murphey
Adrianna Murphy

Bernadette Murphy
Brian Murphy
Christopher Murphy
Cormac Murphy
Darra Murphy
David Murphy
Edward Murphy
Erin Murphy
J. Murphy
James Murphy
Jamie Murphy
Kate Murphy
Kevin Murphy
Michael Murphy
Paddy Murphy
Peter Murphy
Robert Murphy
Stephen Murphy
Vanessa Murphy
William Murphy
Emerson Murphy-Hill
Aimee Murray
Alan Murray
Alexander Murray
Andrew Murray
Brian Murray
Damian Murray
Dennis Murray
Gerald Murray
Gerard Murray
James Murray
Kris Murray
Melanie Murray
Nicholas Murray
Paul Murray
Philip Murray
Samantha Murray
Sarah Murray
Laura Murray-Kolb
Ben Murrell
Ebony Murrell
Rita Murri
Monzur Murshed
Ayesha Murshid
Silvane Murta
Michael Murtaugh
Muhammed Murtaza
Padma Murthi
Krishna Murthy
Srinivas Murthy
Sriyutha Murthy
Vishnu Murty
Jayaseelan Murugaiyan
Dharmani Murugan
Kadarkarai Murugan
N. Arul Murugan
Raju Murugananthkumar
Amudhan Murugesan
S. Murugesan
Kamarul Imran Musa
Estella Musacchio
Ganeswara Musada
Umberto Musazzii
Marcelo Muscará
Keely Muscatell
David Muscatello
Antonella Muscella
Jennifer Muscha
Beate Muschalla
Gabriela K. Muschitz
Ian Musgrave
Adiel Mushi
Taisei Mushiroda
Frauke Musial
Jacek Musiał
Piotr Musiałek
Erik Musiek
Victor Musiime
Geofrey Musinguzi
Khoirul Muslim
Siti Muslimatum
Eri Muso
Antonino Musolino
Ferdinando Mussa-Ivaldi
Michele Mussap
Brent Mussel
Richard Musser
Jan Mussgnug

Margherita Mussi
Valentina Mussi
Tofy Mussivand
John Mussleman
Steven Mussmann
Orlando Musso
Kamal Mustafa
Osama Mustafa
Thomas Mustillo
Satu Mustjoki
Muhammad Mustufa
Mark Musumba
B. Musumeci
Toshizumi Muta
Marko Mutanen
David Mutch
Alexandr Muterko
Felicity Muth
Mehanathan Muthamilarasan
Srinivasa Rao Mutheneni
Gladson Muthian
M. Muthu
Magesh Muthu
Ranjan Muthukrishnan
Pandurangan Muthuraman
Vivek Muthurangu
Hariharan Muthusamy
Saminathan Muthusamy
Kerim Mutig
Franco Mutinelli
Ana Mutis
Haitham Mutlak
Mesut Mutluoglu
Tatsushi Mutoh
Stephen Mutoloki
Peter Mutschke
John Mutter
Donald Mutti
Navdeep Mutti
Bulent Mutus
Philippe Mutwa
Rüdiger Mutz
Tobias Mütze
José Muyor
Monde Muyoyeta
Abimereki Muzaale
Odwell Muzari
Raymond Muzic
Moise Muzigaba
Otto Muzik
Christina Muzny
Brighton M. Mvumi
Peter Mwaba
Waithaka Mwangi
Jessica Mwinyi
Yan My
David Myatt
Landon Myer
Robert Myerburg
Catherine Myers
Dean Myers
Edward Myers
Jenny Myers
Lara Myers
Linda Myers
Marcie Myers
Ross Myers
Teresa Myers
Hellen Myezwa
Nathan Myhrvold
Khin Myint
Eric Mykhalovskiy
Puja Myles
Sean Myles
Hannu Myllykallio
Samy Myllymaa
Mathios Mylonakis
Monika Mynarska
Timo Myohanen
Rho Shin Myong
Atle Mysterud
Karthikeyan Mythreye
Joanniis Mytilineos
Chryssi Mytilineou
Jihwan Myung
Chandrasekaran N.
Chan-Hyun Na
Dong Na

Kyoung-Sae Na
Muzi Na
Sungsoo Na
Amanda Naaum
Kesara Na-Bangchang
Ali Nabavizadeh
M. Naber
Ashraf Nabhan
A. H. M. Nurun Nabi
Ibtissem Nabti
Samia Naccache
Raffael Nachbagauer
Mathieu Nacher
Vladislav Nachev
Freddy Nachtergaele
Petr Nachtigal
Fernando Naclerio
Maliwan Naconsie
Ernest Nadal
Jean-Pierre Nadal
Jesús Nadal
Marcos Nadal
Lydie Nadal Desbarats
Francisco Nadal-Nicolas
Paulo Nadanovsky
Sophie Nadaud
Kari Nadeau
Lubna Nadeem
S. Nadeem
Tamer Nadeem
Koonlawee Nademanee
Antoun Nader
Nader Nader
Thomas Naderer
Nader Naderpajouh
Satish Nadig
Yann Nadjar
Azadeh Nadjarzadeh
Abhijit Nadkarni
Jerry Nadler
Lauren Nadler
Anna Nadolska-Orczyk
Marc Nadon
Sophie Nadot
Patricia Nadworny
Soad Nady
Abdul Naeem
M. Naeem
Somayyeh Naeemi
Michael Naef
David Naeger
Kambiz Nael
Lieve Naesens
Sabine Naessen
Vaidehi Nafade
Cristiane Naffah de Souza
Nadia Naffakh
Emerson Nafziger
Penumaka Nagababu
Genta Nagae
Ryu Nagahara
Shin Nagai
Takeharu Nagai
Jyothi Nagajyothi
Teruyoshi Nagamitsu
Hanatsu Nagano
Yukio Nagano
Tomohito Nagaoka
Bhushan Nagar
Jantakahalli Nagaraj
Ramakrishnan Nagaraj
T. G. Nagaraja
Tavarekere N. Nagaraja
Valakunja Nagaraja
Niranjan Nagarajan
R. Nagarajan
Prabha Nagareddy
Rana Nagarkatti
Tasuku Nagasawa
Yasuyuki Nagasawa
J. Nagashima
Daisuke Nagata
Jason Nagata
Koichi Nagata
Takamaru Nagata
Takashi Nagata
Yosuke Nagata
Satoshi Nagayama

Bonnie Nagel
Jan Nagel
Maria Nagel
Benjamin Nagengast
Olivier Naggara
Susanna Naggie
Mohsen Naghavi
Chandrashakaharam Nagineni
Michaela Nagl
Elizabeth Nagle
John Nagle
Nicholas Nagle
Arnon Nagler
Markus Nagler
Nicolas Nagloo
Eunice Nago
Marko Nagode
Narihito Nagoshi
Rodney Nagoshi
Avinash Nagpal
Prashant Nagpal
Seema Nagpal
Anne Nagra
Sunitha Nagrath
Attila Nagy
Gábor Nagy
László Nagy
Zsuzsanna Nagy
Kyeongah Nah
Quamrun Nahar
Hareth Nahi
Payam Nahid
Nisha Naicker
Kershney Naidoo
Rayapati Naidu
Shibabrat Naik
Mohammad Naim
Cho Naing
Anil Nair
Anup Nair
Dina Nair
Harish Nair
Jayasree Nair
Manisha Nair
Muraleedharan Nair
Rajeshwari Nair
Saidas Nair
Sandeep Nair
Viji Nair
Viswam Nair
Manfred Nairz
Darren Naish
Robert Naismith
Yoshiro Naito
Yuji Naito
Munetaka Naitoh
Mahdi Najafi
Hamid Najafipour
Laleh Najafizadeh
Mohammad Najafzadeh
Abul Najmi
Hiroaki Naka
Kazumi Nakabayashi
Richard Nakache
Susumu Nakae
Hironori Nakagami
Etsuko Nakagami-Yamaguchi
Kazuo Nakagawa
Naoki Nakagawa
Shinichi Nakagawa
Takayuki Nakagawa
Taku Nakagawa
Teru Nakagawa
Kyoko Nakagawa-Goto
Atsushi Nakagomi
Jin Nakahara
Tadaki Nakahara
Tsutomu Nakahara
Kotaro Nakahishi
Akira Nakai
Kenta Nakai
Yuji Nakai
Peter Nakaji
Arata Nakajima
Ayako Nakajima
Hideko Nakajima
Katsuyuki Nakajima
Kenichiro Nakajima

Ryota Nakajima
Koichi Nakajo
Toshio Nakaki
Shingo Nakamoto
Takamichi Nakamoto
Fabio Nakamura
Futoshi Nakamura
Kazufumi Nakamura
Kazuhiko Nakamura
Kazutoshi Nakamura
Keiko Nakamura
Ken Nakamura
Koshi Nakamura
Masafumi Nakamura
Masato Nakamura
Mashio Nakamura
Michinari Nakamura
Miho Nakamura
Satoko Nakamura
Shuichi Nakamura
Takashi Nakamura
Tomoki Nakamura
Tomoyuki Nakamura
Yuki Nakamura
Akio Nakane
Jun Nakanishi
Kaori Nakanishi
Koki Nakanishi
Yoshinobu Nakanishi
Shin-Ichi Nakano
Shintaro Nakano
Takanari Nakano
Ryoma Nakao
Takashi Nakao
Masahiro Nakaoka
Yoshikazu Nakaoka
Hiroshi Nakase
Junsuke Nakase
Ichiro Nakashima
Kazuki Nakashima
Ran Nakashima
Kazuhiro Nakaso
Noboru Nakata
Yoshio Nakata
Masahiro Nakatochi
Helder Nakaya
Tadashi Nakaya
Kazuya Nakayachi
Hiroshi Nakayama
Jiro Nakayama
Masataka Nakayama
Meiho Nakayama
Tetsuo Nakayama
Ricardo K Nakazato
Takefumi Nakazawa
Yoshinori Nakazawa
Inaam Nakchbandi
Maryam Nakhaei
Annettee Nakimuli
Lydia Nakiyingi
Teofil Nakov
Choi Nakwon
Vamsi J. Nalam
Perihan Nalbant
Luigi Naldi
Grzegorz Nalepa
Atchayaram Nalini
Ma. Jenina Nalipay
Eugene Nalivaiko
Venkatram Nalla
Babi Nallamilli
Prakash Nallathamby
Frédéric Nallet
Romesh Nalliah
Mike Nalls
Boo Nam
Hong Gil Nam
Hyo Suk Nam
Joo-Hyun Nam
Ki Jung Nam
Sangkil Nam
Young-Jae Nam
Yunjun Nam
Judith Namanya
Vigneshwaran Namasivayam
Hamidreza Namazi
Fumihiko Namba
D. Nambiar

Irishi Namboothiri
Atsushi Nambu
Ho Namkoong
Amine Namouchi
Proscovia Namujju
Hongmei Nan
Qun Nan
Yun Nan
Gayani Nanayakkara
Nerissa Nance
Jagdeep Nanchahal
Rahul Nanchal
David Nanchen
Indrajit Nanda
Ranjan Kumar Nanda
Vivek Nanda
Ashis Nandi
Nilashis Nandi
Saikat Nandi
Samiran Nandi
Samit Nandi
Sandip Nandi
Vinay Nandicoori
Madhusudan R. Nandineni
Bindu Nanduri
Sushmita Nandy
Eva Naninck
Deepak Nanjappa
Manjunatha Nanjappa
Som Nanjappa
Meera Nanjundan
Amir Nankali
Alan Nankervis
Derek Nankivil
Loris Nanni
Simona Nanni
Nikos Nanos
Christian Nansen
Jeffrey Nanson
Nanda Nanthakumar
Natasha Nanwa
Hasegawa Naoki
Tanake Naoki
Supawadee Naorungroj
Brooke Napier
Bruce Napier
Richard Napier
Agnieszka Napiórkowska-Krzebietke
Thiago Napoleão
Francesco Napoletano
Donna Jo Napoli
Eleonora Napoli
Joseph Napoli
Valerio Napolioni
Fabio Napolitano
Lena M. Napolitano
L. Christian Napp
Sharon Nappier
Solange Nappo
Philippe Naquet
Nawazish Naqvi
Gokay Nar
Atsushi Nara
Satoshi Naramoto
Jagriti Narang
Pratik Narang
Rahul Narang
Sameer Naranje
Eduardo Naranjo
Hua Naranmandura
Madhusudhanan Narasimhan
Kumar Narasingharao
Angela Narayan
Chandan Narayan
Edward Narayan
Malathi Narayan
Prakash Narayan
Sanjiv Narayan
Satya Narayan
Vikram Narayan
Lakshmi Narayanan
Nandakumar Narayanan
Subramonian Narayanan
Kumaratharan Narayanaswamy
Julija Narbutaite
Dean Nardelli
Maria Pia Nardelli

Maximiliano Nardelli
Flavia Domizia Nardi
Claudio Nardiello
Giovanni Nardini
Davide Nardo
Gabriela Nardoto
Elizabeth Narducci
Anjaparavanda Naren
Ajay Narendra
Nivedita Naresh
Hisashi Narimatsu
Dogan Narinc
Gerard Näring
Vihang Narkar
Michael Narkewicz
Daria Narmoneva
Steven Narod
Ganesan Narsimhan
Masataka Narukawa
Satoshi Narumi
Keiji Naruse
Kenneth Narva
Tarun Narwani
Nikolai Naryshkin
Omer Nas
Kenlo Nasahara
Amir Nasajpour
Carla Nasca
David Nascene
Fabio Nascimbeni
João Nascimento
Maria Sao José Nascimento
Megane Nascimento
Silvia Nascimento
Cristiana Nascimento-Carvalho
Benjamin Nash
David Nash
Robert A. Nash
Sh Nash
Milena Nasi
Faiz Nasim
Mita Nasipuri
Mohammad Nasiriavanaki
Subrata Naskar
Richard Naspro
Fatima Nasrallah
Leila Nasrallah
Sharifa Nasreen
Najib Nassani
Antonio Paulo Nassar Junior
Ahmad Salah Edeen Nassef
Mohamed Nasser
Mohd Nasser
Mona Nasser
Panagiotis T. Nastos
Renato Natal Jorge
David Natale
Emanuele Natale
Vincenzo Natale
Yanina Natanzon
Harihara Natarajan
Karthik V. Natarajan
Purushothaman Natarajan
Sathish Kumar Natarajan
Sivaraman Natarajan
Amar Nath
Aritro Nath
Arun Nath
Neetika Nath
Rahul Nath
Utpal Nath
Derek Nathan
Sheila Nathan
Peter Nathanielsz
Jeremy Nathans
Ruvandhi Nathavitharana
Heiko Nathues
Yasodha Natkunam
Usha Natraj
Tassos Natsakis
Donald Natvig
Roland Nau
Michael Nauck
Victor Nauffal
Ida Naughton
Lisa Naughton
Kelly Naugle
David Naumann

Julia Naumann
Elena Naumova
Zakira Naureen
Fauzia Nausheen
Alessandro Navacchia
Marina Naval-Sanchez
Carryl Navalta
Kannan Navaneetham
José Návar
Kristen Navara
Annalan Navaratnam
Rachel Navarra
Paola Navarrete
Daniela Navarro
David Navarro
Juan-Carlos Navarro
Noemi Navarro
R. Navarro
Raúl Navarro
Vicente Navarro
Victor Navarro
Juan Antonio Navarro Langa
Aura Navarro Quezada
Miguel Navarro-Cia
Fernando Navarro-Garcia
Nora Navarro-Gonzalez
Daniel Navarro-Martinez
Elkin Navarro-Quiroz
Juan Navas
Maria-Cristina Navas
Ana Navas-Acien
Klaus Nave
Mikael Naveau
Adrián Naveda-Rodríguez
Juan Navedo
Muhammad Naveed
Samuele Naviglio
Nima Jafari Navimipour
Mark Navin
Thomas Navin
Amy Navratil
Edita Navratilova
Pavla Navratilova
Isa Navruz
Muhammad Azher Nawaz
Martin Nawrot
Katarzyna Nawrot-Chorabik
Roman Nawroth
Francisco Naya
A. K. Nayak
Chandra Nayak
Jayakar Nayak
M. K. Nayak
Nihar Nayak
Prateep Nayak
Sukanta Nayak
Yogendra Nayak
N. Nayar
Muhammad Ali Nayeem
Rodolfo M. Nayga
Larissa Naylor
Louise Naylor
Saeeda Naz
Petr Nazarov
J. Nazaruk
Ehsan Nazemalhosseini-Mojarad
Mona Nazeri
Nushrat Nazia
Shafagati Nazly
A. S. A. Nazri
Nelson Nazzicari
Pius Nde
Nicaise Ndembi
Mamadou Ousmane Ndiath
Wilfred Ndifon
Chizoma Millicent Ndikom
Alain Ndjiongue
D. Ndjonka
G. Ndrepepa
Sunday Nduka
Andrada Neacsiu
Ben Neal
Brad Neal
Bruce Neal
Joel Neal
Lori Neal
Matthew Neal
Elizabeth Neale

Joshua Nealon
Dimitri Neaux
Naresh Nebhinani
Canan Nebigil
Antonio Nebro
Russell Neches
Sarah Necker
Lucky Nedambale
Katarina Nedbalcova
Anne Nedergaard
Saharnaz Nedjat
Bogusław Nedoszytko
Robert Needham
Rebecca Neel
Anjanasree Neelakandan
Nithya Neelakantan
Sriram Neelamegham
Ian J. Neeland
Ben Neely
Kristina Neely
Melody Neely
Claire Neema
Matthias Nees
Emilie Neez
Christopher Neff
Kristin Neff
James Neffendorf
Irina Neganova
R. K. Negi
Vir Singh Negi
Kazuaki Negishi
Cosimo Neglia
Sonia Negrao
Nicolas Negre
Oscar Negrete
Rodolfo Negri
Lukas Negrin
Miguel Ángel Negrín
Fabio Negrino
S. Negus
Alexandra Negut
Dole Neha
Deborah Neher
Saskia Neher
Arye Nehorai
Volker Nehring
Mark Neider
Daniel Neill
Andrew Neilson
Jonathan Neilson
Julie Neilson
Alexander Neiman
Jayme Neiman
Marco Neira
Jay Neitz
Henrique Neiva
Ayna Nejad
Kari Nejak-Bowen
K. Nekaris
Sergei Nekhai
Fahima Nekka
Andrθ Nel
Karri Neldner
Hilde Nelissen
Koen Nelissen
Thomas Nelius
Ruud Nellen
Wolfgang Nellen
Mark Nellist
Matthew Nelsen
Aimee Nelson
Andrew Nelson
Bryce Nelson
Charles Nelson
David Nelson
Delia Nelson
Eliza Nelson
H. Nelson
Jay Nelson
Julie Nelson
Kara Nelson
Kenrad Nelson
Mark Nelson
Martha Nelson
Matthew Nelson
Peter Nelson
R. I. Nelson
Rebecca Nelson

Sven Nelson
Toben Nelson
William Nelson
Sam Nemanich
Houshang Nemati
Lev Nemchinov
Janos Nemcsik
Raphael Nemenoff
Elizabeta Nemeth
Gabor Nemeth
Johannes Nemeth
Jozsef Nemeth
Balázs Németh
László Németh
Federico Nemmi
Masahito Nemoto
Takahiro Nemoto
Dan Nemrodov
Andrew Nencka
Antonio Nenna
Yan Ning Neo
John Neoptolemos
Martin Neovius
Massimo Nepi
Dmitri Nepogodiev
Johannes Nepp
Karin Neppelenbroek
Marco Neppi
Alain Nepveu
Gabriele Neretti
Christian Neri
Andreas Nerlich
Yussif Nermin
Luis Augusto Nero
Luca Nerva
Jeanne Nervina
W. David Nes
Jerry Nesamony
Sterling Nesbitt
Steven Nesheim
Gabriel Nespoli
Amen Ness
Eivind Ness-Jensen
David Nestel
Felippe Leopoldo Dexheimer
Neto
Onicio Leal Neto
Regueira Neto
Theoden Netoff
Elena Netsi
Jeniel Nett
Daniel Nettle
Nikolaus Netzer
Andreas Neubauer
Johannes Neubauer
Simon Neubauer
Ami Neuberger
Regula Neuenschwander
Gera Neufeld
Rachel Neugarten
Alfred Neugut
Andres Neuhaus
Birger Neuhaus
Klaus Neuhaus
Nina Neuhaus
Oliver Neuhaus
Duncan Neuhauser
Sigrid Neuhauser
Christiane Neuhoff
Daniel Neuhoff
John Neuhoff
Merja A. Neukamm
Sven Neulinger
Aaron Neumann
Dietbert Neumann
Dirk Neumann
Elena Neumann
Gabriele Neumann
Johannes Neumann
Kerstin Neumann
Tim Neumann
Nam Neun Kim
Ramona Neunuebel
Binod Neupane
Nilhari Neupane
Sudan Neupane
Markus Neurath
Andrew Neuwald

Jennifer Neuwald
Edward Neuwelt
Hannes Neuwirt
Pavel Neuzil
Ana Luísa Neves
Fabiana Neves
Frederico Neves
J. Neves
Josiane Neves
Lucimara Neves
M. Manuela Neves
Maria Helena Neves
Ana Gisele Neves-Ferreira
Rémi Neveu
Christopher Neville
Matt Neville
Austin Nevin
Michael Nevin
Thomas Nevins
Yoram Nevo
Sona Nevsimalova
Joshua New
Leslie New
Jason Newbern
Ruth Newberry
David Newbery
Edward Newbigin
Carrie Newbold
Tim Newbold
Kerry Newbrook
Nigel Newbutt
Bi-Min Zhang Newby
Paul Newcombe
Peter Newcombe
Thomas Newe
Christopher Newell
Karl Newell
Annie Newell-Fugate
John Newell-Price
Anna-Kaisa Newheiser
Christina Newhill
Daniel Newhouse
Chris Newland
Craig Newlands
David B. Newman
Diane Newman
Eric Newman
Jonathan Newman
Kira Newman
Louise Newman
Mark Newman
Roger Newman-Norlund
Steven Newmaster
Sean Newsom
Timothy Newsome
Martha Newson
Roger Newson
Simon Newstead
Adam Newton
Andrew Newton
Anna Newton
Duane Newton
Hayley Newton
Irene Newton
Kim Newton
Michael Newton
Nicky J. Newton
Paul Newton
Richard Newton
Ryan Newton
Giles Newton-Howes
Denise Ney
John Ney
Samuel Neymotin
Daria Neyroud
Levent Neyse
Adeline S. L. Ng
Alex Ng
Calvin Ng
Chris Fook Sheng Ng
Daniel Ng
Danny Ng
Edmond Ng
G. Ng
Heok Hee Ng
Jack Ng
Johan Yau Yin Ng
Kwan Ng

Kwun Kei Ng
Lauren Ng
Leo Ng
Lorna Ng
Raymond Ng
Shu-Hang Ng
Sophia Ng
Ting Hui Ng
Tze Hann Ng
Tze Pin Ng
Valerie Ng
Vincent Ng
Wai-Leung Ng
Wan-Fai Ng
Linda Ng Fat
Selia Ng'Anjo Phiri
H. W. Ngan
Nobubelo Ngandu
Tiia Ngandu
Umakanta Ngangkham
Matilda Ngarina
Billy Ngasala
Joanne Ngeow
Nhung Nghiem
Peter Nghiem
Phioanh Nghiemphu
Van Ngo
Renay Ngobeni
Calistus Ngonghala
Philip Mutinda Ngumbi
John Ngunjiri
Amanda Nguyen
Annalee Nguyen
Dao Nguyen
Duc Cong Hiep Nguyen
Duy Nguyen
Hoang Nguyen
Hong-Son Nguyen
Hue Nguyen
Hung Nguyen
Isabel Nguyen
Jacques Nguyen
Jean Marie Nguyen
Khoa Nguyen
M. Hong Nguyen
Margaret Nguyen
Michael Nguyen
Nam Nguyen
Nhung Nguyen
Patricia Nguyen
Phan Nguyen
Phuong Nguyen
Quang Nguyen
Ruby Nguyen
Tam Nguyen
Thai-Son Nguyen
Thanh Nguyen
Thao Nguyen
Thi Bich Ngoc Nguyen
Thi Thu Hoai Nguyen
Thi-Phuong-Lan Nguyen
Tho Nguyen
Thuy Nguyen
Trang Quynh Nguyen
Tuan Nguyen
Yann Nguyen
Tuan Nguyen Minh
Aquino Nhantumbo
Joo Young Nho
Kwangsik Nho
Hoang Nhung
Andy Ni
Chaoying Ni
Dong Ni
Hong-Min Ni
Junkang Ni
Rui Ni
Ting Ni
Ya Ni
Yicheng Ni
Zhaohui Ni
Zhen Ni
Zhongfu Ni
Fabien Niama
Naz Niamul Islam
Javed Niazi
Muaz Niazi
Luigi Nibali

Emanuele Nicastri
Giampaolo Niccoli
Chris Nice
Helen Nichol
Peter Nichol
Ben Nicholas
Frank Nicholas
Richard Nicholas
Rob Nicholas
Frank Nichols
Ruthann Nichols
Barbara Nicholson
Jay Nicholson
Lindsay B. Nicholson
Tracy Nicholson
Wayne Nicholson
Georg Nickenig
David Nickerson
Kevin Nickerson
Stuart Nicklin
Thomas Nickolas
Kerry Nickols
Daniele Nico
Barbara Nicol
Caroline Nicol
Fabrício Nicola
Lamberti Nicola
Massimo Nicola
Saleem Nicola
Argyris Nicolaidis
Valentina Nicolardi
Alice Nicolas
Gaël Nicolas
Guillaume Nicolas
Olga Nicolas
Pierre Nicolas
Violaine Nicolas
Belinda Nicolau
Roberto Nicolete
Bruna Nicoletto
Sara Nicoli
Silvia Nicolis
Lindsay Nicolle
F. C. Nicolleau
Massimo Nicolo
Andrea Nicolò
Philippa Nicolson
Teresa Nicolson
Roberto Nicosia
Nicole Nicotera
Franck Nicoud
Aiqing Nie
Jian-Yun Nie
Jingxin Nie
Junsheng Nie
Qian Nie
Qinghua Nie
Shao-Ping Nie
Yaohui Nie
Yong-Wei Nie
Yunqiang Nie
Chris Niebuhr
Ewa Niechwiej-Szwedo
Paula M. Niedenthal
Ellen Niederberger
Tom Niederkrotenthaler
Karin Niedermann
Andreas Niedermayr
Matthias Niedrig
Be'eri Niego
James Nieh
Björn Niehaves
Diederick Niehorster
Manel Niell
Alan Nielsen
Anders Nielsen
Birgitte Nielsen
Brent Nielsen
C. Nielsen
Claus Nielsen
Clayton Nielsen
Finn Arup Nielsen
Forrest Nielsen
Heber Nielsen
Jeppe Nielsen
Line Nielsen
Liza Nielsen
Nathan Nielsen

Peter Nielsen
Sarah Nielsen
Shaun Nielsen
Susanne Nielsen
Suzanne Nielsen
Tenna Nielsen
X. Nielsen
Olav Nielssen
Marvin Nieman
S. Niemann
Andreas Niemeier
Matthias Niemeier
Jari Niemelä
Frank Niemeyer
Matthew Niemiller
Anna-Liisa Nieminen
Tara Niendam
Sandra Niendorf
Marzena Nieroda
Uwe Niesner
Jan Niess
Heike Niessner
Gonzalo Del Monte Nieto
Marcelo Nieto
Nathan Nieto
Rosa Nieto
Alice Nieuwboer
Max Nieuwdorp
Arne Nieuwenhuys
Pythia Nieuwkerk
Rienk Nieuwland
Madeline Nieves-Cintron
Piotr Niewinski
Theo Niewold
Vishal Nigam
Tyler Nighbor
Prashant Nighot
Chingiz Nigmatullin
Cecilia Nigro
Sagar Nigwekar
Kanako Niimori-Kita
Annabel Nijhof
Monique Nijhuis
Vincent Nijman
W. B. Wan Nik
Nik Fakhuruddin Nik Hassan
Hiroshi Nikaido
T. Nikam
Spyros Nikas
Christoph Nikendei
Beatrice Nikiema
Yuri Nikiforov
Mehdi Nikkhah
Martin Niklas
Johan Niklasson
Viacheslav Nikolaev
Pantelis Nikolaidis
Michael Nikolaou
Vasileios Nikolaou
Molly Nikolas
Susanne Nikolaus
Dimitrije Nikolic
Mikeljon Nikolich
D. Nikolla
Efthymios Nikolopoulos
Katerina Nikolovska
Alexander Nikonenko
Sayeh Nikpay
Kjell Nikus
Shivraj Nile
Meredith Niles
Eric Nilles
Yael Nillni
Trude Nilsen
Gustav Nilsonne
Daniel Nilsson
Henrik Nilsson
Lars Nilsson
Lennart Nilsson
Markus Nilsson
Pier Luigi Nimis
Kim Nimon
Johannes Nimpf
Nicolas Nin
Huan Ning
Jianting Ning
Lipeng Ning
Shunbin Ning

Xia Ning
Xinghai Ning
Yuese Ning
Zhangyong Ning
Zhaolong Ning
Elaine Hoi Ning Ng
Phillip Ningst
Shira Ninio
Cinzia Niolu
Shlomo Nir
Jhala Nirag
Rakesh Niraj
Saroj Niraula
Madunil Niriella
Fabio Niro
Teja Srinivas Nirujogi
Alasdair Nisbet
Elizabeth Nisbet
Nicholas Nisbett
Akihiro Nishi
Hiroshi Nishi
Makiko Nishi
Shawn Nishi
Shinichi Nishi
Atsushi Nishida
Motohiro Nishida
Koji Nishifuji
Akihiro Nishiguchi
Reiko Nishihara
K. Nishijima
Kazutoshi Nishijima
Takeshi Nishijima
Takahito Nishikata
Jun Nishikawa
Saori Nishikawa
Yoshikazu Nishikawa
Toshio Nishikimi
Norihiro Nishimoto
Hitoshi Nishimura
Norihisa Nishimura
Tomoyasu Nishimura
Adrienne Nishina
Hiroshi Nishina
Takashi Nishina
Ichizo Nishino
Kunihiko Nishino
Mizuki Nishino
Tatsuya Nishino
Hisanori Nishio
Junko Nishio
Shinta Nishioka
Kiyoji Nishiwaki
Akiko Nishiyama
Chiharu Nishiyama
Keita Nishiyama
Milton Nishiyama
Osamu Nishiyama
Ryota Nishiyori
Masahiko Nishizaki
Vesa Niskanen
Marina Nisnevitch
Enzo Nisoli
Omar Niss
Xavier Nissan
Robert Nissen
John Nitiss
Chikako Nito
Aida Nitsch
Andreas Nitsche
Christoph Nitsche
Atsumi Nitta
Takeshi Nitta
Bjoern Nitzsche
Björn Nitzsche
Franziska Nitzsche
Ben Niu
Canfang Niu
Jianjun Niu
Jingwen Niu
Junqi Niu
Lu Niu
Mutian Niu
Shan-Ce Niu
Suyan Niu
Wenxin Niu
Xiaoping Niu
Yifeng Niu
Markus Nivala

Ryusuke Niwa
Takayoshi Niwa
Justin Nix
Christian Nixon
Kim Nixon
Stephanie Nixon
Christopher Niyibizi
Kostas Nizamis
Sheikh Nizamuddin
Dean Nizetic
Antonio Nizza
Janet Njelesani
Emmanuel Njeuhmeli
Dolores Njoku
Anne Esther Njom Nlend
Richard Njouom
Céline Nkenfou
Jaqcueline Nkhoma
Esther Nkuipou Kenfack
Marriott Nliwasa
Jerome Noailly
Luca Nobile
Flavio Nobili
Marcin Nobis
Ferdinand Noble
Kimberly Noble
Michael Noble
William Noble
Ligia Nobre
Marimes Nobre-dos-Santos
Annalura Nocentini
Arcangelo Nocera
Joe Nocera
Kotaro Nochioka
Viviana Nociti
Nora Nock
Kazuki Noda
Yoshihiro Noda
Yukihiro Noda
Francesco Noè
Agnes Noel
Alexandra Noel
Jean-Paul Noel
Marie-Pascale Noel
Nora Noel
François Noël
Stephen G. Noffsinger
Haruko Noguchi
Ko Noguchi
Masayuki Noguchi
Dalia Nogueira
Denis Nogueira
Leandro Alberto Calazans Nogueira
Miguel Nogueira
Miodeli Nogueira Júnior
Rosa Noguera
Suegene Noh
Sung Hoon Noh
Sung Noh
Susan Noh
Percy Nohama
Atsushi Nohara
Anja Nohe
Sansanee Noisakran
Ali Nokhodchi
John Nolan
Michael Nolan
Vikki Nolan
Maria Nolano
Christopher Nold
Patrick Nolen
Mirja Nolff
Michael Noll-Hussong
Rick Nolte
Kensuke Noma
Cristina Nombela
Jason Nomi
Tetsuo Nomiyama
Hiroshi Nomura
Michio Nomura
Motoo Nomura
Takahito Nomura
Tsutomu Nomura
Amy Non
Peter Nonacs
Michel Nonent
Yibing Nong

Upendra Nongthomba
Alberto Nonis
Larisa Nonn
Dan Nonneman
Hiro Nonogaki
Intawat Nookaew
Jason Noon
Vanessa Noonan
Michael Noone
Peadar Noone
Chadanat Noonin
Ramadhani Noor
Mustapha M. Noordin
M. Noordzij
Hamid Reza Noori
Nadia Noori
Amir Noormohammadi
Fernando Norambuena
Lorena Norambuena
Dan Norbäck
Helin Norberg
Anthony Norcia
Brenda Norcross
Marc Norcross
Peter Nordbeck
Martin Nordberg
Maria Nordfang
Verena Nordhoff
Greg Nordin
Norshariza Nordin
Malin Nording
Rebecca Nordquist
Anne Nordrehaug
Nikolai Nordsborg
Marie Nordstrom
Anders Nordström
Annika Noreen
Martin Norgaard
Mette Nørgaard
Andrew Norgan
Fernando Noriega
Elisabeth Norin
Torsten Norlander
Emma Norling
Åsa Norman
Edward Norman
J. Farley Norman
Keri Norman
Paul Norman
Philippe Normand
Henrique Noronha
Christoph Norrenbrock
Seth Norrholm
Marianna Norring
Andrew Norris
Darren Norris
Lucy Angela Norris
Pauline Norris
Philip Norris
Ivan Norscia
Niels Nørskov-Lauritsen
Alicia North
Robert Northington
William Northrop
Joseph Northrup
Gavin Norton
George Norton
Jeffrey Norton
Kerri-Ann Norton
Pamela Norton
Raymond Norton
Samuel Norton
Terry Norton
Justice Norvienyeku
Therese T. Nøs
Joshua Nosanchuk
Chiara Nosarti
Katsuhiko Nosho
Katharina Noske
Richard Noske
Sergei Noskov
Tahereh Nosratzehi
Andrew Noss
William Notcutt
Jani Nöthling
Eduardo Notivol
Davide Noto
Jennifer Noto

Ill-Sup Nou
A. Noufaily
Antoine Nougairède
Eduardo Nouhra
Anastasios Noulas
M. Nouno
Nazila Nouraee
Mohammad-Zaman Nouri
Amin Nourmohammadi
Celine Nourrisson
Kirill Nourski
Camilla Novaglio
Andrew R. Novak
Klemen Novak
Mario Novak
Katarina Novakovic
Matteo Novara
Marcia Novaretti
Susana Novella
Enrico Novelli
Giorgio Novelli
Mara Novero
Marjana Novič
Richard Novick
Lev Novikov
Mikhail Novikov
Diana Novikova
Mariia Novikova
M. Lelinneth (Len) Novilla
Chad Novince
Juliana Novo
Shenia Novo
Ana Novoa
Isabel Novo-Cortí
Alexey A. Novoselov
Elena Novoselova
Dario Novoselovic
Josef Novotny
Tomas Novotny
Artem Novozhilov
Eva Nowack
Glen Nowak
Stephen Nowak
Thorsten Nowak
Witold Nowak
Aneta Nowakiewicz
Andrzej Nowakowski
Piotr Nowakowski
Kristen Nowell
Bogdan Nowicki
Piotr Nowicki
Cindy Nowinski
Rj Nowling
Krzysztof Nowosielski
Miho Nozaki
Tomoyoshi Nozaki
Sylvie Nozaradan
Ala Nozari
Silvia Nozza
Sabin Nsanzimana
Saad Nseir
Mathieu Nsenga Kumwimba
Léon Nshimyumukiza
Elaine Nsoesie
George Ntaios
Nikoletta Ntalli
Abel Ntambue
Fani Ntana
Richard Nuccitelli
Rolf Nuesing
Alan Nugent
Allison Nugent
Kenneth Nugent
Michael Nugent
Nicole Nugent
Maggy Nugues
Clair Null
Kazuyuki Numakura
Shusuke Numata
Elizabeth Nunamaker
Bruno Nunes
Jean Nunes Nunes
Laura Nunes
Marcio Nunes
Márcio Nunes
Paulo Nunes
Virginia Nunes

Claudia Nunes Duarte dos Santos
Adriano Nunes-Nesi
Cassandra Nunez
Silvia Nunez
Carlos Nuñez
Marco T. Núñez
Juan Núñez-Farfán
Karen A. Nunley
Clifton Nunnally
Ana Nuno
Maxim Nuraliev
Paquita Nurden
Leena Nurminen
Charlotte Nusman
Lucas Nussbaum
Robert Nussbaum
Thomas Nussbaumer
Roel Nusse
Ruth Nussinov
Michael Nute
Iman Nuwayhid
Genoveffa Nuzzo
Jennifer Nuzzo
Regina Nuzzo
Joseph Nwadiuko
Anthea Nwandu
Bright Nwaru
Julius Nwobegahay
Trevor Nyakudya
Hanson Nyantakyi-Frimpong
Tibor Nyari
Samuel Nyarko
Lars Nybo
Anne-Marie Nybo Andersen
Hussain Nyeem
Akos Nyerges
Marianne Nygaard
Henrik Nygård
Annette Nygårdh
Brendan Nyhan
Erika Nyhus
Philip Nyhus
Sylvia Nyilas
Lot Jata Nyirenda
Makandwe Nyirenda
Ivan Nyklicek
Karin Nylander
Susanne Nylen
Stephan Nylinder
Jeffry Nyman
Lau Nyok Sean
Betserai Nyoka
Paul Nyquist
Fredrik Nystrom
Alexander Nyström
Justine Nzweundji
