# Supplementary material for: PLOS ONE 2017 Reviewer and Editorial Board Thank You
Source: PLoS One. 2018 Mar 15;13(3):e0194158. doi: 10.1371/journal.pone.0194158 (PMC5854357; doi:10.1371/journal.pone.0194158)
Supplement: S4 Reviewer List — Alphabetical list of 2017 PLOS ONE peer reviewers (O-S). (PDF) [file pone.0194158.s006.pdf]

PLOS ONE would like to thank all those who reviewed on behalf of the journal in 2017:

Daibhid O Maoileidigh
Clare O’Callaghan
Grant O’Connell
Anna O’Connor
Terry O’Connor
Jim O’Doherty
Brigid O’Donnell
John P. O’Hara
Gregory D. O’Mullan
Declan O’Regan
Brian Oakley
Ingrid Oakley-Girvan
Cristian Oancea
Elena Oancea
Megan Oaten
Gabriela Oates
Björn Oback
Kehinde Obamiro
M. O'Banion
Stephen Obaro
Lina Obeid
Rima Obeid
Greg O'Beirne
André Obenaus
Anita Oberbauer
Birgitta Öberg
Ludwig Oberkircher
Eva Oberle
Lindsay Oberman
Snehlata Oberoi
Sharon Oberstein
Philippe Obert
Timm Oberwahrenbrock
Yoshitsugu Obi
Walter Obiero
Satoshi Obika
Christian Obirikorang
Elia Obis
Concepción Obón
Augustine Obour
Dusanka Obradovic
David O'Brart
Aleksandra Obrępalska-Stęplowska
Charles O'Brien
Dan O'Brien
Kimberly O'Brien
Mary O'Brien
Meagan O'Brien
Michael O'Brien
Patrick O'Brien
Stephen O'Brien
Todd O'Brien
Bill O'Brien-Penney
Neil O'Brien-Simpson
Tomas Obsil
Ekwaro Obuku
Eva Oburger
Nuh Ocak
Konrad Ocalewicz
Claire O'Callaghan
David O'Callaghan
Michael O'Callaghan
Anahi Ocampo-Melgar
Alberto Ocaña
Victor Ocaña
Delvac Oceandy
Rhoune Ochako
Oche Oche
Daisuke Ochi
Hiroki Ochi
Josiah Ochieng
Washingtone Ochieng
Maria Teresa Ochoa
Francisco Ochoa-Corona
Miroslaw Ochodek
Emmanuel Ochola
Rachel Ochola

Isabella Ochola-Oyier
Michael Ochsenkuehn
Torsten Ochsenreiter
Urs Ochsner
Ira Ockene
Sebastian Ocklenburg
Samuel Ocko
Craig O'Connell
Darren O'Connell
Dennis O'Connell
Grace O'Connell
Mary Beth O'Connell
Megan O'Connell
Michael O'Connell
Tamsin O'Connell
Christine O'Connor
Daniel O'Connor
Devin O'Connor
Ellen O'Connor
J. Patrick O'Connor
Kathleen O'Connor
Kevin O'Connor
Michael O'Connor
Pat O'Connor
Peter O'Connor
Richard O'Connor
Roberta O'Connor
Sarah O'Connor
Rachel Oconor
Greg O'Corry-Crowe
Ismail Ocsoy
Simon Oczkowski
Ryo Oda
Sedat Odabas
Hatice Odacı
Marina Odalovic
Francesco Oddone
Maria Cristina Oddone
Hirotaka Ode
Shirley O'Dea
Brian Odegaard
Oreofe Odejide
Adam Odell
Edward Odell
Luke Odell
Nicole Odell
Thomas O'Dell
Katja Odening
Patrizio Odetti
Kate Odgers-Jewell
Emmanuel Odic
Maurice Odiere
Monica Odlare
Daniel C. M. O'Doherty
George O'Doherty
James Odom
Jan Odom-Forren
Angela Odoms-Young
Anna Odone
Brian O'Donnell
James O'Donnell
Kerry O'Donnell
Lauren O'Donnell
Matthew O'Donnell
Sean O'Donnell
Anthony O'Donoghue
Stefani O'Donoghue
Gary O'Donovan
Misari Oe
Walter Oechel
Karin Oechsle
Stefan Oehlers
Ina Oehme
Edwin Oei
Nicole Oei
Thomas Oelgeschläger
Soner Oener-Sieben
Dick Oepkes
Lauge Oestergaard
Arne Oetjen
William Oetting
Orit Oettinger-Barak
A. Oevermann
Mehmet Oezkur
Dietmar Offenhuber
Stefan Offermanns
Bernard Offmann
Rivka Ofir

Nana Ofori-Opoku
Emmanuel Oga
Toru Oga
Masao Ogaki
Oleg Ogarkov
Anne O'Garra
Sadahisa Ogasawara
Kazuhiro Ogata
Norichika Ogata
Aiko Ogawa
Hiroto Ogawa
Keiko Ogawa
Ryohei Ogawa
Yoshihiro Ogawa
Shinya Ogaya
Adamu Ogbe
Felix Ogbo
Brian Ogbonna
Nicholas Ogden
Emmanuel Oger
Frédérik Oger
John Oghalai
Maria Ogielska
Naomichi Ogihara
Akiko Ogino
Keiki Ogino
Shuji Ogino
Michael Oglesbee
Billy Oglesby
Amanda Oglesby-Sherrouse
Juliana Ogliari
Mylène Ogliastro
Shigehiko Ogoh
Jimmy O'Gorman
Kerry-Ann O'Grady
Sven Ove Ögren
Besim Ogretmen
Rajna Ogrin
Ana Ogueta-Alday
Mary Oguike
Ezra Ogundare
Benson Ogunjimi
Samuel T. Ogunjo
Olubunmi Ogunrin
Olufemi Ogunrinde
Oluwaseun Ogunro
Atsushi Ogura
Yuji Ogura
Ezgi Ogutcen
Ipek Oguz
Ozgur Oguz
Tomson Ogwang
Chang Sik Oh
Edwin Oh
Hwamee Oh
Hyungseon Oh
Hyunsung Oh
Jaehoon Oh
Jin-Kyoung Oh
Jong-Ryool Oh
Joonpyo Oh
Jung Hun Oh
Junghwan Oh
Man Ho Oh
Minje Oh
Myoung-Don Oh
S. Paul Oh
Se Won Oh
Stephen Oh
Damien O'Halloran
Ken O'Halloran
Michael O'Hara
Nathan O'Hara
Beth O'Hare
Louise O'Hare
Finbarr O'Harte
Jun Ohashi
Koji Ohashi
Fidelia Ohemeng
Ryoma Ohi
Kousaku Ohinata
Mitsuru Ohishi
Yoshiyuki Ohkawa
Nobuhiro Ohkohchi
Chikahiro Ohkubo
Shinji Ohkubo
Kevin Ohlemiller
Daniela Ohlendorf

Uwe Ohler
Henrik Ohlsson
Jason Ohlstein
Robin Ohm
Christian Ohmann
Michel Ohmer
Tsukasa Ohmori
Haley Ohms
Koichiro Ohmura
Haruya Ohno
Kinji Ohno
Seiko Ohno
Shinsuke Ohnuki
Takahiko Oho
Yuki Ohsaki
Ikuroh Ohsawa
Satoru Ohshima
Shinichiro Ohshimo
Keisuke Ohta
Yasutaka Ohta
Yukari Ohta
Toshiyuki Ohtani
Ryo Ohtomo
Toshio Ohtsubo
Susumu Ohtsuka
Sumio Ohtsuki
Colm O'Huigin
Eric Ohuma
Susumu Ohya
Yusuke Ohya
Takahiro Ohyama
Tomoko Ohyama
Cintia Oi
Akira Oikawa
Yuichi Oike
Jaana Oikkonen
Georgios Oikonomou
Hisashi Oishi
Timo Ojanen
Elena Ojea
Joaquin Ojeda
Chet Ojha
Priyanka Ojha
Shalini Ojha
Tatsuo Oji
Christiana Oji-Mmuo
Odelu Ojjela
Dike Ojji
Akira Oka
Shinichi Oka
Takehiro Okabayashi
Yoshihiro Okabe
Yoshinobu Okabe
Norihiro Okada
Shigeru Okada
Shinji Okada
Takashi Okada
Tomohisa Okada
Tomoo Okada
Yasushi Okada
Yohei Okada
Yoshiyuki Okada
Yuko Okado
Philip Okafor
Yuko Okamatsu-Ogura
Suguru Okami
Akira Okamoto
Daniel Okamoto
Hajime Okamoto
Kenichi Okamoto
Mamoru Okamoto
R. Okamoto
Takashi Okamoto
Yoshitaka Okamoto
Hisayoshi Okamura
Nobuyuki Okamura
Yukiyasu Okamura
Yasmina Okan
Kehinde Okanlawon
Sumiyo Okawa
Sezer Okay
Eiichiro Okazaki
Kazuichi Okazaki
Tomoki Okazaki
Toshiro Okazaki
Victor Okazaki
Hitoshi Okazawa
Graeme O'Keefe

Michael O'Keefe
Sean O'Keefe
Iruka Okeke
Lance Okeke
Richard O'Kennedy
Hellen A. Oketch-Rabah
Thomas Okey
Mariam Okhovat
Shinji Oki
Yasuhiro Oki
Kyoji Okita
Klaus Okkenhaug
Kees Okkersen
Marina Okoshi
Evaezi Okpokoro
Lauri Oksama
Atte Oksanen
Sari Oksanen
Tuula Oksanen
Ole Andreas Økstad
Kenji Oku
Shujiro Okuda
Noriyuki Okudaira
Yoshinaga Okugawa
Sakiko Okumoto
Fredros Okumu
Kenji Okumura
Ko Okumura
Takahiro Okumura
Michele Okun
Christopher Okunseri
Takafumi Okura
Tsuyoshi Okura
Olaoluwa Okusaga
Masashi Okushima
Nduka Okwose
Pınar Okyay
Ramazan Okyay
Celia Olabarria
Anja Olafsen
Adeniyi Olagunju
Andrew Olagunju
Iñigo Olalde
Lynne Oland
Lydia Olander
T. Olaniyan
Funmilola Olaolorun
José Ramón Olarieta
Olalekan Olarogba
Liset Olarte
Dumitru N. Olaru
Tajudeen O. Olasupo
Kelli O'Laughlin
Adedamola Olayanju
Robert Olberg
Eckehard Olbrich
Natasha Olby
Monika Ołdak
Lukasz Oldakowski
Johan Oldekop
Bas Oldenburg
Mark Oldham
Ben Oldroyd
Alvaro Oleaga
Francisco Olea-Popelka
Joann O'Leary
Mary O'Leary
Stephen O'Leary
Thomas O'Leary
Natalia Oleinik
Jason Olejarz
Malgorzata Olejnik
Obinna O. Oleribe
Niels Olesen
Balaji Olety
Elisabeth Olhager
Philipp Olias
Renske Olie
Pierpaolo Olimpieri
Jill Olin
Michael Olin
Tom Olino
Ryszard Olinski
Antonio Olita
Francesco Oliva
Maria Luiza Oliva
Nancy Oliva
Salvatore Oliva

Juan Oliva-Moreno
Pilar Olivar
Alvaro Olivares
Mario Olivari
Aires Oliva-Teles
Sebastien Olive
Alaide Oliveira
Ana Laura Oliveira
Andreia Oliveira
Claudio Oliveira
Cristieli Oliveira
Eugênio Oliveira
Giancarlo Conde Xavier Oliveira
Gustavo H. C. Oliveira
Gustavo P. Oliveira
Jhones Oliveira
Joaquim Miguel Oliveira
Julio Oliveira
Larissa Oliveira
Marcela Oliveira
Maria Leonor Oliveira
Matheus Oliveira
Paulo Oliveira
Raquel Oliveira
Rodrigo Oliveira
Rui Oliveira
Serafim Oliveira
Tiago Oliveira
Trícia Maria Oliveira
Ubirajara Oliveira
Ursula Oliveira
Valéria Oliveira
Vanessa Oliveira
Ana Oliveira Dos Santos
Amparo Oliver
Anthony Oliver
Antonio Oliver
Bonamy Oliver
Constance Oliver
Joseph Oliver
Kathryn Oliver
Stefan Oliver
Clare Oliver-Williams
Eleonora Olivetta
Christoph Olivier
Stephen Olivier
Anna Olivieri
Christopher Olivola
Jean-Marc Olivot
Ike Olivotto
Dan Olk
Maria Olkkonen
Vesa Olkkonen
Sandra Olkowski
Nathalie Ollat
Nicholas Ollberding
Margit Olle
B. O. Olley
John Ollinger
Cheryl Olman
Gabriela Olmedo
Rocío Olmedo-Requena
William Olmstadt
Kenneth Olofsson
Per Olofsson
Valerie O'Loughlin
Samuel Olowookere
Ingar Olsen
Jerome Olsen
Jorn Olsen
Lasse Mork Olsen
Lise Olsen
Ole Olsen
Brian Olshansky
S. Jay Olshansky
Richard Olshen
Bradley Olson
Christine Olson
Erik Olson
John Olson
Jonathan Olson
Ken Olson
Kent Olson
Lucretia Olson
Michael Olson
Nels Olson
Steven A. Olson
Victoria Olson

Zachary Olson
Johan Olsrud
Olof Olsson
Sanna Olsson
Shannon Olsson
Magdalena Olszewska
Adam Olszewski
Horatiu Olteanu
Ana-Maria Olteteanu
Allard Olthof
Scott Oltman
Javier Oltra-Cucarella
Olushayo Olu
Olugbemi Olukolade
Peter Olumese
Aria Olumi
Olutayo Aloba Olutayo Aloba
Olusegun Oluwole
Christine Olver
Rene Olvera
Matthias Olzmann
Yosuke Omae
Siobhain O'Mahony
Paul O'maille
James O'Malley
Amr Omar
Benzakour Omar
Marhanis Salihah Omar
Shaheed Omar
Syed Haris Omar
Megan O'Mara
Perera Omaththage
Tsutomu Omatsu
Willem Ombelet
Michael Ombrello
Roald Omdal
Sidney Omelon
Fred Omengo
Charity Omenka
Jeff Omens
Giovanni Ometto
Diana Omigie
Bernard Omolo
George Omondi
Keiko Omori
Abdou Omorou
Hiroshi Omote
Oriol Oms
Gemma Oms-Oliu
Geoffrey Omuse
Stephen On
Tomokatsu Onaga
Nobuyuki Onai
Mark Onaitis
Gladys Onambele-Pearson
Olakunle Onaolapo
Zafer Onaran
Altan Onat
Mehmet Yekta Oncel
Andrew Onderdonk
Fikret Ondes
Pascale Ondoa
Frank Ondrey
Wanda O'Neal
Birgul Onec
Eric O'neill
Hester O'Neill
Jennifer O'Neill
Katherine O'Neill
Malcolm O'Neill
Stephen O'Neill
F. Cumhur Oner
Çağrı Öner
Albert Ong
Chin Siang Ong
Choon Kiat Ong
Jason Ong
Jia Hoong Ong
Lee-Ling Ong
Peter Ong
Song Ong
Linnet Ongeri
Julina Ongkasuwan
Kentaro Onishi
Masayuki Onishi
Dilek Önkal
Michael Onken
Koh Ono

Sachiko Ono
Shoko Ono
Takashi Ono
Yumie Ono
Yusuke Ono
Andrea Onofri
Francesco Onorati
Paolo Onori
Yoshihiro Onouchi
Kenji Onoue
Ichiro Onoyama
Sheila Ons
Mark Onslow
Suneel Onteru
Luiz Onuchic
Temitope Onuminya
Mehmet Ruhi Onur
Monica Onyango
Win Min Oo
Kenneth Ooi
Peck Toung Ooi
Susumu Ookawara
Antonius Oomens
Ryoko Oono
Katariina Öörni
Chris Oostenbrink
Benjamin Oosterhoff
Egbert Oosterwijk
Ronald Oosting
S. F. Oosting
Lulla Opatowski
Juan Opazo
Ghislain Opdenakker
Don Operario
Joseph Opferman
Lars Opgenoorth
George Opie
George Opit
Esther Opmeer
Mark Oppe
Steffen Oppel
Daniel Oppenheimer
Federico Oppenheimer
Frederik Opperdoes
Henry Oppermann
Brenda Oppert
Joseph Oppong
Samuel Oppong
Kwaku Oppong Asante
Mateusz Opyrchal
Tamer Oraby
Zeki Oralhan
Peter Orazem
Kirill Mark Orci
Matthew Orde
S. Orde
Armando Ordoñez
Enrique Orduna-Malea
Monica Ordway
Ayokanmi Ore
Mislav Oreb
S. M. Oregan
Ciara O'Reilly
Kathleen O'Reilly
Michael O'Reilly
Raphael Orélis-Ribeiro
Ana Maria Orellana
Juan Orellana
Laura Orellana
Elena Orellano
Mark Oremus
Eyal Oren
Evan Orenstein
Alberto Orfao
Chris Organ
Michael Orger
Alberto Orgiazzi
Dennis Orgill
Jennifer Orgill
Guido Orgs
Alessandro Ori
Arianne Pontes Oria
Reinaldo Oriá
Eniyou Oriero
Francesco Origgi
Kazumasa Orihashi
Carlos Orihuela
Pedro Orihuela

Michele Orini
Ieda Orioli
Antonio Orlacchio
Augusto Orlandi
Rosaria Orlandi
Jéssica Orlandin
Giuseppe Orlando
Stefano Orlando
Fanny Orlhac
Marek Orlik
David Orlikowski
David Orlov
Sergei Orlov
Sergey Orlov
Irene Orlow
Eric Orman
Zeynep Orman
Mark Orme
Elena Ormeño
Michael Ormsbee
Christopher Ormsby
Michelle Ormseth
Ana Claudia Ornelas
Juan Francisco Ornelas
David Ornelles
Raffaele Ornello
Heather Orom
Maite Oronoz
Daniel Oros
Ryan Orosco
Ferenc Orosz
Aubrie O'Rourke
Geraldine O'Rourke
Esther Orozco
Victor Orozco
Domingo Orozco-Beltran
Quetzalcóatl Orozco-Ramírez
Christina Orphanidou
Jacob Lund Orquin
Mark Orr
Noreen Orr
Catherine Orrell
Daniel Orringer
Christina Orru
Amy Orsborn
Andrea Orsey
Giovanna Orsini
Kapuy Orsolya
Frank Orson
Dag Ørstavik
Brian Ort
Donald Ort
Daniel Ortega
David Ortega
Dolors Ortega
Enrique Ortega
Felipe Ortega
Jorge Ortega
M. Ortega
Sofia Ortega
Victor Enrique Ortega
Victor Ortega
Alejandro Ortega-Beltran
Elena Ortega-Campos
Carmen Ortega-Rosas
Carmen Ortego
Jason Ortegren
Arturo Ortin-Martinez
Andres Ortiz
Jorge Ortiz
Jose Ortiz
Michael Ortiz
Rodomiro Ortiz
Rudy Ortiz
Vilma Enid Ortiz
Jaime Ortiz Viedma
Lourdes Ortiz-Fernandez
César Ortiz-Moya
Raúl Ortiz-Pulido
Erik Ortmann
David Orton
Richard Orton
Maria Grazia Ortore
Juan Orueta
Akintunde Orunmuyi
Raoul Orvieto
Dan Ory
Daniel Ory

Jeramia Ory
John Oryema
Diego Orzaez
Arkadiusz Orzechowski
Slawomir Orzechowski
Francesco Orzi
Naoki Osada
Naomi Osakabe
Sheha Osam
Opeyemi Osanaiye
Masayuki Osawa
Takeshi Osawa
Takuya Osawa
Olivia Osborn
Andrew Osborne
Juliet Osborne
Lisa Osborne
Molly Osborne
Richard Osborne
Anne Osbourn
Andreas Oschlies
Igor Oscorbin
Padraig O'Seaghdha
Patrick Osei-Owusu
Ivan Oseledets
Saskia J. M. Osendarp
Cecilia Osera
Cristian Osgnach
Ryan O'Shaughnessy
Helen O'Shea
Nir Osherov
Kiyoko Oshima
Atsushi Oshio
Hitoshi Oshitani
Faith Osier
Hans-Jürgen Osigus
Av Osipov
Thordur Oskarsson
Carole Oskeritzian
Michael Osland
Augustine Osman
Mohamed Osman
Ossama Osman
Pavel Osmancik
Stephen Osmani
Clive Osmond
Antonio Osorio
E. Osorio
Fernando Osorio
Johan Osorio
Hugo Osório
Maria Ospina
Alice Ossoli
Piet Ost
Sarah Ostadabbas
Bohdan Ostash
Anna-Lena Ostberg
Ylva Ostby
Bertram Ostendorf
Julien Oster
Matthew Oster
Henrik Osterblom
Karen Østergaard
Frank O. Ostermann
Palle Osther
Jakob Ostien
Nancy Ostiguy
Braxton Osting
Sean Ostlund
Pedro Ostoa-Saloma
Srdjan Ostojic
Elaine Ostrander
Harry Ostrer
Lisa Ostrin
Rachel Ostroff
David Ostrov
Irina Ostrovnaya
Michael Ostrowsky
Gwen O'Sullivan
James O'Sullivan
Orla O'Sullivan
Patricia O'Sullivan
Rory O'Sullivan
Teresa O'Sullivan
Denise O'Sulllivan
Maria Osuna
Szabolcs Osváth
Lynn Oswald

Claudia Osycka-Salut
Theresa Osypuk
Karolina Oszust
Takeyoshi Ota
Tsuguhito Ota
David Otaegui
Masaki Otagiri
Joji Otaki
Timothy Otchy
Juan Oteiza
Dan Otelea
Volkan Oter
Ana Otero
Larissa Otero
Francisco Otero Ferrer
Jorge Otero-Millan
Mona Othman
Megan Othus
Perrie O'Tierney-Ginn
Gard W. Otis
Fadar Otite
Mia O'Toole
Paul O'Toole
Domenico Otranto
Atsushi Otsuka
Shimpei Otsuka
Yasumasa Otsuka
Takemi Otsuki
Beate Ott
Johannes Ott
Martin Ott
Sascha Ott
Cristina Ottaviani
Jennifer Otten
Marte Otten
Jente Ottenburghs
Rainer Otter
Leo Otterbein
Richard Ottermanns
Gregory Otterson
Inger Ottestad
Penelope Ottewell
Gottfried Otting
Laura Ottini
Livia Ottisova
Karl Öttl
Jay Otto
Kevin Otto
Klaus Otto
Lars-Gernot Otto
Lukas Otto
Ross Otto
Suzie Otto
Gail Otulakowski
Easmon Otupiri
Kennedy Otwombe
Daniel Otzen
Fang-Shu Ou
Shuo-Ming Ou
Ting-Chia Ou
Wen-Bin Ou
Yali Ou
Yu-Yen Ou
Halima Ouadid-Ahidouch
Wael Ouarda
Amed Ouattara
Honoré Ouattara
Farhad Oubari
H. Oubrahim
Noriyuki Ouchi
Lavi Oud
Ruud Oudega
Lukas Oudejans
Ferid Oueslati
Kazuhisa Ouhara
Nassima Oumata
Sylvia Ounupuu
Merry Oursler
Cécile Oury
Franck Oury
Britt Ousterhout
Alexander Outkin
Alan Outram
D. Margriet Ouwens
Bo Ouyang
Fangqun Ouyang
Hanlin Ouyang
Lijing Ouyang

Min Ouyang
Minhui Ouyang
Dan Ovando
Sergey Ovchinnikov
Howard Ovens
Isaac Overcast
Will Overholt
T. Overton
Ken Overturf
Marina Oviaño
Pavel Ovseiko
Connie Ow
Tetsuhiro Owaki
Ghanem Oweis
Helen Owen
Leah Owen
Gina Owens
Hannah Owens
Julie Owens
Mandy Owens
Raymond Owens
Robert Owens
Tammy Owens
Amanda Owen-Smith
Patrick Owili
Peter Owira
Jessica Owley
Oluwatoyosi Owoeye
Olumuyiwa Owolabi
Onikepe Owolabi
Daniel Owusu
James Owusu-Kwarteng
Olivia Oxlade
Neil Oxtoby
Ryohei Oya
Tomomichi Oya
Jun-Ichi Oyama
Yoshimasa Oyama
Tonny Oyana
Ayako Oyane
Vinka Oyanedel-Craver
Micah Oyaro
Kola Oyediran
Olusegun Oyediran
Jannike Øyen
Ganiyu Oyetibo
Stephen Oyewobi
Olufemi Oyewole
Michiko Oyoshi
Katsuhisa Ozaki
Kikumi Ozaki
Kouichi Ozaki
Tsuneyuki Ozaki
Joan Ozanne-Smith
Shelly Ozark
Hitochi Ozawa
Fatih Ozaydin
Emel Ozbay
Tayfun Özçelik
Vural Özdemir
Denise Ozdemir-van Brunschot
Mürsel Özdoğan
Cemal Ozemek
Nurten Ozen
Mert Ozer
Mehmet Ozerdem
Andrew Ozga
Uluçkan Özge
Eren Ozguven
Lijo Cherian Ozhathil
Piotr Ozieranski
Engin Ozkan
Selman Özkan
Cigdem Ozkara
Olga Ozoline
Ryoji Ozono
Monica Ozores-Hampton
Tezcan Ozrazgat Baslanti
Yasemin Ozsurekci
Ferhat Ozturk
Yusuf Öztürkmen
M. Ozu
Albouy P.
Meenakshi P. Balakrishnan
Krijn Paaijmans
Taavi Paal
Ken Paap
Trudy Paap

Dave Paaraj
Nina Paauw
Jere Paavola
Noel Pabalan
Stephan Pabinger
Preciosa Corazon B. Pabroa
Christina Pacak
Karel Pacak
Jean-Pierre Paccaud
Claudio Pacchierotti
Amy Pace
Andrea Pace
Betty Pace
Matthew Pace
John Pacella
Settimio Pacelli
Edward Pace-Schott
Alicja Pacholewska
Jose Pachon
Jerónimo Pachón
Joel Pachter
Aude Pacini
Christopher Paciorek
Rene Packard
Theodore Packard
Thomas Packard
Nicolle Packer
Rowena Packer
Florent Paclet
Soumya Padala
Barbara Padalino
Jasdeep Padaria
Nesri Padayatchi
James Padbury
Michelle Paddack
Christopher Paddock
N. Padian
Nancy Padian
Danira Padilha
Nelly Padilla
Norma Padilla
Stephanie Padilla
Pablo Padilla-Longoria
Achuth Padmanabhan
Vasantha Padmanabhan
Luca Padua
Francesco Paduano
Amy Padula
R. Padwal
Nam-Chon Paek
Jin Chul Paeng
Hans Paerl
Adriana Paes Leme
Alessandra Paffi
Giuliano Andrea Pagani
Luca Pagani
Anthony Pagano
Duilio Pagano
Marcela Pagano
Andrew Page
Anne-Laure Page
Eric Page
Henry M. Page
Kimberly Page
Mark Page
Matthew Page
Nicola Page
Rick Page
Robert Page
Benjamin Pageaux
Cara Pager
Vincent Pages
John Paget
Stefano Pagiolo
Giuseppe Paglia
Silvia Pagliardini
Paulo Pagliari
Marco Paglione
Francesco Pagnini
Aline Pagnussat
Niema Pahlevan
Milena Pahlitzsch
Savita Pahwa
Akshay Pai
Kalpana Pai
Priya Pai
Su-Cheng Pai
Judith Paice

Ellie Paige
John Paige
Ken Paige
Samantha Paige
Ew Misty Paig-Tran
Bertrand Pain
Manuel Paina
Robert Paine
Dean Paini
Francesca Paino
Kevin Painter
Prem Pais
Alok Ranjan Paital
Biswaranjan Paital
Artur Paiva
Bruno Paiva
Sandra Paiva
Sergio Paiva
Vitor Paiva
Edward Pajarillo
Jukka Pajarinen
Gholam Pajenda
Krisztián Pajer
Judi Pajo
Massimo Pajoro
Maria Pajunen
Virpi Pajunen
Daniel Pak
On Shun Pak
Fatma Pakdil
Mariya Pakharukova
Omid Paknia
Hamideh Pakniat
Amir Pakpour
Farzaneh Pakzad
Abhijit Pal
Amit Pal
Debnath Pal
Dipan K. Pal
Jiban K. Pal
Krishnendu Pal
Mahadeb Pal
Sanjit Pal
Sudin Pal
Sukumar Pal
Tibor Pál
Maria Pala
Saziye Palabiyik
Cristina Palacios
Gustavo Palacios
Isabel Palacios
Jorge Palacios
Miriam Palacios-Callender
Jose Palacios-Jaraquemada
Nirianne Marie Palacpac
Patricia Palagi
Anna Palagyi
Joseph Palamar
Marco Palanca
Murali Palangat
Ashok Palaniappan
Raghavan Palaniappan
Rajkumar Palaniappan
Arvind Palanisamy
Manimaran Palanisamy
Nallasivam Palanisamy
Thirumoorthy Palanisamy
Senthilkumar Palaniyandi
Kartika Palar
Luis Palazzesi
Paola Palazzo
Ilaria Palchetti
Päivi Paldánius
Anandan Paldurai
Jan Palecek
Boris Igor Palella
Luigi Palese
Peter Palese
Gyorgy Palfi
Janos Palhalmi
Fernando Palhano
Fleur Paling
Miltiadis Paliouras
Isabella Pali-Schöll
Sumit Paliwal
Gopinadhan Paliyath
Claudia Palladino
Virginia Pallante

Federico Pallardo
Lucia Pallecchi
Ståle Pallesen
Nicolas Pallet
Philip Pallmann
Thomas Pallone
Carlo Pallotto
Johannes Pallua
Günther Palm
Giuseppe Palma
Jesse Palma
Regina Guenka Palma
Verónica Palma
Aurore Palmaro
Andre Palmer
Clovis Palmer
Colin Palmer
Craig Palmer
Dave Palmer
David Palmer
Evan Palmer
M. Palmer
Richard Palmer
Robert Palmer
Sally Palmer
Shea Palmer
Stephen Palmer
Jaime Palmer-Hague
Tullio Palmerini
Chiara Palmieri
Gabriella Palmieri
Tina L. Palmieri
Stefano Palminteri
Alida Palmisano
Carolyn Palmquist
Emily Palmquist
Paul Palmqvist
Arto Palmu
Glenn Palomaki
Leonardo Palombi
Gabriela Palomo
Jennifer Palomo
Martin Palomo
Riikka Paloniemi
Nathan Palpant
Snaebjorn Palsson
Arjan Palstra
Yniv Palti
Ora Paltiel
Ivana Palucci
Carsten Paludan-Muller
Peter Palukaitis
Francesca Palumbo
Paul Palumbo
S. Palumbo
Satu Palva
Sahithi Pamarthy
Mohan Pammi
Jeremy Pamplin
James Pampush
Derek Pamukoff
Aaron Pan
Ay-Woan Pan
Billy Pan
Chin-Chen Pan
Dongdong Pan
Fang Pan
Feifei Pan
Feng Pan
Fenghua Pan
G. Pan
Guoqing Pan
Hong-Chun Pan
I-Wen Pan
Jay Pan
Jeff Pan
Jia Pan
Jianbo Pan
Jianqing Pan
Jianxin Pan
Jiayi Pan
Junsong Pan
Li Pan
Linghui Pan
Qing Pan
Qingmin Pan
Qiuwei Pan
Rong Pan

Sheng-Wei Pan
Shin-Liang Pan
Songqin Pan
Tsorng-Whay Pan
Wei Pan
Weijun Pan
Wen-Chi Pan
Wensheng Pan
Xiaodong Pan
Xiaofei (Sophia) Pan
Xiaoju Pan
Xiaoping Pan
Xu Pan
Yong-Bao Pan
Yuchun Pan
Yue Pan
Z. Y. Pan
Eleftheria Panagiotaki
Demosthenes Panagiotakos
Hanna Panagiotopoulou
Panos Panagos
Efstathios Panagou
B. Panahi
Sirous Panahi
Maria Serena Panasiti
Donatella Panatto
Georgia Panayiotou
George Panayotou
Simonetta Pancaldi
Sethuraman Panchanathan
Anna R. Panchenko
Pallavi Panchu
Chinmay Panda
P. K. Panda
Rajmohan Panda
Sandeep Panda
Sanjib Kumar Panda
Subrat Panda
Arvind Panday
Dinesh Panday
Paritosh Pande
Irene Panderi
Ajay Pandey
Anita Pandey
Ashutosh Pandey
B. Pandey
Janmejay Pandey
Krupa Pandey
Manish Pandey
Priyanka Pandey
Rakesh Pandey
Sadanand Pandey
Satish Pandey
Subhash Pandey
Udai Pandey
Nikolaos Pandis
Maharaj Pandit
Sandeep Pandit
Carlos Pando
Anna Pandolfi
Victoria Pando-Robles
Marcus Pandy
Amit Pandya
Ankur Pandya
Covadonga Pañeda
Ronney Panerai
Reynold Panettieri Jr.
Elizabeth Pang
Jing Pang
John Pang
Joyce Pang
Junxiong Pang
Kevin Pang
Li-Li Pang
Myung-Geol Pang
Wenxing Pang
Xiaoming Pang
Ying Han Pang
Yuan-Ping Pang
Ari Pani
Jordi Paniagua
Narel Paniagua-Zambrana
Jalesh Panicker
Paola Panina-Bordignon
Bart Panis
Carol Panis
Ellen A. Panisko
Valéria Panissa

Frank Panitz
Maria Paniw
Carlos Panizo
Peter Panizzi
Neha Panjiar
Santosh Panjikar
Jules Panksepp
Sigamani Panneer
Julie Pannetier
Samuel Pannick
Evan Pannkuk
Giuseppe Pannone
Georgios Panos
Jens Panse
Maurizio Pansini
Chaitanya Pant
Deepak Pant
Sophie Pantalacci
Maria Pantaleo
Vitantonio Pantaleo
Eleonora Pantano
Francesco Pantano
Anastasia Pantazaki
Nikos Pantazis
Yannis Pantazis
Marija Pantelic
Athanasios Pantelous
Igor Pantic
Kostas Pantopoulos
Gabriella Panuccio
Aru Panwar
Francesco Panza
Carlos Arturo Panza Ospino
Francisco Panzera
Yanina Panzera
David Panzoli
Derek Pao
Wansa Paoin
Di Giulio Paola
Gabriele Paolacci
Paolo Paoletti
Pierre Paoletti
Chiara Paoli
George Paoli
Damiano Paolicelli
John Paolillo
Nazareno Paolocci
Ildiko Pap
Filomena Papa
Linda Papa
Federica Papaccio
Efstathios Papachristou
Maria Papadaki
Manos Papadakis
Marios Papadakis
Mike Papadakis
Marietta Papadatou-Pastou
Chrysoula Papadeli-Kourtidou
Georgios Papadelis
Evangelia Papadimitriou
Konstantinos Papadimitriou
Georgios Th. Papadopoulos
Nadia Papadopoulou
Virginie Papadopoulou
Nektaria Papadopoulou-Marketou
M. Papafaklis
Dimitrios Papageorgiou
Eleni Papageorgiou
Spyros Papageorgiou
Andriana Papaioannou
Theodore Papaioannou
Vasilios Papaioannou
William (Vasileios)
Spiros Papakostas
Thanos Papakostas
Demetris Papamichael
P. Papanagiotou
Nikolaos Papanas
Anastasios Papanastasiou
George Papandonatos
Mark Papania
Alexie Papanicolaou
Irene Papanicolas
Seraphim Papanikolaou
Domenico Paparella
Efstathios Paparoditis
Eric Papas

Sokratis Papaspyrou
Thomas Papastergiou
Stefania I. Papatheodorou
Ian Papautsky
Dimitrios Papavassiliou
Panagiota Papazafiri
Diane Papazian
Jason Papazian
Hans-Christoph Pape
Ulrich Pape
Liuba Papeo
Chiara Papetti
Mark Papich
Esther Papies
Magdalena Paplińska-Goryca
Garegin Papoian
Ilona Papousek
Gabor Papp
Zoltan Papp
Anna Maria Pappalardo
Luca Pappalardo
Christos Pappas
Ioannis Pappas
Maria L. Pappas
Peter Pappas
Michel Paques
Nicole Paquet
Paul Paquet
Allison Paquette
Benoit Paquette
Dana Paquette
Daniel Paquette
Max Paquette
Sebastien Paquette
Dominic Paquin-Proulx
Casey Paquola
Riccardo Paracampo
Amy Parachnowitsch
Andrés Parada
Luis Parada
Raul Parada
Marcos Paradelo
Angelo Paradiso
Prasad Paradkar
Kim Paraiso
Nirmala Parajuli
Rajendra Prasad Parajuli
Narayanan Parameswaran
Jesús Paramio
Jose Paramo
Elisabeth Paramythiotou
Ilan Paran
Augusto Paranhos
Beatrice Paranhos
Luiz Renato Paranhos
Thiyagarajan Paranthaman
Simona Paraschiv
Abhinav Parashar
Gayathri Parasivam
Christos Paraskeva
Despoina Parasyri
Vitor Paravidino
Marta Parazzini
Benjamin Parcell
Mark Parcells
Panos Pardalos
Melissa Pardi
Isabel Pardo
Marie Pardon
Zachary A. Pardos
Zachary Pardos
Machelle Pardue
Raul Paredes
Edgar Paredes-Gamero
Deseada Parejo
Bharat Parekh
Jiri Parenica
Christine Parent
Eric Parent
Lucie Parent
Marise Parent
Helena Parfenova
Dan Parfitt
Justin Pargeter
Peter Parham
Bibhash Paria
Ashish Parida
Ashok Kumar Parihar

Hardik Parikh
Sanjai Parikh
Shital Parikh
Urvi Parikh
Mataleena Parikka
Narasimham Parinandi
François Paris
Joel Paris
Sebastian Paris
Cesare Parise
Miriam Parise
Carrigan Parish
Andrea Parisi
Giuliana Parisi
Ludovica Parisi
Loukia Parisiadou
Marc-André Parisien
Nivaldo Parizotto
Beom-Seok Park
Chan Kee Park
Cheol Whee Park
Chloe Park
Choul Yong Park
Chungoo Park
Daeun Park
Elyse Park
Eui Kyun Park
Eun Hye Park
Hana Park
Hansoo Park
Hea Jin Park
Hee-Soo Park
Hwa-Jin Park
Hyun Soo Park
Hyung Sam Park
Hyung Soon Park
Hyung-Soon Park
Hyunjin Park
Hyunsook Park
Ina Park
Jae Bum Park
Jae-Il Park
James Park
Jeffrey Park
Je-Kyun Park
Ji Hoon Park
Ji-Man Park
Jin Park
Jin-Byung Park
Jong Hyock Park
Jongbae Park
Jong-Eun Park
Joon Min Park
Joontaek Park
Jumin Park
Jung Tak Park
Jung-Eun Park
Kevin Park
Kye Won Park
Kyoung-Joo Jenny Park
KyungBae Park
Lesley Park
Mee Sook Park
Me-Hea Park
Meyeon Park
Mina Park
Myung Park
No-Wook Park
Paul Park
Sang Gyu Park
Sang Myun Park
Sang-Kyu Park
Seong Yong Park
Seongjoon Park
Seonjoo Park
Si Hong Park
Song-Yi Park
Su-Hyung Park
Sung Bae Park
Sung Il Park
Sungha Park
Sung-Hye Park
Susanna Park
Sy Park
Tae-Ho Park
Tae-Yoon Park
Walter Park
Yoonjung Park

Yoonkyung Park
Yoonseong Park
Young-Gyu Park
Young-Jun Park
Om Parkash
Adwoa Parker
Albert Parker
Alex Parker
Benjamin Parker
Dan Parker
Dane Parker
Daniel Parker
Darren Parker
David Parker
Dawn Parker
Edward Parker
Helen Parker
James Parker
Jessica Parker
John Parker
Lewan Parker
Lisa Parker
Lukas Parker
Mark Parker
Matthew A. Parker
Matthew Parker
Megan Parker
Stephen Parker
Tony Parker
Tracey Parker
Wendy Parker
William Parker
Neil Parkin
David Parkinson
Dilworth Parkinson
John Parkinson
Kathryn Parkinson
Kyung-Hyun Park-Min
Brad Parks
Robin Parks
Sean Parks
Onur Parlak
Ara Parlakian
Rosanna Parlato
Valentina Parma
Bidhan Parmar
Henk Parmentier
Christopher Parmeter
Sara Parmigiani
Daniel Parnell
Laura Parnell
Andrea Parniczky
Rangsun Parnpai
Alberto Parola
Abhijit Parolia
Carola Parolin
Maria Paola Paronetto
Matteo Parotto
Ann Parr
Nicole Parr
Eduardo Parra
Gabriel Parra
Miguel Parra-Saavedra
Steven Parratt
Mariacristina Parravano
Chiara Parravicini
Ricardo Parreira
Diego Marcel Parreira de Castro
Paola Parrella
Paul Parren
Fey Parrill
Darren Parris
Alan Parrish
Audrey Parrish
Colin Parrish
Benjamin Parrott
Giustino Parruti
Geraint Parry
John Parsch
Simon Parson
Walther Parson
Carole Parsons
Darren Parsons
Mark Parsons
Michael Parsons
Nola Parsons
Seth Parsons
Stuart Parsons

Todd Parsons
Eric Parteli
Stefano Partelli
Kaushik Parthasarathi
Raghuveer Parthasarathy
Sai Parthasarathy
Rajendran Parthiban
Mark Parthun
Santiago Partida-Sanchez
Debra Partington
Timo Partonen
Gavin Partridge
Agnieszka Partyka
Wendy Parulekar
Iffat Parveen
Shama Parveen
Michal Parzuchowski
Sirichai Pasadhika
Fabrizio Pasanisi
Stefano Pascarella
Nicola Pascarelli
Tristan Pascart
Andreas Pasch
Eleftherios Paschalis
Stephan Paschke
Ben Pascoe
David Pascoe
Peter Pascoe
Francesco Pascoli
Belen Pascual
David Pascual
Julio Pascual
Marta Pascual
David Pascual-Ezama
Vincenzo Pascucci
Manijeh Pasdar
Oscar Paseta
Marcela Pasetti
Farhad Pashakhanloo
Nausheen Pasha-Zaidi
Marco Pasi
John Pasick
E. Pasicka
Giulio Pasinetti
Erica Pasini
Giacomo Pasini
Margherita Pasini
Jantsje Pasma
Edoardo Pasolli
Sandra Pasoto
Teresa Pasqua
Louis Pasquale
Valentina Pasquale
Florence Pasquier
Jérémy Pasquier
Amy Pasquinelli
Gianandrea Pasquinelli
Juana Pasquini
Hélène Pasquini-Descomps
Anna Maria Pasquino
Caroline Passaes
Alberto Passalacqua
Sabina Passamonti
Alessandro Passardi
Giuseppe Passarino
Luiz Passero
Samantha Passey
Nadine Paßlack
Elyse Passmore
Jo-Ann Passmore
Maria Rita Passos-Bueno
Sonak Pastakia
Irena Pastar
Jacques M. Pasteels
Robert Pastel
Lucio Pastena
Taras Pasternak
Maria Bruna Pasticci
Pau Pastor
Alvin Pastore
Antonio Pastore
Luca Pastorelli
Gregory Pastores
Luigi Pastormerlo
Bernhard Pastötter
Carlos Pastre
Alexander Pastukhov
Alexander Pastuszak

Kalyan Pasupathy
Solenn Patalano
Jose Patane
Manish Patankar
Manuel Patarroyo
Russell Pate
Aloka L. Patel
Alpa Patel
Amrish Patel
Anjni Patel
Bhavesh Patel
Dipika Patel
Girijesh Patel
Jaymin Patel
Jitu Patel
Kanan Patel
Kaushik Patel
Kepal Patel
Krishna Patel
Mahomed Patel
Mainak Patel
Mayur Patel
Minal Patel
Nachiket Patel
Nilesh Patel
Nita Patel
Oneel Patel
Pragnesh Patel
Rakesh Patel
Rena Patel
Rohit Patel
Rupal Patel
Ryan Patel
Sheila Patel
Shivani Patel
Sona Patel
Vainav Patel
Vinod Patel
Vyomesh Patel
Yashomati Patel
Yashwant Patel
V. Patella
Kelsey Pateman
Abby Paterson
Gavin Paterson
Helen Paterson
Iain Paterson
Ian Paterson
Jessica Paterson
John Paterson
Mary Paterson
Rachel Paterson
Yvonne Paterson
Bhupendra Pathak
Jai Pathak
Medha M. Pathak
Praveen Pathak
Varun Pathak
Sameena Pathan
Sisira Pathirana
Wasu Pathom-Aree
Gregory Patience
Dimitrios Patikas
Anita Patil
Grete Grindal Patil
Indrajeet Patil
Naeem Patil
Pravinkumar Patil
Sujata Patil
Sumeet Patil
Susheel Patil
Franck Patin
Heather Patisaul
Shane Patman
Sourav Patnaik
Jiří Patoka
Chris Paton
Maxime Patout
Abani Patra
Amlan Patra
Sanjukta Patra
Kathryn Patras
Om Patri
Kennerly Patrick
Kirk Patrick
Steve Patrick
Wayne Patrick
Hanley Patrick J.

Paola Patrignani
George Patrinos
Glenn Patriquin
Nicoletta Patrizi
Liliana Patrucco
Antonia Patruno
Vallandramam Pattabhiraman
Smita Pattanaik
Arunava Pattanayak
Sanjaya Pattanayak
Cristian Pattaro
Kim Patten
Adrianna Patterson
Andrew Patterson
Chavis Patterson
Edward Patterson
Jillian Patterson
Kimberly Patterson
Giuseppe Patti
Chris Pattillo
Dorothy Patton
Stephanie Patton
Alexandre Pattyn
Roberto Patuelli
Abhijit Patwardhan
John Patykowski
Jonathan Patz
Babu Ram Paudel
Surya Paudel
Arindam Paul
David Paul
Dennis Paul
Diby Paul
Jaishree Paul
John Paul
Lee Choon Keat Paul
Manoranjan Paul
Matthew Paul
Parameswari Paul
Sanjoy Kumar Paul
Serene Paul
Shyam Paul
Sourav Paul
Sudeshna Paul
Suman Paul
Débora Paula
Jayter Paula
Trazilbo José de Paula Junior
Fabiola Paula-Lopes
Mike Paulden
Harald Pauli
Wolfgang Pauli
Heather N. Paulin
Ariana Paulina-Carabajal
John Pauling
Stefan Pauliuk
Nicole Paulk
Ramasamy Paulmurugan
Silva Paulo
Barbara Pauloski
Dennis Pauls
Peter Pauls
Steffen Pauls
Jonas Paulsen
Peter Paulsen
Joseph Paulson
Achim Paululat
Martin Paulus
Francisco Paumgartten
Tatjana Paunesku
Ralf Paus
Thomas Pausch
Bettina Pause
Zdenka Pausova
Joseph Pauszek
Steve Pauszek
Milica Paut Kusturica
Laurens Pauwels
Mariela Pavalache-Ilie
Fernando Pavan
Nicola Pavan
Vladimir Pavan Margarido
Chiara Pavanello
Sofia Pavanello
Theetha Pavankumar
Mauro Sérgio Pavão
Gaspare Pavei
Roman Pavela

Hermann Pavenstädt
Nicola Pavese
Giulio Pavesi
Chris Pavey
Marco Pavia
Nicolas Pavillon
Brian Pavilonis
Paulo Pavinato
Graham Pavitt
Meda Pavkov
George Pavlakis
Tomas Pavlicek
Mihaela Pavlicev
Nena Pavlidi
Mojca Pavlin
Georgios Pavlopoulos
Chavdar S. Pavlov
V. Pavlov
Youri Pavlov
Vladimir Pavlovic
Christian Pavlovich
Sandrine Pavoine
Graciela Pavon-Djavid
Enea Pavone
Mary Ellen Pavone
Kantikumar Pawar
Rajendra Pawar
Shivshaki Pawar
Graham Pawelec
Magdalena Pawełkowicz
M. Pawlak
Michal Pawlak
Mat Pawley
Lucia Pawloski
Katharina Pawlowski
Izabella Pawluczyk
Julia Paxson
Alexandra Paxton
Eben Paxton
Benjamin Payne
Matthew Payne
Peter Payne
R. Payne
Pierre Payoux
Bernard Payrastre
Jacqueline Payton
Clea Paz
Gabriela Paz-Bailey
Adam Pazda
Gilberto Paz-Filho
Farid Pazhoohi
Simonetta Pazzaglia
Mary Peacock
Nadine Peacock
Zachary Peacock
Corey Peak
Jonathan Peake
Anna Pearce
Bradley D. Pearce
Douglas Pearce
Elizabeth Pearce
Grant Pearce
Hannah Pearce
J. Pearce
Philip Pearce
Stephen Pearce
William Pearce
Michael Pearen
Ronald Pearl
Eric Pearlman
William Pearse
Carl Pearson
David Pearson
Erin Pearson
Gareth Pearson
Heidi Pearson
James Pearson
Paul Pearson
William Pearson
Zoe Pearson
Claire Peart
Jason Peart
Samuel Peasah
Brent Pease
Bruno Peault
Tiago Peçanha
Clint Pecenka
Pavel Pech

Jennifer Pechal
Tibor Pechan
Severine Pechine
Raimund Pechlaner
Sonali Pechlivanis
Bailey Peck
Michael Peck
Robert Peck
Sarah Peck
Daniel Peckham
Emily Peckham
Michelle Peckham
Jörn Peckmann
Christian Peco
Roberto Pecoits-Filho
Lorenzo Pecoraro
Jacques Pecreaux
Tetyana Pedchenko
Valerien Pede
Crisitna Pederiva
Alma Pedersen
Brian A. Pedersen
Carsten Pedersen
Iben Bach Pedersen
Lars-Flemming Pedersen
Lee Pedersen
Mona Kyndi Pedersen
Niels Pedersen
Oliver Pedersen
Timothy Pedley
Ali Pedram
Juan Manuel Pedraza
José Pedraza-Chaverri
Mario Pedraza-Reyes
Monica Pedrazza
María Pedreira
Romina Pedreschi
Alessandro Pedretti
Simone Pedrini
Gianni Pedrizzetti
Joao Pedro
Pedro Bordalo Pedro Bordalo
Thierry Pedron
Fatima Pedrosa-Domellöf
Andrea Pedrosa-Harand
Adriana Pedroso
Chih Yang Pee
Ray Peebles
Corinne Peek-Asa
Ed Peeler
Jaap Peen
Mark Peeples
Cody Peer
Wendy Peer
Olve Peersen
Daniel Peet
Martine Peeters
Sarah Peeters
Koen Peeters Grietens
Marie Pegelow
Elise Pegg
Mark Pegg
João Pêgo
Teresa Pegors
Amarendra Pegu
Haiping Pei
Jianming Pei
Kurtis Pei
Sen Pei
Shuwen Pei
Tao Pei
X. B. Pei
Yonggang Pei
York Pei
Ana Belén Peinado
Victor Peinado
Arne Peine
Stéphane Peineau
Frank Peinemann
Matthias Peipp
M. Peiris
Amado Peiro Gimenez
Rebecca K. Peisley
Panagiotis Peitsidis
Henry Peixoto
Sofia Peixoto-de-Matos
Liba Pejchar
Martin Pejchinovski

Gunnar Pejler
Stanislav Pekar
Joel Pekow
Daniel Pelaez
Pawel Pelczar
Vicent Pelechano
Konstantinos Pelechrinis
Yoav Peleg
David Pelegrín-García
Melanie Pelegrini
Simone Peletto
Giovanni Pellacani
Maria Teresa Pellecchia
Adam Pellegrini
Barbara Pellegrini
Marco Pellegrini
Thais Pellegrini
Gabriella Pellegriti
Chelsea Pelletier
Luc Pelletier
Philip Pellett
Liz Pellicano
Antonio Pellicer
Jaume Pellicer
Vivien Pellis
Paolo Pelosi
Elisa Pelosin
Ville Peltola
Timo Peltomaki
Stephen Pelton
Juha Peltonen
Markku Peltonen
Gilles Peltzer
Karl Peltzer
Ilaria Peluso
Kirsten Pelz-Stelinski
Alex Pemov
Vanessa Pena
Estefania Peña
Fernando Peña
Rafael Peñafiel
Francisco Peñagaricano
Enrique Penalver
Mauro Penasa
Reyes Peña-Santiago
Eugenia Pena-Yewtukhiw
Brian Pence
Dustin Pendell
Julie Pendergast
John Penders
Sarah Pendlebury
Vishnu S. Pendyala
Petronila Penela
Vlada Peneva
Aimin Peng
Chao Peng
Cheng-Yuan Peng
Chiung-Yu Peng
Chun Peng
Daxin Peng
Giia-Sheun Peng
Han Peng
Hanwei Peng
Hui Peng
Jiajie Peng
Jianping Peng
Jiaxi Peng
Jie Peng
Junhua Peng
Ke Peng
Kuan-Po Peng
Lingli Peng
Liu Peng
Man Peng
Ningyue Peng
Shaoliang Peng
Ting Peng
Wei Peng
Xi Peng
Xianjing Peng
Xianjun Peng
Xiao-Long Peng
Xichun Peng
Xin Peng
Xinxia Peng
Xu Peng
Xuefeng Peng
Yan Peng

Yanhui Peng
Yanyan Peng
Yi Peng
Yifan Peng
Yi-Jen Peng
Yong Peng
Yun Peng
Zhangli Peng
Zhouhua Peng
Zongju Peng
Supa Pengpid
Clint Penick
Andrew Penn
Fabio Penna
Angela Pennacchio
Amy Pennay
Iris-Katherina Penner
Johannes Penner
Giuseppa Pennetta
Kathryn Penney
Rosalind Penney
Katie Pennington
Toby Pennington
Kristina Penniston
Robert Pennock
Fulvia Pennoni
Mary Penny
Keith Pennypacker
Caterina Penone
Kalyana Chakravarthy Pentapati
Bradley Pentelute
Heikki Pentikainen
Kirsi Penttinen
William R. Penuel
Marianna Penzo
Anita Peoples
Brandon Peoples
Francesco Pepe
Salvatore Pepe
Nicholas Pepin
Marc Pépino
Beata Peplonska
Antonella Peppe
Daniel Peppe
Alan Pepper
Amber Pepper
Carolyn Pepper
Michael Pepper
Irene Pepperberg
Kathryn Pepple
María Perales
Juan Manuel Peralta-Sanchez
Simone Perandini
Daniela Perani
Francesco Peraro
Mark Perazella
Matjaz Perc
Gennaro Percannella
Gaetano Perchiazzi
Valentina Perciavalle
Piergiorgio Percipalle
Alan K. Percy
Elise Percy
João Perdigão
Paris Perdikaris
Kalliopi Perdikouri
Roberto Perdisci
Catalina Perdomo
Nicolas Perdrial
Hervé Perdry
Katherine Perdue
Silvia Perea
Felipe Perecin
Ariel Pereda
Ernesto Pereda
Carlos Peredo
Giovanni Battista Perego
Ugo Perego
Haydee Peregrina
Anderson Pereira
Andre Pereira
Antonio Pereira
Audrey Pereira
David Pereira
Ethel Pereira
Gabriel Pereira
Gerson Pereira
Gleber Pereira

Jaqueline Pereira
Luisa Pereira
Luiz Filipe Pereira
M. Graça Pereira
Marcos Pereira
Maria Alcina Pereira
Odilon Pereira
Roberto Pereira
Rosa Maria Pereira
Stacey Pereira
Tânia Pereira
Nuno Pereira-Azevedo
Adaucto Pereira-Netto
Mercio Pereiraperrin
C. Pereira-Wilson
Josep Perello
Polina Perelman
Marlon Perera
Ranawaka Perera
Ranjan Perera
Mario Peres
Rosa Perestrelo
Isabelle Peretz
David L. Perez
Enrique Perez
Fernando Perez
Franck Perez
Gilberto Perez
J. Christian Perez
Jesus Perez
Luis Perez
Marc Lucia Perez
Rodrigo O. Perez
Ruth Perez
Samara Perez
Selene Perez
Sonia Perez
Francisco Pérez
G. Pérez
J. Ricardo García Pérez
Laura Pérez
Nicolás Pérez
William Pérez
Magdiel Perez Cruz
Enrique Perez Cuadrado
Ricardo Perez Cuevas
L. Perez de Liano
Luis Pérez De Sevilla Mueller
Miguel Pérez Fontán
Juan Pérez García
Guillem Pérez i de Lanuza
Jorge Perez Juste
Daniel Perez Marcos
Gaspar Pérez Martínez
Guillermo Perez Perez
Nina Pereza
Miguel Pérez-Amador
Alexis Pérez-Bellido
Daisy Perez-Brito
Amaya Perez-Brumer
Juliana Perez-Chaparro
Maria-Cristina Pérez-Conesa
Sofia Perez-del-Pulgar
Miguel Perez-Enciso
Adan Perez-Garcia
Margarita Perez-Jimenez
Faustino Perez-Lopez
Marcos Perez-Losada
Alejandro Perez-Matus
Valentín Pérez-Mellado
Nestor Perez-Mendez
Manuel Perez-Molina
Francisco Pérez-Montaño
Dafne Pérez-Montarelo
Mirna Perez-Moreno
Rogelio Perez-Padilla
Daniel Pérez-Prieto
Antonio Pérez-Pulido
Ariadna Pérez-Ricart
Pilar Perez-Romero
Ana Perez-Ruiz
Dolores Pérez-Sala
Jaume Pérez-Sánchez
Josue Perez-Santiago
Jose Perez-Simon
Ricardo Pérez-Tomás
Javier Perez-Tris
Patricia Pérez-Vera

Francisco Perez-Vizcaino
Mario Pérez-Zepeda
John Perfect
Simona Perga
D. Pergolizzi
Joseph Pergolizzi
Muthu Periasamy
Sivakumar Periasamy
Stojan Perić
Luca Perico
Norberto Perico
Eva Pericolini
Srikanth Perike
Bruno Perillo
Jamie Perin
Jérôme Perin
Giuseppe Perinetti
Renza Perini
David Peris
Palsamy Periyasamy
Lindsey C. Perkin
Adam Perkins
Alex Perkins
Archibald Perkins
Darren Perkins
Douglas Perkins
Jessica M. Perkins
Timothy Perkins
Michael Perko
David Perlin
Dan Perlman
Elisabeth Perlman
Susan Perlman
Jennifer Perloff
Jenny Permuth
Andrea Perna
Eduardo Perna
Cyril Pernet
Diahn-Warng Perng
Guey Chuen Perng
José C. Pernías-Cerrillo
Ernst Pernicka
Gilles Pernod
Claire Pernollet
Ana Carolina Pero
Franck Peron
Guillaume Péron
Rosario Perona
Odile Peroni
Silvio Peroni
Jeanne-Marie Perotin
Silvia Perotto
Olga Perovic
Florence Perquier
Nicola Perra
Adrien Perrard
Alexandra Perras
Cecile Perrault
Matthieu Perreau
Jean-Pierre Perreault
Cyril Perret
Stéphanie Perret
David Perrett
Prescilla Perrichon
Carlo Perricone
Diana Perriman
Abgail Perrin
Benjamin Perrin
Maryanne Perrin
Chaurand Perrine
Laurent Perrinet
Sebastio Perrini
Ronald Perrone
Serafina Perrone
Nora Perrone-Bizzozero
Tara Perrot
Cristiana Perrotta
Nader Perroud
Gianluca Perrucci
Christophe Perruchoud
Adam Perry
Alison Perry
Amanda Perry
Christopher Perry
Conrad Perry
Ian Perry
John Perry
Lynn Perry

Roland Perry
Seth Perry
Sharyn Perry
Tahlia Jade Perry
Yves-Marie Pers
Dominique Persano Adorno
Michael Persans
Deborah Persaud
Gianluca Perseghin
Mike Persia
Marcello Persico
Olga Perski
Derek Peršoh
Jane Persons
Alexander Persoskie
Anna Persson
Jenny Persson
Karina Persson
Patrik Persson
Pontus Persson
Maria Perticone
Luis Pertierra
Giovanni Pertosa
Arkady Pertsov
Inna Pertsovskaya
Manuel Perucho
Ekambaram Perumal
Ramssamy Perumal
Velisha Ann Perumal-Pillay
Francesca Peruzzi
D. Perwitasari
Stephen Perz
Giovanni Pes
Maria Pesantes
Todd Pesavento
Antonio Pesce
Anne Peschel
Simone Peschillo
Mario Pesendorfer
Francesca Pesola
Graziano Pesole
Eero Pesonen
João Bosco Pesquero
George Pess
Juliano Pessan
Sofia Pessanha
Dalton Pessoa Filho
Dominik Pesta
Manuel Pestana
Helen Petach
Udomporn Petchthai
Bogucki Peter
Eibich Peter
Hyde Peter
Karlheinz Peter
Christin Peteranderl
Jutta Peterburs
Angel Peterchev
Miklos Peterfy
Borut Peterlin
William Peterman
Anne Peters
Brian Peters
Donna Peters
Dorien Peters
Dorothea Peters
Ellen Peters
Isabella Peters
James Peters
Jan Peters
Joerg Peters
Jonathon Peters
Jurriaan Peters
Kim Peters
Michael Peters
Mike Peters
Paul Peters
Remco Peters
Reuben Peters
Ryan Peters
Sabine Peters
Wibke Peters
Asa Petersen
Christine Petersen
Inge Petersen
Jakob Petersen
Jessica Petersen
John Petersen

Jörn Petersen
Kitt Petersen
Kristina Petersen
Michael Petersen
Olav Petersen
Rasmus Petersen
Suni Petersen
Simon Petersen-Jones
Amie Peterson
Blake Peterson
Brennan Peterson
Carrie Peterson
Catherine Peterson
Charles Peterson
Christian Peterson
Courtney Peterson
Daniel Peterson
Elizabeth Peterson
Ellena Peterson
Ib Krag Peterson
James Peterson
Jen Peterson
K. M. Peterson
Mark Peterson
Matthew Peterson
Sean D. Peterson
Townsend Peterson
Kevin Pethe
Ronald Petie
Steven Petit
Caroline Petitjean
Eleni Petkari
Chris Petkov
Eva Petkova
Carmen Lucia Petkowicz
Thomas Peto
Tunde Peto
Francesca Petralia
Daniel Petras
Francesco Petrella
Fausto Petrelli
Valery Petrenko
Jay Petrick
Lauren Petrick
Athanasios Petridis
Josh Petrie
Vilma Petrikaite
Claudia Petritsch
Thomas Petro
Vanessa Petro
Nicola Petrocchi
Elena Petroff
Andrew Petroll
Zvezdana Petronijevic
Fotios Petropoulos
Ioannis Petropoulos
Tim Petros
Irene Petrosillo
Stefania Petrosino
Gohar Petrossian
Katherina Petrou
Styliani Petroudi
Djafari Petroudy
Tatjana Petrov
Silviu Petrovan
Constantinos Petrovas
Bojan B. Petrovic
Daniel Petrovic
Mriko Petrovic
Zoran Petrovic
Nikolai Petrovsky
Nicole Petrowski
Robin Petroze
Hilda Petrs-Silva
Harry Petrushkin
Jeffrey Petruska
Antonio Petruzzelli
Clive Petry
Franck Petry
Constantinos Petsoglou
James Pettengill
Kjell Sverre Pettersen
Mark Petticrew
Steve Pettifer
Audrey Pettifor
Kerry Pettigrew
Luisa Pettigrew
Matteo Pettinari

April Pettit
Nathan Pettit
Robin Pettit
Paul Pettitt
Julien Pettré
Tobias Petzold
Fiorenzo Peverali
Joshua Pevnick
Stefanie Peykarjou
Payam Peymani
Adrien Peyrache
Thierry Peyrard
Matthieu Peyre
Leonardo Peyré-Tartaruga
Andy Peytchev
Flora Peyvandi
Adel Pezeshki
Thomas Pezier
Raffaele Pezzilli
Daniela Pfabigan
Alexander Pfaff
Christian Pfaff
Donald Pfaff
Kathryn Pfaff
George Pfaltzgraff
Boris Pfander
Kenneth Pfarr
Christiane Pfarrer
Stefan Pfattheicher
Karin Pfeffer
Ulrich Pfeffer
Carmem Pfeifer
Nico Pfeifer
Yvonne Pfeifer
Christine Pfeiffer
Dagmar Pfeiffer
Dirk Pfeiffer
Douglas Pfeiffer
Karin Pfeiffer
Michaela Pfeiffer
Isabella Pfeil
Frank Pfennig
Stefan Pfenninger
Steffen Pfeuffer
Thorsten Pfirrmann
Riccardo Pfister
Cathie Pfleger
Lance Pflieger
Paul Pfluger
Daniel Pflugfelder
Stephen Pflugfelder
Dominik Pförringer
Michaela Pfundmair
Gert Pfurtscheller
Girija Phadke
Suhas Phadnis
Budy Phaedra
David Phalen
Vincent Phalip
Anh-Duc Pham
Christopher Pham
Kasey Pham
Tuan Pham
Viet-Thanh Pham
Chau-Minh Phan
Chia-Wei Phan
Hang T. T. Phan
Tung Phan
P. Terry Phang
Souly Phanouvong
Gregg Phares
Anastasia Pharris
Carmen Pheiffer
Benjamin Phelps
Suzanne Phibbs
Megan Phifer-Rixey
Michael Philben
Benjamin Philip
Bodo Philipp
Andrew Philippides
Alain Philippon
Anastassios Philippou
Nolan Philips
Veit Phillip
Mark Phillippe
Julia Phillippi
Anna Phillips
Caleb Phillips

Christopher Phillips
Clive Phillips
Ian Phillips
James Phillips
Jeffrey Phillips
John Phillips
Julie Phillips
Nicole Phillips
Patrick Phillips
Rhonda Phillips
Robert Phillips
Samuel Phillips
Stuart Phillips
Thomas Phillips
Timothy (Tim) Phillips
Penelope Phillips-Howard
Andy Philp
Nancy Philp
Helen Phipps
Sam Phiri
Colin Phoon
Ann Phoya
Jack Phu
Sirinya Phulkerd
Sasitorn Phumdoung
Dung Phung
Ho Ngoc Phuong
Mark Phuong
Leonidas Phylactou
Eliane Piaggio
Giulia Piaggio
Maria Piagkou
Jean-Philippe Pialasse
Luis Pianciola
Elisa Pianigiani
Marianne Piano
Timothy Pianta
Steven Piantadosi
Diego Piantedosi
Zhongyun Piao
Renaud Piarroux
Lucila Piasecki
Gretchen Piatt
Ornella Piazza
Victoria Piazza
Luigi Piazzi
Ana Picado
Brigitte Picard
Christine Picard
Frédéric Picard
Martin Picard
Laura Picas
Susan Picavet
Pier Paolo Piccaluga
Simona Picchietti
Maria Picchio
Jonathan Piccini
Francesca Piccinini
Laura Piccio
Fiorenzo Piccioli
Federico Piccioni
Marco Piccirilli
Gianfranco Piccirillo
Agustin Picco
Noemi Picco
Giovanni Piccoli
Alessandro Picelli
Pietro Picerno
Alain Piche
Lukas Pichelstorfer
Anna Pichiecchio
Bernd Pichler
Martin Pichler
Andreas Pichlmair
Sebastien Pichoff
Aurélien Pichon
Claude Pichonnaz
Christian Pichot
Yuriy Pichugin
Marina Picillo
Jean-François Picimbon
Andrea Picin
Andrew Pickering
James Pickering
John Pickering
Mark Pickering
David Picketts
William Picking

Peter Pickkers
Nathaniel Pickle
Michael Pickles
Jana Pickova
Yolanda Pico
Gabriel Picone
Daniel Picot
Erin M. Picou
Vincent Picozzi
Sandrine Picq
Joel Picus
Nataliya Pidkovka
Guillaume Pidoux
Karin Pieber
Piotr Pieczywek
Pedro Piedra
Jorge Piedrahita
Walter Piegorsch
Fredrik Piehl
Dorota Piekutowska-Abramczuk
Annemarie Pielaat
Jan Pielage
Gary Pielak
Martin Pienkowski
Amy Pienta
Barbara Pieper
Robert Pieper
Emily Pieracci
Denis Pierard
Elisa Pierboni
Brandon Pierce
David Pierce
Mark Pierce
Melissa Pierce
Richard Pierce
Brian Pierchala
Peter Piermarini
Paolo Pierobon
Philipp Pieroh
Andrea Pieroni
Valerie Pierrefite
Pascal Pierre-Yves
Luisa Pierro
Silvana Piersanti
Elise Piersol
Hannah Pierson
Mariusz Pierzchala
Guillaume Piessen
Bradley Pieters
Jean Pieters
Roland Pieters
Giacomo Pietramellara
Filomena Pietrantonio
Roberto Pietrantuono
Zbigniew Pietras
Ricardo Pietrobon
Giada Pietrosi
Paul Pietroski
Fabio Pietrucci
Konrad Pietruk
Michal Pietrusinski
Marcin Pietrzykowski
Maik Pietzner
Fabien Pifferi
Simone Pifferi
Jon Piganelli
Hugh Piggins
David Pigott
Lasse Pihlstrøm
Youry Pii
Kristin Piikki
Saija Piiroinen
Tiina Piiroinen
Maarit Piirtola
B. Pijls
Tom Pike
Slaven Pikija
Barbora Piknova
Eva Pila
Natalile Pilakouta
Eleftherios Pilalis
Robert Pilarski
Angelina Pilatti
Adrian Pilatz
Cc Pilbeam
David Pilcher
Nicolas Pilcher
Hans Kristian Pilegaard

Hans Pilegaard
Henriette Pilegaard
Stefano Pileri
Marie-Laure Pilet
Erik Pilgrim
Jennifer Pilgrim
Aleksandr Pilipenko
Natasha Pilkauskas
Anitha Pillai
Dylan Pillai
Smitha Pillai
Suresh D. Pillai
Ajay Pillarisetti
Viness Pillay
Evangeline Pillebout
Flavien Pillet
Elena Pilli
Karin Pillunat
Elizabeth Pilon-Smits
Violetta Pilorz
Terhi Piltonen
Kattja Piltti
Stefan Pilz
Ville Pimenoff
Paulo Pimenta
David Pimentel
Laura Pimentel
Márcia Mattos Gonçalves Pimentel
Catalina Pimiento
François Pimont
Clément Pimouguet
Christopher Pin
Paolo Pin
Chueh Pin Ju
Melanie Pina
Halit Pinar
M. Pinar
Guadalupe Piñar
Luciana Pinato
Lauren Pinault
Gilles Pinay
Sylvain Pincebourde
Stephanie Pincetl
Roxana Pincheira
David Pincus
Alana Pindar
Ross Pinder
Charles Pineau
Pascal Pineau
Federico Pineda
Jose Pineda
Oscar Pineda-Catalan
Pablo Pinedo
Bernadette Pinel-Alloul
David Pinero
Janet Piñero
Heather Pines
G. Pineton de Chambrun
José Pingarrón
Alessandro Pingitore
Pasqualantonio Pingue
Albert Pinhasov
Flávio Pinheiro
Marcos Pinho
Else Marie Pinholt
Alessandro Pini
Laura Pini
Beate Pinior
Ryan Pink
Michael Pinkawa
Katja Pinker
Katja Pinker-Domenig
Aaron Pinkhasov
Somchai Pinlaor
Gavin Pinniger
Karina Pino-Lagos
Maria Pino-Yanes
Peter Pinsky
Mathew Pintar
Lionel Pintard
Camelia M. Pintea
David Pintel
Joseph Pinter
Marcello Pinti
Alexander Pinto
Amelia Pinto
C. Miguel Pinto

Caroline Pinto
Daniel Pinto
Diana Pinto
Ines Pinto
Israel Pinto
Jose Pinto
Juliana Pinto
Lancelot Pinto
Luciana Pinto
Ricardo Mouro Pinto
Rodrigo Pinto
Susana Pinto
Yair Pinto
Rosa Pintó
Sara-Joan Pinto-Sietsma
Eliana Pintus
Jillian Pintye
Alexander Pintzas
Pamela Pinzani
Astrid Pinzano
Manuela Pinzari
David Pinzon
Martha Pinzón Daza
Cheryl Pinzone
Marco Piola
Dario Piombino-Mascali
Katarzyna Piórkowska
Augen Pioszak
Niels Piot
Susanna Piovano
Davide Piovesan
Barbara Pipan
Benjamin Piper
Matthew Piper
Barbara Piperata
Laura Piqueras
Alexis Piquero
Stefano Piraino
Payam Piray
Marco Pirazzini
Sandeep Pirbhulal
Aliny Pires
Bruno Pires
Douglas Pires
Gabriel Pires
Ricardo A. Pires
Ricardo Pires
Sara Pires
Andre Pires da Silva
Renato Jose Pires Machado
Gaëlle Piret
Fraser Pirie
Phyllis Pirie
Steven Pirie-Shepherd
Primoz Pirih
Angela Pirillo
Eija Pirinen
Lucia Pirisi-Creek
Christian Pirk
Robert Pirker
Markus Pirklbauer
Jean-Paul Pirnay
Benoit Piro
Gabriella Piro
Salvatore Piro
Carlos Pirola
Luciano Pirola
Valentino Pironti
Jodi Pirtle
Antonio Pisabarro
Didier Pisani
Elizabeth Pisani
Luigi Pisani
Anthony Pisaniello
J. Pisano
Katarzyna Pisanski
Benoit Pisanu
Monika Pischetsrieder
Livia Pisciotta
Pietro Pisciotta
Toni Piseddu
Andrzej Pisera
David Pisetsky
Rein Jan Piso
Alberto Pisoni
Anne Pisor
Francesco Pistelli
Emidio Pistilli

Alberto Pistocchi
Francesca Pistoia
Vito Pistoia
Wojciech Pisula
Diana Pita
Dyah Pitaloka
Kalliopi Pitarokoili
Ramasamy Pitchappan
Brandelyn Pitcher
Kristopher Pitcher
Toni L. Pitcher
Marco Pitino
Kimberley Pitman
Dario Pitocco
Fabián Pitoia
Johann Pitout
Ian Pitt
Kylie Pitt
Sharon Pitteri
Didier Pittet
Valérie Pittet
Benjamin Pittman-Polletta
R. Pitts
Stephen Pitts
Teresa Pitts
Theresa Pitts-Singer
Joseph Pitula
P. Pitule
Mariana Piuri
Nicolas S. Piuzzi
Bruno Piva
Francesco Piva
Francesca Pivari
Rosario Pivonello
Sarah Piwetz
Ellen Piwoz
Fiona Pixley
John Pixley
Sarbottam Piya
Eric Piza
Marjorie Pizarro-Guajardo
Fabio Pizza
Valentina Pizzamiglio
Vittorio Pizzella
Tommaso Pizzorusso
Milos Pjanic
Dimitris Placantonakis
Christophe Place
Jean Marie S. Place
Vincent Placet
David Plachetzki
Fabio Placidi
Jesse Placone
Eric Plaisance
Beth Plale
Felix Plamper
Antonio Planchart
Julien Plancq
Gernot Plank
Lindsay Plank
Michael Plank
Benjamin Planquette
Ewan Plant
Giles Plant
Richard Plant
Laura Plantinga
Myriam Plantinga
Javier Plasencia
Dietrich Plass
Alejandro Plastina
Hervé Platel
Norman Platnick
Belinda Platt
Brennan Platt
Robert Platt
Tom Platteau
Thomas Platts-Mills
Matthias Platzer
Denese Playford
Gracyna Plaza
Sonia Plaza
Daniel Plaza-Bonilla
Sara Isabel Plazola Hernandez
Melanie Plazy
Federico Plazzi
Katherine Pleasants
Barbara Plecko
Jack Pledger

Kristen Pleil
Jürgen Pleiss
Stephanie Plenty
Nancy Pleshko
Nikolaus Plesnila
Michael Plevin
Thorsten Plewan
Marie-Laure Plissonnier
George Plitas
Maciej Plocharski
Randy Ploetz
Ekaterina Plokhova
Jaap Plomp
Alexander Ploner
Przemyslaw Plonka
Ori Plonsky
Charles Plopper
Lilian Plotkin
Sergey Plotnikov
Ingrid Plotton
Brian Plouffe
Robert Plowes
Raina Plowright
Marie-Cécile Ploy
Alessandro Pluchino
Mateusz Plucinski
Andrea Pluess
Emma Plugge
Jackie Plumbridge
Paul Plummer
Guy Plunkett
Gerd Pluschke
Tomáš Pluskal
Aleks Pluskowski
Barbara Plytycz
May Ee Png
Bian Po
Gregory J. Poarch
Jordan Pober
Dominic Poccia
Ross Poche
Oleh Pochynyuk
Derek Pociask
Roger Pocock
Péter Poczai
Andreas Podbielski
Juan Poderoso
Osvaldo Podhajcer
Jagdeep Podichetty
Rafał Podlaski
Tomaz Podlesnikar
Vedran Podobnik
Viktor Podolskiy
Frances Podrebarac
Lars Podsiadlowski
Stepan Podzimek
Jerrod Poe
Nicole Poe
Jon Poehlman
Jos Poell
Erik H. Poelman
Maartje Poelman
Geert Poelmans
Steven Poelzing
Gerhard Poelzl
Timm Poeppl
Jashvant Poeran
Sharon Poessel
Christina Poethko-Müller
Maya Poffenberger
René Poggenborg
Gabi Poggensee
Paolo Poggio
L. Poghosyan
Marko Poglitsch
Kristin Pogoda
Zenon Pogorelić
Gennady Pogorelko
Claudia Pogoreutz
Marta Pogrzeba
Brian Pogue
Catherine Poh
Geong Sen Poh
Ehmke Pohl
Theresa Pohlkamp
Mariana-Atena Poiana
George Poinar
Hendrik Poinar

Laurent Poirel
Paul Poirier
Marc Poirot
Laurent Poisson
Vincent Poitout
S. Poitras
John Pojman
Roman Pokora
Bostjan Pokorny
Norbert Polacek
Thomas Polak
Angela Polanco
Lillian Polanco
Therese Poland
Ozren Polasek
Stephen Polasky
Nina Polchaninova
Jason Pole
Tino Polen
Chiara Poletto
Michał Polguj
Andrea Poli
Giuseppe Poli
Carlos Poli De Figueiredo
Eugenia Poliakov
Anne Poliard
Richard Policht
Benjamin Policicchio
David Polidori
Maria-Cristina Polidori
Beth Polidoro
Igor Polikarpov
Alberto Polimeni
Antonella Polimeni
Kishore Polireddy
Chelsea Polis
Laura Poliseno
Pavel Polishchuk
Gianfranco Politano
Letterio Salvatore Politi
Pedro Politi
Pierluigi Politi
Ioannis Politikos
Dimitris Politis
Gustavo Politis
Cristina Polito
Federico Polito
Jessica Polito
Anastasia Politou
Karen Polizzi
Mark Polizzotto
Zvonimir Poljak
Hiram Polk
Adam Polkinghorne
Craig Pollack
Murray M. Pollack
Victor Pollak
Yehuda Pollak
Georgios Pollakis
Justin Pollara
Rachel Pollard
Tessa Pollard
Martina Pollastrini
Juergen Polle
David Poller
Aaron Pollett
Howard Pollick
Katrina Pollock
Louisa Pollock
Karen Pollok
Andrea Polo
Luca Polonio
Michael Polonsky
Riccardo Polosa
Jeffrey Polovina
Samuel Poloyac
David Polsky
Andrew Polson
Annikka Polster
Tobias Polte
Palmiro Poltronieri
Eugenia Polverini
Georgios Polychronidis
Marco Pombi
Mark Pomerantz
Emma Pomeroy
Kimball Pomeroy
Anna Pomés

Régis Pomès
Marín Pompa-García
Francois Pompanon
Alfonso Pompella
Maurizio Pompili
Laura Poms
Gerald Ponath
Aldo F. Ponce
Julia Poncela-Casasnovas
Alain Jean Poncelet
Javier Ponce-Saavedra
William Poncin
Emilie Pondeville
Peter Pongracz
Angelica Ponguta
Georgios Ponirakis
V. K. Chaithanya Ponnaluri
Krishna Ponnapalli
Koen Ponnet
Sathibalan Ponniah
Moorthy Ponnusamy
Revathi Ponnusamy
Igor Ponomarev
Vladimir Ponomarev
Antoni Pons
Patricia Pons
Roser Pons
Sylvia Pont
Roberto Pontarolo
Glauco Ponterini
Giovanni Ponti
Antonio Pontiroli
Patrizia Pontisso
Kevin Ponto
Giuseppe Pontrelli
Paola Pontrelli
Marina Ponzio
Sinlan Poo
Sudarsana Poojari
Eva Pool
Brian Poole
C. J. Poole
Charles Poole
David Poole
Emma Poole
Kate Poole
Richard Poole
Robert Poole
Liona Poon
Steven Poon
Cara Poor
Jalal Poorolajal
Lucas Poort
Toney Poovelikunnel
Kittiyod Poovorawan
Eduard Pop
Amirali Popat
Aurel Popa-Wagner
Rodica Pop-Busui
Arden Pope
D. P. Pope
Janet Pope
Karen Pope
Lizzy Pope
Matthew Pope
Richard Pope
Welkin Pope
Gerald Popelka
Gabriela Popescu
Mihail Popescu
Razvan A. Popescu
David Popham
Piotr Popik
Laura Popolo
Andrei Popov
Serguei Popov
Yury Popov
Nina K. Popova
Irena Popovic
Dejan Popovich
Kyle Popovich
Lana Popovich Maneski
Oleksandr Popovych
Jesse Popp
Jürgen Popp
Simon Poppinga
Linda Popplewell
Izabela Poprawa

Christopher Porada
José Porcel
Raphaël Porcher
David Porciani
Giuseppina Porciello
Peter Poredos
Lauren Porensky
Rachel Poretsky
Maurizio Porfiri
Grasiela Porfirio
Jahan Porhomayon
Soujanya Poria
Ali Pormohammad
Konstantinos Porpodis
Pablo Porras
Laura Porretti
Esteban Porrini
Gwendolin Porst
John Port
Alberto Porta
Camillo Porta
Marie Portail
Annabel Porté
J. Portegies
Erik Portelius
Anna Porter
Chad Porter
Craig Porter
Emily Porter
George Porter
Kathleen Porter
Lisa Porter
Thomas Porter
Weston Porter
Matthew Porteus
Bernard Portha
Carmen Portillo
José R. Portillo
Marta Portillo
Aurelio Portincasa
Piero Portincasa
Francesco Portioli
Michele Portman
Zachary Portman
David Portnoy
Laercio R. Porto-Neto
Gwendolyn Portsky
Katja Porvari
Inmaculada Posadas
Anna Posadino
Helen Poser
Karen Posey
Mario Posillico
Mason Posner
Michael Posner
Stephen Posner
Andreas Pospischil
Lourival Possani
Robert Possee
Roberta Possenti
Katherine Possin
David Post
Frank Post
Miriam Postan
Klaas Postema
Sietke G. Postema
Brunella Posteraro
Federico Posteraro
Cosimo Posth
Clara Posthuma
Catherine Postic
Guillaume Postic
Arnold Postlethwaite
Alida A. Postma
Olga Postnikova
Dmitry Postnov
Svetlana Postnova
Dragos Postolache
Teodor Postolache
Marek Postula
Caroline Pot
Gerda Pot
Daniel Potaczek
Judith Potashkin
Ines Pote
Tonia Poteat
Gilles Potel
Luciano Potena

Vasileios Pothakos
Jennifer Poti
Simon Potier
Marieke Potijk
Prasanth Potluri
Eric Potma
Elena Potokina
Giuseppe Potrick Stefani
Hans Pottel
Adam Potter
Barry Potter
Brian J. Potter
Douglas Potter
Kevin Potter
Larissa Potter
S. Steven Potter
Tamara Potter
Nicolas Pottier
Tom Pottinger
Igor Pottosin
Matthew Potts
Francois Potus
Patrice Poubelle
Stephanie Pouch
Krishna Poudel
Sushil R. Poudel
Enrique Pouget
Pierre Pouget
Odile Poulain-Godefroy
Nikos Poulakakis
Dimitra Pouli
Aloisie Poulickova
Lily Poulikakos
Lionel Poulin
Marc Pouliot
Anja Poulsen
Christian Poulsen
Lars Poulsen
Kelsey Poulston-Ellestad
Nicole Poulton
Lucie Poupel
Lluís Pouplana
Claire Pouplard
Christine Pourcel
Farshad Pourmalek
Mojgan Pourmokhtar
Spyros Pournaras
Philippe Pourquier
Geneviève Pourroy
Mohammad Pourshafie
Arash Pourtaherian
Hamed Pouryousef
Robin Pourzal
Paula Pousinha
Hossein Poustchi
Koen Pouwels
Simon D. Pouwels
Frans Pouwer
Giovanni Povero
José Antonio Pow Sang
Nick Powdthavee
Adam Powell
Arfon Powell
Bethan Powell
Bronwen Powell
Daniel Powell
Douglas Powell
Emily Powell
Frank Powell
Janet Powell
Lisa Powell
Matt Powell
Rebecca Powell
Stephen Powell
Tara Powell
David Power
Jennifer Power
Michelle Power
Robert Power
Ronika Power
Jennifer Powers
Randal Powers
Robert Powers
Sean Powers
Simon Powis
Dalva Poyares
Claire Poyart
Pratheeshkumar Poyil

D. R. Poyner
Helen Poynton
Necdet Poyraz
Jose-Luis Poza-Lujan
Josip Pozar
Alex Pozhitkov
Bruno Pozzetto
Ambra Pozzi
Paolo Pozzilli
Lucas Pozzo-Miller
Nishi Prabdial-Sing
Swayam Prabha
B. T. Prabhakar
Chandra Prabhakar
Hemanshu Prabhakar
Pavana Prabhakar
Sg Prabhakaran
Vivek Prabhakaran
Kumar Prabhash
Antony Prabhu
K. Prabhu
Varun Vijay Prabhu
Shubhangi Prabhudesai
Satria Arief Prabowo
Fiorella Prada
Joaquín M. Prada
Rolf Prade
Anubhav Pradeep
Paul Pradeep
Prachi Pradeep
Elizabeth Pradel
Francesco Pradella
Jean Philippe Pradere
Elina Pradhan
Pooja Pradhan
Prajal Pradhan
Pranil Pradhan
Rajat Kumar Pradhan
Sharat Kumar Pradhan
Mónica Pradillo
Anne Pradines
Elizabeth Prado
Ivanor Prado
Jose Prado
Maria Prado
Soizic Prado
Vania Prado
Vincente Prado
Jose Prados
Alexandra Prados-Torres
Stephanie Prady
Patrick Präg
Manuel Praga
Sivaraman Prakasam
Aishwarya Prakash
Hridayesh Prakash
Om Prakash
Punit Prakash
Siddharth Prakash
Sreelakshmi Prakash
Surya Prakesh
Niki Prakoura
Vincent Praloran
Pairot Pramual
Janine Prange-Kiel
Patricia Pranke
Olaf Prante
Giorgio Prantera
Anamika Prasad
Anil Prasad
Jitendra Prasad
Narayan Prasad
Rajendra Prasad
Rashmi Prasad
Sahdeo Prasad
Shailendra Prasad
Srinivasan Prasad
Vishal Prasad
Shikha Prashad
Kapoor Prashant
Antje Prasse
Aleix Prat
Francisco Prat
Nicolas Prat
Salome Prat
Yosef Prat
Lorenza Pratali
Morgan Pratchett

Chelse Prather
Timothy Prather
Francesca Prati
Domenico Pratico
Niken T. M. Pratiwi
María Prats
Harris Pratsinis
Kathleen Pratt
Rebekah Pratt
Wayne Pratt
Zoe Pratte
Francesco Prattichizzo
Guillem Pratx
Michal Pravenec
Leslie Pray
Doddy Prayogo
Filipe Prazeres
Martina De Freitas Prazeres
Damiano Preatoni
Ezio Preatoni
Monamorn Precharattana
Diego Preciado
Armando Preciado Babb
Sanda Predescu
Jochen Prehn
Kristin Prehn
Christine Preibisch
Sarah Preisler
Steffen Preissler
Klaus Preissner
Drazen Prelec
Melissa Premaor
R. Premathilake
Elsie Premereur
Daniel Premkumar
Andrea Premoli
Garreth Prendergast
Luke Prendergast
Lorenzo Prendini
Jessica Prenni
John Prenter
Heather Prentice
Marc Prentki
Daniel Preotiuc-Pietro
Pablo Presa
Graham Prescott
John F. Prescott
Steven Prescott
Deann Presley
Valerie G. Press
Eva Pressman
Gregg Pressman
Clive Prestidge
Catherine Preston
Christopher Preston
Dale Preston
Daniel Preston
Mark Preston
George Preti
Beulah Pretorius
Mias Pretorius
Klaus Prettner
Hans Preus
Claudia Preuschhof
Ulrich Preuss
Roberto Prevete
Marielle Prevoo
Gilles Prevost
Vincent Prevot
Anne-Caroline Prevot-Julliard
Pavel Pribyl
Catherine Price
Charles Price
David Price
John Price
Joseph Price
Lawrence Price
Lisa Price
Marianne Price
Matthew Price
Michael Price
Nathan Price
Owen Price
Rumi Price
Sarah Price
Theodore Price
Trevor Price
William Price

Sara Prickett
Todd Prickett
Frances Priddy
Brendan Prideaux
Peter Pridmore
Gregory Priebe
Janosch A. Priebe
Teresa Priego
Michael Prieler
Caitlin Pries
James Priest
Armando Fernandez Prieto
Franklyn Prieto
Iván Prieto
Alejandra Prieto Davó
Carlos Prieto-Granada
Xavier Prieur
Sean Prigge
Luce Prignano
Brian Primack
Stefany Primeaux
John Primrose
Eric Prina
Emily Prince
Jon Prince
Silvas Prince
Mariabeatrice Principi
Nicola Principi
Ashley Pringle
James Pringle
Claudia Prins
Gail Prins
R. Prins
Taco Prins
Jellina Prinsen
Linda Prinsloo
Sarah Prinsloo
Mechthild Prinz
Philip Prinz
Will Prinz
Simone Priori
Peter Prislan
P. Pristas
Aurelio Pristeri
Antonia Pritchard
David Pritchard
Chris Pritchett
Michael Pritz
Uriel Priva
Lois Privor-Dumm
Sivan Priya
Mallinath Priyadarshan
Medha Priyadarshini
Shantala Priyadarshini
Priyanka Priyanka
Aashish Priye
Anna Prizment
Martin Prlic
Ari Probandari
Chris Probert
William Probert
Alexander Probst
Charlotte Probst
Michel Probst
Thomas Probst
Rainer Probstmeier
Juergen Prochaska
Jürgen Prochaska
Edward Prochownik
Carole Proctor
Heather Proctor
Kenneth Proctor
Robert Proctor
William Procunier
Janey Prodoehl
Vânia Proença
Catherine Proenza
Rae Proeschold-Bell
Antonios Proestakis
Christoph Profanter
Antonio Profico
Thomas Proft
Heike Pröhl
Alan Proia
Marco Proietti
Joe Proietto
Laszlo Prokai
Victor Prokhorenko

Jenni Prokkola
Emmanuel Prokopakis
Christina Prokopenko
Oleg A. Prokopyev
Daniel Promislow
Jeanine Prompers
Edina Prondvai
Johannes Proost
Sebastian Proost
Chiara Prosperetti
David Prosperi
Ennio Prosperi
Hillary Protas
Meredith Protas
John Protzko
Elizabeth Proud
Malcolm Proudfoot
Christos Proukakis
Xavier Prous
Michael Prouty
Nicholas Provart
J. F. Provencher
Mariano Provencio
José Provenzano
Paolo Provenzano
Alice Mado Proverbio
Diogo Provete
Sylvain Provot
Roberta Provvedi
John Prowle
René T Proyer
Amy Pruden
Gérald Prud'homme
Timothy Pruett
Kay Prüfer
Menno Pruijm
Raimon Pruim
Beth Pruitt
Amy Prunuske
Ranjan Prusty
Janet Prvu Bettger
Christopher Pryce
Olena Prykhodko
Daniel Pryluka
Wieslaw Przybylski
Alkis Psaltis
Stelios Psarakis
Anna Psaroulaki
Martin Psenicka
Alexey Pshezhetsky
Sarah Psutka
Costas Psychalinos
Mina Psychogiou
Christopher Ptak
Aneta Ptaszynska
Christy Pu
Fang Pu
Juan Pu
Shenghong Pu
Yi Pu
Yonglin Pu
Steve Publicover
Annibale Puca
Antonio Puccetti
Angela Pucci
Giacomo Pucci
Rosana Puccia
Gianluca Pucciarelli
Nancy Puccinelli
Elizabeth Puchhammer-Stöckl
Andrew Pucker
Haridas Pudavar
Paolo Emilio Puddu
Alexander I. Pudovkin
Sébastien Puechmaille
Luis Puelles
Angela Puente
Esteban Puentes-Rosas
Thomas Pufe
Irina Pugach
Ivan Puga-Gonzalez
Nicolas Pugeault
D. J. Pugh
Meredith Pugh
Alberto Pugliese
Andrea Pugliese
Emanuele Pugliese
Giuseppe Pugliese

Guilherme Pugliesi
Edoardo Puglisi
Fabio Puglisi
Lorenza Pugni
Lluís Puig
Manel Puigcerver
Montse Puigdelloses
Lorri Puil
Bénédicte Puissant-Lubrano
Giulia Puja
Claude Pujol
Flor Pujol
Merardo Pujol
Enriqueta Pujol-Ribera
Miha Pukl
Ammi Pulagam
Lakshmi Pulakat
Surya V. S. R. K Pulavarti
Joyce Pulcini
Erdem Pulcu
Julie Pulerwitz
Oorvashi Puli
Manuel Pulido
Pablo Pulido
Irene Pulido-Valdeolivas
Mrudula Pulimi
Nisha Pulimood
Vinesh Kumar Puliyappadamba
Jacob Puliyel
Sanja Puljas
Filippo Pullara
Matthew Pullen
Nicholas Pullen
Pim Pullens
Christian Puller
Mohammad Pulok
Alfredo Pulvirenti
Andrea Pulvirenti
Lidia Pulz
Denise Pumain
René Pumain
Suzie Pun
Sachin Pundhir
Anna Punga
Kumaradevan Punithakumar
Noelani Puniwai
Mark Punke
Rajesh Punn
David Punt
Michiel Punt
Claudio Punzo
Fulvio Pupilli
André Pupo
Vanja Pupovac
Swati Puranik
Fredrick Purba
Brad Purcell
Jennifer Purcell
Jessica Purcell
Larry Purcell
Steven Purcell
Phil Purdy
Adrian Puren
Deirdre Purfield
Eduardo Purgatto
Ankit Puri
Basant Puri
Rajesh Puri
Yanina Purim-Shem-Tov
Mark Purnell
Kristen Purrington
Bethan Purse
Zachary Pursell
Autun Purser
Edward Purssell
Tertia Purves-Tyson
Ian William Purvis
Richard Purvis
Andreas Püschel
Smruti Pushalkar
Michael Puskarich
Krzysztof Puszynski
Christian Puta
Rohan Puthran
Vesa Putkinen
Breanna Putman
Timothy Putman
Robert Putnam

Tracy Putoczki
Karann Putrevu
Nicolaas Puts
Sumanth Putta
Radhika Puttagunta
Joanna Putterill
Thomas Püttker
Christian Puttlitz
Santhoshkumar Puttur
Juha Puustinen
Laurent Puy
Antonio Puyet
Ana Puyo
Rami Puzis
Gilbert Pwiti
Margo Pybus
Timothy Pychyl
David Pye
Thomas Pyka
Alyssa Pyke
Kevin Pyke
Laura Pyle
Richard Pyle
Magdalini Pylli
Carly Pymont
Jeffrey M. Pyne
S. Pyo
Thidar Pyone
Mark Pyron
Robert Pyron
David Pyrooz
Junaid Qadir
Sami Ullah Qadir
Fatimunnisa Qadri
Firdausi Qadri
Syed Qadri
Matin Qaim
Mahwish Qamer
Khaled Qanud
Atif Qasim
Dima Qato
Bozhao Qi
Chao Qi
Chengzhi Qi
Dunwu Qi
Geqi Qi
Guanghai Qi
Haigang Qi
Hongsheng Qi
Ji Qi
Qingsheng. Qi
Ruifeng Qi
Shaohai Qi
Shutao Qi
Sun Qi
Wei Qi
Xiang Qi
Xiaowen Qi
Xin Qi
Xingshun Qi
Xue-Bin Qi
Yang Qi
Yi Qi
Yongjun Qi
Yun Qi
Cheng Qian
Feifei Qian
Feng Qian
Hong Qian
Jiaming Qian
Jingjing Qian
Jun Qian
Li Qian
Men-Bao Qian
Pei-Yuan Qian
Shuiming Qian
Wei-Jun Qian
Xiaoning Qian
Yongqiang Qian
Yongzhong Qian
Yu Qian
Yuan Qian
Zhijian Qian
Zichen Qian
Cui Qiang
Yan Qiang
Zhang Qiang
Aike Qiao

Ge-Xia Qiao
Jie Qiao
Mingqiang Qiao
Yuan Qiao
He Qiaoning
Gong Qi-Hai
Bo Qin
Fu Qin
Fujun Qin
Hongmin Qin
Hua Qin
Hua-Li Qin
Jichao Qin
Jing Qin
Jun Qin
Kai-Rong Qin
Pengmin Qin
Qing-Ming Qin
Qiwei Qin
Ruijun Qin
Weiping Qin
Wen Qin
Yuanyuan Qin
Yun Qin
Zhihai Qin
Hong Qing
Qin-Min Ge
Chao Qiu
Chenxi Qiu
Fan Qiu
H. Qiu
Hongyu Qiu
Hua-Ji Qiu
Huan Qiu
Huijuan Qiu
Jiangping Qiu
Jing Qiu
Junzhi Qiu
Lihua Qiu
Ling Qiu
Ruoyi Qiu
Shenfeng Qiu
Shuiqing Qiu
Tian Qiu
Wang-Ren Qiu
Wei Qiao Qiu
Weiliang Qiu
Yafeng Qiu
Yingwei Qiu
Yongzhi Qiu
Yueming Qiu
Yujie Qiu
Zhi-Cheng Qiu
Zhiying Qiu
Bo Qu
Cunmin Qu
Hong Qu
Hongxia Qu
Jiuhui Qu
Meng Qu
Mingnan Qu
Shaohong Qu
Shaojian Qu
Xian Qu
Xiaobo Qu
Xinhua Qu
Xinshun Qu
Yanyan Qu
Zhiguo Qu
Zhilin Qu
Marianne Quaas
Jon Quach
Truyen Quach
Joachim Quack
Katharina Quack Lötscher
Lindi Quackenbush
Sandra Quackenbush
Mohammed Quader
Manfredo Quadroni
Fabio Quaglino
Emilio Quaia
Matthew Quaife
Chao Quan
Hude Quan
Lijun Quan
Taihao Quan
Vanessa Quan
Xiaobo Quan

Jane Quandt
Dong Quanjiang
Luciano Quaranta
Mario Quaresma
Chad Quarles
Luca Quaroni
Raquel Quatrini
Mattia Quattrocelli
Aldop Quattrone
Cassandra Quave
Paul Quax
Wim Quax
Jenny Que
Loretta Que
Youxiong Que
B. N. Queenan
Joao Queirós
Odília Queirós
Bernardo Queiroz
Frederick Quelle
Gwenole Quellec
Luis Queme
Josep Quer
Isabelle Quere
Uwe Querfeld
Grazia Marina Quero
Amparo Querol
Alberto Quesada
Antonio Quesada
Humberto Quesada
Peter Quesenberry
Francois Quesque
Jennifer Questel
Christophe Queval
Filipe Quevedo-Silva
Catherine Quiblier
Jacob A. Quick
Nicola Quick
Donald Quicke
David Quigley
Maryka Quik
Joaquín Quílez
Thibaut Quillard
Laura Quilter
Lena Quilty
Juan Pablo Quimbayo
Muriel Quinet
Catherine Quinlan
Ashlinn Quinn
Catherine Quinn
Claire Quinn
Elizabeth Quinn
Frederick Quinn
James Quinn
Joseph Quinn
Liam Quinn
Mark Quinn
Michael Quinn
Patrick Quinn
Carlos Quiñonez
Daniel Quintana
Eric Quintane
Rodrigo Quintanilla
María Quintela
Andrea Quintero
Isabella Quinti
Cristiano Quintini
Emerson Quintino
Gina Quirarte
Maria Quiroga
Mariangeles Quiroga
Marcela Quiroz Castellanos
Lucia Rita Quitadamo
Ursula Quitterer
Abdul Rashid Qureshi
Faisal Qureshi
Kashif Qureshi
Rahat Qureshi
Zahida Qureshi
Ajay Kumar R.
Esperanza R. Matarredona
Eric Raabe
Mohammad Raad
Dina Raafat
Alexander Raaijmakers
Walter Raasch
Truls Raastad
Susan Raatz

Victoria Rabago
Lydia Rabbaa Khabbaz
Shafaat Rabbani
Rudy Rabbinge
Patrick Rabbitt
Sarah Rabbitt
Gernot Rabeder
Luiza Rabelo
Jacob Raber
Nicolas Rabet
Ryan Rabett
Yacov Rabi
Yoed Rabin
Tal-Chen Rabinowitch
Jeremy Rabinowitz
Peter Rabinowitz
Samuel Rabkin
R. Taylor Raborn
Daniel Rabosky
Marco Rabuffetti
Ramon Rabuñal
Ralf Rabus
Gwénaël Rabut
Graham Raby
Lee Raby
Judith Racape
Denis Raccah
P. Sivaramakrishna Rachakonda
Sudhir Rachapelle
Aissaoui Rachid
Tamiris Rachid
Dominik Rachon
Daniela Raciti
Gregory Raciti
Girts Racko
Darius Rackus
Lorraine Racusen
Cristina Rada
Marco Rada
Ante Rađa
Giovanni Radaelli
Zslot Radak
Valery Radchenko
Fiona Radcliff
Tiffany Radcliff
Richard Radcliffe
Jeffrey Rade
Aleksandra Radenovic
Daniel Rader
Jenny Radesky
Andrew Radford
Devesh Radhakrishnan
Jeejabai Radhakrishnan
Krishanakumar Radhakrishnan
Prakash Radhakrishnan
Ravi Radhakrishnan
Karin Rådholm
Marko Radic
Mathilde Radiguet
Madeleine Rådinger
Predrag Radivojac
Jelena Radjenovic
Sina Radke
Rafael Radkowski
Amir Reza Radmard
Balazs Radnai
Ivana Radojevic
Predrag Radović
Milan Radovich
Tamás Radovits
Markus Radsak
Jan Radtke
Beatrice Radu
Joaquim Radua
Katarina Radulovic
Igor Radun
Ibrahim Radwan
Osman Radwan
Svetlana Radyuk
Danuta Radzioch
Mick Rae
Kenneth Raedeke
Robert Raeside
Lori Raetzman
Alex Rafacho
Rodrigo Rafagnin Duarte
Marjan Rafat
Hershel Raff

Giuseppe Raffa
Kenneth Raffa
Robert Raffai
Thomas Raffel
Elena Raffetti
Francesca Raffini
James Raftery
Arthur J. Ragauskas
A. Raggi
Alberto Raggi
Chiara Raggi
Derek Raghavan
Rahul Raghavan
U. Raghavendra
Vinay Raghavendra
K. Raghavendra Kumar
Karthik Raghunathan
Raj Raghupathy
John Ragland
Laura Ragni
Margaret Ragni
Pia Ragno
Sergio Ragonese
Romain Ragonnet
Manon Ragonnet-Cronin
Michael Ragosta
Michael Ragozzino
Minvydas Ragulskis
Robert Raguso
Sandeep Raha
Elias Rahal
Omar Rahal
Abdul Hakkim Rahamathullah
Jamshid Raheb
Adeeba Raheem
Chantal Raherison
Mika Rahikainen
Abdolrasol Rahimi
Hamid Reza Rahimi
Nader Rahimi
Roshanak Rahimian
Abdul Rahman
Habibur Rahman
Hamid Rahman
Irfan Rahman
Kh Shamsur Rahman
M. Shafiqur Rahman
Mahfoozur Rahman
Mahmudur Rahman
Mijanur Rahman
Mohamed Farouk Rahman
Mohammad Tariqur Rahman
Mustafizur Rahman
Nafis Rahman
Nisar Rahman
Shadab Rahman
Shams Rahman
Twyeafur Rahman
Zia Rahman
Mehran Rahmani
Omid Rahmani
Rasoul Rahmani
Arman Rahmim
David Rahn
Amit Rai
Arti Rai
Ashutosh Rai
Dipti Rai
Kirthikumar Rai
Lavanya Rai
Padmalatha Rai
Priyamvada Rai
Rajivnandan Rai
Shalu Rai
Tatemitsu Rai
Umesh Rai
Saša Raicevich
Shane Raidal
Leopoldo Raij
Abbie Raikes
Juste Raimbault
Kaitlin Raimi
Claudio Raimondi
Lavinia Raimondi
Sandy Raimondo
Stefania Raimondo
Soom Raina
Pascal Rainard

Michael Rainbow
Peter Rainer
Douglas Raines
Tom Rainey
George Rainger
Jesica Raingo
Pierre Rainville
Soroush Rais-Bahrami
Nicholas Raison
Kirsimarja Raitasalo
Dan Raiten
Stefan Raith
Simo Raittila
Neeraj Raizada
Rahul Raj
Sudha Raj
Huzefa Raja
Priya Raja
Mehdi Rajabi
P. Rajadurai
Sudarshan Rajagopal
Bhaskaran Rajagopaplan
Anuradha Rajamanickam
Nalini Rajamannan
Neil Rajan
Suja S. Rajan
Krishnamurthy Rajanikant
Hema Rajaram
Murugesan Rajaram
Narasimhan Rajaram
Ramaprasad Rajaram
Satwik Rajaram
Kanshukan Rajaratnam
Ram Rajasekharan
Radha Rajasingham
Arumugam Rajavelu
Sreekumari Rajeev
Singh Rajender
Ganeshkumar Rajendran
K. V. Rajendran
Pradeep Rajendran
Ranjith Rajendran
Pandiyan Rajesh
Mirjana Rajilić-Stojanović
Kenan Rajjoub
T. K. Rajkhowa
K. Rajkumar
S. Rajkumar
Praveen-Kumar Raj-Kumar
Nonika Rajkumari
Rajendram Rajnarayanan
Ann Rajnicek
Edwin Rajotte
Charu Rajput
Kiransinh Rajput
Kishore Rajput
M. Rajput
Sandeep Kumar Rajput
Aj Solomon Raju
Tonse Raju
Mihir S. Rajurkar
Jyutika Rajwade
Stefan Rakete
Gábor Rákhely
Natella Rakhmanina
Seyed Ali Rakhshan
Timothy Rakitan
Rafał Rakoczy
Tebogo Rakola
Jasna Rakonjac
Alexey Rakov
Archayeeta Rakshit
Randeep Rakwal
Catherine Raley
Loukianos S. Rallidis
Stephen Ralph
Mark Ralston
Bali Ram
Nilam Ram
Pavani Ram
Suresh Rama Chandran
Nagaraja Reddy Rama Reddy
Raja Ramachandran
Shyam Ramachandran
Surya Ramachandran
Rabie Ramadan
Habib Ramadhani
Pierluigi Ramadori

Pamela Ramage-Morin
Nagappa Ramaiah
S. Ramakrishnan
Siddharth Ramakrishnan
Sreejith Ramakrishnan
Srinivasan Ramakrishnan
João Ramalho-Santos
Murugan Ramalingam
Satish Ramalingam
Suresh Ramalingam
Ayyalusamy Ramamoorthy
Mahesh Ramamoorthy
Prabhu Ramamoorthy
Senthil Ramamurthy
Jaishree Raman
Karthik Raman
Lakshmi Raman
Vijayasankar Raman
Jayashree Ramana
Venkata Ramana
Sasanka Ramanadham
Arvind Ramanathan
Babu Ramanathan
Gunasekaran Ramanathan
Lalini Ramanathan
Mala Ramanathan
Murali Ramanathan
Parameswaran Ramanathan
Seetha Ramanathan
Sasirekha Ramani
Manjunath Ramanjaneya
Sri Ramarathinam
Rajesh Ramasamy
Ranjith Ramasamy
Thiruganesh Ramasamy
Anand Ramasubramanian
Mani Ramaswami
Amutha Ramaswamy
Anant Ramaswamy
Sharan Ramaswamy
Vijay Ramaswamy
Stefano Ramat
Reshmie Ramautarsing
T. Ramayah
Ashwin Ramayya
Alessandro Rambaldi
Ramugondo Rambau
Nirala Ramchiary
Sahienshadebie Ramdas
Anne Ramelet
Sara Ramella
N. Ramesh
Shunmugiah Ramesh
Vijay Ramesh
Tanjona Ramiadantsoa
Christophe Ramière
Cody Ramin
Darioc. Ramirez
Gerardo Ramirez
Ivan Ramirez
Joel Ramirez
Julia Ramirez
Marie Ramirez
Mário Ramirez
Marizen Ramirez
David Ramírez
Julia Ramírez
María Soledad Ramírez
Ricardo Ramirez
Marina Ramirez-Alvarado
Ricardo Ramirez-Gonzalez
Ignacio Ramírez-González
Monica Ramirez-Mella
Ivan Ramirez-Morales
Alfredo Ramírez-Reveco
Ricardo Ramirez-Romero
Robinson Ramírez-Vélez
Sofia Ramiro
Guillermo Ramis
Jacqueline Ramke
Doraiswami Ramkrishna
Vickram Ramkumar
David Ramler
Padmanabhan Ramnarayan
Vundli Ramokolo
Santiago Ramon y Cajal
Alberto Javier Ramos
Beatriz Ramos

Cayo Ramos
Feljandro P Ramos
Fernando Ramos
Henrique Ramos
Jorge Ramos
Joyce Ramos
María Concepción Ramos
Marina Ramos
Raül Ramos
Sara Ramos
Sonia Ramos
T. B. Ramos
Ana Ramos Brito
Maria Carmen Ramos-Herrera
Priscila Ramos-Ibeas
Erivan Ramos-Junior
G. Ramos-Mandujano
Michal Ramot
Dindial Ramotar
Karam Ramotar
Lekhraj Rampal
Elena Rampanelli
Simone Rampelli
Giordano Rampioni
Luca Rampoldi
Jennifer Ramsay
John Ramsay
Jonathan Ramsay
Rona Ramsay
David Ramsden
John Ramsey
Jon Ramsey
Kathryn Ramsey
Stephen Ramsey
Vijayalakshmi Ramshankar
Chava Ramspek
Kristina Ramstad
Pramod Ramteke
Muhammad Ramzan
Maosheng Ran
Sophia Ran
Xingwu Ran
Aadia Rana
Ashma Rana
Jamal Rana
Muhammad Afzal Rana
Neha Rana
Sarosh Rana
Sohel Rana
Senaka Ranadheera
Isuru Ranasinghe
Giulia Rancati
Tiziana Rancati
Debbie Rand
Kenneth Rand
Troy Rand
Cameron Randall
Paul Randazzo
Morten Randers
Matthew Randesi
Gurch Randhawa
Adriane Randolph
Tendry Randriamanana
Elko Randrianarisoa
Armand Randrianasolo
Chetan Rane
Vinita Rane
Lisa Ranford-Cartwright
René Ranftl
Molebogeng Rangaka
Padmini Rangamani
Anna Rangan
Ajay Rangaraj
Srikant Rangaraju
Tirumalai Rangasamy
Ryan Range
Marisa Rangel
Claudia Rangel-Escareño
Xie Rangjin
Vivek Rangnekar
Asha Rani
P. Rani
Danilo Ranieri
Elena Ranieri
Girolamo Ranieri
Nicola Ranieri
Aashish Ranjan
Ashish Ranjan

Ravi Ranjan
Louis Ranjard
Sree Ranjini K. S.
Manoranjan Ranjit
Nalini Ranjit
Pathegama Gamage Ranjith
Sailesh Ranjitkar
Sarbin Ranjitkar
Claudia Rank
Renate Ranka
Kenneth Rankin
Kristin Rankin
Robert Rankin
Sally Rankin
Shelley Rankin
Vinisha Ranna
Fabrice Rannou
Brigitte Ranque
Jason Ransom
Yusuf Ransome
Barbara Rantner
Marco Ranucci
Laura Ranum
Elia Ranzato
Andrea Ranzi
Aparna Rao
Arvind Rao
Basuthkar Rao
Bindumadhava Hanumantha Rao
Chinthalapally Rao
Christopher Rao
Chythra Rao
Guillaume Rao
Hua-Xiang Rao
Jianyu Rao
K. S. Rao
Ke Rao
Kottapalli Rao
Krishna Rao
Mangala Rao
Mrinalini Rao
Nalam Rao
Neena Rao
P. Rao
Panduranga Rao
Pulivarthi Rao
Raveendra K. Rao
Sarika N. Rao
Satyanarayana Rao
Sheng-Xiang Rao
Sridhar Rao
Sudhakar Rao
Suresh Rao
Timsi Rao
V. Ashutosh Rao
Xiancai Rao
Xiang-Jun Rao
Yunbo Rao
Rekha Rao Manepalli
Didier Raoult
Otto Rapalino
Penny Rapaport
Ronald Rapee
Lauri Rapeli
Dan Raper
Claudio Rapezzi
Dennis Raphael
Ho Tsong Fang Raphael
Jacques Raphael
Andrea Rapisarda
Giammarco Raponi
David Rapoport
Edward Rapp
Thomas Rapp
Francesca Rappa
Bernhard Rappenglueck
Daniel Rappolee
Nadav Rappoport
Tom Raptakis
Vassilios Raptis
Rubhana Raqib
Kevin Rarick
Avinash A Rasalkar
Elena Raschi
Anne Rascle
Ericka Rascon
Davide Rasella

Natalie Rasgon
Awais Rasheed
Muhammad Usman Rasheed
Muneera Rasheed
Benjamin Rashford
Sajid Rashid
Zulqarnain Rashid
Armin Rashidi
Mohammad Mehdi Rashidi
Parisa Rashidi
Hasan Rashidul
Gordana Rasic
Girish Rasineni
Mette Rask
Charlotte Rasmussen
Jon Rasmussen
Kirsten Rasmussen
Laura Rasmussen
Martin Krøyer Rasmussen
Peter Rasmussen
Svein Rasmussen
Mark Rasnake
Vagner Raso
Voahangy Rasolofo
Razanamparany
Aida Hanum Rasool
Raihan Rasool
Hassan Rasouli
Federico Raspini
Tienush Rassaf
Francois Rassendren
Diego Rasskin Gutman
Craig Rassmusen
Gurdeep Rastogi
Giulia Rastrelli
Rolando Rasua
Eva-Maria Ratai
Janina Ratajczak
Laurene Ratcliffe
Michael Ratcliffe
Norman Ratcliffe
Andrew Rate
Hennie Raterman
Martin Rath
N. Rath
Timo Rath
Sushma Rathaur
Phil Rather
Anandharajan
Rathinasabapathy
G. Rathinasabapathy
Sujit Rathod
Ravindranath Rathore
Aivaras Ratkevicius
Katherine Ratliff
Oliver Ratmann
Sujeevan Ratnasingham
Tõnu Rätsep
Raffaele Ratta
Rahul Rattan
Satish Rattan
Ekarat Rattarittamrong
Niels Rattenborg
Bianca Ratti
Pietro-Luca Ratti
Veronica Rattini
Matt Ratto
Linda Raubeson
Frank Rauch
Daniel Raudabaugh
Marjatta Raudaskoski
Terje Raudsepp
Patrick Raue
Katrin Rauen-Eberhardt
Ijaz Rauf
Anne Rauh
Michael Raupach
Steffen Rausch
Alexander Rauscher
Emily Rauscher
Emily Rauschert
Estrella Rausell
Hilpi Rautelin
Riina Rautemaa-Richardson
Erwan Rauwel
Marta Rava
Nisarg Raval
Amir Ravandi

Vineesh Raveendran
John Raven
Stephen Raverty
C. Ravetti
Anita Ravi
Rohit Ravi
Magalie Ravier
Andrea Ravignani
Shruthi Ravimohan
Nikolai Ravin
Kadiyala Ravindra
Sundari Ravindran
Raffaella Ravinetto
Jonathan Raviotta
Kundapura Ravishankar
Srinath Ravulaparthy
Lal Rawal
Shruti Rawal
Rajdeep Singh Rawat
Ann Rawkins
Nic Rawlence
Stephen Rawlinson
William Rawlinson
Alan Rawls
Ann Ray
Bappaditya Ray
Chris Ray
Debabrata Ray
Debashree Ray
Debjit Ray
Evan Ray
Isha Ray
Krishanu Ray
Prabir Ray
Samiran Ray
Stuart Ray
Supriyo Ray
Swayamjit Ray
William Ray
Wilson Ray
Shaon Ray Chaudhuri
Helene Raybould
Siba Raychaudhuri
Mary Katherine Rayens
Yordan Raykov
Kasie Raymann
Jonathan Rayment
H. Raymond
Jason Raymond
Josette Raymond
Leigh Raymond
Olivier Raymond
Laurie Raymundo
Agathe Raynaud Simon
Emmanuel Rayner
Julian Rayner
Yevgeniy Raynes
Annette Raynor
Catherine Rayon
Holly Rayson
Aeyal Raz
Itamar Raz
Lauren Raz
Sarah Raz
Kaisar Raza
Shahid Raza
Shahzad Raza
Syed Muhammad Raza
Khaleel Razak
Babak Razani
Neda Razaz
Ahmed Razek
Alim Al Islam Razi
Luis Razon
Raymund Razonable
Olga Razorenova
Faterneh Razzaghi
Abdul Razzaq
Maria Razzoli
Angela Re
David Rea
Dale Read
Heather Read
Laurie Read
Morgayn Read
Scott Read
Karen Reader
Adam Readhead

Paul Ready
Michaela Reagan
Raimundo Real
Marcella Reale
Catherine Reardon
Cillian Reardon
Claudia Reardon
Eduardo Reategui
Diego Reátegui
Annabelle Reaux Le Goazigo
Drury Reavill
Kelly Reavis
Amanda Rebar
Trudy Rebbeck
Richardson Rebecca
Joseph Rebehmed
Peter Rebeiro
Hugo Rebelo
Laurent Reber
Paul Reber
Stefan Reber
Robert Rebhun
Terri Rebmann
Michael Rebold
Eria Rebollar
Boris Rebolledo-Jaramillo
Laura Rebollo-Neira
Alberto Rebonato
Sergio Recalde
Charlotte Recapet
Marc Recasens
Alessandra Recchia
Maria Cristina Recchioni
Lawrence Recht
Jan Recker
Ana Recober
M. Thomas Record
Nick Record
Sergio Recuenco
Ahmed Reda Ahmed
Ulrike Redanz
Lavanya Reddivari
Hudson Reddon
Ashvini Reddy
Bhargava Reddy
Kasu Prasad Reddy
Narsimha Reddy
Pavankumar N. G. Reddy
Pavankumar Reddy
Rishindra Reddy
Sai Reddy
Sudhakar Reddy
Umesh Reddy
Frank Redegeld
Elizabeth Redente
Robert Redfield
Melissa Redford
Thomas Redick
Christoph Redies
Dawn Reding
Katherine Reding
Klaus Redmann
Anthony C Redmond
Molly Redmond
T. Michael Redmond
Edilberto Redona
Maximino Redondo
Maria Redowicz
Maggie Redshaw
Gerald Redwine
Simon Redwood
Bethany Reeb-Sutherland
E. Albert Reece
Michelle Reece
Dan Reed
Douglas Reed
J. Reed
Kent Reed
Maureen Reed
Michael Reed
Rebecca Reed
Robert Reed
Tanea Reed
Brian Reed Silliman
Glenn Reeder
Leslie Reeder-Myers
Michael Reedijk
Brian Reedy

F. Reen
Alan Rees
Bernard Rees
Christopher Rees
Elliott Rees
Harold Rees
Megan A. Rees
Michael Reese
Koen Reesink
Tim Reeskens
Johnmarshall Reeve
Robert Reeve
Edward Reeves
Emma Reeves
Gregory Reeves
Lawrence Reeves
Patrick Reeves
R. Keith Reeves
Rachel Reeves
Marwan Refaat
Tamer Refaat
Yassin Refahi
Guislaine Refrégier
Daniela Rega
Tarik Regad
Annette Regan
Matthew Regan
Raymond Regan
Scott Regenbogen
Jg Regensteiner
Gili Regev-Yochay
Carlo Reggiani
Santos Regiane
Terry Regier
Jean-Marc Regimbeau
Binod Regmi
Jean-Luc Regnard
Eduardo Rego Reis
Francesco Regoli
Giuseppe Regolisti
Adrian Regos
Noemí Reguart
Wail Rehan
Marit Rehavi
Sebastian Rehberg
Jen Rehg
David Rehkopf
Jurgen Rehm
Philipp Rehm
Ateequr Rehman
Shafiq Ur Rehman
Yasir Rehman
Mubashir Husain Rehmani
Therese Rehn
Ashish Rehni
Gilbert Reibnegger
Kristian Reich
Nick Reich
Martin Reichard
Amy Reichelt
Malte Reichelt
Bettina Reichenbacher
Matthias Reichert
Sophie Reichert
Thomas Reichert
Philipp Reicherts
Nadine Reichhart
Udo Reichl
Robert Reichle
Jerome Reichman
Florian Reichmann
Jonathan Reichner
Brian Reichow
Natalia Reich-Stiebert
Marla Reicks
Christopher Reid
Gregor Reid
J. Spence Reid
Rachel Reid
Russell Reid
St. Patrick Reid
Tony Reid
Matthew Reidenbach
Daniel Reidpath
Christiane Reif
Jiří Reif
Julian Reif
Kathryn Reif

Lennart Reifels
Elizabeth Reifsnider
Maria Reig
Sijmen Reijneveld
Martin Reijns
Kathleen Reilly
Peter Reilly
Colin Reily
James Reimer
Richard Reimer
Karen Reimers
Stian Reimers
Joshua Rein
Robert Rein
Theo Rein
Ramses Reina
Peter Reinach
Maria Reinares
Ester Reina-Romo
A. A. T. Simone Reinders
Ilse Reinders
Holger Reinecke
Annette Reineke
Zeljko Reiner
Michael Reinert
Friedemann Reinhard
Karl Reinhard
Marc-André Reinhard
Matthias Reinhard
René Reinhard
Christoph Reinhardt
Dieter Reinhardt
Keith Reinhardt
Klaus Reinhardt
Richard Reinhardt
Crystal Reinhart
Martin Reinhart
Claudia Reinheimer
Petra Reinhold
Ingars Reinholds
Finn Reinholt
Ulrich Reininghaus
Milan Reinis
Aaron Reinke
Lester Reinke
Sebastian Reinstadler
Vytas Reipa
Simone Reiprich
Adelina Reis
Catarina Pinto Reis
Flavio Reis
Jaques Reis
Katarina Reis
Pedro Reis
Renata Reis
Roberto Reis
Stefan Reis
Tânia Reis
Victor Reis
Gabriela Reis-Avila
Bruce I. Reisch
William Reisen
Ryan Reisinger
Michael Reiskind
Benjamin Reisman
Pedro Reis-Rodrigues
N. Reiss
Monika Reissmann
Alexander Reiter
Eric Reiter
Guido Reiter
Nicholas Reiter
Rolf Reiter
Maarten Reith
Eric Reits
Katarzyna Rejniak
Lars Rejnmark
Åsa Rejno
Jose Del Carmen Rejon-Orantes
Romdhane Rekaya
Daniel Reker
Islem Rekik
Mourad Rekik
Mohamed Rela
Giulio Relini
Rajko Reljic
Christian Rellstab
Alan Remaley

Anthony Remaud
Katja Rembold
Eumorphia Remboutsika
Thomas Remer
Constanze Remi
Fernando Remião
Daniel Remias
Daniel Remick
David Remington
Carol Ann Remme
Daniel Remondini
Torge Rempe
David Rempel
Polychronis Rempoulakis
Andrew Remppis
Baiping Ren
Bin Ren
Bingzhong Ren
Clement Ren
Dacheng Ren
Deyong Ren
Dongwei Ren
Feng Ren
Gang Ren
Guangjun Ren
Han Ren
Hangxing Ren
Haoyu Ren
Jian Ren
Jiangong Ren
Jiangtao Ren
Jianguo Ren
Jie Ren
Jinchang Ren
Jun Ren
Kewei Ren
Lihai Ren
Monica Ren
Pei-Gen Ren
Pingping Ren
Qian Ren
Qiang Ren
Qingzhong Ren
Quansheng Ren
Shan Ren
Shuxin Ren
Suping Ren
Tao Ren
Tianheng Ren
Tiantian Ren
Xiobao Ren
Yi Ren
Yinshi Ren
Zheng-Gang Ren
Zhuoming Ren
Bernhard Renard
Francoise Renard
Seracchioli Renato
Gabriel Renaud
Marek Renco
Alan Rendall
Elizabeth Rendina
William Rendu
Andrew Renfree
Lindsay Renfro
Srinivasan Rengachari
Aravind Kumar Rengan
Deivendran Rengaraj
Zed Rengel
Georges Reniers
Frank Renkewitz
Dimitri Renmans
Stephen Rennard
Elisha Renne
Elizabeth Renner
Ian Renner
Lars Renner
Simone Renner
Elissa Rennert-May
Benjamin Renoust
Mark Renshaw
Ronald Rensink
Chiara Renso
Rebecca Rentea
René Rentería
Fabian Rentzsch
Julie Renwick
Jean-Luc Reny

Diane Renz
Elizabeth Renzoni
Martijn Rep
Elizabeth Repasky
Ombretta Repetto
Jussi Repo
Maria Repolles
Irene Reppa
Jose Requena
Natalia Requena
Vincent Requillart
Patricia Resa-Infante
Geir Resaland
Jaime Resano-Mayor
Pierre-Yves Rescan
Bernd Resch
Bernhard Resch
Jacob Resch
Markus Resch
Leslie Rescorla
Renan Resende
Moises Resende Filho
Salvador Resino
Andrew Resnick
David Resnik
Linda Resnik
Michael Restivo
Claudia Restrepo
Diego Restrepo
Joseph Restuccia
Marc Restuccia
Mauricio Retamal
Michael Rettig
Oliver Rettig
Rainer Rettig
Mallikarjuna Rettiganti
Eva Rettinger
Ruben Retuerto
Bradley Reuhs
Philipp Reuken
Stefan Reuscher
Sebastian Reuter
Christina Reuterskiold
Oliver Reuthebuch
Heiko Reutter
Gunturu Revathi
Kelly Reveles
Carlo Reverberi
Massimo Reverberi
Bruno Revol
Sergei Revskoy
Ana Rey
Gregoire Rey
Rodolfo Rey
Jose Rey Benayas
Hugh Reyburn
Fernanda Reyes
Josephine Reyes
Gustavo A. Reyes del Paso
Constantino Carlos Reyes-Aldasoro
Hugo Reyes-Centeno
Armando Reyes-Engel
Victoria Reyes-García
Carlos Reyes-Moreno
Enrique Reyes-Muñoz
Paul Reyfman
Christine Reyna
Kelly Reyna
Enrique Reynaud
Harmen Reyngoudt
Albert C. Reynolds
Albert Reynolds
Andrew Reynolds
Arthur Reynolds
Clare Reynolds
Harmony Reynolds
Heidi Reynolds
Joshua Reynolds
Julie Reynolds
Kristin Reynolds
Laura Reynolds
Lawrence Reynolds
Leryn Reynolds
Lisa M. Reynolds
Matthew Reynolds
Nick Reynolds
Olivia Reynolds

Staci Sue Reynolds
Susan Reynolds
Todd Reynolds
Ana Rey-Rico
Alexander Reyzelman
Negar Rezaei
Sajad Rezaei
Pouya Rezai
Enrico Rezende
Paulo Rezende
R. A. Rezende
Martin Reznek
David Reznick
Sandra Reznik
Faisal Rezwan
Nasser Rezzoug
Christopher Rhea
Elizabeth Rhea
Sarah Rhea
Jay Rhee
Juong Rhee
Soo Rhee
Taeho Greg Rhee
Maikel Rheinstadter
Haroun Rhemtula
Mijke Rhemtulla
Mikael Rhen
Nick Rhind
William Rhine
Sophia Rhizopoulou
Kimberly Rhoades
Douglas Rhoads
William Rhoads
Corinne Rhodes
Edward Rhodes
Lesley Rhodes
Sara Rhodes
Shelley Rhodes
Marie Rhodin
Mohamed Rholam
Jack Rhyan
Andrew Rhyne
Mee-Ra Rhyu
Leila Riahi
Kristy Rialon
Summaira Riaz
Cecilia Ribalaygua
Catalina Ribas
José Ribas Fernandes
José Ribas-Fernandes
Heather Ribaudo
Admilson Ribeiro
Claudia Ribeiro
Claudio Tadeu Daniel Ribeiro
Fabiola Ribeiro
Haroldo Ribeiro
Joana Ribeiro
José Ribeiro
Laura Ribeiro
Marcela Xavier Ribeiro
Marcelo Ribeiro
Maria Margarida Ribeiro
Patricia Ribeiro
Bruna Ribeiro de Andrade Ramos
José Ribeiro Neto
Ana Ribeiro Rodrigues
Anderson Ribeiro-Carvalho
Rejane Ribeiro-Rotta
Maurizio Ribera d'Alcala
Werner Ribitsch
Jérôme Ribot
Angel Ric
Mark Ricard
Mark Ricca
Massimo Riccaboni
Carlo Riccardi
Niccolò Riccardi
Laura Riccetti
Alessandro Ricci
Claudia Ricci
Fabrizio Ricci
Zaccaria Ricci
Ana Laura Ricci Vitor
Carmela Ricciardelli
Paola Ricciardelli
Enzo Ricciardi
Lucia Ricciardi

Giulia Riccioni
Marco Riccucci
Brian Rice
Charles Rice
Hannah Rice
Ian Rice
Kelly Rice
Laura Rice
Louis Rice
Margaret Rice
Mindy Rice
Nancy Rice
Robert Rice
Simon Rice
Whitney Rice
Alisa Rich
Alison Rich
Jeremy Rich
Josiah Rich
Ryan Rich
Thomas Rich
Anna Richard
Dave Richard
Denis Richard
Isabelle Richard
John Richard
Patrick Richard
Richard Guerrant
Sproat Richard
Tom Richard
Vincent Richard
Stephane Richard-Devantoy
Florence Richard-Forget
Chesley Richards
David Richards
Elizabeth Richards
Jennifer Richards
Joanne Richards
Kyle Richards
Libby Richards
Peter Richards
Philip Richards
Toby Richards
Todd Richards
Rebecca Richards-Kortum
Barbra Richardson
Bryan Richardson
Clive Richardson
Daniel Richardson
Evan Richardson
Ian Richardson
Jason Richardson
Jonathan Richardson
Kelly Richardson
Michael Richardson
Rob Richardson
Robert Richardson
Sarah Richardson
Thomas Richardson
Corinne Richards-Zawacki
Amanda Richdale
Philippe Richebe
Martin Richer
Philip Riches
Matteo G. Richiardi
Jerome Richie
Ilana Richman
Doug Richmond
Jacqueline Richmond
Neil Richtand
Claudia Richter
Daniel Richter
Franziska Richter
Jan Richter
Joel Richter
Juliane Richter
Jurgen Richter
Klaus Richter
Kneginja Richter
Sandra Richter
Sara Richter
Wiltrud Richter
Alex Richter-Boix
Joan Richtsmeier
Jacob Ricker-Gilbert
Volker Rickerts
Christopher Ricketts
Marie-Louise Ricketts

Yessica Rico
Valeria Ricotti
Michele Ricupero
Corinne Riddell
Hugh Riddell
James Riddell
John Ridenour
Cynthia Rider
Elizabeth Rider
Jennifer Rider
Paul Rider
Sarah Ridge
Angela Ridgel
Nicola Ridgers
Jennifer Ridgeway
Anne Ridley
Lisa Ridnour
Ezequiel Ridruejo
Sami Ridwan
Igor Riečanský
Klaus Riede
Tobias Riede
David Riedel
Christine Riedy
Winfried Rief
John Rieffel
Timo Rieg
Bernd Rieger
Diana Rieger
Heiko Rieger
Christina Riehl
Michael Riehle
Gregory Riely
Thomas Riemensperger
Martin Riemer
Chad Rienstra
Michiel Rienstra
Eduardo A. Rienzi
Assunta De Rienzo
Jorge Riera
Andrew Ries
J. A. Riesco
Florian Riese
Thomas Riess
Scott Riester
Jens Rietdorf
Cornelius Rietveld
Guillaume Rieucau
Christian Rifbjerg
Brittany Rife
Jeffrey Riffell
Ian Rifkin
Riaan Rifkin
Francois Rigal
Antonello Rigamonti
Aurora Rigamonti
Maria Manuela Rigano
Chiara Riganti
Stefano Rigattieri
David Rigby
Edward Rigdon
Jessica Rigg
Rebecca Riggins
Blake Riggs
Paolo Righi
Nicoletta Righini
Paola Rigo
Mattia Rigotti
Paolo Rigotti
Thomas Rigotti
Isidore Rigoutsos
Lionel Rigoux
Stephanie C. Rigters
Johanna Riikonen
Robert Gabriel Coumine Riis
Helle Riisgaard
Pappu Rijal
Bob Rijkers
Huub Rijnaarts
Peter Rijnbeek
Monique Rijnkels
Patrícia Rijo
Filipa Rijo-Ferreira
Niels Riksen
Bruce Riley
Edward Riley
Kasandra Riley
Lee Riley

Robert Riley
Rohan Riley
Sean Riley
Thomas Riley
Jonathan Rilinger
Gerald Rimbach
Ulrike Rimmele
Espen Rimstad
Lisa Rimsza
Barbara Rinaldi
Luca Rinaldi
Mauro Rinaldi
Natalia Rinaldi
Simon Rinaldi
Paolo Rinaudo
Luis Rincon
Ole Marius Rindal
Monika Rinder
Thomas Rindflesch
Andrei Rineiski
David Ring
Hans Christian Ring
Dagmar Ringe
Tamar Ringel-Kulka
Jordan Ringenberg
Inge Ringheim
John Rinker
Yasushi Rino
John Rinzel
James Riordan
Anne Rios
Jonathan Rios
Kimberly Rios
Leonardo Rios
Paula Rios
Fernando Riosmena
Rafael Ríos-Tamayo
Blanca Rios-Touma
Diego Rios-Zertuche
Catherine Riou
Alessandro Ripani
John Ripmeester
Cristina Ripoll
Jordi Ripollés
Catarina Rippe
Simon Ripperger
Adrián Riquelme
Inmaculada Riquelme
Matiko Riro
Laurence Ris
Harvey Risch
Patrizia Rise
William Risher
Lavanya Rishishwar
Patricia Risica
Kristin Rising
Evan Risko
Michael Risner
Laetitia Rispel
Theo Rispens
Antti Rissanen
Robert Rissman
Davide Risso
Sinja Rist
G. Ristagno
Jarmo Ritari
Andrew Ritchie
Jan Ritchie
Mona Ritchie
Stephen Ritchie
William Ritchie
Nicolas Riteau
Lauren Riters
Konstantinos Ritis
Kevin Ritsche
Merel Ritskes-Hoitinga
Massimo Rittà
Nuttapol Rittayamai
Chadwick Rittenhouse
Brigitte Ritter
Tim Rittman
Jens Rittscher
Jeanne Rittschof
Eloisa Riva
Michele A. Riva
Emilia Rivadeneira
Casto Rivadulla
Florent Rivals

Peter Rivard
Coralia Rivas
Germán Rivas
Bruno Rivas-Santiago
Ana Rivera
Laura Rivera
Luis Rivera
Maite Rivera Gorrin
David Rivera-Aponte
Rolando Rivera-Pomar
Loren Rivera-Vega
M. Jordana Rivero
Antonio Rivero-Juarez
Caitlin Rivers
Carly Rivers
Charles Rivers
James Rivers
Emily Rivest
Guillaume Riviere
Ilaria Rivolta
Chandrashekar Rivonker
Konstantinos Rizas
Nasser Rizk
Dimitrios Rizos
Giuliano Rizzardini
Francesca Rizzello
Simone Rizzetto
Liara Rizzi
Marco Rizzi
Albert Skip Rizzo
Giuseppe Rizzo
Mariarosaria Rizzo
Mark Rizzo
Milena Rizzo
Victor Rizzo
Wenru Wang Rn
Javier Roa
Sergio Roa
Alexandra Roach
Mack Roach
Michael Roach
Neil Roach
Nadia Roan
Brendan Roark
Thaisa Roat
Ubaidur Rob
Kedir Roba
Bernard Robaire
Kelly Roballo
Joana Robalo
Bernard Robaye
Chiara Robba
Albert Robbat
Cheryl Robbins
Jeffrey Robbins
Nicole Robbins
Paul Robbins
Reuben Robbins
Shawn Robbins
Gwen Robbins Schug
Todd Robeck
Caroline Roben
Eyal Robenshtok
Erik Roberson
Paula Roberson
Shamarial Roberson
Claude Robert
Dominique Robert
Francois Robert
Godfree Robert
Jeanne Robert
Matthieu Robert
Débora Roberti
Josh Roberti
Paula Roberti
Giuseppe Roberto
Blaine Roberts
Charles Roberts
Chris Roberts
Dar Roberts
David Roberts
Donna Roberts
Eric Roberts
Eve Roberts
Georgia Roberts
Irma Roberts
Jeffrey Roberts
John Roberts

Larry Roberts
Lynne Roberts
Marilyn Roberts
Mary Roberts
Matthew Roberts
Michael Roberts
Michelle Roberts
Neil Roberts
Patrick Roberts
R. Micahel Roberts
Rachel Roberts
Reece Roberts
Richard Roberts
Sam Roberts
Susan Roberts
Tawna Roberts
Thomas Roberts
Ashley Robertson
Benjamin Robertson
Christopher Robertson
Colin Robertson
Danielle Robertson
Deborah Robertson
Deirdre A. Robertson
Gavin Robertson
Joshua Robertson
Kevin Robertson
Kis Robertson
Michael Robertson
Sam Robertson
Sherry Robertson
Alice Robie
Charles Robin
Don Robinaugh
Andrew Robins
Joseph Robins
R. Judd Robins
Anne Robinson
Christopher Robinson
Clifford Robinson
Emma Robinson
Jace Robinson
Jason Robinson
Jennifer Robinson
Joann Robinson
Karl Robinson
Kelly Robinson
Leanne Robinson
Lisa Robinson
Matthew Robinson
Meghan Robinson
Michael Robinson
Mikella Robinson
Natalie Robinson
Ralph Robinson
Rob Robinson
Scott Robinson
Sean Robinson
Sonia Robinson
Terry Robinson
Todd Robinson
Nicolas Robinson-Garcia
Kessica Robinson-Papp
R. Aaron Robison
Juan Robledo-Arnuncio
Eva Roblegg
Ana Robles
Francisco Robles
Hugo Robles
Vladimir Robles
Douglas Roblin
Xavier Roblin
Sue Robson
Tracy Robson
Katherine Roby
Mohamed Hussein Roby
Xavier Roca
Corinne Rocca
Maria Rocca
Stefano Rocca
Aldo M. Roccaro
Camilla Rocchi
Francesca Rocchio
Adina Maria Roceanu
Marie Roch
Toralf Roch
A. Rocha
Dominique Rocha

Edson Rocha
Maria José Rocha
Isabelle Rochat
Philipe Rochat
Dominique Roche
Michael Roche
Nicolas Roche
Philippe Roche
Christian Rocheleau
Jonathan Rocheleau
Margarida Rocheta
Patrick Rochette
Michael Rochford
Chelsea Rochman
Bram Rochwerg
Charles Rock
Ronald Rock
David Rocke
Christoph Rocken
Peter Rockers
Bonny Rockette-Wagner
Don Rockey
Joacim Rocklöv
Beth Rockmill
Brigitte Rockstroh
Nathan C. Rockwell
Edward Rockwood
Barry Rockx
André Rodacki
Aylin Rodan
Felicity Roddick
Julian Rode
Wojciech Rode
Thomas Rodebaugh
Francesco Rodeghiero
Markus Rodehutscord
Franz Rödel
Mark-Oliver Rödel
Richard Roden
Richard Rodenburg
Sumayah Rodenburg-Vandenbussche
Stefanie Roder
Marion Röder
Michael Röder
Christoph Roderburg
Damien Rodger
Hamish D. Rodger
James A. Rodger
Matthew Rodger
George Rodgers
Mary Rodgers
Rachel Rodgers
Giovanna Chiara Rodi
Marie Helene Rodier
Siegfried Rödl
Karin Rodland
Marina Rodnina
Fernando Rodrigo
R. Rodrigo
Regina Rodrigo
Karen Rodrigue
Alexandre Rodrigues
André Roger Rodrigues
Bruno Rodrigues
Célia F. Rodrigues
Charlene Rodrigues
Clara Rodrigues
Debora Rodrigues
Fernando Rodrigues
Heber Rodrigues
I. B. Rodrigues
J. L. Rodrigues
Josias Rodrigues
Juliana Rodrigues
Nuno Rodrigues
Sergio Rodrigues
Vasco Rodrigues
Romeu Rodrigues de Souza
Renata Rodrigues Garcia
Alejandro Rodriguez
Alexander Rodriguez
Americo Rodriguez
Clara Rodriguez
Edgardo Rodriguez
Fernando Rodriguez
G. Marcela Rodriguez
Hector Rodriguez

Jan Rodriguez
Lorraine Rodriguez
Nuria Rodriguez
Pablo Rodriguez
Pedro Rodriguez
Ronald M. Rodriguez
Teresa Rodriguez
Tristan Rodriguez
Violeta Rodriguez
Amaia Rodríguez
Ferran Rodríguez
Jose Alberto Rodriguez Castillo
Rayner Rodriguez Diaz
Manuel Rodriguez Gomez
Jesús Rodríguez Huertas
Socorro Maria Rodriguez Pinilla
Mariano Rodriguez Recio
Jose Rodriguez-Alvarez
Lleretny Rodríguez-Álvarez
Fernando Rodriguez-Artalejo
Domingo Savio Rodríguez-Baena
Enrique Rodriguez-Boulan
Sofía Rodríguez-Brenes
Jesus Rodriguez-Calcerrada
Teresa Rodriguez-Calvo
Marisa Rodriguez-Carmona
Adrian Rodriguez-Contreras
Maricela Rodriguez-Cruz
Armando Rodríguez-Durán
Susana Rodríguez-Echeverría
Sara Rodríguez-Enríquez
Naiara Rodríguez-Ezpeleta
Juan Rodriguez-Flores
Francisco Rodriguez-Frias
María Rodríguez-García
Raquel Rodríguez-González
Beatriz Rodriguez-Grande
Nieves Rodríguez-Henche
Bernardo Rodriguez-Iturbe
Ismael Rodriguez-Lara
Heriberto Rodriguez-Martinez
Sarah Rodriguez-Martinez
Ana Rodriguez-Mateos
Alejandro Rodríguez-Molinero
Rosa Rodriguez-Monguio
Alfonso J. Rodriguez-Morales
Alfonso Rodriguez-Moralres
Luis J. Rodríguez-Muñiz
Antonio Rodríguez-Núnez
Manuel Rodríguez-Ortega
Francisco Rodriguez-Panadero
Luis Alberto Rodríguez-Picón
Diego Rodriguez-Puyol
Noe Rodriguez-Rodriguez
Alexandro Rodriguez-Rojas
Adela Rodríguez-Romero
José Domingo Rodríguez-Teijeiro
Juan Rodriguez-Testal
Aída Verónica Rodríguez-Tovar
Laura Rodriguez-Villamizar
Mayela Rodríguez-Violante
Kenny Rodriguez-Wallberg
Dawn Rodriguez-Ward
Raphaël Roduit
Wellington Rody
Brian Roe
R. Roe
Elke Roeb
Claudia Roebers
Anton Roebroek
Anthony J. Roecker
Adrienne Roeder
Karl Roeder
Henry Roediger
Jobst Roehmel
Patrick Roehrdanz
Ute Roehrig
Eric J. Roeland
Bart Roelands
Daniel Roelke
Ferry Roelofs
Ryan Roemmich
Paul Roepe
Troy Roepke
Robert Roer
Luiz Roesch

Annemiek Roescher
Rafael Roesler
Wolfgang Roessler
Hendrik Jan Roest
Herbert Roeyers
George Roff
A. Roffi
Tomasz Rog
Jacek Rogala
Emily Rogalski
Eleanor Rogan
John Rogan
Jane Rogathi
Inez Rogatsky
Elizabeth Rogawski
Andrés Rogberg-Muñoz
Ole Rogeberg
Boris Rogelj
Joeri Rogelj
Lars Rogenmoser
Clair Roger
Sébastien Roger
Amino Rogerio
Angela Rogers
Ann Rogers
Anna Joy Rogers
Buck Rogers
Carolyn Rogers
Catherine Rogers
Chris Rogers
Christopher Rogers
Jeremy Rogers
Kerrylee Rogers
Kristen Rogers
Lynette Rogers
Mary Rogers
Melissa Rogers
Raymond Rogers
Terry Rogers
Toby Rogers
Stephen Rogerson
Hans Roggendorf
Mehrdad Roghani
Florian Roghmann
Mary-Claire Roghmann
Eric Rogier
Gerhard Rogler
Cecilia Rogmark
Ute Rogner
Torbjørn Rognes
Carla Rognoni
Moshe Rogosnitzsky
Artem Rogovskyy
Ana Rogulja-Ortmann
Hyolyun Roh
Seong Woon Roh
Tibor Rohacs
Lisa Rohan
Camelia Rohani
Anand Rohatgi
Christopher Rohde
Gudrun Rohde
Holger Rohde
Paul Rohde
Stefan Rohde
Nerea Roher
Aarti Rohira
Helena Rohlf
Katrin Rohlf
Ingo Rohlfing
Marko Rohlfs
Jos Rohling
Robert Rohling
Sascha Rohn
Nicolas Rohner
Jason Rohr
Julia Rohr
Olivier Rohr
Elene Rohrbach
Ron Rohrbaugh
Benjamin Rohrer
Doug Rohrer
Rod Rohrich
Thomas Rohrlack
Nataliya Rohr-Udilova
Remo Rohs
Damaris Rohsenow
Forest Rohwer

Jens Rohwer
Giulio Sergio Roi
Jordi Roig
Marc Roig
Anna Roik
Marina Roizenblatt
Xavier Rojano i Luque
Laia Rojano-Doñate
Fabio Rojas
I. Rojas
Mauricio Rojas
Rafael Rojas-Herrera
Marcela Rojas-Pierce
Jorge Rojas-Serrano
Pablo Rojo
Theodore Rokkas
Mohammad Rokni
Lisa Rokoff
Casper Rokx
M. Roland
Wibke Roland
Eduardo Roldan
Barry V. Rolett
Leàn Rolfes
Ronda Rolfes
Kristofer Rolfhus
Stefano Rolfo
Martin Rolfs
Ola Rolfson
Jonathan Rolison
Daniel Rolke
Filip Rolland
Vivien Rolland
Teresa Rolle
Luca Rollè
Derrick Rollins
Janet Rollins
Christine Rollinson
Barbara Rolls
Daniel Rolnik
Anne Rolton
Tim Rolvien
Slava Rom
William Rom
Renato Romagnoli
Stefano Romagnoli
Jesus Romalde
Brian Roman
Jesse Roman
Michael Roman
Nicolette V. Roman
Shaun Roman
Rosa Roman Cuesta
Christopher Romanek
Alessandra Romanelli
Razvan Romanescu
Andrea Romani
Roberto Romani
Michael Romann
Corrado Romano
Giovanna Romano
Mario Romano
Marta Romano
Maurizio Romano
Patrick Romano
Marcel Romanos
Victor Romanov
Iza Romanowska
Mateusz Romanowski
Pascal Romans
Andres Roman-Urrestarazu
Dan Romanyk
Luisa Romao
Jan-Erik Romar
Artur Rombenso
Jörg Römbke
Yoann Rombouts
Vincenzo Romei
Jörg Romeis
Orazio Romeo
Russell Romeo
Stefania Romeo
Stefano Romeo
Daniel Romer
Fabio Romerio
Alejandro Romero
Antonio Romero
Daniel Romero

Irene Romero
José Romero
Laura Romero
Miguel Romero
Pedro Romero
Philip Romero
Veronica Romero
Santiago Romero Brufau
Pedro Romero-Aroca
Juanita Romero-Diaz
Adriana Romero-Olivares
Marina Romero-Ramos
Martin Enrique Romero-Sanchez
Jeanne Romero-Severson
Paul Romesser
Christopher Romines
Sarah Rominski
Anna-Sophie Rommel
Annette Rompel
Stefano Romussi
Eliora Z. Ron
Isobel Ronai
Zsuzsanna Rónai
James Ronald
Massimo Roncalli
Vittoria Roncalli
Carlos Roncero
Andrea Ronchi
Enrico Ronchi
Chiara Ronchini
Maurizio Ronci
Paolo Roncon
Mariangela Rondanelli
Matthew Rondina
Hadassa Roness
Charles Roney
Mingqiang Rong
Peijing Rong
Zhihai Rong
Christopher Rongo
Arvid Rongve
Antti Ronkainen
Regin Rønn
Lars Rönnstrand
Gunnar Ronquist
Julian Rood
Garson Roodman
Elizabeth Roof
Jerri Rook
Lorenzo Rook
Mieneke Rook
Matti Rookus
Kieron Rooney
Nicola Rooney
Vinai Roopchansingh
Chris Rooper
Caroline Roos
Dirk Roos
Leonie Roos
Natalia Roos
Wynand Roos
Johnna Roose
Mikeal Roose
Tiina Roose
Christof Röösli
Marilyn Roossinck
Karen Root
Kraig Roozeboom
Bob Roozenbeek
Stefan Ropele
Caroline Roper
James Roper
Louise Roper
Michael Roper
Randall Roper
Stephen Roper
K. Ropka-Molik
Athina Ropodi
André Luiz Roque
F. O. Roque
Fátima Roque
Anne-Marie Roque-Afonso
Antoine Roquilly
Jacki Rorabaugh
Anna Karla Roriz
Maria Vilain Rørvang
Christian Ros

Alberto Rosa
Daniela Rosa
Luiz Rosa
Alfonso Rosa Garcia
Elizabeth Rosado Balmayor
Ji Rosales Leal
Sergio Rosales-Corral
Gustavo Rosania
Mario Rosanova
Steven Rosansky
Karyna Rosario
Michael Rosario
Fernando Rosas
Mauricio Rosas Ballina
Rigoberto Rosas-Luis
Christian Rosas-Salazar
Alberto Rosati
Alexandra Rosati
Ernest Rosato
Hamilton Roschel
Anna Roschke
Federica Roscioni
Sabine Roscoat
Federico Rosconi
Jullia Rosdahl
Bob Rose
Chad Rose
Dorian Rose
Greg Rose
Jason Rose
Jessica Rose
Jonathan Rose
Nathan Rose
Peter Rose
Ray Rose
Robert Rose
Shyanika Rose
Stephen Rose
Steven Rose
Laura Rosella
Marianna Roselli
Mario Roselli
Daniele Rosellini
Stefanie Rosema
Stephanie Rosemann
Mario Rosemblatt
Bradley Rosen
David Rosen
Evan Rosen
Michael Rosen
Charlotte Rosenbach
Kierstin Rosenbach
David Rosenbaum
Michael Rosenbaum
Aaron S. Rosenberg
Avi Rosenberg
Helene Rosenberg
Kenneth Rosenberg
Michael Rosenberg
Molly Rosenberg
Robin Rosenberg
Shoshana Rosenberg
Susan Rosenberg
Kerstin Rosenberger
Peter Rosenberger
William Rosenberger
Miriam Rosenberg-Lee
David Rosenblatt
Noah Rosenblatt
Mosi Rosenboim
Jenny Rosendahl
Jonas Rosendahl
Anatoly Rosenfeld
Jason Rosenfeld
Jordan Rosenfeld
Roni Rosenfeld
Axel Rosenhahn
Jessica Rosenholm
David Rosenkranz
Gerd Rosenkranz
J. Rosenkranz
Tim Rosenow
Robert Rosenson
Alan Rosenstein
Elliot Rosenstein
Ruth Rosenstein
Tatiana Rosenstock
Eyal Rosenstreich

Robert Rosenswig
Benjamin Rosenthal
Eben Rosenthal
Lawrence Rosenthal
Martin Rosenthal
Meagen Rosenthal
Victor Rosenthal
Astrid Rosenthal-von der Pütten
Alan Rosenwasser
Stav Rosenzweig
Steven Rosenzweig
Connie Roser-Renouf
Florencia Rosetti
Henry Rosevear
Roger Ros-Freixedes
Abdolreza Roshani
Baback Roshanravan
James Roshetko
Peter F. W. M. Rosier
Francesca Rosignolo
Diane Rosin
Paul Rosin
Zuzanna Rosin
Tomas Roslin
Guy Rosman
Yossi Rosman
Bernard Rosner
Mara Rosner
A. Ross
Adam Ross
Amanda Ross
Amy Ross
Bernhard Ross
Christina Ross
Cody Ross
David Ross
Donald Ross
Jonathan Ross
Kenneth Ross
Lori Ross
Rebecca Ross
Robert Ross
Ryan Ross
Shannon Ross
Sharon Ross
Stephen Ross
Ted Ross
Will Ross
Jan Rossaint
Mateus Rossato
Davi Rossatto
Dennis Ross-Degnan
Josep Rosselló
Charles Rosser
Andrea Rossetti
Giulia Rossetti
Luca Rossetti
Maurizio Rossetto
Vanessa Rossetto Marcelino
Anna Rita Rossi
Antonio Rossi
David Rossi
Denise Rossi
Ethan Rossi
F. Rossi
Federico Rossi
Giulio Rossi
Iara G. Rossi
Leonardo Rossi
Luca Rossi
Maria Chiara Rossi
Marvin A. Rossi
Maximo Rossi
Mirko Rossi
Paolo Rossi
Robert Rossi
Sandrine Rossi
Sergio Rossi
Simone Rossi
Sonja Rossi
Valentina Rossi
F. Rossi Caruso
Giampiero Rossi-Fedele
Mandy Rossignol
Maria Rossing
Marcos Rossi-Santos
Roland Rössler
Kent Rossman

Sam Rossman
Moritz Rossner
Daniela Eugenia Rosso
Paolo Rosso
Raphael Rosso
Rebecca Rossom
Theresa Rossouw
Edina Rosta
Carlo Rostagno
Lionel Rostaing
Muhamad Ashraf Rostam
Amir Rostami
Javad Rostami
Vittorio Rosti
Siri Rostoft
Thomas Roston
Magdalena Rost-Roszkowska
Jason Roszik
Fernando Rotellar
Nirit Rotem
Susan Rotenberg
Amber Roth
Christian Roth
Daniel Roth
Edward Roth
Elliot Roth
Michael Roth
Tania Roth
Terri Roth
Zvi Roth
Anjali Röth
Paul Rothberg
Hansjörg Rothe
Richard Rothenberg
Sarah Rothenberg
Martina Rothenbühler
Gernot Rother
Magdalena Rother
Till Rothig
Adam Rothman
Jason Rothman
Michael Rothman
Ling Rothrock
Michael Rothrock
Bruce Rothschild
David Rothschild
David Rothstein
Rodney Rothstein
Edward Rothwell
Anna Rotkirch
Pichayanoot Rotkrua
Josep Rotllant
Oren Rotman
Yaron Rotman
Daniel Rotroff
David Rotsch
Hannelore Rott
Leahora Rotteau
Robbert Rottier
Eric Rottinger
Ben Rottman
Hatem Rouached
Nathalie Rouas-Freiss
Tracey A. Rouault
Pierre Roubertoux
David Roubik
Aymeric Rouchaud
Eric Rouchka
Carlos Rouco
Bram Roudijk
François Rouet
Lieze Rouffaer
Catherine Rougeot
Guillermo Rougier
Anna Roujeinikova
Tarik Roukny
Dimitrios Roukos
Anne-Lyse Roulin Ducrest
Carli Roulston
Nadia Roumeliotis
Lubka Roumenina
Sharon Rounds
Alex Rouquette
Cedric Roure
Liam Rourke
Michelle Rourke
Matthew Rouse
Robert Rouse

Kathrin Rousk
David Rousseau
Eric Rousseau
Guillaume Rousseau
Marie-Claude Rousseau
Marthe Rousseau
Ronald Rousseau
Erwan Roussel
Morgan Roussel
Jonathan Roussey
Panos Roussos
Gyana Rout
Prasant Kumar Rout
Sarit Rout
Jean-Marc Routaboul
Drona Routh
Jean-Pierre Routy
Céline Rouveirol
Berndt Rouven
Christian Roux
Christophe Roux
Perrine Roux
Saartjie Roux
Simon Roux
Heloise Rouze
Christine Rouzioux
Michail Rovatsos
Christian Röver
Patrizia Rovere-Querini
Francesco Rovero
John Rovers
P. Rovira
Joel Rovnak
David Row
Ashley Rowden
Brian Rowe
Gene Rowe
Gilbert Rowe
Glenn Rowe
Steven Rowe
Tawandra Rowell-Cunsolo
Pedram Rowhani
Anisa Rowhani-Farid
Andrew Rowland
Gill Rowland
Raymond Rowland
James Rowlett
Andrew Rowley
Christopher Rowley
James Rowley
Jodi Rowley
Sonia Rowley
Emily Rowlinson
Bethany Rowson
Monica Roxo-Rosa
Abhrajeet Roy
Allison Roy
Ambuj Roy
Arunava Roy
Bidyut Roy
Chad Roy
Charlotte Roy
Denis Roy
Denis-Claude Roy
Eileen Roy
François Roy
Helen Roy
Jean-Sebastien Roy
Laurence Roy
Peter Roy
Pratik Roy
Raja Roy
Rajendra Roy
Sabita Roy
Sayon Roy
Shyamal Roy
Snehashis Roy
Somanth Roy
Somenath Roy
Somendu Roy
Sudipto Roy
Vikas Roy
Henry Royal
Walter Royal III
Astrid Roy-Engel
Aurélien Royer
Danielle Royer
Todd Royer

Luiz Royes
Phaedra Royle
Aritz Royo-Esnal
Sanya Roysommuti
Mercedes Royuela
Luca Roz
Ignacio Rozada
Dmitri Rozanov
Malgorzata Rózanowska
Elizabeth Rozansk
Scott Rozelle
Romke Rozema
Patrick Rozenberg
Danaë Rozendaal
Alexander Rozental
Ales Rozman
Vlatka Rozman
Virginie Rozot
Laurentiu Rozylowicz
Kais Rtibi
Shaoguo Ru
Megan A. Rua
Bowen Ruan
Hai-Bin Ruan
Jue Ruan
Junhu Ruan
Qiang Ruan
Xiangcai Ruan
Zhongyuan Ruan
Alberto Ruano
Edgar Ruano-Fajardo
Jorge Ruas
Martial Ruat
Alevtina Ruban
Matt Rubashkin
Speranza Rubattu
Dennis Rubbenstroth
Paola Rubbioni
Leonid Rubchinsky
Daniel Rubenson
Daniel Rubenstein
Jeff Rubenstein
Jesús Ruberte
Nicolas Rubido
David Rubilar-Rogers
Benjamin Rubin
Katie M. Rubin
Kenneth Rubin
Lewis Rubin
Robert Rubin
Shimshon Rubin
Clara Rubincam
Dvora Rubinger
Alessandro Rubini
Michele Rubini
Orly Rubinsten
Jaime Rubio
Luis Rubio
Marie-Thérèse Rubio
Vicente Rubio
Rafael Rubio de Casas
Juan Rubiolo
Patrizia Rubiolo
Pp Rubis
Diego Rubolini
Nadia Rucci
Klaus Ruckdeschel
Jason Rucker
Julia Rucklidge
Martin Rudasingwa
Anthony Rudd
Jason Rudd
Kristina Rudd
Paula Rudenick
Assaf Rudich
Adam Rudner
Mary Rudner
Abby Rudolph
Heike Rudolph
A. K. M. Ashiqul Haqze Rudra
Shiva Rudraraju
Stephanie Rudwick
Havard Rue
Jon Rue
Meghan Ruebel
Karen Ruebens
Felix Rueckert
María Del Mar Rueda

Marta Rueda
Manuel Ruedi
Sandra Rueger
Joëlle Rüegg
Antonio Ruellas
Aaron Ruesch
Jennifer Ruesink
Dietrich Ruess
Miriam Ruesseler
Maja Ruetten
Kurt Ruetzler
Viktoria Ruf
Katharina Rufener
Enrico Ruffini
Francesco Ruffino
Giancarlo Ruffo
Davide Ruffoni
Paschal Ruggajo
Kai Ruggeri
Paolo Ruggerone
Carmelinda Ruggiero
Giuseppina Ruggiero
Laurie Ruggiero
Jorun Rugkåsa
Anja Ruhdorfer
Zachary Ruhe
Philipp Ruhnau
Isabelle Ruhnke
Stephan Ruhrmann
Hallgeir Rui
Jian Rui
Ynte Ruigrok
Jan Ruijter
Marit F. L. Ruitenberg
Federico Ruiz
Gustavo Rodrigo Sanches Ruiz
Isaac Ruiz
Jeanette Ruiz
Joaquim Ruiz
Jorge Ruiz
Juan Ruiz
Marilyn Ruiz
Marta Ruiz
Cristina Ruiz-Castellano
Mari Carmen Ruiz-Domínguez
Jose Francisco Ruiz-Fons
Mariano Ruiz-Gayo
Paulo Ruiz-Grosso
Juan Manuel Ruiz-Lozano
Kepa Ruiz-Mirazo
Edward Ruiz-Narvaez
Francisco Ruiz-Ojeda
Magdalena Ruiz-Rodriguez
Julian Ruiz-Saenz
Iñaki Ruiz-Trillo
Victor Ruiz-Velasco
Florian Rüker
Nick Ruktanonchai
Godfrey Rukundo
Stephen Rulisa
Martin Rumbo
Darren Rumbold
Tillmann Rumenapf
Franklin Rumjanek
Andrea Rummel
Daniel Runcie
Andrew Rundle
Simon Rundle
Bo Runeson
Kurt Runge
Troy Runge
Elin Runnqvist
Ann Runstrom
Raymond Runyan
Justin Runyon
Michelina Ruocco
Peter Ruoff
Aino Ruohola
Kalle Ruokolainen
Margherita Ruoppolo
John Rupe
Stefan Rupf
Audrey Ruple
Maja Rupnik
Christoph Rupprecht
Colin Ruprecht
Horea Rus
Violeta Rus

Mar Rus-Calafell
Doug Rusch
Laura Rusche
Massimiliano Ruscica
Piero Ruscitti
Roberto Rusconi
Stefano Rusconi
Cristian Ruse
Barret Rush
Kathy Rush
Dinela Rushani
Jacqueline Rushby
Blake Rushing
Jonathan Rushton
Stuart Rushworth
Tatjana Ruskovska
Garry Russ
Katheryn Russ
Lina Russ
Kory Russel
Aaron Russell
Armistead Russell
Clark Russell
Daniel Russell
Douglas Russell
F. Leland Russell
George Russell
James Russell
Jeffrey Russell
John Russell
Keith Russell
Kelly Russell
Mark Russell
Michael Russell
Neal J. Russell
Nerissa Russell
Richard Russell
Roslyn Russell
Scott Russell
Steven Russell
Travis Russell
Maurizio Russello
Jeremy Russell-Smith
Marion Russier
Alessandra Russo
Annapina Russo
Antonio Russo
Claudia Russo
Daniela Russo
Francesco Russo
Frank Russo
Giandomenico Russo
Giulia Russo
Luca Russo
María Julieta Russo
Tommaso Russo
Vincenzo Russo
Carmen Russoniello
Michael Russwurm
Tiana Rust
Navin Rustagi
Arild Rustan
Elena Rustchenko-Bulgac
Aldo Rustichini
Alina Rusu
Cristian Rusu
Daniel Rusyniak
Elizeus Rutebemberwa
Paul Rutecki
Markus Rütgen
Ruth Ruth Ndjaboue
Ed Rutherford
Julienne Rutherford
Mark Rutherford
Sarah Rutherford
Eric Ruthruff
Justin Ruths
Vera Ruthsatz
Jonas Rutishauser
Tom Rutkowski
William Rutledge
Frank Rutsch
Sereina Rutschmann
Elisabet Rutstrom
Michael J. Rutter
Michael Rutzke
Olli Ruuskanen
Timo Ruusuvirta

Pekka Ruusuvuori
Jean-Marie Ruysschaert
Erik Ruzek
Daniel Růžek
Manuela Ruzzoli
Susan Rvachew
Oxana Ryabaya
Natalya Ryabogina
Vyacheslav Ryabov
Annette C. Ryan
Bridget Ryan
Catherine Ryan
Cristin Ryan
David Ryan
Elizabeth Ryan
John Ryan
Joseph Ryan
Julie Ryan
Kathleen Ryan
Lindsay Ryan
Martin Ryan
Michael Ryan
Peter Ryan
Timothy Ryan
Tracii Ryan
William Ryan
Lev Ryashko
Nicholas Ryba
Michael Rybak
Edward Rybicki
Diego Rybski
Ivan Rychlik
Michael Rychlik
Malgorzata Rychlowska
Jens Rydell
Mikael Rydén
Alex Ryder
Sheila Ryder
Kerry-Anne Rye
Evan Ryer
Jamie Rylance
Bartosz Rylski
Brian Rymond
Agnieszka Rynda-Apple
Angela Rynne Vidal
Chongsuk Ryou
Wojciech Rypniewski
Marc Ryser
Peter Ryser
Ales Ryska
Rachel Ryskin
Maren Rytter
James Rytuba
Euijung Ryu
Han Suk Ryu
Jae Jun Ryu
Jong Hoon Ryu
Kwon-Yul Ryu
So Young Ryu
Soojin Ryu
William Ryu
Inui Ryutei
Dmitry Ryvkin
Umesha S.
Shauhrat S. Chopra
Mariana S. De Lorenzo
Alan S. Hazell
Jeffrey S. Morris
Michel Sá
Matthew E. Saab
Gloria Saab-Rincón
Elie Saade
Michael Saag
Hannes Saal
Emily Saarinen
Mart Saarma
Urmas Saarma
Belen Saavedra
Emma Saavedra
Juan Saavedra
Maria Jose Saavedra
Miguel Saavedra
Nabil Saba
Naser Sabaghnia
Maite Sabalza
Richard Saban
Sead Sabanadzovic
Marion Sabart

Debora Sabatelli
Mario Sabatelli
Alfonso L. Sabater
Gerardo Sabater-Grande
Coleen Sabatini
Denise Sabatino
Maura Sabatos-DeVito
Venkatareddy Sabbasani
Maria Sabbatini
Yogesh D. Sabde
Abrar Sabeh
Bastian Sabel
Meead Saberi
Mohammadmehdi Saberioon
Caroline Sabin
Lora Sabin
Caetano Sabino
Joao Sabino
Raquel Sabino
Suraj Sable
Gaurav Sablok
Amit Sabne
Roy Sabo
Ivan Sabol
Zakee Sabree
Kaeser Sabrin
Nirmeen Sabry
Myami Sabu
Juan Sabuco
Sarven Sabunciyan
Ali Mohammad Sabzghabaee
Ahmet Sacan
Sergio Saccà
Isabel Sacco
Melanie Sacco
Ricardo Sacco
Gabriele Saccone
Riccardo Sacconi
Rosa Sacedó
Pawel Sachadyn
Shilpa Sachdeva
Lucia Maria Sacheli
George Sachs
Laurent Sachs
Michael Sachs
Patrick Sachs
Rainer Sachs
Ulrich Sachs
David Sack
Kenneth Sack
Michael Sack
Michael Sacks
Ned Sacktor
Patricia M. Saco
Ken-Ei Sada
Amela Sadagic
Sridha Sadasivam
Behnam Sadeghi
Ebrahim Sadeghi-Demneh
Javad Sadeghinejad
Amir Sadeghpour
Samy Sadek
Vincent Saderme
Siegal Sadetzki
Sushabhan Sadhukhan
Gökhan Sadi
Alexa Sadier
Christian Sadik
Mauricio Sadinle
Kirsten Sadler Edepli
Laura Sadofsky
Laura Sadori
Ewa Sadowy
Alireza Sadr
Arif Mohaimin Sadri
Sohrab Saeb
Kourosh Saeb Parsy
Eva Saedder
Fahad Saeed
Maythem Saeed
Muhammad Saeed
Taheri Saeed
Alireza Saeidi
Hiroshi Saeki
Jun Saeki
Susanne Gjørup Sækmose
Xavier Saelens
Christoph Saely

Fabian Saenz
Carlos Sáenz
Cuauhtemoc Saenz-Romero
Phoonthawee Saetear
Stig Arve Sæther
Bjørn Sætrevik
A. Saez
Fabrice Saez
Juan Saez
Ahmad R. Safa
Mohammad Reza Safaei
Adriana Vaz Safatle-Ribeiro
Darya Safavian
Stephen Safe
Joshua Safer
Saveez Saffarian
David Saffen
Jenny Saffran
Kamran Safi
Saaid Safieddine
Barbara Safiejko-Mroczka
Robert Safirstein
Steven Safren
Ilya Safro
Ahmed Safwat
Sandrine Sagan
Yoshimasa Sagane
K. Sagar
Malvika Sagar
Manish Sagar
Brad Sagarin
Katayo Sagata
Andrew Sage
Colin Sage
Valeria Sagheddu
Mohammad Ali Saghiri
Mehrnoosh Saghizadeh
Dov Sagi
Moshe Sagi
Ramesh Sagili
Jesse Saginor
Evangelista Sagnelli
Kwamena Sagoe
Mark Sagoff
Gurdeep Sagoo
Ardan Saguner
Evans Sagwa
Pratha Sah
Rajan Sah
Robert Sah
Biswarup Saha
Goutum Saha
Krishanu Saha
Nirmalendu Saha
Sibu Saha
Ajay Sahajpal
Tony R. Sahama
Kate Sahan
Goutam Sahana
Dushyant Sahani
Vikas Anand Saharan
Pipsa Saharinen
Anagh Sahasrabuddhe
Bikash Sahay
Gaurav Sahay
Pooyan Sahbaee
Baljinder Sahdra
Mahbod Sahebi
Cagdas Sahin
Hacer Sahin
Kazim Sahin
Endalkachew Sahle-Demessie
Gregory L. Sahlem
Chaima Sahli
Michelle Sahli
N. Sahnane
Sanjeev Sahni
Daisy Sahoo
Harekrushna Sahoo
Lingaraj Sahoo
Malaya Sahoo
Manoranjan Sahoo
Sanjaya Sahoo
Satyaprakash Sahoo
Mariam Sahrawy
Itishri Sahu
Ranabir Sahu
Saura Sahu

Ronald Sahyouni
Francesco Saia
Joanne Said
Reza Saidi
Nahel Saied
Milton Saier
Samia Saif
Romesh Saigal
Hiroto Saigo
Eri Saijo
Masayuki Saijo
Yasuaki Saijo
Weerachai Saijuntha
Shizuya Saika
Vesa Saikko
G. Saikumar
Pothana Saikumar
Kerstin Sailer
Satheesh Sainathan
Neeraj Saini
Amanda Sainsbury
Frank Sainsbury
Dave Saint-Amour
Magali Saint-Geniez
Olivier Saint-Lary
Pedro Saint-Maurice
Akinori Sairaku
Yoshifumi Saisho
Akira Saito
Atsuko Saito
Atsushi Saito
Kanako Saito
Mariko Saito
Reiko Saito
Shigeyiki Saito
Wataru Saito
Yoshihiko Saito
Kazuyoshi Saitoh
Tatsuya Saitoh
Inigo Saiz-Fernandez
Alfonso Saiz-Lopez
Sima Sajedinejad
Hideo Saji
Muhammad Sajid Nadeem
Umadevi Sajjan
Tolulope Sajobi
Mamta Sajwan-Khatri
Yosuke Saka
Sava Sakadzic
Masamichi Sakagami
Hirokazu Sakaguchi
Kazuhiko Sakaguchi
Koichi Sakaguchi
Yusuke Sakaguchi
Daisuke Sakai
F. Sakai
Kyoko Sakai
Shinji Sakai
Takayoshi Sakai
Tetsuro Sakai
Hitoshi Sakakibara
Koichi Sakakura
John Sakaluk
Masaki Sakamoto
Masaya Sakamoto
Shuichi Sakamoto
Taiichi Sakamoto
Takuma Sakamoto
Tomoya Sakamoto
Cristina Sakarikou
Jun Sakata
Shogo Sakata
Khashayar Sakhaee
Sachin Sakhare
Amrit Sakhi
Selvaraj Sakhthivel
Yuya Sakimoto
Kazuo Sakka
Samir Sakka
Vangelis Sakkalis
Lazaros Sakkas
Nilva K. Sakomura
George Sakoulas
Agata Sakowicz
Yasser Sakr
Joseph Sakran
Seema Saksena
Kunihiro Sakuma

Tetsushi Sakuma
Anavaj Sakuntabhai
Takaya Sakura
Tsutomu Sakurada
Akihiko Sakurai
Ken Sakushima
Angelo Sala
Claudia Sala
Mariaelvina Sala
Núria Sala
Concepcion Salaberria
Amanda Salacinski
Estela Salagre
Akram Salah
Cherif Salama
Guy Salama
Mohamed Salama
Nabeil Salama
Suhyb Salama
Kabiru Salami
M. Salami
Simpa Salami
Attila Salamon
D. Salamone
Asaf Salamov
Laura Salanova
Laure Salanova
Ali-Akbar Salari
Antonio Salas
Eva Salas
David Salas De Leon
Massimiliano Salati
Ma Isabel Salazar
Mariano Salazar
Miguel Salazar
Valerie Salazar
Gilles Salbert
Rosalba Salcedo
D. D. Salcido
Silvia Salcuni
Clara Saldamando-Benjumea
Omar Saldarriaga
Paulo Saldiva
Sandra Saldivia
Raquel Sá-Leão
Paul Saleeb
Ali Saleem
Haneefa Tasleem Saleem
Imram Saleem
Anas Saleh
Ahmad Salehi
Maryam Salehi
Ali Salehzadeh-Yazdi
Reza Salek
Giuseppe Salemi
Graciela Salerno
Patricia Salerno
Rosangela Salerno-Goncalves
G. Salesi
Antonio Salgado
Roberto Salgado
Teresa Salgado
Ana Salge
Francisco Salguero
Anthony Sali
Elie Saliba
Faouzi Saliba
Miguel Salido
Samina Salim
Elena Salina
Armando Salinas
Emilio Salinas
Pablo Salinas
Roberto Salinas
Abraham Salinas-Miranda
Sarah Salisbury
Daniel Salkeld
Abd El-Azeem Sallam
Ahmed Sallam
Aurélien Sallé
Mohd Zaki Salleh
Arghavan Salles
Rosana Salles-Costa
Isabelle Salles-Crawley
Brenda Salley
Mikael Sallinen
Hannes Sallmon
Stephan Salloway

Fabio Sallustio
Fala Salma
Tamer Salman
Reza Salman Yazdi
Mehrdad Salmasi
Nico Salmaso
Ladislao Salmeron
Paulina Salminen
Dominique Salmon
J. Meghan Salmon
Maëlle Salmon
Patrick Salmon
Jordi Salmona
Lyubov Salnikova
Emili Saló
Matias Salomao
Maria Salomidi
Dor Salomon
Kristen Salomon
Tim Salomons
Deborah Salon
Antti Saloniemi
Emma Salt
Kate Salters
Stephen Salton
Jaclyn A. Saltzman
Wendy Saltzman
Ashok Saluja
Andres Salumets
B. Salvador
Jorge A. R. Salvador
Raymond Salvador
Anna Salvati
Ariel Salvatierra
Gerardo Salvato
Francesco Salvatore
Mary Salvatore
Silvia Salvatore
Renato Salvatteci
Elisa Salvetti
Massimo Salvetti
Mario Salvi
Rosanna Salvia
Alessandra Salvioli
Francisco Salzano
Giulia Salzano
Thomas Salzberger
Walter Salzburger
Sonja Sälzer
Oren Salzman
I-Ching Sam
Susan Sam
Zilioli Sam
M. Constantine Samaan
Zainab Samaan
Nasser Samadi
Afshin Samani
Victoria Samanidou
Edgar Samaniego
Horacio Samaniego
Reyna Sámano
Ashis Samanta
Luna Samanta
Mrinal Samanta
Palash Samanta
Saheli Samanta
Simbarashe Samapundo
Ibrahim Samar
Theodoros Samaras
Tom Samaras
Amali Samarasinghe
Iryna Samarska
Eric Samarut
Nisha Sambamurthy
Antonia Sambola
Vittorio Sambri
Benjamin Samelson-Jones
Arun Samidurai
Thangavel Samikkannu
Martina Samiotaki
Reham Samir
Konstantinos Samitas
R. Douglas Sammons
Tarik Sammour
Ivan Sammut
Mihail Samnaliev
Andriy Samokhvalov
Valerie Samouillan

Alexandre Samouilov
Cesar Sampaio
B. Sampaio-Maia
John Sampalis
Bhaven Sampat
Venkatesh Sampath
Sruthi Sampathkumar
Jelmer Samplonius
David Sampson
John Sampson
Matthew Sampson
Timothy Sampson
Antony Samrot
Timofey Samsonov
Rebekah Samsonraj
Anjas Samsudin
Jayesh Samtani
Buka Samten
Chandramathi Samudi Raju
Ismael Samudio
Chrishan Samuel
Jastin Samuel
Michael Samuel
Preethy Samuel
Ravi Samuel
Thelma Samuels
Ole Samuelsen
John Samuelson
Anne-Maj Samuelsson
John Samuelsson
Abdallah Samy
Ana San Felix
Alejandro San Juan
Barindra Sana
Thibault Sana
Shib Sankar Sanab
Federico Sanabria
Hiromi Sanada
Kenji Sanada
Kiyoshi Sanada
Shoji Sanada
Faisal Sanai
Madhusudhan Sanaka
Tiziana Sanavia
Paul Sanborn
Flavio Sanches Politi
Angel Matías Sanchez
Cecilia Sanchez
Diana Sanchez
Elda Sanchez
Esteban Sanchez
Gloria Sanchez
Helmuth Sanchez
Luis Sanchez
M. Sanchez
Mauricio A. Sanchez
Otto A. Sanchez
S. Sanchez
Susan Sanchez
Yolanda Sanchez
Angel Sánchez
Carlos Sánchez
Clarisa Sánchez
Daniel Sánchez
Ignacio Sánchez
Marina Sánchez
Maria Teresa Sanchez Aparicio
Rut Sanchez de Dios
Gonzalo Sanchez Duffhues
Antonio Sanchez-Amat
Carmen Sanchez-Avila
Maria Teresa Sanchez-Ballesta
Francisco Sanchez-Bayo
Tilman Sanchez-Elsner
Sergio Sanchez-Esquivel
Alejandro Sanchez-Flores
Antonio Jesus Sánchez-Fuentes
Sergio Sanchez-Garcia
Amaya Sánchez-Gómez
Carmen Sanchez-Gonzalez
Jorge Sanchez-Guerrero
Fermin Sanchez-Guijo
Ernesto Sanchez-Herrero
Maximiliano Sanchez-Lamas
Luis Sánchez-Medina
Adrián Sánchez-Montalvá
Manuel SáNchez-Moreno
Santiago Sánchez-Pagés

L. Nelson Sanchez-Pinto
Martha Sanchez-Rodriguez
Javier Sánchez-Rosas
José Sánchez-Tomero
Andrea Sanchez-Vallet
Marcelo Sánchez-Villagra
Jose Sanchez-Vizcaino
Fabian Sanchis
Juan Sanchis-Gimeno
Joaquin Sanchis-Moysi
Pau Sancho-Bru
Mary J. Sandage
David Sandberg
N. Sandeep
S. Sandeep
Carl Sandén
Guy Sander
J. Sander
Lasse Sander
Myriam Sander
P. Martin Sander
G. R. H. Sandercock
Colin Sanders
David Sanders
Eduard Sanders
Elizabeth Sanders
James Sanders
Jennifer Sanders
Lauren Sanders
Robert Sanders
Scott Sanders
Stacey Sanders
Yan Sanders
Charlotte Sandersen
Eric Sanderson
Hans Sanderson
J. Thomas Sanderson
Ralph Sanderson
Helady Sanders-Pinheiro
Dennis Sandgathe
Roger Sandhoff
Jaspal Sandhu
Paul Sandifer
Pilar Sandín-España
Clara Sandino
Tim Sandle
Dale Sandler
Peter Sandner
Leonidas Sandoval
Luis Sandoval
Milagros Sandoval
Pilar Sandoval
Tatiana Sandoval Guzman
Edson Sandoval-Castellanos
Ionel Sandovici
Olivier Sandra
Leonardo Sandrini-Neto
Inga Sandrock
Anna Sandrucci
Harold H. Sandstea
Gillian Sandstrom
Alessandro Sanduzzi
Daniel Sandweiss
John Sandy
Aniruddha Sane
Jussy Sane
Monica Sañé Schepisi
Yves-Henri Sanejouand
Brooke Sanford
Thomas Sanford
Huiyan Sang
Ali Sangawi
Hoshiar Sangawi
Florencia Sangermano
Morgan Sangeux
Anita Sanghani-Kerai
Dharambir Sanghera
Per Sangild
Sophia Sangiorgio
Kazunori Sango
George Sangster
Chinapa Sangsuwon
Anawin Sanguankeo
Karen Sanguinet
Elena Sanguinetti
Maurizio Sanguinetti
N. Sangwan
Virender Sangwan

Mario Sanhueza
Marc Sani
Shabnam Sani
Kazi Sanin
Shomyseh Sanjabi
Neville Sanjana
Mohammad Ali Sanjari
Kadek Sanjaya
Luis San-Jose
Daniel Sanjuan
Adam Sanjurjo
Renu Sankar
Saikolappan Sankaralingam
Vijay Sankaran
Sumathi Sankaran-Walters
Sriram Sankararaman
Vishwanath
Sankarasubramanian
Eric Sankey
Ceyda Sanli
Raimon Sanmarti
Marcelo Sanmartin-Fernandez
Serena Sanna
Simone Sanna-Cherchi
Michel Sanner
Philip Sannes
Anna Sannino
Larissa Sano
Motoaki Sano
Shigetoshi Sano
Teruo Sano
Margarita Sanromán-Junquera
Giovanni Sansavini
Fiona Sansom
Stephanie Sansom
Marcos Sanso-Navarro
Clementina Sansone
Francesco Sansone
Mario Sansone
Charles Sansur
Rita Santacroce
Jose Santacruz
Carlo Santaguida
Alfredo Santalla
Niovi Santama
Ivan Santamaria-Holek
Luis Santana
Maria Santana
Maria Santana-Gallego
Adam Santanasto
Elisa Santandrea
Philip Santangelo
Sabato Santaniello
Mauricio Sant'Anna
Anirban Santara
Enrica Santarcangelo
Michael Santare
Sara Santarelli
Libero Santarpia
Anthony Santella
Regina Santella
Marco Santello
Eduardo Santero
Arvind Santhanakrishnan
Lakshmi Santhanam
Nayantara Santhi
Daniele Santi
Helton Santiago
Jose Santiago
Tania Santiago
Begoña Santiago García
Efraín Santiago-Rodríguez
Sabine Santibanez
Pilar Santidrián Tomillo
Mark Santillan
Ines Santin
Carlo Santini
Ferruccio Santini
Inge M. G. A. Santman-Berends
Debora Santo
Daniele Santoni
Giorgio Santoni
Matteo Santoni
Manuel Santonja
Domenico Santoro
Maria Santoro
Mario Santoro
Nanette Santoro

Rosaria Santoro
Ana Santos
Andres Santos
Carlos Santos
Catarina P. Santos
Celio Santos
Cláudia Santos
Diana Santos
Ernesto Santos
Filipe Santos
Guido Santos
Javier Santos
Joana Santos
Kassius Klay Santos
Leandro Santos
Leonardo Santos
Márcio José Dos Santos
Patricia Santos
Robson Santos
Sílvio Santos
Sofia Santos
Tony Santos
Lara Lívia Santos da Silva
Fernanda Santos Farnese
Alejandro Santos Lozano
Luis Santos Pinto
Ailiana Santosa
Pablo Santos-Iglesias
Alejandro Santos-Lozano
Heri Santoso
Joseph Santos-Sacchi
Gustavo Santoyo-Pizano
Priyabrata Santra
Annalisa Santucci
Sabine Santucci-Darmanin
Lia Santulli
Pietro Santulli
Pierachille Santus
Alessandro Santuz
Lorraine Santy
Mrinmoy Sanyal
Sankar Sanyal
Suparna Sanyal
Guillermo Sanz
Jose Antonio Sanz
Pedro Sanz
Joaquin Sanz Remon
Ana Sanz-Aguilar
Edgardo Sanzana
Simona Marianna Sanzani
Victoria Sanz-Fernandez
Elena C. Sanz-Miralles
Júlio Sá-Oliveira
Murat Saparbaev
Elizabeth Sapey
Przemyslaw (Mike) Sapieha
Piotr Sapiezynski
Anna Sapino
Nir Sapir
Gonzalo Sapisochin
M. Saponari
Thomas Sappington
Muhammad Saqib
Mohammed Saqr
M. Saqui-Salces
Fabio Saracco
Annalisa Saracino
Rebecca Saracino
Renu Saradadevi
Santosh Saraf
Victoria Sarafian
Bruno Saragiotto
Lucilia Saraiva
Margarida Saraiva
Petar Sarajcev
Naowarat Saralamba
(Tanomsing)
Jari Saramaki
Debalin Sarangi
Babak Sarani
Vanessa Sarathy
Athanasios Saratzis
Gurusankar Saravanabhavan
Muthupandian Saravanan
Rathi Saravanan
Shanmugam Saravanan
Jordy Saravia
Kavitha Saravu

Paul Sarbescu
Pinaki Sarder
Luca Sardo
Pedro Sardo
Celestino Sardu
Mona Sarfaty
David Sargan
Carolyn Sargent
L. Sargento
Massimo Sargiacomo
Selahattin Saribay
Hakan Sarikaya
Mehmet Sarikaya
Neera Sarin
Shiv Sarin
Abhinav Sarje
Ananda Sarkar
Banwarilal Sarkar
Debi Sarkar
Dhruba Jyoti Sarkar
Joy Sarkar
Kamal Sarkar
Partha Sarkar
Rupa Sarkar
Sagartirtha Sarkar
Sobhan Sarkar
Sohinee Sarkar
Somwrita Sarkar
Sumit Sarkar
Abdur Sarker
Abeed Sarker
Rim Sabrina Jahan Sarker
Shah-Jalal Sarker
Tiina Särkinen
Rafael Sarkis-Onofre
Birinchi Sarma
Kandarpa Sarma
Moinak Sarma
Nayan Sarma
Hakan Sarman
Sanjeev Sarmukaddam
Tadeusz Sarna
Eli Sarnat
Stefania Sarno
Martin Sarnovsky
Tomasz Sarnowski
Johannes Sarnthein
Pablo Sarobe
Viji Sarojini
Antonio Sarolic
Happiness Saronga
Mihail Sarov
Radka Sarova
Michael Sarr
Amrou Sarraj
Pooria Sarrami
Jean Philippe Sarrette
Alejandro Sarrion-Perdigones
Ehab Sarsour
Prashanat Sarswat
Marcello Sartarelli
Marina Sartini
Cristina Sartor
Maureen Sartor
Vittorio Sartorelli
Erik Sartori
Luisa Sartori
Benn Sartorius
Jhones Sarturi
G. Sarwar
John Sarwark
Mahmood Sasa
Kazutoshi Sasahara
Keisuke Sasaki
Ken-Ichiro Sasaki
Matthew Sasaki
Tamaki Sasaki
Tatsuya Sasaki
Tsuyoshi Sasaki
Kazuhiro Sase
Maria Sasiain
M. Sasikala
Smitha Sasindran
Pavel Saska
Agus P. Sasmito
Giedrius Sasnauskas
Chodon Sass
Greg Sass

Kenneth Sassaman
Jennifer Sasser
Davide Sassera
Lauren M. Sassoubre
Amen Sassy
Leandro Sastre
Jaume Sastre-Garriga
Akiko Satake
Vijaya Satchidanandam
Vijay Sathe
Mike Sathekge
D. Sather
Noppadon Sathitsuksanoh
Vidyasagar Sathuvalli
Thozhukat Sathyapalan
Seydi Ahmet Satici
Ambika Satija
Jitendra Satija
Leslie Satin
Aruna Satish
Jordan Satler
Michael Satlin
Ilhan Satman
Akira Sato
Amy Sato
Ana Paula Sato
Atsuhisa Sato
Jun Sato
Kazuhiko Sato
Keisaku Sato
Kengo Sato
Masamitsu Sato
Miho Sato
S. Sato
Satoshi Sato
Shinji Sato
Shuichi Sato
Takami Sato
Takuya Sato
Tsutomu Sato
Yasufumi Sato
Yukie Sato
Ayano Satoh
Kimio Satoh
Michihiro Satoh
Noriko Satoh-Asahara
Ieiri Satoshi
Anjali Satoskar
Yutaka Satou
Jorgina Satrustegui
Jack Satsangi
Yoko Satta
Venkata Satthenapalli
Michael Sattler
Thomas Sattler
Carmela Satumino
Kapaettu Satyamoorthy
Allebonia Satyanarayana
Ande Satyanarayana
Labani Satyanarayana
Tulasi Satyanarayana
Catherine Satzke
Apurba Sau
Narcis Saubi
Christophe Sauboin
Emil Saucan
John Sauceda
Francois Saucy
Leonor Saúde
František Saudek
Carsten Sauer
Igor Sauer
Karin Sauer
A. Elisabeth Sauer-Eriksson
Emily Sauers
Bernd Saugel
Ola Saugstad
Michael Saul
Paul Saultier
Bryan Saunders
Charles Saunders
Christobel Saunders
David Saunders
Diane Saunders
James Saunders
Jo Saunders
Kate Saunders
Luke Saunders

Manu Saunders
Michael Saunders
Ryan A. Saunders
Rachel Saunders-Pullman
Olivier Saut
Daniel Sauter
Carola Sauter-Louis
Anthony A. Sauve
Nicolas Sauvion
Helene Sauzeon
Cary Savage
Christina Savage
Eileen Savage
James Savage
Lisa Savage
Paul Savage
Ram Savan
Rashmin Savani
Warren Savary
Isabelle Savary-Auzeloux
Nazan Savas
Sureyya Savasan
Senay Savas-Erdeve
Maria Cristina Savastano
Mustafa Savci
Huub Savelkoul
Jenny Säve-Söderbergh
Tadeja Savi
Michael Savic
Miroslav Savić
Judith Savige
Mark Savill
Stephen Saville
Virginia Savin
Jean-Pierre Savineau
Carlo Savini
Tommaso Savini
Luiz Eduardo Savio
Raushan Savirov
Jonathan Savitz
Suzana Savkovic
Carmine Savoia
Francesco Savorani
Elizabeth Savory
Virginia Savova
Ioannis Savvas
Eugene Saw
Tomoo Sawabe
Kenjiro Sawada
Reiko Sawada
Susumu Sawada
Yu Sawada
Souleymane Sawadogo
Hirofumi Sawai
Nobukatsu Sawamoto
Samir Sawant
Takuya Sawaoka
Surendra Sawarkar
Tatsuya Sawasaki
Sterling Sawaya
Andrew Sawers
Gregory Sawicki
Jakub Sawicki
Vanessa Sawicki
Jason Sawyer
Kayle Sawyer
Amulya Saxena
Anjana Saxena
Deepak Saxena
Vijay Saxena
Lynora Saxinger
Andrew Saxon
Tamsin Saxton
Jong Say
Yee-How Say
Rama Prakasha Saya Saya
David Sayah
Somphou Sayasone
Danish Sayed
Mohamed Sayed-Ahmed
Anakk Sayeepriyadarshini (Sayee)
Ayman Sayegh
Mazin Sayegh
John Sayer
Yazile Sayin
Katherine Sayler
Hidetoshi Saze

Hossain Sazzad
David Sbarra
Douglas Sborov
Stefano Scabini
Simone Scacchi
Anna Scaglione
K. Scaglione
Carolina Scagnolari
Andrea Scala
Antonio Scala
Stefania Scala
Elisa Scalco
Brittan Scales
Suzie Scales
Salvatore Scali
Mariafrancesca Scalise
Marina Scalon
Fabien Scalzo
Rebecca Scalzo
Giovanni Scambia
Massimo Scandura
Elliot Scanes
Frank Scannapieco
Claire Scantlebury
Bruno Scanu
Giovanni Scapagnini
Rosa Scapaticci
Luca Scapoli
Giseppe Scarascia-Mugnozza
Giuliano Scarcelli
Joy Scaria
Carlotta Scarpa
Roberto Scarpato
Federica Scarpina
Daniel Scarr
Pietro Scaturro
Carlos Marcelo Scavuzzo
Arthur Schaafsma
Beatriz Schaan
Kyle Schachtschneider
Eva Schaden
Andreas Schadschneider
Benedikt Schaefer
Douglas Schaefer
Hanno Schaefer
Karl-Herbert Schaefer
Kurt Schaefer
Liliana Schaefer
Michael Schaefer
Niklaus Schaefer
Saul Schaefer
Tobias Schaefers
Anthony Schaeffer
Satu Schaeffer
Beat Schaer
Marissa Schafer
Michal Schafer
William Schafer
Hendrik Schäfer
Simon Schäfer
Thorsten Schäfer
Jean Schaffer
Brian Schaffner
Raffael Schaffrath
Helmut Schaider
Paula Schaiquevich
Coby Schal
Camilla Schalin-Jantti
Inga Schalinski
Casper Schalkwijk
Joost Schalkwijk
Marina Schall
Bernhard Schaller
Hubert Schaller
Stefan Schaller
Julie Schallhorn
Axel Schambach
Alessandra Schanoski
Gert Schansker
Joost Schanstra
Jacob Scharcanski
Gereon Schares
Anne Scharf
Marc Schargus
Ursula Scharler
Andrea Scharnhorst
Dieter Scharnweber
Sara Scharoun

Manfred Schartl
Helmut Schaschl
Heide Schatten
Jörn Schattenberg
Glenn Schattman
Mirta Schattner
Enid Schatz
Jonathan Schatz
Patrik Schatz
Philip Schatz
Hermann Schatzl
Monika Schaubeck
Thomas Schaubroeck
Dörthe Schaue
Frieder Schaumburg
Gustavo Schaun
Nils Helge Schebb
Mauricio Schebitz
Alan Schechter
Marcos Schechter
Gaston Schechtmann
Martin Schecklmann
Anne Scheel
Michael Scheel
Ben Scheele
Kay Scheets
Todd Scheetz
Jacob Scheff
M. Scheffer
Dirk-Jan Scheffers
Jodi Scheffler
Rudolf H. Scheffrahn
Mark Scheibler
David Scheifele
Blanca Scheijen
Jochen Schein
Meir Scheinfeld
Dustin Scheinost
Katharina Scheiter
Simone Scheithauer
Michael Schell
Huub Schellekens
E. Glenn Schellenberg
Eyk Schellenberger
Bruno Scheller
Herb Schellhorn
Robert Schelonka
Susan Schembre
Mark Schembri
Federico Schena
Diana Schendel
Bettina Schenk
Elizabeth Schenk
Gerhard Schenk
Simon Schenk
Katerina Schenke
Kimberley Schenke
Katja Schenke-Layland
Sasja Schepers
Igor Schepetkin
Caroline Schepker
Arnaud Scherberich
Laura Scherer
Philipp Scherer
Reinhold Scherer
Ronny Scherer
Margie Scherk
Ellen Scherl
Eva Schernhammer
Marie Helene Schernthaner-Reiter
Matthew D. Scherreik
Daniel Scherrer
Gregory Scherrer
Jeffrey Scherrer
Kurt Schesser
Georg Schett
Phillip Scheuerman
Richard Scheuermann
Taneisha Scheuermann
Marina Scheumann
Torsten Scheyer
Silvia Schiaffino
Lawrence Schiamberg
Monica Schiappacassi
Michela Schiavon
Andreas Schicho
Rudolf Schicho

Robert Schick
Jean-Nicolas Schickel
Silke Schicktanz
Ralf Schiebel
Matthew Schiefer
John Schieffelin
Arrigo Schieppati
Hans Schierbeek
Matías Schierenbeck
Rudolf Schierl
Andreas Schiermeyer
Matthew Schiewer
Susan Schiffman
Julia Schiffner-Rohe
Laura Schifman
Tamara Schikowski
Nathan Schilaty
Karin Schilbach
Leonhard Schilbach
Claus Schildberg
Stefan Schildknecht
Lawrence Schiller
Martin Schiller
Kurt Schilling
Oliver Schilling
Tobias Schilling
Pablo Schilman
Michael Schilsky
John Schimenti
Bart Schimmel
Adriano Schimmenti
Dominic Schinagl
Allan Schinckel
Christian Schindelhauer
Rainer Schindl
Andreas Schindlbacher
Charles Schindler
Karin Schindler
Bernhard Schink
Tania Schink
Alison Schinkel-Ivy
Ernestina Schipani
Lieke Schiphof-Godart
Jochen D. Schipke
Serena Schippa
Chiara Schiraldi
Peter Schirmbacher
Annett Schirmer
Eudoxia Schismenou
Johannes Schittny
Katrine Schjoldager
Robert Schlaberg
Thomas Schlacher
David Schlaepfer
Karl Schlag
Axel Schlagenhauf
Oliver Schlager
Michael Schlame
Michael Schläppi
Robert Schlauch
Elizabeth Schlaudecker
Geraldine Schlecht-Louf
Nicolas Schlegel
Paul Schlegel
Erwin Schleicher
Gudrun Schleiermacher
Yosef Schlein
Dorit Schleinitz
Mark Schleiss
Daniel Schlenk
Evelyn Schlenker
Christian Schlenstedt
Sabrina Schlesinger
Kai Schleutker
Michael Schleyer
Paul Schliekelman
Christian Schlieker
Georg Schlieper
Edgar Schliephake
Henning Schliephake
Fernando Schlindwein
Romy Schlögel
Bruce Schlomer
Joel Schlosburg
Karen Schloss
Dietmar Schlosser
Gerhard Schlosser
Thomas Schlösser

Tom Schlösser
Michael Schloter
Florian Schlotter
Christian Schlötterer
Ursula Schlötzer-Schrehardt
Anna-Barbara Schlüer
Klaus-Dieter Schlueter
Nadine Schlueter
Neil Schluger
Yolanda Schlumpf
Vivi Schlünssen
Vera Schlüssel
Christian Schluter
Fredrik Schlyter
Ralph Schmaelz
Alvin Schmaier
Karen Schmaling
Gottfried Schmalz
Rosemarie Schmandt
Daniel Schmehl
Michael Schmeltz
Dénes Schmera
Leopold Schmetterer
Thomas Schmickl
Florian Schmid
Jan Schmid
Jeffrey Schmid
Katrina Schmid
Matthias Schmid
Philipp Schmid
Scott Schmid
Sebastian Schmid
Silvan Schmid
Sophie Schmid
Stefan Schmid
Doreen Schmidl
Ana Lucia Schmidt
Andrea Schmidt
Angelika Schmidt
Anja Schmidt
Ansgar Schmidt
Antje Schmidt
Birgit Schmidt
Börge Schmidt
Christiane Schmidt
Christoph Schmidt
Cory Schmidt
Daniel Schmidt
Eric Schmidt
Felix Schmidt
Francis Schmidt
Jean Schmidt
Joshua Schmidt
Klaus Schmidt
Kristina Schmidt
Krzysztof Schmidt
M. K. Schmidt
Maria Schmidt
Martin Schmidt
Michael Schmidt
Monica Schmidt
Olaf Schmidt
Paul Schmidt
Robert Schmidt
Sabine Schmidt
Sebastian Schmidt
Stefan Schmidt
Thomas Schmidt
Warren Schmidt
Ursula Schmidt-Erfurth
Sebastian Schmidtlein
Heike Schmidt-Posthaus
Florian Schmiedek
Brian Schmit
Andreas Schmitt
Daniel Schmitt
Laetitia Helene Marie Schmitt
Lauren Schmitt
Gerd Schmitz
Hendrik Schmitz
Ingo Schmitz
Lenon Schmitz
Mary Schmitz
Melodie Schmitz
Thomas Schmitz
Ulf Schmitz
Tanja Schmitz-Hübsch
Christopher D. Schmoutz

Landon Schnabel
Renate Schnabel
Robert Schnabel
Rebecca Schnall
Jörg Schnecker
Daniel Schneditz
Karin Schneeberger
Mark Schneegurt
John S. Jr Schneekloth
Olaf Schneewind
Michael Schneide
Andre Schneider
Augusto Schneider
Barbara Schneider
Bohdan Schneider
Bryan Schneider
Daniela Schneider
David Schneider
Dona Schneider
Frank M. Schneider
Hans Gerhard Schneider
Jay Schneider
Jesper Schneider
Juliane Schneider
Kai Markus Schneider
Kate Schneider
Laura Schneider
Margaret Schneider
Markus Schneider
Mathias Schneider
Maurício Schneider
Richard Schneider
Sabine Schneider
Stanley Schneider
Uwe Schneider
Victor S. Schneider
William Schneider
Yves-Jacques Schneider
Henriette Schneider-Haßloff
Deborah Schneiderman
Casey Martin Schneider-Mizell
Jürgen Schneider-Schaulies
David Schnell
Jordan Schnell
Susanne Schnell
Robert Schnepp
Sebastian Schnettler
Stephan Schneuwly
Armin Schnider
Kathryn Schnippel
Jason Schnittker
Hans Schnittler
Frank Schnorrer
Stefan Schob
Andreas Schober
Joseph Schober
Robert Schoborg
Frieder Schöck
H. Schoechl
Felix Schoeller
James Schoelz
Marina Schoemaker
Corrie Schoeman
Christoph Schoen
Victor Schoenbach
Michael Schoenbaum
Daniel Schoene
Manuela Schoenenberger
Alan Schoenfeld
David Schoenfeld
Mark H. Schoenfisch
Mark Schoenfisch
Florian Schoenhoff
Laura Schoenle
U. Joseph Schoepf
Verena Schoepf
Randal Schoepp
Brett Schofield
Casey Schofield
Christopher Schofield
Gail Schofield
Jesse Schold
Shaun Scholes
M. Scholey
Ludger Schöls
Carsten Scholz
Christopher Scholz
Holger Scholz

Markus Scholz
Tomas Scholz
Chris-Carolin Schön
Daniele Schön
Jennifer Schön
Sara Schonfeld
Mirco Schönfeld
Pia Schönfeld
Gabriele Schönian
Johanna Schonrock-Adema
Axel Schönthal
Valerie Schoof
Mario Schootman
Sebastian Schoppmann
John Schorge
Benedikt Schoser
Angelika Schoster
Klaas Schotanus
Helena Schotland
Michael Schotsaert
Gunnar Schotta
Ulli Schotten
Catherine Schouler
Bruno Schoumaker
Tony Schountz
John Schousboe
Judith Schouten
Jennifer A. Schrack
Maarten Schrama
Peter Schramm
R. Schramp
Eric Schrauben
Ingrid Schraufstatter
Monika Schreckenbach
Adrian Schreiber
Barbara Schreiber
Karl Schreiber
Martin Schreiber
Merritt Schreiber
Sanja Schreiber
Pamela Schreiner
Sabrina Schreiner
Thomas Schreiner
Michael Schreinlechner
Eric Schreiter
Bernard Schreurs
Aaron Schrey
Colleen C Schreyer
Karin Schrieber
Albert Schriefer
Michael Schroda
Jaap Schroder
Christin Schröder
Martin Schröder
Sara Schröder
Stine Schrøder
Julia Schroders
Michael Schrödl
Dana Schroeder
John Schroeder
Lee Schroeder
Natalia Schroeder
Ralph Schroeder
Sven Schroeder
Ulrich Schroeders
Tracy Schroepfer
Ronald Schroeter
Stephen Schroeter
Henning Schroll
Helmut Schrom-Feiertag
Martien Schrooten
Martine Schroyen
Jacob Schrum
Andras Schubert
Grit Schubert
Thomas Schubert
Tilman Schubert
Florian Schuchardt
Amie Schuck
Kathrin Schuck
Wolfgang Schuehly
Anne Schueler
Emil Schueler
Amy Schueller
Jan Schuemann
Mark Schuenke
Wiebke Schuett
Ursel Schuette

Heike Schuetze
Scott Schuetze
Stephan Schug
Klaus Schughart
Amy Schuh
Jane Schuh
Michael Schulder
Chrisitine Schuler
Gerhard Schuler
Hannes Schuler
Michael Schuler
Rainer Schuler
Dirk Schüler
Hildegard Schuller
Kathy Schuller
Christoph Schüller
Stephanie Schüller
Sam Schulman
Joshua Schulman-Marcus
Bruce Schulte
Michael Schulte
Mieke Schulte
Patricia Schulte
Albrecht Schulte-Hostedde
Holger Schultheis
Maximilian Schultheiss
Bettina Schulthess
Alyssa Schultz
Cheryl Schultz
Julia Schultz
Michael Schultz
Tim Schultz
William Schultz
Anna Schultze
Elke Schulz
Geralyn Schulz
Joerg Schulz
Martin Schulz
Rudiger Schulz
Wolfgang Schulz
Juliane Schulze
Ralf Schulze
Torsten Schulze
Gundula Schulze-Tanzil
Sven Schulzke
Ellen Schulz-Kornas
Jeanette Schulz-Menger
Anne Schumacher
Fredrick Schumacher
Stefan Schumacher
Tracy Schumacher
Brant Schumaker
Ronnie Schumann
Kate Schunke
Heribert Schunkert
Celia Schunter
Jorg Schupbach
Gertraud Schüpbach-Regula
Jonas Schupp
Marcus-Oliver Schupp
Hans-Christian Schuppe
Peter Schur
Mark Schurdak
Claudia Schurmann
Amos Schurr
Michael Schurr
Alexander Schuster
Jessica Schuster
Holger Schutkowski
Russell Schutt
Stephanie Schuttler
Charlotte Schutz
Michael Schutz
Mark Schutze
Heike Schütze
Marieke Schuurmans
Clarissa Schwab
Helmut Schwab
Jan Schwab
Julian Schwab
Markus Schwab
Nicholas Schwab
Annette Schwabe
Justus Schwabedal
Martin Schwacha
Hermann Schwameder
Adrian Schwan
G. Schwan

Hannes Schwandt
Sherri Schwaninger
Jason Schwans
Helen Schwantje
David Schwappach
Robert Schwarcz
Angela Schwarm
Benjamin Schwartz
Bradley Schwartz
Gregory Schwartz
Jacob Schwartz
Joel L. Schwartz
John Schwartz
Peter Schwartz
Richard G. Schwartz
Sheree Schwartz
Todd Schwartz
Tonia Schwartz
Judith Schwartzbaum
Eyal Schwartzberg
Michael Schwartze
Kevin Schwartzman
Edward Schwarz
Eli Schwarz
Jaclyn Schwarz
Katharina Schwarz
Kathleen Schwarz
Sandra Schwarz
Stefan Schwarz
Theresa Schwarz
Christopher Schwarzbach
Margaret Schwarze
Adrian Schwarzer
D. Samuel Schwarzkopf
Michael Schwarzl
Uli Schwarz-Linek
David Schwebel
Kathy Schwehr
Nicolas Schweighofer
Darius Schweinoch
Andrew Schweitzer
Carrie Schweitzer
Magali Schweizer
Matthias Schweizer
Michael Schweizer
Michael Schwemm
Lars Ole Schwen
Holger Schwender
Falk Schwendicke
Kathryn Schwertfeger
A. J. Schwichtenberg
Charles Schwieters
Sharon Schwille
Patrick Schwing
Andreas Schwingshackl
James Schwob
Christian Schyllert
Alessandro Sciahbasi
Andrea Sciarretta
Sebastiano Sciarretta
Christina Scifres
Marco Scioscia
Carlo Scirè
Anthony Sclafani
Valentina Sclafani
R. Hal Scofield
Monica Scognamiglio
John Scolaro
Alison Scope
Kézia Scopel
Diana Scorpio
Marco Scortichini
Antonella Scorziello
Bryan Scotney
David Scott
Evan Scott
Fraser Scott
Jeremy A. Scott
Jessica Scott
John Scott
Kirsten Scott
Lesley Scott
Molly Scott
Nancy Scott
Nick Scott
Paul Scott
Peter Scott
Stephen Scott

Susana Scott
Lorenza Scotti
Nicholas Scott-Samuel
Isabella Screpanti
Marianna Scrima
Olga Scrivner
Ricardo Scrosati
Caroline E. Scruggs
Domenico Scrutinio
Mark Scudder
Margaret Scull
Laura Scullion Hall
Nicholas Scurich
Angelo Scuteri
Steven Scyphers
Andrei Sdrulla
Haitham Seada
Marc Seal
Lucia Seale
Judith Sealy
Thomas Seamans
Giorgio Seano
Grant Searchfield
William Searcy
Catherine Searle
Iain Searle
Kelly Searle
Edmund Searles
Catherine Sears
Derek Sears
S. J. Seashols-Williams
Timothy Seastedt
James Seaward
Alvaro Sebastian
Martin Sebastian
Shaji Sebastian
Siby Sebastian
Esther Sebastián-González
Federico Sebastiani
Giada Sebastiani
Guido Sebastiani
M. Sebastiani
Paola Sebastiani
Irene Sebastianutto
Mireille Sebbag
Alexandre Magno Sebbenn
Marek Sebela
Silvia Secchi
Paola Secchiero
David Secco
Annalisa Sechi
Ousman Secka
Ahmad Sedaghat
John Sedbrook
Anna Sedda
Jennifer Seddon
Philip Seddon
Christopher Seder
Karim Sedky
Jan Sedlacik
Thomas Sedlak
Cara Sedney
Chan Hwang See
Chee Gee See
Kelvin See
Sarah See
Violaine See
J. E. Seeb
Frank Seebacher
Trine Seeberg
Brian Seed
Adam Seegmiller
Matthew Seeley
Dominik Seelow
Andrew Seely
Ego Seeman
Arivudainambi Seenichamy
Yuwalee Seesao
Arpamas Seetasith
Mostafa Sefidgar
Leopoldo Segal
Mark Segal
Nicolas Segal
Jeffrey Segar
Lakshman Segar
Ana Lucia Segatto
Christopher Sege
Kerri Seger

Suzanne Segerstrom
Mohamed Seghier
Abd-Krim Seghouane
Tebogo Segolodi
Lorenzo Segovia
Vanesa Segovia
Colin Segovis
Chris Segrin
Marc Seguin
Nicole Seguin
Ana Segura
Maira Rubi Segura Campos
Marta Segura Valverde
Juan Segura-Aguilar
Deepmala Sehgal
Herman Sehmbi
Stephan Sehmisch
Bettina Sehnert
Sebastian Seibold
Verena Seibold
Nabil Seidah
Michael Seidel
Michael Seidenbusch
Dara E. Seidl
Rachael Seidler
Stephanie Seidlits
Daniel Seidman
Ernest Seidman
Gabriel Seidman
Mark Seielstad
Magy Seif El-Nasr
David Seifer
Colleen Seifert
Erin Seifert
Michael Seifert
Thomas Seifert
Moritz Seiffert
Ali Seifi
Alexander Seiler
Gabriela Seiler
Gerald Seiler
Roger Seiler
Titus Seilheimer
Michael Seimetz
Yutaka Seino
Ryan Seipke
Elena Seiradake
Sophie Seite
Hervé Seitz
Stefan Seitz
Barbara Seitz-Polski
Peter Seizer
Haruna Sekabira
Moorine Sekadde
Juliet Sekandi
Denise Sekaquaptewa
Ramalingam Sekar
Sukran Seker
Nigar Sekercioglu
Motoaki Seki
Shu Seki
Mitsuhiro Sekiguchi
Takahiro Sekiguchi
Chiyoko Sekine
Joji Sekine
Yasuyo Sekiyama
Damir Sekulic
John Osei Sekyere
Shifra Sela
Zeliha Selamoglu
Katri Selander
Duygu Selcen
Paul Selden
Steven Self
Brian Selgrade
Paul Seli
Hervé Seligmann
Abdelfattah Selim
Sameh Selim
Luciano Selistre
Manuela Selitto
Sabrina Selk
Ana Maria Sell
Rebecca Sell
Jeremie Sellam
Jacobo Sellares
Roberta Sellaro
Dyan Sellayah

Julia Sellers
Damien Sellier
Lorenz Sellin
Donald Sellitti
Michael Sellix
Frank Sellke
Johann Sellner
Colin Selman
Lucy Selman
Will Selman
Jonas Selmeryd
Joshua Selsby
Jean-Paul Selten
Maria Selvaggi
Periasamy Selvaraj
Sridhar Selvaraj
Sudhakar Selvaraj
Dinesh Selvarajah
Christine Selvey
Innocent Semali
Richard Semba
Aggrey Semeere
Christina Semeniuk
Ekaterina Semenova
Gregg Semenza
Gianpietro Semenzato
Francesco Semeraro
Michael Seminelli
Elena Semino
Jeff Seminoff
Matthew Semler
Joanna Semlyen
Oliver Semmes
Joseph Sempa
John Semple
Stuart Semple
Katherine Semrau
Margaret Semrud-Clikeman
Aditya Sen
Bisakha Sen
Dwaipayan Sen
Parimal Sen
Pradip Sen
S. Sen
Shaunak Sen
Shilpa Sen
Taner Sen
Emily Sena
Shantibhusan Senapati
Senait Senay
Kacper Sendra
M. Sendra
Marcela Sene_Fiorese
Martin Senechal
Marjanne Senekal
Julien Seneschal
Giorgio Senesi
Mathias Senge
Ryan Senger
Mathilde Sengoelge
Frank Sengpiel
Aditi Sengupta
Sadhak Sengupta
Sejuti Sengupta
Shiladitya Sengupta
Sonali Sengupta
Sumita Sengupta
(Bandyopadhyay)
Abdulkadir Şengür
Stephen Senn
Bilal Senol
Ladislav Šenolt
Carlo Senore
M. Sensi
Stefano Sensi
Enrique Sentandreu
Leigha Senter
Loïc Sentilhes
Hiroshi Sentsui
Mehmet Senturk
Mert Senturk
Serif Senturk
Brigitte Senut
Hideaki Senzaki
Sawa Senzaki
Byung-Kwan Seo
Cheong Hoon Seo
Dong-Woo Seo

Kyoung Yul Seo
Minah Seo
Mitsunori Seo
Seongjin Seo
Young Ah Seo
Young-Su Seo
Jose A. Seoane
Enrique Seoane-Vazquez
Yeong-Jae Seok
Han Seol-Heui
Jae Young Seong
Moon-Woo Seong
Kok-Min Seow
Ali Sepahdari
Frances Separovic
Hessam Sepasi
Romina Sepe
Zahra Sepehri
Perttu Seppä
Alison September
Edwards Septimus
Maritza Sepúlveda
Osvaldo Jhonatan Sepulveda Villet
Fernando Sequeira
Guillermo Sequera
Božena Šerá
Gianluca Serafini
Zeba Seraj
Bertrand Seraphin
Florina Serbanescu
Mirgissa Serbessa
Natalia Serbina
Tom Serby
Michael Sereda
Fabrizio Serena
Chanuki Seresinhe
Albrecht Serfling
Inna Serganova
Gerard Sergeant
Simona Serini
Giovanna Serino
Matteo Serino
Silvia Serino
Kirill Serkh
Mireille Serlie
Walter Sermeus
Ainhoa Serna
Evelyne Sernagor
Joao Serodio
Ary Serpa Neto
Marcelo Serpe
Louise Serpell
Engin Serpersu
Andreas Serra
Margherita Serra
Michael Serra
Monica Serra
Raffaele Serra
Ruth Serra-Moreno
Daniel Serrani Azcurra
Jose Serrano
Karine Groenen Serrano
María Serrano
Oscar Serrano
Ricardo Serrano
Manuel Serrano Ríos
Jesús Serrano-Luna
Antonio Serrano-Mislata
Carmelo Serrantino
Aurelio Serrao
Ester Serrao
José Eduardo Serrão
Robert Serrouya
Igor Sersa
Elisavet Serti
Sarah Sertic
Martina Šeruga Musić
Laurent Servais
Nicolas Servant
Susan Sesack
Thea Sesardic
Ramakrishnan Seshadri
Newton Sesma
Paola Sessa
Francesca Seta
Vijayasaradhi Setaluri
Ajay Seth

Pankaj Seth
Ratanesh Seth
Ricky Sethi
Sanjay Sethi
Sidharth Sethi
Tavpritesh Sethi
Varun Sethi
Vaheshta Sethna
Kandan Sethumadhavan
Didik Setiawan
Keamogetse Setlhare
Sunita Setlur
J. Seto
Karen Seto
Michael Seto
Wai-Kay Seto
Yasuyuki Seto
Giuseppina Settanni
Paola Sette
Francesco Setti
William Setzer
Héctor Seuánez
Janina Seubert
John Seubert
Min Seung-Kee
Sabina Sevcikova
Nick Sevdalis
Sanja Sever
Feride Severcan
Alberto Severini
Carlo Severini
Konstantin Severinov
David Severs
John Severson
Rachel Severson
William Severud
Turker Sevgi
Noemí Sevilla
Daniel Sevilla-Sanchez
Timur Sevincer
Caroline Sevoz-Couche
Katherina Sewald
Norbert Sewald
Nadine Seward
William Sexauer
Claire Sexton
Alex Sextonoates
Cheikh Seye
Mirjalili Seyedali
Seyedmojtaba Seyedmousavi
John Seymour
Kiley Seymour
Olivier Seynnes
Volkan Seyrantepe
Uğur Sezerman
Spyros Sfenthourakis
Ioannis Sfontouris
Chiarella Sforza
Fabio Sgolastra
Giovanni Sgubin
Antonella Sgura
Aihua Sha
Wenwen Sha
Monir Shababi
Andrey Shabalin
Svetlana Shabalina
Nadia Shaban
Farzin Shabani
Sakineh Shab-Bidar
Asad Shabbir
Yakov Shacham
S. Shachar
Shay Shachar
Kenneth Shackel
Starley Shade
William Shadel
Aaron Shafer
Jenifer Shafer
Robert Shafer
Tim Shafer
Zachary Shaffe
Jonathan Shaffer
Ronald Shaffer
Tariq Shafi
Sanaz Shafian
Anum Shafiq
Sheikh Shafiq
M. Shafiq-Ur Rahman

Sharoni Shafir
Bridget Shafit-Zagardo
Ashesh Shah
Devendra Shah
Dhara Shah
Dilip Shah
Hemangi Shah
Jasmit Shah
Jigna Shah
Jyotsna Shah
Kairav J. Shah
Karan Shah
Kavita Shah
Kushani Shah
Maulin Shah
Minesh Shah
Monica Shah
Neeraj Shah
Nirali Shah
Nirmish Shah
Parantu Shah
Payal Shah
Prachi Shah
Raj Shah
Rashed Shah
Shailee Shah
Shiraz Shah
Sudhin Shah
Tayyab Shah
Trushil Shah
Zahoor Shah
Saleh Shahabivand
Shai Shaham
Yavin Shaham
Uma Shahani
Ron Shahar
Syahrul Sazliyana Shaharir
Muhammad Shahbaz
Forooz Shahbazi
Sina Shahbazmohamadi
Ameer Shaheed
Tharwat Shaheen
Mohammad Shahid
Muhammad Shahid
Saleem Ullah Shahid
Shaouli Shahid
Bahar Shahidi
Mimoza Shahini
Mohsen Shahlaei
Linah Shahoumi
Saeid Shahraz
Vahid Shahrezaei
Rifat Shahriyar
Mohammad Shahrokh Esfahani
A. Shai
Abjal Shaik
Saleem Shaik
Mateen Shaikh
Nida Shaikh
Saijuddin Shaikh
Kathy Shair
Natan Shaked
Pninit Shaked-Mishan
Muhammad Shakeel
Joseph Shaker
Heman Shakeri
Judy Shakespeare
Tom Shakespeare
Holly Shakya
Tara Shakya
Ali S. Shalash
Edith Shalev
Idit Shalev
Nadav Shalit
Guy Shallom
Joel Shalowitz
Barjadze Shalva
Lior Shamir
Mehrdad Shamloo
Farid Shamo
Rawand Shamoon
Changjuan Shan
Hongli Shan
Libo Shan
Shan Shan
Weixing Shan
Xiaoyi Shan
Y. Shan

Yi Shan
Zhongyan Shan
Kwame Shanaube
Asifkhan Shanavas
Siddharth Shanbhag
Aryeh Shander
Andi Shane
Matthew Shane
Nathan Shaner
Pei-Jen Shaner
Ce Shang
Fanhua Shang
Jing Shang
Jingwei Shang
Limin Shang
Pengjian Shang
Ronghua Shang
Shi Shang
Xuejun Shang
Xuequn Shang
Donghui Shangguan
Hariharan Shankar
Karthik Shankar
Ravi Shankar
S. Shankar
Sreejith Shankar Pooppanal
Leslie Shanks
Orin Shanks
Robert Shanks
Erin Shanle
K. Shanmuganandan
Sabesan Shanmugavelu
Santhanam Shanmughapriya
Jonathan Shannahan
Lynne Shannon
Oliver Shannon
Lisa M. Shantz
Bing Shao
Changwei Shao
Chun-Kui Shao
Di Shao
Guofan Shao
Hui Shao
Jianlin Shao
Jian-Zhong Shao
Kwang-Tsao Shao
Ming Shao
Qing Shao
Tao Shao
Ying Shao
Yi-Ta Shao
Yongqi Shao
Yu-Yun Shao
Zeshu Shao
Philip Shapira
Adrienne Shapiro
Arthur Shapiro
Charles Shapiro
Erik Shapiro
Eugene Shapiro
James Shapiro
Karen Shapiro
Kimron Shapiro
Lawrence Shapiro
Leo Shapiro
Paul Shapiro
David Shapiro-Ilan
Deepak Sharan
Mahmoud Sharara
Maziar Sharbafi
Melissa Sharer
Yaron Shargall
Nikki Shariat
Behzad Shariati
Adnan Sharif
Ariane Sharif
Behnam Sharif
Bonita Sharif
Jafar Sharif
Leila Sharif
Muhammad Sharif
Rabab Sharif
Salma Shariff-Marco
Atena Sharifi-Rad
A. Sharma
Aditi Sharma
Ajitha Sharma
Akshay Sharma

Amit Sharma
Amitabh Sharma
Anamika Sharma
Anil Sharma
Anket Sharma
Anupama Sharma
Arpeeta Sharma
Arun Sharma
Ashish Sharma
Atul Sharma
Balram Sharma
D. K. Sharma
Deep Sharma
Dharmesh Sharma
Dipali Sharma
G. Taru Sharma
Gaurav Sharma
Geetanjali Sharma
Gyan Sharma
Harshita Sharma
Ketan Sharma
L. N. Sharma
Lakesh Sharma
Mahak Sharma
Mamta Sharma
Manu Sharma
Monisha Sharma
Mukut Sharma
Navneet Sharma
Nilesh Sharma
Nilima Sharma
Nitin Sharma
Prabhat Sharma
Pradeep Sharma
Priyanka Sharma
Rahul Sharma
Rakesh Kumar Sharma
Rakesh Sharma
Ram Kumar Sharma
Ramaswamy Sharma
Rameshwar Sharma
Ratika Sharma
Reecha Sharma
Rekha Sharma
Robin Sharma
Sadarshana Sharma
Sai Kiran Sharma
Sandeep Sharma
Sanjita Sharma
Sharan Sharma
Shiva Sharma
Shivani Sharma
Shiwani Sharma
Shyam Sharma
Sudesh Sharma
Sunny Sharma
Surendra Sharma
Suresh Sharma
Tarun Sharma
Tilak Sharma
Uma Sharma
Umesh Sharma
Vijay Sharma
Vishal Sharma
Vivekanand Sharma
Yogendra Sharma
Neelam Sharma-Walia
Ashoke Sharon
Michal Sharon
Carla Sharp
Christine Sharp
Claire Sharp
Frank Sharp
Gemma Sharp
Jon Sharp
Julie Sharp
Paul Sharp
Tyler Sharp
Louise Sharpe
Martyn Sharpe
Richard Sharpe
Adam Sharples
Christopher Sharpley
Tarek Sharshar
Robert Sharwood
Ajit Shasany
Mohamed Tarek Shata
Ivan Shatsky

Oleg Shatursky
John Shaver
Andrey Shaw
Blanka Shaw
Bronwen Shaw
Bryan Shaw
Calvin Shaw
Christopher Shaw
Dana Shaw
Dennis Shaw
Edward Shaw
Eric Shaw
Evelyn Shaw
George Shaw
Gerry Shaw
J. A. Shaw
Jeremy Shaw
Jonathan Shaw
Lindsey Shaw
Natalie Shaw
Pang-Chui Shaw
Philip Shaw
Richard Shaw
Robin Shaw
Robyn Shaw
Rosalind Shaw
Santosh Shaw
Shih-Lung Shaw
Stacey Shaw
Steven R. Shaw
William Shawlot
Md. Shajedur Rahman Shawon
Anna Shawyer
Dmitry Shayakhmetov
Renat Shaykhiev
Natalia Shcherbina
N. A. Shchipanov
David She
Maoyun She
Barry Shea
John Shea
Kimberly Shea
Patrick Shea
Steven Shea
Thomas Shea
Will Shead
David Shealer
Richard Sheardy
Darlene Shearer
Glen Shearer
Keith Shearwin
Bryony Sheaves
Laura Sheble
David Shechter
Orit Shechtman
Emma Sheehan
John Sheehan
Katie Sheehan
Penelope Sheehan
Riley Sheehan
Terrence Sheehan
Vivien Sheehan
Grace Sheehy
Jennifer Sheehy-Skeffington
Shyr-Ming Sheen-Chen
Patrick Sheets
Sandra J. Shefelbine
Richard Shefferson
Orit Shefi
Hosam Sheha
Nader Sheibani
Richard Sheibley
Alaullah Sheikh
Farah Sheikh
Javaid Sheikh
Kazim Sheikh
Mashhood Sheikh
Taiwo Sheikh
M. Sheikholeslami
Orla Sheils
Jonathan Sheindlin
Stephen Sheinkopf
Paul Shekelle
G. S. Shekhawat
Louis Shekhtman
Kevin Shelburne
Eric Shelden
Kim Shelden

I. Sheldon
Jessica Sheldon
Christopher Sheldrick
Miriam Shelef
Ekaterina Shelest
Mack Shelley
Todd Shelly
James Shelton
Carrie Shemanko
C. Shen
Cangliang Shen
Chaopeng Shen
Che-Kun Shen
Chenjia Shen
Chiayi Shen
Chuanliang Shen
Dehua Shen
Feng Shen
Fo-Ting Shen
Guohua Shen
Guozhen Shen
Hao Shen
Hao-Qing Shen
Hong Shen
Hui Shen
Jianlin Shen
Jianwei Shen
Jieli Shen
Jing Shen
Jingshi Shen
John Shen
Kelly Shen
Li Shen
Lin Shen
Megan Shen
Mingyi Shen
N. Shen
Qiang Shen
Qingtang Shen
Qirong Shen
Rong Shen
Shihua Shen
Tang-Long Shen
Te-Chun Shen
Wei Feng Shen
Wei Shen
Xiaoying Shen
Xinlian Shen
Xu Shen
Yang Shen
Yi Shen
Ying Shen
Youxin Shen
Yuan Shen
Z. Shen
Zhesi Shen
Zhigang Shen
Zhong-Jian Shen
Vikram Shende
Bin Sheng
Changsheng Sheng
Jianqiu Sheng
Quanhu Sheng
Weian Sheng
Yang Sheng
Justin Shenje
Donald Shepard
Peter Sheperdson
Bryan Shepherd
Greene Shepherd
John Shepherd
Megan Shepherd
Amanda Sheppard
Amy Sheppard
Chris Sheppard
Donald Sheppard
James Sheppard
Lawrence Sheppard
Paula Sheppard
Anna Sher
Roger Sher
Yuh-Pyng Sher
Gajanan Sherbet
Renslow Sherer
Sukhwinder Shergill
Brian Sheridan
Megan Sheridan
Laguinn Sherlock

Lori Sherlock
Arthur Sherman
David Sherman
Jodi Sherman
Kerry Sherman
Lauren Sherman
Phillip Sherman
Ryne Sherman
Sue Sherman-Broyles
Stanton Keith Shernan
Lorraine Sherr
Frances Sherratt
Michael Sherratt
Catherine Sherrington
William Sherwin
David Sherwood
Edward Sherwood
Mark Sherwood
Patrick Sherwood
Richard Sherwood
Namratha Sheshadri
Mark Sheskin
Anna Shestakova
Anandi Sheth
Sameer Sheth
Nagaraj Shetti
Shrimati Shetty
Pradip Shetye
Chau-Chyun Sheu
Sheng-Hsiung Sheu
Shwu-Jiuan Sheu
Lalita Shevde
Dmitry Shevela
Yuri Y. Shevelyov
Mark Shevlin
Maxim Shevtsov
Hemant Shewade
Muki Shey
Ainong Shi
Baochen Shi
Cynthia Shi
D. Shi
Da-Zhuo Shi
Dee Shi
De-Li Shi
Feng Shi
Fu-Dong Shi
Gangrong Shi
Gongle Shi
Guo-Ping Shi
Haifei Shi
Hao Shi
Hong Shi
Huai-Long Shi
Huaizhong Shi
Huazhong Shi
Hui Shi
Jie Shi
Jiong Shi
Jishu Shi
Jun Shi
Junqing Shi
Lijun Shi
Liyun Shi
Luwen Shi
Min Shi
Nan Shi
Pei-Yong Shi
Peng Shi
Qiong Shi
Qizhen Shi
Rongguang Shi
Run Zhang Shi
Runmin Shi
Shaoping Shi
Shengjing Shi
Shengqing Shi
Shengyou Shi
Tujin Shi
Tuo Shi
Weibin Shi
Weiyu Shi
Wenyin Shi
Xiaoguang Shi
Xiaojie Shi
Xiaoyang Shi
Xuan Shi
Y. Shi

Yang Shi
Yeyin Shi
Yimin Shi
Yongquan Shi
Yongyong Shi
Yufei Shi
Yuguang Shi
Yulan Shi
Yunyu Shi
Yuqian Shi
Z. H. Shi
Zhanghong Shi
Zhumei Shi
Shine-Gwo Shiah
Chih-Chung Shiao
Stephen Shiao
Stephanie Shiau
Yuo-Hsien Shiau
Daisuke Shiba
Koichiro Shiba
Yugo Shibagaki
Koji Shibasaki
Darryl Shibata
Hiroshi Shibata
Hirotaka Shibata
Hiroyuki Shibata
Shigenobu Shibata
Toshio Shibata
Yoko Shibata
Jamil Shibli
Caroline Shiboski
Makoto Shibutani
Akira Shibuya
Kei Shibuya
Naoto Shibuya
Frederick Shic
Farzad Shidfar
Sarah Shidgar
Yoshihiro Shidoji
Adrian Shieh
Carol Shieh
Chun-Chien Shieh
Renee Shield
Anthony Shields
Cleveland Shields
Michael Shields
Robert Shields
Shannon Shields
Vonnie Shields
Clive Shiff
Michael Shiflett
Tsuyoshi Shiga
Kunihiro Shigematsu
Kazuhiro Shigemoto
Katsumi Shigemura
Kohei Shigeta
Arthur Chun-Chieh Shih
Chilin Shih
Helen Shih
Huang-Chia Shih
Hung-Jen Shih
Ie-Ming Shih
Kendrick Co Shih
Ludy Shih
Wei-Chuan Shih
Wen-Ling Shih
Shuichiro Shiina
Teiichiro Shiino
Ronald Shikiya
Nicole Shilkofski
Alicia Shillington
Jae-Jun Shim
David Shima
Hideaki Shimada
Hiroaki Shimada
Hiroyuki Shimada
Kazunori Shimada
Masayuki Shimada
T. Shimada
Takehiko Shimada
Tomohiro Shimada
Yusuke Shimakawa
Satoshi Shimamura
Takeshi Shimamura
Hitoshi Shimano
Shigeki Shimba
Hideaki Shimizu
Hideki Shimizu

Ippei Shimizu
Kimiya Shimizu
T. Shimizu
Takahiko Shimizu
Tomoharu Shimizu
Toru Shimizu
Yuuki Shimizu
Yuko Shimizu-Motohashi
Shigeto Shimmura
Kazuya Shimoda
Masayuki Shimoda
Shingo Shimoda
Joshua Shimony
Derya Shimshek
Aesun Shin
Chan Young Shin
Chanseok Shin
Damian Shin
Dong Wook Shin
Hainsworth Shin
Hye-Won Shin
Hyung-Ik Shin
Jae-Won Shin
Jinwook Shin
Jong-Yeon Shin
Kwang-Soo Shin
Min-Ho Shin
Sung Jae Shin
Teo Jeon Shin
Yong Shin
Marwan Shinawi
Arti Shinde
Satomi Shinde
Vishal Shinde
Vivek Shinde
Kenneth Shindler
Yoichiro Shindo
Hideo Shindou
James Shine
Jennifer Shing
Takuya Shinjo
Reiko Shinkura
Ken Shinmura
Douglas Shinneman
Mary Ann Shinnick
Cristiane Shinobu Mesquita
Y. Shinohara
Yoshiaki Shinohara
Shinichiro Shinzaki
Takashi Shinzato
Tatsuo Shioda
Daisuke Shiomi
Masahiro Shiomi
Hiroyuki Shiono
Masakazu Shiota
Seiji Shiota
Akiko Shiotani
Bill Shipley
Ceri Shipton
Koji Shirai
Yuichiro Shirai
Atsushi Shiraishi
Kenta Shirasawa
Katsuhiro Shiratake
Yukihiko Shirayama
Abolfazl Shirazi
Sajjad Shirazi
Tahmina Shirin
Alireza Shirneshan
Roman Shirokov
Eric Shiroma
Helio Shiroma
Paulo Shiroma
Priyattam J. Shiromani
C. Shirong
Yuichiro Shirota
Ben Shirt-Ediss
Elena Shishkina
Vladimir Shishov
Egor Shitikov
Sruti Shiva
Thimmappa Shivanandappa
Amol Shivange
K. R. Shivanna
Carolyn Shivers
Kalyanam Shivkumar
Chelsey Shivley
Nirajan Shiwakoti

Ziv Shkedy
Alena Shkumatava
Judith Shlay
Netalie Shloim
Amir Shlomai
Mark Shlomchik
Adrienne Shnier
Muhammad Shoaib
Clinton Shock
Keith Shockley
Yehuda Shoenfeld
David A. Shoham
Menachem Shoham
E. Shohami
Kyrillus Shohdy
Iman Shojaei
S. Reza Shojaei
Shahla Shojaei
Mohammad Shojafar
Hirotaka Shoji
Ikuo Shoji
Kazuhiro Shoji
Kotaro Shoji
Masaki Shoji
Yalda Shokoohinia
Yalda Shokooninia
Sepideh Shokouhi
Babak Shokrani
Oluwafolahan Sholeye
Lynette Sholl
Sergey Sholom
Sabrina Sholts
Addmore Shonhai
Natalie Shook
Steven Shoptaw
Roman Shor
Anna Shore
Jennifer Shore
Boris Shorning
Martin Short
Rebecca Short
Sarah Short
Chris Shortall
Stephen Shortell
Linda Shortliffe
Michael Shott
Dan Shou
Dauha Shou
Wilson Z. Shou
Scott Showalter
Kurt Showmaker
K. Shoyama
Max Shpak
Amjad M. Shraim
Sindu Shree
Ashish Shrestha
Bharat Shrestha
Dev Shrestha
Gentle Shrestha
Govinda Shrestha
Mahendra Shrestha
Mani Shrestha
Nirman Shrestha
Rajendra Shrestha
Ram Shrestha
Roman Shrestha
Deeb Shrestha Dangol
Manju Shri
Susan Shriner
Shubham Shrivastava
Sushant Shrivastava
Monal Shroff
Zubin Shroff
Joshua Shrout
Blerta Shtylla
Bangrong Shu
Chin Shu
Gang Shu
Panpan Shu
Sheng Shu
Xiaohong Shu
Xiaokang Shu
Xu Shu
Xin Shuai
Brian Shuch
Hao-Ai Shui
Devesh Shukla
Girish Shukla

Ratnakar Shukla
Sanjeev Shukla
Shruti Shukla
Sourabh Shukla
Sudhanshu Shukla
Surendra Shukla
Tiberiu Shulimzon
Abraham Shulman
Julia Shulman
Lee Shulman
Boaz Shulruf
Howard Shuman
Sara Shumway
Robert Shura
Michael Shurin
Stephen Shuster
David Shveiky
Michael Shwartz
Stephanie Shwiff
Michael Shy
Yu Shyr
Kou-Gi Shyu
Guangwei Si
Hongwei Si
Huaijun Si
Lei Si
Yali Si
Elaine Sia
Seyed Siadat
Ahmed Siah
Cephas Sialubanje
Popluechai Siam
Gina Siapco
Miroslawa Siatecka
Farideh Siavoshi
R. Gary Sibbald
Kibrom Sibhatu
Oriol Sibila
Nicholas Sibinga
Carol Sibley
Shalamar Sibley
Ody Sibon
Luigia Sica
Pierre Sicard
Laura Sichero
Gabriele Siciliano
Jason Sicklick
Cosmin Sicora
Jaime E. Sidani
Benazir Siddeek
Aaron Sidder
Busi Siddhardha
Sridhar Siddharth
Rafat Siddiq
Imran Siddiqi
Hifzur Siddique
Javed Siddique
Zahra Siddique
Imtiaz Siddiqui
Jawed Siddiqui
M. Siddiqui
Maheen Siddiqui
Zarrin Siddiqui
Peter Sidebotham
Lucia Sideli
Matthew Samuel Amuel
Siderhurst
Venkataramana Sidhaye
Pritam Sidhu
Hatta Sidi
Kyriaki Sidiropoulou
Emil Sidky
Christian Sidor
Marina L. Sidorenko
Grigori Sidorov
Arend Sidow
P. Sie
Andrea Siebenhofer-Kroitzsch
Alrun Siebenkäs
Fritz Sieber
Volker Sieber
Tobias Siebert
Ursula Siebert
Derek Sieburth
Cynthia Sieck
Gary Sieck
Jochen Sieck
Karen Siedecki

Lynn Siefferman
Krista J. Siefried
Jeffrey Siegel
Jennifer Siegel
Jerome Siegel
Jonathan Siegel
Karolynn Siegel
Martin Siegel
Paul Siegel
Rebecca Siegel
Deborah Siegele
Bob Siegerink
Courtney Siegert
Olaf Siegert
Sandra Siegert
Ilene Siegler
Lee Siegmund
Thomas Siegmund
Claire-Anne Siegrist
Emilio Siendones
Amanda Sierra
Gerardo Sierra
Juan Carlos Sierra
Constantinos Siettos
Beau Sievers
Karl-Dietrich Sievert
Ricardo Siewert
Roland Siezen
Richard Sifers
Geofrey Sigalla
Mr Sigdel
Erwin Sigel
Trevor Siggers
Robert Sigl
Michael Sigler
Eric Sigmund
Erik Sigmund
Isabel Signes
Margaret Signorella
Salvatore Signorelli
Giuseppe Signoriello
Simona Signoriello
Martin Sigurdsson
Ólafur Sigurjónsson
Erika Sigvardsdotter
Per Sigvardsen
Susan Sigward
Fred Sigworth
Tiffany Sih
Debjani Sihi
Xu Sihua
Aleksi Sihvonen
Paul Sijens
Derek Sikes
Mile Šikić
Predrag Sikiric
Neal Sikka
Suresh Sikka
Aleksandra Sikora
Justyna Sikora
Martin Sikora
Timothy Sikora
Sverker Sikström
Parames Sil
Radu Silaghi-Dumitrescu
Asli Silahtaroglu
Emerson Silami
Emerson Silami-Garcia
Giorgia Silani
Philippe Silar
Lawrence Silbart
Alan Silberberg
Alon Silberbush
Dafne Magali Silberman
Yuval Silberman
Rebecca Silbermann
Janet Silbernagel
Claudia Silberstein
J. Silberstein
Noah Silbert
Nyssa Silbiger
Cameron Siler
Theresa M. Siler-Khodr
Antonietta Silini
Sonja Siljak-Yakovlev
Joan Silk
Neftalí Sillero
Erin Sills

Greg Silsbe
Ed Silson
Abigail Silva
Adriana Silva
Alexandre Silva
Antonio Silva
Catarina Silva
Cecy Silva
Claudia Lucia Silva
Claudia Silva
Claudionor Silva
Daniel Silva
Diego Silva
Emmanuel Silva
Erick Silva
Fabio Silva
João Silva
Jonathan Silva
Jorge Silva
Jose Silva
Leonardo Silva
Manuela Silva
Maria Joao Silva
Mario Silva
Matthew Silva
Neuza Silva
Paula Silva
R. Silva
Ricardo Silva
Tatiane Silva
Vanessa Silva
Veronica Silva
Vivian Silva
Washington Silva
Weliton Silva
Andreia Silva Machado
Nicholas Silvaggi
Mario Silva-Neto
Ludovica Silvani
Fernando Silveira
Henrique Silveira
Luis Silveira
Marcelo Silveira
Patrícia Silveira
Brian Silver
Jonathan Silver
Julie Silver
Roxane Silver
Samuel Silver
Donald Silverberg
Michael Silverman
Neal Silverman
Ronald Silverman
William Silverman
Candice Silversides
Roy Silverstein
Ricardo Silvestre
Gerard Silvestri
Mauro Silvestrini
Nicola Silvestris
Serino Silvia
Silvia Silvia Lai
Alexander Silvis
Juha Silvola
Vipul Silwal
Cheolho Sim
Judith Sim
Kyra Sim
Yang-Hu Sima
J. Marc Simard
Leickness Simbayi
Sara Simblett
Fekade Sime
Jožef Šimenko
Charles Simenstad
Pavla Simerská
Marija Simic
Paul Simion
Dan Simionescu
Paolo Simioni
Frank Simmen
Thomas Simmen
Blake Simmon
Michael Simmonds
Chelsey Simmons
David Simmons
Sarah Simmons
W. Kyle Simmons

William Simmons
Davina Simoes
Maria Luisa Simoes
Zilá Simões
Ana Cristina Simões e Silva
Marcos Simoes-Costa
Alexander Simon
Amma L. Simon
Arne Simon
Chris Simon
David Simon
Dominique Simon
Edina Simon
Fabrice Simon
Gregory Simon
Jonathan Simon
Luke Simon
Marcelo Simon
Michael Simon
Michaela Simon
Olivier Simon
Perikles Simon
Raphael Simon
Rüdiger Simon
Sandra Simon
Scott Simon
Stéphanie Simon
Calogera Simonaro
Michele Simonato
Eero Simoncelli
Hannah Simonds
Luiz Simone
Michael Simone-Finstrom
Javier A. Simonetti
Jane Simoni
Warner Simonides
Clémence Simonin
Andrean Simons
Andrew Simons
Brenna Simons
Nadja Simons
Jakob Simonsen
Kari Simonsen
Dean Keith Simonton
Tzahit Simon-Tuval
Karen Simonyan
Kristina Simonyan
Colin Simpfendorfer
David L. Simpong
Alastair Simpson
Amber Simpson
Beryl Simpson
Craig Simpson
David Simpson
Eleanor Simpson
Elizabeth Simpson
Jane Simpson
Jared Simpson
Judy Simpson
Melanie Simpson
Nigel Simpson
Norah Simpson
Paul Simpson
Pippa Simpson
Stephen Simpson
Carrie Sims
Emily Sims
Hatice Simsek
Suat Simsek
Sakari Simula
Edgar Simulundu
Celine Sin
David Sinacore
David Sinclair
Rod Sinclair
Ilango Sindana
John Sinden
Caroline Sindet-Pedersen
Sardar Sindhu
A. K. Singal
David Singer
Fred Singer
Greg Singer
Harold Singer
Pierre Singer
Stacy Singer
Steven Singer
Aadesh Singh

Agam Singh
Ajay Singh
Akanksha Singh
Akhilesh Singh
Aman Preet Singh
Amar Singh
Amarjeet Singh
Amaya Singh
Amit Singh
Amrita Singh
Anil Singh
Anup Singh
Arun Singh
Avtar Singh
Bashisth Narayan Singh
Bhagat Singh
Bijender Singh
Brahmanand Singh
Brajendra Singh
Davinder Singh
Devinder Kaur Ajit Singh
Devraj Singh
Dharmendra Singh
Dhirendra Singh
Garima Singh
Ghanshyam Singh
Gurmukh Singh
Hardeep Singh
Harminder Singh
Inderjit Singh
Inderpaul Singh
Jay Shankar Singh
Jerome Singh
Jugpreet Singh
Kamalesh Singh
Kashmir Singh
Kavita Singh
Kern Singh
Kiran Singh
Krishna Singh
Mahavir Singh
Manpreet Singh
Mayank Singh
Meera Singh
Mohita Singh
Nagendra Singh
Nalini Singh
Neeloo Singh
Nilendu Singh
Pawan Kumar Singh
Pawan Singh
Pomila Singh
Pooja Singh
Pushpendra Singh
R. Singh
Rajan Singh
Rakesh Singh
Ram Singh
Raman Singh
Ramesh Singh
Sachin Singh
Samiksha Singh
Sanjay Singh
Sarjit Singh
Sarman Singh
Satinder Singh
Shailender Singh
Shailesh Singh
Sheelendra Singh
Shoovir Singh
Simron Singh
Soudamani Singh
Sudhir Kumar Singh
Sudhir Singh
Sukhwinder Singh
Sunita Singh
Varsha Singh
Vijay Singh
Vikash Singh
Vinayak Singh
Vineet Singh
Vinodkumar Singh
Vir Singh
Vishal Singh
Vivek Singh
Yadvir Singh
Yogendra Singh
Yugpreet Singh

Ujjal Singha
Pravin Singhal
Vibha Singhal
Vijay Singhal
Abhishek Singharoy
Surinder Kumar Singla
Ian Singleton
Peter Singleton
Aakanksha Sinha
Alok Sinha
Amit Sinha
Arjun Sinha
Devesh Sinha
Dhiraj Sinha
Gaurav Sinha
Ian Sinha
Kalyan Sinha
Meenal Sinha
Rajiv Sinha
Rohit Sinha
Surajit Sinha
Pritam Sinharoy
Juha-Jaakko Sinikumpu
Antonio Siniscalchi
Marcello Siniscalchi
Antonio Sinisi
Christopher Sinke
Dong Hyun Sinn
Christoph Sinning
Robert Sinsabaugh
Anthony Sinskey
Herman Sintim
Albert Sinusas
Christian Sinzger
Wai Ting Siok
Chrissa Sioka
Haruhiko Siomi
Edward Sionov
J. M. Sions
Szabolcs Sipeki
Brent Sipes
Matthias Sipiczki
Kalle Sipila
Tomasz Sipko
André Siqueira
Luiz Siqueira
Marcos Siqueira
Paul Siqueira
Patricia Siques
Rossana Sirabella
Mark Siracusa
Jeffrey Siracuse
Nikolay Sirakov
Marc-André Sirard
Ravi Sirdeshmukh
Adiatma Siregar
Anna Siri
Hellen Siril
Ubonrat Siripatrawan
Sawanya Siriphakhamongkhon
Charalampos Siristatidis
Dimuth Siritunga
Arunotai Siriussawakul
Davud Sirjani
Aubrey Sirman
Caroline Sirois
Brian Siroky
Michael Sirover
Shashank Sirsi
Daniel Sirtes
Albert Siryaporn
Mitike Sisay
Gaetano Siscaro
Lacey Sischo
Meghan Sise
Sergio Sismondo
Daniella Sisniega
Susan Sisson
Erik Sistermans
Ainara Sistiaga
Regina Sit
Cassian Sitaru
Vasilis Sitras
Elske Sitsen
Gilman Siu
Ka Yu Siu
Michelle Siu
J. Siuda

Aksel Siva
Shankar Siva
Mamata Sivagnanam
Rathinam Sivakumar
Sushama Sivakumar
Ranjit Sivanandham
Kisha Sivanathan
Katia Sivieri
Harry Siviter
S. M. Siviy
Aida Sivro
Justyna Siwy
Diana Six
Titia Sixma
Stephane Sizonenko
Christina Sizun
Alexander Sizykh
Anders Sjalander
Matthias Sjerps
Gottfrid Sjödahl
Virginie Sjoelund
Erik Sjögren
Karl-Göran Sjögren
Anita Sjölander
Marie Sjolund
Anders Sjöstedt
Todd Skaar
Sergii Skakun
Melissa Skala
Pavel Škaloud
Rebecca Skalsky
Helen Skaltsa
Marion Skalweit
Stephen Skaper
Petros Skapinakis
Aneta Skaradzinska
Christina Skarpe
Dariusz Skarzynski
Murray Skeaff
Patrick Skelly
Peter Skelsey
Dawn Skelton
Arne Skerra
Timothy Skerry
Lori Skibbe
Randal Skidgel
Andre Skidmore
Nathan Skillen
Greg Skilleter
Asheley Skinner
Daniel Skinner
Eila Skinner
Frances Skinner
Jonathan Skinner
Matthew Skinner
Michael Skinner
Timothy Skinner
Lisa Skinner (Louth)
Ingiridur Skirnisdottir
Kathryn Skivington
Lars Skjærven
Rolv Skjarven
Finn Egil Skjeldestad
Carsten Skjoth
Ella Sklan
Larry Sklar
Mikhail Skliar
Varadhan Skm
Szymon Skoczynski
Pontus Skoglund
Lesli Skolarus
Anders Sköldunger
Adrianna Skoneczna
Emmanuel Skordalakes
Sabrina Skorski
Elżbieta Skorupska
Matej Skorvanek
Brian G. Skotko
A. N. Skouloudis
Rachid Skouta
Jakob Skov
Peter Skov
Søren Skov
Pernille Skovby
Knut Skovereng
Dorota Skowyra
Riste Skrekovski
Karen Skriver

Jerzy Skrzyszewski
Daniel Skupski
Thomas Skurk
M. Skutsch
Krzysztof Skuza
Jerod Skyberg
Mark Slabaugh
Hans Slabbekoorn
Catherine Slack
Emma Slack
Jonathan Slack
Mary Slack
Todd Slack
Linda Slack-Smith
Susan Slade
Tim Slade
Ronald Sladky
Heleen Slagter
Douglas Slain
Nada Slakeski
Clare Slaney
Robert Slany
Jan Šlapeta
Dennis Slate
Luke Slater
Margaret Slater
Mel Slater
James Slaughter
Laurel Slaughter
Virginia Slaughter
Roderick Slavcev
Svetoslav Slavov
William Slayton
Emma Sleath
Pawel Sleczka
Jamie Sleigh
Zdenka Slejkovec
Sergey Slepchenko
Dennis Slice
Ilja Sligte
Mahmoud Slim
Rima Slim
Ludovic Slimak
Maamer Slimani
Maja Slingerland
Bernard Slippers
Jadwiga Śliwka
Frank Sloan
Luke Sloan
Amy Slogrove
Katherine Sloman
Lauren Slone
Donna Slonim
Lizeth Sloot
Jens Sloth
Lukas Slovak
Hamutal Slovin
Nikolai Sluchanko
Judith Sluimer
James Sluka
Pablo Slullitel
Cornelis H. Slump
Tina Slusher
David Slusky
Laurence Slutsker
Ronald Sluyter
William Sly
Jennifer Slyker
Fatima Smagulova
David Šmajs
Paul Smaldino
Brian Small
Christopher Small
Gaston Small
Mitchell Small
Tesh Smalle
Isabel Smallegange
Ian James Smalley
Jonathan Smallwood
Jonathan Smart
Lawrence Smart
Leonard Smart
Michael J. Smart
Neil Smart
Simon Smart
Cynthia Smas
Sarra Smati
Linnéa Smeds

Donald Smee
Bart Smeets
Jeroen Smeets
T. Smeets
Sonia Smeraldo
Piera Smeriglio
Anna Smetanová
Richard Smeyne
Maria Śmiałowska
Joost Smid
Marcela Smid
Jiří Šmíd
Jaroslaw Smieja
Joe Smilanick
Age Smilde
James Smiley
Jeff Smiley
Tara M. Smiley
Pablo Smircich
Daniela Smirni
Alexandra Smirnova
Ekaterina Smirnova
Michelle Smirnova
Natalia Smirnova
Vytautas Smirnovas
Berend Smit
Egbert Smit
John Smit
Joost Smit
Suzanne Smit
Theo Smit
A. Peyton Smith
Adam Smith
Adrian Smith
Albert Smith
Alexia Smith
Alexis Smith
Amy Smith
Andra Smith
Andrew Smith
Andrew G. Smith
Ann Smith
Beth Smith
Brennan Smith
Bruce Smith
Bryan Smith
C. M. Smith
Carine Smith
Carol Smith
Charles Smith
Colin Smith
Corey Smith
Craig Smith
Darci Smith
David Smith
Derek Smith
Diarmuid Smith
Donald Smith
Elizabeth Smith
Emily Smith
Eric Smith
Frank Smith
Frank W. Smith
Gary Smith
George Smith
Gerald Smith
Gordon Smith
Heather Smith
Helen Smith
J. Carson Smith
James Smith
Jason Smith
Jennifer Smith
Jeramiah Smith
Jeremy Smith
Joe Smith
John Smith
Jonathan Smith
Joseph Smith
Joyce Smith
Julia Smith
Justin Smith
Kevin Smith
Kristina Smith
Kumi Smith
Lee Smith
Lewis Smith
M. Smith

Michael Smith
Monique Smith
Nick Smith
Noelle Smith
Phillip Smith
Rebecca Smith
Richard Smith
Rob Smith
Robert Smith
Russell Smith
Shamus Smith
Stephanie Smith
Stephen B. Smith
Stephen Smith
Steve Smith
Steven Smith
Stuart Smith
Susan Smith
Thomas Smith
Tim Smith
Timothy Smith
Todd Smith
Tracey Smith
Trenton Smith
W. Smith
William Smith
Laura Smith Callahan
Sarah Smith Lundsford
Lindsey Smith Taillie
Thomas Smithgall
Karen Smith-McCune
Kenneth Smithson
Erica Smithwick
Zeljka Smit-McBride
Anke Smits
Frans Smits
Peter Smits
Sander Smits
Veronique Smits
Bouwien Smits-Engelsman
Joseph Smoak
Joost Smolders
Albert Smolenski
Agnieszka Smolinska
Adam Smolka
Eva Smolka
Betty Smoot
David Smotkin
Peter Smouse
Sophie Smout
Tom Smulders
Petr Smýkal
Bobby Smyth
Davida Smyth
Winona Snapp-Childs
Charles Snart
Margaret Snead
Christopher Sneddon
Rodlescia Sneed
Kirstine Sneider
Cameron Snell
Warren Snelling
Patrick Snellings
Claire Snell-Rood
Kim Sneppen
Eveleen Sng
Nathan Sniadecki
Martin Snickars
Matt Snider
Natasha Snider
Soham Snih
Roland A. Snijder
Antoine Snijders
Tom Snijders
Eelke Snoeren
Aisling Snow
Andrew Snow
Catherine Snow
Jessica Snowden
Jonathan Snowden
Allan Snyder
Christopher Snyder
Hannah R. Snyder
Hannah Snyder
Jason Snyder
Kenneth Snyder
Kristine Snyder
Michael Snyder

Peter Snyder
Carl Snyderman
Po-Wah So
Jeffrey Soar
Ana Luiza Soares
Claudio Soares
Helena Soares
Joao Soares
Marcelo Soares
Marianne Soares
Michael Soares
Milena Soares
Sylvia Soares
Tatiana Soares da Costa
Rafael Soares Lindoso
Abilio Soares-Gomes
Luisa Soares-Miranda
Samuel Sober
Patrick Sobetzko
Christopher Sobey
Iradj Sobhani
Przemysław Sobiech
Pawel Sobkowicz
Gabriela Sobral
Paula Sobral
Marcone Sobreira
Lucia Sobrin
Francisco Sobrino
Mohammad Sobuh
John Socha
Ryan Sochol
Sanjeev Sockalingam
S. Jacob Socolar
Tim Soderlund
Maria Söderlund-Venermo
Komal Sodhi
Manmohan S. Sodhi
Andrea Sodi
Devin Sodums
Radina Soebiyanto
Signe Soegaard
Kenzo Soejima
Surjo Soekadar
Takeshi Soeki
Pilar Soengas
Isabelle Soerjomataram
Yoshiharu Soeta
Brian Soetikno
Gerald Soff
Mohd Sofian-Azirun
Seidu Sofo
Tadashi Sofue
Susan Sogard
Enitan Sogbesan
Jean-Jacques Soghomonian
May Soh
Sze-Ee Soh
Md. Mahmodul Hasan Sohel
Terry Sohl
Ebba Sohlberg
Yoshiro Sohma
Haim Sohmer
Annette Sohn
Chul-Ho Sohn
Hongchul Sohn
Kitae Sohn
Moon-Jun Sohn
Seil Sohn
Yu-Mee Sohn
Jae-Kyung Sohng
Brent Sohngen
Kazuhiro Sohya
Robert Soiffer
Harry Sokol
Joshua Sokoloski
Kevin Sokoloski
Arseny Sokolov
Małgorzata Sokołowska-Wojdyło
Katie Sokolowski
Marcus Sokolowski
Marla Sokolowski
Marina Sokovic
Elke Sokoya
Evgeni Sokurenko
Carretero Sol
Anna Sola
Elsa Sola

Jordi Sola
Ivan Solà
Zakaria Solaiman
Jitender Solanki
Francisco Solano
Laia Solano-Gallego
Arturo Solares
Fiorella Solari
Giulia Solda
Anja Soldan
Samantha Soldan
Anastasiia Soldatenkova
Larisa Soldatova
Albert Sole
Gisela Sole
Floréal Solé
M. Soleimani
Jehuda Soleman
Juan Soler
Manuel Soler
Maria Jose Soler
María José Soler
Xavier Soler
Javier Solera
Anne Lee Solevåg
Samaneh Solhjouy-Fard
Cosimo Solidoro
Karam Soliman
Marc Solioz
M. Solis
Jose Solis-Lemus
Rodolfo Solís-Vivanco
David Soll
Lynn Sollenberger
Andrea Solnes Miltenburg
Joseph Solomkin
Barry Solomon
Daniel Solomon
George Marty Solomon
George Solomon
Jeffrey Solomon
Justin Solomon
Richard Solomon
Thomas Solomon
Tessa Solomon-Lane
Alexandre Solon
Thamar Solorio
Carmen Solorzano
Nikos Solounias
Caius Solovan
Maxim Solovchuk
Irina Solovei
Mikhail Soloviev
Madjid Soltani
Soheil Soltani
Cinna Soltanpur
Florian Sölter
Arkadiusz Soltysiak
Una Solvik
Sudipta Som
Kiran Soma
Venugopal Rao Soma
Yuki Soma
Stephen C. Somach
Arun Somani
Agila Somasundaram
Rajesh Somasundaram
George Somero
Shawn Somerset
Yuki Someya
Kritapon Sommart
Alfred Sommer
Anders Sommer
Christine Sommer
Christoph Sommer
Gerhard Sommer
Gunhild Sommer
J. Ulrich Sommer
Marni Sommer
Ted Sommer
Olaf Sommerburg
Christopher Sommers
Carolin Sommer-Trembo
Endre Somogyi
Gustavo Somoza
Prakit Somta
Francis Somville
Hokyoung Son

Lisa Son
Seung Hyun Son
Youngdoo Son
Humira Sonah
Avinash Sonawane
Ganesh G. Sonawane
Yogesh Sonawane
Rakesh Sondekoppam
Mark Sonderup
Daichi Sone
Hideko Sone
Hirohito Sone
Toshimasa Sone
Samir Soneji
Abraham Sonenshein
Jenny L. Sones
Alex Song
Bin Song
Bo Song
Brian Song
Chang Song
Chang-Hwa Song
Chang-Xu Song
Chunyu Song
Conghe Song
Cunjiang Song
Dandan Song
Eunju Song
Fangchao Song
Fengju Song
Fengming Song
Gaoyuan Song
Guang Song
Guili Song
H. S. Song
Haegeun Song
Haengseok Song
Heng Song
Ho June Song
Hojun Song
Hongbin Song
Hongjun Song
Hongsheng Song
Houbing Song
Huan Song
Hyun Kyu Song
Hyun-Joo Song
J. Song
Jae-Jin Song
Jeongmin Song
Jeonqmin Song
Jian Song
Jiangdian Song
Jiangning Song
Jiasheng Song
Jie Song
Jikui Song
Jingjing Song
Jingyuan Song
Jinxi Song
Jiuzhou Song
Juan Song
Kai Song
Kunhua Song
Kyuyoung Song
Lei Song
Lele Song
Li Song
Li-Peng Song
Long-Sheng Song
Malin Song
Mina Song
Minghua Song
Mingyang Song
Moshi Song
Nan Song
Qiankun Song
Qijian Song
Qing Song
Qisheng Song
Rong Song
Seongho Song
Shaojuan Song
Shiya Song
Shujie Song
Si Yeol Song
Sihong Song
Sijie Song

Tao Song
Wei Song
Weiguo Song
Wenxia Song
Xiang Song
Xianliang Song
Xiao Ping Song
Xiaofeng Song
Xicheng Song
Xue Song
Yajun Song
Yaling Song
Yan Song
Yiying Song
Yong Song
Yongjia Song
Yu Song
Yuanlin Song
Yufang Song
Yun Seon Song
Zhaojian Song
Zhenyuan Song
Zigen Song
Maryam Songhorzadeh
Marco Songini
Apurv Soni
Neetu Soni
Rajan Sonik
Steve Sonis
Fatma Sonmez
Geoffrey Sonn
Ranil Sonnadara
Gabriella Sonnante
Oliver Sonnentag
Junichiro Sonoda
Noriyuki Sonoda
Fuminori Sonohara
Kintake Sonoike
Masahiro Sonoo
Tad Stewart Sonstegard
Sadanand Sontakke
Laura Sonter
Po-Chi Soo
Akshay Sood
Ashwani Sood
Gaurav Sood
Rashmi Sood
Ravi Sood
Richa Sood
Salej Sood
Sajid Soofi
Kathleen Soole
Keryea Soong
Wen-Jue Soong
Boonchoy Soontornworajit
Hamid Soori
Rajini Sooryanarayana
Miroslav Soos
Dimitrios S. Sophianopoulos
Beltran-Bech Sophie
Jörg Soppa
Massimiliano Sorbello
Mary Sorci-Thomas
Karim Sorefan
Robert Soreng
Jesper Sorensen
Marjorie Sorensen
Rikke Sørensen
Uffe Sørensen
Hermona Soreq
Joseph Sorg
Paul Sorgen
Jeannette Soria
Victor Soria-Aledo
Francesc Soriano
Francisco Soriano
Marlene Soriano
Sergi Soriano
Emanuele Soricelli
Hiroyuki Sorimachi
Didier Sornette
Liliana Soroceanu
Victoria Soroker
Maria Sorokina
Maria Soroko
Iryna Sorokulova
Francesco Sorrentino
Rosa Sorrentino

Rosalinda Sorrentino
Sabato Sorrentino
Marina Sorrentino Hernandes
Muluken Sorrie
Bruno Antonio Sorrija
Cascade Sorte
Maria Angela Sortino
Jouni Sorvari
Aaron Sosa
Victoria Sosa
Roberto Sosa-López
Sindia Sosdian
Natalia Soshnikova
Heidi Sosik
Jonathan Soslow
Gabriel Sosne
Philippe Sosner
Jacob Sosnoff
Tomasz Sosnowski
Ana Sotelo
Constantino Sotelo
José Sotelo
Julio Sotelo
Rogerio Sotelo-Mundo
Andreas Diomedes Soteriades
Ioanis Sotiroppoulos
Antonia Soto
Braulio Soto
Daniel Soto
Esteban Soto
Karina Soto
Martha Soto
Sara Soto
Sergio Soto-Acuña
Claudio Soto-Azat
Diego Sotomayor
Abdolreza Sotoodeh Jahromi
Cristina Sottani
Matthew Sottile
Albert Sotto
Carlos Soubervielle-Montalvo
Guillaume Souchay
Jean-Paul Soucy
Hugo Soudeyns
Seynabou Sougoufara
Renata Sõukand
Laura Soul
Hedi Soula
Christophe Soulage
Tanya Soule
Maria Souli
Saha Soumendra
Nagasamy Soumittra
Pierre Jean Souquet
Pierre Sourdille
Harald Sourij
Jeffrey Souris
Alejandro Sousa
Daniela Sousa
Lia Sousa
Magda Sousa
Marlos Sousa
Tais Sousa
Valeria Sousa
Vanessa Sousa Moreno
Andrew South
Mikle South
Christopher Southan
Robert Southard
Brian Southern
Paul Southgate
Amber Southwell
Brian Southwell
Bridget Southwell
David Souto
Miguel Souto
Olga Soutourina
Alexandre Navarro Alves Souza
Erica Souza
Givago Souza
Max Souza
Raquel Souza
Richard Demo Souza
Roshan Souza
Thársis Souza
Reinaldo Souza-Santos
Flavia Souza-Smith
Erin Sovansky Winter

Tamás Sovány
Anna Soveri
Amina Sow
Marianne Sowa
Wojciech Sowa
Magdalena Sowa-Kucma
Richard Sowell
James Sowers
Pawel Sowinski
Joseph Sowka
Paul Sowman
Hideaki Soya
H. Peter Soyer
Oluwole Soyinka
Ireneous Soyiri
Efe Soyman
Giulia Sozio
Ramón Spaaij
Erica Spackman
Teresa Spadea
Julia Spaet
George Spaeth
Johannes Spaethe
Simone Spagnol
Giulio Spagnoli
Sean T. Spagnoli
Anna Spagnolli
Matteo Spagnolo
Paolo Spagnolo
David Spain
Debbie Spain
Seth M. Spain
Marta Špakulová
Heather Spalding
Katharina Spalek
Stjepan Spalj
Jacob Spallek
Espen Spangenburg
Giuseppe Spano
Herbert Spapen
Laura Sparaci
Giovanni Sparacino
Joseph Sparano
Katrin Sparbier
Tim Sparer
Henrik Sparholt
Joy Spark
Erica Sparkenbaugh
Adam Sparks
Caroline Sparks
Corey Sparks
Jackson Sparks
Tim Sparks
Marco Sparro
John E. Sparrow
Dennis Sparta
Nicole Spartano
Francesca Sparvoli
Philipp Späth
Michelle Spaulding
Norah Spears
Russel Spears
Silvia Speca
Alessandro Specchia
Stephen Spector
Ben Speers-Roesch
Jeffrey Spees
Sean Speese
Walter Speidl
Douglas Speirs
Ilene Speizer
Chester Spell
Camilla Speller
Charles Spence
Dana Spence
J. David Spence
John Spencer
Joseph Spencer
Karen Spencer
Robert Spencer
Ryan Spencer
Robert Spengler
Felipe Sperandio
Olivier Sperandio
Holger Franz Sperdin
Alessandra Sperduti
Miriam Spering
Billy Sperlich

Darcy Sperlich
Barbara Sperner-Unterweger
Jason Sperry
Robert Speth
Maartje Spetter
Anna-Lea Spetz
Karina Speziale
Corinne Spickett
David Spiegel
Jerry Spiegel
Martin Spiegel
Kai Spiegelhalder
Thomas Spiegler
Tobias Spielmann
Sharon Spier
Claudia Spies
Georgina Spies
John Spiesberger
Andrej-Nikolai Spiess
Patrizia Spigaglia
Sabine Spijker
Konstantinos Spiliopoulos
Marios Spiliotopoulos
Jiří Spilka
Brad Spiller
David Spiller
Robin C. Spiller
Josh Spin
Michele Spina
Andrea Spinazzè
Pascal Spincemaille
Dominik Spinczyk
Volker Spindler
Loukia Spineli
Antonello Spinelli
Francesca Romana Spinelli
L. Spinelli
Sara Spinelli
Sherry Spinelli
Susanna Spinsante
Michael Spiotto
Beny Spira
Micha Spira
Tara Spires-Jones
Anthony Spirito
Carlo Spirlì
Christina Spiropoulou
Hergen Spits
Manuel Spitschan
Rachel Spitzer
Mary Spitznagel
Adam Spivak
Marla Spivak
G. Splitter
Iris Spoerri
Ana Maria Spohr
Kurt Spokas
Antanas Spokevicius
Goran Spong
Jeff Spooner
Michael Sporn
Peter Sporns
Bruno Sposato
Richard Sposto
Silvia Spoto
George Spracklin
Frank Spradley
M. Katherine Spradley
Matthew Sprague
Stuart Sprague
Ben Sprangers
Remco Sprangers
Daniel Spratt
Heidi Spratt
Neil Spratt
David Spray
Armand Sprecher
Simon Sprecher
Nikica Sprem
R. Nathan Spreng
Ole Sprengeler
Andreas Sprenger
Lawrence Spriet
Stephen Sprigle
Boris Springborn
Henriet Springelkamp
Mark Springer
Sandra Springer

Richard Sproat
Julann Spromberg
Daisy Sproviero
William Sproviero
Martin Spüler
Anne Spurkland
Robert Spurney
Ioakim Spyridopoulos
Letizia Squarcina
Flaminio Squazzoni
Nicola Squillace
Geoffrey Squire
Iain Squire
Stefania Squizzato
Martin Sramek
Marek Sramko
Benjamin Sredni
Lakshmi Sreedharan Nair
G. B. Sreekanth
S. Sreelatha
Sheshshayee Sreeman
Chandrashekhar
Sreeramareddy
Angie Sremba
Ashwin Sridhar
Jayanth Sridhar
Kandikere Sridhar
Sanjeev Sridharan
Keerthi Srinivas
Sangly Srinivas
Aravind Srinivasan
C. S. Srinivasan
Krishnapura Srinivasan
Mandyam Srinivasan
Narayanan Srinivasan
P. Srinivasan
Prakash Srinivasan
Rahul Srinivasan
Rajagopalbabu Srinivasan
Ramaprasad Srinivasan
Ramprakash Srinivasan
Sathish Srinivasan
Shanthi Srinivasan
Sujatha Srinivasan
Supriya Srinivasan
Uma Srinivasan
Venkateswara Sripathi
Shiranee Sriskandan
Hathaitip Sritanaudomchai
Akhil Srivastav
Anubhav Srivastava
Bhupendra Srivastava
Neha Srivastava
Raghvendra M Srivastava
Raghvendra Srivastava
Rajesh Srivastava
Rajeshwar Srivastava
Ruchi Srivastava
Shireesh Srivastava
Shraddha Srivastava
Sweta Srivastava
Anjana Srivatsan
Edward Srour
Nadim Srour
Victor Ssempijja
Willy Ssengooba
Samuel St. Clair
Cynthia St Hilaire
Alexander St. John
Benoit St. Pierre
Arie Staal
Henk Staats
Sarah Stabenfeldt
Mikkel Staberg
Michael Stabin
Fiona G. Stacey
William Stacey
Thomas Stach
Shawn Stachel
Nina Stachenfeld
John Stachowicz
Katarzyna Stachura-Suchoples
Rebecca Stack
Steven Stack
Steven Stacker
Lucy Stackpool-Moore
Shaina Stacy
Tomasz Stadejek

Daniel Stadlbauer
Andreas Stadler
Krisztian Stadler
Zsofia Stadler
Martin Staege
Mary Staehle
Willem Staels
Katharina Staerk
Nina Stærke
James Stafford
Mai Stafford
Walter Stafford
Massimo Stafoggia
Panagiotis Stafylas
Maria Stager
Sheila V. Stager
Rita Stagni
Mirjam Stahl
Rolf Stahl
František Št'Áhlavský
Henning Stahlberg
Karen Stahlheber
Richard Stahlhut
Zachary Stahlschmidt
Jona T. Stahmeyer
Amanda Staiano
Harald Staiger
Joseph Stains
Bashar Staitieh
Kenneth Stalder
Christopher Staley
Michael Stallcup
Sericea Stallings-Smith
Hanneke Stam
Efstathios Stamatatos
Iva Stamatova
Laura Stambaugh
Davor Stamenovic
Dimitrije Stamenovic
James Stamey
Jessica L. Heier Stamm
Daniela Stan Raicu
Elena Stanca
Sonja Stander
Ansley Stanfill
Craig Stanford
Kim Stanford
Kristin Stanford
Madlen Stange
Biljana Stangeland
Gabriele Stangl
James Stangoulis
Jessica Stanhope
Phillip Staniczenko
Monika Staniszewska
Lazar Stankov
Konstantina Stankovic
Tomislav Stankovski
Dragana Stanley
Kenneth Stanley
Pamela Stanley
Ronald Stanley
Nicola Stanley-Wall
Miron Stano
Pasquale Stano
Sanja Stanojevic
Olivera Stanojlović
Christina Stantis
Bruce Stanton
Daniel E. Stanton
Jessica Stanton
Peter Stanton
Sasha Stanton
Tasha Stanton
Anne Staples
Karl Staples
Heather Stapleton
Jack Stapleton
Michael Starek
Noemy Starita
Konstantin Stark
Renee Stark
Robert Starke
Brooke Starkoff
Angela Starkweather
Athena Starlard-Davenport
Melissa Starling
Joseph Starobin

Ariel Starr
Alexander Staruschenko
Creed Stary
Philip Stashenko
Ruth Stassart
Frank Stassen
Demetris Stathakis
Meir Statman
Meg Staton
Sarah Statt
Eike Staub
Kaspar Staub
Christine Stauber
Leah Stauber
Rudolf Stauber
Tobias Stauber
Jay Stauffer
Soren Staugaard
Jürgen Stausberg
Harald Stauss
Joel Stavans
Rhían Stavely
Doekele Stavenga
Chrysanthi Staveri
Stavros Stavrakis
Anneli Stavreus-Evers
Menelaos Stavrinides
Andreas Stavropoulos
Antonis Stavropoulos
Katherine Stavropoulos
Vasilis Stavropoulos
Philipp Stawowy
Dimitre Staykov
Margarita Staykova
Mike Stear
Stephen (Steve) Stearns
Deborah Stearns-Kurosawa
Julie Stebbins
Robert Stebbins
Boguslaw Stec
David Stec
Barbara Stecca
Alessandro Stecco
Susan Steck
Todd Steck
Anke Steckelberg
Ulrike Steckelings
Jonathon Stecyk
Maggie Stedman-Smith
Douglas Steeber
Rick Steeds
Thomas Steeger
Régine Steegers-Theunissen
David Steel
Brecht Steelant
Colin Steele
Olivia Steele-Mortimer
Harald Steen
Jessica Steen
R. Grant Steen
James Steenberg
Berit Steenbock
Maria Steenhof
Tammo Steenhuis
Kyle Steenland
Bart Steensma
Colin Steensma
Matt Steensma
Richard Steet
Jeff Steevens
Norbert Stefan
Simona Stefan
Renae Stefanetti
Alessandro Stefani
Ambra Stefani
Ilaria Stefani
Giulio Stefanini
Bruno Stefanon
Spiro Stefanou
Andrej Stefanov
Detelin Stefanov
Darko Stefanovski
Justyna Stefanowicz-Hajduk
Ewa Stefańska-Krzaczek
Bergur Stefansson
Clara Stefen
Ingolf Steffan-Dewenter
Annika Steffen

Armin Steffen
Daniel Steffens
Niklas Steffens
Scott Steffensen
Michele A. Steffey
Elizabeth Stegemoller
Gerhard Steger
Karen Stegers-Jager
David Stegner
Peter Stehle
Samuel Stehle
Bertine Stehouwer
Nadia Steiber
Jamie Steichen
Rebecca Steidl
Günter Steidle
Axel Steiger
Hans Steiger
Katrina Steiling
Ariel Stein
Daniel Stein
Eric Stein
Jeffrey Stein
Nils Stein
Phyllis Stein
Wolfgang Stein
Jody Steinauer
Nick Steinauer
William Steinbach
Katherine Steinbeck
Daniel Steinberg
Fabian Steinberg
Nili Steinberg
Michael Steinborn
Harry Steinbusch
Daniel Steinemann
Laurie Steiner
Michael Steiner
Robert Steiner
Ruth Steiner
Ulrich Steiner
Daniel Steinfort
Steinn Steingrimsson
Laura Steinhardt
David Steinhauer
Martin Oliver Steinhauser
Tobias Steinhoff
Ulrich Steinhoff
John Steinke
Amir Steinman
Bernard A. Steinman
David Steinman
Eike Steinmann
Thomas Steinmann
Andreas Steinmayr
Anke Steinmetz
Holger Steinmetz
Ivo Steinmetz
Oliver Steinmetz
Paschalis Steiropoulos
Joanne Stekler
Lukasz Stelinski
Salvatore Stella
Jennifer Stellar
Giovanni Stellato
Talia Stelling-Wood
Josiane Steluti
Elsebeth Stenager
Johan Stender
Carolina Stenfeldt
Stefan Stenfelt
Michael Stenger
Sari Stenholm
Ulf-Hakan Stenman
Lauri Stenroth
Marie Stenseke
Marcus Stensmyr
Kerstin Stenson
Ulrike Stentzel
Jan Stenvang
Peter Stenvinkel
Stefanie Stenzel
Karin Stenzelius
Rebecca Steorts
Alain Stepanian
Victoria Stepanova
Zeljko Lj Stepanovic
Ruben Stepanyan

Holger Stephan
Schnorr Stephanie
Olof Stephansson
Arnott Stephen
Ruth Stephen
David Stephens
Edward Stephens
Jaclyn Stephens
Jacqueline (Jackie) Stephens
Moira Stephens
Scott Stephens
Skye Stephens
Torrance Stephens
Fabrice Stephenson
Jessica Stephenson
Alisa Stephens-Shields
Emmanuel Stephen-Victor
Carol A. Stepien
Łukasz Stępień
Susan Stepney
Cara Stepp
Katja Sterflinger
Kathleen Sterling
Lesley Sterling
Michele Sterling
Chadly Stern
Hal Stern
Joshua Stern
Michael Stern
Dagmar Sternad
Eleanore Sternberg
Rolf Sternglanz
Harvey Sterns
Richard Sterns
Gaetana Sterrantino
Torsten Sterzenbach
George Stetten
Mark Stettner
Dominik Steubl
Andrea Steuer
Simone Stevanin
Aleksandar Stevanovic
Dejan Stevanovic
Tatjana Stevanovic
Alison Steven
Steven E. Sesnie
Aaron Stevens
Andrew Stevens
Christopher Stevens
D. R. Stevens
Jamie R. Stevens
Jan Stevens
Mark Stevens
Matthew Stevens
Robert Stevens
Stanley Stevens
Vanessa Stevens
Jennifer Stevens Aubrey
Jennifer Stevens-Lapsley
Ben Stevenson
Bradley Stevenson
Dennis Stevenson
E. Stevenson
Jessica K. Stevenson
Mark Stevenson
Nathan Stevenson
Philip Stevenson
Scott Stevenson
Pablo Roberto Stevenson Diaz
Ben Steventon
Brian Steward
Trevor Steward
Alastair Stewart
Alexandre Stewart
Bryan Stewart
Christopher Stewart
Douglas Stewart
Elizabeth Stewart
Frances Stewart
George Stewart
Ian Stewart
Jane Stewart
Jesse Stewart
Laura Stewart
Mathew Stewart
Orion Stewart
Ralph Stewart
Robert Stewart

Rodney Stewart
Samuel Stewart
Sarah Stewart
Sherri Stewart
Susan Stewart
Tricia Stewart
Anna Stewart Ibarra
Jennifer Stewart Williams
Arnaud Steyaert
Ewout Steyerberg
Nelia Steyn
Steven Stice
Felix Stickel
Abigail Stickford
Hadas Stiebel-Kalish
Oliver Stiedl
James Stieger
Stefan Stieger
John Stieglitz
Jonathan Stieglitz
Jens Stieler
Charles Stier
Alessandro Stievano
Marc Stift
Florian Stigler
Sara Stigliani
David Stillman
Isaac Stillman
Jonathon Stillman
Eileen Stillwaggon
David Stillwell
Serge Stinckwich
Luca Stingeni
Krunoslav Stingl
Catalina-Suzana Stingu
Sandra Stinnett
Alin Stirban
Tom Stiris
David Stirling
Oliver Stirrup
Etienne St-Jean
Anthony Stobbe
Ellen Stobberingh
Matteo Stocchero
Andrea Stocco
Sarah Stock
James David Stockand
Randy Stockbridge
Heather Stockdale Walden
Fabian Stöcker
Philipp Stockhammer
Heidi Stöckl
Creina Stockley
Jürg Stöcklin
Hugh Stoddard
Phillip Stoddard
Joachim Stoeber
Barbara Stoecker
Johannes Stoeckl
Mark Stoeckle
Angela Stoeger
Tobias Stoeger
Michael Stoehr
Viola Stoermer
Alexander Stoessel
Martin Stoffel
Thomas Stoffregen
Reinhard Stöger
John Stogner
Jure Stojan
Jadranka Stojanovski
Christopher Stojanowski
Monika Stojek
Maja Stojiljkovic
Clare Stokes
David Stokes
Lynissa R. Stokes
Philip Stokes
Maria Stokstad
Maciej Stolarski
Beatriz Stolf
Jan Stolk
Alexandra Stoll
Gautier Stoll
Eckart Stolle
Jason Stoller
Carmen Stolwijk
Andrew Stolz

Bernadette Stolz
Uwe Stolz
Rachael Stolzenberg-Solomon
Elijah Stommel
Martin Stommel
Tjeerdjan Stomph
Samantha Stonbraker
Alexandra Stone
Brandee Stone
Gregory Stone
Joseph Stone
Lisanne Stone
Pamela Stone
Wendy Stone
William Stone
Angela Stone-MacDonald
Julie Stoner
Kimberly Stoner
Barbara Stonestreet
William Stoops
Arkadiusz Stopczynski
Christian Stoppe
Kai-Florian Storch
Helena Štorchová
Ole Jakob Storebø
Daniela Storino
Stefan Störk
Jonathan Storkey
Walter Storkus
Curt Storlazzi
Benjamin Storm
Philip Storm
Laurent Storme
Jorge Storniolo
Brian Storrie
Allan Storr-Paulsen
Glenn Storrs
Kate Storrs
Julie Stortz
Jay Storz
Carleen Stoskopf
Paul Stothard
Shannon Stott
Travis Stoub
Rudi Stouffs
Petr Stourac
Jason Stout
Michael Stout
Daniel Stover
John Stover
C. Jill Stowe
Ryan Stowe
Dan Stowell
Sean Stowell
Lisa Stowers
Stefan Strack
Travis Stracker
Giovanni Stracquadanio
Marcin Straczkiewicz
Larissa Straffon
Uwe Strähle
Jana Strahler
Diana Stralberg
Daniel Stram
Sebastiano Stramaglia
Bjornheine Strand
Robin Strand
Sven-Erik Strand
Morten Tune Strandberg
Sara Strandberg
Ariana Strandburg-Peshkin
Blair Strang
Saverio Stranges
Cinzia Strano
Dominik Strapagiel
Giacomo Strapazzon
Janette Strasburger
Reto Strasser
Beverly Strassmann
Lorraine Strathie
Jan Stratil
Ariane Stratmann
Gareth Stratton
Kelly Stratton
Margaret Stratton
Christian Stratz
Adam Straub
Timothy Straub

Eberhard Straube
Robert Straubinger
Lawrence Straus
Suzana Straus
J. Strauss
Roland Strauss
Daniel Strawn
Stephen Stray
Nathalie Strazielle
Joanna Streck
Charles Streckfus
Carl Streed
Richard Street
Timothy Street
Kristi Streeter
Renate Strehlau
Matthew Strehlow
David Streiner
Elani Streja
Peter Strelan
Marina Strelin
Gillian Stresman
Paul Stretesky
Enrica Strettoi
Caroline Strevens
Laura Streyffeler
Pasquale Striano
Henry Strick van Linschoten
Bronson Strickland
Dudley Strickland
Justin Strickland
J. Rudi Strickler
Jennifer Strickler
Rachel Strickman
Ylva Strid
Ella Striem-Amit
Karen Strier
Alfredo Strigazzi
Rob Striker
Maria Giuseppina Strillacci
Eve Stringham
Rebecca Stringwell
Raffaele Strippoli
Oliver Strobel
Philipp Ströbel
Bernhard Strobl
Clare Strode
Katerina Stroffekova
Albrecht Stroh
William Strohl
Erhard Strohm
Deborah Stroka
Anders Strom
Bodil Strom Holst
Patrick Stroman
Birgit Strommenger
Allan Strong
Amy Strong
Alex Strongin
Karien Stronks
Robert Strosznajder
David Stroud
Donna Stroup
Annemarie Stroustrup
Nicholas Stroustrup
Claudio Struchiner
Lisa Strug
Richard Strugnell
Yael Strulovici-Barel
Tara Strutt
Weston Struwe
Dieter Struyf
Sarah Strycharz-Glaven
Robert Strzodka
Amy Stuart
Beth Stuart
Bryan Stuart
Jeff Stuart
Andrew Stubbs
Kent Jason Stuber
Alexi Stuchebrukhov
Heiko Stuckas
Jeanne Stuckey
Joseph Studer
Romain Studer
Susan Studlar
Eva Stüeken
Pascal Stuelsatz

Snorre Stuen
Steven Stufflebeam
Jan Stuhler
Jens-Hermann Stuke
Mike Stukel
Therese Stukel
Jan-Bernd Stukenborg
Jorg Stulke
Phyllis Stumbo
Jason Stumhofer
Andrew Stumpf
Richard Stumpf
Jason Stumpff
Nicole Stupka
Christina Sturesson
Erich Sturgis
James Sturgis
Armin Sturm
Ekkehard Sturm
Elena Sturm
Lynne Sturm
Richard Sturm
Andrew Sturman
Roger Sturmey
Edward Sturrock
Franck Sturtz
Nico Stuurman
Olaf Stüve
Norman Stuzig
Besiki Stvilia
Craig Styan
J. Styczynski
Iain Styles
Charis Styliadis
Nicholas Stylopoulos
Jenny Stynoski
John Styrsky
Anselm Ting Su
Bin Su
Chia-Ting Su
Chien-Wei Su
Chun-Hsu Su
Chunlei Su
Dejun Su
Emily Chia-Yu Su
Fengge Su
Fengxi Su
Guanfang Su
Guifu Su
Guosheng Su
Hairui Su
Hua Su
Huabo Su
Huanxing Su
Jennifer Su
Jianguo Su
Jianmin Su
Kang-Cheng Su
Kang-Yi Su
Leona Yi-Fan Su
Ming-Wei Su
Qi Su
Ran Su
Ren-Wei Su
Rui Su
Ruitao Su
Shi-Bing Su
Timothy Su
Tung-Hung Su
Vincent Yi-Fong Su
Wei Su
Wei-Juin Su
Wei-Wen Su
Wenqing Su
Wr Su
Xiong Su
Yi Su
Yi-Hsien Su
Yingchao Su
Ying-Hsiu Su
Yu-Ru Su
Zhen Su
Zheng Su
Zhixi Su
Zhi-Yuan Su
Zi-Ren Su
Carlos Suabuste
David Suarez

Juan Suarez
Marina Suarez
Luis Suarez-Arrones
Mayte Suarez-Farinas
Benjamin Suarez-Jimenez
Eduardo Suárez-Morales
Aroa Suarez-Vega
Jose Suazo
Papasani Subbaiah
Gunter Subbarao
Ishwaria M. Subbiah
Vignesh Subbian
Damien Subit
Marion Subklewe
Bala Subramaniam
Mangala Subramaniam
Mythily Subramaniam
Renuka Subramaniam
Srinivasa Subramaniam
Poorna Subramanian
Prem S. Subramanian
Sandeep Subramanian
Shivashankar Subramanian
Sujha Subramanian
Manivannan Subramaniyan
Arohan Subramanya
A. Subramanyam
Bhadriraju Subramanyam
Enketeswara Subudhi
Matus Sucha
Stepan Suchanek
Carmen Sucharov
Jordan Suchow
John Suckling
Sandra Šućurović
Kamal Sud
Arnold J. Suda
Eneida Yuri Suda
Goki Suda
Mateus Sudano
Mysore Sudarshana
Sonja Sudarski
Ken Sudduth
Staci Sudenga
Matthew Suderman
Aditya Sudhalkar
G. Sudhandiran
Raghavi Sudharsan
Rathore Sudhir
Hari Kishan Sudini
Runglawan Sudmoon
Hideki Sudo
Bertrand Sudre
Claudia Suemoto
Garret Suen
Jau-Ling Suen
Tadahiro Suenaga
Jerome Sueur
Toshiaki Sueyasu
Piotr Suffczynski
A. M. Sufrinko
Kunio Sugahara
Alan Sugar
Kenji Sugase
Akira Sugawara
Heather Sugden
Kent Sugden
Kristoffer Sugg
Michael Sughrue
Yukiko Sugi
Genichi Sugihara
George Sugihara
Shunichi Sugihara
Bambang Sugiharto
Kazuhiro Sugimoto
Ken Sugimoto
Koichi Sugimoto
Manabus Sugimoto
Haruhiko Sugimura
Koichiro Sugimura
Keishi Sugino
Akiko Sugio
Yodo Sugishita
Minoru Sugiura
Yoshinori Sugiura
Hitoshi Sugiyama
Catur Sugiyanto
Meryam Sugulle

Byung-Chang Suh
Insoo Suh
Sang-Il Suh
Se Won Suh
Young So Suh
Yousin Suh
Metka Suhadolc
Ferenc Suhai
Frank Suhling
Klaus Suhling
Karsten Suhre
Dawen Sui
Feng Sui
Guangchao Sui
Jian-Feng Sui
Jing Sui
Na Sui
Xuemei Sui
Zhenghong Sui
Helen Suich
Clara Suied
E. Sujatha
N. Sujatha
Gintarė Sujetovienė
Heung-Il Suk
Ki Tae Suk
Theresa Sukal-Moulton
Darin Sukha
Igor Sukhotnik
Evi Sukmaningrum
Olga Sukocheva
Naeti Suksomboon
Yaowalark Sukthana
Sunitha Sukumaran
Jang Sukwoo
Irshad Sulaiman
Zaharah Sulaiman
Amrita Sule
Preeti Sule
Muhammad Saibu Suleiman
Noor Suleiman
Mehrunisha Suleman
Samir Sulemane
Courtney Sulentic
Hafiz Ansar Rasul Suleria
Edyta Suliga
Danielle Sulikowski
Sara Suliman
Sharain Suliman
Walid Suliman
Giorgia Sulis
Paul Sullam
Amanda Sulliva
Alexis Sullivan
Christopher Sullivan
Cris Sullivan
Daniel Sullivan
David Sullivan
Edith Sullivan
Helen Sullivan
Jennifer Sullivan
Justin Sullivan
Kelly Sullivan
Margaret Sullivan
Noelle Sullivan
Robert Sullivan
Ryan Sullivan
S. Mazeika Sullivan
Stephen Sullivan
William Sullivan Jr.
Gerhard Sulo
Sławomir Sułowicz
Ronan Sulpice
Ali Sultan
Arshiya Sultana
Hameeda Sultana
James Sulzer
Raymond Kim Wai Sum
Daniel Suma
Ronen Sumagin
Shubhankar Suman
Saulius Sumanas
Hector Sumano
Surahyo Sumarsono
Petra Sumasgutner
Irene Sumbele
Rachita Sumbria
Naokata Sumitomo

Kenta Sumiyama
Artur Summerfield
Tina Summerfield
Glenn Summerhayes
David Summers
Jamie Summers
Jennifer Sumner
Baozhong Sun
Bo Sun
Bob Sun
Chang-Guk Sun
Chao Sun
Chen Sun
Cheng Sun
Chengming Sun
Cheuk-Kwan Sun
ChuanBao Sun
Congshan Sun
Daekyu Sun
Daniel Sun
Deqing Sun
Dianjianyi Sun
Dianqing Sun
Dongxiao Sun
Fang Sun
Fei Sun
Feng-Hua Sun
Gang Sun
Ge Sun
Gui-Quan Sun
Haibo Sun
Haixiang Sun
Hai-Xiang Sun
Hao Sun
Haoran Sun
Hong Sun
Hongbing Sun
Hong-Qiang Sun
Hongying Sun
Hua Sun
Jiajun Sun
Jian Sun
Jian-Guo Sun
Jianyuan Sun
Jie Sun
Jing Sun
Jingchen Sun
Jingyong Sun
Jinqiao Sun
Jinsheng Sun
Junkui Sun
Junming Sun
Kai Sun
Keer Sun
Kehui Sun
Kiwi Sun
Kun Sun
Lei Sun
Lena Sun
Li Sun
Liang Sun
Lili Sun
Limin Sun
Liwei Sun
Li-Wei Sun
Luni Sun
Lun-Quan Sun
M. Sun
Mei Sun
Peng Gang Sun
Pengming Sun
Peter Sun
Qifu Sun
Quanbin Sun
Rui Sun
Ruping Sun
Sheng Sun
Shengkai Sun
Shiwen Sun
Stella Sun
Tianying Sun
Wei Sun
Weili Sun
Weina Sun
Xi Sun
Xiaodan Sun
Xiaodong Sun
Xiaojuan Sun

Xiaoming Sun
Xiaoqun Sun
Xin Sun
Xinghuai Sun
Xingmin Sun
Xuecheng Sun
Xuezheng Sun
Xuxu Sun
Y. S. Sun
Yan Sun
Yanlong Sun
Yanni Sun
Yanshuo Sun
Yan-Xia Sun
Yi Sun
Yihong Sun
Ying Sun
Yingpu Sun
Yongle Sun
Yougang Sun
Yuchun Sun
Yuefang Sun
Yukun Sun
Yun Sun
Yuna Sun
Yunfu Sun
Yuxiang Sun
Yuyang Sun
Yvonne Sun
Zeyi Sun
Zhaogui Sun
Zhenan Sun
Zhengrong Sun
Zhi Sun
Zhichao Sun
Zhonghua Sun
Zhongren Sun
Zhongwu Sun
Zhuo Sun
Yoshihide Sunada
Hiroshi Sunaga
Hajime Sunagozaka
James Sundali
Harshavardhan Sundar
Isaac Sundar
Maria Sundaram
Arumugam Sundaramanickam
P. Sundararaj
T. Sundararaman
Gobalakrishnan Sundaresan
Mackenzie Sunday
Eric Sundberg
John Sundberg
Per Sundberg
Johanne Sundby
Roger Sunde
Ulrik Sundekilde
Sridhar Sunderam
Benedikt Sundermann
Jag Sunderram
Josefin Sundh
Debasish Sundi
Inger Sundström-Poromaa
Jung-Joon Sung
Kyung Rim Sung
Li-Ying Sung
Sheng-Feng Sung
Shih-Hsien Sung
Sibum Sung
Yon Sung
Sujatha Sunil
Lakshmi Sunkara
Venkata Sunkesula
Per Sunnerhagen
Gary Sunter
Jack Sunter
Zacharias Suntres
Rainer Suntz
Zhili Suo
Tarja Suominen
Taniawati Supali
Matej Supej
Alexander Supin
Stéphane Supiot
Sivalingam Suppiah
Vijay Suppiah
Vishnu Suppiramaniam
Claudiu Supuran

Marc Suquet
G. K. Suraishkumar
Benjamin Suratt
Satya Surbhi
Daniel Surder
Sripathi Sureban
Amritha Suresh
C. G. Suresh
M. Suresh
Sahana Suresh
Sridevi Sureshkumar
Michael Surette
Young-Joon Surh
Luca Surian
Lilis Surienty
Roman A. Surmenev
Ranu Surolia
Alexey Surov
Peter Surový
Agnieszka Surowiecka
Ben Surridge
Suleman Surti
Saurabh Suryavanshi
Fetzner Susanne
John Suschak III
Karolina Susek
Petri Susi
Marie Lucie Susini
Edward Susko
Heinz-Martin Süß
Michael Sussman
Davide Susta
Angela Sutan
Vijay Sutariya
Alastair Sutcliffe
Catherine Sutcliffe
Daniel Suter
Steven Suter
Mehul Suthar
Clare Sutherland
Heidi Sutherland
Kate Sutherland
William (Bill) Sutherland
Tuangporn Suthiphongchai
C. Suthisisang
Angelina Sutin
Carolyn Sutter
Gerd Sutter
Catherine Suttle
Alexandra Sutton
Scott Sutton
Steven Sutton
Troy Sutton
Ariana Sutton-Grier
Michael Suvak
Masataka Suwa
Keita Suwabe
Abdulhadi Suwandi
Suhendro Suwarto
Walter P. Suza
Akifumi Suzuki
Ayako Suzuki
Haruo Suzuki
Harzu Suzuki
Hideaki Suzuki
Hidenori Suzuki
Hirokazu Suzuki
Hitoshi Suzuki
Kohta Suzuki
Koji Suzuki
Madoka Suzuki
Masami Suzuki
Masatoshi Suzuki
Nobuhiro Suzuki
Rie Suzuki
Rumiko Suzuki
Shinsuke Suzuki
Susumu Suzuki
Taichi Suzuki
Toshio Suzuki
Tsukasa Suzuki
Wendy Suzuki
Yoshio Suzuki
Yuichiro Justin Suzuki
Yuichiro Suzuki
Yuko Suzuki
Yuriko Suzuki
Yusuke Suzuki

Noriko Suzuki-Kemuriyama
Ramesh Sv
Anita Svadzian
Mona Svanteson
Mia Svantesson
Staffan Svard
John Svaren
Alena Svatkova
Veronica Svedhem
Pernilla Svefors
Emeritus Sven Beer
Erik Svendsen
Per Svenningsen
Henrik Svensen
Birte Svensson
Lidija Šver
Yuriy Sverchkov
Oleksandr Sverdlov
Nicola Sverzellati
Valentina Svicher
Elena Svirshchevskaya
John Swaddle
Beth Swadener
Banikalyan Swain
Lija Swain
Prafulla Swain
Scott D. Swain
Subhashisa Swain
Michelle Swainson
Daniel Swale
C. Swallow
Sunil Swami
Viren Swami
Issac Swamidoss
Aishwarya Swaminathan
Kunchithapadam Swaminathan
Mallikarjuna Swamy
Kristine Swan
Pamela Swan
Dawn Swancutt
Manon Swanenburg
Jennifer Swann
William Swann
Aimee-Noelle Swanson
David Swanson
John Swanson
Kelly Swanson
Kendall Swanson
Kenneth Swanson
Mark Swanson
Randel Swanson
Raymond Swanson
Clarence J. Swanton
Seshadri Swapna
Walter Swardfager
Gaurav Swarnkar
Manuj Swaroop
Daan Swarts
Ann Swartz
Justin Swartz
Talia Swartz
Wilf Swartz
Andrea Swartzendruber
Ghanshyam Swarup
Sanjay Swarup
David Swayne
Andrew Sweatt
Karen Sweazea
Gote Swedberg
David Swedler
Emma Sweeney
Marva Sweeney
Shannon Sweeney
Torres Sweeney
Kevin Sweet
Samuel Sweet
Tracy Sweet
Annika Sweetland
Andrea Swei
Ingemar Swenne
Alton Swennes
Jennifer Swenson
Russell Swerdlow
Elfie Swerts
Tyson Swetnam
Keith Swetz
Luc Swevers
Edwin Swiatlo

Juls Swierczynski
Pawel Swietach
David Swigon
Boyd Swinburn
Jean Swings
Eva Swinnen
Lode Swinnen
David Swinney
Marc Swiontkowski
Briony Swire
Moshe Swissa
Piotr Świtaj
Marek Switonski
Gregory Sword
Laura Swystun
Jay Sy
Jocelyn Sy
Ming Hui Sy
S. Syafwan
Kirtimaan Syal
Adam Sybilski
Laura Sycuro
Bukhari Syed
Yasir Syed
Martin Sykora
Peter Sykora
Francesca Sylos-Labini
Øystein Sylta
Gabriel Sylvestre
Marie-Pierre Sylvestre
George Symeon
Craig Symes
Matthew Symmonds
A. G. Symon
Erin Symonds
Anne Synnes
Grazyna Sypniewska
Vana Sypsa
Astri Syse
Aniko Szabo
Csaba Szabo
Eva Szabo
Judit Szabo
Les Szabo
Sandor Szabo
Olga Szafran
Szymon Szafranski
Tamas Szakmany
Jirina Szakova
Gabor Szalai
Gabriella Szalai
Orsolya Szalardy
Balazs Szalontai
Ildiko Szanto
Monika Szczecińska
Mark Szczelkun
Izabela Szczerbal
Rhonda D. Szczesniak
Paweł Szcześniak
Marc Sze
Sing-Hoi Sze
Andrea Szekely
Robin Szekely
Julia Szekeres Bartho
Leszek Szenborn
Julia Szendroedi
J. A. Szental
Pierre Szepetowski
Nicholas Szerlip
Nick Szerlip
Cheuk-Chun Szeto
Bernadeta Szewczyk
Nathaniel Szewczyk
Timothy Szewczyk
Bożena Szewczyk-Taranek
Damian Szklarczyk
Radek Szklarczyk
Grazyna Szklarz
Tomasz Szmatola
Anna Szmigielska-Kaplon
Kristin Sznajder
Daniel Sznycer
Courtney Szocs
Cassandra Szoeke
Agnieszka Szostak
R. Sztajzel
Attila Szücs
Pawel Szulc

Malgorzata Szumacher-Strabel
Grzegorz Szumski
Tamas Szuts
Cody Szuwalskid
Jo Szwarc
Maciej Szydlowski
Krzysztof Szyfter
Monika Szymańska-Czerwińska
Caroline Szymanski
Christine Szymanski
Laura Szymanski
Mariusz Szymczak
Sylke Szymczak
Mieczyslaw Szyszkowicz
Marie Sθmon
