# Supplementary material for: PLOS ONE 2017 Reviewer and Editorial Board Thank You
Source: PLoS One. 2018 Mar 15;13(3):e0194158. doi: 10.1371/journal.pone.0194158 (PMC5854357; doi:10.1371/journal.pone.0194158)
Supplement: S5 Reviewer List — Alphabetical list of 2017 PLOS ONE peer reviewers (T-Z). (PDF) [file pone.0194158.s007.pdf]

PLOS | ONE

PLOS ONE would like to thank all those who reviewed on behalf of the journal in 2017:

Leen M. 't Hart
Martinek T.
Prathna T. C.
Lakhanpal T. N.
Maarten Taal
Leonie Taams
Lawrence Tabak
Joel Tabak-Sznajder
Valentina Tabanelli
Lucia Tabares
Yasuhiko Tabata
Massimo Tabaton
Luai Tabaza
Karen Tabb
Christoph Tabeling
Barbara Taborsky
Michael Taborsky
Guillaume Tabouret
Takahiro Tabuchi
Angel Tabullo
Anne-Marie Taburet
Kaissar Tabynov
Evelina Tacconelli
Chakrit Tachaaphaikoon
Shin-Ichiro Tachibana
Toshiya Tachibana
Yuuya Tachiki
Frank Tacke
Sebastian Tacke
Björn Tackenberg
Justin Tackney
Micky Tackx
Hayato Tada
Yuko Tada
Mano Tadaaki
Miki Tadaishi
Maria Taddei
Niccolò Taddei
Zerihun Tadele
Bernard Hope Taderera
Tamara Tadich
Duje Tadin
Satyashankara Tadinada
Kim Tae Hee
Jan Taeymans
Abbas Tafakhori
Marco Tafani
Hooman Tafreshi
Angela Taft
Benjamin Taft
Ali Tafti
Takashi Tagami
Elya Tagar
Ozlem Tagay
Kaitlin Tagg
Clifford Taggart
Florian Tagini
Michele Tagliabue
Pierosandro Tagliaferri
Maurizio Taglialatela
Nevio Taglieri
Hideki Taguchi
Mioko Taguchi
Tetsushi Taguchi
Yuzuru Taguchi
Ameer Taha
Zahari Taha
Sarah Tahamont
N. Tahara
Ali Biala Taher
Hoda Taheri
Masoud Tahmasian
Chi-Ming Tai
Chun-Hwei Tai
Dar-In Tai
Leon Tai
Robert H. Tai
Julien Taileur

You-Lin Tain
John Tainer
Men-Wong Taing
Michael Tainsky
Emanuela Taioli
Joona Taipale
Hanna Taipaleenmaeki
Kunihiko Taira
Karen Tait
Leigh Tait
Robert Tait
Rhim Taiyoun
Maral Tajerian
Yuki Tajika
Yuko Tajima
Naoki Tajiri
Endre Takács
Károly Takács
Ayato Takada
Ichiro Takada
Silvia Takada
Tappei Takada
Toshihiko Takada
Yoshikazu Takada
Seido Takae
Atsushi Takagi
Daisuke Takagi
Hideki Takagi
Hiroshi Takagi
Satoshi Takagi
Yasuharu Takagi
Yasushi Takagi
Yuichiro Takagi
Kazuo Takahama
Teruhiko Takahara
Aki Takahashi
Akiyoshi Takahashi
Chris Takahashi
Hirokazu Takahashi
Hironobu Takahashi
Ichiro Takahashi
Katsuhide Takahashi
Kayoko Takahashi
Kazuo Takahashi
Kazutaka Takahashi
Makoto Takahashi
Masafumi Takahashi
Masanobu Takahashi
Masashi Takahashi
Michio Takahashi
Mizuki Takahashi
Naoki Takahashi
Taro Takahashi
Yoshimasa Takahashi
Yoshitaka Takahashi
Idris Takai
Yoshimi Takai
Tetsuya Takaishi
Akinobu Takaki
Jun'Ya Takakura
E. Takala
Shigeyuki Takamatsu
Shinzo Takamori
Iseki Takamoto
Akiteru Takamura
Kenzi Takamura
Hitoshi Takano
Keisuke Takano
Masanori Takano
Shigeru Takano
Takehito Takano
Motoharu Takao
Hiro Takaoka
Tohru Takarada
Tim Takaro
Hajime Takase
Masayuki Takase
Atsuko Takashima
Eizo Takashima
Nao Takashina
Fuyuko Takata
Koichiro Takayama
Yoshiharu Takayama
Hirohide Takebayashi
Soichi Takeda
Yoshiyu Takeda
Naofumi Takehara
Fumio Takei

Yasuchika Takeishi
Minoru Takemoto
Genzo Takemura
Kosuke Takemura
Yukinori Takenaka
Nikawa Takeshi
Norio Takeshita
Shin Taketa
Teruko Taketo
Fumihiko Takeuchi
Hideaki Takeuchi
Hideyuki Takeuchi
Masaru Takeuchi
Nobuto Takeuchi
Osamu Takeuchi
Yasuo Takeuchi
Yutaka Takeuchi
Aya Takeyama
Naoshi Takeyama
Hajime Takikawa
Toru Takimoto
Fumio Takizawa
Willem Takken
Mary Takla
Elad Tako
Miklós Takó
Harold Takooshian
Bertin Takoutsing
Mohamed Taktak
Tomoyuki Takura
Hana Takusagawa
Reshef Tal
Kawsar Talaat
Maria Talavera
Douglas Talbert
Robert Talbot
Evelyn Talbott
Alan Talevi
Nicole Talge
Sheikh Talha
Farid Talih
Sanna Talja
Monica Taljaard
Anne Talkington
Emmanuel Talla
Venu Talla
Victor A. Tallada
Daniel Talley
Giovanni Tallini
Melissa Tallman
Michelle Tallquist
Narendra Talluri
Henk Talma
William Talman
Dibyendu Talukdar
Rupjyoti Talukdar
Antoine Taly
Emily Tam
Julian Tam
Kingsley Tam
Nicholas Tam
See-Ying Tam
Masanori Tamaki
Kenichi Tamama
Phanourios Tamamis
Dietmar Tamandl
Bishal Tamang
Rakesh Tamang
Sujata Tamang
Mayumi Tamari
Josep Lluis Tamarit
Monica Tamariz
Francesca Tamarozzi
Peter Tamás
Aracely Tamayo
Evandro Tambarussi
Arielle Tambini
Ernest Tambo
Robyn Tamboli
Vittoradolfo Tambone
James Tambong
Cecilia Tamborindeguy
Rosemary Tambouret
Jerome Tamburini
Jeremy Tame
Andrea Tamellini
Silvia Tamez Gonzalez
Vaijayanti Tamhane

Marco Tamietto
Eleonora Tamilia
Motohiro Tamiya
Toomas Tammaru
Imke Tammen
Markku Tammi
Athanasios Tampakis
Owen Tamplin
Ranjan Tamuli
Atsushi Tamura
Hiroshi Tamura
Kouichi Tamura
Masahito Tamura
Toshihiro Tamura
Yuichi Tamura
Alex Tan
Andrew Tan
Benjamin Tan
Chee Seng Tan
Cheemeng Tan
Chengquan Tan
Cher Heng Tan
Choo Hock Tan
Christopher Tan
Chrystalle Tan
Dun Y. Tan
Dunxian Tan
Eng-King Tan
Fei Tan
Frankie Tan
Geak Poh Tan
Gene Tan
Heng Wee Tan
Hong Tan
Hong Yien Tan
Hongzhuan Tan
Hui Shan Tan
Jiangning Tan
Jiwei Tan
Joanne Tan
Jun Tan
Kok Wei Tan
Maw Pin Tan
May Loong Tan
Roderick Tan
Rui Zhen Tan
S. Tan
Shyh-Han Tan
Suet-Mien Tan
Susanna K. Tan
Tao Tan
Thuan-Chew Tan
Tina Tan
Tuan Zea Tan
Wei Shen Tan
Wen Tan
Wenjie Tan
Xiao Tan
Xiaochao Tan
Xiaoling Tan
Xuhua Tan
Xungang Tan
Yee-Joo Tan
Yulong Tan
Zhi Tan
Zhibing Tan
Nobuhiro Tanabe
Shihori Tanabe
Aki Tanaka
Eiichi Tanaka
Gouhei Tanaka
Hidekazu Tanaka
Hiroaki Tanaka
Kazuhiro Tanaka
Kenichi Tanaka
Kiwamu Tanaka
Kohichi Tanaka
Leonardo Tanaka
Masafumi Tanaka
Masaru Tanaka
Masayuki Tanaka
Michiko Tanaka
Minoru Tanaka
Motomasa Tanaka
Shigeho Tanaka
Shota Tanaka
Sota Tanaka
Yasuhito Tanaka

Yoichi Tanaka
Yoshikazu Tanaka
Yoshiya Tanaka
Yosuke Tanaka
Yujiro Tanaka
Ivan Tancevski
Tara Tancred
Gianluigi Tanda
Himani Tandon
Pooja Tandon
Vibha Tandon
Robert Tanenberg
Naoko Tanese
Alessandra Tanesini
Lisa Taneyhill
Aihua Tang
Alice Tang
Chengxiang Tang
Ching-Wen Tang
Chunbo Tang
Chuyang Tang
Clara Tang
Cynthia L. Tang
Dale Tang
Fei Tang
Fu Lei Tang
Fulei Tang
Gau-Jun Tang
Grace Tang
Guixiang Tang
Hansong Tang
Hao Tang
Hong-Wu Tang
Huilin Tang
James Tang
Jianwu Tang
Jihua Tang
Jing Tang
Jinjun Tang
Julian Tang
Juliet Tang
Jun Tang
Li Tang
Liang Tang
Lu Tang
Mei San Tang
Ming Tang
Mingchun Tang
Pan Tang
Peifu Tang
Pingbo Tang
Qiyi Tang
Rong Tang
Rui Tang
Sen-Lin Tang
Shangfeng Tang
Shanjiang Tang
Shao-Jun Tang
Shaoxun Tang
Shibo Tang
Shijie Tang
Shuang Tang
Simon Tang
Sindy Tang
Tong Boon Tang
W. H. Wilson Tang
Wei Tang
Weiming Tang
Wenbo Tang
Wenwu Tang
Willcyn Tang
Xiangdong Tang
Xiangming Tang
Xiaojia Tang
Xiaojun Tang
Xiaolin Tang
Xu Tang
Xueying Tang
Yan Tang
Yang Tang
Yingying Tang
Yuanyuan Tang
Yunqiang Tang
Yuqing Tang
Yuxing Tang
Zhangui Tang
Zhichuan Tang
Zhili Tang

Viroj Tangcharoensathien
Michael Tangermann
Balamurugan Tangiisuran
Charles Nkufi Tango
Vin Tangpricha
Arnaud Tanguy
Jun Tani
Toshiyasu Taniguchi
Kousuke Tanimoto
Ryuta Tanimoto
Karen Tanino
Yoshinori Tanino
Yoshihiro Taniyama
Toshihiro Tanizawa
Margaret Tankard
Angelo Tanna
Charles Tanner
Hannah Tanner
Rachel Tanner
Jun Tanno
Lisa Tannock
Elias Tannous
Jason Tanny
Kouichi Tanonaka
Issam Tanoubi
Mathew Tantama
Udaya Tantry
Michele Tanturli
Mohsin Tanveer
Pradeep Tanwar
Anna Tan-Wilson
Bahattin Tanyolac
Elisabetta Tanzi
Dacheng Tao
Fangbiao Tao
Fei Tao
Guoyu Tao
Haiying Tao
Jun Tao
Lei Tao
Mi-Hua Tao
Rui Tao
Sha Tao
Sheng-Ce Tao
Shi Tao
Wendong Tao
Wensi Tao
Xiaofeng Tao
Ye Tao
Yongfu Tao
Samir Taoudi
Deodato Tapete
Milagritos Tapia
Vesa Tapiainen
Soile Tapio
Ibrahim Tapki
Stephanie Taplin
Juhani Taponen
Karthik Tappa
Marta Tapparo
S. W. Tappin
L. Tapsell
Linda Tapsell
Niteen Tapuria
Liudmila Tarabashkina
Jakub Taradaj
Avijit Tarafdar
Stefano Tarantini
Giovanni Tarantino
Umberto Tarantino
Alex Taranto
Howard Taras
Eustachio Tarasco
Sarunas Tarasevicius
Francisco Jose Tarazona Santabalbina
John Tarbell
Adi Tarca
Eric Tardif
Daniel Tardona
Florence Tardy
Meghana Tare
Corrado Tarella
Douglas Taren
Md. Tareque
Aditya Tarigoppula
Amare Tariku
Sergey Tarima
Edith Tarimo

Amara Tariq
M. Umer Tariq
Mohsin Tariq
Muhammad Tariq
Shema Tariq
Ina Tarkka
Susan Tarlo
Kustiariyah Tarman
Alexander Tarnutzer
Paolo Tarolli
Aaron Tarone
Margaret Tarpley
Garth Tarr
Philip Tarr
Wassim Tarraf
Giulio Tarro
Achille Tarsitano
Gian Tartaglia
Daniele Tartarini
Barbara Tartarotti
Rodrigo Tarté
Phillip Taru
Takashi Tarumi
Rebecca Tarvin
Laura Tarzia
Neslihan Tas
Sheikh Tasduq
Alexandre Tashima
Satoshi Tashiro
Yasutaka Tashiro
Donald Tashkin
Sarah Tasian
Bosiljka Tasic
Ljubica Tasic
Robert Tasker
Arzu Taskiran Comez
Admasu Tassew
Laura Tassi
Bruno Tassin
Cristina Tassorelli
Chrysoula Tassou
Dimitris Tatakis
Marc Tatar
Andrey Tatarenkov
Tatiana Tatarinova
Kelly Tatchell
Jacqueline Tate
James Tate
Nicholas Tate
Shunsuke Tatebe
Yutaka Tateda
Satyanarayana Tatineni
Amanda Tatler
Rachael Tatman
Ichiro Tatsuno
Masami Tatsuno
Ian Tattersall
Pierre Tattevin
Lorenzo Tattini
Marco Tatullo
James Tatum
Margaret Taub
Mary Taub
Uma Tauber
Uwe Täuber
Jessica Taubert
Stefan Taubert
Svenja Taubner
Rahil Taujale
Douglas Taupin
Jean-Luc Taupin
Norma Tavakoli
Alessandro Tavano
Davi Tavares
Ivan Tavares
Joana Tavares
Joao Manuel R. S. Tavares
Kaio Tavares
Ali Tavassoli
Negar Tavassolian
Iman Tavassoly
Giacomo Tavecchia
Gianluigi Taverna
Dan Tawfik
Bill Tawil
Bouchra Tawk
Alvin Tay
Stacey Tay

Sun Tee Tay
Chandrakant Tayade
Prakriti Tayalia
Ichiro Tayasu
Muzahir Tayebjee
Aysegul Taylan Ozkan
Hande Taylan Sekeroglu
Nancy Tayles
A. Taylor
Allan Taylor
Andrea Taylor
Andrew Taylor
Anna Taylor
Barbara Taylor
Brandie Taylor
Brett Taylor
Caroline Taylor
Cath Taylor
Chloe E. Taylor
Christopher Taylor
D. Taylor
Dane Taylor
David Taylor
Douglas Taylor
G. Taylor
Graham Taylor
Janet Taylor
Jeremy Taylor
John Taylor
John-Stephen Taylor
Kira Taylor
Lauren Taylor
Mackenzie Taylor
Mark Taylor
Matt Taylor
Matthew Taylor
Michael Taylor
Nicholas Taylor
Nigel Taylor
Paul Taylor
Peter Taylor
R. Taylor
Rebecca Taylor
Richard Taylor
Robin Taylor
Rod Taylor
Roy Taylor
Sara Taylor
Simon Taylor
Stephanie Taylor
Steve Taylor
Steven Taylor
Susan Taylor
W. Robert Taylor
William Taylor
Zachary Taylor
Martin Taylor-Rowan
Jean-Marc Taymans
Saad Tayyab
Asmaa Tazi
Andre Tchernof
André Tchernof
Veronika Tchesnokova
Anastase Tchicaya
Nicolas Tchitchek
J. Michel Tchuenche
Hein Te Riele
Mark Teaford
Holly Teagle
Gregory Teague
Chloe Teasdale
Rohan Teasdale
Sarah Teatero
Christoph Tebbe
Angsana Techatassanasoontorn
Vania Techio
Karl Technau
Frank Technow
Sang Teck Oh
Stacey Tecot
Anna Tedeschi
Francesco Tedesco
Serena Tedesco
Andrew Tee
Y. K. Tee
Lawrence Teen
K. Teeuwen
Chee-Keng Teh

Cheng Teh
Cindy Shuan Ju Teh
Jessica Teh
Louise Teh
Ying Wah Teh
Yit Teh
Peta Tehan
Parsa Tehranchian
Hajime Tei
Beverly Teicher
Markus Teige
John Teijaro
Petteri Teikari
Ebrahim Teimoury
Yee Yang Teing
Martin Teintze
Justin Teissié
Ana Teixeira
Andreia Sofia Teixeira
António Teixeira
Cristina Teixeira
Fernanda Teixeira
Fernando Teixeira
Gustavo Teixeira
Januana Teixeira
Laetitia Teixeira
Luis Teixeira
Marta Teixeira
Renan Teixeira
Robson Teixeira
Silmar Teixeira
Armando Teixeira-Pinto
Carlos Tejeda-Guzmán
Mathurin Tejiokem
Aylin Tekes
Daniel Tekiela
Ramazan Tekin
Ayse Tekinay
Yenenesh Tekletsion
Alula Teklu
Gebreyohans Teklu
Rajeshwar Tekmal
Kardi Teknomo
Amalio Telenti
Cody Telep
Pedro Teles
Fabrice Teletchea
Joseph Telfair
Janice Telfer
Natalie Telis
Lisa Tell
Paolo Tell
Ross Tellam
Paola Tellaroli
Olivier Telle
Guillermo Tellez
George Tellides
Charles Tellier
Jennifer Telling
Richard Tello
Ariel Telpaz
Narasaraju Teluguakula
Fatih Temel
Fehminaz Temel
Johnna Temenoff
Leonard A. Temme
Liesbet Temmerman
Marleen Temmerman
Herbert Tempfer
Victoria Templer
Matthew Templeton
Andre Tempone
Rachel ten Bruggencate
Arina ten Cate-Hoek
Kevin ten Haaf
Twan ten Haaf
Huib ten Napel
J. ten Oever
Manuel Tena-Sempere
Scott Tenenbaum
Tobias Tenenbaum
Alexander Teney
Andrea Teng
Ching-Hao Teng
Edmond Teng
Lisong Teng
Michael Mu Huo Teng
Michael Teng

Peng Teng
Shaolei Teng
Sheng Teng
Weiping Teng
Yong Teng
Yuanwen Teng
Yue Teng
Zhaogang Teng
Zhongzhao Teng
Tengku Ismail Tengku Alina
Shivendra Tenguria
Fitsum Teni
Jonathan Tennant
Henk Tennekes
Kamani Tennekoon
Jacob Tennessen
Claudio Tennie
Joan Teno
Carol Tenopir
Leonardo Tenori
Lyson Tenthani
Tenzin Tenzin
Chee How Teo
Miang Chneh Teo
Stephen Teo
Wei-Peng Teo
Catalin Teodoriu
Jose Teodoro
Luciana Teofili
Henrique Teotónio
Jetze Tepe
Michele Tepedino
Bojan Tepeš
Beverly Tepper
Clifford Tepper
Sigal Tepper
Benno ter Kuile
Anne ter Wal
Yasuhiko Terada
Masanori Terajima
Jumpei Teramachi
Keisuke Teramoto
Enrique Teran
Adam Terando
Junji Terao
Ei Terasawa
Hiroyuki Terawaki
Maxim Terekhov
Alex Terenin
Dimitrios Terentes-Printzios
Alexander Terent'ev
Andrew Terentis
Dmitry Terentyev
Larisa Tereshchenko
Vinay Tergaonkar
Devin Terhune
Karen Terio
Assel Terlikbayeva
Antonio Terlizzi
Carlos Termignoni
Nigel Ternan
David Ternant
Sten Ternström
Ulrich Terpitz
Nicole Terpolilli
Antonio Terracciano
Niccolo Terrando
Laura Terraneo
Marco Terraneo
Nora Terrasini
Norah Terrault
Teresa Terrazas
Elodie Terrer
Marie-Emilie Terret
Levi Carina Terribile
Alexandre Terrier
Gianluca Terrin
D. Terry
John Terry
Kathryn Terry
Jean Tersak
Jose Teruel
Maria Teruel
Mari Tervaniemi
Serdal Terzi
Valeria Terzi
Massimo Terzolo
Andrew Teschendorff

Rackeb Tesfaye
Amare Teshome
Luigi Tesio
Valentina Tesio
Andreas Teske
Erik Teske
Giovanni Tesoriere
Giulia Tessa
Leticia Tessarolli
Jeffery Tessem
Gizachew Tessema
Alessandro Tessitore
Joerg Tessmar
Kristin Tessmar-Raible
Domenico Testa
Gianluca Testa
Maria Testa
Francois Teste
Anna Testen
Traci Testerman
Raffaele Testolin
Sarada Tetali
David Tetard
Jens Tetens
Kenneth Teter
Ian Tetlow
Sotirios Tetradis
Osamu Tetsu
Tetsuhiro Tetsuhiro Tanaka
Ciro Tetta
Mauro Tettamanti
Hervé Tettelin
Tom Tetzlaff
Michel Teuben
Katrin Teubner
Nicolette Teufel-Shone
Alexander Teumer
Charlotte Teunissen
Rita Tewari
Pierre Yves Teycheney
Michał Teżyk
Jecko Thachil
Anne Thackeray
Rosemary Thackeray
Larry Thaete
Chan Thai
Nhi Thai
Truc Thai
Izabella Thais da Silva
Christoph Thaiss
Madhuri Thakar
Monica Thakar
Maria Thaker
Vrinda Thaker
Neel Thakkar
Prashant Thakkar
Priyank Thakkar
Krishna Thakur
Madhav Thakur
Meghna Thakur
Sunitha Thakur
Piyushimita Thakuriah
Freitas Thales Renato Ochotorena De
Olaf Thalmann
Mau-Luen Tham
Waihong Tham
Kimberlee Thamatrakoln
Bo Thamdrup
Mae Thamer
Markus Thamm
Nisha Thampi
Cindy Thamrin
Van Thai Than
David Thanassi
Rajarajan Thandavarayan
Balamugesh Thangakunam
Shankar Thangamani
Lakshmipriya Thangavel
Sam Thangiah
Panida Thanyasrisung
Dharendra Thapa
Kanchan Thapa
Kusum Thapa
Narbada Thapa
Narendra Thapa
Subash Thapa
Roopa Thapar
Binu Tharakan

Pierre-Louis Tharaux
Eriona Thartori
Andrew Thatcher
Louise Thatcher
Natalie Thatcher
Sean Thatcher
Stefan Thau
Vijey Thayananthan
Wesley Thayer
Zaneta Thayer
Bertram The
Donald Thea
Nipon Theera-Umpon
Steven Theg
Elizabeth Theil
Nathan Theill
Rebecca Theilmann
Gregor Theilmeier
Cathrin Theis
Maphefo Thekiso
Antonia Thelen
Eric Thelin
Michael Thelwall
Katsila Theodora
Theodossis Theodossiou
N. Theodulidis
Dimitrios Theofanidis
Ulrich Theopold
Jakob Theorell
Jenny Theorell-Haglöw
Christine Theoret
Mahamadou Thera
Sebastien Theriault
Alex G. Therien
Christophe Theroude
Manuel Thery
Jörn Theuerkauf
Igor Theurl
Eric Theveneau
Frank Thevenod
Catherine Thevenot
Karin Thevissen
Sascha Thewes
J. G. M. Thewissen
Jay Thiagarajah
Devi Thiagarajan
Helene Thibault
Thierry Thibaut
Benoit Thibodeau
Eric Thibodeau
Kittiphong Thiboonboon
David Thickett
Cora Thiel
David Thiel
Gerhard Thiel
Marco Thiel
Teresa Thiel
Vera Thiel
William Thiel
M. Thiele
Tamara Thiele
Todd Thiele
Barbara Thiem
Tara Thiemann
René Thieme
Friedrich Thienemann
Guillaume Thierry
Denis Thiery
Hans-Jürgen Thiesen
Nils Thiessen
Ingo Thievessen
Bart Thijs
Sofie Thijs
Vincent Thijs
Troels Thim
Bryan Thines
Marco Thines
Tron Frede Thingstad
Chloe Thio
Samuel Thiriot
Nivethida Thirugnanasambandam
Chandini Thirukkumaran
Parthasarathy Thirumala
Soumya Narayani Thirumoorthy
C. Thirunavukkarasu
Søren Skou Thirup
Muthusamy Thiruppathi
Suntaree Thitiwichienlert

Ramkumar Thiyagarajan
Pradeep Thiyyagura
Aaron Thode
Felicitas Thol
Gerold Thölking
David Thom
Dominik Thom
Jeanette Thom
Maria Thom
Matthew Thom
Myriam Thoma
Manuela Thomae
Adam T. Thomas
Andrew Thomas
Anna Thomas
Binu Thomas
Crystal Thomas
David Thomas
Elizabeth Thomas
Freya Thomas
Fridtjof Thomas
Gabriel Thomas
Graham Thomas
Holly Thomas
Isabelle Thomas
James S. Thomas
Jennifer Thomas
John Thomas
Jonathan Thomas
Kevin Thomas
Margaret Thomas
Michael Thomas
Nancy Thomas
Neil Thomas
Nicky Thomas
Olivier Thomas
Owain Thomas
Peter O. Thomas
Peter Thomas
Philipp Thomas
Rae Thomas
Richard Thomas
Roger Thomas
Ronald Thomas
Stephen Thomas
Sufi Thomas
Sunil Thomas
Tim Thomas
Vernon Thomas
Victoria Thomas
Vinai Thomas
Walter Thomas
Wayne Thomas
Yoann Thomas
Andrea Thomas-Bachli
Thierry Thomas-Danguin
Melissa Thomason
Pamela Thomason
Wade Thomason
Margot Thome
Ulrich Thome
Sebastien Thomine
Bart Thomma
Edward Thommes
Edward W. Thommes
Stavros Thomopoulos
Aiko Thompson
Amanda Thompson
Angela Thompson
Azure Thompson
Brennan Thompson
Caroline Thompson
Charee Thompson
Christine Thompson
David Thompson
Debbie Thompson
Diane Thompson
Elizabeth Thompson
Emma Thompson
Engelberta Thompson
Erika Thompson
Hannah Thompson
Helen Thompson
James Thompson
Joel Thompson
John Thompson
Khristy Thompson
Kim Thompson

Lisa Thompson
Loren Thompson
M. A. Thompson
Mary Lou Thompson
Michael Thompson
Nicola Thompson
Paul R. Thompson
Piers Thompson
R. Houston Thompson
Randall Thompson
Robert Thompson
S. Thompson
Sally Thompson
Sandra Thompson
Tess Thompson
Todd Thompson
Victor Thompson
William Thompson
Martin Thoms
Edward Thomsen
Gerry Thomsen
Hauke Thomsen
Henrik Thomsen
Mads Thomsen
Simon Francis Thomsen
Thordis Thomsen
Alison Thomson
Allyson Thomson
Cynthia Thomson
D. Thomson
David Thomson
James D. Thomson
Kimberly Thomson
Michael Thon
Charat Thongprayoon
Ryan Thoni
Peter Thor
Stefan Thor
Fredrik Thoren
Bernard Thorens
Gabriel Thorens
Marianne Thoresen
Gary H. Thorgaard
Henrik Thorlacius
Simon Thorn
Michael Thorne
Sally Thorne
Andrew Thornhill
Daniel Thornton
Hannah Thornton
John Thornton
Justin Thornton
Laura Thornton
Ruth Thornton
Barry Thorp
Jim Thorson
Jon Thorson
Einar Thorsteinsson
Anilkrishna Thota
Malathi Thothathiri
Michael Thouless
Mathieu Thoury
Derrick Thrasher
Timothy Thrasher
Amy Throckmorton
Mahender Thudi
Luiz Thuler
Caroline Thum
Barbara Thumann
S. Thumbi
Michele Thums
Gilles Thuret
Philipp Thurner
Rebecca Thurston
Lelwala Guruge Thushani Shanika
Gregor Thut
Chanitra Thuwajit
Julie Thwing
Baskaran Thyagarajan
Dominic Thyagarajan
Anders Thygesen
Gregory Thyssen
Hanna Tiainen
Appolinaire Tiam
Bin Tian
Bo Tian
Changhai Tian
Chaoguang Tian

Dayong Tian
Dechao Tian
Geng Tian
Jian Tian
Jin Tian
Jing Tian
Kun Tian
Ling Tian
Linwei Tian
Lixia Tian
Miaoying Tian
Ning Tian
Qiang Tian
Renmao Tian
Shiying Tian
Subo Tian
Tianhai Tian
Xiaohong Tian
Xiao-Jun Tian
Xiao-Li Tian
Xing Tian
Ye Tian
Yin Tian
Yixiang Tian
Yuan Tian
Saeed Tiari
Ramires Tibana
Katharine Tibbetts
Helen Tibboel
Iolanda de Fátima Tibério
Shailja Tibrewal
Vladimir Tichelaar
Tobias Tichelbaecker
Andrea Ticinesi
Tamara Ticktin
Nicola Ticozzi
Harm Tiddens
T. Tideman
Rosana Tidon
Emmanuele Tidoni
Andreas Tiede
Thomas Tiefenboeck
Zoe Tieges
Henning Tiemeier
Bea Tiemens
Jean Tien
Ming Tien
Phyllis Tien
Dieu Tien Bui
Adam Tierney
Keith Tierney
Mary Tierney
Hong Van Tieu
Lina Tieu
Sigal Tifferet
Zoran Tiganj
William Tigbe
Anu Tiitsaar
Chris Tijs
Peter Tijssen
Denis Tikhonov
Elena Tikhonova
Ruben Tikidji-Hamburyan
Ritva Tikkanen
Alan Tilbrook
Tamara Tilburgs
Benedikt Till
Gayani Tillekeratne
Liyanaaratchige Tillekeratne
Rebekah Tiller
Douglas Tilley
Richard Tilley
Thomas Tilling
Jochen Tillmanns
Sebastien Tilloy
Antonio Tilocca
Vera Tilson
Susan Tilton
Roland Richard Tilz
Susanna Timeo
Sujan Timilsina
Joerg Timm
Stefan Timm
Reinier Timman
Lori Timm-Davis
Jennifer Timmer
Wilhelmina Timmermans
Brian Timmons

Suzanne Timmons
Brian Timms
Peter Timms
Verlaine Timms
Salme Timmusk
Tonis Timmusk
Maria Timofeeva
Vladimir Timoshevskiy
Winston Timp
Britta Timpane-Padgham
Simon Timpka
Sebastian Tims
Jean-Francois Timsit
Serge Timsit
David Timson
Willard Tinago
Camilla Tincati
Michela Tinelli
Kang Ting
Lauren Tingey
Yotsawan Tinikul
Kara Tinker
Arthur Tinoco
Gisela Tinone
Fausto Tinti
Nathan Tintle
T. Joyce Tiong
Ramreddy Tippana
Donna Tippett
Lynette Tippett
Alice Tipton
Craig Tipton
David Tipton
Virginia Tirino
Franck Tirode
Elena Tirrò
Madhan Tirumalai
Raghunand Tirumalai
Mahesh Tirumkudulu
Daniela Tirziu
Shelley Tischkau
Anna Tischler
Dirk Tischler
James Tisdale
Peter Tiselius
Anders Tisell
Samuel Tisherman
Jean-Christian Tisserand
Fadel Tissir
Joshua Titlow
Vladimir Titorenko
Igor Titov
Derek Tittensor
Claus Tittiger
Arjun Tiwari
Ashutosh Tiwari
M. Tiwari
Manoj Tiwari
Neeraj Tiwari
Rakesh Tiwari
Sharad Tiwari
Shuchita Tiwari
Siddharth Tiwari
Vishvanath Tiwari
Michèle Tixier-Boichard
Hamid Tizhoosh
Michele Tizzoni
Julius Tjelele
Michael Tjepkema
Annelie Tjernlund
Arnt Tjønna
Ivan Tkac
Dominika Tkaczyk
Eric Tkaczyk
Esteban Tlelo-Cuautle
David Tng
Kien Gia To
Matthew To
Ted Toal
Bruno Toaldo
Franklin Toapanta
Toru Tobe
Raquel Tobes
Valentina Tobia
Deirdre K. Tobias
Desmond Tobin
Irene Tobler
Mathias W. Tobler

Carlo Tocchetti
Giuliano Tocci
Douglas R. Tocher
Mamoru Tochigi
Chitoku Toda
Yasuka Toda
Andrew Todd
Brian Todd
Catherine Todd
Jim Todd
Jonathan V. Todd
Michael Todd
Nick Todd
Giuseppe Todde
David Todem
Shusuke Toden
Anne Todgham
Giancarlo Todiere
McWelling Todman
Jelena Todoric
Alexander Todorov
Michail Todorov
Margarita Todorova
Slobodan Todorovic
Stefan Toegel
Jan Toelen
Agnieszka Toepper
David Toews
Benjamin Toff
Claire Toffano-Nioche
Giuseppe Toffoli
Farzad Tofigh
Egon Toft
Søren Toft
Anne Pernille Toft-Petersen
Dieudonnée Togbe
Johannes Töger
Arnaud Tognetti
Massimiliano Tognolini
Gianluca Tognon
Masahiro Tohkin
Rutaiwan Tohtong
Chiaki Toida
Diana Toivola
Jaroslaw Tokarczyk
Daniel Toker
Serhat Tokgoz
Hale Toklu
Savvas Tokmakidis
Andrew Tokmakoff
Hisanori Tokuda
Makoto Tokuda
Daisuke Tokuhara
Fuyuki Tokumasu
Masanori Tokunaga
Kumpei Tokuyama
Shogo Tokuyama
Nicole Tolan
Gergely Toldi
Stefano Toldo
Estefania Toledo
Geissiane Toledo
Luís Felipe Toledo
Néstor Toledo
Roberta Toledo
José Tolezano
D. J. Tolich
Jonas Toljander
Agusti Toll
Travis T. Tollefson
David Tollervey
Keith Tolley
Stephen Tolley
Eleni Tolma
Mate Tolnay
Martijn Tolsma
Michael Tolstorukov
Claudia Toma
Wisam Toma
Boguslav Tomanek
Scott Tomar
Sunil Tomar
Gustavo Tomás
Roman Tomaschitz
Thomas Tomasi
Nicolás Tomasini
Richard Tomasini
Adam Tomasovych

Adam Tomašových
Antonella Tomassetti
Shunji Tomatsu
Stanislas Tomavo
Cândida Tomaz
Carlos Tomaz
Claudio Tomazzoli
Ivan Tomba
Diana Tomback
Jeffery Tomberlin
Francesco Tombola
Huseyin Tombuloglu
Michal Tomczyk
Rade Tomic
Makoto Tominaga
Jun Tomio
Haruaki Tomioka
Kohji Tomita
Luciana Tomita
Natsuo Tomita
Hirofumi Tomiyama
Kazuhito Tomizawa
Jonna Tomkiewicz
Scott Tomlins
Darren Tomlinson
Mark Tomlinson
Ryan Tomlinson
Tucker Tomlinson
Desiree Tommasi
Massimo Tommasino
Niels Tommerup
Akemi Tomoda
Hiroshi Tomoda
Kazunori Tomono
Edward Tomoye
Emile Tompa
Phillip Tomporowski
Alejandro Tomsic
Hoai Ton
Alessandro Tondelli
Claudio Tondo
Leonardo Tondo
Adriano Toneli
Renata Tonelli
Michela Tonetti
Giang Tong
Haijie Tong
Henry Tong
Jingou Tong
Juan Tong
Louis Tong
Philip Tong
Qingchun Tong
Seng Fah Tong
Shaocheng Tong
Shuping Tong
Tiejun Tong
Xiangrong Tong
Xiaogang Tong
Xiuhong Tong
Yan Tong
Yiping Tong
Yufeng Tong
Marcel Tongo
Pierluigi Toniutto
Anke Tönjes
Nicholas Tonks
Utsana Tonmukayakul
J. Tonna
Tor Inge Tonnessen
Marie-Christine Tonon
Giulio Tononi
Ergin Tönük
Lay San Too
Kristen Tooley
Christopher Toomajian
Ryan Toonen
Amir Toor
Zahra Toossi
Karina Top
Evangelos Topakas
Jozsef Topal
Haluk Topaloglu
David Topham
Alexis Topjian
Sascha Topolinski
Sanna Katriina Toppila-Salmi
Mattie Tops

Alireza Torabi
Elena Torban
Nathan Torbick
Leslie Torburn
Maria Torcia
Andrew Torda
Adrian Tordiffe
Michael Tordoff
Rubben Torella
Federico Torelli
Kjell Torén
Bart Torensma
Paul Torgerson
Toshihiko Torigoe
Claudia Torino
Åsa Torinsson Naluai
Dante Torio
Kinya Toriyama
Martin Tornai
James Torner
Daniel Tornero
Maria Lina Tornesello
Jeffrey Tornheim
Luigi Tornillo
Viviana Toro-Ibacache
John Torous
Mia Torpdahl
Fraser Torpy
Andrea Torrão
Ignasi Torre
Ann-Marie Torregrosa
Montserrat Torremorell
Angelo Torrente
Yvan Torrente
Jordina Torrents-Barrena
Arturo Torres
Jaume Torres
Javier Torres
Jiram Torres
Joaquin Torres
José Luis Torres
José Torres
Luisa Torres
Miguel Torres
Ricard Torres
Ricardo Torres
Susan Torres
Tatiana Torres
Joaquin Torres Agudo
Janaina Torres Carreira
Verônica Torres da Costa e Silva
Maria Torres Spicer
Mario Torres-Acosta
Diego Torres-Dueñas
Lisette E. Torres-Gerald
Annelyn Torres-Reveron
Laura Torres-Rovira
Miguel Torres-Ruiz
Jorge Torres-Sánchez
Eduardo Caio Torres-Santos
Sandra Torriani
Elena Torrieri
Laura Torroja
Donald Torry
Torbjørn Torsheim
Alex Torson
Lluis Tort
Diana Torta
Marta Tortajada
Miguel Tortajada-Girbés
Philippe Tortell
Mauro Torti
Maria Teresa Tortola
Paolo Tortora
Anna Maria Tortorano
Avelina Tortosa
German Tortosa
Franco Toscani
Massimiliano Toscano
Michael Toscano
Nicola Toschi
Tiziana Tosco
Anela Tosevska
David Tosh
Sho Toshino
Matsushige Toshinori
Dimiter Toshkov
Simone Tosi

Elona Toska
Christian Toso
Andrea Tosti
Hervé Tostivint
Satish Totey
Arnold Toth
Bettina Toth
Damon Toth
Louis A. Toth
Attila Tóth
Balázs Tóth
Erzsébet Tóth
Emily Toth Martin
Edmond Totin
Patricia Totten
Janet Tou
Takahiro Tougan
Kevin Tougeron
Suzanne Tough
Omar Touhami
Chafia Touil-Boukoffa
Mahmoud Toulany
Maroua Tounsi
Nicolas Tournier
Olivier Tourny
Dayna Touron
Jason Tourville
Jonathan Touryan
Benoit Tousignant
Cécile Toussaint
Emmanuel Toussaint
Jean-François Toussaint
Loren Toussaint
Paule Toussaint
Pierre-Louis Toutain
Hazem Toutounji
Maria Touz
William Tov
Sulay A. Tovar
Armando Tovara
John Tower
Sherry Towers
Aloen Townsend
Dewayne Townsend
Jasmine Townsend
Jason M. Townsend
Kristy Townsend
Nathan Townsend
Steven Townsend
Tim G. Townshend
Michael Towsey
Flavio Toxvaerd
Kensuke Toyama
Hidenori Toyoda
Masao Toyoda
Shigeru Toyoda
Satoshi Toyokawa
Tomonobu Toyomasu
Yuka Toyoshima
Teruaki Tozaki
Mark Tozer
Jozsef Tozser
Alessandro Tozzi
Vincent Traag
Luigia Trabace
Daniela Trabattoni
Maret Traber
Edouard Trabulsi
Kevin Tracey
Howard Trachtman
Melissa Tracy
Adrian Traeger
Joanne Traeger-Synodinos
Sarah Tragesser
Brian Trainor
Ephraim Trakhtenberg
Annalisa Trama
Scott Trammell
Donatella Tramontano
Ben Tran
Bich Tran
Luan Tran
Minh-Ha Tran
Ngoc Lan Tran
Ngoc Tam Tran
Ngoc Tran
Nham Tran
Pamela Tran

Simon Tran
Thach Tran
Tuan Tran
Vuvi Tran
Guy Tran Van Nhieu
Patrick Tranel
Phan Trang
Steven Trangmar
Jean-François Trani
Joan Tranmer
Michael Tranter
Heather Trantham-Davidson
Constantin Tranulis
Isidore Tiandiogo Traore
Birna Trap
Andrew Trapp
Aaron Trask
Catherine Trask
Paul Tratnyek
Walter Traunspurger
Michael Traurig
Peter Trautman
Lydie Trautmann
Sebastian Trautmann
R. Alberto Travagli
Rui Travasso
Ana Travassos
Bruno Travassos
Ana Traven
Cheryl Travers
Colm Travers
Alberto Traverso
Carlos Travieso
Katherine Travis
Ilias Travlos
Zdeněk Trávníček
Sophie Trawalter
Jakub Trawiński
Nikki Traylor-Knowles
Vincent Traynelis
Richard Traystman
Marsha Treadwell
Jim Treasurer
Jonel Trebicka
Rowan Trebilco
Polonca Trebše
Arnaud Trebucq
Edward Tredget
Jay Tree
Yada Treesukosol
Nicole Trefault
Indi Trehan
Sandra Trehub
Emmanuel Treiner
Nathaniel Simon Treister
Vilna Treitler
Joanna Trelinska
Jacek Trelinski
Maria Trelis
Jacques Tremblay
Sara Tremblay
Sebastien Tremblay
Helen Tremlett
Marta Tremolada
Kevin Trenberth
Verena Trenkel
Luca Trentin
Marco Trerotola
Jesús Tresguerres
Odile Tresse
Christoph Treude
Florette Treurnicht
Km Treurniet
Analia Trevani
James Trevaskis
Janine Trevillyan
Kelly Trevino
Claudia Treviño
Andrea Trevisan
Chiara Trevisan
Erminio Trevisi
Shaun Treweek
Matteo Trezzi
Helen Triantafyllidi
Maria Triantaphyllou
Calvin Tribby
Rachel Tribe
Stefano Triberti

Paula Tribulo
Elena Tricarico
Timothy Tricas
Larissa Trierveiler-Pereira
Elena Trifiletti
Gianluca Trifirò
Oleg V. Trifonov
Vladimir Trifonov
Mark Trigg
Antonio Ivano Triggiani
Robert Trigiano
Jose Trigo
Jeff Trimarchi
Bruno Trimarco
Antonio Trincone
Ha Ngoc Trinh
Nghia Quoc Trinh
Thu Le Trinh
Erik Trinkaus
Vickery Trinkaus-Randall
Laura Trinkle-Mulcahy
Anubhav Tripathi
Deeksha Tripathi
Deepak Tripathi
Dharmendra Tripathi
Kaushlendra Tripathi
Kumud Tripathi
Madhulika Tripathi
Prateek Tripathi
Alok Tripathy
Amiya Kumar Tripathy
Chittu Tripathy
Devjit Tripathy
Mukta Tripathy
Shreejoy Tripathy
Sucheta Tripathy
Julien Tripette
Matthew Triplette
Marco Tripodi
Katie Tripp
Paul Trippier
Vincenzo Trischitta
Jordis Trischler
Vivian Tristão
Luigi Tritapepe
Tobias Tritschler
Drenka Trivanovic
Alpa Trivedi
Hargovind Trivedi
Niyati A. Trivedi
Maria Triviño
Jan Trmal
Margarita Trobos
Carolina Trochine
Mats Troedsson
Justin Trogdon
Lill Trogstad
Richard Troiano
Christian Troidl
Maria Trojano
Maria Trojanowska
Thomas Trolle
Ivani Trombetta
Cecile Tromeur
Gerard Tromp
Rodrigo Troncoso
Xoana Troncoso
Juan Ramon Troncoso-Pastoriza
Ulven Trond
Ana Tronholm
Natalie Tronson
Peter Trontelj
Esther Troost
Gareth Trope
Thomas Tropea
Robert Tropek
Marius Troseid
Daniel Trott
Darren Trott
Christopher Trotta
Vincenzo Trotta
Julie Trotter
Paula Trotter
Jocelyn Trottier
Lucy Troup
Amalia Trousson
Antonio Trovato
Fabio Trovato

Frank Trovato
Amy Trowbridge
Karen Troy
Ray Truant
Leora Trub
Oriana Trubiani
Thai Thanh Truc
Cecilia Trucchi
Emiliano Trucchi
Emanuele Trucco
Johannes Trück
Stefan Trück
Vance Trudeau
Claudia Trudel-Fitzgerald
Gabriel Trueba
Santiago Trueba
N. Seth Trueger
Heather True-Krob
Shaun Truelove
Lisbeth Truelstrup Hansen
Patrick Truffert
Allison Truhlar
Anjali Truitt
Andrea Trujillo
Carlos Trujillo
Cleber Trujillo
Madia Trujillo
Maria Trujillo
Nelson Trujillo-Barreto
Irina Trukhanova
Stephen Trumble
Stephen Trumbo
Ben Trump
Saskia Trump
Martin Trunec
Pavel Trunecka
Buu Chau Truong
Lisa Truong
Mandy Truong
Sandra Truong
Eugenia Trushina
George Truskey
Anita Truttmann
Roberto Truzoli
Piotr Tryjanowski
Krzysztof Trzciński
Clarissa Trzesniak
Kali Trzesniewski
Olga Tsachouridou
Konstantinos Tsagakis
Konstantinos Tsagarakis
I. Tsagaris
Anastasia Tsagkarakou
Landry Tsague
Chia-Fen Tsai
Chieh-Chih Tsai
Ching-Hui Tsai
Ching-Piao Tsai
Feng-Jen Tsai
Henghsiu Tsai
Henry Tsai
Hsin-Jen Tsai
Hui-Jen Tsai
I-Chun Tsai
Jang-Zern Tsai
Jaw-Ji Tsai
Jer Chia Tsai
Keng-Chang Tsai
Kenneth Tsai
Kun-Hsien Tsai
Kuo-Wang Tsai
Ming-Horng Tsai
Ming-Ju Tsai
Ming-Tsun Tsai
P. P. Tsai
Pei-Chien Tsai
Pei-Wei Tsai
Ray Tsai
Tzung-Yi Tsai
Vicky Tsai
Wei-Lun Tsai
Wen-Chan Tsai
Wen-Chieh Tsai
Yi-Chun Tsai
Yien Tsai
Yu Tsai
Yueh-Ting Tsai
Yu-Huan Tsai

Evangelos Tsakalos
Panagiotis Tsakanikas
Alkiviadis Tsamis
Athanasios Tsanas
Benjamin Tsang
Ling Ming Tsang
Wang Tsang-En
Eleni Tsantili
Anna Tsantili-Kakoulidou
Betty Tsao
Kuo-Chien Tsao
Nina Tsao
Pei-Chen Tsao
Tsu-Yu Tsao
Nicolas Tsapis
Chia Jung Tsay
James Tsay
Erwin Tschachler
Philipp Tschandl
Tim Tschaplinski
Sebastian Tschauner
Thomas Tschernig
Thomas Tscheulin
Walter Tschinkel
Naomi Tschirhart
Diogo Tschoeke
Gary Tse
Hubert Tse
Raymond Tse
Wai S. Tse
D. Tsegaye
Aster Tsegaye Abebe
Theodore Tsekeris
G. E. Tsekouras
Theodore Tselios
Nikolaos Tselis
Chih-Wei Tseng
Henry Tseng
Joseph Tseng
Kuo-Chih Tseng
Michelle Tseng
Po-Lin Tseng
Tai-Chung Tseng
Te-Ming Paul Tseng
Roumen Tsenov
Elias Tsepis
Stella Tsermentseli
Konstantin Tsetsarkin
George Tsiamis
Chryssa Tsiara
Athe Tsibris
Anne Tsicopoulos
Konstantinos Tsikopoulos
Karl Tsim
Maria Tsimidou
Ianthi Tsimpli
Panagiotis Tsiotras
Konstantinos Tsioutis
Aristotelis Tsirigos
Zisis Tsiropoulos
Georgios Tsivgoulis
Susan Tsivitse Arthur
Janice Tsoh
James Kit Hon Tsoi
Georgios Tsoktouridisa
Anthoula Tsolaki
Apostolos Tsolakis
Maria Tsoli
Michael Tsoory
James Tsoporis
Hsiao-Hui (Sophie) Tsou
Andrew Tsourkas
Akihito Tsubota
Takuya Tsuchihashi
Satoru Tsuchikawa
Atsunori Tsuchiya
Kiichiro Tsuchiya
Naotsugu Tsuchiya
Hiroko Tsuda
Leo Tsuda
Yoshiaki Tsuda
Tsuyoshi Tsuduki
Masataka Tsuge
Amy Tsui
Kin Ming Tsui
Takanobu Tsuihiji
Atsushi Tsuji
Satsuki Tsuji

Taishi Tsuji
Yamato Tsuji
Yoshihide Tsujimoto
Kayoko Tsujino
Hideo Tsukada
Tomohide Tsukahara
Kazunori Tsukuda
Kumiko Tsukui
Kouhei Tsumoto
Shizuka Tsuneki
M. Tsunematsu
Nobumitsu Tsunematsu
Chou Tsung-Han
Kanami Tsuno
Nobuo Tsuru
Jennifer Tsuruda
Masashi Tsuruta
Kazuhiko Tsuruya
Takumi Tsutaya
Rie Tsutsumi
Kiyomi Tsuyuki
Shinya Tsuzuki
Peter Tsvetkov
Olga Tsyusko
Bin Tu
Bo Tu
Cf Tu
Haicheng Tu
Huakang Tu
Ping Tu
Qichao Tu
Tsang-Wei Tu
Wei Tu
Yaping Tu
Jack Tubbs
Roberto Tuberosa
Tracy Tuberville
Debara Tucci
Tiziano Tuccinardi
Mihran Tuceryan
Allan Tucker
C. A. Tucker
Carole Tucker
Colin Tucker
Conrad Tucker
Don Tucker
Robin Tucker
Sue Tucker
Robert Tuckey
Barbara Tudek
Maria Tudela Isanta
Francesco Tudisco
Carrie Tudor
Ana Tudoran
Zbynek Tudos
Eva Tudurí
Bettina Tudzynski
Catalin Tufanaru
Cristina Tufarelli
Ty Tuff
Pierre Tuffery
Can Tugcu
Eimer Tuite
Mick Tuite
Markku Tukiainen
Anil Tuladhar
Izabela Tuleta
Catherine Tuleu
Hardeep Singh Tuli
Fabricio Tulini
Shripad Tuljapurkar
Paul Tulkens
Jennifer Tullet
Francesca Tulli
Jonathan Tullis
Heather Tulloch
Marshall Tulloch-Reid
Mark Tully
Phillip Tully
Madalina Tuluc
Hayrettin Tumani
Dmitry Tumin
Serdar Tumkor
Luca Tummolini
Pürhan Barbaros Tuncer
Rosa Tundis
Chun-Wei Tung
Keith T. S. Tung

Tao-Hsin Tung
Tung-Hung Su
Gro Tunheim
James Tunnell
David J. Tunnicliffe
Hanna Maja Tunset
Tiinamaija Tuomi
Delphine Tuot
Srinivasan Tupal
Ali Turab
Rama Mohana R. Turaga
Angela Turalba
Belma Turan
Kadriye Turan
Dominique Turck
Ronald Turco
Lorraine Turcotte
Mazda Turel
Luca Turella
Christine Turenne
Nicolas Turenne
Emre Turer
Kürsad Turgay
Jean Turgeon
Nathalie Turgeon
Ali Turhan
Nicholas Turiano
Luca Turin
Adrián Turjanski
Dusan Turk
Kendra Turk
Tarek Turk
Teja Turk
Ismail Türkan
Metin Türkay
Barış Türkbey
Isik Turker
Kemal Turker
Onur Can Türker
Hasan Türkez
Sadok Turki
Thomas Turkington
Murat Turkoz
Meltem Türkyılmaz
Mustafa Turkyilmazoglu
Eva Turley
Ted Turlings
Akif Turna
Katarzyna Turnau
Colin Turnbull
Isaiah Turnbull
Jeremy Turnbull
Travis Turnbull
Andrew Turnell
Barbara Turner
Bradley Turner
Claire Turner
David Turner
Erin Turner
Harvey Turner
James Turner
Karly Turner
Lucy Turner
Monique Turner
Nancy Turner
Nigel Turner
Raymond Turner
Robin Turner
Russell Turner
Thomas Turner
Wendy Turner
Julie Turner-Cobb
Tiago Turnes
Xavier Turon
Konstantin K. Turoverov
Konstantin Turoverov
Tomasz Turowski
Andrew Turpin
Mario Turri-Zanoni
Ernest Turro
Francesca Turroni
Silvia Turroni
Ailie Turton
Bathsheba Turton
Mikko Turunen
Ossi Turunen
Anna Turusheva
Yerlan Turuspekov

James Turvill
Stuart Turville
Stefano Turzi
Víctor Manuel Tuset
Jack Tuszynski
John Tuthill
Tobias Tuthill
Joshua D. Tuttle
Antonino Tuttolomondo
Raymond Tutu
Anja Tuulonen
Lea Tuzovic
Asta Tvarijonaviciute
Anne Therese Tveter
Cedric Twardzik
Conor Tweed
Laurie Twells
Hannock Tweya
Maureen Twiddy
Gilad Twig
Homer Twigg
Marinka Twilt
Sally Twining
Jeffery Twiss
Craig Twist
Martin Twiste
Nikhil Tyagi
Wricha Tyagi
J. Tyberg
Anna Tyborowska
Erika Tyburski
Andrea Tyler
Anna Tyler
Damian Tyler
Anna Tylki-Szymanska
John Tyminski
Peter Tymms
Brian Tyo
Andrew Tyre
Joanna Tyrowicz
Christopher Tyrrell
Gregory Tyrrell
Ole-Bjørn Tysnes
Gregory Tyson
Reny Tyson
T. Tyson
Eric Tytell
Esa Tyystjärvi
Emmanuel Tzanakakis
Nikolaos Tzanakis
Evangelos Tzanatos
George Tzanetakis
Giorgos Tzanetakis
Ioannis Tzanetakis
Socrates Tzartos
Athanasia Tzelepi
Yi-Ting Tzen
Chi-Meng Tzeng
Tzong-Der Tzeng
Wen-Guey Tzeng
Vasilios Tzilas
Golfo Tzilos
Anastasios Tzingounis
Athina Tzinia
Orna Tzischinsky
Rumiana Tzoneva
Inna Tzoran
Shey-Cherng Tzou
Nathalie Tzourio-Mazoyer
Argyris Tzouvelekis
Yoshifumi Ubara
Paloma Ubeda
Douglas Ubelaker
John Ubels
Francesca Uberti
Darrell Ubick
Eunice Ubomba-Jaswa
Yurdanur Ucar
Ilaria Uccella
Mariano Ucchesu
Ines Magan Uceda
Nurcan Üçeyler
Evgenya Uchasova
Chiyoko Uchida
Hayato Uchida
K. Uchida
Kazuyuki Uchida
Keiko Uchida

Sae Uchida
Shizuka Uchida
Shunya Uchida
Takafumi Uchida
Thomas Uchida
Yoshikazu Uchida
Ken Uchino
Shigehiko Uchino
Ikuo Uchiyama
Mariko Uchiyama
Alfred Uchman
Taizo Uda
Nobuyuki Udagawa
Joshua Udall
A. C. Udayashankar
Arif Uddin
Imam Uddin
Jamal Uddin
Klas Udekwu
Sean Udow
Jayaram Udupa
Nayanabhirama Udupa
Andrew Udy
Pittawat Ueasangkomsate
Severin Uebbing
Uwe Ueberham
Atsushi Ueda
Kazuhiro Ueda
Kouhei Ueda
Kyosuke Ueda
Mitsuyoshi Ueda
Natsuo Ueda
Seiji Ueda
Shinichiro Ueda
Tadashi Ueda
Yukio Ueda
Christopher Uejio
Noriko Ueki
Hiroji Uemura
Mamoru Uemura
Masaki Ueno
Naoto Ueno
Shinji Ueno
Shinya Ueno
Takamasa Ueno
Takashi Ueno
Takayuki Ueno
Yoshihiro Uesawa
Yuzaburo Uetake
Akihito Uezato
Akiyoshi Uezumi
Rafael Ufret-Vincenty
Estefania Ugarte Berzal
Alberto Ugolini
Alessandro Ugolini
Carlos Ugrinowitsch
Olaf Uhl
Florian Uhle
Anne-Catrin Uhlemann
Peter Uhlhaas
Ondrej Uhlik
Eric Luis Uhlmann
Kumiko Ui-Tei
Jouni Uitto
Bernadett Ujhelyi
Beata Ujvari
Dorina Ujvari
Kazuyoshi Ukena
Osamu Ukimura
Dede Ukueberuwa
Kingsley Ukwaja
Roshani Ulak
Niels Uldbjerg
Neslihan Uler
Adam Ulfsbo
Monica Ulibarri
Alexandru Ulici
Salvatore Ulisse
Insha Ullah
Lena Uller
Sarah Ullevig
Buddy Ullman
Megan Ullman
Gerhild Ullmann
Ekkehard Ullner
J. Ulloa
Rita Ulloa
José Ulloa Fulgeri

Oliver Ullrich
Sophie Ullrich
Stephan Ulmer
Alexis Ulrich
Deborah Ulrich
Yvonne Ulrich-Lai
Ahmet Uludag
Safak Ulusoy
Chad Ulven
Peter Ulz
Sang-Won Um
Aleksandr Umanetc
Sadiq Umar
Colin Umeda
Hiroyuki Umegaki
George Umemoto
Naoyuki Umemoto
Toshitaka Umemura
M. Renée Umstattd Meyer
Noella Umulisa
Lale Umutlu
Belgin Unal
Hamiyet Unal
Elçin Ünal
Aynur Unalp-Arida
Philippe Unbc
David Underhill
Charles Underwood
Fiona Underwood
Jonathan Underwood
Martin Underwood
Tony Underwood
Monika Undorf
Jaime Undurraga
Chigozie Jesse Uneke
Andrea Ungar
Lyle Ungar
Christoph Ungemach
Dana Unger
Daniel Unger
Elizabeth Unger
Erica Unger
Holger Unger
Jakob Unger
Jennifer Unger
Juliane Unger
Keith Unger
Kristian Unger
Tamar Unger
Thomas Unger
Rodolfo Ungerfeld
Leanne Unicomb
Northeast University
Steffen Unkel
Christian Unkelbach
Ercan Ünlü
Peter Unmack
Kathryn Unruh
Costin Untaroiu
Eva Untersmayr
Sathya Unudurthi
David Unwin
Abhinav Upadhyay
Ashish Upadhyay
Gaurav Upadhyay
Meera Upadhyay
Santosh Upadhyay
Swati Upadhyay
Ushma Upadhyay
Vaibhav Upadhyay
John Updegraff
John Upham
Melissa Upjohn
Raul Uppot
Priya Uppuluri
Dominic Upton
Aqeel Ur Rehman
Yuji Urabe
Lianne Urada
Shimpei Uraguchi
Ani Ural
Norifumi Urao
Karen Uray
Kevin Urayama
Shiro Urayama
Alexander Urban
Daniel Urban
Jill Urban

Julie Urban
Thierry Urban
Renata Urban-Chmiel
Henryk Urbanczyk
Zofia Urbanczyk-Lipkowska
Jacek Urbanek
Konrad Urbanek
Petr Urbanek
Camilla Urbaniak
Michael Urbaniak
Ferdinando Urbano
Rosa Urbanos Garrido
Ewa Urbanska
Krystyna Urbanska
Ralph Urbatzka
Julio Urbina
Elena Urcelay
Gonzalo Urcelay
Bruce Urch
Silvio Urcuqui-Inchima
Henrik Urdal
Aykut Üren
Pablo Ureña-Torres
Silvan Urfer
Oğuzhan Urhan
Iker Uriarte
Flavio Uribe
Phillip Uribe
Armando Uribe-Rivera
Kenneth Urish
Angelika Url
Vlada Urlacher
Oleg Urminsky
Mira Aničić Urošević
Donna Urquhart
Erin Urquhart
Mauricio Urquiza
L. Urrego
Elena Urrestarazu
Julio Urrets-Zavalía
Robert Ursano
Kristine L. Urschel
Sylvain Ursenbacher
Francesco Ursini
Raquel Urtasun
Mark Urtel
Yuksel Urun
Paweł Uruski
Olivier Urwyler
Javier Urzay
Ulises Urzua
Marija Ušćumlić
Sergio Useche
Jakob Usemann
James Usherwood
Takahiro Ushida
Emi Ushigome
Koichiro Ushijima
Atushi Ushimaru
Takashi Ushimaru
Masayuki Ushio
Dinara Usmanova
David Ussery
Jane M. Ussher
Dmytro Ustianenko
Kentaro Usuda
Federico Usuelli
Vera Usuelli
Chie Usui
Emiko Usui
Kengo Usui
Tomoko Usui
T. Utescher
Tor Utheim
Maria Uther
Olalekan Uthman
Thorsten Uthmeier
Sevuk Utkan
Jim Uttley
Ingo Uttner
Sagar Utturkar
Marcel Utz
Matti Uusitupa
Vladimir Uversky
Miguel Uyaguari
Shin-Ichi Uye
Linda Uyeda
Y. Uyeno

Sahra Uygun
David Uystepruyst
Francisco Uzal
Orit Uziel
Ashraf Uzma
Chigozie Uzoh
Ezeonwu Uzoma
Alper Uzun
Natallia Uzunbajakava
Esen Uzuntiryaki Kondakci
Michele Vacca
Mentore Vaccari
Monica Vaccari
Gustavo Vaccaro
Maria Vaccaro
Alberto Vaccheri
Tushar Vachharajani
Vidula Vachharajani
Mary Vachon
Daniel Vader
Rajanikanth Vadigepalli
Maya Vadiveloo
Ratna Vadlamudi
Viral Vadwai
Viveka Vadyvaloo
Henrik Vægter
Nahid Vaez
Leila Vaezazizi
Reza Vafashoar
Sina Vafi
Dimitrios Vagenas
Panagiotis Vagenas
Marie Vagner
Hamed Vahabi
B. Vahdani
Mayank Vahia
Wilfried Vahjen
Emil Vahtera
Stefania Vai
R. K. Vaid
Achala Vaidya
Varun Vaidya
Sriram Vaidyanathan
Subramanian Vaidyanathan
Mika Vaihekoski
Anu Vaikkinen
Daniel Vail
David Vail
Daniel Vaiman
Anssi Vainikka
Seppo Vainio
Baruch Vainshelboim
Edi Vaisbuch
Ulka Vaishampayan
Shipra Vaishnava
Tiziana Vaisitti
Iosif Vaisman
Mojtaba Vaismoradi
Tyler Vaivada
Sandor Vajda
Peter Vajkoczy
Madhu Vajpeyi
Vik Vakharia
Alexander Vakhitov
Erik Vakil
Nikolaos Vakirlis
Andrew Vakulin
Stéphanie Val
Faramarz Valafar
Emily Vala-Haynes
Rea Valaperta
Mary Valcanis
David Valcarcel
Silvana Valdebenito
Pawel Valde-Nowak
Miguel Valderrábano
Mario Valderrama
Augusto Valderrama-Aguirre
Juan Valdés
R. Valdes-Ramos
Gregorio Valdez
Laura Valdez
Ruby Valdez-Ojeda
Leonardo Valdivi
Abel Valdivia
Raphael Valdivia
Sylviane Valdois
Filipa Vale

Petterson Vale
Neena Valecha
Claudia Valeggia
Arnubio Valencia
Braulio Valencia
Salome Valencia
Rafael Valencia-Quintana
Peter Valent
Patrícia Valentão
André Valente
Carina Valente
Eduardo Valente
Marco Valente
Marialuisa Valente
Pablo Valente
Cesare Valenti
Davide Valenti
Luca Valenti
Maria Teresa Valenti
Vitor Valenti
Webers Valentijn
Ana Valentim
Marcel Valentim da Silva
Stephanie Valentin
Thomas Valentin
Aljosa Valentincic
Catherine Valentine
Marla Valentine
Rudy Valentine
Vincent Valentine
Elia Valentini
Rita Valentino
Peter Valentin-Weigand
Dario Valenzano
Alejandro Valenzuela
C. Fernando Valenzuela
Manuela Valenzuela
Michael Valenzuela
Olivia Valenzuela
Rodrigo Valenzuela
Andrés Valenzuela-Sanchez
Antonio Valera
Fabiana Valera
Michael Valerius
Antonio Valero
Julien Valette
Birgit Valhberg
Francisco J. Valiente-Soriano
Alireza Valizadeh
Priit Väljamäe
Emmanuel Valjent
Sofie Valk
Orsolya Valko
Jari Valkonen
Maria Valkonen
Bruce Vallance
Denise Valle
Javier Valle
Manuel C. Vallejo
Daniel Vallero
Soraya Valles
Antonino Vallesi
Isabelle Vallet-Gely
Richard Vallett
Maria Beatrice Valli
Helen Vallianatos
Natalia Vallianou
Marie Vallier
Justin Valliere
Giovanni Vallini
Lori Ann Vallis
Lothar Vallon
Olivier Vallon
Daniela Vallone
Giorgio Vallortigara
Jaume Valls
Marc Valls
Valerie Valls-Bertault
Ravi Valluru
Anna Valros
Matteo Valsecchi
Mia Valtonen
Miguel Valvano
Jose Valverde
Rodrigo Valverde
Sergi Valverde
Cornelis van Achterberg
Rebecca Van Acker

Tom van Agtmael
Benoit van Aken
N. van Alfen
Benjamin G. Van Allen
Mike Van Amburgh
Tinde van Andel
Richard van Arkel
Thea van Asselt
Marcel van Assen
Sander van Assen
Freekje van Asten
Sunshine Van Bael
Femke van Beek
Mireille van Beekvelt
Hetty van Beers
Jim van Belzen
Paul van Bergen en Henegouwen
Elisabeth Van Beveren
Wim Van Biesen
Koen Van Bisean
Ad van Bodegraven
Steven Van Borm
Wim Van Bortel
Kevin Van Bortle
Job van Boven
Anton van Boxtel
Eric van Breda
Shane Van Breda
Cornelis van Breemen
Ariena van Bruggen
Daan Van Brusselen
Jaap van Buul
Leen Van Campenhout
Fabien Van Coppenolle
Amaryllis Van Craenenbroeck
Reinout van Crevel
Steven Van Cruchten
Jeroen van Cutsem
Douglas Van Daele
P. Van Daele
Andrea van Dam
Rob van Dam
Sipko van Dam
Inge Van Damme
Mark Van de Casteele
Marie-Cecile van de lavoir
Anne-Els van de Logt
Yohan van de Looij
Pierre-Francois Van de Moortele
Fredrik Van de Steen
Dominique Van de Velde
Anne van de Ven
Fons Van De Vijver
Julie Van de Vyver
Dedmer Van de Waal
Gerlinde Van de Walle
Joanie Van de Walle
Thomas Van de Water
Angela Van de Wouw
Johannes van Delden
Emile van den akker
Elske Van den Akker-van Marle
Marius van den Beek
Bart van den Bemt
Hugo Van den Berg
J. W. van den Berg
Joost van den Berg
Ronald van den Berg
Thomas van den Berg
Timo van den Berg
Hubert van den Bergh
Mattis van den Bergh
Menno van den Bergh
Peter Van den Besselaar
Tobi Van den Bossche
Jan van den Brand
Marianne van den Bree
Kris Van den Broeck
Emanuel Van den broeke
Sander van den Burg
Ronald Van Den Bussche
Dawie van den Heever
Diana van den Heuvel
Joop van den Heuvel
Leigh van den Heuvel
Lotus van den Hoogen
Wilbert van den Hout
Andrew van den Hurk

Anne van den Nouweland
Philippe Van den Steen
Jan Van den Stock
Roland van den Tillaar
Ignatia Van den Veyver
Gert Van der Auwera
Johanna van der Bom
Erik Van der Burg
Willem Van der Does
Jerine Van Der Eijk
Arie van der Ende
Martin van der Esch
Markus van der Giet
Brandon Van Der Heide
Yuri van der Heijden
Floor van der Hilst
Antoine van der Hoeven
Marinka van der Hoeven
Nina van der Hoeven
Justin Van Der Hooft
Anouk van der Hoorn
G. van der Horst
Mathieu van der Jagt
Mart van der Kam
Saskia van der Kam
Katinka van der Kooij
Lauren Van Der Kraak
Peter van der Kraan
Anke van der Kwaak
Theodorus van der Kwast
Jeroen van der Laak
Janneke van der Laan
Robin van der Lee
Just van der Linde
M. Christien Van Der Linden
Mark van der Linden
Sander van der Linden
Koen van der Maaden
Lia Van der Maas
Jan Van der Made
Nathalie van der Mee-Marquet
Felix Van der Meer
Jaap Van der Meer
Peter van der Meer
Jorista van der Merwe
Sarel van der Merwe
Jan van der Ploeg
Barbara Van Der Pol
Jan van der Scheer
Yvonne van der Schouw
Sophie van der Sluis
Wouter van der Sluis
Jenny van der Steen
Jozef (Sjef) van der Steen
Ariane van der Straten
Daan Van der Veen
Alike van der Velden
Sanne van der Ven
Peter van der Voort
Erhard van der Vries
Philip Van der Wees
F. Van der Weijden
Aj van der Wekken
Christian van der Welf
Tjip van der Werf
Henk van der Werff
Peter Van der Windt
Kim van der Zwaluw
Peter van der Zwan
Mikael van Deurs
Margaretha van Dijk
Marie van Dijk
Lotte van Dillen
Jenneke van Ditzhuijzen
Jan van Dixhoorn
Anne van Dongen
Thijs van Dongen
Sander van Doorn
Christa Van Dort
Alwin van Drongelen
Cornelis van Drunen
D. van Duin
Eelco van Duinkerken
Leonie van Duivenvoorde
Bram Van Dun
Therese Van Durme
Zoé van Dyck
Thomas Van Dyke

Raymond van Ee
Laura Van Eerd
Frank van Eerdenburg
Casper van Eijck
Annemieke van Eijk
Ronald van Eijk
Evelien Van Ekert
Thomas van Elsen
Wouter H van Geffen
Russell Van Gelder
Frederik van Gemert
Martin J. C. van Gemert
M. van Genderen
Marit van Gils
Joost van Ginkel
Bethany Van Guelpen
Gary Van Guilder
Robbert W. van Hamersvelt
Michelle Van Handel
Wim van Harten
Karen van Hedel
Stephen Van Hedger
Loek van Heerebeek
Vincent van Hees
Dirk F. van Helden
Paul van Helden
Nick Van Helleputte
Mieke Van Hemelrijck
Vincent Van Heuven
Alain Van Hiel
Jo Van Hoof
Pim van Hooft
S. Van Hooft
Linda Van Horn
Marion Van Horn
Rob Van Houdt
Denise van Hout
Bennett Van Houten
Sabine Van Huffel
Marc Van Hulle
Eric van Hullebusch
Sven van Ijzendoorn
Erik Van Iterson
Luc Van Kaer
Irene van Kamp
Jo Ann Van Kessel
Michiels van Kessenich Laurens
John van Kesteren
Ronald van Kesteren
Geert van Kollenburg
Cees van Kooten
Arianne van Koppen
Richard van Kranenburg
Arno van Kuijk
Jacques van Lankveld
Edwin van Leeuwen
Frank van Leeuwen
Hans van Leeuwen
Matthijs van Leeuwen
Theo van Leeuwen
James Van Leuven
Hein van Lith
Anita van Loenhoud
Geert van Loo
Andy Van Loocke
Barbara van Loon
Bram Van Loon
Kris Van Looy
P. van Luijk
Nico van Meeteren
Laurence Van Melderen
Tessa van Middelaar
Clare Van Miert
Margo van Mol
Sara Van Mossevelde
A. Van Muylem
Trung Van Nguyen
Steven Van Nocker
Vera van Noort
An Van Nuffel
Monique van Oers
Josefien van Olmen
Pim Van Ooij
Sandra van Oostrom
Arjen van Ooyen
Filip Van Opstal
Frits van Osch
Hans Van Ososterwyck

Martin van Overveld
Pieter van Paassen
Willem Van Panhuis
Ans van Pelt
Tom van Raaij
Lisa van Raalte
Anthony van Raan
Terry Van Raay
Capucine Van Rechem
Nick Van Reet
Joram van Rheede
Ildiko Van Rhijn
Annelies Van Rie
Ronald Van Rij
Piet van Rijn
Wj van Rooij
Mark van Rossum
Dimitri Van Ryckeghem
Bernadette Van Ryssen
Robert Van Saun
Erin van Schaik
Paul Van Schaik
Constant van Schayck
Onno van Schayck
Kim van Schooten
Alper van Sijl
Douwe van Sinderen
Sybrand van Sittert
Esther van Sluijs
Maarten van Smeden
Maren Van Son
Ann van Soom
Nina van Sorge
Bregje Van Spijker
Anneloes van Staa
Marie-Anne van Stam
John Van Stan II
Sigrid van Steen
Barbara Van Straaten
Arco Van Strien
Lyn Van Swol
Thomas van 't Erve
Anna van 't Veer
Katrien Van Tichelen
Lonneke van Tuijl
Lex van Velsen
Loes van Velthuysen
Susanne van Veluw
Wesley Van Voorhis
Jeroen van Vugt
Maaike van Vugt
Marieke van Vugt
Jeroen van Waarde
Robert van Waardenburg
David Van Wagoner
Corne van Walbeek
Chris Van Weel
Mirjam van Weissenbruch
Madelon van Wely
Cornelis van Werkhoven
Gilles van Wezel
Herman van Wietmarschen
Maarten van Wijhe
Charles van Wijk
Phillippe Van Wilder
Marijn van Wingerden
Robert van Woesik
Frans van Workum
Nelleke van Wouwe
Andoret van Wyk
Rosan van Zoest
Wieske van Zoest
Alissa Van Zutphen
Jelle van Zweden
Paul van Zwieten
Gert van Zyl
Suzanne van Gils
Maddi Vanaja
Abi Vanak
Perumal Vanamail
Jairam Vanamala
A. Vanbergen
Carrie Vance
David Vance
Jan Vanco
Marie Vancová
Mark VanDam
Peter Vandamme

Bolormaa Vandanmagsar
Christine Vande Velde
Greetje Vande Velde
Ina Vandebroek
Marie Vandecasteele
Wim Vanden Berghe
Heidi Vanden Brink
Carol Vandenberg
Bram Vandenbergh
David Vandenbergh
Stefaan Vandenberghe
Loren Vandenbroucke
Emilie Vander Haar
Christy Vander Jagt
Craig Vander Kooi
Marc Vander Linden
Vincent Vander Poorten
Stephen Vander Wall
Jolijn Vanderauwera
Dieter Vanderelst
Veronique VanderHorst
Leigh Vanderloo
John Vandermeer
Maryse Vanderplanck
Rodney Vanderploeg
Herve Vanderschuren
Nele Vandersickel
Hayo VanderWerf
Willem Vandeveen
Gilles Vandewalle
Jennifer Vandooren
Katrien Vandoorne
Tushna Vandrevala
Moriel Vandsburger
Wim Vanduffel
Stefanie VanDuine
Nicola Vanello
Odelu Vanga
Rohini Vanga
Siri Vangen
Jürgen Vangeyte
Guido Vanham
Paul Vanhoutte
Diana Vania
Nicholas VanKuren
Michael Vanlandewijck
Dana Vanlandingham
Veerle Vanlerberghe
Eric Vanman
Eugeen Vanmechelen
Marc Vanmeerbeek
Mich Vann
Willie Vann
Elizabeth Vanner
Jennifer Vannest
Joel Vanneste
Ester Vanni
Kirsten Vannice
Edouard Vannier
Jean Vannier
Michael Vannier
Irene Vänninen
Betteke Van-Noort
Steven Vannoy
Jeroen Vanoirbeek
Carlos Vanoye
Christophe Vanpouille
Wouter Vansteelant
Maarten Vansteenkiste
Arie Van't Spijker
Tim Vantilborgh
Vincent Vantrepotte
M. C. Vantyghem
Tavé VanZyl
Dolors Vaqué
Humberto Vaquera Huerta
Manuel Vaquero
Kulandaiappan Varadaraj
Karthik Mahesh Varadarajan
Kartik Varadarajan
Sudhahar Varadarajan
Pritish Varadwaj
Francis Varaine
Pietro Varaldo
Fabiana Varallo
Sooryanarayana Varambally
M. Varcamonti
Konstantinos Vardakas

Ricardo Vardasca
Bojjam V. Vardhini
Andrew Vardy
Emilio Varea
Marjolaine Vareille-Delarbre
Manuel Varela
Ruben Varela
Sara Varela
Andrea Varela-Stokes
Andrea Varella
Cristina Varese
Orsolya Varga
Tamas Varga
Zoltan Varga
Zsuzsanna Varga
Ignacio T. Vargas
Oscar Vargas
Carmen Vargas Garcia
Einar Vargas-Bello
Diego Vargas-Inchaustegui
María I. Vargas-Rojas
Neil Vargesson
Beena Varghese
Markku Varjosalo
Tracey Varker
Nissi Varki
Matthew Varley
Dileep Varma
Rohit Varma
Sashank Varma
Kambiz Varmira
Aniko Varnai
Michael Varner
Onur Varol
Serena Varotto
Øystein Varpe
Antonio Varriale
Bruno Varriale
Luisa Varriale
A. Varrone
Cristiano Varrone
Liana Varrone
Arvind Varsani
Gaurav Varshney
Raunak Varshney
Jaleh Varshosaz
Lenny R. Vartanian
Maria Vartiainen
Sara Varughese
Athanasia Varvaresou
Prashanth Vas
Frantisek Vasa
Lilian Vasaitis
Archana Vasanthakumar
Gabor Vasarhelyi
A. Vasas
Daniel Vasconcelos
Heraldo Vasconcelos
Maria José Vasconcelos
Raquel Vasconcelos
Sonia Vasconcelos
Daniel Vasconcelos-Santos
Marmar Vaseghi
Vipin M. Vashishtha
Vishal Vashistha
Samuel Vasikaran
Konstantina Vasileiou
Oleg Vasilenko
Vidanka Vasilevski
Maroula Vasilopoulou
Nikolay Vasilyev
Shravan Vasishth
Bruno Vaslin
Gustavo Vasques
Carlos Vasquez-Salinas
Massimo Vassalli
R. Vassallo
Suzane Vassallo
Eliana Vassena
R. Vassena
Nikon Vassilakos
Andon Vassilev
Dimitrios Vassilopoulos
James Vasslides
Evangelos Vassos
Sebastiaan Vastert
Ramesh Vasudeva
Sugumar Vasudevan

Marianne Vater
Vatsalya Vatsalya
Matteo Vatta
Akhila Vattekatte
Lars Vatten
Thomas Vatter
Anna Vaudano
Gaetano Vaudo
Emmanuelle Vaudour
David Vaudry
Brett Vaughan
Catherine Vaughan
Neil Vaughan
Owen Vaughan
Roger Vaughan
Amy Vaughan Van Hecke
Dominique Vautier
David Vaux
David Vauzour
Divya Teja Vavilala
Eric Vaz
Marco Vaz
Nuno Vaz
Pedro Vaz
António Vaz Carneiro
Almira Vazdarjanova
Nosratola Vaziri
Nadia Vazirpanah
Alberto Vazquez
Enrique Seoane Vazquez
Francisco Vazquez
Patricia Vazquez
R. Vazquez Guillamet
Carlos Arturo Vázquez-Chacón
Rafael Vazquez-Duhalt
Riccardo Vázquez-Juárez
Peter Vďačný
Jaimie Veale
Ashley Veatch
Maren Veatch-Blohm
Kari Veblen
Carlo La Vecchia
Nerina Vecchio
Carmine Vecchione
Raffaele Vecchione
Ivan Vechetti
Miklós Vecsernyés
Srinivasan Vedantham
Rajesh Vedanthan
Alicia Vedio
Gabriela Vedovato
Thia Vedovatto
Silvia Vedovello
S. Swaroop Vedula
Roland Veelken
Marjolein Veenendaal
Jan Veenstra
Janne Veerbeek
J. Lennert Veerman
Almudena Vega
Fidel Vega
Tomás Vega
Didier A. Vega-Oliveros
Annemarie Vegh
Viktor Vegh
Gianluigi Veglia
Sigrun Vehling
Anne Vehlow
Nélio Veiga
Juha Veijola
Kristof Veitschegger
Eric Vejerano
Armands Veksins
David Vela-Corcía
Frederic Velard
Enriqueta Velarde
Cruz Velasco
Ines Velasco
Miguel Velasco
Javier Velasco-Vazquez
Nelson Velasquez
Gustavo Velásquez
Thirumalaisamy Velavan
Selvaraj Velayutham
Miguel Velazquez
Omaida Velazquez
Norma Velázquez-Guadarrama
Jet Veldhuijzen van Zanten

Edwin Veldhuizen
Rudolf Veldhuizen
Scott Veldhuizen
Christopher Veldkamp
Hans Veldkamp
Marieke Veldkamp
Menno Veldman
Sanne Veldman
Adam Vele
Ivan Velez
María Vélez
Carlos Velez-Pardo
Sergia Velho
Violeta Velikova
Brigitte Velkeniers-Hoebanckx
Elena Velkoska
Patricia Vella Bonanno
Sukumar Vellakkal
Pablo Vellando
Kumutha Vellasamy
Marley M. B. R. Vellasco
Licio Velloso
Guillermo Velo-Anton
A. C. M. Veloo
Adriano Veloso
Artur Veloso
Craig Velozo
Kiran Kumar Velpula
Sadanandan Velu
Vijayakumar Velu
Sumithra Velupillai
Naga Vemprala
Lakshmi Vemu
Emilie Venables
Patrick Venail
Miguel Vences
Sofia Venceslau
Márton Venczel
Françoise Vendittelli
Charles Venditti
Mario Venditti
Venkata Pulla Rao Vendra
Debora Vendramini-Costa
Marc Vendrell
Juan Venegas
Erik Veneklaas
Monica Venere
Tiziana Venesio
Fabienne Venet
Christos Venetis
Jonathan Venezia
Alessio Veneziano
Vincenzo Veneziano
Viktoria Venglovecz
Paola Venier
Domenica Veniero
Sreedhar Venkannagari
Arvind Venkat
Naga Venkata Pothineni
Archana Venkataraman
Vivek Venkataraman
Manjunatha Venkataswamy
G. Venkatesan
Byrappa Venkatesh
Pradeep Venkatesh
Sudhakar Venkatesh
Neeraja Venkateswaran
Divi Venkateswarlu
Anand Venkatraman
Vishwanath Venketaraman
Katja Venko
Giovanni Vennarecci
Conchita Vens
Amanda Venta
Andre Venter
Estelle Venter
Gert Venter
Hermoine Venter
Marietjie Venter
Mauritz Venter
Peter Ventevogel
Maximo Vento
Iván Ventoso
Kathleen Ventre
Beth Ventura
Daniele Ventura
Francesc Ventura
Hector Ventura

Rossella Ventura
Samuel Ventura
Sebastian Ventura
Stephen Ventura
Carlos Ventura Bort
Martin Venturas
Venessa Venturi
Charles Venuto
Henna Vepsalainen
Antonio Vera
Jelle Veraart
Francisco Vera-Badillo
Lucio Vera-Cabrera
Jw Verano
Michelle Verant
John Veranth
Lucas Verardo
Chiara Verbano
Jef Verbeek
Caroline Verbeke
Wouter Verbeke
Frank Verbraak
Stefaan Verbruggen
Jeanmarie Verchot
Fabrice Vercruyssen
Luciano Verdade
Nuria Verdaguer
Pasquale Verde
Luis Verde Arregoitia
Ivan Verdenik
Ashton Verdery
Julien Verdier
Megan Verdon
Irma Verdonck-de Leeuw
José Verdú
Elvira Verduci
Balazs Veres
György Veress
Daniela Vergara
Giuseppe Vergaro
Pierre Verger
Shilpi Verghese
Nikhil Vergis
Anna Vergmann
T. Vergne
Nathalie Vergnolle
Olivia Vergnolle
Remi Vergnon
Cristobal Vergudo
Andrey Vergun
Annette Vergunst
Frank Verhaegen
Nick Verhaeghe
A. P. Verhagen
François Verheggen
Michel Verheij
G. Verheyden
An Verheyen
Hans Verhoef
Corine Verhoeven
David Verhoeven
Koen Verhoeven
Els Verhoeyen
Chris Verhofstede
Niels Verhulst
António Veríssimo
Georges Verjans
Sergio Verjovski-Almeida
Erwin Verkade
Alexey Verkhovtsev
Pavan Verkicharla
Alan Verkman
Peter Verkoeijen
Heleen Verlinden
Maite Verloigne
Eliseu Verly Jr.
Abhai Verma
Ajit Verma
Amit Verma
Anurag Verma
Digvijay Verma
Karun Verma
Mayank Verma
Mohit Verma
Naresh Kumar Verma
Pankaj Verma
Raj Verma
Rajeev Verma

Rajesh Kumar Verma
Sandeep Verma
Subhash Verma
Surbhi Verma
Suresh K. Verma
Pierre Vermeersch
Johannes Vermehren
Mark Vermeij
Monica Vermeulen
Wim Vermeulen
Christine Verna
Pietro Vernazza
Don Vernekohl
Marine Vernet
Lawrence Vernetti
Laurent Vernhet
Gianluca Vernillo
Andy Vernon
Hilary Vernon
Mieke Vernooij
Robin W. M. Vernooij
Eve Veromann
Dana Veron
Nicola Veronese
Fabio Veronesi
Giulia Veronesi
Camila Veronez
Brixner Veronika
Pierfrancesco Veroux
B. Verplanken
Franck Verrecchia
Fellippo Verri
Diarmuid Verrier
Charlotte Verroken
Elise Verron
Paolo Versacci
Elisabetta Versace
Erik Verschueren
Thomas A. Verschut
Chris Verslype
Marjan Versnel
Eric Verspoor
Karin Verspoor
M. G. Vervloet
Bart Verwaaijen
Niek Verweij
Willem Verwey
Eleni Verykouki
Robert A. Veselis
Petr Veselý
Michael Vesia
Peter Vesk
Zachary Vesoulis
Cordula Vesper
Torbjorn Vestberg
Jan Vester
Robert Veszteg
Davide Liborio Vetrano
Mario Vetrano
Brooke Vetter
Celine Vetter
Irina Vetter
Lael Vetter
Enzo Vettorato
Martina Vettoretti
Vaclav Vetvicka
Paolo Vezza
Giuseppe Vezzoli
Esther Via
Anouck Viain
Flavie Vial
François Vialard
Pierluigi Viale
Annie Viallat
Taryn Vian
Duarte Viana
Jose Marcelo Viana
Mafalda Viana
María Teresa Viana
Alberto Vianello
Fernanda Vianna
Angela Maria Vianna Morgante
Diego Viasus
Guillaume Viau
Muriel Viaud
Francesca Viazzi
Marcelo Ricardo Vicari
Carmelo M. Vicario

Antonio Vicent
Ana Carolina Vicente
C. Rachel Vicente
Cláudia Vicente
Isabel Vicente
João Vicente
Mata Silva Vicente
Renato Vicente
Ruben Vicente
James Vickers
Lance Vickers
Neil Vickers
Laurence Vico
Michele Vicovaro
Tamás Vicsek
Benjamin Victor
Christina Victor
John Victor
Sarah Victor
Victor Victor
Cesar Victora
András Viczián
Benedicto Vidal
Cecilio Vidal
Daniel Vidal
Lital Vidal
Manuel Vidal
Pierre-Paul Vidal
Roberto Vidal
Silvia Vidal
Stefan Vidal
Xavier Vidal
Pierre-Olivier Vidalain
Emmanuelle Vidal-Petiot
Manuel Vidal-Sanz
Carmen Vidaurre
Romeu Videira
Luiz Videla
Elizabeth Videlock
Vibeke Videm
Antonello Vidiri
Marianne Vidler
Pietro Vidossich
Jennifer Vidrine
Meghan Vidt
Dharmapuri Vidyasagar
Henri Vié
Monica Viegas Andrade
Giovanni Viegi
Vincent Vieillard
Angelica Vieira
Armando Vieira
Bruna Vieira
Dorice Vieira
Filipe Vieira
Gregory Vieira
Leda Vieira
Luiz Henrique Vieira
Margarida Vieira
Matias Vieira
Monica Vieira
Rodolfo Vieira
Taian Vieira
Ana Catarina Vieira de Castro
Jose Vieira Jr.
Victoria Vieira-Potter
Tania Viel
Jörg Vienken
Emilie Viennois
Maria Viga-De Alva
Paola Vigano
Davide Vigetti
Olga Vigiak
Giampaolo Vigilia
Elena Vigliar
Nora Vigliecca
Simona Viglio
Aurelia Viglione
Edward Vigmond
Luisella Vigna
Cécile Vignal
Luca Vignatelli
Ramachandran Vignesh
Philippe Vignon
Irene Vignon-Clementel
Daniel Vigo
Carlo Vigorito
Stafford Vigors

Bernard Vigué
C. Viguié
Kaido Viht
Ene Viiard
Jorma Viikari
Heli Viiri
Sanna Viitanen
Monika Vij
Shruti Vij
Kodati Vijayalakshmi
Mamannamana Vijayan
Mathilakath Vijayan
Mathilakth Vijayan
Shibu Vijayan
Vimalan Vijayaragavan
Srinivasan Vijayaraghavan
Kiruthiga Vijayaraman
Daniel Vijlbrief
Maria Viketoft
Prashant Vikram
Bjørn Egil Vikse
Sofie Viksten
Alejandro Vila
Xose Anton Vila
Ana Vila Concejo
Sibelle Vilaca
José Vilaça-Alves
Gemma Vilahur
Rk Vilaichone
Laura Vilander
Guillermo Vilanova
Manuel Vilanova
Ana Vila-Parrish
Francisco Vilaplana
César Vilar
Jose Vilar
Eduardo Vilar Gomez
Mireya Vilar-Compte
Raquel Vilar-López
Antoni Vilaseca
Marina Vilaseca
Diogo Vila-Viçosa
Juan J. Vilchez
Noa Vilchinsky
Andreas Vilcinskas
Bruno Vilela
Rytas Vilgalys
Lars Vilhelmsen
Albertus Viljoen
Laura Vilkaitė
Alessandro Villa
Federica Villa
Gianluca Villa
Paola Villa
Roberto Federico Villa
Pedro A. Villablanca
Luis Villada
Jose Villadangos
Javier Villafuerte-Gálvez
Emilio Villa-González
Rafael Villalobos-Molina
Fernando Villalta
Frederick Villamena
Aquilino Villamonte-Chevalier
Dan Villamor
Vincenzo Villanacci
Daniela Villani
Gabriela Vanina Villanova
Anastasio J. Villanueva
Augusta Villanueva
Elmer Villanueva
Sonia Villapol
Enrique Villar
José Augusto Villar
Bernardo Villareal-Ramos
Paolo Villari
Francisco Villarreal
Jose Villarreal-Barajas
Morris Villarroel
Sebastian Villasante
Paulina Villaseca Delano
Laura Villa-Torres
Cecilia Villaverde
Silvia Villaverde
Valentin Villaverde
Nathalie Villa-Vialaneix
Andrés Villegas
Juana Villegas

Victor Villegas
Julio Villena
Remi Villenave
Jandouwe Villinger
Peter Vilmos
Claudia Vilo
Alicia Viloria-Petit
Jean-Luc Vilotte
Antonella Vimercati
Catarina Vinagre
Jillian Vinall
Jordi Vinas
Miguel Viñas
Boris Vinatzer
Sumiti Vinayak
A. C. Vinayaka
Maj Vinberg
Christian Vincenot
Amy Vincent
C. Vincent
Florence Vincent
Grace Vincent
Isabel Vincent
Jean-Louis Vincent
Maxence Vincent
Neil J. Vincent
Stephen Vincent
Bruno Vincenzi
Antonio Vinciguerra
Manlio Vinciguerra
Paolo Vinciguerra
Patrick Vinck
Fabien Vinckier
Brigitte Vinçon-Leite
Aron Vincze
Éva Vincze
Sam Vine
Claudio Vinegoni
Paolo Vineis
Dragos Vinereanu
Tim Vines
Timothy Vines
Jonathan Vinet
Donald Vinh
Jan Vinjé
Cor Vink
Gerko Vink
Claudia Vinke
Peter Vinkler
Sarah Vinnicombe
K. Vinodhini
Francesco Viola
Manuela Viola
Salvatore Viola
Inês Violante
Nerissa Viola-Villegas
Jonathan Violin
Pooja Vir
Jitka Virag
Zsofia Viranyi
Satpal Virdee
Delia Virga
Massimiliano Virgilio
Pensabene Virginia
Plummer Virginia
Raimo Virkkala
Victor Virlogeux
Isabelle Virlogeux-Payant
Nitin Virmani
John Virostko
David Virshup
Akvilė Viršilė
Marianna Virtanen
Christiaan Vis
Heidy Visbal
Giovanni Viscogliosi
Alessia Visconti
Valerio Visconti
Jeane Visentainer
Roberto Visentin
Marcella Visentini
Karthik Vishwanath
Sneha Vishwanath
Karen Visick
Phillip Visintin
Scott Visovatti
Shelina Visram
Chris Visscher

Christian Visscher
Benoit Visseaux
Jenny Visser
Leo H. Visser
Theo Visser
Arjan Vissink
Joao Vissoci
Giulio Vistoli
Akila Visvanathan
Anand Viswanathan
Shiv Kumar Viswanathan
Vijay Viswanathan
Sandhya Visweswariah
Alessandro Vitale
Augusto Vitale
Carmine Vitale
Flavia Vitale
Luca Vitale
Vincenzo Vitale
Paolo Vitali
Libor Vitek
Santiago Viteri
Michael Vitevitch
Stanislav Vitha
U. Vitharana
Ben Vitiello
Peter Vitiello
Victoria Vitkova
Rui Vitorino
Rodrigo Vitório
Clement Vitrac
Ragini Vittal
Brigitte Vittrup
Dario Vitturi
Adele Vivacqua
Ana Vivas
Miguel Viveiros
E. Vivekanandan
Perumal Vivekanandan
Celine Vivien
Denis Vivien
Régis Vivien
Juan Antonio Vizcaino
Colin Vize
Margaret Vizzard
Dimitrios Vlachakis
Marilena Vlachou-Konstantinidou
Vladimir Vladimirov
Virginie Vlaeminck-Guillem
Johan W. S. Vlaeyen
Ross Vlahos
Petia Vlahovska
Mart Vlam
Alexander Vlantis
Joseph Vlasak
Marija Vlaski
Anastasia Vlasova
Harissios Vliagoftis
Quang Trung Vo
Christopher Vockley
Anthony Vodacek
Ulrich Voderholzer
Suman Vodnala
Christoph Voelter
Vincent Voelz
Terri Voepel-Lewis
Ellis Voerman
Cees Voesenek
Ilja Voets
Britta Vogel
Heiko Vogel
Ida Vogel
Jackie Vogel
Joseph Vogel
Jürgen Vogel
Lotte Vogel
Robert Vogel
Stephan Vogel
Thomas Vogel
Ulla Vogel
Rufin Vogels
Tim Vogels
Wolf-Dieter Vogl
Roberto Vogler
Josef Voglmeir
Andreas Vogt
Dawne Vogt
Klaus Vogt

Richard Vogt
Rolf Vogt
Stefan Vogt
Emily Vogtmann
Brian Vohnsen
Betty R. Vohr
Fahim Vohra
Triet Vo-Huu
Brian Voigt
Christian Voigt
Karen Voigt
Michael Voigt
Olivier Voinnet
Mathieu-Benoit Voisin
Pierre Voisine
Eberhard Voit
Renate Voit
Dina Vojinovic
Vladislav Volarevic
Gregor Volberg
Bastian Volbers
Petr Volf
Catherine Volgenant
Olena Volik
Ernest Volinn
Alex Volinsky
David Volk
Thomas Volken
Leentje Volker
Mirko Völkers
Brian Volkman
E. Volkmann
Anne-Nathalie Volkoff
Vadim Volkov
Svitlana Volkova
Victoriya Volkova
Benjamin Volland
L. Vøllestad
Nina Vøllestad
Linda Vollmer
Marcus Vollmer
Vitaly T. Volobouev
Ilya Volodin
Elena Volodina
Matias Volonte
Alexandra Voloshina
Elena Voloshina
Stefano Volpato
Umberto Volpe
Lays Volpini
Rildo Volpini
David Volz
Erik Volz
Jan vom Brocke
Dagny von Ahrens
Christian von Buchwald
Christoph von Castell
Noreen von Cramon-Taubadel
Yasmin von Dassow
Johannes Von den Hoff
Werner von der Ohe
Carol von Dohlen
Henrique von Gersdorff
Alexander von Gise
Stephan von Gunten
Stephanie von Hinke
Marina von Keyserlingk
Andreas von Knethen
Kathrein von Kopylow
Marie Von Lilienfeld-Toal
Mike von Massow
Rudolf von May
Veronika von Messling
Anna von Mikecz
Claire von Mollendorf
Clemens von Schacky
Malcolm von Schantz
Katherine von Stackelberg
Esther von Stebut
Christine von Toerne
Vinzenz von Tscharner
Sibylle von Vietinghoff
Eric von Wettberg
Thomas von Zglinicki
Constantin von zur Mühlen
Linda Vona-Davis
Alexander Vonbank
Tomas Vondra

Indira Vonhögen
Kimberly Vonnahme
John Vontas
Gerrit Voordouw
Maarten Voordouw
Maarten Voors
Kalpit Vora
Nirali Vora
H. Martin Vordermeier
I. Vorechovsky
Charles Vorhees
Barbara Vornam
Eugene Vorobeychik
Dimitri Voronine
Judit Vörös
Thomas Vorup-Jensen
Robin Vos
Alex Voskoboinik
Ayelet Voskoboynik
Soroush Vosoughi
Helen Vosper
Andreas Voss
Barbara Voss
Jameson Voss
Kenneth Voss
Logan Voss
Robert Voss
Stephen Randal Voss
Anna Vossenkämper
Alexander Vostroknutov
Mark Vosvick
Konstantinos Votis
Jan Votypka
Dimitris Voudouris
Athena Vouloumanos
Srinivas Vourganti
Georgia Vourli
George Vourlitis
Brynn Voy
James Voyvodic
Paul Voziyan
Jeffrey Vrabec
Paul Vrana
Bram Vrancken
Liesbet Vranken
Wim Vranken
Milka Vrecl
Thom Vreven
Chris Vriend
Jerry Vriend
Minka Vries
Frank Vriesekoop
Tomaz Vrtovec
Olga Vsevolozhskaya
Sonja Vucen
Aleksandra Vuckovic
Dajana Vuckovic
Ivan Vuckovic
Karen Vuckovic
Eric Vugrin
Isadora Vujcic
Maja Vujic
Tatjana Vujović
Milica Vukmirovic
Vanja Vukojevic
Nikola Vukovic
Olivera Vukovic
Matthew Vukovich
Mila Vulchanova
Tom Vulliamy
Tri Vuong
Heikki Vuorela
Raj Vuppalanchi
Loc Vu-Quoc
Dervis Vural
Beyza Vurusaner
Teraporn Vutyavanich
Dominique Vuvan
Saleha Banu Vuyyuri
Bellington Vwalika
Jatin Vyas
Seema Vyas
Veronika Vymetalkova
Dimitrios Vynios
Boris Vyskot
Eugene Vysotski
J. W.
Andrew Morehu Waa

Marit Waaseth
Dagmar Waberski
Lucy-Joy Wachira
Elisha Wachman
Rebecca Wachs Wachs
Mitchell Wachtel
Rachel Wachtenheim Jackson
Rolf Wachter
Margarethe Wacker
Sarah Wackerbarth
Werner Wackernagel
Hisashi Wada
Nikolas Wada
Takashi Wada
Takehiko Wada
Toshifumi Wada
Tabassum Wadasadawala
Kate Waddams
Simon Waddell
Jonathan Waddington
Alex Wade
Joseph Wade
Julia Wade
Michael Wade
Nick Wade
T. Joel Wade
Timothy Wade
Claes Wadelius
Mandheer Wadhwa
Antonia Wadley
Lyn Wadley
Danielle Wadsworth
Christian Waeber
Lynn Waelde
Hagen Waesche
Sharifah Wafa
Francis Wafula
Bart Wagemans
Alexander Wagenaar
Bradley Wagenaar
Brant Wagener
Elizabeth Wager
Adrian Wagg
Shailesh Waghmare
Mahendra Wagle
Eric Wagnac
Abram Wagner
Alan Wagner
Ana Wagner
Anjuli Wagner
Anne Wagner
Bradley Wagner
C. Wagner
Carol Wagner
Caroline Wagner
Carston Wagner
Dale Wagner
Denae Wagner
Diane Wagner
Eric Wagner
Henrik Wagner
Johanna Wagner
Josef Wagner
Lars M. Wagner
Lisa Wagner
Shannon Wagner
William Wagner
Zachary Wagner
Konrad Wagstyl
Saw Wah
Rizwan Wahab
M. Wahb-Allah
Nadia Waheed
Waqar Waheed
Khan A. Wahid
Md. Wahid
Rezwanul Wahid
Anna-Sophia Wahl
Niklas Wahlberg
Claes Wahlestedt
Thomas Wahli
Walter Wahli
Jens Wahlstrom
Joyce A. Wahr
Jennifer Wai
Israel Waichman
Khor Waiho
Vishwanath Waikhom

Stephen Wainer
Brandon Wainwright
Charlotte Wainwright
Derek Wainwright
Haruko Wainwright
Mark Wainwright
Peter Wainwright
Peter Wais
Dan Waisman
D. Wait
Catriona Waitt
John Waitumbi
Narendra Wajapeyee
Jose Roberto Wajman
Samuel Otabor Wajuihian
Kazuyuki Wakabayashi
Shigeo Wakabayashi
Ron Wakai
Yoshio Wakamatsu
Shigeharu Wakana
Nicole Wake
Juliet Wakefield
Thomas Wakefield
Joanna Wakefield-Scurr
Sonia Wakelin
Kevin Wakeman
Ajay Wakhloo
Hironori Waki
R. Wakili
Hiroaki Wakimoto
Shu Wakino
Peter Wakker
Joseph Wakshlag
Tomonori Waku
Keisuke Wakusawa
Salimah Walani
Lukasz Walasek
Christopher Walcek
Hedy S. Wald
George Waldbusser
Marcel D. Waldinger
Bruce Waldman
Ronald Waldman
Scott Waldman
Elisabeth Waldmann
Victor Waldmann
Albert Waldo
Matthew Waldor
Charles Waldren
Nathan Waldron
Nadine Waldschmitt
Anna Walduck
Etienne Waleckx
Marta Walentynowicz
Marie-Laure Walet-Balieu
Alicia Walf
Brian Walitt
Henry Walke
Alan Walker
Alexander Walker
Amy Walker
Ashley Walker
Berkley Walker
Candice Walker
David Walker
Edward Walker
Forbes Walker
Glenn Walker
Jemma Walker
John Walker
Kathleen Walker
Mark Walker
Michael Walker
Michelle Walker
Paul Walker
Robert Walker
Shawn Walker
Stephen Walker
Susan Walker
Susannah Walker
Timothy Walker
Trevor Walker
William Walker
Malcolm Walkinshaw
Benjamin Wall
Brian Wall
Emma Wall
Jonathan Wall

Kristin Wall
Michael Wall
Peter Wall
Rudolf Wall
Susan Wall
Aaron S. Wallace
Aaron Wallace
Bruce Wallace
Danielle Wallace
Darren Wallace
Deanna L. Wallace
Denise Wallace
Douglas Wallace
Edward Wallace
Emma Wallace
Jack Wallace
Kenneth Wallace
Nicholas Wallace
Ryan Wallace
Sarah Wallace
Vincent Wallace
Gwenyth Wallen
Anna Wallenbeck
Anna Waller
Bridget Waller
Donald Waller
Helen Waller
Jo Waller
Brenda Walles
Alexander Walley
Justin Walley
Tristan Wallhead
Håkan Wallin
Robert Wallinder
Heather Walline
Hm Walline
Anne M. Walling
Jason Walling
Corinna Wallinger
Carole Wallis
Gareth Wallis
Guy Wallis
Janette Wallis
Marianne Wallis
Michael Wallis
Robert Wallis
Bernard Wallner
Melissa Walls
Ramona Walls
Owen Wally
Cj Walsby
Carolyn Walsh
Conor Walsh
Craig Walsh
Dana Walsh
Daniel Walsh
Edward Walsh
Gregory Walsh
John Walsh
Kathleen Walsh
Kyle Walsh
Megan Walsh
Michael Walsh
Sophie Walsh
Stephen Walsh
Tom Walsh
William Walsh
David Walt
Benjamin Walter
Fruzsina Walter
Katharine Walter
Louise Walter
Lutz Walter
Mark Walter
Nicholas Walter
Richard Walter
Ronald Walter
Scott Walter
W. David Walter
Mark Walterfang
Arthur Walters
Eric Walters
J. Walters
John Walters
Keith Walters
Elizabeth Walter-Shea
Stephan Walterspacher
Nathan Waltham

Birgit Walther
Grit Walther
Paul Walther
Sebastian Walther
Thomas Walther
Marina Walther-Antonio
Pierre Walthery
Ludo Waltman
Chad Walton
Joseph Walton
William Walton
Margaret Walton-Roberts
Wendy Walwyn
Thomas Walz
Rosemary Walzem
Richard Wamai
Meredith Wampler-Kuhn
Bruce Wampold
C. W. S. Wan
Fc Wan
Hongquan Wan
Julian Wan
Lei Wan
Qiquan Wan
Shixiang Wan
Xia Wan
Xiao-Chun Wan
Xiaoyang Wan
Xiu-Feng Wan
Xuechao Wan
Xuehua Wan
Yanmin Wan
Ying Wan
Yinglang Wan
Yinsheng Wan
Yu-Feng Wan
Yung-Liang Wan
Wan Safwani Wan Kamarul Zaman
Jon Wanagat
Metha Wanapat
Hans Wandall
Brian Wandell
Joseph Wander
Wolfgang Wanek
Aijun Wang
Aiming Wang
Aisuo Wang
Amy Wang
Andrew Wang
Anthony Wang
Aolin Wang
August Wang
B. G. Wang
Baohua Wang
Baosheng Wang
Beixi Wang
Benquan Wang
Ben-Xin Wang
Biao Wang
Bin Wang
Bing Wang
Bingxue Wang
Bohan Wang
Bour-Jr. Wang
C. Wang
Caiyun Wang
Changbiao Wang
Chao Wang
Chao-Hung Wang
Chaozhan Wang
Chen Wang
Chengming Wang
Cheng-Ping Wang
Chengqi Wang
Chen-Ying Wang
Chi Wang
Chien-Chia Wang
Chih-Chi Wang
Chih-Hung Wang
Chi-Jane Wang
Ching-Ho Wang
Ching-Wei Wang
Chin-Tien Wang
Chong Wang
Chongjun Wang
Chuang Wang
Chun Wang
Chun-Chieh Wang

Chun-E Wang
Chunliang Wang
Chunlin Wang
Chunyan Wang
Chunyu Wang
Conan Wang
Congli Wang
Congrong Wang
Cui Wang
Cw Wang
Da Mao Wang
Dabuxilatu Wang
Dai Wang
Dajiang Wang
Dapeng Wang
Daping Wang
David Wang
Dayong Wang
Deqiang Wang
Deshou Wang
Dianhui Wang
Dong Wang
Donghua Wang
Dongqing Wang
Duan Wang
Duochun Wang
Edith Wang
Enru Wang
Eric Wang
Fa Yuan Wang
Fan Wang
Fang Wang
Fei Wang
Feilong Wang
Feng Wang
Gang Wang
Gang Wei Wang
Gang-Jin Wang
Guan Wang
Guangchao Wang
Guanghua Wang
Guangxing Wang
Guangyi Wang
Guan-Hong Wang
Guanyu Wang
Guifang Wang
Guijing Wang
Guiling Wang
Guiming Wang
Guirong Wang
Guixue Wang
Guo-Dong Wang
Guohua Wang
Guoye Wang
Guoying Wang
Guozhen Wang
Hai-Bo Wang
Haichang Wang
Haichao Wang
Haihua Wang
Hai-Jie Wang
Haixian Wang
Haiyan Wang
Han Wang
Han-Ching Wang
Hansong Wang
Hao-Ven Wang
Heming Wang
Hengtong Wang
Hequn Wang
Hetang Wang
Hong Wang
Hong-Bin Wang
Hongbing Wang
Hongchao Wang
Hongjie Wang
Hongjun Wang
Hong-Lun Wang
Hongmin Wang
Hongran Wang
Hongxia Wang
Hongyang Wang
Hongzhi Wang
Horng-Dar Wang
Hsin-Ell Wang
Hua Wang
Huafeng Wang
Huahua Wang

Huaichun Wang
Huaimin Wang
Hua-Ling Wang
Huanjiong Wang
Hui Wang
Hui-Cong Wang
Huijuan Wang
Huiming Wang
Huitian Wang
Hui-Yu Wang
Hung-Jung Wang
Hurng-Yi Wang
I-Jen Wang
I-Jong Wang
I-Kuan Wang
Irene Wang
J. G. Wang
Jann-Tay Wang
Jaw-Yuan Wang
Jean Wang
Ji Wang
Jia Wang
Jia-Kang Wang
Jiakun Wang
Jialiang Wang
Jian Wang
Jianbo Wang
Jiandong Wang
Jianfei Wang
Jianguo Wang
Jianhua Wang
Jiankang Wang
Jianke Wang
Jianmin Wang
Jianming Wang
Jianping Wang
Jian-Sheng Wang
Jianwei Wang
Jianwu Wang
Jianxin Wang
Jiaojian Wang
Jiaqing Wang
Jiayi Wang
Jia-Yi Wang
Jida Wang
Jiebiao Wang
Jih-Terng Wang
Jimeng Wang
Jin Wang
Jinfeng Wang
Jin-Feng Wang
Jing Wang
Jingfei Wang
Jing-Houng Wang
Jingming Wang
Jingwen Wang
Jingyu Wang
Jingyun Wang
Jinhui Wang
Jinke Wang
Jinniu Wang
Jinwei Wang
Jinxin Wang
Jinyu Wang
Jinzhong Wang
Jinzhou Wang
Jiu Yao Wang
Jiun-Ling Wang
Joanne Wang
John Wang
Jong-Shyan Wang
Joseph Tao-Yi Wang
Josh Jianxin Wang
Juan Wang
Jue (Carolyn) Wang
Jue Wang
Juejin Wang
Jun Wang
Jung-Der Wang
Junjian Wang
Junjie Wang
Junming Wang
Kai Wang
Kaijie Wang
Kan Wang
Kang Wang
Kanix Wang
Ke Wang

Kejian Wang
Kelin Wang
Kenneth Wang
Ketong Wang
Kevin Wang
Kewei Wang
Ko-Pen Wang
Kuei-Chun Wang
Kun Wang
Lan Wang
Lei Wang
Leon Wang
Li Wang
Lianchun Wang
Liang Wang
Liang-Jen Wang
Lianming Wang
Liansheng Wang
Licheng Wang
Li-Fang Wang
Lifeng Wang
Li-Guo Wang
Li-Hsuan Wang
Lihua Wang
Lijia Wang
Lijun Wang
Lili Wang
Lin Wang
Lina Wang
Linding Wang
Ling Wang
Lingling Wang
Lingxin Wang
Lin-Jie Wang
Liping Wang
Li-San Wang
Li-Shun Wang
Lixin Wang
Lizhong Wang
Lp Wang
Lu Nick Wang
Lubin Wang
Lubing Wang
Luqiao Wang
Meng Wang
Michael Wang
Ming Li Wang
Ming-Cheng Wang
Mingyi Wang
Mingyu Wang
Minxian Wang
Mo Wang
Mu Wang
Na Wang
Nancy Wang
Nan-Mai Wang
Nian Wang
Ning Wang
Ningli Wang
Ningning Wang
Ningtao Wang
P. Jeremy Wang
Pei Wang
Pei-Hui Wang
Peitao Wang
Peng Wang
Pin Wang
Pingli Wang
Pu Wang
Qi Wang
Qiang Wang
Qianghu Wang
Qiaohua Wang
Qiaomei Wang
Qiaoying Wang
Qi-En Wang
Qilong Wang
Qin Wang
Qing Wang
Qinghong Wang
Qinglian Wang
Qingwen Wang
Qingyun Wang
Qinhong Wang
Qiongxin Wang
Qiuyu Wang
Quan Wang
Quanhua Wang

Qun Wang
Ran Wang
Ren Wang
Rong Wang
Rubin Wang
Rui Wang
Ruihui Wang
Ruijia Wang
Ruiming Wang
Ruiping Wang
Ruisi Wang
Runping Wang
Ruofan Wang
S. C. Wang
Sa Wang
Seok Mui Wang
Sha Wang
Shanquan Wang
Shaohui Wang
Shaojian Wang
Shaokun Wang
Shaomeng Wang
Sheng Wang
Shengke Wang
Shengzhang Wang
Shen-Nien Wang
Shi Wang
Shi-An Wang
Shi-Gui Wang
Shih-Kai Wang
Shiping Wang
Shiqi Wang
Shiyang Wang
Shu-Chi Wang
Shu-Huei Wang
Shuihua Wang
Shukui Wang
Shuliang Wang
Shuo Wang
Shur-Jen Wang
Shusen Wang
Shuxia Wang
Simeng Wang
Song Wang
Stephanie Wang
Su Wang
T. H. Wang
T. Wang
Tao Wang
Taotao Wang
Thomas Wang
Tian Wang
Tiansheng Wang
Tian-Yun Wang
Tieyu Wang
Ting Wang
Ting-Yu Wang
Tong Wang
Tuanlao Wang
W. M. Wang
Wangsheng Wang
Wei Wang
Weichen Wang
Wei-Jie Wang
Wei-Kuang Wang
Wei-Lin Wang
Wei-Ting Wang
Wenhua Wang
Wenjuan Wang
Wenqin Wang
Wenquan Wang
Wenxin Wang
William Shi-Yuan Wang
Winfred Wang
Xia Wang
Xiangdong Wang
Xiao Wang
Xiaodong Wang
Xiao-Dong Wang
Xiaodu Wang
Xiaofang Wang
Xiaofeng Wang
Xiaogang Wang
Xiao-Hong Wang
Xiaojing Wang
Xiaoju Wang
Xiaolei Wang
Xiaolu Wang

Xiaoping Wang
Xiao-Ping Wang
Xiaoqin Wang
Xiaoqing Wang
Xiaotong Wang
Xiaowei Wang
Xiaoxi Wang
Xiaoyi Wang
Xicheng Wang
Xifeng Wang
Xin Wang
Xingang Wang
Xingxiang Wang
Xingya Wang
Xingyuan Wang
Xinjiang Wang
Xinlei (Sherry) Wang
Xinlong Wang
Xinping Wang
Xinxin Wang
Xinyu Wang
Xiue Wang
Xiuhua Wang
Xiuli Wang
Xiuqing Wang
Xiyang Wang
Xiyin Wang
Xu Wang
Xue Wang
Xuefeng Wang
Xuehui Wang
Xuequn Wang
Xuerong Wang
Xuewen Wang
Xueyan Wang
Xujing Wang
Xun Wang
Xuwen Wang
Xu-Wen Wang
Yadong Wang
Yajing Wang
Yan Wang
Yandan Wang
Yang Wang
Yangming Wang
Yanhui Wang
Yanlin Wang
Yanlu Wang
Yanshan Wang
Yanzhuang Wang
Yaxing Wang
YaXing Wang
Ya-Yun Wang
Yazhong Wang
Yeli Wang
Yen-Feng Wang
Yi Nan Wang
Yi Wang
Yi-Chen Wang
Yigang Wang
Yihong Wang
Yijie Wang
Yi-Lei Wang
Yin Wang
Ying Wang
Yingjie Wang
Ying-Wei Wang
Yingxiang Wang
Yingyi Wang
Yingying Wang
Yining Wang
Yinron Wang
Yinshan Wang
Yi-Ping Wang
Yisheng Wang
Yiwei Wang
Yong Wang
Yongbiao Wang
Yongbo Wang
Yonghong Wang
Yonghua Wang
Youwei Wang
Yuan Wang
Yuanbo Wang
Yuanchao Wang
Yuanhong Wang
Yuanyuan Wang
Yuanzhi Wang

Yuanzhong Wang
Yucheng Wang
Yue Wang
Yuesong Wang
Yuhua Wang
Yuji Wang
Yujiong Wang
Yuliang Wang
Yumeng Wang
Yuming Wang
Yu-Ming Wang
Yun Wang
Yunhe Wang
Yunsheng Wang
Yunxia Wang
Yuru Wang
Yutao Wang
Yuxi Wang
Z. Wang
Zan Wang
Ze Wang
Zeneng Wang
Zhangyang Wang
Zhanwei Wang
Zhan-You Wang
Zhaohui Wang
Zhaoxia Wang
Zhe Wang
Zhenbao Wang
Zhen-Bo Wang
Zheng Wang
Zhenghe Wang
Zheng-Xu Wang
Zhengyi Wang
Zhenjia Wang
Zhibo Wang
Zhifang Wang
Zhiguang Wang
Zhi-Lu Wang
Zhiwen Wang
Zhixiang Wang
Zhiyong Wang
Zhizhi Wang
Zhongqiu Wang
Zhongxiao Wang
Zhongyu Wang
Zhou Wang
Zixin Wang
Ziyi Wang
Ziyu Wang
Zunyao Wang
Zuoguang Wang
Zuoheng Wang
Zuoyao Wang
Kinley Wangdi
Sujata Wangkheimayum
Daniel Wangpraseurt
Altaf Wani
Nisar Wani
Marcin Waniek
Susitha Wanigaratne
Vishvarani Wanigasekera
Amal Wanigatunga
David Wanik
Umesh Wankhade
D. Wankhede
Kathleen Wannemuehler
Kevin Wanner
Samuel Wanner
Bill Wannop
Laurent Wantiez
Sylvia Wanzala
Humphrey Wanzira
Wendela Wapenaar
Torsten Wappler
Darren E. R. Warburton
Elizabeth Warburton
Marilyn Warburton
E. Warchałowska-Śliwa
Emily Ward
Jamie Ward
Ken Ward
Leigh Ward
Marie-Louise Ward
Mary Ward
Nicholas Ward
Peter Ward
Renee Ward

Robert Ward
Thomas Ward
Zachary Ward
Claire Wardak
Cavin Ward-Caviness
Inger Wårdh
J. Wardlaw
Chris Ware
Jerry Ware
Norma Ware
Joanna Wares
Terry Warholak
Molly Waring
Christina Warinner
Vivek Warkad
Sarah Warkentin
Theodore Warkentin
Majid Warkiani
Jane Warland
Michiel C. Warlé
Soren Warming
Saman Warnakulasuriya
Christina Warnecke
Barbara Warner
Chris Warner
Erica Warner
John Warner
Timothy Warner
Clemens Warnke
Amanda Warr
Anne Warren
Charlotte Warren
Christine Warren
Christopher Warren
Fiona Warren
Joseph Warren
Katherine Warren
Kristin Warren
Lucas Warren
Narelle Warren
Paul Warren
Robin Warren
Sharon Warren
Steve Warren
Philip Warrick
Keith Warriner
Jonas Warringer
Nicole Warrington
Adilia Warris
Arjumand Warsy
Reece Wartenberg
Karolina Wartolowska
David Warton
Kristina Warton
Anthony Waruru
Missaka Warusawitharana
Pawel Waryszak
Benjamin Waschki
Katherine Waselkov
Philip Washbourne
Auriel Washburn
David Washburn
Michael Washburn
Kazuo Washida
Naoki Washida
Gapare Washington
Robert Washington-Allen
Yukiko Washio
Kazushige Washizaki
Jay Wason
Leonard Wassenaar
Trudy Wassenaar
Christoph Wasser
Samuel Wasser
Gideon Wasserberg
Clive Wasserfall
Jennifer Wasserman
Michael Wasserman
Fentaw Wassie
Eugen Wassiliwizky
Samuel Wassmer
Dariusz Wasyl
Bohdan Wasylyk
Kounosuke Watabe
Shugo Watabe
Tadashi Watabe
Akihiko Watanabe
Hidemi Watanabe

Hiroshi Watanabe
Katsutoshi Watanabe
Masayuki Watanabe
Shuichi Watanabe
Tomonobu Watanabe
Toshihiro Watanabe
Yosuke Watanabe
Taketo Watase
Karen Watchman
Lynn Waterhouse
Matti Waterman
Richard Watermeyer
Allison Waters
Charles Waters
Elizabeth Waters
Michael Waters
Nigel Waters
Patrick Waters
Valerie Waters
W. Waters
Patrick Waterson
John Waterton
Malcom Watford
D. Wathes
Elizabeth Watkin
Adam Watkins
Christopher Watkins
David Watkins
John Watkins
Laura Watkins
Shannon Watkins
Stephanie Watkins
Laurent Watroba
Bruno Watschinger
Cathy Watson
Dennis Watson
Joseph Watson
Linda Watson
Roger Watson
Tamara Watson
Wendy Watson
Fiona E. Watt
Roger Watt
Rory Watt
Alice Wattam
Peemongkon Wattananon
Elizabeth Wattenberg
Kristi Watterberg
Pierre Wattiau
Nick Wattie
Ashlee Watts
Caroline Watts
David P. Watts
Duncan Watts
George Watts
Ian Watts
Joel Watts
Joseph Watts
Stephanie Watts
Stephen Watts
Henrik Watz
Ralf Watzlawick
Bianca Waud
John Waugh
Aurélien Wauters
Lucas Wauters
Nadeeka Wawegama
Adam Wax
David Waxman
Joshua Waxman
Stephen Waxman
Heidi Wayment
Nicholas Waytowich
Mohammad Wazid
Artur Wdowiak
David Wear
Robert Wears
Dan Weary
Anthony Weatherley
Adam Weaver
Chris Weaver
Connie Weaver
David Weaver
Mark Weaver
Samantha Weaver
Barbara Webb
Christine Webb
Grahame Webb

Ian Webb
Ken Webb
Michael R. Webb
Penelope Webb
Stephen Webb
Tonya Webb
William Webb
Sandra Webber
Sophie Webber
Allison Webel
Andreas Weber
Arthur Weber
Bernhard Weber
Daniela Weber
David M. Weber
Fabian Weber
Friedemann Weber
Georg F. Weber
Janet Weber
Jf Weber
K. Scott Weber
Konrad Weber
Marc-André Weber
Mary Weber
Melanie Weber
Thomas Weber
Laure Weber-Vintzel
Amanda Webster
Berenika Webster
Bonnie Webster
John Webster
Nicole Webster
Peter Webster
Simon Webster
A. Wechalekar
Larry Wechsler
Floyd Weckerly
Markus Weckmann
Lisa Wedding
Neil Wedlock
Chong-Yaw Wee
Natalie Wee
Séamas Weech
Gareth D. Weedall
Brendan Weekes
Andrew Weeks
Brian Weeks
Emma Weeks
Kevin Weeks
Lori Weeks
William Weeks
Eranthie Weerapana
Harshini Weerasinghe
Manori Weeratunga
Scott Weese
David Weetman
Ezra Wegbreit
Henning Wege
Mia Wege
Stefan Weger
Ulrich Weger
Andrea Weghofer
Michael Wegner
Christian Wehenkel
Axel Wehrend
Fernando Wehrmeister
Andrew Wei
Changli Wei
Chaoling Wei
Chen Wei
Chi-Ju Wei
Dang-Heng Wei
Dongqing Wei
Fan Wei
Fang-Fei Wei
Fulan Wei
Guowu Wei
Hairong Wei
Hong Wei
Hong-Jiang Wei
Hongping Wei
Huafeng Wei
Hui Wei
Jianing Wei
Jiufeng Wei
Jiwu Wei
Juncheng Wei
Kang Wei

Lai Wei
Ling Wei
Long Wei
Meiqin Wei
Minxin Wei
Qingqing Wei
Qingshan Wei
Ru Wei
Shu-Chen Wei
Shu-Jun Wei
Tonglu Wei
Wei Wei
Wenbin Wei
Xiaohui Wei
Xiaolin Wei
Xiaomei Wei
Ya-Lan Wei
Yanqiang Wei
Yiliang Wei
Yina Wei
Yingying Wei
Yongbin Wei
Yonglong Wei
Yongyue Wei
Yu-Chung Wei
Z. J. Wei
Zhiliang Wei
Gabrielle Weidemann
Michael Weiden
Christopher Weidenmaier
Matthias Weider
David Weidman
Manfred Weidmann
Nils Weidmann
Sara Weidmayer
Ralph Weidner
Markus Weigand
Steffen Weigend
Andreas Weigert
Cora Weigert
Roberto Weigert
S. Weigt
Shannon Weigum
Martin Weih
Zhang Weihua
Roxane Weijenberg
Danielle Weiler
Richard Weiler
Rosa Weiler
Arved Weimann
Lawrence Wein
Nicolas Wein
Gerhard Weinbauer
Annelie Weinberg
Clarice Weinberg
David Weinberg
Richard Weinberg
Zasha Weinberg
Daniel Weinberger
Miles Weinberger
Günther Weindl
Carl Weiner
Eran Weiner
Ina Weiner
January Weiner
Evan Weingarten
Markus Weingarth
Birgit Weingessel
Alexander Weinhold
Carolyn Weiniger
John Weinman
Neal Weinreb
U. Weinreich
Shantel Weinsheimer
Galit Weinstein
John Weinstein
Karen Weinstein
Robert Weinstein
Jaco Weinstock
Lauren Weinstock
Adi Weintraub
Genevieve Weir
Joseph Weir
Matthew Weir
Richard Weir
Michelle Weirathmueller
David Weis
Nina Weis

William Weis
Deena Weisberg
Christopher Weise
John Weisel
Sara Weisenbach
Giora Weiser
Glen Weiser
Thomas Weiser
Kristen Weishaar
Arthur Weiss
Barry D. Weiss
Bernhard Weiss
Craig Weiss
Curtis Weiss
Helen Weiss
Johannes Weiß
Lawrence Weiss
Louis Weiss
Mark Weiss
Michael A. Weiss
Noel Weiss
Robert Weiss
Taylor Weiss
Tobias Weissgerber
Daniel Weissman
Irit Weissman-Fogel
Chelsea Weitekamp
Tomas Weitoft
Mervyn Weitzmann
Megan Weivoda
He Weixiong
Ronald Wek
Scott Welak
Jariya Welbat
Sarah Welby
Andreanna Welch
D. Welch
Danny Welch
H. Welch
Heather Welch
James Welch
Mark Welch
Boye Welde
Christopher Weldon
Paul Weldon
Rebecca Weldon
Nathan Welham
Aalim Weljie
Erik Welk
Martin Welk
Keith Welker
David Welkie
Donald Weller
Jennifer Weller
Sandra Weller
Melanie Wellington
Barry Wellman
Charles Wellman
Hannah Wellman
Beth Wells
Brian Wells
Chad Wells
Ellen Wells
Hannah Wells
J. Wells
James Wells
Jeffrey Wells
Jim Wells
Kentwood Wells
Mark Wells
Scott Wells
Sue Wells
Wendy Wells
Yvonne Wells
David Wellsted
David Welsh
Ian Welsh
JoEllen Welsh
Paul Welsh
Robert Welsh
Kathleen Welsh-Bohmer
Wade Welshons
Paco Welsing
Riccardo Welters
Françoise Wemelsfelder
Franziska Wemheuer
Aidong Wen
Amy Wen

Chen Wen
Chi-Kuang Wen
Colin Wen
Deliang Wen
Guanghui Wen
Hong-Mei Wen
Jiahao Wen
Jie Wen
Jin-Kun Wen
Junhao Wen
Li Wen
Qiuting Wen
Rong Wen
Sijin Wen
Tzai-Hung Wen
Wen Wen
Xiang Wen
Yan Wen
Yao Wen
Yingqiang Wen
Zhiqiang Wen
Adam Wende
Kristian Wende
Wolfgang Wende
Aaron Wendelboe
David Wendell
Claire Wendland
Olaf Wendler
Mark Wener
Andrew Weng
Ching-Feng Weng
Chi-Yu Weng
Helen Weng
Juyang Weng
Lei Weng
Mao-Lun Weng
Shijun Weng
Shinuo Weng
Wen-Kai Weng
Yanqiu Weng
Tobias Wengenmayer
Amelia Wenger
Georg Wengert
Tang Wenjing
Caspar Wenk
Alexandra Wennberg
Roman Wenne
Benjamin Wenner
Theodore Wensel
Annemarie Wensing
Jeffrey Wenstrup
Felix Wensveen
Cui WenTao
Kirsten Wentlandt
Xu Wenxiang
Eva Wenzel
Mario Wenzel
Philip Wenzel
Tom Wenzel
Ulrich O. Wenzel
Wolfgang Wenzel
Frank WenzHöFer
Eric Wenzler
Benedict Weobong
Hang-Yeon Weon
Thomas Weppelmann
Dan Werb
Nomi Werbeloff
Lawrence Were
Elizabeth Weretilnyk
Wopke Werf
Heidrun Wergeland
Lars Werhagen
Kai Wermker
Thomas Wernberg
Guilherme Werneck
Erika Werner
Guido Werner
Haim Werner
Maria Werner
Martin Werner
Philipp Werner
Rene Werner
Thomas Werner
Allison Werner-Lin
Sara Wernersson
Kerstin Wernike
Jonathan Werry

Alexander Werth
Esther Werth
Joel Wertheim
Mark Wertheimer
Maria M. Wertli
Margaret Werts
Jasmin Wertz
Eduardo Weruaga
Jørgen Wesche
Daniel Wescott
Heather Wescott
Wim Wesemael
Jagila Wesley
Johnna Wesley
Susan Wesmiller
Jeff Wesner
Olga Wesolowska
Robert Wesolowski
Jürgen Wess
Niels Wessel
Mariska Wesseling
Hunter Wessells
K. Wessells
Robert Wessells
Daniel Wesson
Elizabeth Wesson
Andrew West
Brady West
Brooke S. West
Christopher West
Colin West
Daniel West
Greg West
Hannah West
James C West
Janne West
Jevin West
Kathryn West
Keith P. West
Nancy West
Natalie West
Nicholas West
Nicola West
Robert West
Lars Westberg
Andrew Westbrook
Chris Westbury
Sarah Westbury
Corli Westcott
Virginie Westeel
Peter Westenskow
Nelli Westercamp
Shawn Westerdale
Bjorge Westereng
Berend Westerhof
Paul Westerhoff
Maria Westerholm
Tommy Westerlund
Thomas Westermaier
Erica Westerman
Benedikt Westermann
Jukka Westermarck
Gunilla Westermark
Mark Western
Theresia Westers
Gert Westert
Klaas Westerterp
Margaret Westfall
Carri Westgarth
Philip Westgate
Marita Westhrin
James Westland
J. Lee Westmaas
Eric Westman
Peter Westman
Judy West-Mays
Mark Westneat
Christina Westphal
Matthias Westphal
Johanna Westra
Daniel Westreich
Erica Westwood
Heather Westwood
James Westwood
Bradley Wetherbee
Elaine Wethington
James Wetmore
Stacy A. Wetmore

Alyssa Wetterau
Shawn Wettig
Eunike Wetzel
Ronald Wetzel
William Wetzel
Kimberley Wever
Vera Wewer
Deborah Wexler
Tina Wey
Jacqueline Weyer
Karsten Weylandt
Christopher Whalen
Joann Whalen
Ben Whalley
Edward Whang
Joyce Whang
Young Whang
Jon Wheat
Aaron Wheeler
David Wheeler
Derek Wheeler
Graham Wheeler
Richard Wheeler
Ward Wheeler
Katherine Wheeler-Martin
Ana Wheelock
Brendan Whelan
Christopher Whelan
Kimberly Whelan
Patrick Whelley
Sarah Wherry
Kathryn Whetten
Geoff While
Corrie Whisner
Desley Whisson
John Whitaker
Michael Whitaker
Brian Whitcomb
Alex White
Billy White
Daniel White
Dave White
David White
Elisha White
Fletcher White
Garry White
Gary C. White
Jen White
Kate White
Kevin White
Kirsten White
Leonard White
Lori White
Michael White
Neil White
Norman White
Patricia White
Richard White
Robert White
Robin White
Sara White
Sarah White
Scott White
Sean White
Shane White
Thomas White
Tom White
Hal Whitehead
Ross Whitehead
Christopher Whitehurst
Michael Whitehurst
Benjamin Whitelaw
Julian Whitelegge
Victoria Whitelock
Andrew Whiten
Theresa Whiteside
Timothy J. S. Whitfeld
Alan Whitfield
Marjorie Whitfield
Martin Whitham
Penny Whiting
Matthew Whitley
Brian Whitlock
Kathleen Whitlock
Carol Whitney
Julie Whitney
Paul Whitney
Tim Whitsett

Timothy Whitsett
Heather Whitson
Jennifer Whitson
Jeremy Whitson
Gary Whittaker
Joseph Whittaker
Tony Whitten
Camilla Whittington
Kjersten Whittington
Monica Whitty
Jennifer Whitwell
Kristin Whitworth
Kristina Whitworth
Steve Whyard
Elisabeth Whyte
Shona Whyte
Florence Wianny
Gudrun Wibbelt
Daniel Wibberg
Marieke Wichers
Julian Wichmann
Oliver Wicht
Dan Wichterle
Elizabeth Wick
Macdonald Wick
Christine Wickens
Anthony Wickham
Samuel Wickline
Reed Wickner
Kremlin Wickramasinghe
Mary Wicksten
Eric Wickstrom
J. Widder
Julian Widder
Ulla Wide
Randall Widelitz
Laurie Wideman
Robert Widerman
Alik Widge
Kimberley Widger
Alan Widgerow
Kurt Widhalm
Hans Widlund
Laura Widman
Nicole Widmar
Franco Widmer
Giovanni Widmer
Hans Widmer
Jay Widmer
Samantha Widmeyer
Sheldon P. Wiebe
Eric Wieben
Oliver Wieben
Christine Wiebking
M. Wieckiewicz
Charles Wiedmeyer
Rudolf Wiegand
Armin Wiegering
Therese Wiegers
Brian Wiegmann
Lisa Susan Wieland
Susan Wieland
Piotr Wielopolski
Mark Wielpütz
Lyle Wiemerslage
Josef Wiemeyer
Howard Wiener
Seth Wiener
Julian Wienert
Brian Wienhold
Andreas Wienke
Pamala Wiepking
Richard Wierichs
Corinde Wiers
Rodney Wiersma
Justyna Wierzbicka
Holger Wiese
Stefan Wiese
Matthias Wieser
Lars Wieslander
Alex Wiesman
Lucas Wiessing
Brian Wigdahl
George Wiggans
Øystein Wiggen
Gregory Wiggins
Janey Wiggs
Paul Wigley

Sobanawartiny Wijeakumar
Chandrika Wijerathne
Tissa Wijeratne
Herman Wijnen
Lukasz Wiklendt
Df Wikoff
John Wikswo
Linda Wilbrecht
Damien Wilburn
Matthew Wilce
Bridget Wilcken
Andrew Wilcock
Donna Wilcock
David Wilcockson
Travis Wilcoxen
Walter Wilczynski
Beate Wild
Katherine Wild
Margaret Wild
Parke Wilde
Brigitte Wildemann
Britt Wildemann
Benjamin Wilder
Jocelyn Wilder
Tom Wilderjans
Mary Wildermuth
Moritz Wildgruber
Martin Wilding
Raelene Wilding
Christine Wildsoet
Vari Wileman
Craig Wilen
Dorothy Wiley
Emily Wiley
James Wiley
Michael Wiley
Robert Wiley
Veronica Wiley
Traci Wilgus
Adalbert Wilhelm
Barbara Wilhelm
Clare Wilhelm
Dirk Wilhelm
Helmut Wilhelm
Margit Wilhelm
Oliver Wilhelm
Charlotte Wilhelm-Benartzi
Ulrika Wilhelmsson
D. Ray Wilhite
Anna Wilk
Claus Wilke
Hans-Joachim Wilke
Thomas Wilke
Heinrike Wilkens
Caroline Wilkensin
Barbara Wilkey
David Wilkie
Iain Wilkie
Stephanie Wilkie
Thomas Wilkie
Hendrik Wilking
Jayne Wilkins
Anna Wilkinson
Christina Wilkinson
David Wilkinson
J. Mark Wilkinson
Jenny Wilkinson
Joyce Wilkinson
Keith Wilkinson
Thomas J. Wilkinson
Colin Wilks
Erin Wilkus
Elżbieta Wilk-Woźniak
Manuel Will
Torsten Will
Andy Willaert
Susan Willard
Barry Willardson
Christian Willberg
Rebecca J. Willcocks
David Willcox
Michelle Wille
Florian Willecke
Louise Willemen
Mark Willems
Michel Willems
Staphan Willems
Philippe Willenz

Henning Willers
Eske Willerslev
Cali Willet
Jonathon Willets
Christopher Willett
Rebecca Willett
James Willey
Joshua Willey
Basem M. William
William N. William Jr.
Adrienne Williams
Alan Williams
Alexander Williams
Allison Williams
Alwyn Williams
Andrew Williams
Arthur Williams
B. A. Williams
Billy Williams
Blaise Williams
Brett Williams
Brian Williams
Christopher Williams
Darlisha Williams
David Williams
Dewayne Williams
Dionna Williams
Faustine Williams
Gabrielle Williams
Gareth Williams
Garrath Williams
Gary Williams
Guy Williams
Hadyn Williams
Ivor Williams
James Williams
Jan Williams
Jonathan Williams
June Williams
Justin H. G. Williams
Kipling Williams
Kristina Williams
Lana Williams
Lindsay Williams
Lisa Williams
Lovoria Williams
Mark Williams
Matthew Williams
Melissa Williams
Michelle Williams
Mitchel Williams
Nathaniel Williams
Neal Williams
Nicole Williams
Paige Williams
Patricia Williams
Perry Williams
Rachel Williams
Ray Williams
Redford Williams
Riccardo Williams
Richard Williams
Robin Williams
Sally Williams
Samual Williams
Scott Williams
Sophie Williams
Steve Williams
Steven E Williams
T. Williams
Thomas Williams
Tom Williams
Wendy Williams
Adele Williamson
Catherine Williamson
Diane Williamson
Esther Williamson
Heather Williamson
Jane Williamson
Patrick Williamson
Peter Williamson
Bettina Willie
Amanda Willig
Remy Willinger
Erin Willis
Joseph Willis
Stuart Willis
Zachary Willis

Maximilian Willkomm
Patrick Willems
Matthew Willman
Janna Willoughby
Pamela Willoughby
Andrew Wills
Martin Wills
Zachary Wills
John Willson
Bettina Wilm
Alan Wilman
Matthias Wilmanns
James Wilmott
Stephanie Wilsher
Maciej Wilski
Ander Wilson
Andrew Wilson
Anthony Wilson
Bethany Wilson
Brent Wilson
Bridget Wilson
Christopher J. Wilson
Cyril Wilson
Darrell Wilson
David Wilson
Deborah Wilson
Donald Wilson
Eleanor Wilson
Fiona Wilson
Greg Wilson
Hayley Wilson
Ian Wilson
James Wilson
Jeffrey Wilson
Jennifer Wilson
John Wilson
John-James Wilson
Justin Wilson
Kaitlyn P. Wilson
Karen Wilson
Kenneth Wilson
Kimberly Wilson
Mark Wilson
Michael Wilson
Nick Wilson
Nobert Wilson
Patrick Wilson
Peyton Wilson
Philip Wilson
R. Wilson
Rhodri Wilson
Robert Wilson
Robin Wilson
Ruth Wilson
Shaun Wilson
Shelby Wilson
Stuart Wilson
Sylia Wilson
Tammy Wilson
Thomas Wilson
Wyndham Wilson
Heather Wilson-Robles
Jennifer Wilson-Welder
Jörg Wilting
Stephen Wilton
Steve Wilton
Roswitha Wiltschko
Calistus Wilunda
Jeffrey Wilusz
Jeremy Wilusz
Catriona Wimberley
Michael Wimberly
Ernst Wimmer
Isabella Wimmer
Klaus Wimmer
Marina Wimmer
Gina M. Wimp
Aung Win
Kiara Winans
Peter Winch
David Winchester
Thomas Winckler
Joshua Wind
Virginia Winder
Anne Winding
Christian Windischberger
Brad Windle
Hannah Windley

Martin Windpessl
Paul Windschitl
Jack Windsor
Eytan Wine
Alan Wineman
Laura Winer
Robert Winfield
Au Wing
Yun Kwok Wing
Kristian Winge
Mårten Winge
Paul Winger
Noel Wingers
Marcelle Winget
Brenda Wingfield
Thomas Wingfield
Tom Wingfield
Aliza Wingo
Beverly Winikoff
Michael Wininger
Alle Meije Wink
Michael Wink
Per Winkel
Holger Winkels
Lizziane Winkelstroter
Piotr Winkielman
Melissa Winkle
Cheryl Winkler
Daniel Winkler
Johannes Winkler
Margit Winkler
Thomas Winkler
Konstanze Winklhofer
Michael Winklhofer
David Winlaw
Philip Winne
Eva Winnebeck
Ellen Winner
Katarzyna Winnicka
Dean Winslow
Brent Winston
John Winston
G. R. Wint
Carlos Winter
Christian Winter
Desmond Winter
Edward Winter
Jennifer Winter
Jessica Winter
Patricia Winter
Stephan Winter
Stuart Winter
Hanlie Winterbach
Stephen Winters
Almut Winterstein
Johan Winterwerp
Richard Wintle
Peter Wipf
Alexander Nimo Wiredu
Aaron Wirsing
Kathleen Wirth
Lori Wirth
Michael Wirth
Michelle Wirth
Oliver Wirths
Derrick Wirtz
Mary Wirtz
Theresa Wirtz
Rudi Wisaksana
Jonathan Wisco
Lauren Wise
Robert Wise
Toby Wise
Jennifer Wisecaver
Melody Wiseheart
R. Luke Wiseman
David Wishart
Joost Wiskerke
Alan Wisler
Sabine Wislet
Daniel Wisneski
Stephen Wisniewski
Scott Wissinger
Birte Wistinghausen
Joachim Wistuba
Henryk Witas
Selma Witchel
Michael Witcher

Henryk Witek
Maria Witek
David Withers
Melissa Withers
Jeffrey Withey
Veronique Witko-Sarsat
A. M. Witkowska
Marta Witkowska
Andrzej Witkowski
Benoit Witkowski
Jon Witman
Janusz Witowski
Mj Witry
Arne Witt
Olaf Witt
Hans-Ulrich Wittchen
Klaudia Witte
S. Witte
Veronica Witte
Wolfgang Witte
Falk Wittel
Uwe Wittel
Holly Witteman
Jurrian Witteman
Timo Wittenberger
Dörte Wittenburg
Luke Wittenburg
Paula Witt-Enderby
Gary Wittert
Briana Witteveen
Linda Wittkop
Christiane Wittmann
Johannes Wittmann
Kenneth Witwer
Azher Razzak Witwit
Carsten Witzel
Peter Witzgall
Maren Witzig
Alexander Witzki
Charles Wiysonge
Nick Wlazlo
Piotr Włodarczak
Anna Wlodarczyk
Mary Wlodek
Wendy Wobeser
Birgitta Woehrl
Godfrey Woelk
Tobias Woerner
Malte Woestmann
Cl Wogamon
Guinevere Wogan
Elizabeth Wohifert
Teddy John Wohlbold
Eric Wohleb
Ingo Wohlgemuth
Stephanie Wohlgemuth
Alexandre Wohlkonig
Wendel Wohlleben
Sean Wojcik
Katarzyna Wojczulanis-Jakubas
Dorota Wojnicz
Johann Wojta
Andrew Wojtovich
Eric Wolanski
Gregor Wolbring
Traci Wolbrink
Jeff Wolchok
Marc Wold
Yimtubezinash Woldeamanuel
Yohannes Woldeamanuel
Andrea Wolf
Cameron Wolf
Dennis Wolf
Douglas Wolf
Eckhard Wolf
Elmar Wolf
Erika Wolf
Harald Wolf
Jana Wolf
Jennifer Wolf
John Wolf
Jürgen Wolf
Kristina Wolf
Matthias Wolf
Michael Wolf
Patrick Wolf
Peter Wolf
Petra Wolf

Steven Wolf
Tiffany Wolf
Zena Wolf
Adam Wolfberg
Alan Wolfe
David Wolfe
Joanna Wolfe
Sierra Wolfenbarger
Daniel Wolff
Jan Wolff
Mark Wolff
Matthias Wolff
Michael Wolff
Bruce Wolffenbuttel
James Wolffsohn
Driever Wolfgang
Linden Wolfgang
Nicholas H. Wolfinger
Dietmar Wolfram
Christian Wolfrum
Lara J. Wolfson
Justyna Wolinska
Pierre Wolkenstein
Willem Wolkers
Barbara Wollenberg
K. Wollenberg Valero
Tobias Wollersheim
Kai Wollert
Lonnie Wollmuth
Patricia Wollstadt
Marc Wolman
William Wolowich
Jeffrey Wolt
Ernst Woltering
Maike Wolters
Julie Womack
Andrea Stevenson Won
Misun Won
Sungho Won
Young-Joo Won
Amy Wonder
Elizabeth Wonderlich
Dessie Salilew Wondim
Adrian Wong
Alex Wong
Anderson Wong
Andy Wong
Brian Wong
Carlos Wong
Chit Wai Wong
Cho Lee Wong
Christopher Wong
Chung F. Wong
Chung Wong
Connie Wong
David Wong
Elaine Wong
Flora Wong
George Wong
Gloria Wong
Grace Wong
Hector Wong
Henry Wong
Jason Wong
Jessica Wong
Jocelyn Wong
Jonathan Wong
Joshua Wong
Julia Wong
Kelvin Wong
Ken Koon Wong
Li Ping Wong
Lily Wong
M. S. Wong
Madeline Wong
Maria Wong
Marlon Wong
Melissa Wong
Ngai Sze Wong
Rachel Wong
Ronald Wong
Season Wong
Sheen Wong
Sing-Wai Wong
Suzy Wong
Sze Chuen Cesar Wong
Tak Pan Wong
Tit Yee Wong

Tuck Seng Wong
Tzu-Tsung Wong
Victor Chun-Lam Wong
Wai Wong
Weng Kee Wong
William W. L. Wong
Wilson Wong
Preechaya Wongkrajang
Gabrielle Wong-Parodi
Cherry Wongtrakool
Susan Wonnacott
Connie Woo
Jean Woo
Jeong-Taek Woo
John Woo
Jong-Min Woo
Sang Woo
Se Joon Woo
Sukyung Woo
Daniel Wood
David Wood
Ian Wood
Jamie Wood
Joanne Wood
John Wood
Kevin Wood
Lisa Wood
Mathew Wood
Matthew Wood
Rachel Wood
Spencer Wood
Stacey Wood
Stephen Wood
Susanna Wood
Thomas Wood
Troy Wood
Tyler Wood
William Woodall
Pamela Woodard
Neal Woodbury
Stephen Woodbury
Marc Woodbury-Smith
Ben Woodcock
Thomas Woodcock
Susannah Woodd
Lorayne Woodfield
Michael Woodford
James Woodhall
Francis Woodhouse
Jason Woodhouse
Jim Woodhouse
David Woodland
Neal Woodman
Emma Woodmansey
Carl Woods
Clive Woods
Gregory Woods
Heather Woods
Kerry Woods
Leah Woods
Michael Woods
Parker Woods
Stephen C. Woods
Steven Paul Woods
Steven Woods
Thomas S. Woodson
John Woodward
Mark Woodward
Owen Woodward
Steven Woodward
Wendy Ann Woodward
Craig Woodworth
Matthew Woolhouse
Kevin Woollard
Ian Woolley
J. Timothy Wootton
Sarah Wootton
R. Ann Word
Solomon Woreta
Thierry Work
Jerry Workman
Margitta Worm
Paulo Worm
Michael Wormstone
Marcus Worsley
Noel Wortham
Daniel Worthley
Leah Worton

Bernd Wöstmann
Franz Wotawa
Carsten Wotjak
David Wotton
Donna Woulfe
Anke Wouters
Edwin Wouters
Emiel Wouters
Inge Wouters
Paul Wouters
Marjan Wouthuyzen-Bakker
Andrew Wozniak
Karen Wozniak
Michał Woźniak
Grzegorz Woźniakowski
Lawrence Wrabetz
Jens Wrammert
John Wrangle
Anika Wranke
Dannah Wray
Laura Wray
Karsten H. Wrede
Anna Wredenberg
Alexander Wree
Jonathan Wren
Herrmann Wrigge
Brad Wright
Brent Wright
David Wright
Edwina Wright
Emma Wright
Eric Wright
Graham Wright
J. Timothy Wright
James Wright
Jerome Wright
Joshua Wright
Katharine Wright
Lori Wright
Mark Wright
Marvin Wright
Melecia Wright
Melissa Wright
Patricia Wright
Robert Wright
Rosalind Wright
Rosanna Wright
Scott Wright
William Wright
Katarzyna Wrobel
Kristin Wrobleski
Lydia Wroblewski
Jan Wrzesinski
Krzysztof Wrzesinski
Ailong Wu
Al Wu
Alan Wu
Alexander Wu
Amery Wu
Angela Wu
Bin Wu
Bing Wu
Bo Wu
Boyang Wu
Changcheng Wu
Changshan Wu
Chao Wu
Chaodong Wu
Chaozhong Wu
Chau-Chung Wu
Chen Wu
Chengbiao Wu
Cheng-Chia Wu
Cheng-Kun Wu
Chengtie Wu
Chen-Shiou Wu
Chia-Chao Wu
Chia-Ching Wu
Chia-Lung Wu
Ching-yi Wu
Christina Wu
Chuanyu Wu
Chung-Chun Wu
Colin Wu
Cynthia Wu
Dan Wu
Dezhi Wu
Di Wu

Dianxing Wu
Ding-Kwo Wu
Dong Chuan Wu
Dongrui Wu
Edwin Wu
Eveline Wu
Fan Wu
Fang-Tzy Wu
Feihua Wu
Fei-Yun Wu
G. C. Wu
Gang Wu
Gregory Wu
Guan-Chung Wu
Guangxi Wu
Guodong Wu
Guohao Wu
Guojin Wu
Guojun Wu
Haitao Wu
Haiyan Wu
Hao Wu
Hau Wu
Hongjie Wu
Hongkai Wu
Hongli Wu
Honglu Wu
Hongxian Wu
Hongzhuan Wu
Huei-Nan Wu
Hui Wu
Huijun Wu
Huiyan Wu
Jennifer Wu
Jia-Feng Wu
Jia-Ming Wu
Jianfeng Wu
Jian-Hui Wu
Jianqiang Wu
Jianyong Wu
Jiawang Wu
Jiayuan Wu
Jie Wu
Jim Wu
Jincheng Wu
Jinshan Wu
Jiunn-Tzong Wu
Jiyan Wu
Joanna Wu
John Wu
Joy Wu
Jun Wu
Junfang Wu
Junhua Wu
Kejin Wu
Kevin Wu
Kun-Pin Wu
Liang-Huan Wu
Li-Ling Wu
Lin-Bo Wu
Lingqian Wu
Linzhi Wu
Liyun Wu
Long-Jun Wu
Mei-Lin Wu
Min Wu
Ming Wu
Ming-Chya Wu
Ming-Heng Wu
Ming-Ju Wu
Ming-Ping Wu
Mingquan Wu
Monica S. Wu
Nan Wu
Ning Wu
Pei-Chang Wu
Pei-Chen Wu
Pei-Hsun Wu
Peipei Wu
Pui-Kei Wu
Qianhong Wu
Qiaofeng Wu
Qingbo Wu
Qingping Wu
Qingyao Wu
Qiong Wu
Qunhong Wu
Renyi Wu

Ruey-Meei Wu
Rui Wu
Ruibo Wu
Ruifang Wu
Sean Wu
Shang-Gin Wu
Shaohui Wu
Sheng Wu
Shengzhou Wu
Shey-Lin Wu
Shih-Chi Wu
Shih-Hsiung Wu
Shuicai Wu
Shulin Wu
Song Wu
Szu-Yuan Wu
Ta Yeong Wu
Tangchun Wu
Tao Wu
Te Wu
Tianfu Wu
Tsu-Yin Wu
Tzu-Yi Wu
Wanqing Wu
Wei Wu
Weiren Wu
Wen-Bin Wu
Wen-Chau Wu
Went Wu
Wenxiang Wu
X. M. Wu
Xiaofeng Wu
Xiaohong Wu
Xiaohua Wu
Xiaojin Wu
Xiao-Lin Wu
Xiao-Ming Wu
Xiaoping Wu
Xiaoyun Wu
Xinchun Wu
Xingtang Wu
Xinkai Wu
Xinyin Wu
Xiufeng Wu
Xufeng Wu
Xugan Wu
Yan Jing Wu
Yangfeng Wu
Yanhong Wu
Yanyan Wu
Yifan Wu
Yihong Wu
Yi-Jun Wu
Yimin Wu
Ying Choon Wu
Yinghao Wu
Yongda Wu
Yong-Ping Wu
Yongqi Wu
Yuanxin Wu
Yu-Chung Wu
Yuping Wu
Yu-Ting Wu
Yuzhe Wu
Yvonne Wu
Zeyan Wu
Zhaohui Wu
Zhiang Wu
Zhibing Wu
Zhijian Wu
Zhimeng Wu
Zhiqiang Wu
Zhiwei Wu
Zhouqiao Wu
Zhung-Han Wu
Zong-Meng Wu
Zunyou Wu
Ansgar Wübker
Darrell Wudunn
Peter Wuehr
Mark Wuenschel
Mignon Wuestman
Wahyu Wulaningsih
Dirk Wulff
Jeremy Wulff
Sven Wunder
Zeba Wunderlich
Phanindra Wunnava

Frederik Wurm
Tobias Würschum
Friedrich Wurst
Jean-Marie Wurtz
Sarah Wurz
Torsten Wüstenberg
Daniel Wüstner
Kaspar Wuthrich
Sonia Wutzke
Wim Wuyts
Christina Wyatt
Katherine Wyatt
Michael Wyatt
Nicky Wybouw
Sławomir Wybraniec
Miroslav Wyczesany
Adrian Wydeven
Lesley Wye
Brian Wygal
Til Wykes
Dennis Wykoff
Joanna Wykrzykowska
Ann Wylie
Dennis Wylie
Kristine Wylie
Stephen Wylie
Judith Wylie-Rosett
Susan Wyllie
Elvin Wyly
Matthias Wymann
Brian Wymbs
Grace Wyngaard
James Wynn
Julia Wynn
John Wysolmerski
Agnieszka Wyszynska
Stavra Xanthakos
Christos Xanthis
Melissa Xanthopoulos
Petros Xanthopoulos
André Xavier
Basil Britto Xavier
Gilberto Xavier
Caroline Regina Delma Xavier Paul
Ezhilan
Dionysis Xenakis
Michalis Xenos
V. Xepapadaki
Henri Xhaard
Chuanwu Xi
Gang Xi
Guohua Xi
Kelai Xi
Lei Xi
Min Xi
Ming-Rong Xi
Rongwen Xi
Xiaodong Xi
Yi-Bo Xi
Yutao Xi
Ai Xia
Ao Xia
Chao-Ming Xia
Cheng-Yi Xia
Hongfei Xia
Jianguo Xia
Jiangyan Xia
Kaiwen Xia
Liqiu Xia
Lirong Xia
Lu Xia
Mingfeng Xia
Ningshao Xia
Qiang Xia
Qingyou Xia
Rui Xia
Shujun Xia
Ting Xia
Xiao-Jian Xia
Xuhua Xia
Yang Xia
Yankai Xia
Yun Xia
Zhao-Fan Xia
Shiji Xiahou
Mo Xian
Ying Xian

Dehui Xiang
Fengning Xiang
Huiyun Xiang
Ming Xiang
Sen Xiang
Siheng Xiang
Tao Xiang
Wenhua Xiang
Wensheng Xiang
Xiaoguo Xiang
Yan Xiang
Yang Xiang
Yu Xiang
Zheng Xiang
Huang Xiangyan
Bin Xiao
Chunwang Xiao
Fei Xiao
Feifei Xiao
Guanghua Xiao
Hai-Peng Xiao
Han Xiao
Hang Xiao
Hong Xiao
Honglin Xiao
Hua Xiao
Jiajie Xiao
Jianzhong Xiao
Jie Xiao
Jin Xiao
Jin-Zhong Xiao
Junfeng Xiao
Lanbo Xiao
Li Xiao
Lihua Xiao
Lin Xiao
Ming Xiao
Naiqi Xiao
Pengcheng Xiao
Qiang Xiao
Rui Xiao
Shijun Xiao
Shuqi Xiao
Sumei Xiao
Xi Xiao
Xiangshu Xiao
Xiao Xiao
Xuerong Xiao
Xusheng Xiao
Yong Fu Xiao
Yong Xiao
Yonghong Xiao
Yuguo Xiao
Zhi Xiao
Zhong-Dang Xiao
Zhao Xiaogang
Long Xiaohua
Bai-Chen Xie
Bin Xie
Bowen Xie
Chuanxiao Xie
Conghua Xie
Dexuan Xie
Dong Xie
Fang Xie
Gang Xie
Gengqiang Xie
Guo-Sen Xie
Guosheng Xie
Hua-Tao Xie
Huijun Xie
Huikai Xie
Huisheng Xie
Jian-Hui Xie
Jianping Xie
Jing Xie
Junhui Xie
Jun-Hui Xie
Kabin Xie
Linglin Xie
Lingtian Xie
LiQin Xie
Peng Xie
Pengtao Xie
Shang Xie
Shanghong Xie
Shangxian Xie
Shao-Hua Xie

Shengbo Xie
Shicong Xie
Shouqi Xie
Shuguang Xie
Tao Xie
Wei-Ping Xie
Wenjia Xie
Wen-Jie Xie
Xiangqun Xie
Xiaoming Xie
Xueqian Xie
Xue-Qian Xie
Yang Xie
Yanping Xie
Yi Xie
Yibin Xie
Yuying Xie
Z. E. Xie
Zheng Xie
Zubin Xie
Cecilia Ximenez
Haiping Xin
Hong Xin
Lu Xin
Aiguo Xing
Changsheng Xing
Chao Xing
Chuanhua Xing
Danmou Xing
Liping Xing
Lizhi Xing
Qi Xing
Wanli Xing
Xin Xing
Yaowu Xing
Wang Xinyu
Ai-Sheng Xiong
Chengfeng Xiong
G. Xiong
Guangyan Xiong
Jian Ping Xiong
Jinbo Xiong
Lei Xiong
Li Xiong
Lifeng Xiong
Tou Cheu Xiong
Wu Xiong
Ye Xiong
Yin Xiong
Yong Xiong
Yulan Xiong
Zhili Xiong
Fangming Xiu
Zhilong Xiu
Aimin Xu
Aiqiang Xu
Angao Xu
Bei Xu
Bianli Xu
Biao Xu
Bin Xu
Chen Xu
Chengwu Xu
Chenming Xu
Chuandong Xu
Chuanshan Xu
Chun Xu
Da Xu
Daguang Xu
Dan Xu
Deli Xu
Dong Xu
Dongmei Xu
Duan Xu
Fei Xu
Feng Xu
Fengfeng Xu
Fenglian Xu
Fuhua Xu
Fujie Xu
Fuzhou Xu
Gongjun Xu
Guangquan Xu
Hai-Chuan Xu
Haineng Xu
Hanfu Xu
Heping Xu
Hong Xu

Honghe Xu
Hongwei Xu
Hongyan Xu
Huaxi Xu
Hui Xu
Jian Xu
Jianbo Xu
Jianhua Xu
Jianliang Xu
Jianping Xu
Jianrong Xu
Jintao Xu
Jisheng Xu
Jiting Xu
Juan Xu
Julia Xu
Junfeng Xu
Junkai Xu
Junwang Xu
Kai-Feng Xu
Ke Xu
Lei Xu
Li Xu
Liang Xu
Lisheng Xu
Meifeng Xu
Meng Xu
Mengsi Xu
Min Xu
Minggang Xu
Mingjiang Xu
Mingming Xu
Mingyi Xu
Ni Xu
Nianjun Xu
Nuwen Xu
Pan Xu
Pei Xu
Peng Xu
Ping Feng Xu
Ping Xu
Qing Xu
Quanbin Xu
Ren Xu
Renfeng Xu
Renshi Xu
Shengchun Xu
Shiqing Xu
Shu Xu
Shuai Xu
Shun Xu
Tianjun Xu
Ting-Yan Xu
Wei Xu
Wei-Hai Xu
Weirong Xu
Wenbo Xu
Wenfang Xu
Wentao Xu
Wenzhong Xu
Xianglong Xu
Xiang-Xi Xu
Xiaobiao Xu
Xiaobin Xu
Xiaokun Xu
Xiaoyong Xu
Xin Xu
Xingliang Xu
Xin-Jian Xu
Xuan Xu
Yan Xu
Yang Xu
Yanhao Xu
Yanjie Xu
Yechun Xu
Yi Xu
Yidong Xu
Yifan Xu
Ying Xu
Ying-Chun Xu
Yinghui Xu
Yingying Xu
Yixiang Xu
Yong Xu
Yuanqing Xu
Yue Xu
Yuhang Xu
Yunbi Xu

Yuting Xu
Zeshui Xu
Zheng Xu
Zhenzhu Xu
Zhichao Xu
Zhiliang Xu
Zhimin Xu
Zhiqiang Xu
Zhiwei Xu
Zongbo Xu
Qi Xuan
Tran Dang Xuan
Zhao Xu-Dong
Baolin Xue
Bing Xue
Chaoyang Xue
Cheng Xue
Dawei Xue
Feng Xue
Hong Xue
Huijie Xue
Jie Xue
Jinzhuang Xue
Lixiang Xue
Rong Xue
Rui-De Xue
Sheng-Guo Xue
Teng Xue
Xian Xue
Xiang Xue
Xiangyang Xue
Yali Xue
Yang Xue
Zuo Xue
Pengcheng Xun
Meng Xu-Welliver
Samuel Y. Boateng
Beery Yaakov
Rami Yaari
Patrick Yachimski
Magdi Yacoub
Sophie Yacoub
Talene Yacoubian
Ajar Nath Yadav
Anupama Yadav
Chandra Yadav
Dinesh Yadav
H. K. Yadav
Manuj Yadav
Mukesh Yadav
Prashant Yadav
Shailenda Kumar Yadav
Shriram Yadav
Siddhartha Yadav
Pramod Yadava
Tejabhiram Yadavalli
M. Yadrick
Mark Yaffe
Naoto Yagi
Koji Yahara
Daykan Yair
Mahmoud Yaish
Javed Yakoob
Sergiy Yakovenko
Vasily Yakovlev
Aminu Yakubu
Igor Yakymenko
Matt Yalch
Ozlem Yalcin
Ilhan Yalçın
Özgür Yaldizli
Jason C. S. Yam
Montarop Yamabhai
Akira Yamada
Hayato Yamada
Hisakata Yamada
Kaori Yamada
Kaoru Yamada
Norikazu Yamada
Sohsuke Yamada
Soichiro Yamada
Toshihiko Yamada
Kazuya Yamagata
Masakazu Yamagishi
Sho-Ichi Yamagishi
Atsushi Yamaguchi
Junna Yamaguchi
Masahiro Yamaguchi

Motoko Yamaguchi
Motonori Yamaguchi
Seiji Yamaguchi
Takashi Yamaguchi
Takefumi Yamaguchi
Takeshi Yamaguchi
Taku Yamaguchi
Yuki Yamaguchi
Keiko Yamaji
Takeo Yamakawa
Kouya Yamaki
Gamal Abdel Naser Yamamah
Akihito Yamamoto
Hideya Yamamoto
Kazuki Yamamoto
Kohji Yamamoto
Koichi Yamamoto
Masashi Yamamoto
Masato Yamamoto
Masayoshi Yamamoto
Masayuki Yamamoto
Nobuhiko Yamamoto
Norio Yamamoto
Ryohei Yamamoto
Satoshi Yamamoto
Seiji Yamamoto
Suguru Yamamoto
Tatsuya Yamamoto
Yasuhiko Yamamoto
Kazuhiko Yamamuro
Teresa Yamana
Hiroki Yamanaka
Kenta Yamanaka
Daisuke Yamane
Tomohiko Yamane
Koji Yamanegi
Noriko Yamano
Seiichi Yamano
Tomoyoshi Yamano
Keitaro Yamanouchi
Kunihiro Yamaoka
Keishi Yamasaki
Kenshi Yamasaki
Takahiro Yamasaki
Kenji Yamashiro
Atsushi Yamashita
Haruyuki Yamashita
Hiroharu Yamashita
Takehiro Yamashita
Tatsuya Yamashita
Tomohisa Yamashita
Tomoya Yamashita
Yukiko Yamashita
Tie Yamato
Mitsuo Yamauchi
Motohiro Yamauchi
Satoshi Yamauchi
Hiroki Yamaue
Akira Yamawo
Mutsuo Yamaya
Takayoshi Yamaza
Hiroshi Yamazaki
Kazuo Yamazaki
Soh Yamazaki
Wataru Yamori
Mark Yampolskiy
Biao Yan
Bin Yan
Chaogan Yan
Dandan Yan
Dong-Ming Yan
Erjia Yan
Gang Yan
Guangmei Yan
Guijun Yan
Hai Yan
Hong-Mei Yan
Huihuang Yan
Jiajie Yan
Jianhua Yan
Jianyun Yan
Jinchuan Yan
Jinpei Yan
Jinyuan Yan
Jizhou Yan
Jun Yan
Lei Yan
Lijing Yan

Lirong Yan
Qingpi Yan
Qingyun Yan
Sen Yan
Shian-Jang Yan
Ting Yan
Weiwei Yan
Wen Yan
Wenjun Yan
Xiaoyong Yan
Xuebing Yan
Ying Yan
Yiqing Yan
Yonghong Yan
Yongping Yan
Yue Yan
Yueming Yan
Yue-Ming Yan
Zhang Yan
Zhen Yan
Zhiqiang Yan
Yasuo Yanagi
Manabu Yanagita
Ruth Yanai
Ryoji Yanai
Paul Yancey
Brian Yandell
Tracy Yandle
Kei Yane
Elisabeth Yaneske
Betina Yanez
Maria Yanez
Carlos Yañez-Arenas
Maria Yanez-Mo
Anli Yang
Baichun Yang
Baijian Yang
Bao Yang
Baoru Yang
Bing Yang
Bo Yang
Caimei Yang
Caiyun Yang
Cao Yang
Changdeng Yang
Chang-Hao Yang
Changjun Yang
Charles Yang
Che-Ming Yang
Cheng-Chieh Yang
Chenguang Yang
Chenquan Yang
Chii-Shen Yang
Chinhua Yang
Chongguang Yang
Chuan Yang
Chuanwei Yang
Chung Yang
Chunsheng Yang
Chun-Song Yang
Cong Yang
Deng-Ho Yang
Deok-Hwan Yang
Dongqin Yang
En-Cheng Yang
Fan Yang
Fang Yang
Fei Yang
Fengtang Yang
Fu Chi Yang
Fuyu Yang
George Yang
Guanci Yang
Guangdong Yang
Guang-Ran Yang
Guijun Yang
Gunan Yang
Guomin Yang
H. S. Yang
Haining Yang
Haizhao Yang
Hanchun Yang
Haokai Yang
Hong Yang
Hongyuan Yang
Hu Yang
Huaan Yang
Huaiyu Yang

Hui Yang
Huijie Yang
Huijuan Yang
Hung-Chih Yang
Hushan Yang
Ivana Yang
Jaemoon Yang
James Yang
Jenny J. Yang
Jialiang Yang
Jian Yang
Jianchang Yang
Jianfeng Yang
Jianhua Yang
Jiaxin Yang
Jie Yang
Jing Yang
Jinghua Yang
Jingjing Yang
Jingzhen Yang
Jiongjiong Yang
Juan Yang
Jun Yang
Jungwoo Yang
Junning Yang
Junqi Yang
Ju-Yeh Yang
Jyh-Chin Yang
Kai Yang
Kai-Chien Yang
Kailin Yang
Keqiang Yang
Kuen Cheh Yang
Lang Yang
Lee-Wei Yang
Lei Yang
Li Yang
Lianping Yang
Li-Chan Yang
Lifen Yang
Li-Jun Yang
Lilin Yang
Limin Yang
Lingling Yang
Lixia Yang
Li-Ye Yang
Mary Yang
Meng Yang
Min Yang
Nae-Cherg Yang
Nianlan Yang
Ning Yang
Otto Yang
Peixin Yang
Pengyuan Yang
Ping Yang
Qian Yang Yang
Qianzi Yang
Qing Yang
Qinghua Yang
Qiong Yang
S. Y. Yang
Seung Hwan Yang
Shengping Yang
Sheng-Shun Yang
Shi Yang
Shihao Yang
Shi-Ming Yang
Shi-Xia Yang
Shu Yang
Shwu-Huey Yang
Songguang Yang
Songtao Yang
Stephan Yang
Stephen Yang
Su Yang
Sung Jae Yang
Sung-Yin Yang
Tao Yang
Tiantian Yang
Tse-Chuan Yang
Tse-Yen Yang
Tsung-Lin Yang
Wancai Yang
Wanqin Yang
Wei Yang
Wei-Hsiung Yang
Weili Yang

Wenhui Yang
Wenlong Yang
Wenxing Yang
Wen-Yi Yang
Wenyu Yang
Wu Yang
Wuyun Yang
X. Frank Yang
Xia Yang
Xiangling Yang
Xiao Yang
Xiaobao Yang
Xiaodong Yang
Xiaofan Yang
Xiaoju Yang
XiaoLi Yang
Xiaoyang Yang
Xiaoying Yang
Xin Yang
Xing-Ke Yang
Xinrong Yang
Xinsong Yang
Xiongfeng Yang
Xue Yang
Xuejun Yang
Xueli Yang
Xuerui Yang
Xuesong Yang
Xufei Yang
Y. Tony Yang
Yajun Yang
Yan Yang
Yang Yang
Yanjie Yang
Yanli Yang
Yanqi Yang
Ye Yang
Yi Yang
Yi-Hsin Yang
Yihua Yang
Yi-Ling Yang
Yilong Yang
Yi-Mei (Amy) Yang
Ying Yang
Yingjie Yang
Ying-Ying Yang
Yi-Ning Yang
Yi-Qing Yang
Yisheng Yang
Yixian Yang
Yong Yang
Yongchao Yang
Yu Yang
Yuan Yang
Yuchen Yang
Yuedong Yang
Yufeng Yang
Yun Yang
Yungjen Yang
Yunjun Yang
Yu-Sheng Yang
Yushi Yang
Yutao Yang
Yuting Yang
Zequan Yang
Zhen Yang
Zhenhua Yang
Zhibo Yang
Zhihao Yang
Zhongxia Yang
Zi-Feng Yang
Zili Yang
Ziyin Yang
Zu-Jun Yang
Elizabeth Yanik
Emre Yanikkerem
Thomas Yankee
Gustavo Yannarelli
Akira Yano
Ikuko Yano
Ryoichi Yano
Philip Yanos
Svetlana Yanushkevich
Zhang Yanxiao
Baoli Yao
Bing Yao
Bo Yao
Guorui Yao

Hiroshi Yao
Honghong Yao
Hongwei Yao
Hong-Wu Yao
Jia Yao
Jing Yao
Justin Yao
Ke Yao
Li Yao
Liming Yao
Lingling Yao
Linong Yao
Lixia Yao
Minjie Yao
Qiang Yao
Qizhi Yao
Shanguo Yao
Shuqiao Yao
Song Yao
Wei Yao
Weiwu Yao
Xiaojun Yao
Xiaoqiang Yao
Xiaoxi Yao
Xincheng Yao
Xin-Qiu Yao
Xiongliang Yao
Yan Yao
Ye Yao
Yingyin Yao
Yong-Ming Yao
Yuangen Yao
Yucheng Yao
Yuchun Yao
Zhi Yao
Chee Kong Yap
Hong Kai Yap
Roseline Yap
Chatu Yapa
Manisha Yapa
Hakan Yarali
Gorkem Yararbas
Oded Yarden
Neil Yarlett
N. Yarpuz-Bozdogan
Anthony Yartel
Joanne Yarwood
Zimri Yaseen
Erika Yashiro
Masakazu Yashiro
Shigeko Yashiro
Yoko Yashiroda
Ifat Yasin
Amber Yasmeen
Rubina Yasmin
Sumera Yasmin
Annalee Yassi
Nawaf Yassi
Aymen Yassin
Hideto Yasuda
Hitoshi Yasuda
Shinsuke Yasuda
Yuichiro Yasuda
Shingo Yasuhara
Kyuichi Yasui
Teruhito Yasuia
Motoshige Yasuike
Yuji Yasukochi
Misato Yasumura
Fumihiko Yasuno
Akihito Yasuoka
Hidekata Yasuoka
Akiba Yasutada
Koji Yasutomo
Midori Yatabe
Clayton Yates
Dustin Tyler Yates
Jennifer Yates
Julia Yates
Oliver Yates
Andrii Yatskul
Hiroshi Yatsuhashi
Shu Yau
Stephen Yau
Young Yauger
Duran Yavuz
Metin Yavuz
Ali Yawar

Kursat Yaykasli
Seyhan Yazar
Pascal Yazbeck
Iman Yazdi
Mark Yazer
Ron Ydenberg
An Ye
Changyun Ye
Chuyang Ye
Cunqi Ye
Ding-Wei Ye
Guodong Ye
Haihui Ye
Han-Jia Ye
Huihui Ye
Jiangbin Ye
Jun Ye
Kaixiong Ye
Li Ye
Lin Ye
Liu Ye
Min Ye
Minzhi Ye
Qingsong Ye
Rongzhong Ye
Xiao Qi Ye
Xiaofang Ye
Xin Ye
Xinyue Ye
Xuehua Ye
Maurice Yeadon
David Yeager
Justin Yeager
Kevin Yeager
Sakiko Yeagshi
Bu Yeap
Mohammed Yeasin
Sara Yeatman
Andrew Yee
Anne Yee
Jennifer Yee
Lynn Yee
Marianne Yee
Herman Yeger
Benjamin Yeh
Chau-Ting Yeh
Chih-Jung Yeh
Chun Chieh Yeh
Chun-I Yeh
Elizabeth Yeh
Emily Yeh
Fang-Cheng Yeh
Jui-Feng Yeh
Kai-Wun Yeh
Kun-Yun Yeh
Kuo-Chen Yeh
Kuo-Wei Yeh
Ming-Lun Yeh
Shean-Ya Yeh
Shuyuan Yeh
Sonia Yeh
Yi-Ting Yeh
Yung-Hsin Yeh
Zohar Yehoshua
Nadir Yehya
Sowmya Yelamanchili
Sailu Yellaboina
Daniel Yelle
Chandra Yelleswarapu
Steven Yellon
B. Linju Yen
David H. T. Yen
Jian Yen
Laurann Yen
Sheng-Che Yen
Shen-Horn Yen
Tzu-Chi Yen
Tzung-Hai Yen
Wen-Jiuan Yen
Yi-Hao Yen
Veerasamy Yengopal
Yener Yeni
Jenna Yentes
Leonard L. L. Yeo
Sun Kok Yeo
Aditya M. Yeolekar
Seon-In Yeom
Carl Yeoman

W. Y. Yeong
Gloria Yepiz-Plascencia
Helene Yera
Venkata Yeramilli
Justin Yerbury
Kowiyou Yessoufou
Marc Yeste
Meliha Yetişgen
Chi Yeung
Nick Yeung
Polly Yeung
Sai-Ching Yeung
Susanna Yeung
Andrew Yew
Joanne Yew
Bin Yi
Daqing Yi
Darvin Yi
Eunhee Yi
Fei Yi
Gi-Hwan Yi
Hankuil Yi
Hongliang Yi
Hongtao Yi
Hongyu Yi
Huiyu Yi
Jing Yi
Jumi Yi
Juneho Yi
Li Yi
Meisheng Yi
Ping Yi
Shuhua Yi
Siyan Yi
So Young Yi
Tao Yi
Xianwen Yi
Xin Yi
Yugen Yi
Yu-Jun Yi
Zhenghui Yi
Kit Yi Yam
Eugenia Yiannakopoulou
Lo Yi-Chen
Sun Yifan
Arda Yildirim
Eda Yildirim
Funda Yildirim
Metin Yildirim
Pinar Yildirim
Kubilay Yıldırım
H. Yildirimerbil
Akar Yilmaz
Ali Yilmaz
Enver Demirel Yilmaz
Ilker Yilmaz
Mustafa Yilmaz
Serhat Yilmaz
Tuba Yilmaz-Savas
Jae-Joon Yim
Mark Yim
Cameron Yin
Changjun Yin
Chaoran Yin
Chunyue Yin
Dwight Yin
Erwei Yin
Gavin Yin
Guo Yin
Guohua Yin
Hong Yin
Jia Yin
Jianbo Yin
Jieyun Yin
Jingjing Yin
Jinlong Yin
Junjun Yin
Kaifeng Yin
Kedong Yin
Lihong Yin
Lu Yin
Meng Yin
Michael Yin
Rui Yin
Ruichuan Yin
Viravuth Yin
Wei-Hsian Yin
Xian-Yong Yin

Xiaojian Yin
Xin Yin
Xue-Ren Yin
Yanbin Yin
Yanhai Yin
Yian Yin
Yulong Yin
Zhimin Yin
Gui-Shuang Ying
Shihui Ying
Tiejin Ying
Wen Ying
Zhekang Ying
John Yinger
Benjamin Hon Kei Yip
Connie Yip
Linwah Yip
Paul S. F. Yip
Rowena Yip
Tsz Leung Yip
Kesetebirhan Yirdaw
Sean Yiu
Olavi Ylikorkala
Tapati Yli-Mattila
Gina Ylitalo
Arne Yndestad
Nigel Yoccoz
Anne Yoder
Jonathan Yoder
Matthew Yoder
Paul Yoder
Supachai Yodkeelee
Michael Yodzis
Ram Yogev
Sileshi Yohannes
Raymond Yokomi
Hiroshi Yokomichi
Kyoko Yokomori
Edna Yokoo
Takashi Yokoo
Takeshi Yokoo
Toshifumi Yokota
Hitoshi Yokoyama
Kenichi Yokoyama
Shinji Yokoyama
Bethany Yon
Mark Yondola
Yoshihiro Yoneda
Taishi Yonetsu
Eu-Leong Yong
Ge Yong
Jeongsik Yong
Jia Ying Yong
V. Wee Yong
Dongwan Yoo
Eun-Hye Yoo
Jung-Yoon Yoo
Jun-Il Yoo
Roh-Eul Yoo
Sang-Dong Yoo
Seung-Hee Yoo
Tae-Hyun Yoo
Young Chul Yoo
Yun Joo Yoo
Do-Kun Yoon
Hyun-Kyu Yoon
In-Young Yoon
Kyung Chul Yoon
Mee-Sup Yoon
Minn Yoon
Sangpil Yoon
Soweon Yoon
Sung Yoon
Tae-Young Yoon
Woong Yoon
Yisang Yoon
Young Yoon
Shibu Yooseph
Boyan Yordanov
Mark Yorek
Ian York
Jessica Yorzinski
Goro Yoshida
Hiroki Yoshida
Hiroshi Yoshida
Masaki Yoshida
Masayuki Yoshida
Matsunori Yoshida

Naoto Yoshida
Ruriko Yoshida
Ryusuke Yoshida
Shigetaka Yoshida
Shigeto Yoshida
Shiomi Yoshida
Shuzo Yoshida
Tadashi Yoshida
Takuya Yoshida
Atsuo Yoshido
Hajime Yoshifuji
Fumiki Yoshihara
Hiroki Yoshihara
Akiomi Yoshihisa
Tohru Yoshihisa
Akira Yoshii
Fumihito Yoshii
Eisho Yoshikawa
Tetsushi Yoshikawa
Koichi Yoshimasu
Koichi Yoshimura
Michihiro Yoshimura
Reiji Yoshimura
Takashi Yoshimura
Yukinori Yoshimura
Christine Yoshinaga-Itano
Jun Yoshino
Jun Yoshioka
Ken-Ichi Yoshioka
Yosuke Yoshioka
Kohei Yoshiyama
Ayumi Yoshizaki
Tomokazu Yoshizaki
Ryota Yoshizawa
Christian Yost
Christopher Yost
Russell Yost
William Yost
Ihor Yosypiv
Thewjitcharoen Yotsapon
Hiroshi Yotsuyanagi
Chun You
Hong You
Hyung-Keun You
Jane You
Je Sung You
Jingjing You
Jinsheng You
Liangzhi You
M. You
Mingxu You
Shengyi You
Sungeun You
Sylvaine You
Weiwei You
Xinxin You
Zhongsheng You
Ada Youk
Daniel Youkee
Adel Youkhana
Chun Youn
Hyewon Youn
Mehwish Younas
Nadia Younes
Mauricio Younes-Ibrahim
Aaron Young
Adrienne Young
Benjamin Young
Deborah Young
Erica Young
G. Bryan Young
Heather Young
Henry Young
Ian Young
Jamey Young
Jean-Gabriel Young
Jodi Young
Kelly Young
Kymberly Young
Marian Young
Mary Young
Melissa Young
Michael Young
Nicolas Young
Richard Young
Robert Young
Robin Young
Shuenn-Tsong Young

Simon Young
Steven Young
Vincent Young
James A. Youngclaus
Jane Younger
Jarred Younger
Brett Younginger
Robert Youngquist
Reef Youngreen
Shawn Youngstedt
Kathryn Yount
Gregory Yourek
Shatha Yousef
George Youssef
Noha Youssef
Jan Youtie
Yossi Yovel
Laetitia Yperzeele
Tomas Ysehak Abay
Lidia Yshii
Hans Yssel
Alan Yu
Alfred Yu
Bailang Yu
Bin Yu
Bing Yu
Cai-Guo Yu
Chack-Yung Yu
Chang Yu
Charles Yu
Chin Sheng Yu
Chong Yu
Dahui Yu
Dan Yu
Daohai Yu
Dazhao Yu
Fangyuan Yu
Fei-Hai Yu
Fengqun Yu
François Yu
Guanghui Yu
Guang-Yan Yu
Guann-Yi Yu
Guihai Yu
Guojun Yu
Guoqin Yu
Hai Yu
Haibo Yu
Han-Gang Yu
Hon Yu
Hong Yu
Hui Yu
Huilong Yu
Hyeong Gon Yu
Jack Yu
James Yu
Jane Yu
Jianming Yu
Jieh-Juen Yu
Ji-Guo Yu
Jinghua Yu
Jingquan Yu
Jinming Yu
Jinpu Yu
Jinsheng Yu
Jiujiang Yu
Kai Yu
Kaixian Yu
Ke-Da Yu
Keming Yu
Lean Yu
Lehua Yu
Li Yu
Lianchun Yu
Lin Yu
Liping Yu
Liuqian Yu
Lu Yu
Luis Yu
Maoqun Yu
Mei Yu
Meichen Yu
Ming Yu
Ming-Jiun Yu
Paul Yu
Pei-Lin Yu
Peng Yu
Pengchun Yu

Peter Yu
Qigui Yu
Qili Yu
Qing Yu
Qingbao Yu
Qiujun Yu
Rina Yu
Rong Yu
Rwei-Ling Yu
S. W. Yu
Seong-Jin Yu
Shiqi Yu
Shun Yu
Shuo Yu
Simin Yu
Su Jong Yu
Sue Yu
Tianxin Yu
Tinghe Yu
Wei Yu
Weihui Yu
Weijian Yu
Wen-Kuang Yu
Wenkui Yu
Wenli Yu
Wenwu Yu
Xiang Yu
Xiaolin Yu
Xiaona Yu
Xiaowen Yu
Xiezhi Yu
Xin Yu
Xinghuo Yu
Xinhua Yu
Xiping Yu
Yan Yu
Yang Yu
Yanjie Yu
Yao Yu
Ye Yu
Yen-Rei Yu
Yi Yu
Yingpu Yu
Yinxian Yu
Yong Yu
Yunqiu Yu
Yu-Tian Yu
Zhanyang Yu
Zhaoxia Yu
Zhichao Yu
Zhiguo Yu
Zhiming Yu
Zhonghai Yu
Ziniu Yu
Zitong Yu
Bo Yuan
C. Yuan
Changzheng Yuan
Christina Yuan
Chunyan Yuan
Daojun Yuan
Gonglin Yuan
Guoyong Yuan
Hua Yuan
Jason X. J. Yuan
Jiahai Yuan
Jiazheng Yuan
Jichao Yuan
Jing Yuan
Jingsong Yuan
Junhua Yuan
Kai Yuan
Kuo-Ching Yuan
Lihua Yuan
Lijuan Yuan
Lin Yuan
Mengting Yuan
Mengyao Yuan
Ming Yuan
Ming-Long Yuan
Mingwei Yuan
Ning Yuan
Pu-Qing Yuan
Quan Yuan
Songtao Yuan
Tian Yuan
Ting Yuan
Wenjie Yuan

Xiaochen Yuan
Xiaofang Yuan
Xu Yuan
Xue Yuan
Y. Yuan
Yan Yuan
Ying Yuan
Yuan Yuan
Zengqiang Yuan
Zhaokang Yuan
Zheng Yuan
Xie Yuansheng
Elena Yubero-Serrano
Kuaybe Yucebilgili Kurtoglu
Gönenç Yücel
Can A. Yucesoy
Chengyan Yue
Feng Yue
Hong Yue
Jia-Xing Yue
Jinhuan Yue
Jun Yue
Junming Yue
Patrick Ying-Kit Yue
Qiang Yue
Yujuan Yue
Yun Yue
Chau Yuen
Courtney Yuen
Kevin Kam Fung Yuen
Kevin Yuen
Kit-San Yuen
Shao Yu-Hsuan Joni
Jae-Min Yuk
Vyacheslav Yukalov
Michi Yukawa
Koichi Yuki
Ihsan Yüksel
Shirley Yuk-Wah Liu
Yen Na Yum
Dmitry Yumashev
Futoshi Yumoto
Wako Yumura
Bo Hyon Yun
Eun Ju Yun
Jong Won Yun
Mi Ja Yun
Sook Yun
Tae Jin Yun
Unil Yun
Yeoheung Yun
Chee Fu Yung
Mingo Yung
Mikihiro Yunoki
Fakir Yunus
Raudah Yunus
Alison Yurasek
Stanislav Yurchenko
Natalia Yurlova
Andrew Yurochko
Bülent Yurtlu
Sadık Yurttutan
Mohd Yusof
Stuart Yuspa
Jason Yustein
Erlangga Yusuf
Mohd Yusuf
Nabiha Yusuf
Afzalhussein Yusufali
Katherine Yutzey
Cynthia Yu-Wai-Man
Vilma Yuzbasiyan-Gurkan
Mustafa Yuzukirmizi
Muhammad Z. Ihsan
Hans Zaaijer
Jabi Zabala
Mark Zabel
Darya L. Zabelina
Piotr Zabielski
Nadja Zaborsky
Germana Zaccagnini
Michele Zaccai
Francesco Zaccardi
Serena Zacchigna
Manuela Zaccolo
Georges Zaccour
Evelyn Zacharewicz
Rony Zachariah

Anita Zacharias
Martin Zacharias
Kai Zacharowski
Christopher Zachary
Frank Zachos
Nicholas Zachos
Christin Zachow
Stefan Zachow
Donald Zack
Martin Zack
Shawn Zack
James Zacny
Ruth Zadoks
Ernő Zádor
Sabrina Zadrozny
Kurt S. Zaenker
Romi Zaeske
Sherin Zafar
Syed Nabeel Zafar
Stéphane Zaffran
Alexey Zagalsky
Soroush Zaghi
Kareem Zaghloul
Maja Zagmajster
Anderson Zago
Matteo Zago
Monique Zagorec
Maxim Zagoskin
Marta Zagrebelsky
Hisamitsu Zaha
Jean-Ralph Zahar
Norzarina Zaharim
Camila Zahdi
Zafar Zaheer
Muhammad Zahid
Sandrine Zahn
Benjamin Zahneisen
James Zahniser
Natalie Zahr
Thomas Zahrt
Joseph Zaia
Dahlia Zaidel
Habib Zaidi
Irfan Zaidi
Mussaret Zaidi
Shehla Zaidi
Syed Zaidi
Alexander Zaika
Alexey Zaikin
Kayvan Zainabadi
Awang Zainudin
Dietmar Zaiss
Nadia Zakaria
Philip Zakas
Dan Zakay
Zichria Zakay-Rones
Bijan Zakeri
Sedigheh Zakeri
Eleonora Zakharian
Wei Zakharov
Ibrahim Zakhary
Hasan Zaki
Noha Zaki
Tanya Zakrison
Ahmed Zaky
Samer Zaky
Bernard Zalc
Anna Zalewska
Nickolas Zaller
Charles Zaloudek
Myron Zalucki
Nadia Zalunardo
Alberuni Zamah
Yoshito Zamami
Khalequ Zaman
Hasniza Zaman Huri
Nasim Zamani
Fereshteh Zamani Alavijeh
Roham Zamanian
Mona Zamanian Azodi
Marta Zamarbide
Federico Zambon
Giovanni Zambon
Alessandra Zambonelli
Paolo Zamboni
Michal Zámečník
Eyal Zamir
Welder Zamoner

Ruben Zamora
Francisco Javier Zamora-Camacho
Raul Zamora-Ros
Paola Zamparo
Anna Zampetaki
Fernando Zampieri
Sandro Zampieri
Aleksander Zampronio
Antonio Zamunér
Scott Zamvil
Andrey Zamyatnin
Kendra Zamzow
Lin-Sen Zan
Thais Zanata
Elodie Zana-Taieb
Rayssa Zanatta
Anouk Zancarini
Nilson Zanchin
Meet Zandawala
Nicole Zander
Roya Zandi
Anthony Zanesco
Damián Zanette
Regis Zanette
Chunpeng Zang
Xiaonan Zang
David Zangen
Shahab Zangeneh
Márcio Zangeronimo
Heidi A. Zangi
Massimiliano Zaniboni
Fabio Zanini
Caroline Zank
Anthony S. Zannas
Khatun Zannat
Ginevra Zanni
Markella Zanni
Romano Zannoli
Gian Franco Zannoni
Arianna Zanolini
Luisa Zanolla
Marco Zanon
Ivan Zanoni
Daniel Capella Zanotta
Ilaria Zanotti
A. Zanotto-Filho
Alfeu Zanotto-Filho
Nicholas Zaorsky
Lucia Zappalà
Antonio Zapparata
Filippo Zappasodi
Martina Zappaterra
Fadi Zaraket
Hassan Zaraket
Xristo Zarate
C. Zarcadoolas
Maria Zarcone
Gerardo Zardi
Pauline Zardo
Homeira Zardooz
Habil Zare
Wojciech Zareba
Nicholas Zareifopoulos
Kamran Zargar-Shoshtari
Jose Zariffa
Wasifa Zarin
Rasa Zarnegar
Daniela Zarnescu
Kelly Zarnke
Paul Zarogoulidis
Sofia Zarraga
Arash Zarrine-Afsar
Ali Zarrinpar
Olfa Zarrouk
Marketa Zarybnicka
Manuel Zarzoso
Rebecca Zash
Alan Zaslavsky
Jason Zastre
Robert Zatorre
Mayana Zatz
Roberto Zatz
Egija Zaura
Andreas Zautner
Daniele Zavagno
Ernesto Alejandro Zavala Gonzalez
María Zavala-Paramo
Silva Zavarsek

Elizabeth Zavitz
Yana Zavros
Mark Zaydman
Amro Zayed
Yekaterina Zaytseva
Antonio Zaza
Zachery Zbinden
Maciej Zborowski
Jan Zdarek
Christina Zdenek
Ivan Zderic
Steve Zderic
Nevijo Zdolec
Eftim Zdravevski
Sherali Zeadally
Bernie Zebarth
Qendrim Zebeli
Matthias Zebisch
Lynn Zechiedrich
Stefan Zechmann
Guenther Zeck
Melinda Zeder
Aglaia Zedlitz
Marcel Zeelenberg
Luiz Zeferino
Assefa Zegeye
Ellen Zegura
Ingeborg Zehbe
Christoph Zehe
Gianguglielmo Zehender
Margit Zeher
Alan Zehnder
James Zehnder
Sarah Zehr
Martin Zeier
Robert Zeiger
Adam Zeilinger
Katrin Zeilinger
Stefan Zeisberger
Benjamin Zeitlin
Jennifer Zeitlin
Scott Zeitlin
Christina Zeitz
Bleranda Zeka
Amir Zeki
Abdel-Rahman Zekri
Howard Zelaznik
Renáta Zeleníková
Asta Zelenkauskaite
John Zelenski
Karl Zelik
Tomas Zelinka
Erin Zelinski
Sara Zella
Dirk Zeller
Riley Zeller-Townson
Johannes Zellner
Jonathan Zelner
Jessica Zelt
Nadja Zeltner
Lori Zeltser
Cedric Zeltz
Elazar Zelzer
David Zeman
Edith Zemanick
Olivier Zemb
Marian Zembala
Rostislav Zemek
Erika Zemkova
Janos Zempleni
Franck Zenasni
Nardia Zendarski
Benjamin Zendel
An Zeng
Chen Zeng
Chunhua Zeng
Fangqin Zeng
Guanwen Zeng
Gucheng Zeng
Hui Zeng
Jincheng Zeng
Linghe Zeng
Ling-Li Zeng
Liping Zeng
Ming Zeng
Ruochen Zeng
Shaohua Zeng
Sijin Zeng

Tieyong Zeng
Wei Zeng
Xi Zeng
Xiang Jun Zeng
Xian-Tao Zeng
Xiaofeng Zeng
Xuhui Zeng
Yawen Zeng
Yong Zeng
Yukai Zeng
Zhen Zeng
Zhenling Zeng
Zhenshun Zeng Zeng
Zhenxiang Zeng
Ziqiang Zeng
Olga Zeni
Yoshitaka Zenke
Giselle Zenker
Marcos Zenobi
Juergen Zentek
Karen Zentgraf
Lorena Zentilin
Gabriel Zentner
Marcel Zentner
Patrizia Zeppegno
Gadi Zerach
Valerio Zerbi
Paolo Zerbinati
Andrea Zerboni
Yacob Zereyesus
Paula Zermoglio
Magdalena Zernicka-Goetz
Ron Zernicke
Colin A. Zestcott
Henrik Zetterberg
Madeleine Zetterberg
Kirstin Zettlitz
Holger Zetzsche
Jose Zevallos
Maximilian Zeyda
Emil Zeynalov
Annette Zeyner
Janice Zgibor
Tianshan Zha
Yong Zha
Maxat Zhabagin
Olena Zhabenko
Peiyong Zhai
Weiwei Zhai
Bin Zhan
Le Zhan
Liang Zhan
Wenbin Zhan
Xiang Zhan
Xiangjiang Zhan
Xianyuan Zhan
Aidong Zhang
Anru Zhang
Aston Zhang
Bangshan Zhang
Baochang Zhang
Baoshan Zhang
Bei Zhang
Ben-Gang Zhang
Benxi Zhang
Bin Zhang
Bingjian Zhang
Bo Zhang
Bowen Zhang
C. Zhang
Caiguo Zhang
Changlin Zhang
Changqing Zhang
Chao Zhang
Chen Zhang
Cheng Zhang
Chengcheng Zhang
Chengfei Zhang
Chengjun Zhang
Chengxian Zhang
Chengxin Zhang
Chengyuan Zhang
Chengzhi Zhang
Chenming Zhang
Chenwang Zhang
Chi Zhang
Chong Zhang
Chun-Ting Zhang

Chunxia Zhang
Chunyan Zhang
Chun-Yang Zhang
Claire Shuiqing Zhang
Da Zhang
Dabao Zhang
Dabing Zhang
Dadong Zhang
Dan Zhang
Dao-Qi Zhang
Dawei Zhang
Dayu Zhang
Defu Zhang
Degan Zhang
Dekui Zhang
De-Liang Zhang
Delong Zhang
Deqiang Zhang
Dezhi Zhang
Dianchang Zhang
Dingwen Zhang
Dong Zhang
Dongqing Zhang
Dong-Yan Zhang
Donna Zhang
E. Zhang
Fan Zhang
Fang Zhang
Fangyuan Zhang
Feng Zhang
Fujun Zhang
Ganwei Zhang
Ge Zhang
Gengxin Zhang
Gong Zhang
Guangyan Zhang
Gui Zhang
Guimin Zhang
Guo Zhang
Guo-Chang Zhang
Guohui Zhang
Guolong Zhang
Guoqiang Zhang
Guoquan Zhang
Guosheng Zhang
Guoxin Zhang
Guo-Zhong Zhang
H. Zhang
Haifeng Zhang
Haijie Zhang
Haijun Zhang
Hai-Ping Zhang
Haitao Zhang
Hai-Tao Zhang
Han Zhang
Hanye Zhang
Hao Zhang
Henggui Zhang
Heng-Mu Zhang
Hengwei Zhang
Hong Zhang
Hongbo Zhang
Hongcai Zhang
Honghai Zhang
Hongsheng Zhang
Hongxia Zhang
Hongyan Zhang
Hu Zhang
Huaguang Zhang
Hui Zhang
Huijie Zhang
Hui-Min Zhang
Ji Zhang
Jiachao Zhang
Jian Zhang
Jianbin Zhang
Jiandong Zhang
Jian-Feng Zhang
Jiang-Hua Zhang
Jianguo Zhang
Jianlei Zhang
Jianli Zhang
Jianmin Zhang
Jianming Zhang
Jianqi Zhang
Jianxiong Zhang
Jianzhen Zhang
Jiaquan Zhang

Jiaxing Zhang
Jie Zhang
Jin Zhang
Jin-An Zhang
Jindong Zhang
Jinfa Zhang
Jing Jing Zhang
Jing Zhang
Jingchen Zhang
Jingjing Zhang
Jingyang Zhang
Jingyu Zhang
Jin-Lin Zhang
Jinling Zhang
Jinpeng Zhang
Jinsong Zhang
Jinwei Zhang
Jiqian Zhang
Jisen Zhang
Jiwang Zhang
Jiyu Zhang
Juan Zhang
Jue Zhang
Jun Zhang
Junfang Zhang
Junjie Zhang
Junli Zhang
Junling Zhang
Junpeng Zhang
Kai Zhang
Kang Zhang
Ke Zhang
Kebin Zhang
Ke-Ying Zhang
Kui Zhang
Kunpeng Zhang
Kunpu Zhang
Kunyan Zhang
Laichang Zhang
Lei Zhang
Li Zhang
Liang Zhang
Liangliang Zhang
Liangsheng Zhang
Liangxiao Zhang
Libiao Zhang
Lijun Zhang
Limao Zhang
Limin Zhang
Lin Zhang
Ling Zhang
Lingjun Zhang
Linjun Zhang
Linxia Zhang
Liqin Zhang
Lisha Zhang
Long Jiang Zhang
Long-Wa Zhang
Longxian Zhang
Lu Zhang
Luqing Zhang
Mei Zhang
Meiling Zhang
Meng Zhang
Meng-Chen Zhang
Mengxi Zhang
Miao Zhang
Miaomiao Zhang
Mike Zhang
Mingfang Zhang
Mingli Zhang
Ming-Li Zhang
Mingxi Zhang
Mingyong Zhang
Mingzhen Zhang
Min-Ling Zhang
Naixia Zhang
Nan Zhang
Ning Zhang
Peijun Zhang
Peng Zhang
Ping Zhang
Qi Zhang
Qian Zhang
Qiang Zhang
Qibo Zhang
Qing Zhang
Qingfu Zhang

Qingguang Zhang
Qing-He Zhang
Qingke Zhang
Qingpeng Zhang
Qingrun Zhang
Qing-Shuo Zhang
Qingyang Zhang
Qingying Zhang
Qinli Zhang
Qiong Zhang
Qisheng Zhang
Qiwei Zhang
Quan Zhang
Quanwei Zhang
Renyi Zhang
Ri-Jun Zhang
Rong Zhang
Rongxiao Zhang
Rui Zhang
Runlin Zhang
Sanliang Zhang
Sarah Zhang
Shanshan Zhang
Shao-Ling Zhang
Shao-Qing Zhang
Shaoting Zhang
Shaowu Zhang
Shenggui Zhang
Shenghong Zhang
Shengwei Zhang
Shi-Hong Zhang
Shihua Zhang
Shijun Zhang
Shiting Zhang
Shiyan Zhang
Shuang Zhang
Shu-Bin Zhang
Shuhua Zhang
Shuxu Zhang
Sichen Zhang
Siqi Zhang
Song Zhang
Songning Zhang
Songying Zhang
Suiqi Zhang
Tao Zhang
Tiantao Zhang
Tianyu Zhang
Tiejun Zhang
Tiemei Zhang
Tingting Zhang
Tong Zhang
Wangfeng Zhang
Wei Zhang
Weidong Zhang
Weihua Zhang
Weiping Zhang
Weishan Zhang
Wen Zhang
Wenhui Zhang
Wenjun Zhang
Wenli Zhang
Wenqing Zhang
Wenyan Zhang
Wenyi Zhang
Xi Zhang
Xian Zhang
Xiangning Zhang
Xiao Zhang
Xiaobo Zhang
Xiaofan Zhang
Xiaoge Zhang
Xiaoguang Zhang
Xiaohong Zhang
Xiaohui Zhang
Xiaojun Zhang
Xiaoke Zhang
Xiaoli Zhang
Xiaolin Zhang
Xiaoling Zhang
Xiaomin Zhang
Xiaoming Zhang
Xiaotong Zhang
Xiaowu Zhang
Xiaoying Zhang
Xiaoyong Zhang
Xiao-Yu Zhang
Xifeng Zhang

Xin Zhang
Xin Zhong Zhang
Xingyi Zhang
Xinhua Zhang
Xinming Zhang
Xinping Zhang
Xiongqing Zhang
Xiquan Zhang
Xiufeng Zhang
Xiu-Hong Zhang
Xiujun Zhang
Xiuqing Zhang
Xiuwei Zhang
Xiying Zhang
Xiyun Zhang
Xu Zhang
Xuanping Zhang
Xuemei Zhang
Xue-Song Zhang
Xuewu Zhang
Xue-Xian Zhang
Xueying Zhang
Xueyong Zhang
Xu-Xiang Zhang
Yajie Zhang
Yajun Zhang
Yalin Zhang
Yan Zhang
Ya-Nan Zhang
Yanbo Zhang
Yang Zhang
Yangsong Zhang
Yani Zhang
Yanjin Zhang
Yan-Lin Zhang
Yanna Zhang
Yanqiong Zhang
Yanxian Zhang
Yao Zhang
Yaoping Zhang
Yaou Zhang
Yaxu Zhang
Ye Zhang
Yelin Zhang
Yi Zhang
Yichao Zhang
Yi-Fan Zhang
Yifeng Zhang
Yihui Zhang
Yi-Min Zhang
Yingying Zhang
Yingze Zhang
Yisi Zhang
Yiyi Zhang
Yiying Zhang
Yiyue Zhang
Yong Zhang
Yongfa Zhang
Yonggang Zhang
Yong-Guo Zhang
Yongjie Zhang
Yong-Jie Zhang
Yong-Jun Zhang
Yongli Zhang
Yongliang Zhang
Yong-Mei Zhang
Yongqiang Zhang
Yongrong Zhang
You Zhang
Youzhong Zhang
Yu Zhang
Yuan Zhang
Yuanming Zhang
Yu-Dong Zhang
Yue Zhang
Yue-Miao Zhang
Yuhang Zhang
Yu-Jing Zhang
Yulong Zhang
Yumin Zhang
Yun Zhang
Yunhai Zhang
Yunqi Zhang
Yunqing Zhang
Yunqiu Zhang
Yuqiu Zhang
Yuxiang Zhang
Yu-Xiang Zhang

Yuyang Zhang
Zengqiang Zhang
Zhanyuan Zhang
Zhao Zhang
Zhaoqi Zhang
Zhaowei Zhang
Zhaoyun Zhang
Zhe Zhang
Zhen Zhang
Zheng Zhang
Zhengbin Zhang
Zhengfeng Zhang
Zhengsheng Zhang
Zhenguo Zhang
Zhengwang Zhang
Zhengwei Zhang
Zhengzhi Zhang
Zhenhua Zhang
Zhenhuan Zhang
Zhen-Lin Zhang
Zhenming Zhang
Zhenqiu Zhang
Zhenyu Zhang
Zheshen Zhang
Zhi Jie Zhang
Zhibin Zhang
Zhi-Gang Zhang
Zhihao Zhang
Zhihong Zhang
Zhiren Zhang
Zhiyong Zhang
Zhongheng Zhang
Zhongnan Zhang
Zhong-Yuan Zhang
Zhou Zhang
Zhu Zhang
Zhu-Ming Zhang
Ziding Zhang
Zi-Jian Zhang
Ziping Zhang
Ziqiang Zhang
Zong-Hong Zhang
Zugui Zhang
Zuxin Zhang
Amy Zhao
Aqun Zhao
Ben Zhao
Bingzi Zhao
Bo-Wen Zhao
Chao Zhao
Chenchen Zhao
Chengyi Zhao
Chunhui Zhao
Chunjiang Zhao
Chunlin Zhao
Da Zhao
Dawei Zhao
Dayong Zhao
Dejian Zhao
Di Zhao
Fangqing Zhao
Fei-Fei Zhao
Fengdong Zhao
Feng-Qi Zhao
Feng-Qin Zhao
Gang Zhao
Guanghui Zhao
Guangyao Zhao
Guifang Zhao
Haijun Zhao
Hailin Zhao
Haitao Zhao
Han Zhao
Hang Zhao
Helen Zhao
Hong Zhao
Hong-Mei Zhao
Hongwei Zhao
Hongyong Zhao
Hua Zhao
Huaqiang Zhao
Huiying Zhao
Jiangqin Zhao
Jichang Zhao
Jichao Zhao
Jie Zhao
Jin Zhao
Jincun Zhao

Jingjing Zhao
Jinling Zhao
Jinzhuo Zhao
Junfei Zhao
Junqiao Zhao
Kai Zhao
Kaiyu Zhao
Le Zhao
Lei Zhao
Linguo Zhao
Lingzhi Zhao
Min Zhao
Ming Zhao
Mingbo Zhao
Mingwei Zhao
Nai-Qing Zhao
Ni Zhao
Peng Zhao
Ping Zhao
Qi Zhao
Qinjian Zhao
Qinshi Zhao
Rui Zhao
Shiguang Zhao
Shiteng Zhao
Shuhe Zhao
Sihai Zhao
Sumei Zhao
Ting C. Zhao
Tong Zhao
Tongbiao Zhao
Tongyan Zhao
Wei Zhao
Weian Zhao
Weiguo Zhao
Xiangyu Zhao
Xiaoli Zhao
Xiaoshan Zhao
Xin Zhao
Xincheng Zhao
Xing Zhao
Xingbo Zhao
Xingquan Zhao
Xinming Zhao
Xinqing Zhao
Xinyu Zhao
Xinzhi Zhao
Yan Zhao
Yanan Zhao
Yang Zhao
Yaofeng Zhao
Yichuan Zhao
Yidan Zhao
Ying-Yong Zhao
Yong Zhao
Yu Zhao
Yuhua Zhao
Yun Zhao
Yunfeng Zhao
Yunjie Zhao
Yunpeng Zhao
Yutong Zhao
Yuzhen Zhao
Zhi-Dan Zhao
Zhijun Zhao
Zhiyong Zhao
Zhong-Qiu Zhao
Zibo Zhao
Ziming Zhao
Ziyi Zhao
Irina Zhdanova
Tetyana Zhebentyayeva
Valtcho Zheljazkov
Yong Ya Zhen
Yu Zhen
Zonglei Zhen
Bin Zheng
Bing Zheng
Binglian Zheng
Bochao Zheng
Bowen Zheng
Chuntian Zheng
Dazhi Zheng
Fang Zheng
Guang Zheng
Guo-Xing Zheng
H. Zheng
Hao Zheng

Hongchun Zheng
Hua Zheng
Huaixin Zheng
Jiang Zheng
Jiashun Zheng
Jie Zheng
Jingming Zheng
Jun Zheng
Jusheng Zheng
Lianming Zheng
Lin Zheng
Linda Zheng
Mingyue Zheng
Muhua Zheng
Nan Zheng
Pan Zheng
Qiang Zheng
Qinsi Zheng
Qipeng Zheng
Rongqin Zheng
Shengping Zheng
Shuai Zheng
Songfeng Zheng
Tianqing Zheng
Wang Zheng
Wenwen Zheng
Xiaofei Zheng
Xiaoxu Zheng
Xiaoyi Zheng
Xiaoying Zheng
Xiufen Zheng
Xu Zheng
Xudong Zheng
Xueli Zheng
Yaguang Zheng
Yalin Zheng
Yi Zheng
Yong-Ping Zheng
Yong-Tang Zheng
Yuanyi Zheng
Yu-Jun Zheng
Yulong Zheng
Yun Zheng
Zeyu Zheng
Zhe Zheng
Zhen Zheng
Zhiming Zheng
Anatoly Zhitkovich
Miglena Zhiyanski
Hu Zhiyong
Bineng Zhong
Bojian Zhong
Boxiong Zhong
Guohua Zhong
Hua Zhong
Jiangmin Zhong
Jianhui Zhong
Jilong Zhong
Jing-Quan Zhong
Lei Zhong
Min Zhong
Ning Zhong
Ping Zhong
Qu Zhong
Ray Y. Zhong
Rui Zhong
Suyu Zhong
Wei Zhong
Wenwan Zhong
Xiao-Bo Zhong
Yi Zhong
Yiwen Zhong
Yuanguang Zhong
Yueqi Zhong
Zhenhui Zhong
Bin Zhou
Bing Zhou
Bo Zhou
Changlin Zhou
Dan Zhou
Dong Zhou
Dongming Zhou
En-Min Zhou
Fang Zhou
Feng Zhou
Fengyu Zhou
Fu-Bao Zhou
Fuchun Zhou

Fusheng Zhou
Gaofeng Zhou
Guang-Biao Zhou
Guangming Zhou
Guohua Zhou
Guomo Zhou
Hai-Jun Zhou
Haiming Zhou
Haitao Zhou
Hongbo Zhou
Houcheng Zhou
Hui Zhou
Huiping Zhou
Huiyu Zhou
Jian Zhou
Jiang Zhou
Jiansong Zhou
Jian-Xin Zhou
Jie Zhou
Jiehua Zhou
Jiliang Zhou
Jing Zhou
Jinyuan Zhou
Jiyong Zhou
Ke Zhou
Kun Zhou
Lei Zhou
Li Zhou
Liang Zhou
Libin Zhou
Lihua Zhou
Lijun Zhou
Liqun Zhou
Lufang Zhou
Man Zhou
Mengchu Zhou
Mi Zhou
Ming Zhou
Mu Zhou
Nanrun Zhou
Ping Zhou
Qi Zhou
Qiao Zhou
Qifa Zhou
Qingtao Zhou
Qun-Yong Zhou
Rong Zhou
Shenglu Zhou
Shoujun Zhou
Shui-Hong Zhou
Shun-Li Zhou
Shuntai Zhou
Song-Lin Zhou
Suhong Zhou
Tao Zhou
Tianlun Zhou
Tieli Zhou
Weibin Zhou
Wenchao Zhou
Wenna Zhou
Wen-Wu Zhou
X. M. Zhou
Xiangmei Zhou
Xianju Zhou
Xiaobo Zhou
Xiaofei Zhou
Xiaohong Zhou
Xiaojie Zhou
Xiaoming Zhou
Xin Zhou
Xuyu Zhou
Yang Zhou
Yao Zhou
Yi-Hua Zhou
Yilu Zhou
Yinghui Zhou
Yinzuo Zhou
Yong Zhou
Yongquan Zhou
Yuan Zhou
Yufeng Zhou
Yuling Zhou
Yun Zhou
Yuyu Zhou
Zeping Zhou
Zhanxiang Zhou
Zhao Shen Zhou
Zhen Zhou

Zheng Zhou
Zhenjiang Zhou
Zhenming Zhou
Zhiahua Zhou
Zhigang Zhou
Zhi-Guo Zhou
Zhixiong Zhou
Zhongliang Zhou
Zhong-Shi Zhou
Zhou Zhou
Ziyuan Zhou
Galina Zhouravleva
Banghao Zhu
Bangzhu Zhu
Bo Zhu
Carolyn Zhu
Changfu Zhu
Chengsong Zhu
Chuanmei Zhu
Daochen Zhu
Fang Zhu
Fansan Zhu
Feiyun Zhu
Feng Zhu
Feng-Cai Zhu
Feng-Xiang Zhu
Fenxia Zhu
Frank Zhu
Gengping Zhu
Guangyu Zhu
Guanheng Zhu
Guohui Zhu
Guoqiang Zhu
Haijiang Zhu
Hai-Yan Zhu
Hao Zhu
Hua Zhu
Huaguo Zhu
Huilin Zhu
Jian Zhu
Jianbo Zhu
Jiang Zhu
Jian-Ming Zhu
Jia-Qiao Zhu
Jiewen Zhu
Jun Zhu
Junli Zhu
Kai Zhu
Ke Zhu
Keming Zhu
Kenny Q. Zhu
Kun Yan Zhu
Kun Zhu
Liang Zhu
Lihua Zhu
Lisha Zhu
Lu Zhu
Luchang Zhu
Luke Zhu
Meidong Zhu
Mengmeng Zhu
Mike Zhu
Min Zhu
Ning Zhu
Ningyan Zhu
P. Fei Zhu
Pengfei Zhu
Qianhao Zhu
Qian-Hao Zhu
Qing Zhu
Qiyun Zhu
Rongda Zhu
S. Zhu
Shaihong Zhu
Shengfeng Zhu
Shenyin Zhu
Shimin Zhu
Shuifang Zhu
Shusheng Zhu
Wei Zhu
Weiyun Zhu
Wenbo Zhu
Wencheng Zhu
Wenhan Zhu
Xiangru Zhu
Xiao-Dong Zhu
Xiaofeng Zhu
Xiaoping Zhu

Xiaoyuan Zhu
Xihua Zhu
Xucheng Zhu
Xun Zhu
Yajuan Zhu
Yalin Zhu
Yan Zhu
Yi Zhu
Yifan Zhu
Yimin Zhu
Yiwen Zhu
Yong Zhu
Yongchuan Zhu
Yongtao Zhu
Yongxu Zhu
Yue Zhu
Yue-Qi Zhu
Zaichun Zhu
Zexuan Zhu
Zhen Zhu
Zhigang Zhu
Zude Zhu
F. Zhuang
Jieyun Zhuang
Jun Zhuang
Lenan Zhuang
Wei Zhuang
Xiaoxi Zhuang
Xuran Zhuang
Yijie Zhuang
Yong Zhuang
Yu Zhuang
Zhihao Zhuang
Vita Zhukova
Yaroslava Zhulina
Chuanjun Zhuo
Renying Zhuo
Wei Zhuo
Yehong Zhuo
Keyan Zhu-Salzman
Zhike Zi
Roseanna N. Zia
Assem Ziady
Kazem Zibara
Lara Zibarras
Suzanna Zick
Janis Heinrich Zickfeld
Tawanda Zidenga
Alexander Ziebart
Nicolas Ziebarth
Falko Ziebert
John Ziebuhr
Douglas Ziedonis
Anette-Gabriele Ziegler
Dan Ziegler
Dylan Ziegler
Ekhard Ziegler
Maren Ziegler
Mathias Ziegler
Matthias Ziegler
Jeanette Ziehm
Barbara Zielinski
Daniel Zielinski
Jacek Zielonka
Tjalf Ziemssen
Christian Ziener
Stéphan Zientara
Manfred Zierhut
Anke Zieseniss
Brendan Zietsch
Zivile Zigaite
Lynn Zijenah
Jenny Zilberberg
Marietta Zille
Thomas Zilli
Francesca Zimetti
Jochen Zimmer
Manuel Zimmer
Sara Zimmer
Sebastian Zimmer
Andrew Zimmerman
Emily Zimmerman
Jeffrey Zimmerman
Steven Zimmerman
Albert Zimmermann
Fridolin Zimmermann
Hanna Zimmermann
Jan Zimmermann

Marita Zimmermann
Nils Zimmermann
Ralf Zimmermann
Stefan Zimmermann
Thalita Zimmermann
Teresa Zimmers
Kory Zimney
Basilia Zingarelli
Brian Zink
Martin Zinkernagel
Steven Zinn
Christoph Zinner
Julie Zinnert
Sophie Zinn-Justin
Jakob Zinsstag
Argyrios Ziogas
Justyna Ziolkowska
Natalia Ziolkowski
Peter Zioupos
Ouliana Ziouzenkova
Stephan Zipfel
Katherine Zipp
Alexander Zipprich
Danny Zipris
Marc Zirnsak
Panagiotis Zis
Hans Zischka
Devy Zisman
Ulrich Zissler
Carly Ziter
Federica Zito Marino
Emanuel Zitt
Tania Zittoun
Carolin Zitzmann
Elad Ziv
Noam Ziv
Robert Zivadinov
Dana Zive
Paul Zivich
Angela Zivkovic
Lada Zivkovic
Zorica Zivkovic
Andrey Ziyatdinov
Valentina Zizzari
Gaetano Zizzo
Milica Zlatković
Inti Zlobec
Sharon Zlochiver
Berislav Zlokovic
Jaroslaw Zmijewski
Piotr Zmijewski
Denis Zmirou-Navier
Pawel Zmora
Ariana Znaor
Chris Zobel
Gosia Zobel
Daniel Zoccal
Luca Zoccarato
D. Zochodne
Gulab Zode
Julia Zograf
Konstantina Zografou
S. Zographos
Fatemeh Zohoori
Jerome Zoidakis
Jaroslaw Zola
Janeta Zoldan
Heinz Zoller
Sue Anne Zollinger
Kevin Zollman
Frank Zöllner
Fabiana Zollo
Brian Zoltowski
Helena Zomer
Ali Zomorrodi
Marielle Zondervan-Zwijnenburg
Wei-Xing Zong
Lyonne Zonneveld
María Zonta
Sandro Zonta
Alexander Zook
Regine Zopf
Chiara Zoppiroli
Pietro Zoppoli
Pinar Zorlutuna
Erik Zornik
Michaela Zorn-Kruppa
Leonardo Zornof

Dmitry Zorov
Carmen Zorrilla
Raphael Zory
Dominique Zosso
Moises Zotti
Michela Zottini
Changfu Zou
Guanyang Zou
Haidong Zou
Haiming Zou
Huasong Zou
Jian Zou
Jun Zou
Kun Zou
Lan Zou
Ming-Hui Zou
Qiang Zou
Qingze Zou
Quan Zou
Tiefang Zou
Wei Zou
Wen Zou
Xuenong Zou
Yajie Zou
Yong Zou
Zhen Zou
Zhenhua Zou
Zhongmin Zou
Amina Zoubeidi
Mohamed Zouch
Hassane Zouhal
Rita Zrenner
Raida Zribi Zghal
Volker Zschorlich
Sandor Zsebok
Xuyu Zu
Yao Zu
Philine zu Ermgassen
Karol Zub
Haseeb Zubair
Ana Zubiaga
Arkaitz Zubiaga
Celia Zubrinich
Riccardo Zucchi
Gianna Zuccotti
Andreas Zucker
Nancy Zucker
Robert Zucker
Diana Zuckerman
Marc Zuckerman
Molly Zuckerman
Jessica Zucman-Rossi
Iker Zudaire
Johannes Zuegg
Konstantin Zuev
Marco A. L. Zuffi
Antonio Zuffiano
Inge Zuhorn
Daniel Zuj
Marlene Zuk
Leah L. Zullig
Tjerk Zult
Leo Zulu
Michael Zulyniak
Alfredo Zuniga
Gerardo Zúñiga
Alejandra Zuniga-Feest
J. Jaime Zúñiga-Vega
Wu Zunyou
Maria Victoria Zunzunegui
Bo Zuo
Jian Zuo
Li Zuo
Lijun Zuo
Rujuan Zuo
Siming Zuo
Yi Zuo
Yongchun Zuo
Zhentao Zuo
Mary Zupanc
Vesna Zupančič
Petra Zurbig
Nicole Zurcher
Robert Zurier
Gustavo Zurita
Jeannete Zurita
Michal Zurovec
Mateusz Zurowski

Mary Zutter
Susan Zvacek
Elika Zvesk
Alexander Zvoleff
Michail Zvyagintsev
Wendy Zwaanswijk
Marcel Zwahlen
Jaco Zwanenburg
Sara Zwart
Simon Portegies Zwart
Ellen Zwarthoff
Fried Zwartkruis
Larry Zweibel
Christiane Zweier
Virginia Zweigenthal
Robert Zweigerdt
David Zweiker
Andreas Zwergal
Samue Zwetchkenbaum
Janusz Zwiazek
Lech Zwierzchowski
Reyer Zwiggelaar
Norberto Zwirner
Cosmas Zyambo
Izabela Zych
Ramūnas Žydelis
Gayle Zydlewski
Theodoros Zygiridis
Krzysztof Żyła
Sarah Zylinski
Marek Żywicki
Abilash
Basappa
